# Precision medicine for atherosclerotic cardiovascular disease: Integrative genomics maps risk loci and AI‐predicted functional consequences

**DOI:** 10.1002/ctm2.70732

**Published:** 2026-07-10

**Authors:** Liwan Fu, Xiaodi Han, Qin Liu, Yuquan Wang, Yue‐Qing Hu

**Affiliations:** ^1^ Center for Non‐Communicable Disease Management Department of Neurology Beijing Children's Hospital, Capital Medical University, National Center for Children's Health Beijing China; ^2^ Department of Ultrasound Capital Center for Children's Health, Capital Medical University Beijing China; ^3^ State Key Laboratory of Genetic Engineering, Human Phenome Institute, Institute of Biostatistics, School of Life Sciences, Fudan University Shanghai China

**Keywords:** AI prediction, ASCVD, DCLRE1B, genomic SEM, rs11552449

## Abstract

**Background:**

Atherosclerotic cardiovascular disease (ASCVD) is a leading cause of global morbidity and mortality, but its genetic architecture remains incompletely understood. This study aims to uncover novel genetic insights into ASCVD through multivariate genomic analysis and molecular structure predictions.

**Methods:**

We analysed genomic data from over 3.8 million individuals across various ASCVD phenotypes, including coronary heart disease, stroke, transient ischemic attack, peripheral artery disease and abdominal aortic aneurysm. Advanced tools such as Genomic Structural Equation Modeling, fine‐mapping, FUSION, FOCUS and other functional annotation methods were applied to identify causal single nucleotide polymorphisms associated with ASCVD. Protein structural analysis was performed using AlphaFold3, and AI‐driven thermodynamic analysis (ThermoMPNN) assessed the stability and functional consequences of mutations.

**Results:**

Our analysis revealed 347 genome‐wide significant variants linked to ASCVD, distributed across 213 loci. Ninety of these variants were not identified in any of the five input GWAS datasets. Upon cross‑referencing with large‑scale external GWAS, 19 of the 90 variants showed no prior association with any cardiovascular or metabolic trait, 15 were previously reported only in risk factor GWAS, and 56 had been reported in direct ASCVD endpoint GWAS. The latter group includes the *DCLRE1B* rs11552449 missense mutation. Nevertheless, AI‑based structural and thermodynamic analyses revealed that this mutation (H61Y) disrupts DCLRE1B protein stability, increases conformational flexibility, and alters solvent‑accessible surface area—mechanistic insights that have not been previously described.

**Conclusions:**

This study provides a hypothesis‑generating genetic landscape of ASCVD, unveiling novel variants and their molecular impacts. These findings enhance our understanding of ASCVD mechanisms and may offer potential avenues pending experimental validation and targeted therapies in cardiovascular disease.

## Graphical abstract

- Genomic SEM of five ASCVD traits in > 3.8 million individuals identified 347 risk variants, including 19 novel loci.
- Integrating fine‐mapping and TWAS prioritized causal genes and revealed endothelial cell enrichment in vascular tissues.
- AI modelling shows the *DCLRE1B* mutation destabilizes the protein, impairing DNA repair and transcription.
- This integrative framework uncovers novel ASCVD mechanisms and supports precision cardiovascular medicine.

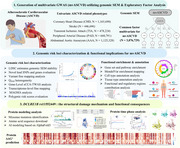

## 1 INTRODUCTION

Atherosclerotic cardiovascular disease (ASCVD), mainly encompassing coronary heart disease (CHD), stroke, transient ischemic attack (TIA), abdominal aortic aneurysm (AAA) and peripheral arterial disease (PAD), is a leading cause of morbidity and mortality worldwide, accounting for approximately 18.6 million deaths annually—nearly one‐quarter of all global deaths.^1^, ^2^ ASCVD is a progressive disorder characterized by lipid deposition, chronic inflammatory activation and endothelial dysfunction, shaped by intricate interactions among genetic predisposition, environmental exposures, and lifestyle factors.^3^, ^4^, ^5^, ^6^ With the continued acceleration of global population ageing, the incidence and burden of ASCVD are rising sharply, posing a critical challenge to medical research, public health and socioeconomic systems.^3^, ^7^ Despite substantial advances in lipid‐lowering therapy, anti‐inflammatory strategies and vascular regeneration, the precise genetic and molecular mechanisms underlying ASCVD remain incompletely understood. While dysregulated lipid metabolism, chronic inflammation and endothelial dysfunction are recognized as the key drivers, these factors fail to fully explain the substantial interindividual variability in disease susceptibility and clinical outcomes.^8^, ^9^

To address this gap, we conducted a genome‐wide association study (GWAS) targeting latent, unmeasured ASCVD phenotypes using genomic structural equation modelling (SEM).^10^ By integrating publicly available GWAS summary statistics from ASCVD‐related diseases,^11^, ^12^, ^13^, ^14^ this approach enabled estimation of SNP‐level associations with the latent ASCVD phenotype and identification of residual genetic components beyond known biomarker effects, providing a refined set of candidate genetic markers. This strategy mitigates confounding inherent in single‐biomarker‐based analyses and enhances the resolution of complex genetic signals.

Among the prioritized genes, *DCLRE1B* emerged as a candidate of interest due to its central role in genomic stability and post‐transcriptional regulation, with potential impacts on endothelial function, smooth muscle proliferation and inflammatory responses.^15^, ^16^ Although *DCLRE1B* variants have been associated with cardiovascular risk in genetic studies,^12^, ^17^ mechanistic evidence remains scarce. Leveraging AlphaFold3 structural prediction,^18^ ThermoMPNN stability assessment^19^ and molecular dynamics simulations, we systematically elucidated the structure–function relationships of key *DCLRE1B* variants. Our results demonstrate that these variants markedly alter protein stability and its interactions with nucleic acids and protein complexes, thereby perturbing downstream transcriptional and translational regulatory networks—offering structural biological insight into its potential role in ASCVD pathogenesis.

**FIGURE 1:**
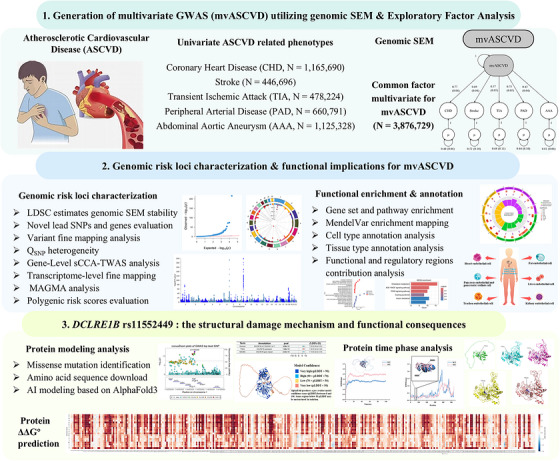
Flowchart illustration for this study. AI, artificial intelligence; GWAS, genome‐wide association study; LDSC, linkage disequilibrium score regression; MAGMA, multi‐marker analysis of GenoMic annotation; mvASCVD, the common factor model for atherosclerotic cardiovascular disease; sCCA‐TWAS, sparse Canonical Correlation Analysis‐based Transcriptome‐Wide Association Study; SEM, structural equation modelling; SNP, single nucleotide polymorphism.

By integrating genomic SEM‐based latent phenotype modelling with AI‐driven molecular structure and dynamics prediction, we identified 347 independent association signals across 213 loci, and performed multi‐scale, multi‐layered analyses of a key missense variant in *DCLRE1B*. We reveal how this variant perturbs protein stability and nucleic acid interactions, influencing transcriptional and translational regulation and potentially contributing to ASCVD development (Figure 1). This study establishes a methodological framework for dissecting multi‐layered mechanisms in complex diseases, while providing candidate targets and a theoretical basis for precision prevention, early diagnosis and targeted therapy in cardiovascular medicine.

## 2 METHODS

### 2.1 Univariate input GWAS data sources

The univariate GWAS summary statistics used as input for our multivariate GWAS of ASCVD were derived from five individual GWASs investigating closely related cardiovascular conditions: CHD,^13^ stroke,^11^ TIA, PAD^12^ and AAA.^14^ All contributing GWAS datasets were approved by their respective institutional review boards, included informed consent from participants and underwent stringent quality control procedures.

The CHD GWAS endpoint (*n* = 1 165 690; 46% female) encompassed both prevalent and incident CHD cases within the study populations.^13^ This GWAS meta‐analysis incorporated data from nine newly included cohorts not previously analysed in large‐scale CHD GWAS, and combined them with datasets from UK Biobank and the CARDIoGRAMplusC4D consortium. An inverse‐variance weighted meta‐analysis was performed, yielding 181 522 cases among 1 165 690 individuals of predominantly European ancestry.^13^ Stroke was defined according to the World Health Organization criteria, which describe it as a rapidly developing clinical syndrome characterized by focal (or global) cerebral dysfunction lasting more than 24 h or leading to death, with vascular causes.^11^ Summary statistics for stroke were obtained from a meta‐analysis of 17 cohorts with genome‐wide genotyping data imputed to the 1000 Genomes Project (Phase 1 v3 or equivalent), as part of the MEGASTROKE and related consortia. This dataset comprised 40 585 stroke cases and 406 111 controls of European descent.^11^ TIA summary statistics were sourced from the FinnGen Release 12 dataset (https://www.finngen.fi/en/access_results), which includes 24 948 individuals diagnosed with TIA and 478 245 controls, all of European ancestry. The mean age at DNA sampling in FinnGen was 51.8 years, and 56.3% of participants were female. TIA was defined as a transient episode of neurological dysfunction resulting from temporary cerebral ischemia, typically resolving within 24 h and not causing permanent infarction (https://www.finngen.fi/en/researchers/clinical‐endpoints).

PAD GWAS summary data were taken from a recent European ancestry‐focussed meta‐analysis that combined results from UK Biobank and FinnGen.^12^ The UK Biobank is a large population‐based prospective cohort that enroled approximately 500 000 participants across the UK, with a mean age of 56.8 years at recruitment and 53.8% female. PAD cases were identified using ICD‐10 codes, totalling 11 226 cases and 649 565 controls.^12^ AAA GWAS data were obtained from a large‐scale meta‐analysis conducted by the AAAgen Consortium, which included 14 cohorts (*n* = 1 125 328), such as UK Biobank, deCODE and ARIC.^14^ Detailed descriptions of cohort composition, recruitment strategies, case‐control definitions, genotyping platforms, imputation protocols and GWAS methodologies are available in the original publication.^14^ An overview of all univariate GWAS sources included in this study is provided in Table S1.

### 2.2 Quality control of univariate input GWAS

Quality control (QC) of the GWAS summary statistics was performed using a standard filtering criteria for summary‑level data. SNPs with ambiguous alleles, strand inconsistencies, or imputation INFO score < .6 were removed. In addition, the major histocompatibility complex (MHC) region, located on chromosome 6 and spanning approximately 25 000 000 to 35 000 000 base pairs (bp), was carefully considered. The MHC region is known for its structural and functional complexity, containing immune‐related genes that display significant genetic polymorphism. Due to this complexity, the MHC region necessitates specialized handling and analytical approaches in genetic studies. Given the potential confounding effects of polymorphisms in this region, it was excluded from the analysis prior to conducting SEM.

We did not perform additional SNP overlap visualization between input GWASs, as genomic SEM inherently accounts for sample overlap and differential SNP coverage through its multivariate LD score regression framework. To generate the multivariate summary statistics (mvASCVD), we followed the LD score regression defaults as recommended. This involved removing SNPs whose minor allele frequency (MAF) exceeded .01, because LDSC tends to overestimate standard errors when analysing low‑frequency variants. We also restricted our analysis to HapMap3 variants, using the 1000 Genomes Phase 3 European reference panel as the backbone. Both the genetic covariance matrix and the sampling covariance matrix were built exclusively from HapMap3 SNPs, ensuring reliable LDSC estimates. For the subsequent multivariate GWAS, we took all autosomal SNPs from the five input GWAS that met the standard QC criteria. These variants were then aligned to the 1000 Genomes Phase 3 EUR panel and further cleaned by applying several exclusion rules: SNPs with MAF < .01 (prone to genotyping errors and inflated LDSC standard errors), SNPs with effect sizes exactly zero (which would hinder matrix inversion in genomic SEM), SNPs not aligned with the reference panel, and SNPs with allele mismatches.

### 2.3 Sample overlap in univariate input GWAS

The univariate GWAS summary statistics used in our study came from several distinct genomic databases, each drawing from different sets of study participants. Consequently, during the GWAS process, we meticulously examined the overlap of samples between the cohorts to ensure the robustness and generalizability of the findings. This careful consideration also addressed the potential statistical implications arising from any sample overlap.

### 2.4 Genomic SEM

We performed a multivariate GWAS on CHD, stroke, TIA, PAD, and AAA using the genomic SEM framework^10^ as implemented in the GenomicSEM R package (v.0.0.5), with the goal of uncovering the shared genetic liability underlying these ASCVD‑related traits. This multivariate approach,^10^ described in detail in Table S1, allows flexible exploration of different models for the genetic architecture of complex traits. A key advantage is that it remains unbiased by sample overlap (e.g., when UK Biobank participants appear in multiple input GWASs) or unequal sample sizes. Moreover, it can distinguish variants that affect only a subset of the traits from those that influence the broad cross‑trait liability.^10^

The analysis consisted of two main phases. First, we estimated the empirical genetic covariance matrix and its corresponding sampling covariance matrix. To do so, we aggregated the ASCVD‑related GWAS summary statistics and applied the multivariate version of cross‑trait LD score regression,^10^, ^20^ which produced the empirical genetic covariance matrix among the five traits. This matrix then served as input for the common factor SEM (Table S3). Second, we specified an SEM that minimizes the discrepancy between the model‑implied covariance structure and the empirical matrix obtained in the first phase. Given our aim to capture a genetic signature common to all five ASCVD phenotypes, we tested a one‑factor model. Model fit was assessed using SRMR, χ^2^, AIC and CFI (Table S4). With the appropriate common factor specification, we incorporated individual autosomal SNP associations into the genetic and sample covariance matrices, generating a multivariate GWAS (termed mvASCVD) that represents the shared covariance across the five input GWASs. Ultimately, 3 914 020 SNPs present in all input datasets were retained in the multivariate summary statistics and used for the subsequent GWAS.

Model specification and dimensionality assessment. Before performing confirmatory factor analysis, we evaluated the suitability of the genetic covariance matrix for factor analysis using the Kaiser–Meyer–Olkin (KMO) measure. The overall KMO was 0.69, indicating moderate common variance among the five traits, which does not strongly favour a complex multi‑factor structure. Based on the genetic covariance matrix among the five traits and applying the maximum number of factors formula, we determined that the maximum number of plausible factors is two. Guided by the factor loadings from exploratory factor analysis, we then assigned specific traits to each factor: CHD and PAD loaded on Factor 1, while stroke and TIA loaded on Factor 2. Nevertheless, given our primary goal of discovering shared genetic signals across all five clinically relevant ASCVD phenotypes, we proceeded with a one‑factor common factor model including all five traits. Model fit was evaluated using CFI, SRMR, χ^2^ and AIC. As a sensitivity analysis (Table S22), we also tested a two‑factor model (CHD + PAD vs. stroke + TIA). Because the factor correlation was high (r ≈ 0.80) and the one‑factor model already met good fit criteria, we retained the one‑factor model as the primary, more parsimonious and comprehensive representation.

### 2.5 *Q*_SNP_ heterogeneity in genomic SEM

To examine whether the mvASCVD SNP associations are properly captured by the multivariate SEM framework, we computed the *Q_SNP_* heterogeneity statistic.^10^ Under the null hypothesis, the SNP associations observed in the single‑phenotype GWASs are fully explained by the mvASCVD model. Consequently, a significant *Q_SNP_* result would imply that a given SNP influences the individual ASCVD traits through pathways outside the shared genetic liability represented by mvASCVD. We applied a Bonferroni correction for the 347 lead SNPs, setting the significance threshold at *p* = 1.44 × 10^−4^ (0.05/347).

### 2.6 Multilevel assessment of genomic SEM

Beyond the goodness‑of‑fit measures described above, we also used a separate LDSC‑based approach to check the stability of our genomic SEM This involved inspecting several diagnostic parameters: the mean χ^2^, genomic inflation factor (λ_GC_), maximum χ^2^, heritability (h^2^), LDSC intercept and the attenuation ratio defined as (intercept – 1)/(mean χ^2^ – 1). When running LDSC, we deliberately kept certain SNPs that might ordinarily be filtered out: those with missing values, those with an INFO score below 0.9, and those with a MAF under 0.01. Only SNPs whose *p*‑values fell outside a plausible range or whose strand orientation could not be determined were excluded.

### 2.7 Defining genomic loci and determining novel variants

To pinpoint genomic loci and lead SNPs associated with mvASCVD at genome‑wide significance (*p* < 5 × 10^−8^), we used the FUMA platform^21^ (‘functional mapping and annotation of genetic associations’). We required that lead SNPs be in only weak linkage disequilibrium (*R^2^ *< .1). A locus was defined as a 250 kb window around a lead SNP plus any additional SNPs in high LD (*R^2^* > .6) with at least one independent variant. We first extracted summary statistics for these lead mvASCVD SNPs from the original univariate GWASs to gauge their association strength. To assess novelty, we compared our lead SNPs and loci with those from each univariate GWAS; a locus was considered novel if it lay more than 1 Mb away from any locus reported in the univariate data. We also queried the GWAS Catalog^22^ for previously reported associations (*p* < 5 × 10^−8^) to see if any of the 347 mvASCVD lead SNPs show pleiotropy. Using the same significance cutoff, we ran a risk locus analysis on the mvASCVD model within FUMA. The output was then fed into MAGMA (Multi‑marker Analysis of GenoMic Annotation),^23^ a post‑GWAS tool that aggregates SNP‑level signals into gene‑level associations. MAGMA's goal is to extract functional insights by evaluating how strongly each gene is linked to the phenotype, using a Bonferroni‑corrected threshold of *p* < 2.75 × 10^−6^ (0.05 divided by 18 205 tested genes). Finally, we proposed an additional strategy called the ‘GWAS Reduction Locus Approach’. This method compares the lead loci identified by genomic SEM against those found in the univariate GWASs, using the same genome‑wide significance threshold. By doing so, it helps uncover extra novel loci at lead positions that might otherwise be missed.

### 2.8 Fine mapping

To pinpoint the most plausible causal variants for mvASCVD, we applied both SuSIE^24^, ^25^ and FINEMAP^26^ as implemented in the R package echolocator^27^ v.2.0.3. For each lead SNP, we examined a ± 250 kb window and set a posterior probability threshold of 0.95 to define credible sets of candidate causal variants. Within echolocatoR, a ‘consensus SNP’ is one that is identified by both algorithms. The package then computes the average posterior probability and constructs an averaged credible set; this set is flagged as ‘consensus’ (value = 1) only when the mean SNP‑wise posterior probability from SuSIE and FINEMAP exceeds .95, otherwise it receives a value of 0. All fine‑mapping analyses used the 1000 Genomes Phase 3 European panel as the LD reference. The parameters (window size ± 250 kb, posterior probability threshold .95) were verified and no technical errors were present. Because of the polygenic architecture of ASCVD and complex LD structures, only a subset of loci yielded a variant with posterior probability > .95; this is consistent with previous large‑scale fine‑mapping studies of complex traits.

### 2.9 Transcriptome‐wide association study

After identifying potential causal variants, we carried out a transcriptome‑wide association study (TWAS) to prioritize genes that may be involved in mvASCVD. For this purpose, we used the FUSION TWAS framework,^28^ which relies on precomputed eQTL weights for 37 920 gene–tissue pairs from GTEx version 8. ASCVD is a systemic disease involving arteries, liver, adipose tissue and immune cells. Instead of restricting to a few presumed relevant tissues, we employed the sparse canonical correlation analysis (sCCA) implementation in FUSION, which integrates multi‑tissue expression data to identify cross‑tissue transcriptional programs associated with mvASCVD. This approach improves statistical power, accounts for shared genetic regulation across tissues, and has been successfully applied to other complex traits.^29^, ^30^ Our mvASCVD dataset included enough variants to analyse 36,149 out of the 37,920 features. Genes associated with mvASCVD that passed Bonferroni correction for multiple comparisons (*p*‐value  <  1.38  ×  10^−6^) were selected for further investigation, including fine mapping via the FOCUS method, specifically developed for TWAS.^31^ We prioritized ‘high‐confidence’ mvASCVD genes identified by FUSION based on additional supporting evidence for fine mapping. In line with prior research,^32^ we focussed on TWAS‐significant genes associated with mvASCVD that were likely to be causal, as indicated by a FOCUS posterior inclusion probability greater than 0.5.

### 2.10 Gene‐set and disease ontology enrichment

We conducted pathway enrichment analyses utilizing the Molecular Signatures Database (MsigDB)^33^ in conjunction with Gene Set Enrichment Analysis (GSEA) to explore potential associations between ASCVD and relevant pathways for the genes identified by MAGMA^23^ through FUMA gene‐to‐function. Furthermore, we examined the potential links between mvASCVD and Mendelian disease genes, as well as related pathways, using MendelVar.^34^

### 2.11 Cell‐type enrichment

To pinpoint cell types that may contribute to mvASCVD, we applied the cell‑type expression‑specific integration method for complex traits (CELLECT)^35^ together with single‑cell RNA‑seq data. The transcriptomic profiles came from the Tabula Muris project,^36^ which covers roughly 100 000 cells across 20 mouse organs and tissues. After normalizing and preprocessing these data with CELLEX,^35^ we derived expression specificity likelihood scores for each gene. Cell types were then labelled according to the naming system used in the LDSC software, and we set a false discovery rate (FDR) of 0.05 as the significance threshold.

### 2.12 Partitioning of SNP heritability

We employed stratified linkage disequilibrium score regression (S‐LDSC)^37^ to partition SNP heritability. SNPs were assigned to 53 distinct genomic annotation categories, including genes, enhancers, repressors and others. Heritability enrichment for each annotation was calculated as the ratio of the proportion of heritability attributed to SNPs within a given category to the proportion of total SNPs assigned to that category. Precomputed LD scores from the European panel of the 1kGP3 and publicly available annotation datasets were used. Statistical significance was determined using a Bonferroni‐corrected threshold for multiple testing across the 53 annotations, set at *p* < .05/53 = 9.43 × 10^−4^.

### 2.13 Polygenic risk score construction

We derived polygenic risk scores (PRS) using GWAS summary statistics to assess the genetic contributions of specific chromosomal regions to disease susceptibility. To compute the PRS, we implemented the PRS‐CS (Polygenic Risk Score‐Continuous Shrinkage) method,^38^ which integrates GWAS summary data with an external LD reference panel to infer posterior SNP effect sizes. PRS‐CS adopts a Bayesian regression framework that incorporates LD information from the reference panel, enabling continuous shrinkage of SNP effect estimates across the genome. By accounting for both LD structure and the statistical noise inherent in GWAS summary statistics, this approach enhances the precision and reliability of polygenic risk prediction. The PRS was built for descriptive purposes only, to evaluate the relative genetic contribution of different chromosomal regions to ASCVD susceptibility, and was not externally validated or intended for clinical risk prediction.

### 2.14 Identification of the amino acid substitution site resulting from the missense mutation

A missense mutation refers to a single nucleotide substitution that results in an amino acid change, which can potentially alter the structure and function of the encoded protein. From the 90 identified mvASCVD SNPs, we prioritized missense variants that were also significant in TWAS and MAGMA. Only *DCLRE1B* rs11552449 (Figure 5A,B) satisfied all three criteria and was therefore selected for in‑depth AI‑based protein modelling.

To determine the precise impact of the rs11552449 missense mutation on the DCLRE1B protein sequence, we conducted a site‐specific query using the Ensembl database (https://www.ensembl.org/) and the NCBI database (https://www.ncbi.nlm.nih.gov/). Our analysis revealed that this mutation occurs at the 61st amino acid position of the DCLRE1B protein. Specifically, a cytosine (C) is substituted with a thymine (T) at the DNA level, resulting in the replacement of histidine (His) with tyrosine (Tyr) at this position. Given that missense mutations frequently alter the structure and function of proteins—and considering that DCLRE1B is one of the evolutionarily conserved genes involved in interstrand crosslink (ICL) repair^15^—this amino acid substitution is likely to affect the function of the DCLRE1B protein. Therefore, confirming the exact location of this mutation and investigating its potential effects on protein structure and function is of critical importance.

### 2.15 The acquisition of the wild‐type DCLRE1B structure and download of the amino acid sequence

As a prerequisite for the subsequent modelling and simulation work, we needed the wild‑type DCLRE1B protein's three‑dimensional structure and its full amino acid sequence (Table S21). We therefore first consulted the GeneCards database to gather detailed information on the *DCLRE1B* gene, including its function, disease links and expression patterns. From this search we obtained the Ensembl identifier ENSG00000118655. Using this Ensembl ID and the UniProt resource (with the species set to *Homo sapiens*), we retrieved and downloaded the complete protein sequence under the UniProt accession Q9H816. The UniProt entry also provided an experimentally determined structural model. Examining this structural information helped us better understand the functional role of *DCLRE1B*, especially in interstrand crosslink (ICL) repair. The downloaded structural file subsequently served as the starting point for all protein dynamics simulations and analyses of the mutant form.

### 2.16 AI‐based modelling of *DCLRE1B* rs11552449 mutation using AlphaFold3

To model the structure of the mutant protein, we turned to AlphaFold3—a state‑of‑the‑art deep learning platform that delivers high‑accuracy three‑dimensional protein predictions by leveraging large‑scale biological data and neural network architectures. The model incorporates amino acid sequences, evolutionary signals, and physicochemical rules to produce reliable structural estimates. In contrast to conventional experimental methods like X‑ray crystallography or NMR, AlphaFold3 is substantially faster while maintaining comparable or even better accuracy. It is particularly well suited for capturing structural alterations caused by amino acid substitutions. We therefore submitted the amino acid sequence of the *DCLRE1B* mutant to the AlphaFold3 online server (https://alphafoldserver.com/). The server then automatically produced a predicted three‑dimensional structure of the mutant. We downloaded the resulting CIF (Crystallographic Information File) and used PyMOL (version 3.2, Educational Edition) to convert it into PDB (Protein Data Bank) format, allowing us to use the structure in further simulations and analyses. The pLDDT score (> 90) was used only as a quality filter to ensure a reliable starting structure for downstream molecular dynamics simulations, not as evidence of functional accuracy.

### 2.17 In vivo simulation of DCLRE1B protein

With the three‑dimensional structures of both wild‑type and mutant DCLRE1B in hand, we ran molecular dynamics (MD) simulations using GROMACS 2024.5^39^ to examine the protein's dynamic behaviour and stability in aqueous solution. To speed up the computations, we employed an NVIDIA GeForce RTX 4090 GPU, whose parallel processing capabilities greatly accelerate simulations of large protein‑water systems—especially over long timescales. The simulations ran on Ubuntu 24.04 LTS, a stable operating system well suited for large‑scale parallel tasks. We chose the AMBER14SB force field to parameterize DCLRE1B, as it accurately model's interactions between amino acid residues, and the TIP3P water model to describe solvent behaviour. The system was built by generating a topology file with the pdb2gmx tool, placing the protein in a cubic box with a minimum distance of 1.0 nm to the box edges, and then adding water molecules with the solvate command. Na^+^ and Cl^−^ ions were introduced via the genion tool to achieve electrical neutrality and a physiological ionic strength of 0.15 M NaCl. Energy minimization was performed with the steepest descent algorithm for 1000 steps (step size .01 nm) to remove unrealistic contacts and distortions. The system was then equilibrated for 100 ps under constant temperature and volume (NVT), followed by another 100 ps under constant temperature and pressure (NPT). After equilibration, a 100 ns production MD run was carried out under NPT conditions at 300 K and 1.0 atm, with a 2 fs time step and hydrogen bond lengths constrained by the SHAKE algorithm. The resulting trajectory files were saved for subsequent analysis and visualization.

### 2.18 Prediction of free energy changes (ΔΔG°) in *DCLRE1B* rs11552449 mutation using the AI‐based ThermoMPNN model

To evaluate how the *DCLRE1B* rs11552449 mutation affects protein thermal stability and, consequently, its function and structural integrity, we used the ThermoMPNN tool^19^ to predict the change in free energy (ΔΔG°). ThermoMPNN (Thermodynamic Mutant Selection Neural Network) is a deep‑learning model that forecasts the thermodynamic outcome of point mutations by combining protein structural data, mutation type, and experimentally measured thermostability information. It relies on graph neural networks and transfer learning, with a design specifically aimed at stability changes from single‑amino‑acid substitutions. The model comprises several functional components: a ProteinMPNN embedding extractor that obtains structural features for each residue, a graph neural network module that captures spatial relationships among residues, a lightweight attention mechanism that weighs local structural context, and a multi‑layer perceptron output layer that computes the ΔΔG° difference between wild‑type and mutant. By analysing the amino acid sequence and mutation data, ThermoMPNN predicts how thermostability and structure may change at elevated temperatures, helping to determine whether a given substitution weakens or strengthens the protein—a valuable guide for subsequent computational studies.

We ran ThermoMPNN on the Google Colab platform, which offers GPU‑accelerated computing well suited for machine learning and bioinformatics tasks. After setting up the environment by installing the required libraries and loading pre‑trained models, we uploaded the PDB file of the *DCLRE1B* rs11552449 mutant—containing its three‑dimensional coordinates—as the main input. The GPU resources in Colab were essential to handle the model's computational load, greatly speeding up the prediction. Using the default parameters and the PyTorch implementation, ThermoMPNN then generated outputs including the predicted melting temperature, a thermostability score and an assessment of the mutation's likely structural impact on the DCLRE1B protein.

### 2.19 In silico knockout analysis of *DCLRE1B* in endothelial cells

To functionally assess the role of *DCLRE1B* in endothelial biology, we performed an in silico knockout using the scTenifoldKnk framework.^40^ This method constructs a single‑cell gene regulatory network from wild‑type control samples and then virtually deletes the target gene by setting its outgoing edges to zero, allowing prediction of differentially expressed genes and pathway perturbations. We applied scTenifoldKnk to publicly available single‑cell RNA‑sequencing data from human skin‑derived endothelial cells obtained from Medical University of Vienna.^41^ The analysis was performed using default parameters as described in the original publication.^40^ Differentially expressed genes were identified using an FDR threshold of < .05. GSEA was conducted on the KEGG pathway database to identify enriched biological pathways following *DCLRE1B* knockout.

## 3 RESULTS

### 3.1 Structural equation modelling

**FIGURE 2:**
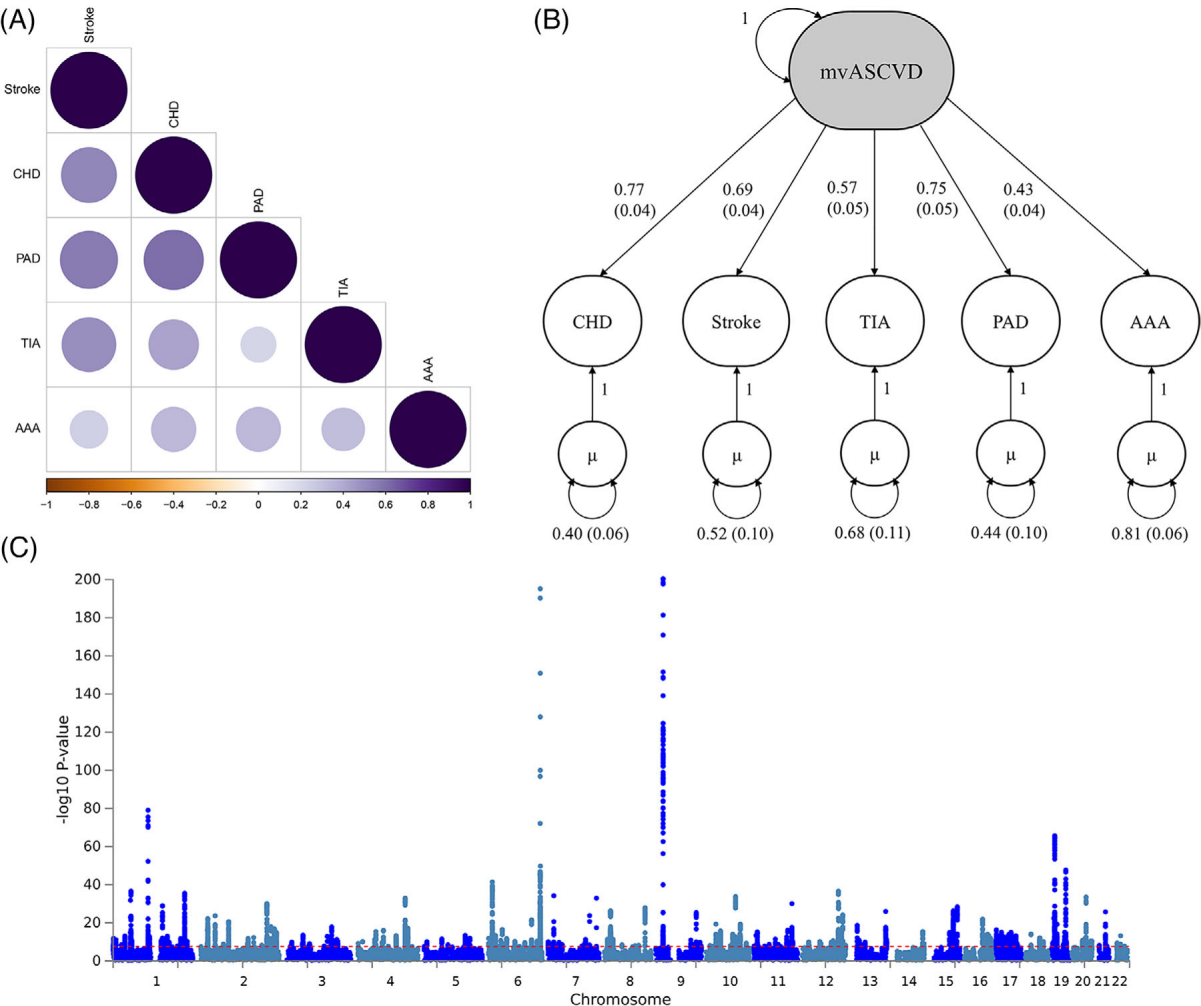
Multivariate ASCVD GWAS modelled with genomic SEM. (A) Genetic correlations for SEM with genomic SEM, presenting pairwise LD score genetic correlation estimates for the five univariate phenotypes. (B) Path diagram of the common factor model estimated with genomic SEM, with standardized factor loadings (standard error in parentheses). (C) Manhattan plot showing SNP associations (‐log_10_(*p* value)) with mvASCVD, ordered by chromosome. The red dashed line indicates the threshold for conventional genome‐wide significance (*p *= 5 × 10^−8^). CHD, Coronary Heart Disease; TIA, Transient Ischemic Attack; PAD, Peripheral Arterial Disease; AAA, Abdominal Aortic Aneurysm; mvASCVD, the common factor model for Atherosclerotic Cardiovascular Disease. *p* values are derived from two‐sided Wald tests for each SNP on mvASCVD. *μ* reflects the residual variance in the genetic indicators for the input univariate ASCVD related GWASs not explained by the mvASCVD common factor.

LD score regression analysis revealed a positive correlation among the five traits (Figure 2A; Tables S1 and S2). Before factor analysis, we assessed the suitability of the data. The Kaiser–Meyer–Olkin measure of sampling adequacy was .69, indicating that the data are suitable for factor analysis. Based on the genetic covariance matrix among the five traits and applying the maximum number of factors formula, we determined that the maximum number of plausible factors is two. Guided by the factor loadings, we assigned CHD and PAD to Factor 1 and stroke and TIA to Factor 2; AAA did not load strongly on either factor. Analysis with the two‑factor model (Factor 1: CHD+PAD; Factor 2: stroke + TIA; AAA excluded) showed slightly better fit (CFI = .98, SRMR = .051) but with only 1 degree of freedom (χ^2^(1) = 9.13, *p* = .0025). The correlation between the two factors was very high (*r* = .798), indicating substantial shared genetic variance. Given the moderate KMO (.69), the satisfactory fit of the one‑factor model, and the loss of AAA in the two‑factor solution, we retain the one‑factor mvASCVD as our primary model. The one‑factor common factor model nevertheless demonstrated good fit (comparative fit index (CFI)  =  .97, standardized root mean square residual (SRMR)  =  .067) (Figure 2B; Tables S3 and S4), providing strong evidence for a shared genetic factor, termed mvASCVD. Subsequently, we expanded the SEM framework to include individual variants, yielding a multivariate GWAS that identified 3 914 019 associations at the single nucleotide polymorphism (SNP) level for the shared ASCVD factor, mvASCVD.

### 3.2 Evaluation of SEM model stability via LDSC

We used LDSC with strict parameter controls, which led to the removal of 3 328 635 SNPs. Among the 585 384 retained SNPs, the mean chi‐square was 1.875, the genomic inflation factor Lambda GC was 1.498 (Figure S2), the maximum chi‐square reached 913.763, the heritability was 0.1308 (SE = .0065), and the LDSC intercept was 1.0078 (SE = .0142). These values indicate a reasonably well‑fitting model with only minor population stratification or residual confounding. The attenuation ratio (.0089, SE = .0162) suggests that confounding factors have a modest influence, while genetic effects dominate.

### 3.3 Novel lead SNPs and genes

Analysing 3 914 019 SNPs from the generated mvASCVD by genomic SEM, 8652 significant variants detected, we eventually identified 347 lead SNPs in 213 genomic loci (*p* value < 5 × 10^−8^) (Figure 2C; Tables S5 and S6). We displayed the locuszoom of the 213 genomic loci in Extended Data 1 (Supporting Information). Ninety of the 347 SNPs were not genome‑wide significant in any of the five input GWAS datasets underlying mvASCVD (Tables S6 and S7), highlighting the increased power of genomic SEM. To assess true novelty in the context of the broader literature, we cross‑referenced these 90 variants against large‑scale external GWAS resources, including the UK Biobank, Million Veteran Program (MVP), CARDIoGRAMplusC4D, MEGASTROKE, the Global Lipids Genetics Consortium (GLGC), the International Consortium for Blood Pressure (ICBP) and the GWAS Catalog. Using a significance threshold of *p* < 5×10^−8^, we classified the variants into three tiers (Table S23). Tier 1 (genuinely novel, *n* = 19): Variants with no significant association with any cardiovascular disease endpoint or cardiometabolic risk factor in any of the external databases. These represent true discoveries enabled by genomic SEM. Tier 2 (risk‑factor only, *n *= 15): Variants previously reported only in GWAS of intermediate traits (e.g., lipids, blood pressure, BMI, type 2 diabetes) but not in direct ASCVD endpoint GWAS (Table 1; Tables S8 and S9). For these, our study provides new evidence of their involvement in shared ASCVD liability. Tier 3 (previously reported in ASCVD endpoints, *n *= 56): Variants that had been associated with CHD, stroke, PAD, or AAA in prior large‑scale GWAS.^13^ Gene‐based analysis using MAGMA highlighted a total of 471 (Table S10) potential ASCVD related genes were identified as significantly controlled by the whole genome (Bonferroni‐adjusted *p* < .05). The corresponding gene‑based Manhattan plot is shown in Figure S3.

**TABLE 1:** Novel lead SNPs identified in mvASCVD.

SNP	CHR	BP	NEA	EA	MAF	*P*	Beta	SE	Nearest Gene	Q_pval_
rs1019307	7	12251790	C	G	.4036	1.80E‐09	‐.015	.002	TMEM106B	.59
rs10214652	6	161366947	A	G	.0696	2.89E‐12	‐.036	.005	RP3‐428L16.1	.18
rs10741066	10	25075370	T	C	.3330	2.29E‐08	.015	.003	PRTFDC1	.43
rs10919134	1	169286785	A	G	.4404	2.45E‐13	.018	.002	NME7:RP4‐800F24.1	.60
rs10928241	2	145831428	T	C	.3231	3.87E‐12	‐.019	.003	TEX41	.03
rs111246576	5	137633001	T	C	.1670	4.88E‐08	.017	.003	CDC25C	.49
rs111352470	12	133761664	A	G	.2406	1.12E‐08	‐.017	.003	ZNF268; CTD‐2140B24.4	.27
rs11167260	20	33775200	A	G	.0875	3.58E‐11	.026	.004	EDEM2	.05
rs11206803	1	56877509	T	C	.4722	2.00E‐12	‐.017	.002	RP4‐710M16.2	.01
rs11244061	9	136153981	T	C	.1133	3.78E‐10	‐.025	.004	ABO	.01
rs112484789	14	103919097	T	C	.1173	1.85E‐09	‐.024	0.004	MARK3	0.27
rs113970872	10	104778812	T	C	.0895	1.54E‐14	.033	0.004	CNNM2	0.01
rs11552449	1	114448389	C	T	.1909	4.06E‐13	.023	0.003	DCLRE1B	0.09
rs11613352	12	57792580	T	C	.1938	2.37E‐09	.017	0.003	R3HDM2	0.03
rs11695624	2	144177167	A	T	.1571	1.91E‐09	.020	0.003	ARHGAP15; AC096558.1; RP11‐570L15.2	0.08
rs1177562	11	118949331	T	C	.3618	1.28E‐10	‐.016	.003	VPS11	.01
rs12260962	10	82262826	T	C	.2992	2.18E‐14	.020	.003	TSPAN14	.02
rs12442575	15	78995488	A	G	.0746	6.23E‐12	‐.032	.005	CHRNB4; RP11‐160C18.2	.15
rs12555241	9	139387676	A	G	.2555	2.58E‐08	.016	.003	NOTCH1	.19
rs12589575	14	100122731	T	G	.1600	8.71E‐16	.027	.003	HHIPL1	5.36E‐11
rs12941550	17	17753828	A	C	.4095	2.07E‐15	.022	.003	TOM1L2	.10
rs13003675	2	233584109	T	C	.2853	3.15E‐15	‐.020	.003	GIGYF2	.51
rs1363465	5	128001170	T	G	.2644	9.52E‐14	‐.020	.003	SLC27A6; CTC‐573M9.1	.07
rs1429137	4	148282109	T	C	.1561	2.17E‐19	.028	.003	MIR548G	.82
rs145542470	9	21953099	A	G	.0199	6.20E‐12	‐.069	.010	RP11‐145E5.5	.20
rs148812085	2	203877233	T	C	.1302	1.98E‐30	‐.046	.004	WDR12	1.04E‐24
rs1510758	8	25061807	A	G	.2316	2.73E‐09	‐.017	.003	DOCK5	.62
rs17263917	5	9552338	A	G	.1402	1.72E‐10	.022	.004	SNHG18	2.89E‐04
rs185244	3	138092889	T	C	.1511	3.19E‐18	‐.029	.003	MRAS	.02
rs1870635	10	44480694	T	C	.3360	3.95E‐16	‐.021	.003	LINC00841	6.51E‐07
rs1883711	20	39179822	C	G	.0398	4.22E‐10	‐.046	.007	SNORD112	.009421
rs1970014	9	110517130	A	C	.2833	2.48E‐09	.016	.003	AL162389.1	6.87E‐05
rs1982072	19	41864509	A	T	.3111	7.21E‐12	.018	.003	CTC‐435M10.3; TMEM91; B9D2	3.80E‐04
rs2013694	19	8616392	T	C	.1461	1.41E‐08	.023	.004	MYO1F	.19
rs2077750	6	35089811	A	G	.1819	3.08E‐08	.017	.003	TCP11	.69
rs2306363	11	65405600	T	G	.1988	7.80E‐12	.021	.003	SIPA1	.02
rs2476602	1	114396955	A	G	.2256	2.52E‐10	.017	.003	PTPN22	.45
rs2493296	1	3327032	T	C	.1620	3.62E‐12	‐.025	.004	PRDM16	.31
rs249760	5	141915692	T	C	0.2058	5.54E‐09	‐.017	.003	AC005592.2	.19
rs2504927	6	160860430	T	C	.4344	1.97E‐19	.022	.002	SLC22A3	4.30E‐04
rs2672592	10	124230750	T	G	.3549	1.62E‐08	.014	.003	HTRA1	2.63E‐05
rs28399637	19	45324138	A	G	.3221	7.10E‐10	‐.017	.003	BCAM	.76
rs2909217	17	66463985	T	C	.2048	5.80E‐09	‐.017	.003	RP11‐120M18.2	.48
rs2920150	11	10296653	T	C	.2127	1.19E‐09	‐.019	.003	SBF2	.10
rs34232196	1	55489542	T	C	.2336	3.50E‐23	.029	.003	BSND	.81
rs34452617	9	21874553	A	G	.0895	9.90E‐13	‐.033	.005	MTAP; RP11‐145E5.5	.13
rs34894639	3	135798658	T	C	.2376	5.63E‐13	.021	.003	PPP2R3A	.04
rs35056688	6	12955104	T	C	.2425	7.98E‐18	‐.025	.003	PHACTR1	.06
rs35976034	19	17832748	T	G	.4245	1.23E‐14	‐.019	.002	MAP1S	.54
rs3778231	6	161672625	A	G	.0805	1.38E‐12	‐.029	.004	AGPAT4	.15
rs3798945	6	161685583	T	C	.4374	2.03E‐08	.014	.002	AGPAT4	.61
rs3846663	5	74655726	T	C	.3897	7.89E‐09	−014	.002	HMGCR	4.57E‐04
rs4418728	10	94839724	T	G	.4871	1.36E‐09	.014	.002	CYP26A1	7.33E‐03
rs4504897	1	151766851	A	T	.1412	1.53E‐09	‐.019	.003	RP11‐98D18.9	.03
rs4766578	12	111904371	A	T	.4771	5.76E‐37	.032	.003	ATXN2	4.05E‐04
rs4800401	18	20003625	T	C	.4046	1.68E‐14	‐.019	.002	RP11‐863N1.4	.17
rs4846913	1	230294715	A	C	.3996	1.15E‐10	.016	.002	GALNT2	.16
rs489693	18	57882787	A	C	.3221	1.37E‐10	‐.016	.003	RP11‐795H16.2	.05
rs4977733	9	21840777	A	G	.4095	1.67E‐16	.020	.002	MTAP; RP11‐145E5.5	.79
rs5017040	10	124028560	C	G	.3469	5.43E‐09	‐.015	.003	BTBD16	.39
rs55716353	19	11112153	A	T	.3519	1.43E‐13	.021	.003	SMARCA4	.41
rs56408342	8	22048490	A	G	.0696	5.15E‐09	‐.032	.005	BMP1	6.08E‐03
rs57301765	7	19052733	A	G	.1690	1.41E‐34	‐.038	.003	TWIST1	2.61E‐04
rs589985	13	110819586	A	G	.3221	1.80E‐09	.015	.003	COL4A1	.50
rs6003988	22	24295388	A	G	.2942	3.41E‐08	‐.016	.003	AP000350.8	.27
rs6102343	20	39924279	A	G	.1998	7.21E‐12	‐.019	.003	ZHX3	.97
rs616381	2	45891708	A	G	.4384	4.70E‐08	‐.013	.002	PRKCE	.70
rs61871680	10	124070455	A	G	.1720	1.04E‐09	.020	.003	BTBD16	.26
rs67651018	16	88527222	A	G	.2942	8.69E‐10	.016	.003	ZFPM1	.02
rs688359	6	160465291	A	G	.3807	8.42E‐15	.021	.003	IGF2R	0.04
rs6999158	8	19928013	A	T	.3062	6.86E‐14	.020	.003	AC100802.3	.08
rs7099085	10	75735804	T	C	.1978	1.78E‐08	‐.017	.003	VCL	1.63E‐03
rs7138688	12	112978204	T	G	.1183	3.12E‐10	.029	.005	RPH3A	.01
rs71646019	1	59433354	T	C	.2137	3.67E‐11	‐.020	.003	PHBP3	.04
rs72675569	1	56626575	T	C	.3499	2.16E‐09	‐.015	.003	RP1‐158P9.1	.97
rs72689147	4	156639888	T	G	.2008	1.86E‐17	.027	.003	GUCY1A3	3.13E‐04
rs73045269	19	41825191	T	C	.1551	2.03E‐15	‐.028	.003	TGFB1; CCDC97	2.82E‐03
rs73596816	6	161017363	A	G	.0288	1.96E‐19	‐.065	.007	LPA	.13
rs7440763	4	156433520	T	G	.1153	1.88E‐13	.027	.004	RP13‐487K5.1	7.38E‐03
rs7766436	6	22598259	T	C	.2863	9.74E‐14	‐.020	.003	RP1‐309H15.2	.69
rs77902220	13	29031238	C	G	.0706	9.05E‐10	.031	.005	FLT1	.22
rs78310134	13	110954922	A	T	.0706	8.29E‐09	‐.030	.005	COL4A1	.05
rs80056186	6	57158766	A	T	.0736	7.31E‐12	‐.034	.005	PRIM2	5.54E‐03
rs8108474	19	46301479	T	C	.3280	7.21E‐11	‐.017	.003	RSPH6A	.09
rs896848	8	95981810	A	G	.1879	1.97E‐08	‐.017	.003	NDUFAF6	.61
rs9319429	13	28973703	T	C	.3091	4.11E‐11	‐.017	.003	FLT1	.05
rs9337951	10	30317073	A	G	.3489	1.14E‐19	‐.025	.003	KIAA1462	4.83E‐06
rs9684467	4	54582132	A	G	.1700	4.15E‐08	.018	.003	FIP1L1; RP11‐317M11.1	.42
rs983817	17	59222069	T	C	.1928	3.25E‐12	.022	.003	BCAS3	.23
rs9943599	11	9752741	T	C	.4056	1.33E‐15	‐.020	.002	SWAP70	6.32E‐03

*Note*: Lead SNPs were defined as novel if they were > 1Mb from previously identified loci in the univariate ASCVD related GWASs comprising the mvASCVD. Q_SNP_ heterogeneity statistics, statistic of χ^2^ distributed, evaluated whether the multivariate SNP associations are appropriately modeled through a multivariate framework (Q_pval_ sourced two‐sided test and passed Bonferroni corrected). Because the null hypothesis of the Q_SNP_ test is that the SNP associations on the univariate GWASs are statistically mediated by the resultant multivariate GWAS, significant Q_SNP_ tests in the multivariate GWAS summary statistics suggest that the SNP impacts the univariate GWASs by pathways other than mvASCVD (see ‘Methods’ and Supporting Information).

Abbreviations: BP, position; CHR, chromosome; EA, effect allele; MAF, minor allele frequency; NEA, non‐effect allele.

### 3.4 Fine mapping

**FIGURE 3:**
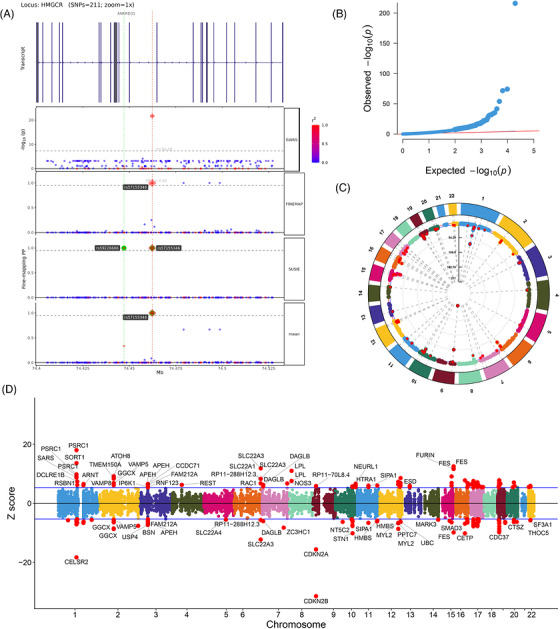
Fine mapping results for HMGCR from mvASCVD and plots for TWAS. (A) Fine mapping results of HMGCR with strong associations (PP > 0.95) identified by FINEMAP. (B) QQ plot for the results of sCCA‐TWAS. (C) Circular Manhattan plot results from sCCA‐TWAS analysis for mvASCVD. (D) Manhattan plot of the TWAS Z‐scores for mvASCVD. The *x*‐axis represents chromosomes, and the *y*‐axis displays the *Z*‐scores. The horizontal blue lines mark the absolute *Z*‐score value of 7, which represents the threshold for significance. mvASCVD, the common factor model for Atherosclerotic Cardiovascular Disease; sCCA‐TWAS, sparse Canonical Correlation Analysis‐based Transcriptome‐Wide Association Study.

For each of the 347 lead SNPs, we took a ± 250 kb window around the index variant and performed fine‑mapping using SuSIE and FINEMAP. We identified 26 variants with a posterior probability > .95 (putative causal). These reside on several chromosomes: 1 (rs12729727 in the *HORMAD1* locus, rs12030407 in the *PPAP2B* locus), 2 (rs77723314 in the *AC019055.1* locus, rs2230115 in the *PRKAG3* locus), 3 (rs1122525 in the *ARHGEF26* locus, rs1279086 in the *PPP2R3A* locus), 4 (rs57010886 in the *HGFAC* locus), 5 (rs57155346 in the *HMGCR* locus, rs72720042 in the *PAPD7* locus), etc. Specific characteristics of these SNPs are displayed in Table S11. Regional association plots show clear peaks at these loci, with additional credible‑set variants also supporting the associations (Figure 3A, Extended Data S2).

### 3.5 Q_SNP_ heterogeneity

The Q_SNP_ heterogeneity test assesses whether a SNP's associations with the five input traits are adequately captured by the common mvASCVD factor. A significant Q_SNP_ would suggest that the SNP influences the individual traits through pathways outside the shared ASCVD liability. Of the 347 lead SNPs, 52 exceeded the Bonferroni‑corrected threshold (*p* < 1.44 × 10^−4^ = .05/347). Among the 90 loci not significant in any input GWAS, six showed Q_SNP_ *p* < 1.44 × 10^−4^ (Table 1, Table S6). This suggests that these variants may influence the five ASCVD phenotypes through pathways beyond the shared mvASCVD factor, possibly reflecting trait‑specific pleiotropic effects (e.g., affecting only stroke or only lipid metabolism). The results indicated that the 84 newly reported loci impact five input ASCVD phenotypes via mvASCVD.

### 3.6 Transcriptomic imputation

We next ran a TWAS with FUSION^28^ to find genes whose predicted expression is associated with mvASCVD. The QQ plot for the sCCA‑based TWAS is displayed in Figure 3B. After Bonferroni correction (0.05/37 917, threshold *p *< 1.32 × 10^−6^), 289 genes remained significant (Figure 3C,D; Table S12). Fine‑mapping of these signals with FOCUS (posterior inclusion probability > .8) produced 332 genes (Table S13). Intersecting the FUSION‑significant and FOCUS‑supported lists gave 61 ‘high‑confidence’ genes (Table S14). Among them, PSRC1 showed the strongest positive association, with the most extreme test statistic. Other positively linked genes include *FES*, *SLC22A3* and *LPL*. In contrast, *CDKN2B* (and to a lesser extent *CDKN2A*) exhibited strong negative associations, implying that lower expression of these genes might increase ASCVD risk.

### 3.7 Pathway, cell type and tissue enrichment

**FIGURE 4:**
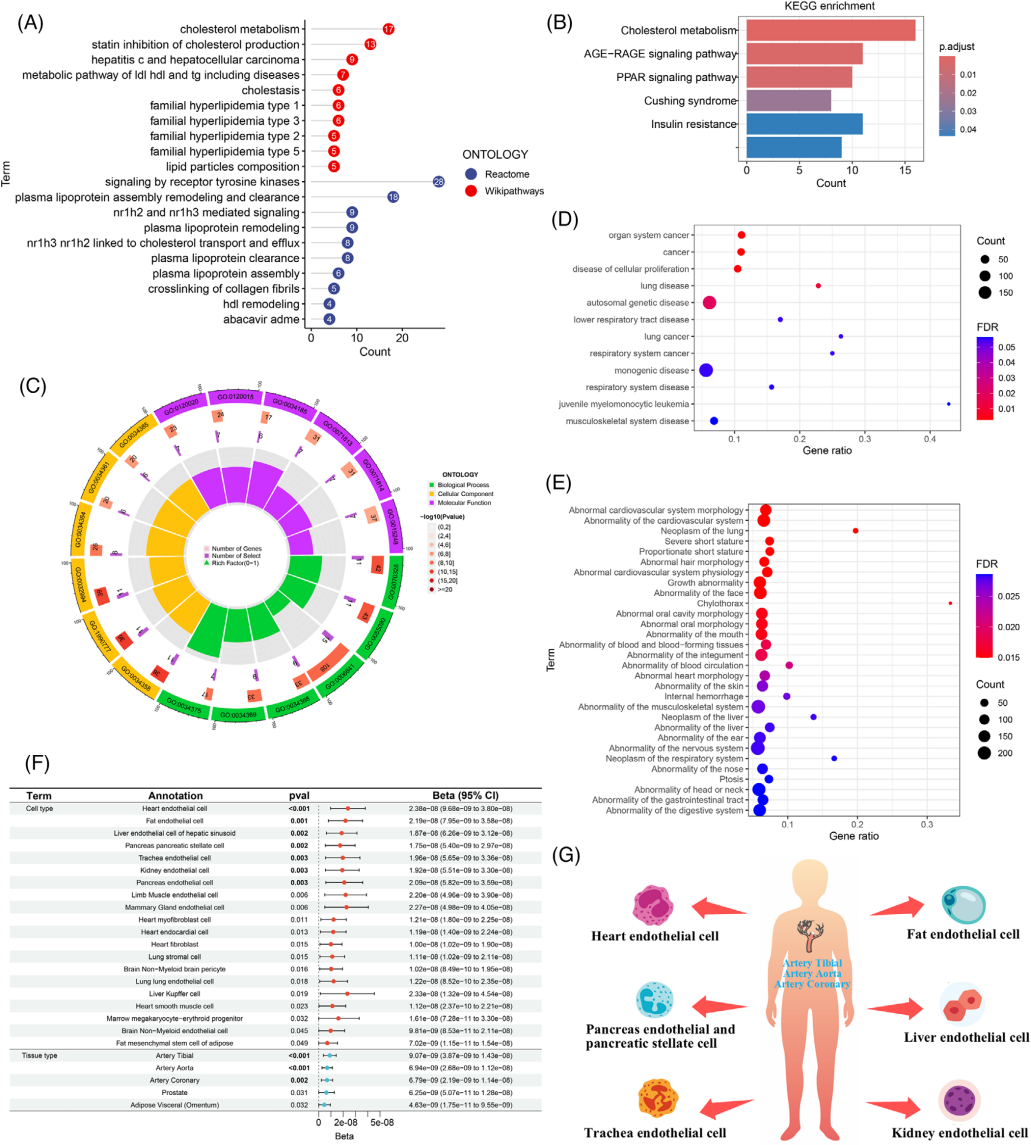
Pathways, cell type and tissue enrichment of the identified genes. (A) Lollipop plot for the identified genes involved in the pathways for Reactome and Wikipathways. (B) Bar plot for the significant pathways by the KEGG enrichment. (C) Circulate plot for the significant pathways by GO analysis. (D) Bubble plot of the Mendelian disease enrichment. (E) Bubble plot of the Mendelian pathway enrichment. (F) Forest plot of the cell‐type and tissue‐type enrichment. (G) Schematic diagram of the tissue‐type and cell‐type enrichment. FDR, false discovery rate; GO, gene ontology; KEGG, Kyoto encyclopedia of genes and genomes.

Gene‐based mapping via the multimarker analysis of genomic annotation (MAGMA)^23^ identified 471 genes (Table S10), which were subsequently used in our gene‐set analysis. These genes were significantly enriched for curated gene sets, canonical pathways, Gene Ontology, WikiPathways and Reactome terms (Figure 4A–C; Table S15). Many of the enriched gene sets were related to lipid processes, such as plasma lipoprotein assembly, cholesterol metabolism, high‐density lipoprotein particle remodelling, apolipoprotein binding and acylglycerol homeostasis. Additionally, the associations for these genes extended to ASCVD risk factors, including hypertension, lipid metabolism, body mass index and metabolic syndrome, as well as neuropsychiatric conditions like brain morphology and schizophrenia.

Cell‐type enrichment analysis revealed that six cell types passed the correction for multiple comparisons (Figure 4F,G; Table S16). The top three cell types were heart endothelial cells, fat endothelial cells, and liver endothelial cells in the hepatic sinusoid. Tissue‐type enrichment analysis identified three tissues (tibial artery, aortic artery and coronary artery) as significantly enriched after correction for multiple comparisons (Figure 4F,G). Notably, mvASCVD showed a strong enrichment in endothelial cells, with 11 of 20 cell types exhibiting *p* values  <  .05 associated with endothelial cells. Further enrichment analysis of mvASCVD in Mendelian disease genes and related pathways^34^ identified twelve Mendelian diseases, including various cancers and musculoskeletal disorders (Figure 4D; Table S17). Additionally, 82 phenotypic abnormalities were found to be enriched, including gene sets linked to cardiovascular and musculoskeletal function (Figure 4E; Table S18).

### 3.8 Heritability enrichment via genomic functional and regulatory regions

S‑LDSC enrichment analysis (Table S19) revealed that heritability is significantly concentrated in epigenetic regulatory regions and core functional gene regions. Coding sequences, evolutionarily conserved enhancers, and DNase I hypersensitive sites (DGF) showed the strongest positive contributions. This pattern suggests that these regulatory elements influence complex traits mainly through cis‑regulatory networks. Collectively, these results emphasize that cis‑regulatory variation—especially within enhancers and conserved elements—may be a key driver of ASCVD susceptibility and its multimorbid presentation.

### 3.9 Construction of polygenic risk scores from summary data

Our analysis demonstrates that the variants in our PRS are strongly associated with disease onset risk, and that the genetic contribution to disease susceptibility varies significantly across chromosomal regions (Table S20). Considering all SNPs, chromosomes 1 and 3 contributed the most positive genetic variance. When we restricted to the top 1000 SNPs by effect size, chromosomes 6 (102 SNPs) and 1 (93 SNPs) dominated; in the top 10 000, chromosomes 1 (916 SNPs) and 2 (817 SNPs) were most frequent. This rank‑dependent shift indicates that different genomic regions—and the genes/regulatory elements they harbour—contribute to susceptibility at different effect‑size tiers. We emphasize that this PRS has not been validated in an independent cohort; therefore, the observed chromosomal contributions should be interpreted as descriptive and hypothesis‑generating.

### 3.10 Confidence analysis of modelling results

**FIGURE 5:**
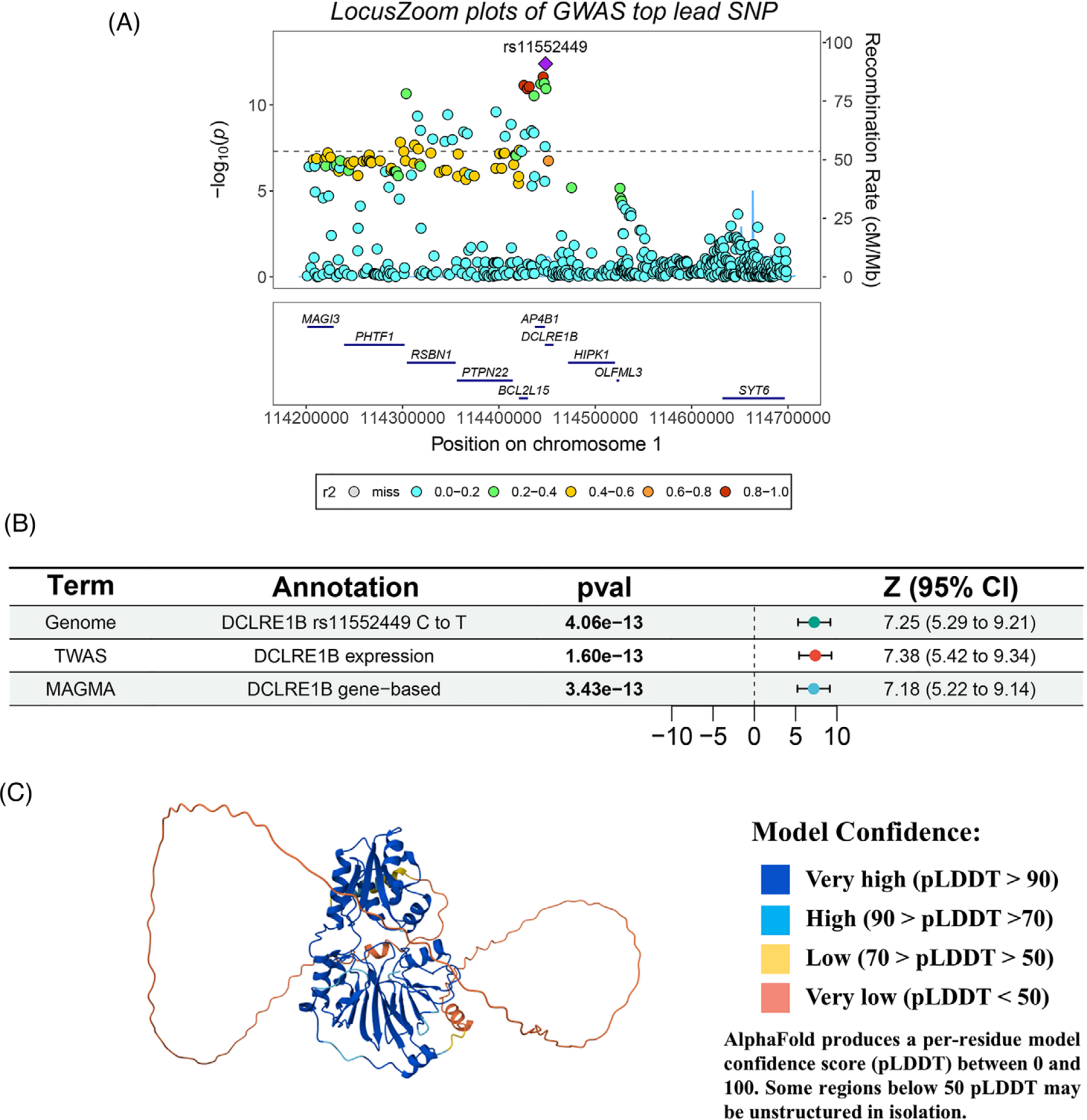
Genetic and protein information diagram of the missense variant DCLRE1B rs11552449 associated with mvASCVD identified via genomic Structural Equation Modeling. (A) LocusZoom plots of the missense variant DCLRE1B rs11552449. (B) Forest plots showing the effects of the missense variant DCLRE1B rs11552449, DCLRE1B transcript expression, and gene‐based associations. (C) Protein structural diagram of the missense variant DCLRE1B rs11552449 predicted by the AlphaFold3 model. CI, confidence interval; MAGMA, multi‐marker analysis of GenoMic annotation; pLDDT, predicted local distance difference test; TWAS, transcriptome‐wide association study.

Among the 90 identified mvASCVD SNPs, *DCLRE1B* rs11552449 was the only missense variant that achieved genome‑wide significance in mvASCVD and additionally passed the significance thresholds in both TWAS and MAGMA. This multi‑level genetic evidence justified its selection for downstream structural analysis. The genomic location and genetic association of this missense variant are shown in Figure 5A,B. We note that the TWAS association reflects genetically predicted expression levels and may be driven by regulatory variants in LD with rs11552449, rather than by the missense variant itself. We acknowledge that conventional variant annotation tools (e.g., SIFT, PolyPhen) predict rs11552449 as benign or neutral. However, these tools rely on sequence conservation and simple substitution matrices, and may not capture context‑dependent conformational or dynamic effects. Therefore, we performed higher‑resolution AI‑based structural modelling, molecular dynamics simulations, and thermodynamic stability predictions to investigate potential cryptic functional consequences that are not evident from sequence‑based annotations alone.

**FIGURE 6:**
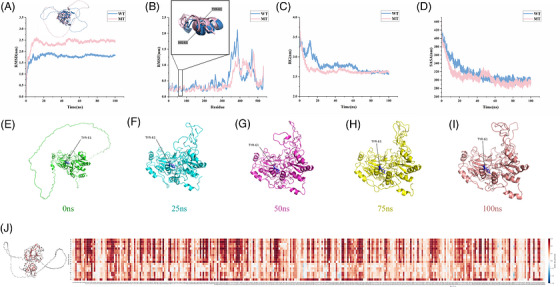
Comparative structural modeling analysis of the wild‐type and mutant forms of the missense variant DCLRE1B rs11552449. (A) Dynamic analysis of root mean square deviation. (B) Root mean square fluctuation profile. (C) Dynamic analysis of the radius of gyration. (D) Dynamic analysis of solvent accessible surface area. (E–I) Molecular dynamics snapshots of the DCLRE1B rs11552449 mutant protein from 0 to 100 ns. (G) Heatmap of predicted free energy change (ΔΔG°) for the DCLRE1B rs11552449 mutation based on the AI‐based ThermoMPNN model. MT, mutant; RMSD, root mean square deviation; RMSF, root mean square fluctuation; RG, radius of gyration; SASA, solvent accessible surface area; Tyr, tyrosin; WT, wild‐type.

The pLDDT scores from AlphaFold3 modelling are shown in Figure 5C (blue colour indicates high confidence). pLDDT is a residue‑wise metric that reflects how often the model predicts each residue to be in the indicated position and conformation; it is not a global measure of overall model quality. Most residues have pLDDT > 90, and residues with scores below 70 are located at the protein's edges, far from the missense mutation site. Critically, His.61 (the site of the rs11552449 mutation) lies in a region with pLDDT > 90, indicating that this residue belongs to a highly ordered structural domain (see also Figure 6E, where His.61 is marked). Therefore, the structural predictions around the mutation site are reliable, despite the overall model having inherent limitations.

### 3.11 Protein simulation result analysis

Molecular dynamics simulations of the wild‐type (WT) and mutant (MT) forms of the DCLRE1B protein were performed, and four key structural metrics were analysed: root mean square deviation (RMSD) (Figure 6A), root mean square fluctuation (RMSF) (Figure 6B), radius of gyration (Rg) (Figure 6C) and solvent accessible surface area (SASA) (Figure 6D). RMSD analysis shows that, compared to the WT, the RMSD of the MT increases continuously over time (0–100 ns), rising from 1.0 nm to 2.7 nm, whereas the WT remains stable within the range of 1.5–2.0 nm. This indicates that the MT fails to maintain a stable conformation and is prone to irreversible structural collapse. In the early phase of instability (> 10 ns), the MT RMSD rises sharply, suggesting that its conformation may shift into a non‐physiological state, such as partial unfolding or misaggregation.

RMSF analysis reveals that the mutation significantly alters the conformational flexibility of specific regions when comparing WT (blue) and MT (pink). In particular, the RMSF values of the MT increase in key functional regions (e.g., active sites, binding interfaces), indicating reduced structural stability that may compromise protein function. Moreover, the MT exhibits broader RMSF fluctuations at multiple residue positions, with more prominent peaks and troughs, implying that the mutation may disrupt local hydrogen bond networks or hydrophobic interactions.

Rg analysis shows that the Rg value of the MT decreases rapidly within the first 10 ns, while the WT remains relatively stable between 3.0 and 3.5 nm. This suggests that the MT adopts a looser overall structure in the early stages. Such structural loosening may impair the spatial arrangement of functional domains and increase the risk of misfolding, which could in turn affect protein function and cellular homeostasis.

SASA analysis indicates that the MT exhibits a significantly sharper decline in SASA during the 0–10 ns period compared to the WT, along with greater fluctuations throughout the simulation. This reflects increased exposure of the hydrophobic core on the protein surface. Potential risks include abnormal protein–protein interactions, such as erroneous binding with molecular chaperones, or promotion of pathological aggregation.

### 3.12 Phase‐specific analysis of protein simulation trajectories

Analysis of the mutant protein indicates a loss of structural stability, as evidenced by increased RMSD and pronounced fluctuations in Rg and SASA, suggesting uncontrolled conformational flexibility and an inability to maintain the structural rigidity necessary for proper function. To further investigate the dynamic behaviour, representative structures were extracted at five time intervals from 0 to 100 ns (Figure 6E–I). Structural comparisons between WT and MT reveal that the mutation at nucleotide position 181 (C to T), resulting in a histidine (His) to tyrosine (Tyr) substitution at residue 61, leads to the loss of key hydrogen and ionic bonds, as shown in the insets of Figure 6A,B. This disrupts local interactions, increases regional flexibility and results in a more relaxed overall conformation. Consequently, the mutant protein exhibits reduced structural stability and is more likely to lose its native functional properties.

### 3.13 Protein ΔΔG° Prediction

The missense mutation at position 61, where His is substituted by Tyr or Asp, is predicted to significantly affect protein stability. As shown on the left side of Figure 6J, the region around residue 61 is marked in red, indicating a mutation‐sensitive site where alterations are more likely to impact protein function, potentially compromising its role in DNA transcription and replication. ThermoMPNN predicted that the H61Y substitution (rs11552449) increases free energy by +2.1 kcal/mol, indicating reduced structural stability. The heatmap also includes the non‑human H61D substitution as a computational control; both H61Y and H61D were predicted to be destabilizing, suggesting that the loss of histidine at position 61, rather than the specific amino acid change, may be critical for the observed structural effect. No experimental ΔΔG values for DCLRE1B are currently available for comparison.

### 3.14 In silico knockout of *DCLRE1B* induces pro‑inflammatory and endothelial dysfunction signatures

To provide functional evidence, we performed an in silico knockout of *DCLRE1B* in human endothelial cells using scTenifoldKnk. Virtual knockout led to significant upregulation of pro‐inflammatory genes, including *IFI27* (FC = 66.5, adj. *p* = 8.6 × 10^−14^), *CD74* (FC = 52.3, adj. *p* = 8.1 × 10^−11^), *IFITM1* (FC = 40.5, adj. *p* = 2.4 × 10^−8^), and multiple HLA class II genes, while downregulating endothelial integrity genes such as *CLDN5*, *VWF* and *COL3A1* (Table S24, Figure S1). GSEA revealed enrichment of immune‐related pathways (e.g., antigen processing and presentation, NES = 2.23, *p* = 1.80 × 10^−6^) and suppression of innate immune sensing pathways (e.g., NOD‐like receptor signalling, NES = –1.80, *P* = 0.0033) (Table S25, Figure S1). These results suggest that *DCLRE1B* loss promotes a pro‐inflammatory endothelial state and compromises vascular integrity, supporting its potential role in atherosclerosis. However, in silico knockout and structural predictions are computational in nature and do not establish causality.

## 4 DISCUSSION

This study presents a comprehensive investigation into the heritability and pathophysiology of ASCVD, including CHD, stroke, TIA, PAD and AAA. By employing an integrative approach that incorporates GWAS, Genomic SEM (mvASCVD), fine mapping, transcriptional profiling, PRS and AI models, we have identified novel genetic loci that predispose individuals to these conditions. Importantly, our findings demonstrate that genetic variants not only predispose to mvASCVD but also exert their effects through gene expression, protein structure, cellular machinery and other downstream mechanisms, thereby profoundly influencing disease development, pathology and clinical outcomes in affected individuals. Our study provides a theoretical framework for understanding how genetic loci shape the development of mvASCVD, offering critical insights for the development of precision medicine strategies and adaptive therapeutic interventions. Furthermore, our work highlights the potential for leveraging these genetic insights to inform preventive strategies, thereby improving outcomes in populations at risk of cardiovascular events.

### 4.1 Genetic structure and shared genetic factors

In recent genetic studies, a growing body of attention has been directed towards the genetic relationships among various cardiovascular disease phenotypes. Analysis using Genomic SEM (mvASCVD) revealed significant genetic covariances among these phenotypes, indicating that they share common genetic factors. This finding supports the hypothesis proposed by Rheenen et al. (2019) that genetic correlations among certain disease phenotypes may reflect shared genetic factors, with these factors potentially exerting profound effects on the aetiology, pathogenesis and clinical outcomes of these diseases.^42^ In our study, these cardiovascular phenotypes, particularly CHD, stroke, and PAD, demonstrated a particularly striking pattern, suggesting that these conditions are not isolated but are interconnected through shared genetic influences. By synthesizing GWAS data across diverse populations and environmental contexts, we identified genetic effects that transcend traditional boundaries, further solidifying the role of genetic factors in the mechanistic underpins of multiple cardiovascular diseases. Recent work by Dichgans et al. (2014)^43^ and Haritala et al. (2021)^44^ has further corroborated the presence of shared genetic factors among various cardiovascular disease phenotypes. For instance, Dichgans et al. (2014)^43^ demonstrated that multiple common cardiovascular disease risk loci overlap significantly between CHD and stroke. These findings are consistent with our study, underscoring the importance of genetic models in elucidating the complex architecture of cardiovascular disease.

We acknowledge that the five ASCVD‑related phenotypes have distinct pathophysiological features. However, the KMO measure of sampling adequacy (.69) and the good fit of the one‑factor common factor model (CFI = .97, SRMR = .067) support the existence of substantial shared genetic variance across these conditions. Using the genetic covariance matrix and the maximum number of factors formula, we identified a possible two‑factor structure (CHD + PAD vs. stroke + TIA), but the high factor correlation (r ≈ 0.80) indicated that most genetic variance is shared. The two‑factor sensitivity model provided only marginal improvement in fit while excluding AAA, a clinically important ASCVD entity. Therefore, we interpret mvASCVD as a statistical summary of the shared genetic liability across common atherosclerotic and cerebrovascular diseases, rather than a claim that all five conditions are biologically identical.

### 4.2 The relationship between novel SNPs and complex traits

We acknowledge that the term ‘novelty’ must be defined relative to a specific reference. In this study, 90 variants were not significant in any of the five input GWAS, demonstrating the enhanced statistical power of genomic SEM. However, comparison with the broader literature revealed that only 19 of these 90 variants had never been associated with any cardiovascular or metabolic trait. The majority (56 variants) had been previously reported in ASCVD endpoint GWAS.

Through Genomic SEM (mvASCVD) subsequent analysis, we successfully identified multiple SNPs that exhibited significant associations with lipid profiles,^45^ obesity,^46^ cognitive function^47^ and bone mineral density.^48^ These identified SNPs primarily reside in the gene regulatory regions, particularly non‐coding regions, suggesting that non‐coding regions may play a key role in the genetic mechanisms underlying complex traits. Non‐coding variants are widely recognized to influence complex traits as they are believed to regulate gene expression through mechanisms such as transcription factor binding, RNA splicing, and stability regulation.^49^, ^50^ In our study, many of the identified SNPs were associated with cardiovascular risk factors such as high lipid levels, which not only provide potential genetic targets for future research but also offer new insights into the genetic connections among mvASCVD. Specifically, these genetic markers associated with obesity and lipid metabolism, typical cardiovascular risk factors, may help identify individuals at high risk and provide evidence for early intervention strategies. Recent studies by Zhang et al. (2021) and Yang et al. (2025)^51^, ^52^ have further highlighted the role of non‐coding regions in cardiovascular diseases, demonstrating specific SNPs’ genetic associations with lipid and obesity levels. As our previously published researches^2^, ^53^, ^54^, ^55^ indicate, in the mechanism exploration stage, the non‐coding region, as an instrumental variable, has significant value in detecting the causal relationship between exposures and outcomes. These findings enhance our understanding of the genetic underpinnings of complex traits.

### 4.3 Fine mapping revealed genetic loci

Through genome‐wide fine mapping, we have identified multiple key SNPs that exhibit significant associations with genes linked to lipid metabolism abnormalities, obesity and hypertension.^56^, ^57^ These findings align with the results of Ding et al. (2024),^58^ who demonstrated that key genomic loci associated with cardiovascular diseases and lipid metabolism are typically highly correlated with specific SNPs. The identification of these SNPs enables a more precise understanding of how these genes influence lipid metabolism, atherosclerotic plaque formation and inflammatory responses, ultimately affecting individual health and disease susceptibility. Notably, in multiple genomic regions associated with lipid metabolism and inflammatory responses, we have discovered genetic markers that suggest these regions may play a pivotal role in the formation of mvASCVD phenotypes.

Transcriptomic analysis has further elucidated the disease‐related genes and their associated biological pathways. Through FUSION transcriptomic analysis, we have identified potential disease‐associated genes linked to the SNPs under investigation. These genes predominantly involve key biological pathways such as lipid metabolism and immune responses, and are closely associated with known disease pathways, including those related to cardiovascular metabolism, cancer and others.^59^ For instance, variations in genes involved in lipid metabolism may influence processes such as cholesterol and fatty acid oxidation, thereby increasing susceptibility to chronic metabolic disorders (e.g., diabetes, atherosclerosis). Similarly, genes associated with immune response pathways, such as those involved in immune tolerance and inflammation regulation, have been confirmed to play a role in this study. These pathways may contribute significantly to the genetic basis of mvASCVD, offering new insights into how the immune system regulates cardiovascular metabolism and disease progression. These findings underscore the importance of studying genetic pathways in understanding the genetic architecture of complex cardiovascular diseases.

### 4.4 Genomic elements and risk chromosome identification

This study reveals multiple disease‐related risk chromosomal regions, with many located in non‐coding regions. Studies demonstrate that these regions regulate the expression of nearby genes, thereby influencing various biological processes, including cell proliferation, differentiation and immune responses.^49^, ^50^ For instance, we identified multiple high‐risk loci spanning chromosome 1, 2, 3 and 6, which are associated with lipid metabolism and cardiovascular diseases. These loci are often enriched in functional genomic regulatory regions, such as enhancers and promoter regions. This finding aligns with Khalifa's (2024) hypothesis^60^ that non‐coding genomic regions play a critical role in the inheritance of genetic traits and diseases. Through these regulatory regions, the modulation of gene expression—either activation or repression—can directly influence specific biological processes, thereby determining individual vulnerability to diseases. During the identification of risk chromosomal regions, we uncovered several key genomic regulatory elements, including promoter regions, enhancer regions and long non‐coding RNAs (lncRNAs), which may play pivotal roles in the genetic architecture of complex diseases, such as CNS disorders and cancer.^61^, ^62^, ^63^, ^64^ These genomic elements are known to regulate the spatio‐temporal expression patterns of genes, exerting long‐term impacts on specific biological processes. Notably, in certain risk chromosomal regions, variations in lncRNA expression have been observed to correlate with an individual's susceptibility to disease, suggesting that these regions may also influence disease progression through RNA‐level regulation.

### 4.5 Protein function alterations caused by missense mutations

The study reveals that the *DCLRE1B* rs11552449 may induce instability and loss of protein functionality through the following mechanisms: structural relaxation. The mutation leads to a more relaxed conformation of the DCLRE1B protein, losing its structural rigidity. Structural relaxation is closely associated with the loss of protein functionality, as protein function often relies on its stable three‐dimensional structure. Our protein dynamics simulations indicate that the mutant protein adopts a more relaxed overall structure early on, suggesting that the mutant structure tends toward a more relaxed state. Such changes may disrupt the functional domains of the protein, impairing its normal activity. Structural relaxation may influence protein functionality via the following mechanisms: first, the exposure of hydrophobic cores may increase the risk of misfolding and aggregation of proteins, leading to erroneous protein interactions within the cell. Second, the loss of structural stability may impair the protein's ability to recognize its binding partners or perform its normal biological functions.

*Exposure of hydrophobic residues*: The mutation induced by rs11552449 increases the exposure of hydrophobic residues on the protein surface. These residues were typically hidden within the protein's interior in the wild‐type structure. The exposure of hydrophobic residues may trigger abnormal protein interactions, particularly with other proteins or partner proteins, leading to misfolded protein aggregates and pathological protein aggregation. Such erroneous protein interactions may trigger pathological protein aggregation, activate the cell's stress response, ultimately leading to apoptosis. Additionally, the exposure of hydrophobic residues may promote the formation of aberrant proteosomes, leading to an increase in the protein aggregate volume. This may activate the cell's ubiquitin‐proteasome system and the unfolded protein response (UPR). These misfolded proteins may disrupt cellular functions and contribute to disease pathogenesis, which is closely linked to several inflammatory and immune‐related diseases.

*Loss of functional domain stability*: The mutation significantly increases the flexibility of functional regions and binding interfaces. This may lead to the loss of stability in these critical functional regions. Our RMSF analysis demonstrates that the mutant protein shows increased flexibility in multiple functional regions, suggesting a decline in the structural stability of these regions. This phenomenon may result in the loss of protein functionality, especially in regions critical for binding partners or other protein interactions. For example, DCLRE1B is a key DNA repair protein involved in DNA chain intercalation repair and plays a critical role in processes such as cell metabolism, DNA damage repair and immune system development.^65^ The structural changes induced by the mutation may impair these repair functions, impairing the protein's ability to perform its role in DNA repair. This, in turn, may negatively impact the stability of genes and cellular metabolism. Furthermore, DCLRE1B has a potential role in cardiovascular diseases, such as atherosclerosis. Although *DCLRE1B* rs11552449 has been implicated in breast cancer risk,^66^ its role in cardiovascular diseases, particularly atherosclerosis, remains underexplored. As a DNA repair protein, mutations in *DCLRE1B* may impair DNA damage repair in the endothelium, leading to the accumulation of DNA damage in vascular tissues. Over time, such DNA damage may exacerbate atherosclerotic lesion formation and disease progression. The long‐term accumulation of DNA damage may further impair cellular metabolism and contribute to the pathological progression of cardiovascular diseases. Our findings suggest that *DCLRE1B* mutations not only impair its function in DNA repair but may also promote protein misfolding and aggregation, increasing the risk of cellular functional dysregulation. This may further exacerbate the pathological progression of atherosclerotic disease. These discoveries provide new insights into the molecular mechanisms underlying these complex diseases and identify potential therapeutic targets for related diseases.

A potential discrepancy should be noted: our TWAS indicated that higher predicted expression of *DCLRE1B* is associated with increased ASCVD risk, whereas the in silico knockout suggested that complete loss of *DCLRE1B* promotes a pro‑inflammatory endothelial state. These observations are not necessarily contradictory. The missense variant H61Y is predicted to destabilize the DCLRE1B protein, likely impairing its function. The TWAS signal, however, reflects total DCLRE1B transcript levels and may be driven by a distinct regulatory variant in LD with rs11552449, or may represent a compensatory transcriptional response to protein dysfunction. Moreover, complete gene knockout differs from a missense mutation that partially impairs protein function. Thus, our combined findings suggest that *DCLRE1B* plays a role in ASCVD, but the precise mechanism—whether through loss of DNA repair function, altered inflammatory signalling, or both—requires further experimental investigation, as these computational findings should be considered hypothesis‑generating.

### 4.6 Advantages

This work offers fresh perspectives on the genetic underpinnings of ASCVD. By merging genome‑wide structural equation modelling, fine‑mapping, and transcriptomic profiling, we uncovered multiple previously unrecognized genetic loci and demonstrated how they influence complex traits through modulation of gene expression. These findings deepen our mechanistic grasp of ASCVD and open new avenues for precision medicine and public health strategies. Furthermore, this investigation represents the first effort to integrate AlphaFold3, molecular dynamics simulations, and the ThermoMPNN thermodynamic stability predictor to systematically explore how mutations in *DCLRE1B* alter protein stability and function. The structural model of DCLRE1B predicted by AlphaFold3, coupled with dynamic simulations, reveals that mutations lead to protein instability and increased flexibility in critical functional regions, thereby affecting function. Thermodynamic analysis shows that the mutations significantly increase the free energy of DCLRE1B, reducing its stability, suggesting that these mutations may result in functional loss. This study uncovers mechanisms such as structural loosening, exposure of the hydrophobic core and destabilization of functional regions induced by mutations, offering new molecular insights into DCLRE1B‐associated inflammatory diseases. The multi‐faceted research framework not only advances the understanding of protein structure and function but also provides theoretical support for the treatment of ASCVD and related diseases.

### 4.7 Limitations

Despite the new genetic insights into ASCVD presented here, several caveats should be acknowledged. One major limitation is that nearly all participants were of European ancestry, leaving the applicability of our findings to other ethnic groups untested; larger and more diverse cohorts will be needed to confirm generalizability. Second, while fine‑mapping and transcriptomic analyses pinpointed numerous loci associated with mvASCVD, the exact biological pathways through which these genes operate remain largely unknown—a challenge that future work must address by examining how the variants influence gene expression, inflammatory responses, and metabolic processes at the individual level. The cell‑type enrichment analysis was performed using the Tabula Muris mouse atlas; future studies using human single‑cell datasets (e.g., Human Cell Atlas) would further strengthen these observations. Moreover, although our study emphasizes the importance of genetic determinants, environmental factors (such as diet, physical activity and living conditions) should not be ignored. Their interplay with genetic risk variants in shaping ASCVD phenotypes warrants deeper investigation into gene–environment interactions. Additionally, while AlphaFold3 provides high‑confidence static structures, it does not capture the full conformational ensemble, and mutant structures may be less accurate in regions involved in protein‑protein interactions. Our use of 100 ns molecular dynamics simulations partially mitigates this limitation, but experimental structural validation (e.g., X‑ray crystallography or cryo‑EM) would be required to confirm the dynamic behaviour of the DCLRE1B mutant. Moreover, while the ThermoMPNN model demonstrates high accuracy in thermodynamic stability predictions, not experimentally validated neutral or stabilizing mutations in DCLRE1B were available as internal controls for ThermoMPNN predictions. Therefore, we cannot completely exclude the possibility that the predicted destabilization reflects general model bias rather than mutation‑specific effects. Future experimental studies (e.g., circular dichroism, thermal shift assays) are needed to validate these predictions. Additionally, no experimental ΔΔG measurements for *DCLRE1B* mutations exist to validate our ThermoMPNN predictions. Future biophysical studies (e.g., differential scanning fluorimetry) are needed to confirm the predicted destabilization and to assess the relevance of the H61D control. Our molecular dynamics simulations were limited to 100 ns, which is relatively short for observing large‑scale conformational changes. The mutant RMSD continued to rise throughout the simulation without reaching a plateau, suggesting that the system had not fully equilibrated. To test reproducibility, we performed two independent replicates with different random seeds; both showed similar RMSD trajectories, indicating that the trend is not a stochastic artefact. Nevertheless, longer simulations (≥500 ns or 1 µs) and alternative force fields would be required to determine whether the mutant eventually stabilizes in a new conformation or continues to unfold. Therefore, our simulation results should be interpreted as indicative of early instability rather than definitive evidence of complete unfolding. Furthermore, although our in silico knockout analysis using scTenifoldKnk provides functional evidence linking *DCLRE1B* to endothelial inflammation and dysfunction, these computational predictions cannot replace definitive in vitro or in vivo experiments. Future studies using CRISPR‐Cas9 knockout in primary human endothelial cells, followed by functional assays (e.g., inflammatory cytokine secretion, endothelial permeability, leucocyte adhesion), are warranted to conclusively establish the role of *DCLRE1B* in ASCVD pathogenesis.

## 5 CONCLUSION

Our integration of Genomic‐SEM with AI‐driven molecular predictions has elucidated novel genetic variants that critically influence the pathogenesis of ASCVD, particularly through their effects on transcriptional regulation and protein stability. These findings not only enhance our mechanistic understanding of the disease but also provide a robust framework for the development of precision medicine strategies, including personalized prevention, early diagnostics and targeted therapeutic interventions. The implications of this work underscore the potential for leveraging advanced genetic and computational approaches to transform cardiovascular disease management and outcomes.

## AUTHOR CONTRIBUTIONS

*Study concept and design*: Liwan Fu. *Acquisition of data*: Liwan Fu and Xiaodi Han. *Analysis and interpretation of data*: Liwan Fu and Xiaodi Han. *Drafting of the manuscript*: Liwan Fu and Xiaodi Han. *Critical revision of the manuscript for important intellectual content*: Liwan Fu, Xiaodi Han, Qin Liu, Yuquan Wang and Yue‐Qing Hu. *Funding recipients*: Liwan Fu and Yue‐Qing Hu.

## CONFLICT OF INTEREST STATEMENT

The authors declare no conflicts of interest.

## ETHICS STATEMENT

The authors have nothing to report.

Publicly available code and software were used to perform the analyses. The software used in this study is publicly available and accessed without restriction. R package GenomicSEM v.0.0.5 is available at https://github.com/GenomicSEM/GenomicSEM. R FUSION Pipeline v.1.4.2 for TWAS analysis is available at http://gusevlab.org/projects/fusion/. Python package FOCUS v.0.6.10 for FOCUS fine mapping for FUSION is available at http://github.com/bogdanlab/focus/. R package coloc v.5.1.0.1 for colocalization is available at https://cran.r‐project.org/web/packages/coloc/index.html. Python package CELLECT v.1.3.0 for single‐cell enrichment analyses is available at https://github.com/perslab/CELLECT. Python package CELLEX v.1.2.1 for single‐cell processing is available at https://github.com/perslab/CELLEX. R package echolocatoR v.2.0.3 for fine mapping using SuSIE and FINEMAP is available at https://github.com/RajLabMSSM/echolocatoR. MendelVar is available at https://mendelvar.mrcieu.ac.uk/. FUMA and MAGMA v.1.4.0 are available at https://fuma.ctglab.nl/. R v.4.2.1 and Python v.3.8 were used to format data for analyses. For protein simulation, we used Ensembl, https://www.ensembl.org/; NCBI, https://www.ncbi.nlm.nih.gov/; GenCards, https://www.genecards.org/; UniProt, https://www.uniprot.org/; AlphaFold3, https://alphafoldserver.com/; ThermoMPNN, https://colab.research.google.com/.

## Supporting information



Supporting Information

Supporting Information

Supporting Information

Supporting Information

Supporting Information

Supporting Information

## Data Availability

All analyses were based upon publicly available data. Summary‐level statistics for coronary heart disease are available at https://ftp.ebi.ac.uk/pub/databases/gwas/summary_statistics/GCST90132001‐GCST90133000/GCST90132314; stroke, https://ftp.ebi.ac.uk/pub/databases/gwas/summary_statistics/GCST005001‐GCST006000/GCST005838; transient ischemic attack, https://www.finngen.fi/en/access_results; peripheral artery disease, https://ftp.ebi.ac.uk/pub/databases/gwas/summary_statistics/GCST90018001‐GCST90019000/GCST90018890; and abdominal aortic aneurysm, https://csg.sph.umich.edu/willer/public/AAAgen2023. GTEx weights for FUSION analyses are available at https://gusevlab.org/projects/fusion/. Single‐cell gene expression data from the Tabula Muris study are available at https://tabula‐muris.ds.czbiohub.org/. All other data supporting the findings of this study are available from the corresponding author upon reasonable request.
