# Supplementary material for: Precision medicine for atherosclerotic cardiovascular disease: Integrative genomics maps risk loci and AI‐predicted functional consequences
Source: Clin Transl Med. 2026 Jul 10;16(7):e70732. doi: 10.1002/ctm2.70732 (PMC13351343; doi:10.1002/ctm2.70732)
Supplement: Supplementary file 1 — Supporting Information [file CTM2-16-e70732-s002.pdf]

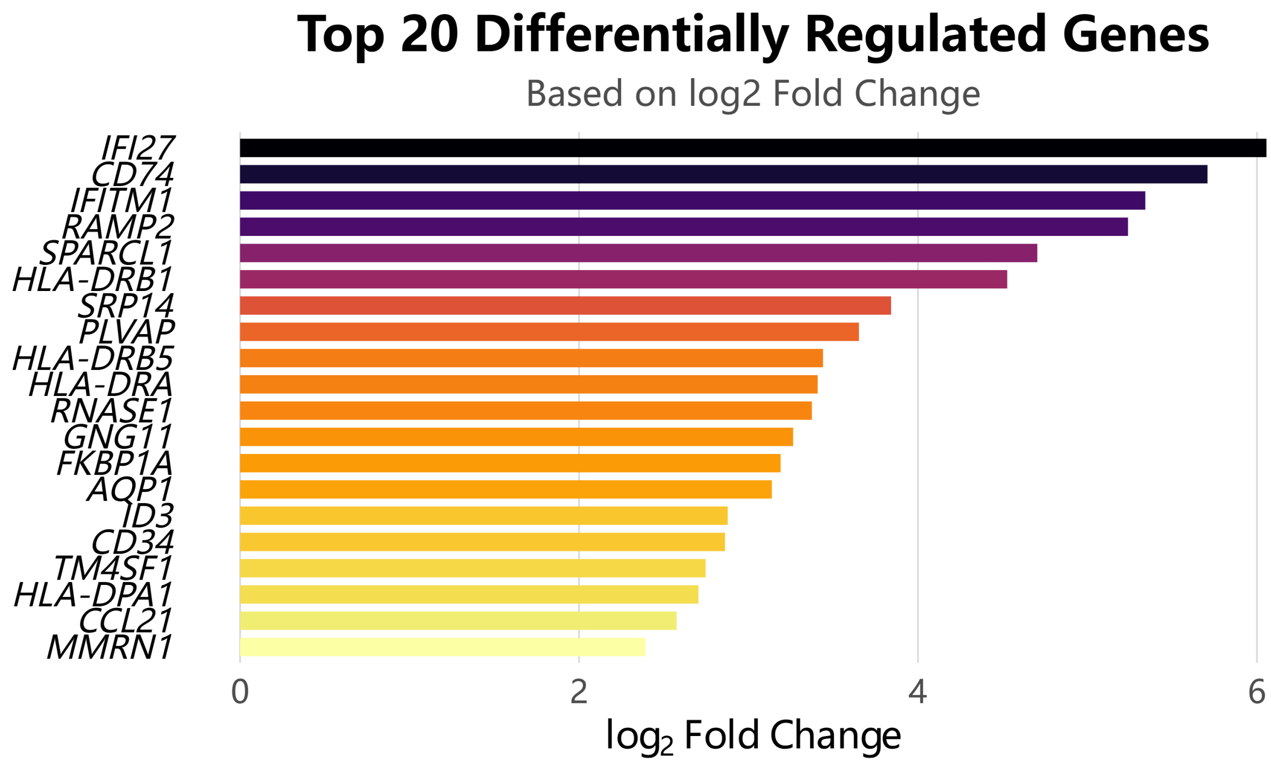

Top 20 Differentially Regulated Genes
Based on log2 Fold Change
IFI27
CD74
IFITM1
RAMP2
SPARCL1
HLA-DRB1
SRP14
PLVAP
HLA-DRB5
HLA-DRA
RNASE1
GNG11
FKBP1A
AQP1
ID3
CD34
TM4SF1
HLA-DPA1
CCL21
MMRN1
0
2
4
6
log2 Fold Change

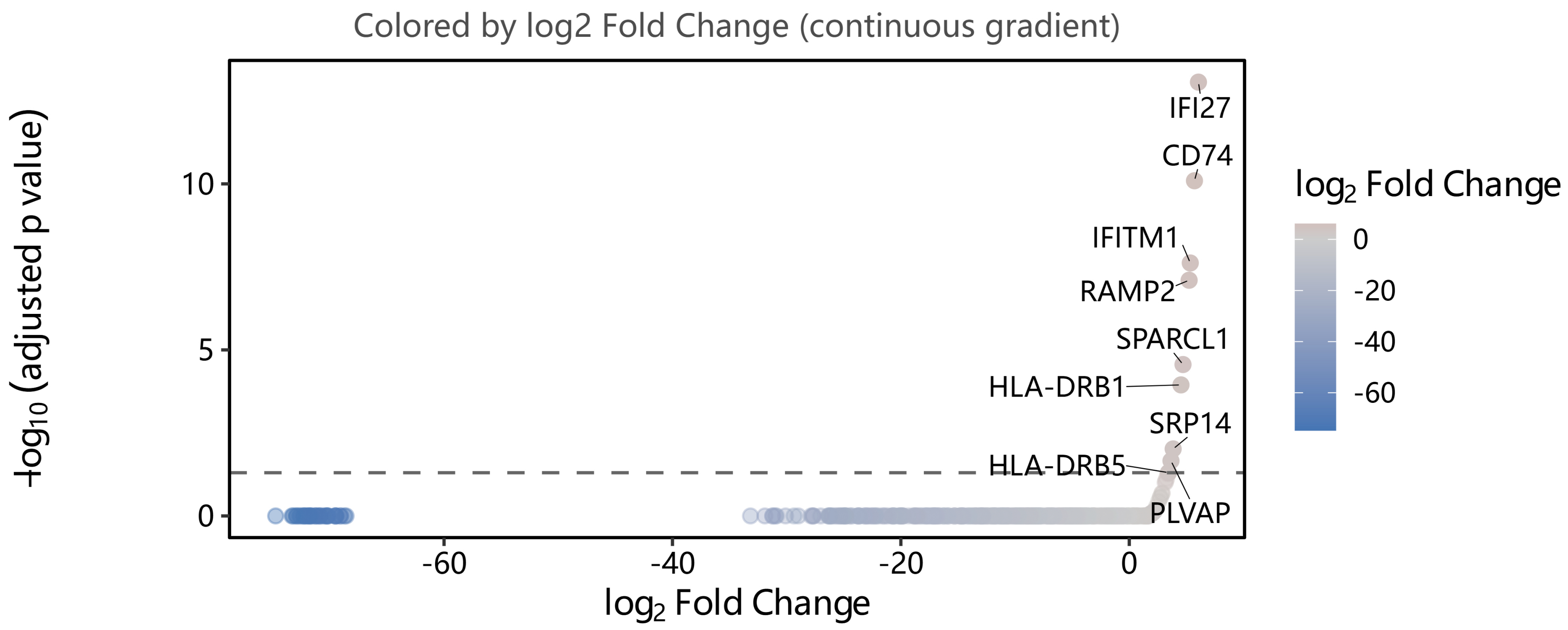

Colored by log2 Fold Change (continuous gradient)
-log10 (adjusted p value)
10
5
0
-60
-40
-20
0
log2 Fold Change
IFI27
CD74
IFITM1
RAMP2
SPARCL1
HLA-DRB1
SRP14
HLA-DRB5
PLVAP
log2 Fold Change
0
-20
-40
-60

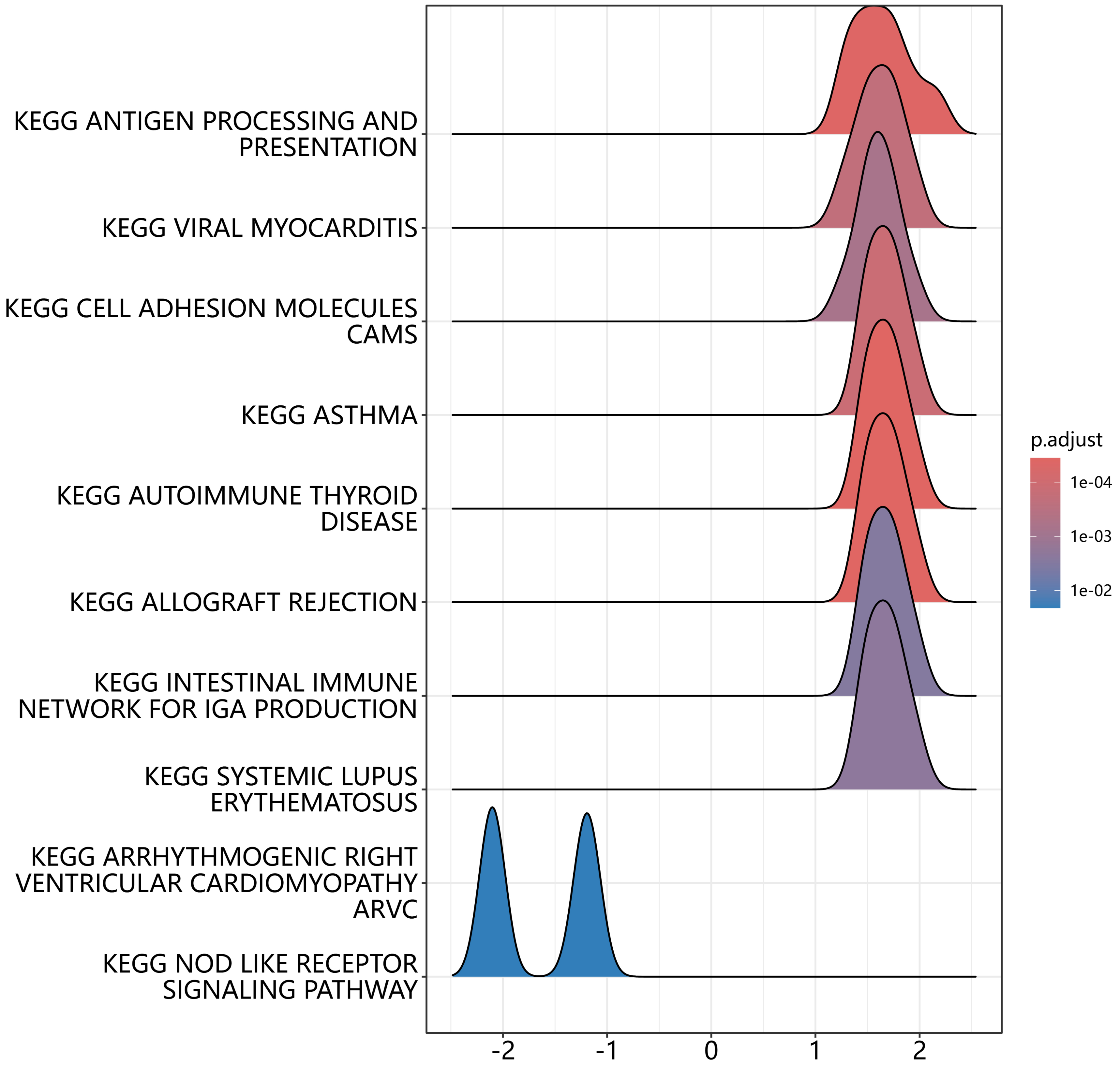

KEGG ANTIGEN PROCESSING AND PRESENTATION
KEGG VIRAL MYOCARDITIS
KEGG CELL ADHESION MOLECULES CAMS
KEGG ASTHMA
KEGG AUTOIMMUNE THYROID DISEASE
KEGG ALLOGRAFT REJECTION
KEGG INTESTINAL IMMUNE NETWORK FOR IGA PRODUCTION
KEGG SYSTEMIC LUPUS ERYTHEMATOSUS
KEGG ARRHYTHMOGENIC RIGHT VENTRICULAR CARDIOMYOPATHY ARVC
KEGG NOD LIKE RECEPTOR SIGNALING PATHWAY
-2
-1
0
1
2
p.adjust
1e-04
1e-03
1e-02
