# Supplementary figures and images for: Precision medicine for atherosclerotic cardiovascular disease: Integrative genomics maps risk loci and AI‐predicted functional consequences (part 1 of 3)
Source: Clin Transl Med. 2026 Jul 10;16(7):e70732. doi: 10.1002/ctm2.70732 (PMC13351343; doi:10.1002/ctm2.70732)

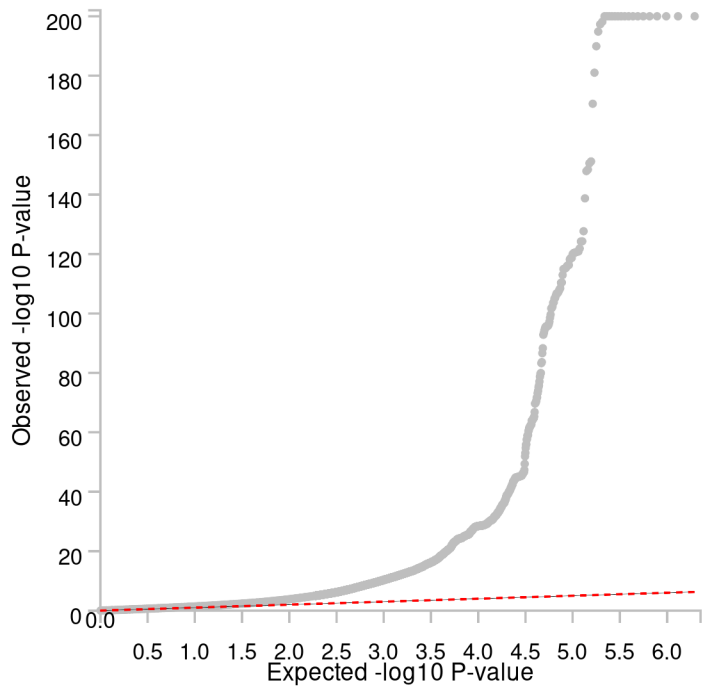

Supplement: Supplementary file 2 — Supporting Information [file CTM2-16-e70732-s006.pdf]

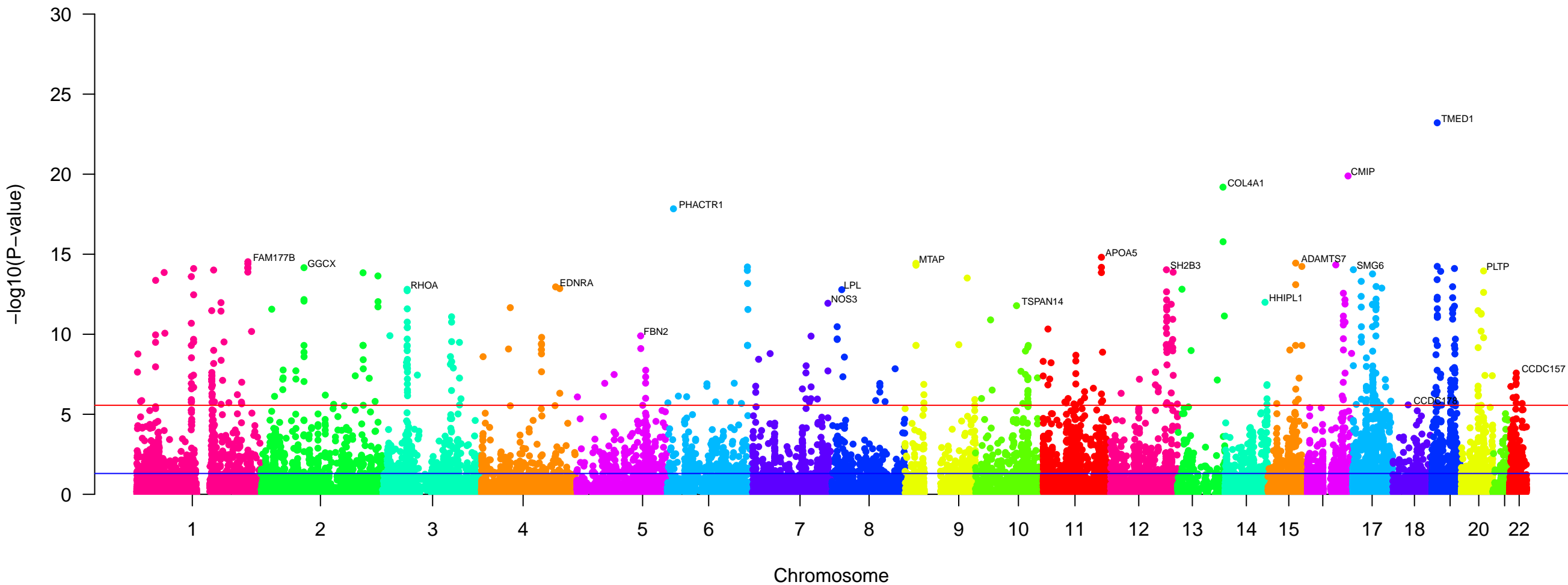

Supplement: Supplementary file 3 — Supporting Information [file CTM2-16-e70732-s004.pdf]

# LocusZoom plots of GWAS top lead SNP

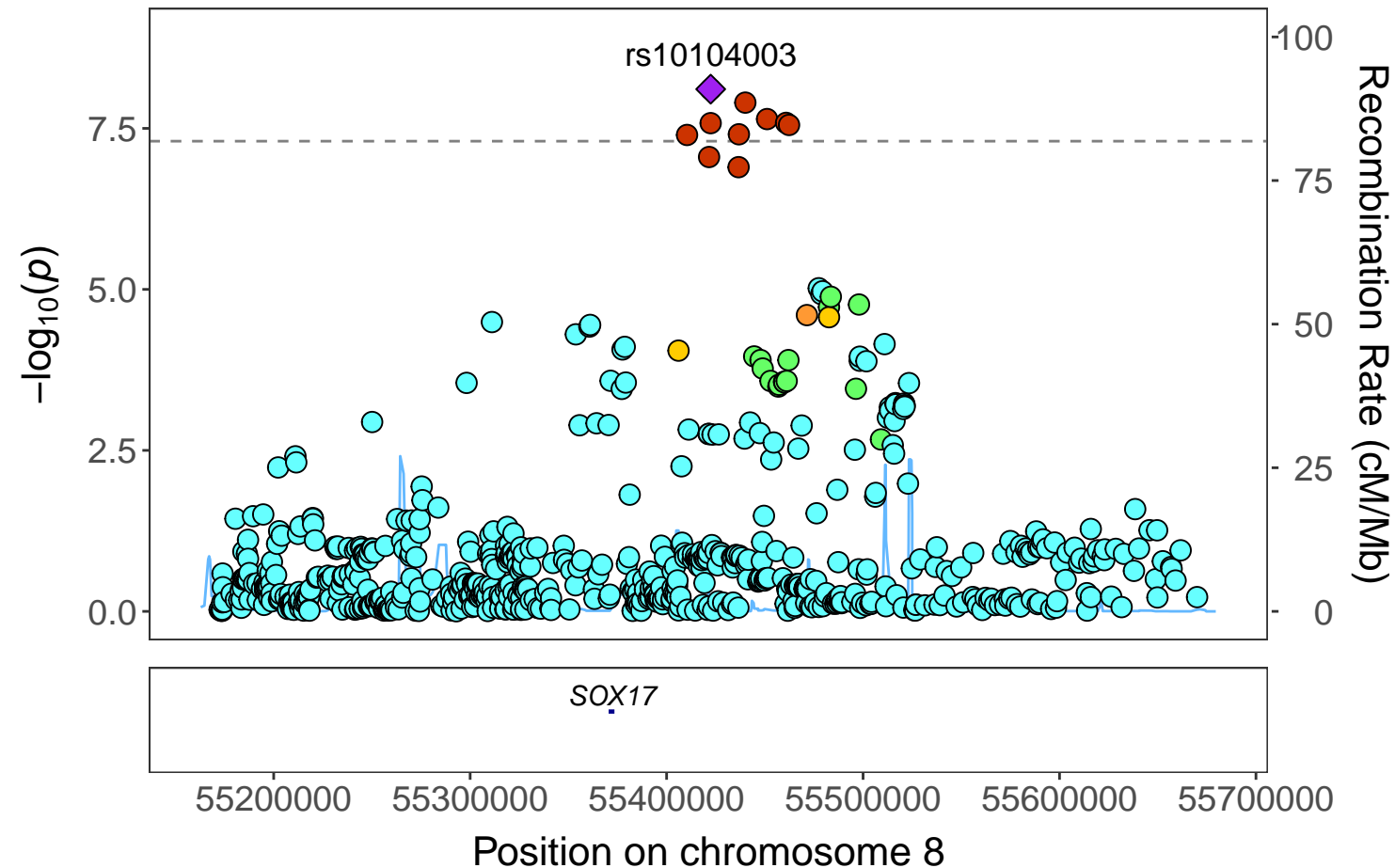

$r^2$    miss   0.0–0.2   0.2–0.4   0.4–0.6   0.6–0.8   0.8–1.0

Supplement: Supplementary file 5 — Supporting Information [file CTM2-16-e70732-s001.zip › LocusZoom/Sfig_rs10104003_locusZoom.pdf]

# LocusZoom plots of GWAS top lead SNP

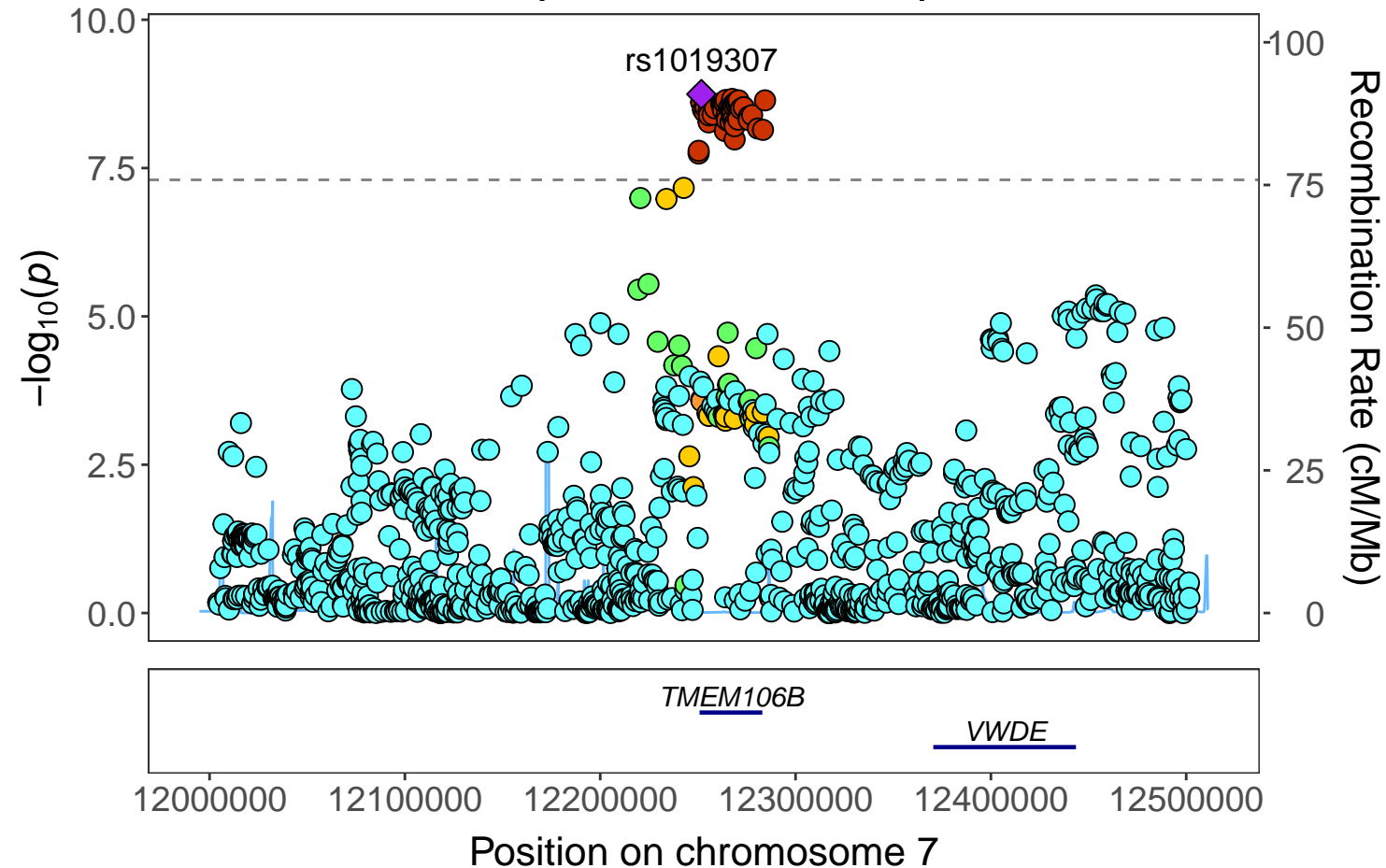

$r^2$    miss   0.0–0.2   0.2–0.4   0.4–0.6   0.6–0.8   0.8–1.0

Supplement: Supplementary file 5 — Supporting Information [file CTM2-16-e70732-s001.zip › LocusZoom/Sfig_rs1019307_locusZoom.pdf]

# LocusZoom plots of GWAS top lead SNP

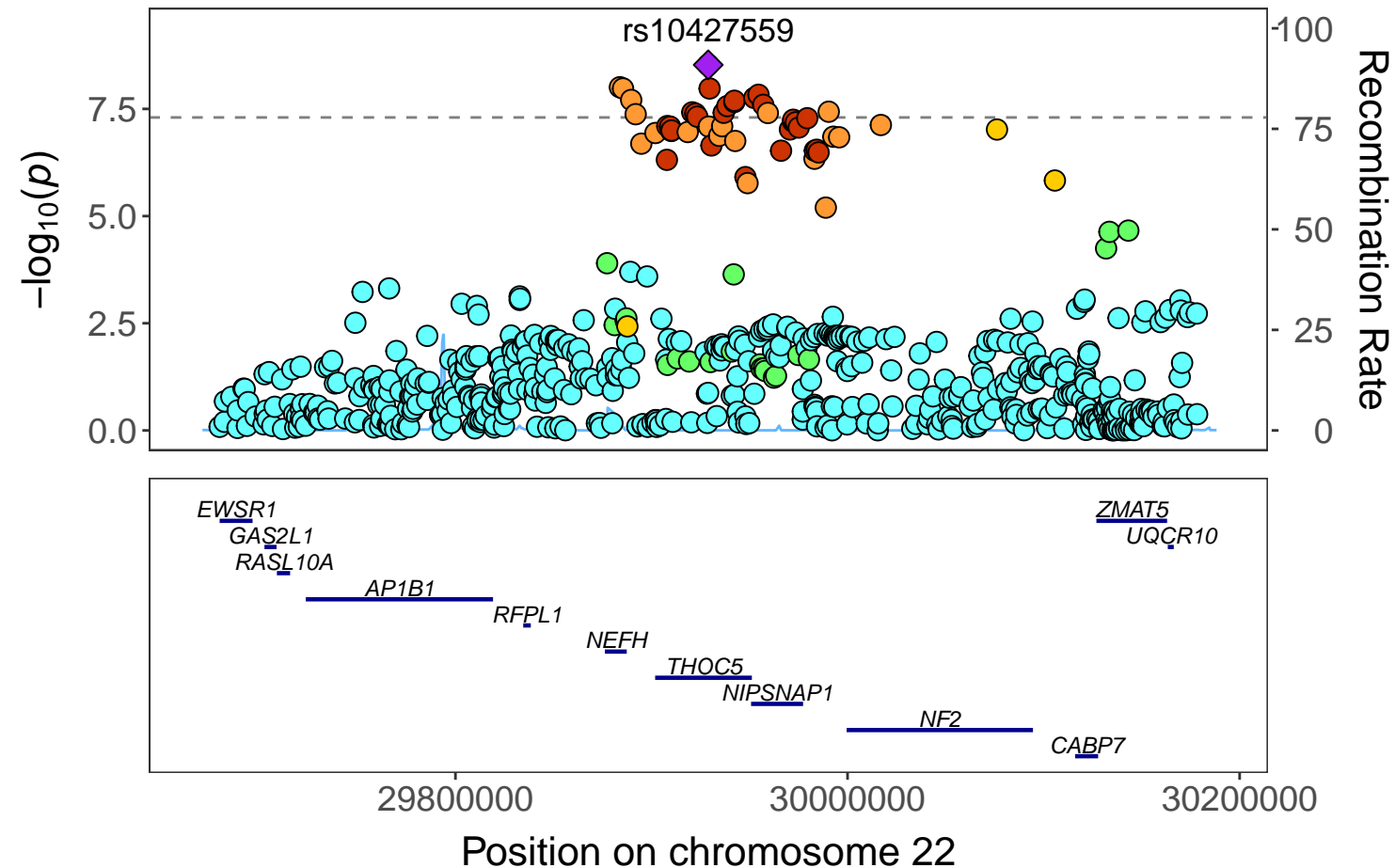

Supplement: Supplementary file 5 — Supporting Information [file CTM2-16-e70732-s001.zip › LocusZoom/Sfig_rs10427559_locusZoom.pdf]

# LocusZoom plots of GWAS top lead SNP

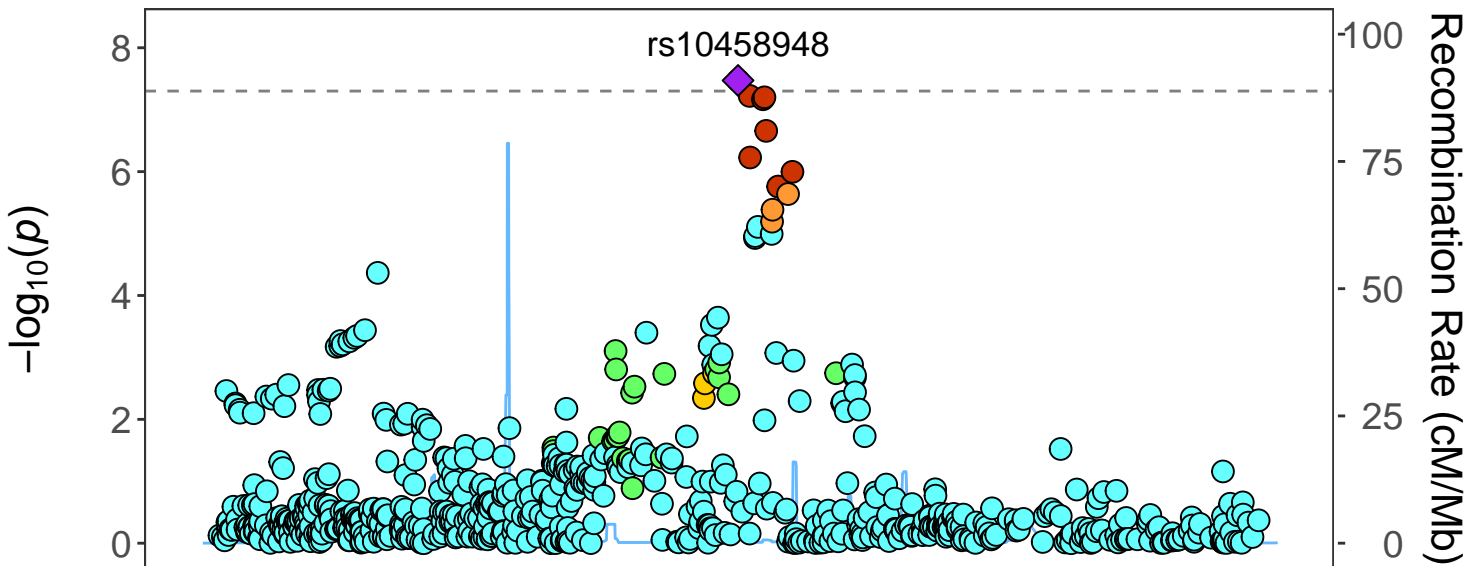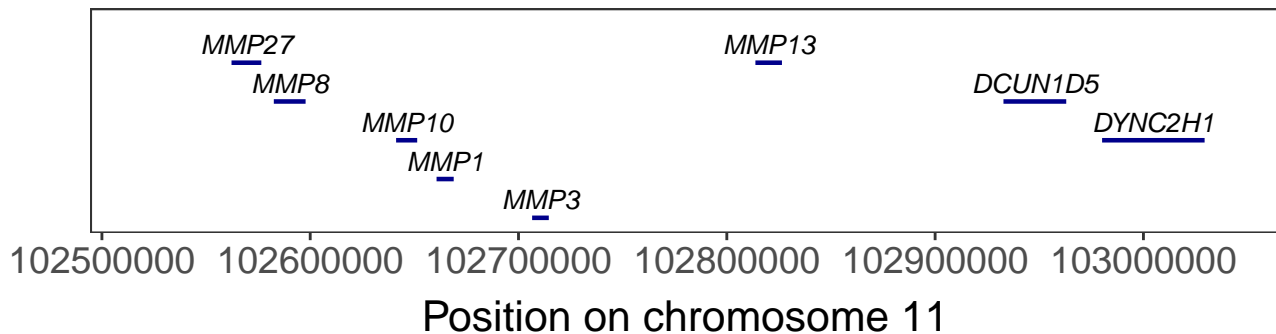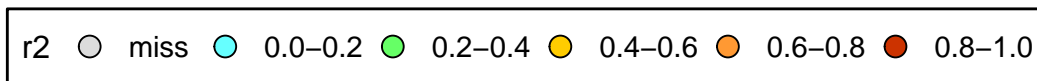

Supplement: Supplementary file 5 — Supporting Information [file CTM2-16-e70732-s001.zip › LocusZoom/Sfig_rs10458948_locusZoom.pdf]

# LocusZoom plots of GWAS top lead SNP

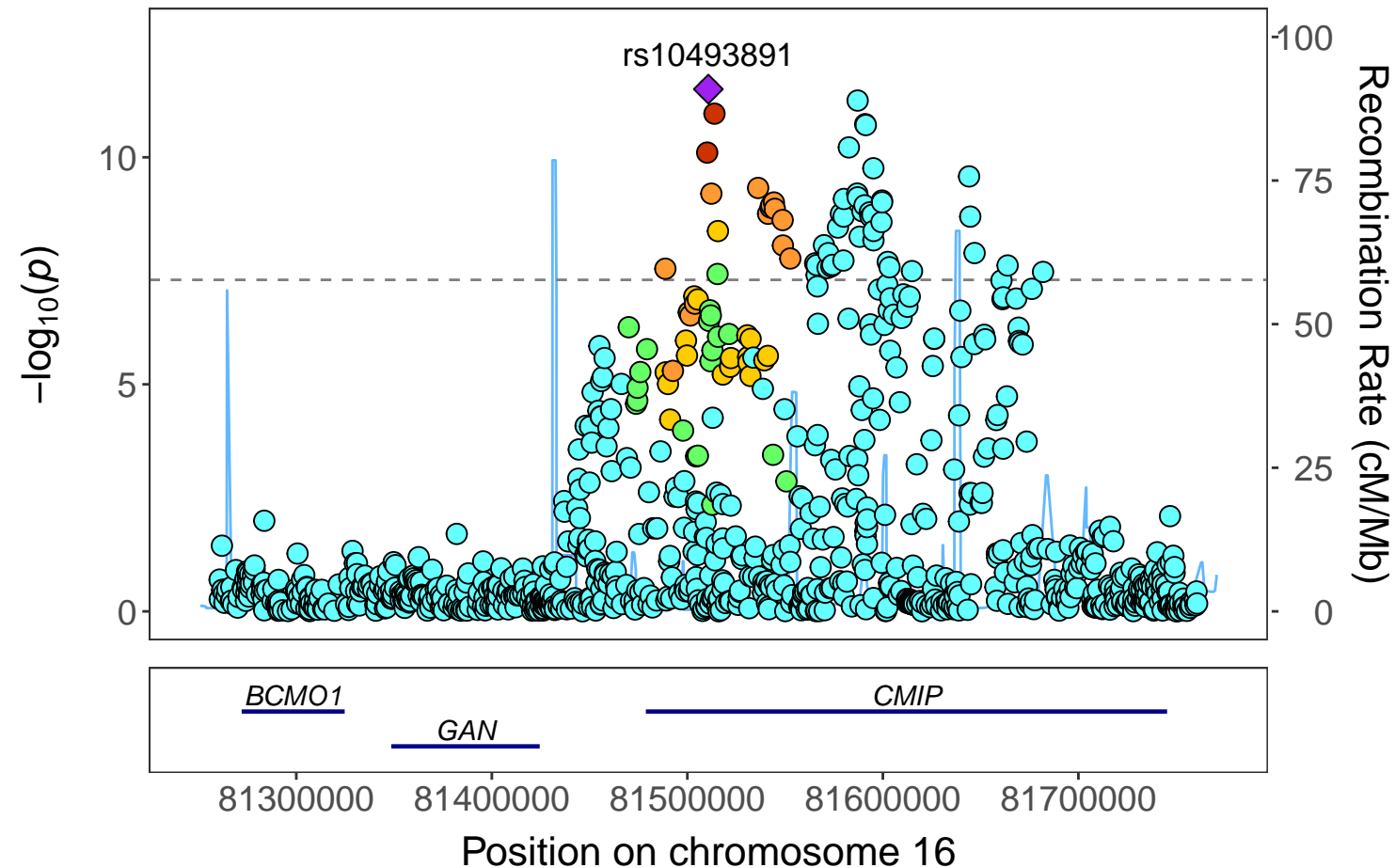

Supplement: Supplementary file 5 — Supporting Information [file CTM2-16-e70732-s001.zip › LocusZoom/Sfig_rs10493891_locusZoom.pdf]

# LocusZoom plots of GWAS top lead SNP

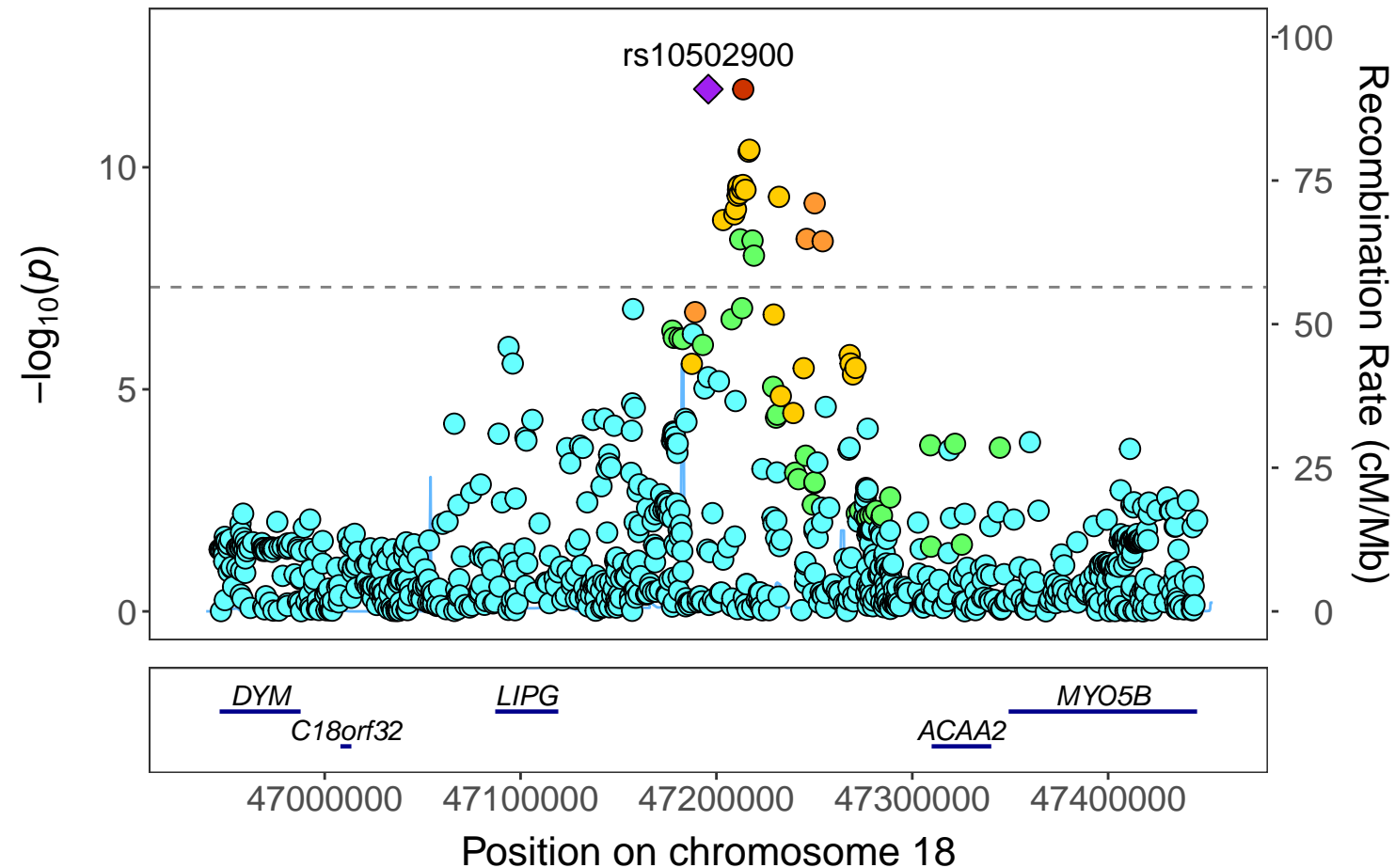

$r^2$     $\circ$  miss    $\circ$  0.0–0.2    $\circ$  0.2–0.4    $\circ$  0.4–0.6    $\circ$  0.6–0.8    $\circ$  0.8–1.0

Supplement: Supplementary file 5 — Supporting Information [file CTM2-16-e70732-s001.zip › LocusZoom/Sfig_rs10502900_locusZoom.pdf]

# LocusZoom plots of GWAS top lead SNP

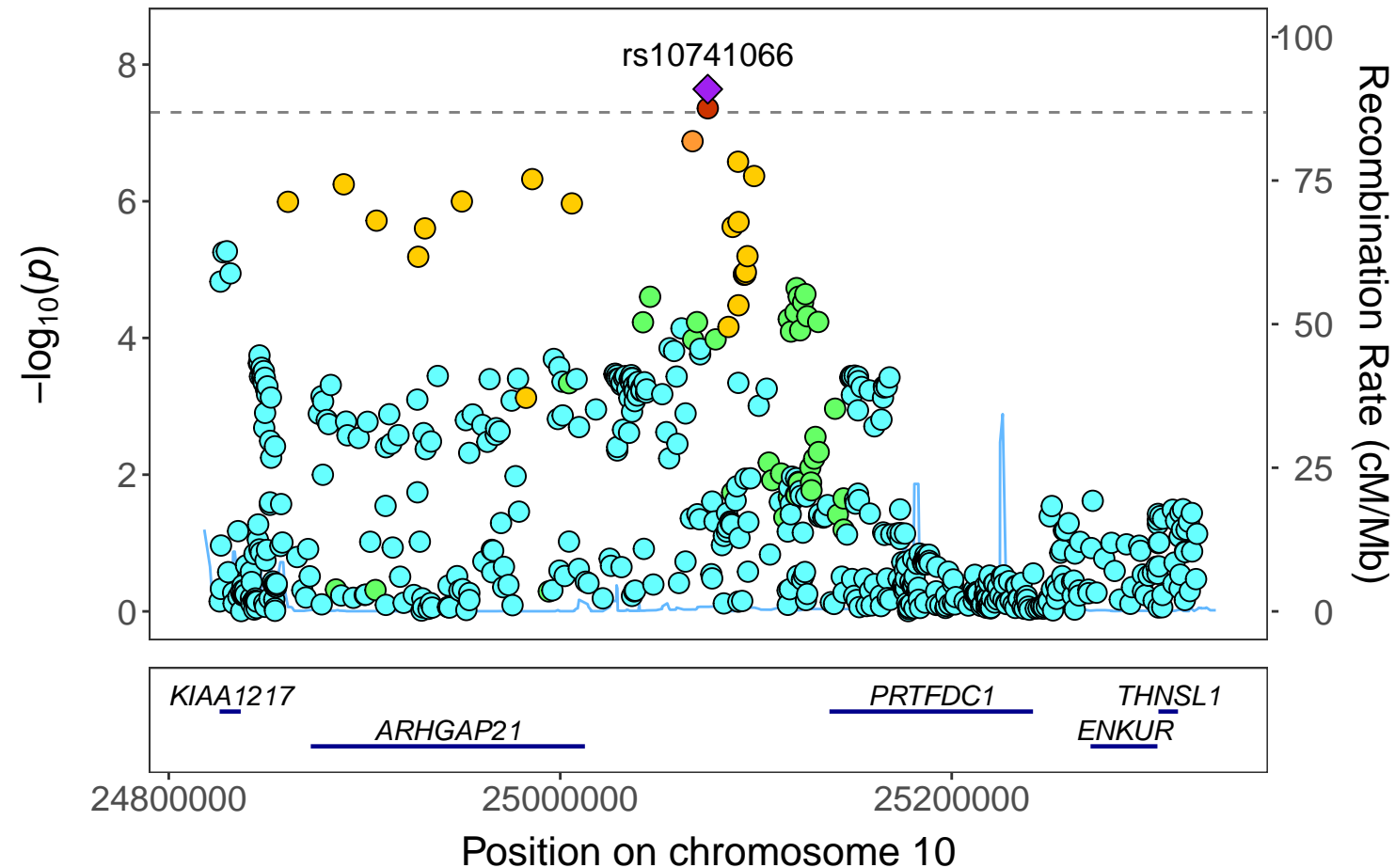

r2   miss   0.0–0.2   0.2–0.4   0.4–0.6   0.6–0.8   0.8–1.0

Supplement: Supplementary file 5 — Supporting Information [file CTM2-16-e70732-s001.zip › LocusZoom/Sfig_rs10741066_locusZoom.pdf]

# *LocusZoom plots of GWAS top lead SNP*

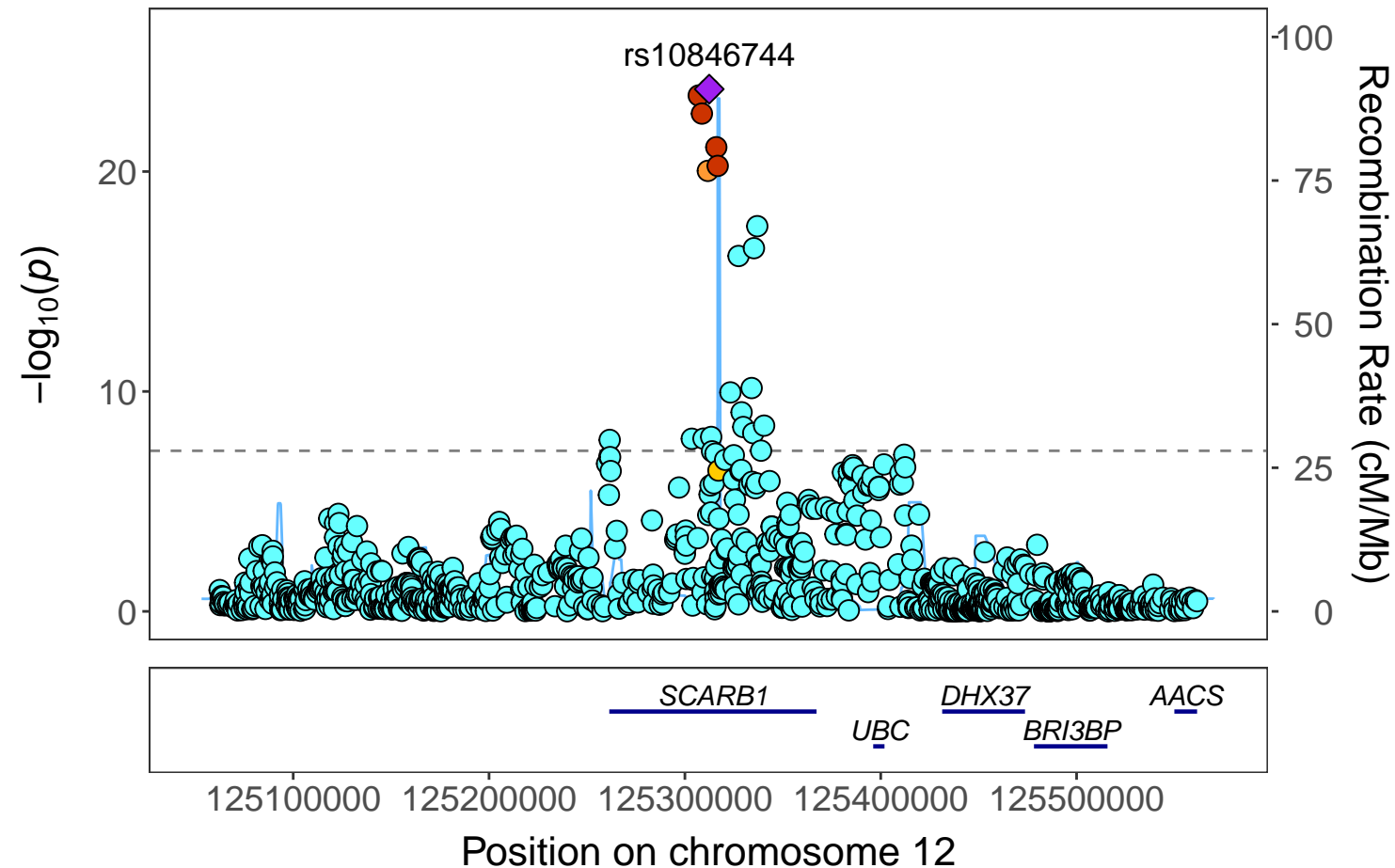

r2   miss   0.0–0.2   0.2–0.4   0.4–0.6   0.6–0.8   0.8–1.0

Supplement: Supplementary file 5 — Supporting Information [file CTM2-16-e70732-s001.zip › LocusZoom/Sfig_rs10846744_locusZoom.pdf]

# LocusZoom plots of GWAS top lead SNP

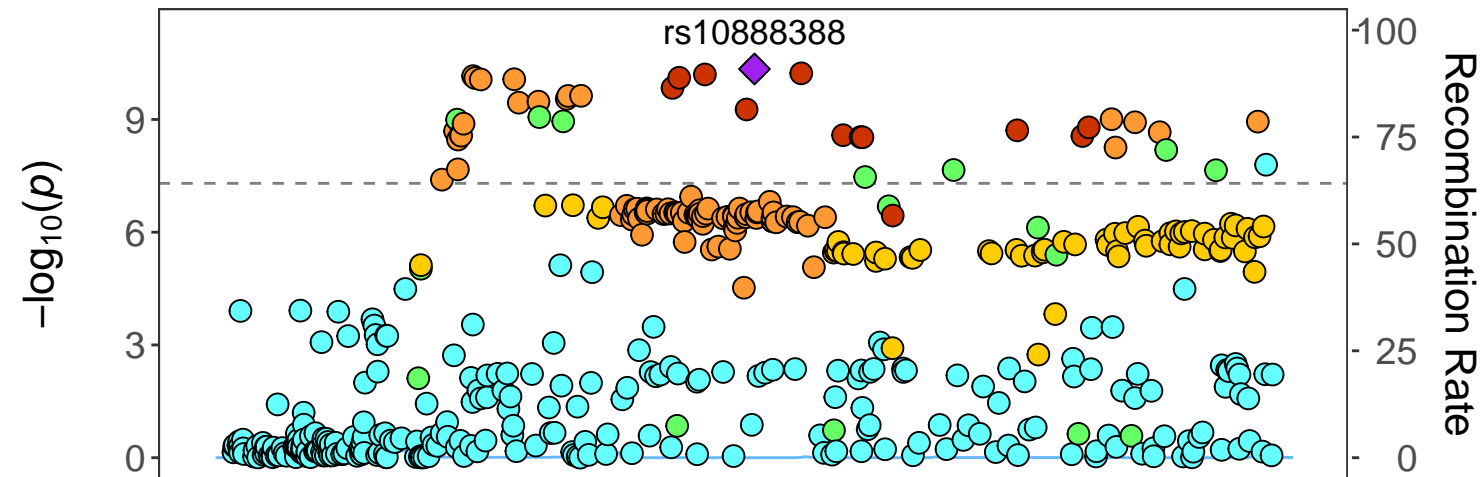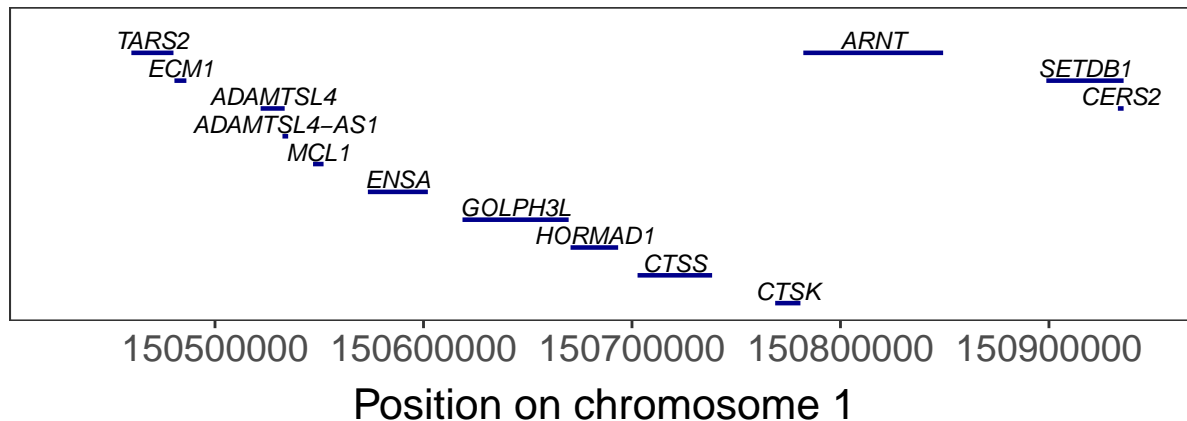

r2    miss    0.0–0.2    0.2–0.4    0.4–0.6    0.6–0.8    0.8–1.0

Supplement: Supplementary file 5 — Supporting Information [file CTM2-16-e70732-s001.zip › LocusZoom/Sfig_rs10888388_locusZoom.pdf]

# LocusZoom plots of GWAS top lead SNP

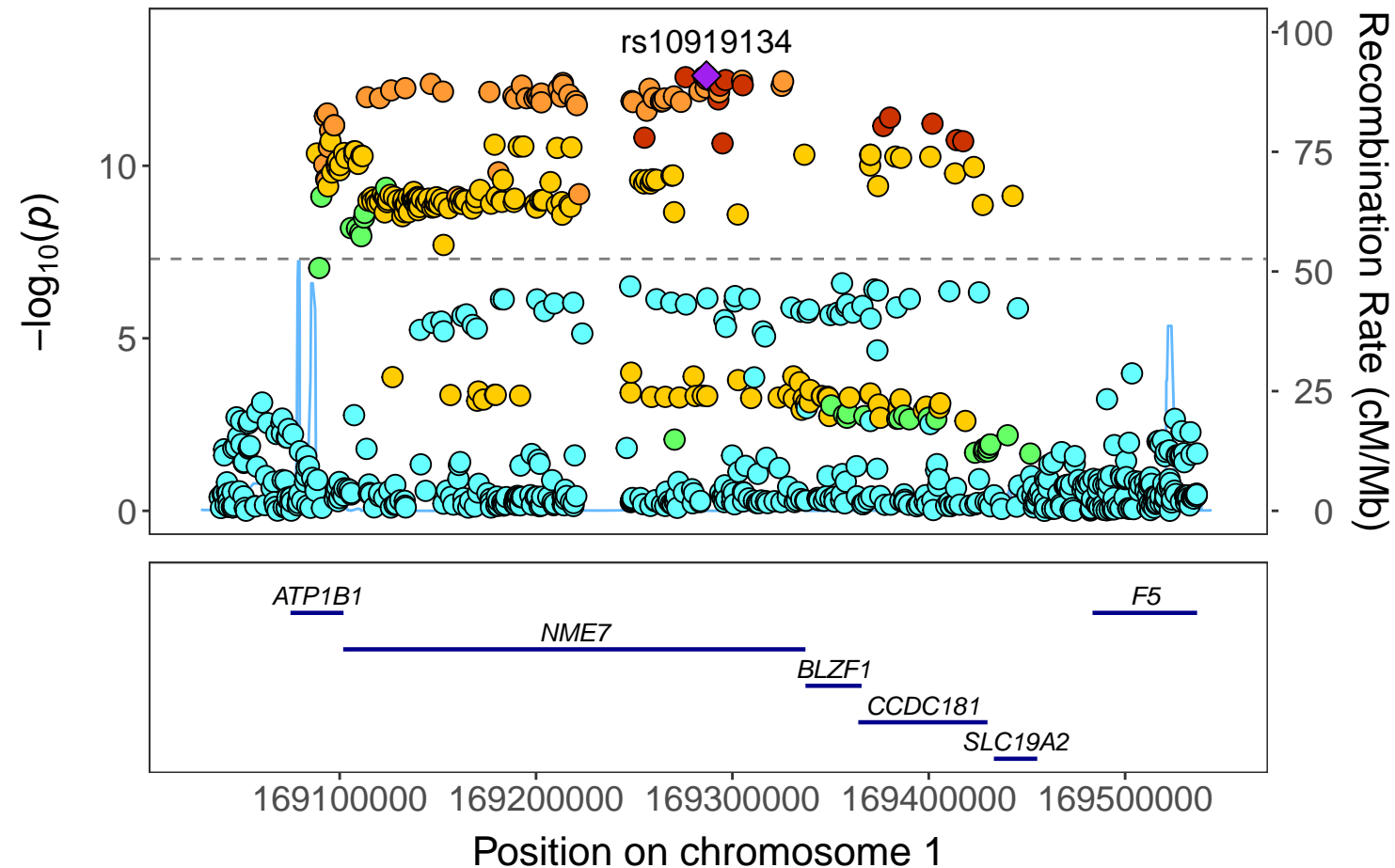

Supplement: Supplementary file 5 — Supporting Information [file CTM2-16-e70732-s001.zip › LocusZoom/Sfig_rs10919134_locusZoom.pdf]

# *LocusZoom plots of GWAS top lead SNP*

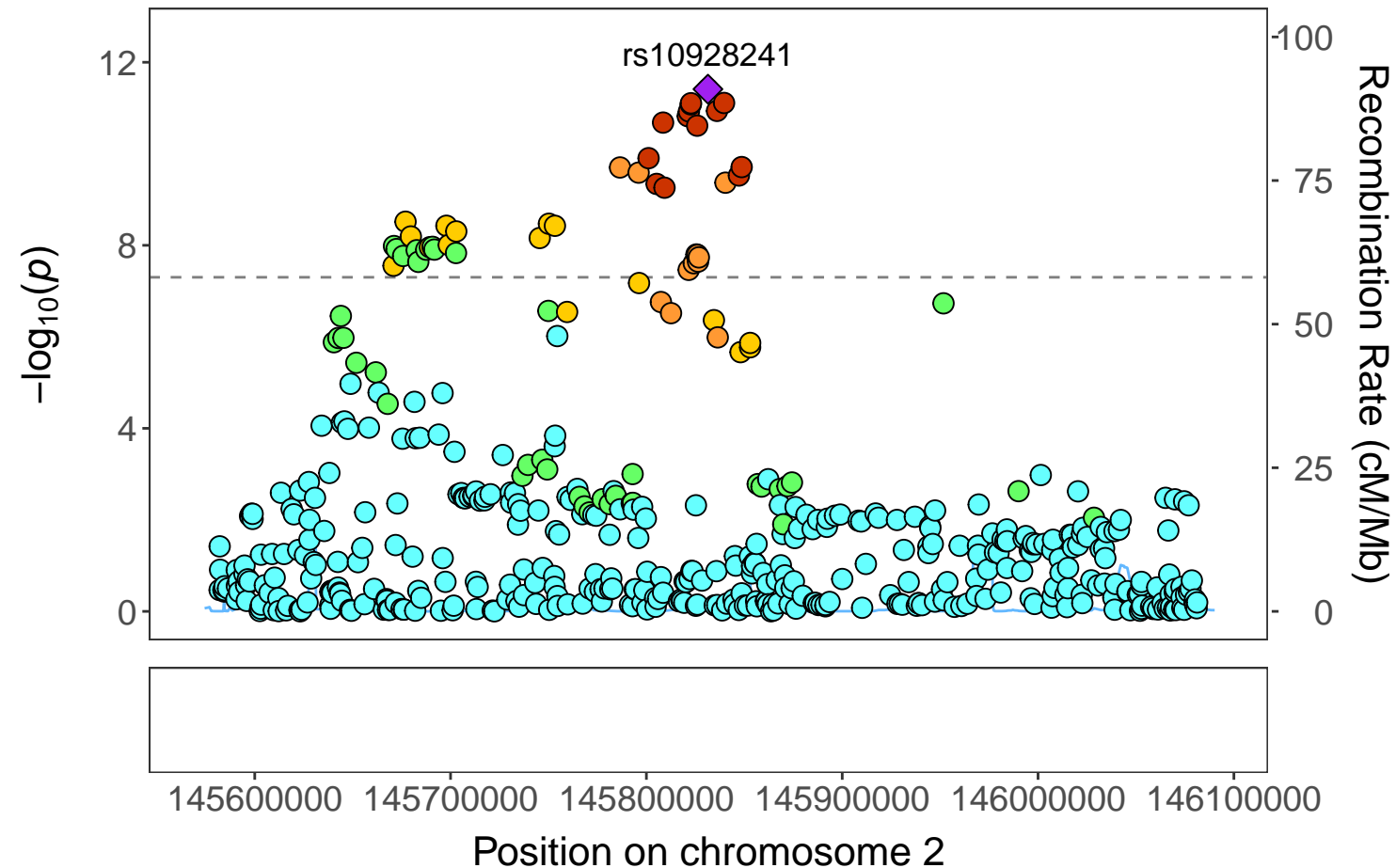

r2   miss   0.0–0.2   0.2–0.4   0.4–0.6   0.6–0.8   0.8–1.0

Supplement: Supplementary file 5 — Supporting Information [file CTM2-16-e70732-s001.zip › LocusZoom/Sfig_rs10928241_locusZoom.pdf]

# LocusZoom plots of GWAS top lead SNP

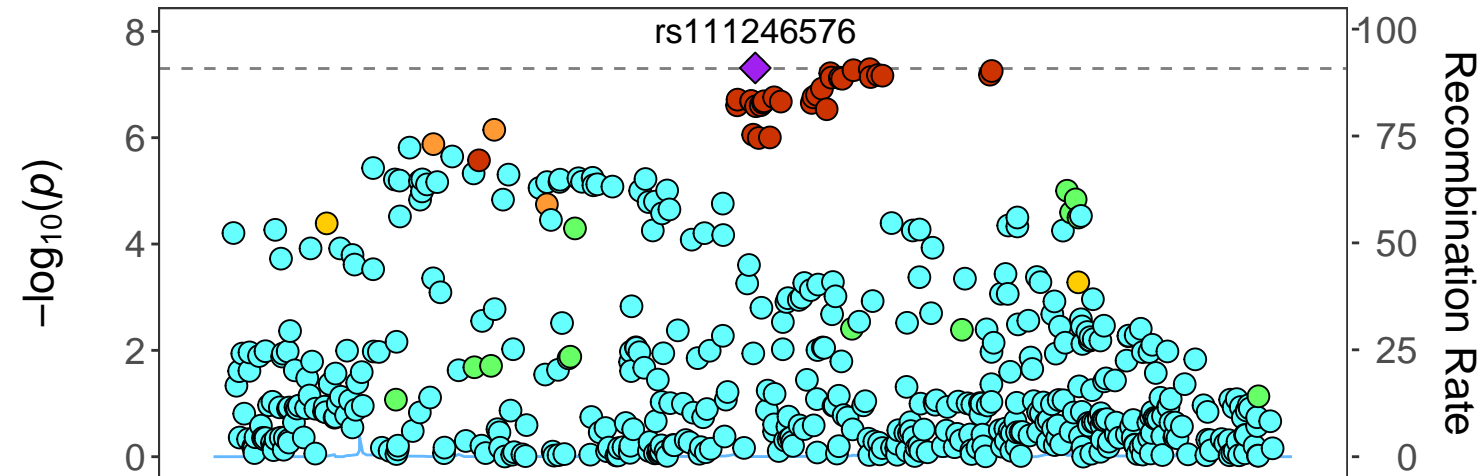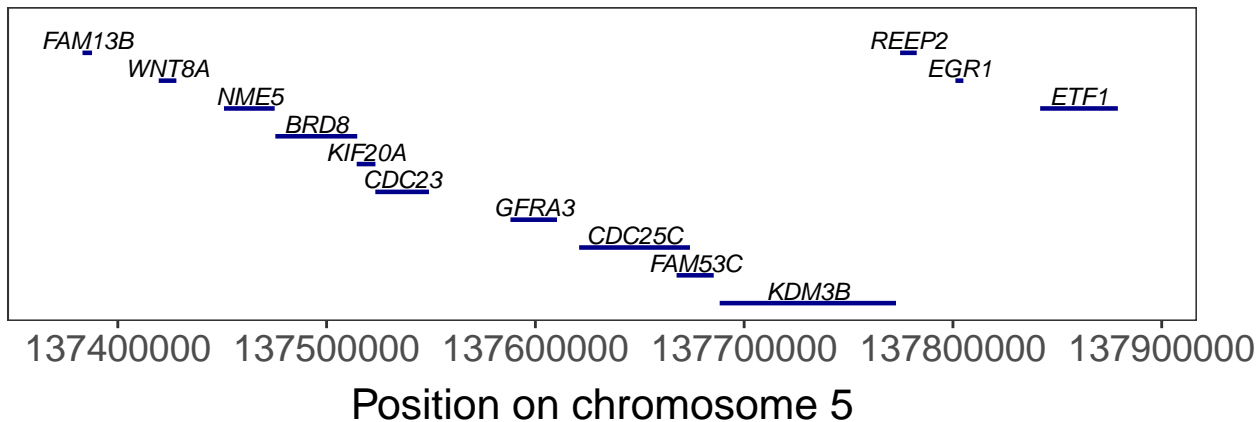

$r^2$     $\circ$  miss    $\circ$  0.0–0.2    $\circ$  0.2–0.4    $\circ$  0.4–0.6    $\circ$  0.6–0.8    $\circ$  0.8–1.0

Supplement: Supplementary file 5 — Supporting Information [file CTM2-16-e70732-s001.zip › LocusZoom/Sfig_rs111246576_locusZoom.pdf]

# LocusZoom plots of GWAS top lead SNP

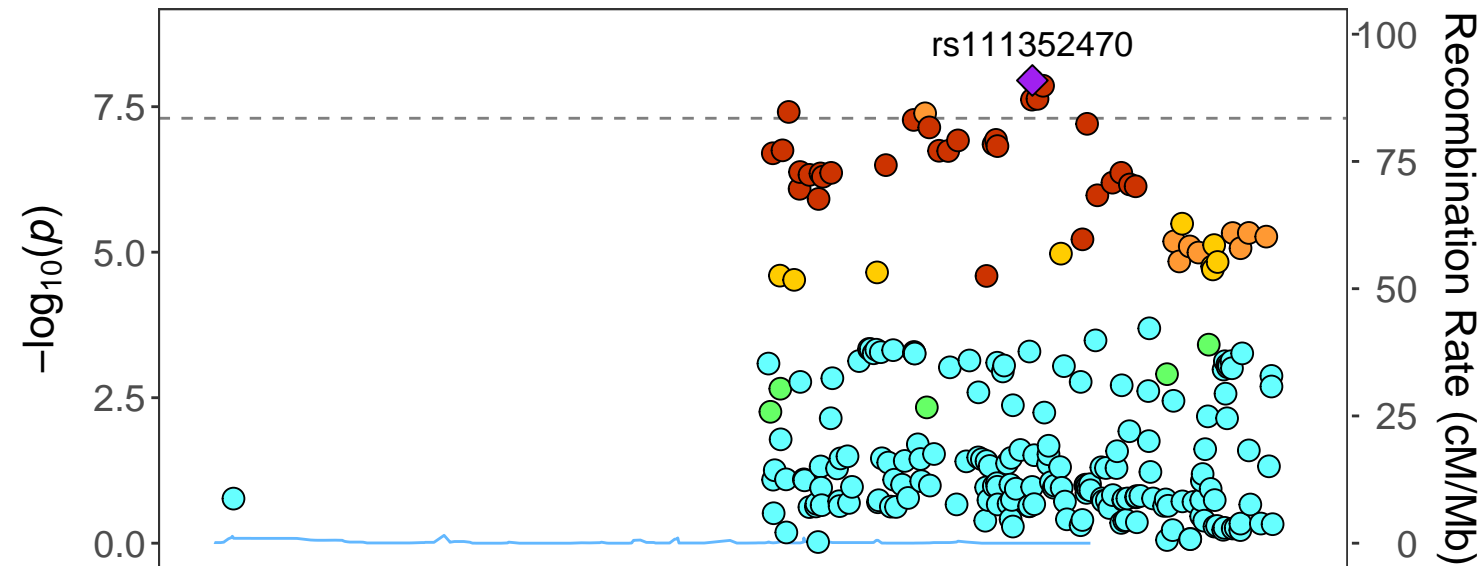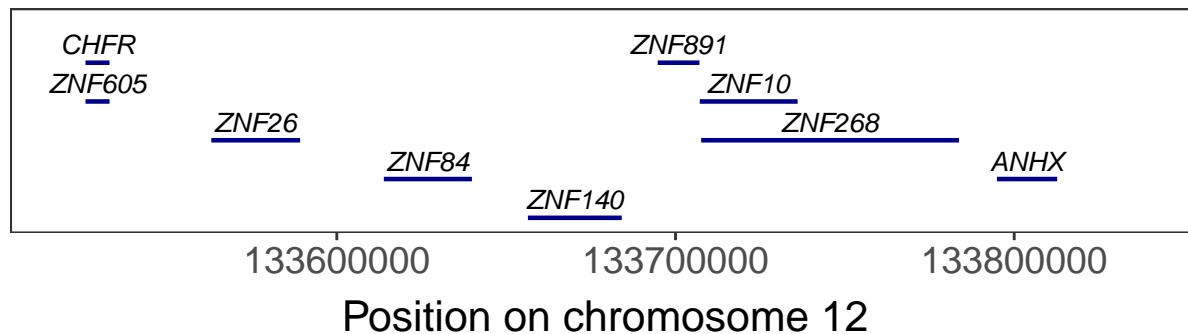

r2   miss   0.0–0.2   0.2–0.4   0.4–0.6   0.6–0.8   0.8–1.0

Supplement: Supplementary file 5 — Supporting Information [file CTM2-16-e70732-s001.zip › LocusZoom/Sfig_rs111352470_locusZoom.pdf]

# LocusZoom plots of GWAS top lead SNP

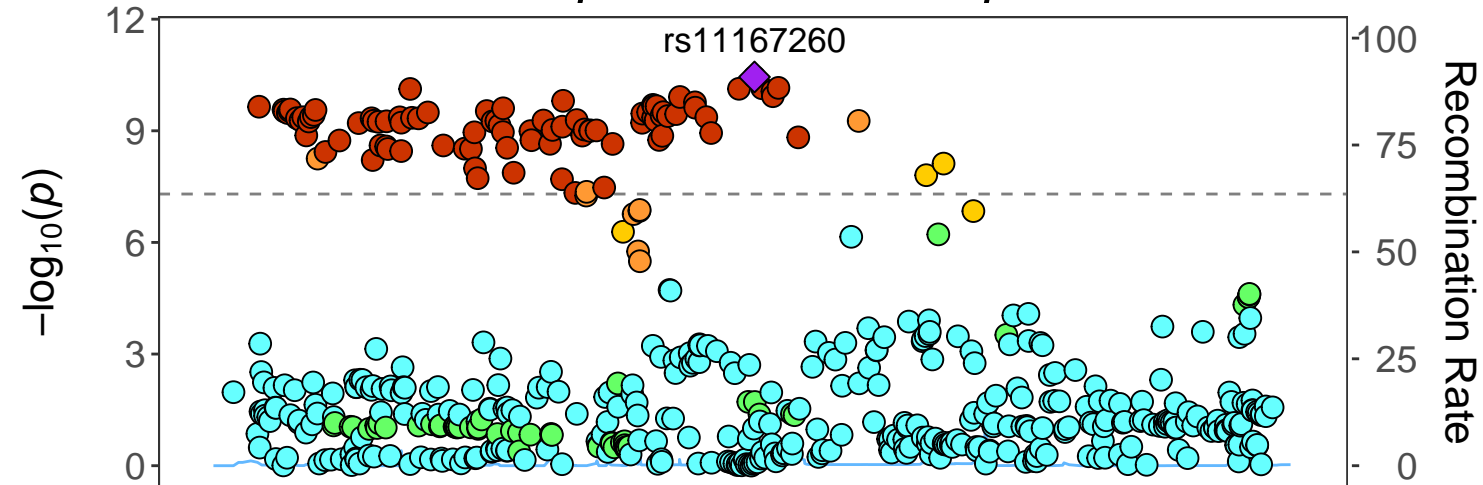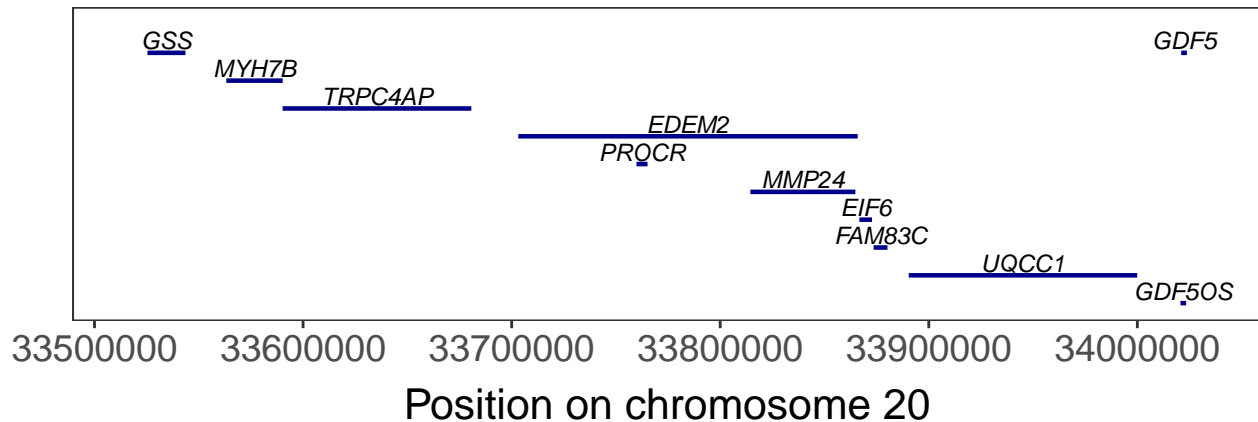

r2   miss   0.0–0.2   0.2–0.4   0.4–0.6   0.6–0.8   0.8–1.0

Supplement: Supplementary file 5 — Supporting Information [file CTM2-16-e70732-s001.zip › LocusZoom/Sfig_rs11167260_locusZoom.pdf]

# LocusZoom plots of GWAS top lead SNP

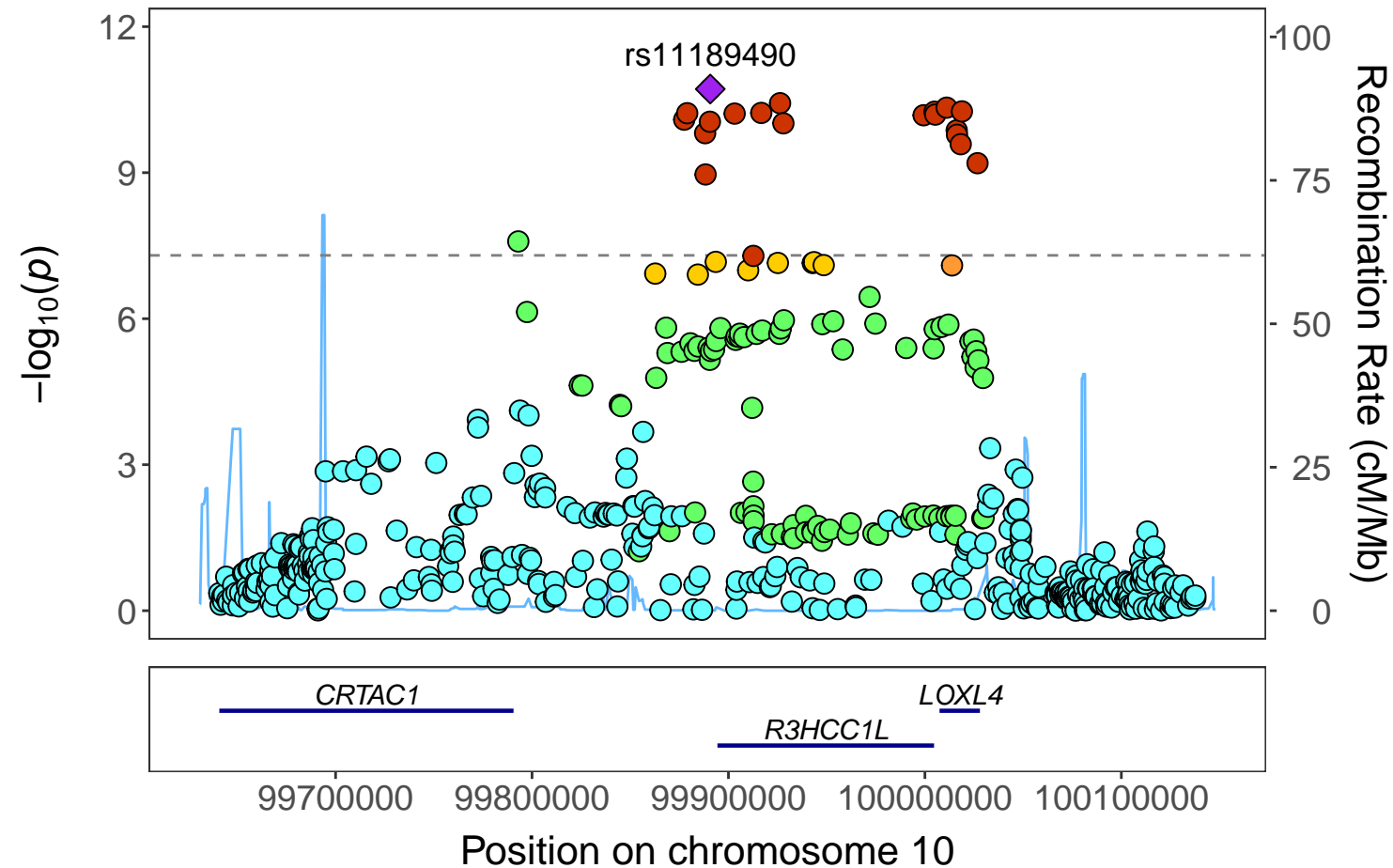

$r^2$     $\circ$  miss    $\circ$  0.0–0.2    $\circ$  0.2–0.4    $\circ$  0.4–0.6    $\circ$  0.6–0.8    $\circ$  0.8–1.0

Supplement: Supplementary file 5 — Supporting Information [file CTM2-16-e70732-s001.zip › LocusZoom/Sfig_rs11189490_locusZoom.pdf]

# LocusZoom plots of GWAS top lead SNP

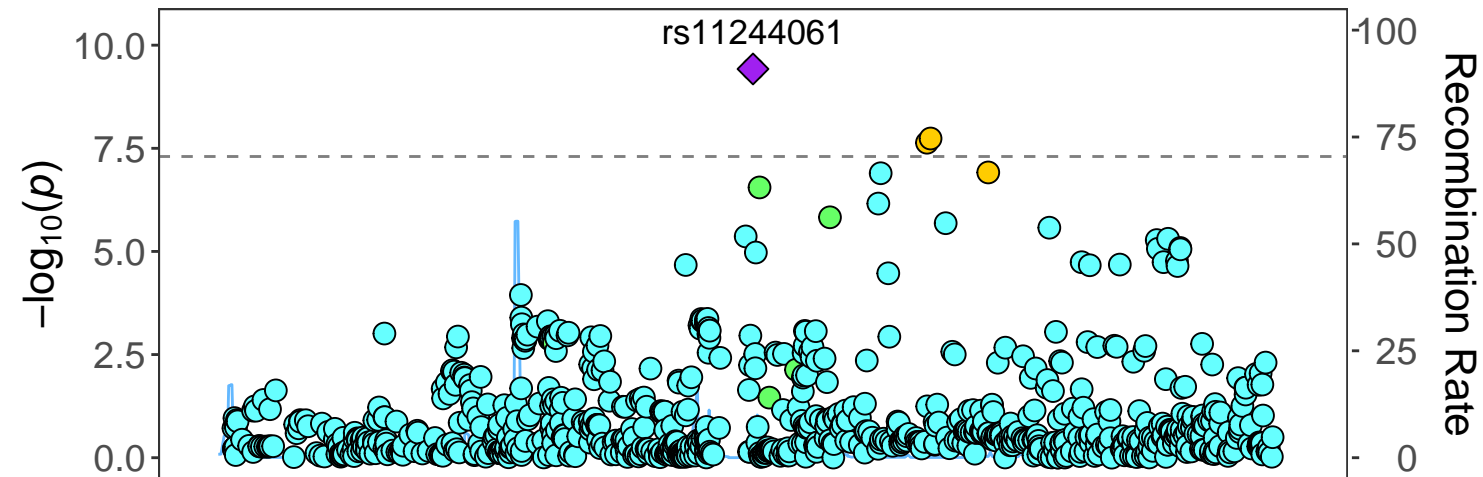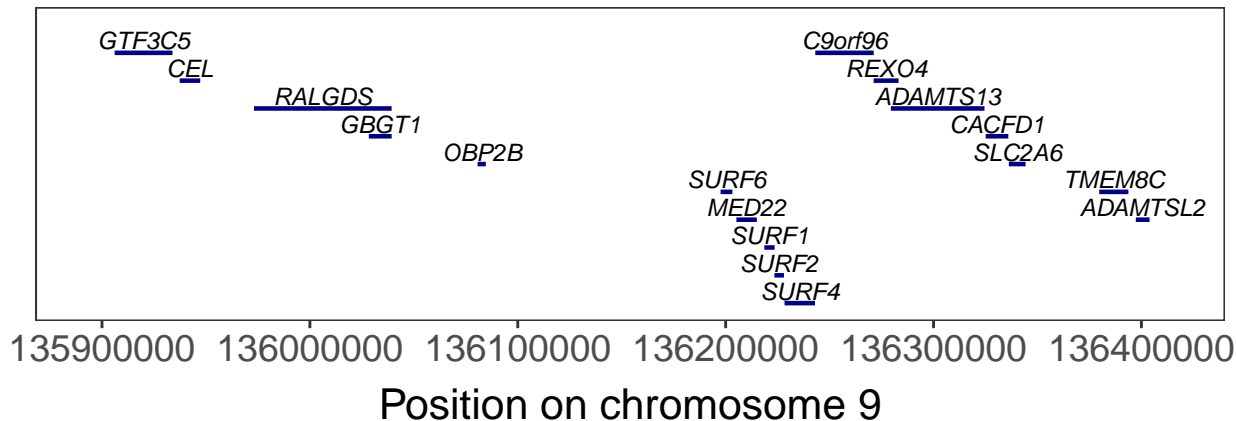

r2   miss   0.0–0.2   0.2–0.4   0.4–0.6   0.6–0.8   0.8–1.0

Supplement: Supplementary file 5 — Supporting Information [file CTM2-16-e70732-s001.zip › LocusZoom/Sfig_rs11244061_locusZoom.pdf]

# *LocusZoom plots of GWAS top lead SNP*

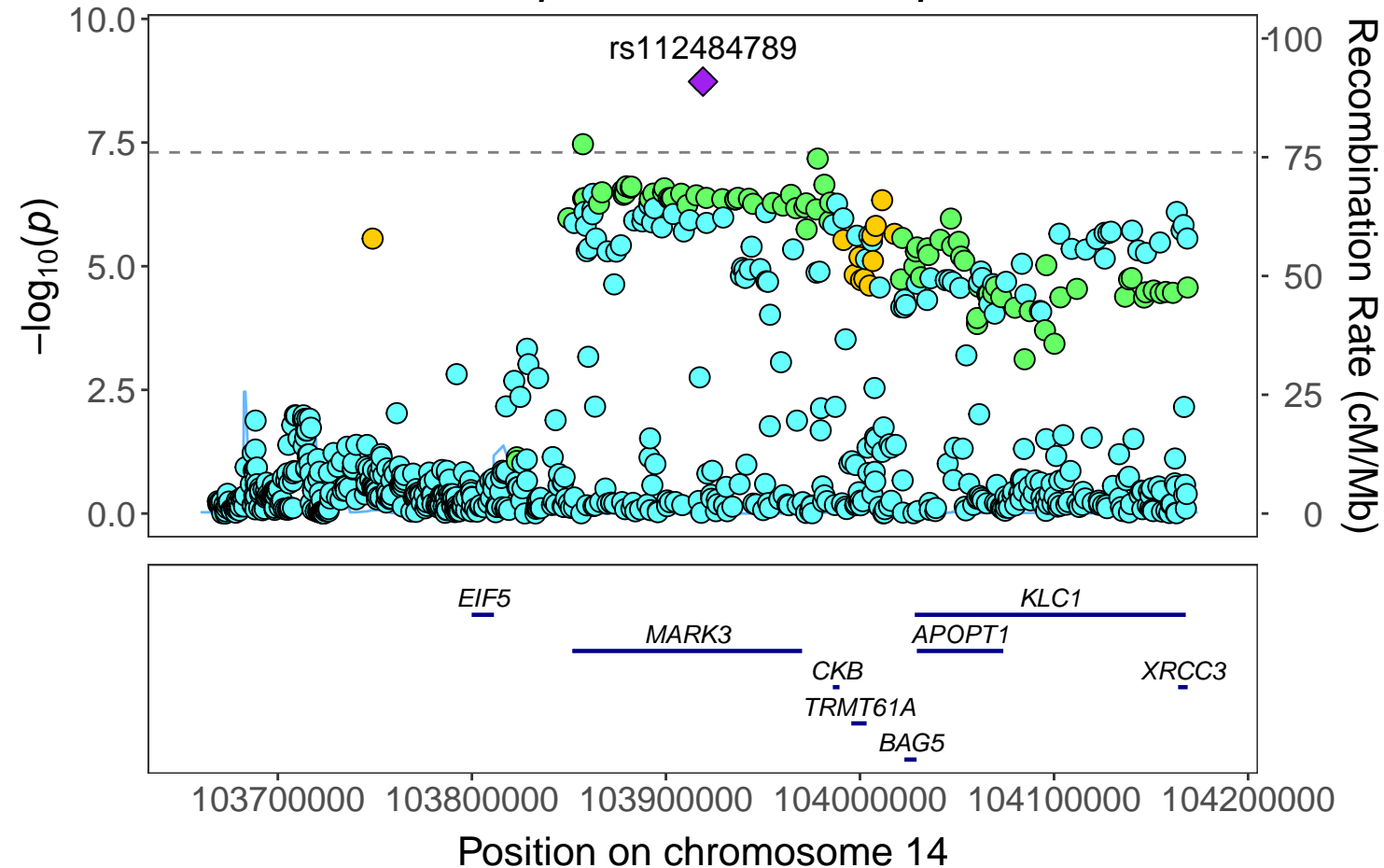

r2   miss   0.0-0.2   0.2-0.4   0.4-0.6   0.6-0.8   0.8-1.0

Supplement: Supplementary file 5 — Supporting Information [file CTM2-16-e70732-s001.zip › LocusZoom/Sfig_rs112484789_locusZoom.pdf]

# LocusZoom plots of GWAS top lead SNP

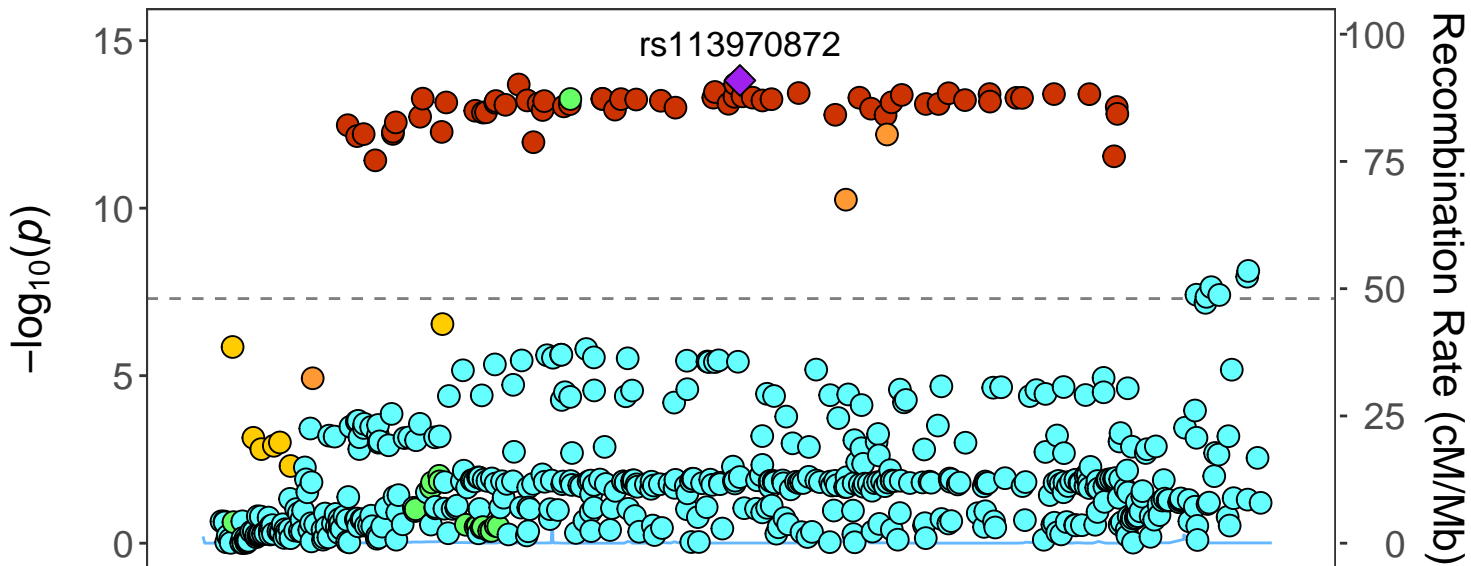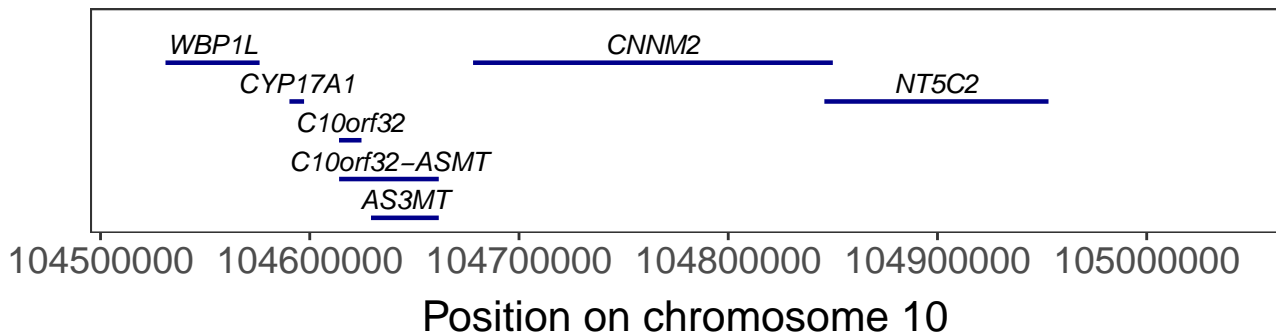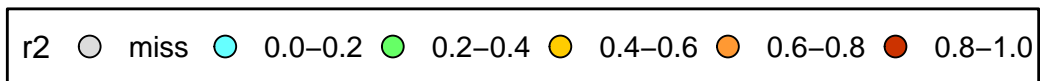

Supplement: Supplementary file 5 — Supporting Information [file CTM2-16-e70732-s001.zip › LocusZoom/Sfig_rs113970872_locusZoom.pdf]

# LocusZoom plots of GWAS top lead SNP

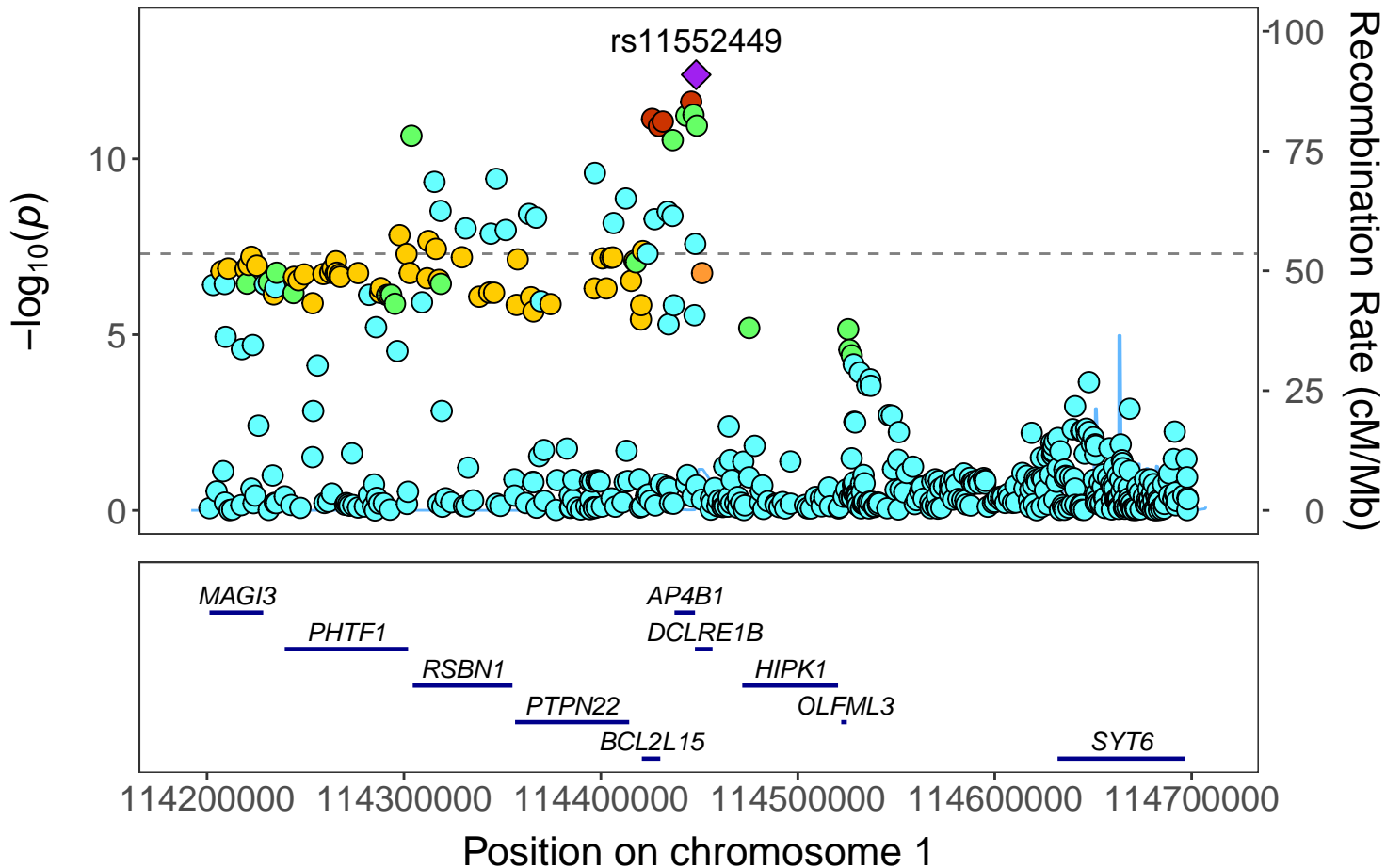

Supplement: Supplementary file 5 — Supporting Information [file CTM2-16-e70732-s001.zip › LocusZoom/Sfig_rs11552449_locusZoom.pdf]

# LocusZoom plots of GWAS top lead SNP

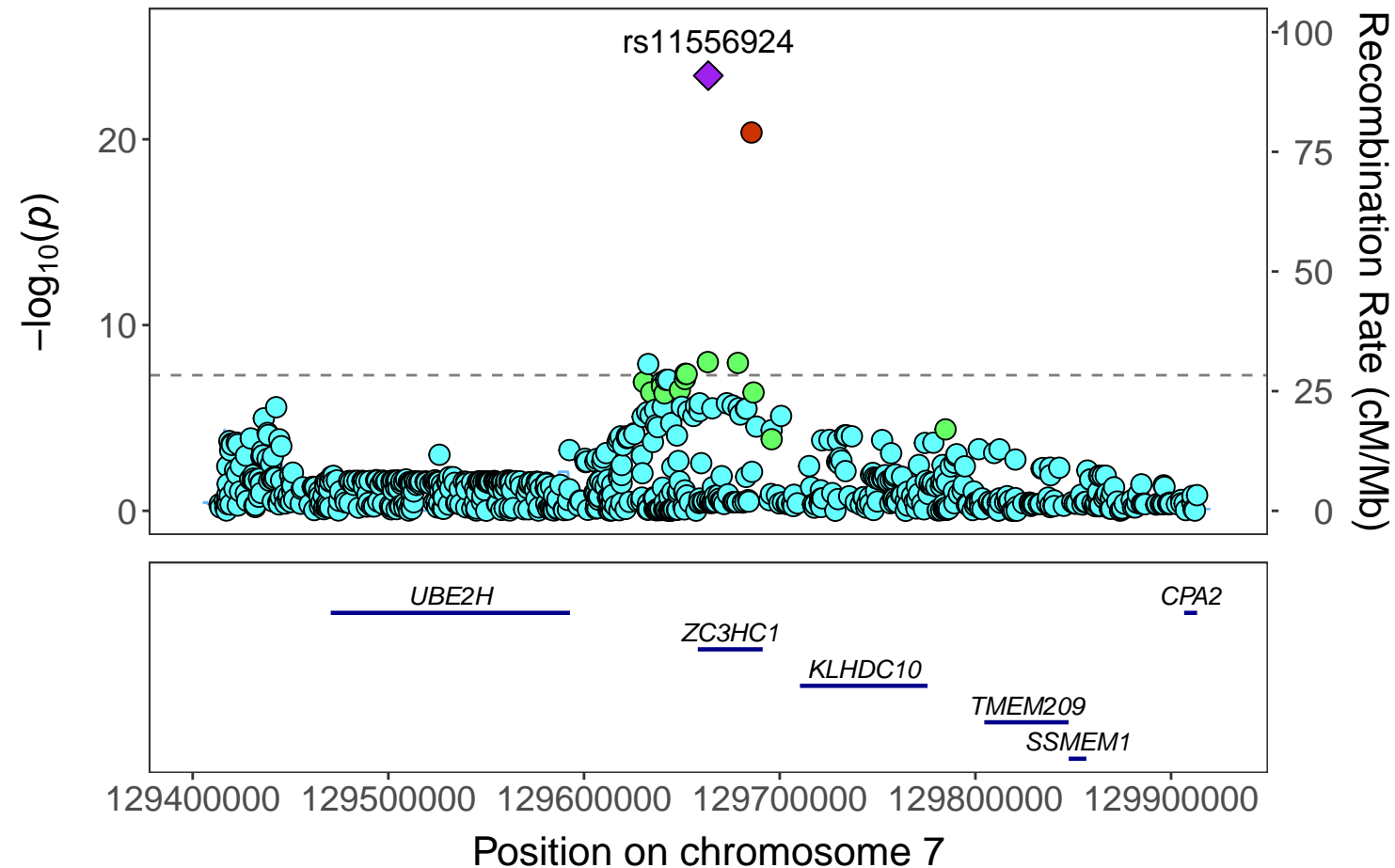

r2   miss   0.0–0.2   0.2–0.4   0.4–0.6   0.6–0.8   0.8–1.0

Supplement: Supplementary file 5 — Supporting Information [file CTM2-16-e70732-s001.zip › LocusZoom/Sfig_rs11556924_locusZoom.pdf]

# LocusZoom plots of GWAS top lead SNP

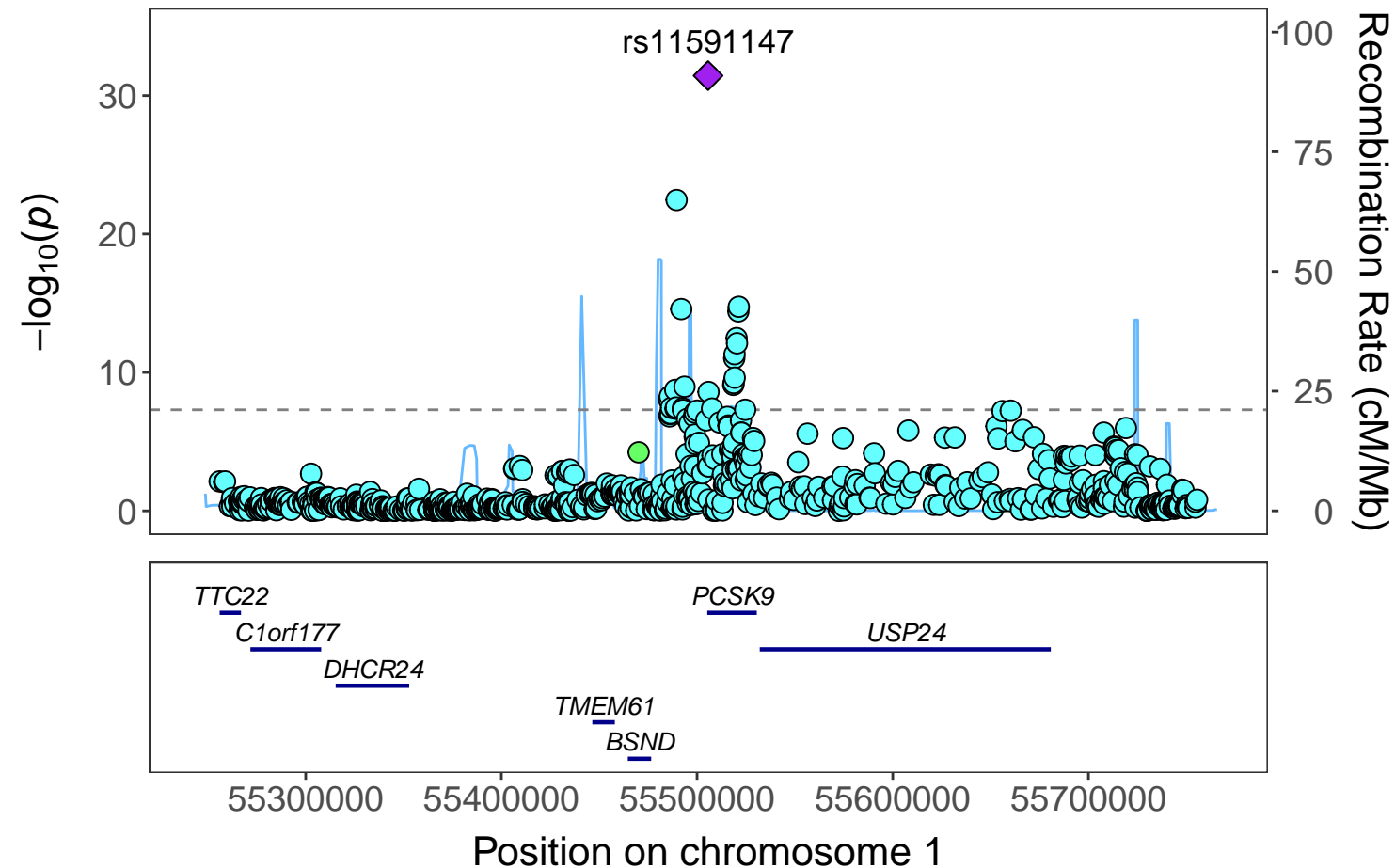

Supplement: Supplementary file 5 — Supporting Information [file CTM2-16-e70732-s001.zip › LocusZoom/Sfig_rs11591147_locusZoom.pdf]

# LocusZoom plots of GWAS top lead SNP

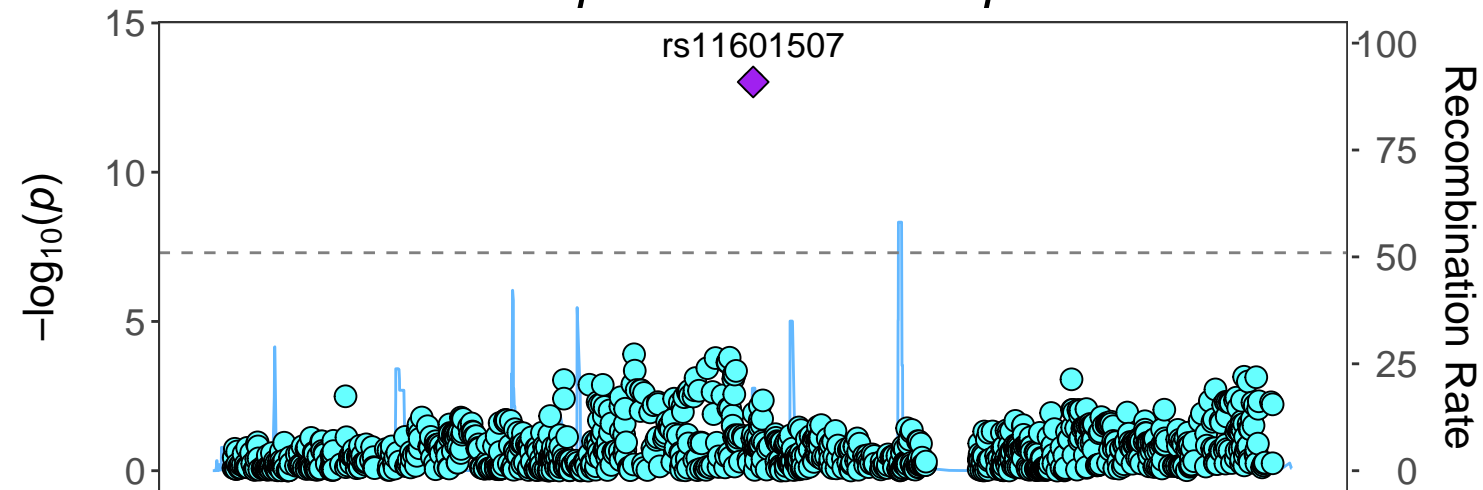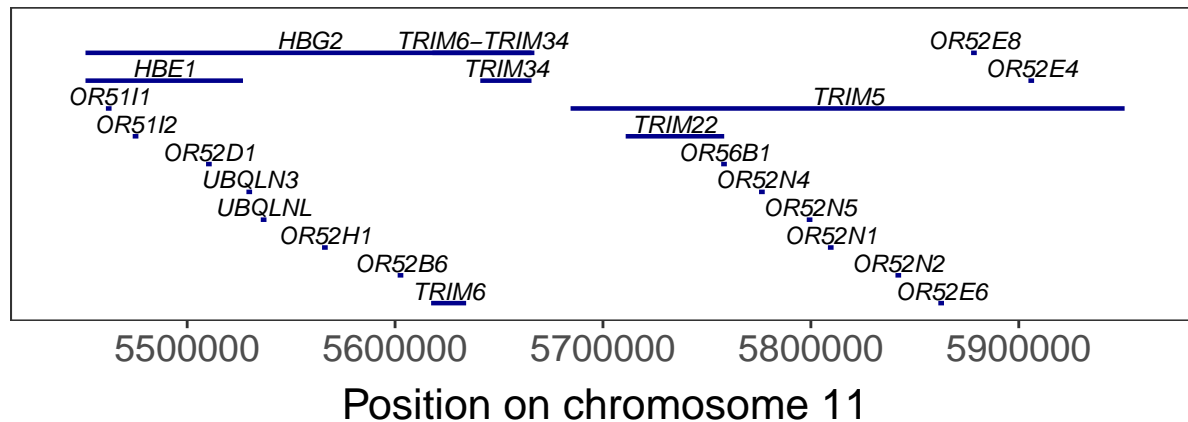

$r^2$  ○ miss ● 0.0–0.2 ● 0.2–0.4 ● 0.4–0.6 ● 0.6–0.8 ● 0.8–1.0

Supplement: Supplementary file 5 — Supporting Information [file CTM2-16-e70732-s001.zip › LocusZoom/Sfig_rs11601507_locusZoom.pdf]

# LocusZoom plots of GWAS top lead SNP

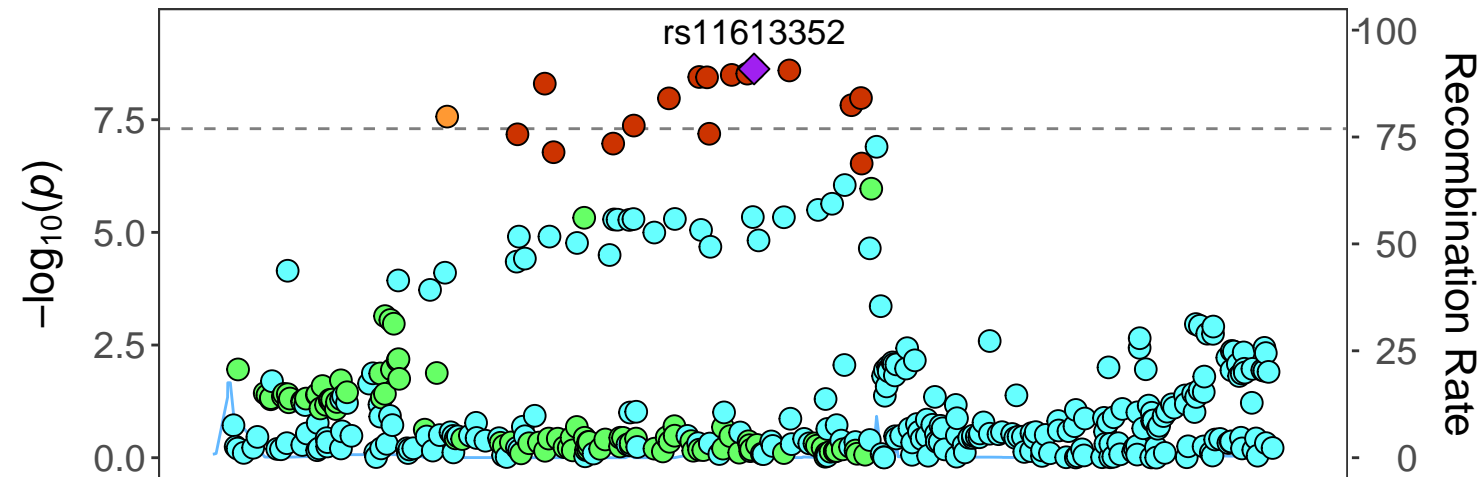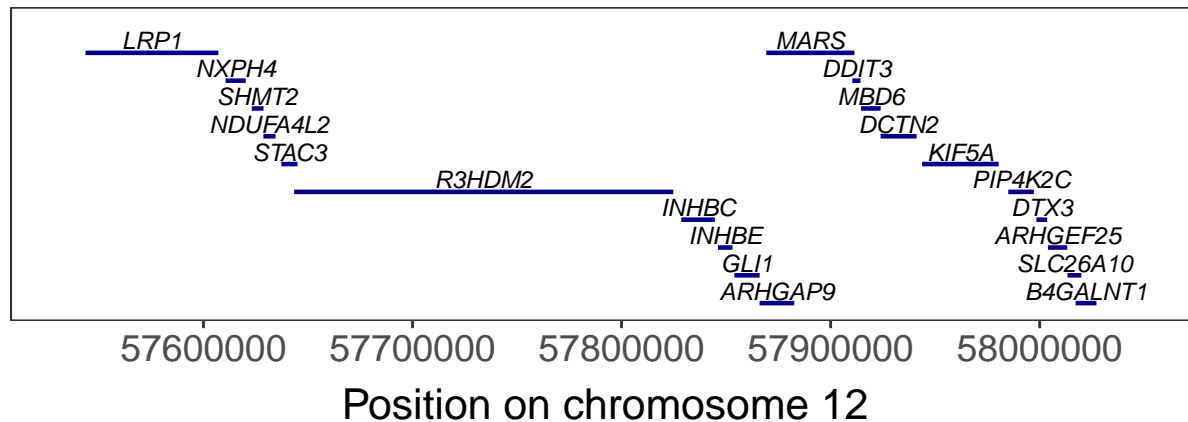

r2    ○   miss    ●   0.0–0.2    ●   0.2–0.4    ●   0.4–0.6    ●   0.6–0.8    ●   0.8–1.0

Supplement: Supplementary file 5 — Supporting Information [file CTM2-16-e70732-s001.zip › LocusZoom/Sfig_rs11613352_locusZoom.pdf]

# LocusZoom plots of GWAS top lead SNP

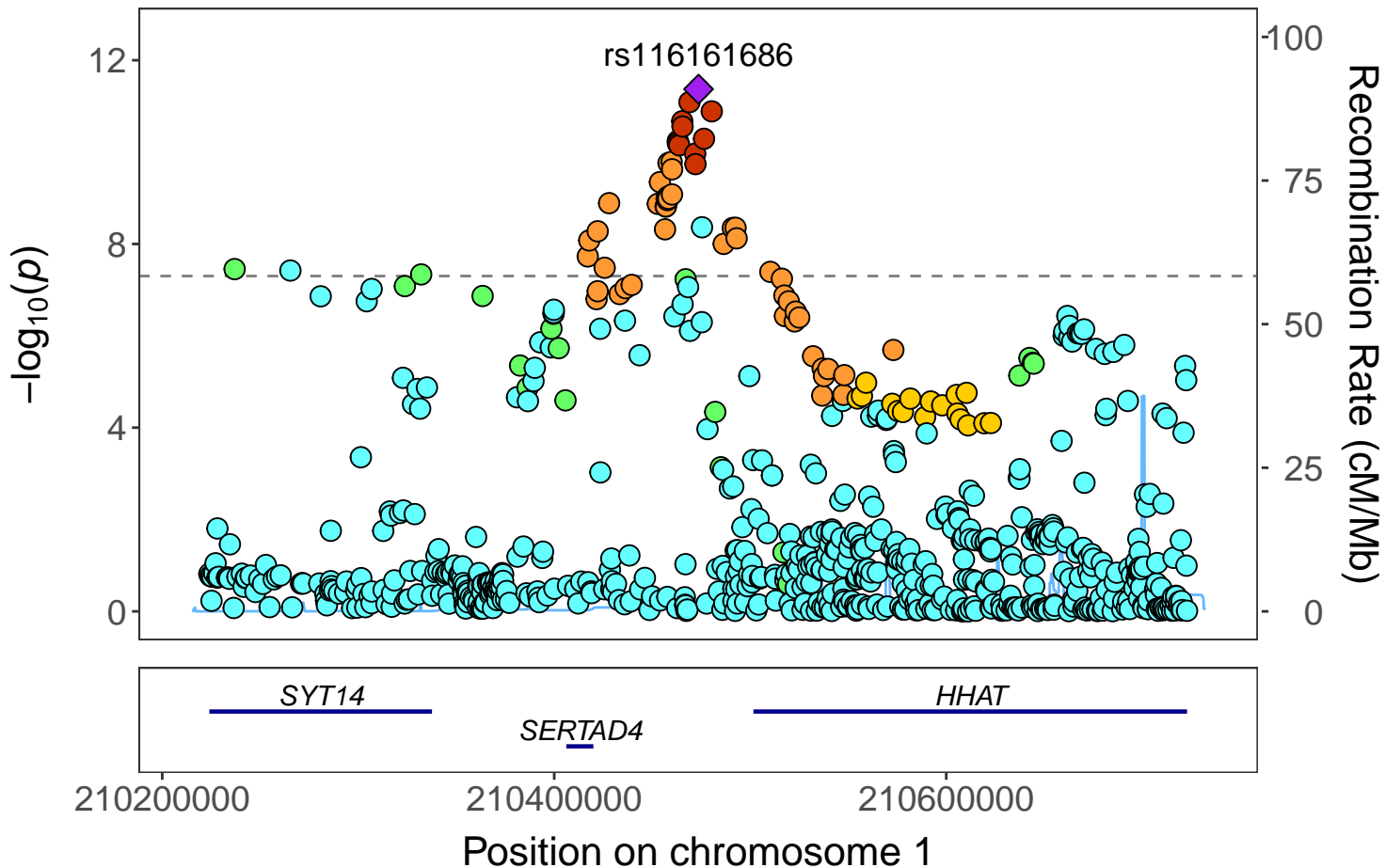

$r^2$    miss   0.0–0.2   0.2–0.4   0.4–0.6   0.6–0.8   0.8–1.0

Supplement: Supplementary file 5 — Supporting Information [file CTM2-16-e70732-s001.zip › LocusZoom/Sfig_rs116161686_locusZoom.pdf]

# *LocusZoom plots of GWAS top lead SNP*

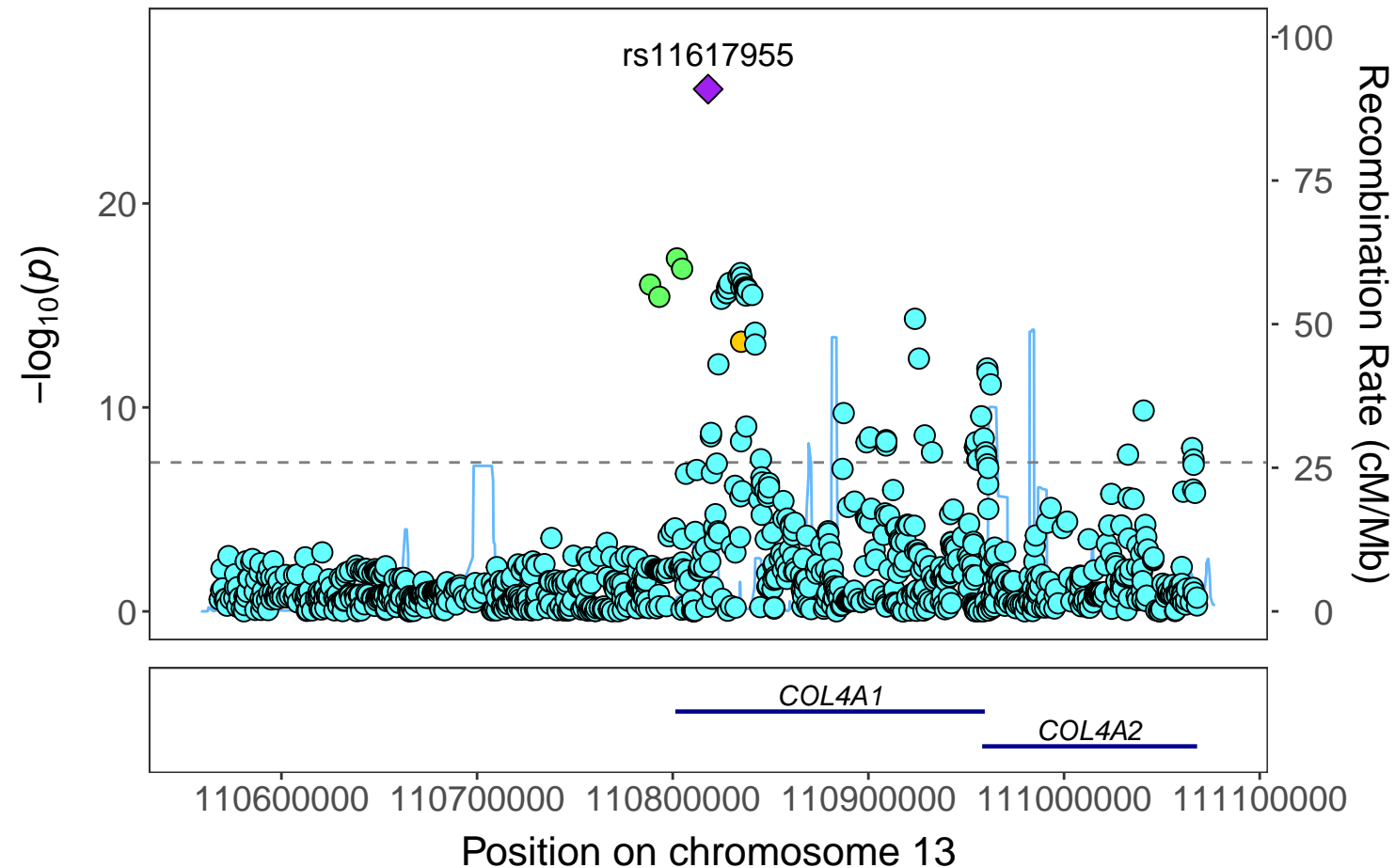

$r^2$    miss   0.0–0.2   0.2–0.4   0.4–0.6   0.6–0.8   0.8–1.0

Supplement: Supplementary file 5 — Supporting Information [file CTM2-16-e70732-s001.zip › LocusZoom/Sfig_rs11617955_locusZoom.pdf]

# LocusZoom plots of GWAS top lead SNP

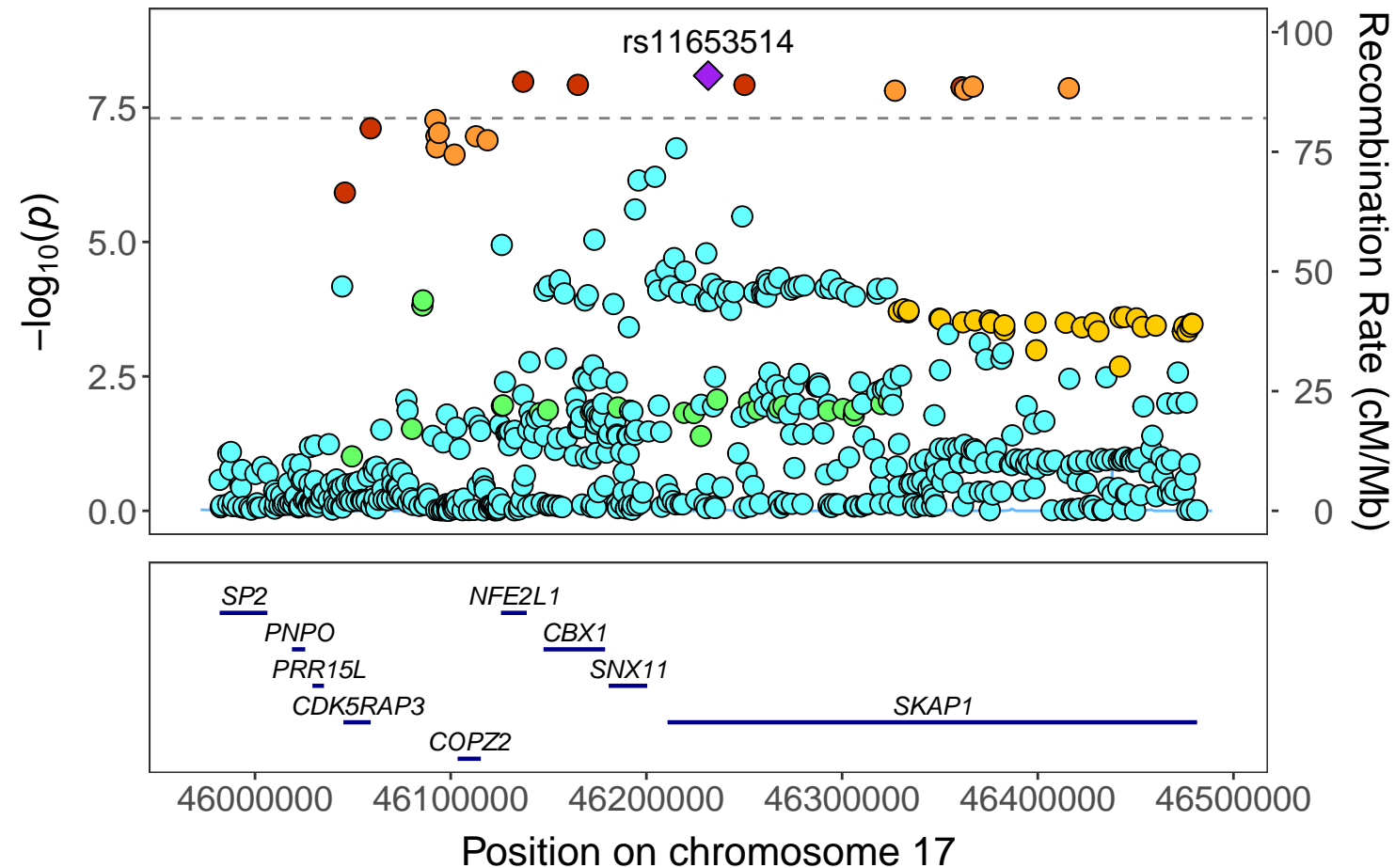

$r^2$    miss   cyan   0.0–0.2   green   0.2–0.4   yellow   0.4–0.6   orange   0.6–0.8   red   0.8–1.0

Supplement: Supplementary file 5 — Supporting Information [file CTM2-16-e70732-s001.zip › LocusZoom/Sfig_rs11653514_locusZoom.pdf]

# LocusZoom plots of GWAS top lead SNP

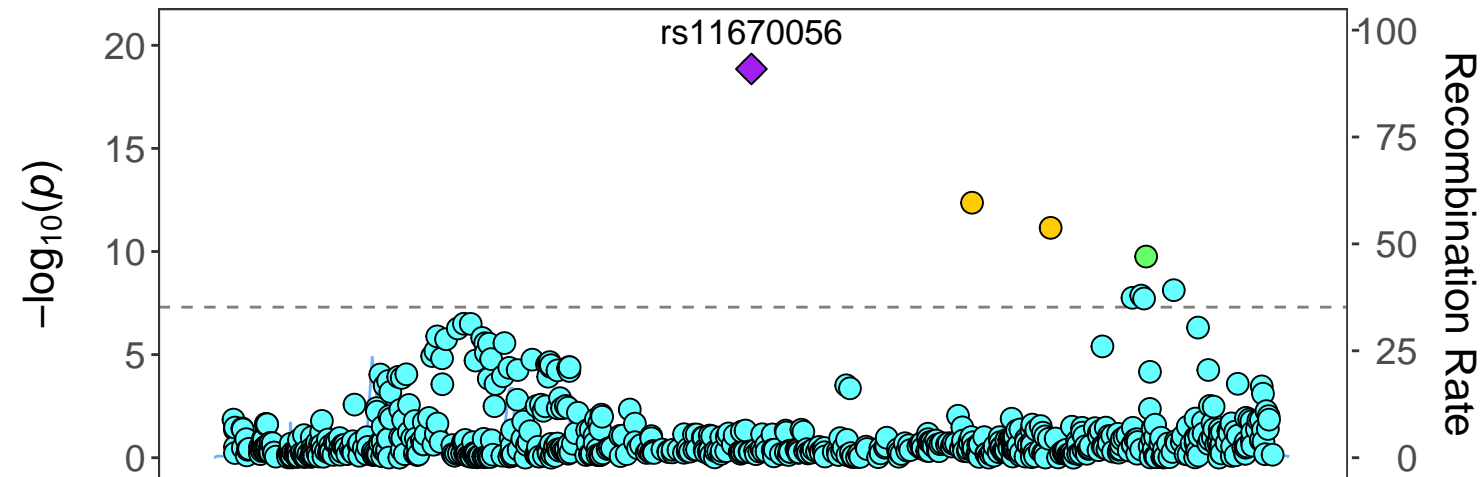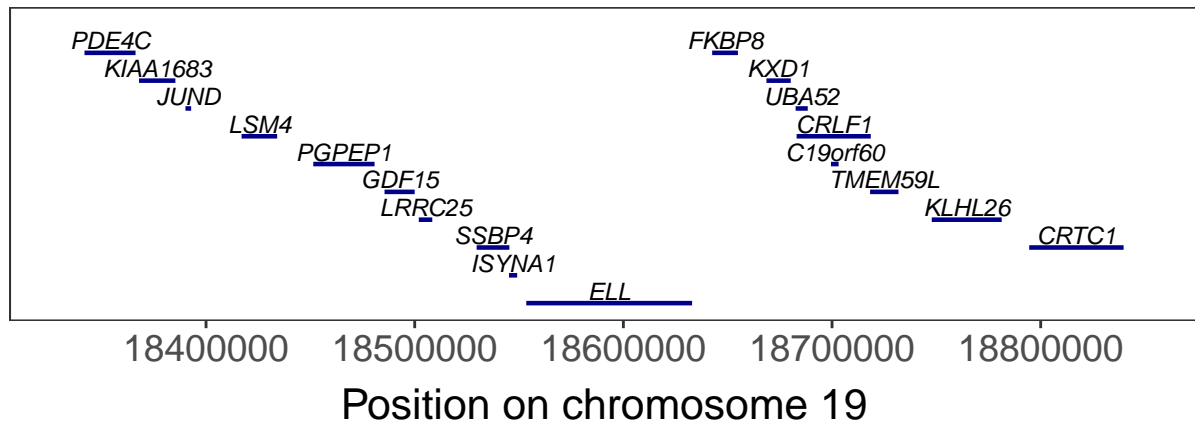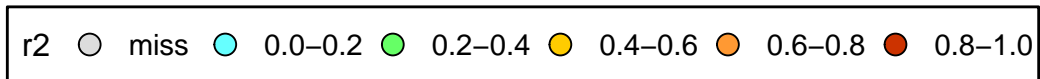

Supplement: Supplementary file 5 — Supporting Information [file CTM2-16-e70732-s001.zip › LocusZoom/Sfig_rs11670056_locusZoom.pdf]

# LocusZoom plots of GWAS top lead SNP

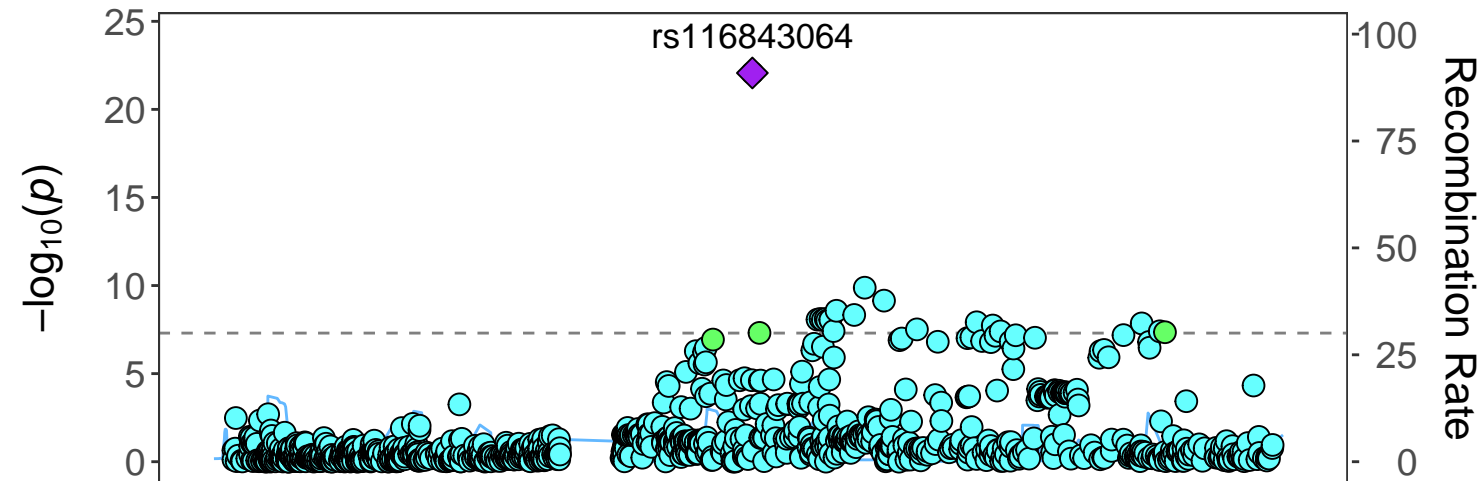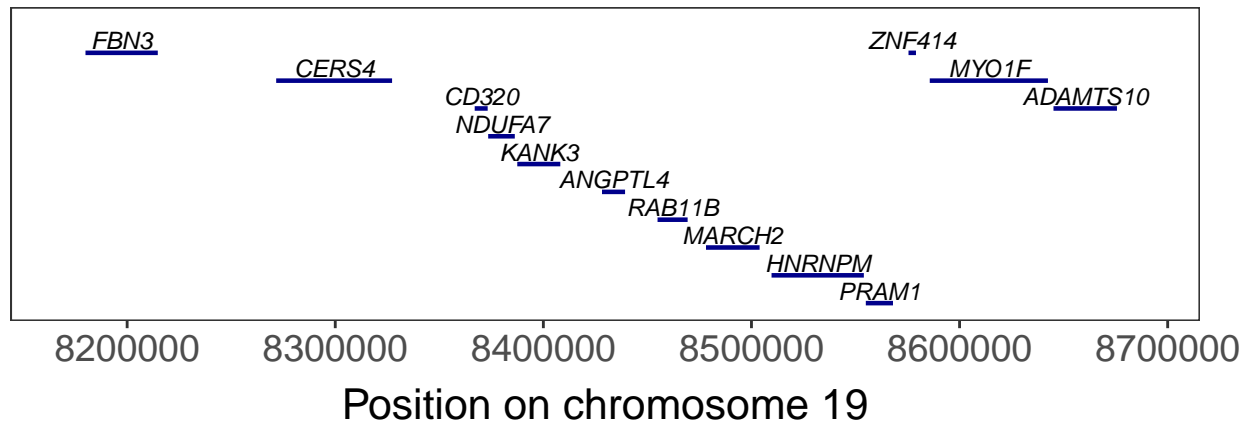

$r^2$  ○ miss ● 0.0–0.2 ● 0.2–0.4 ● 0.4–0.6 ● 0.6–0.8 ● 0.8–1.0

Supplement: Supplementary file 5 — Supporting Information [file CTM2-16-e70732-s001.zip › LocusZoom/Sfig_rs116843064_locusZoom.pdf]

# *LocusZoom plots of GWAS top lead SNP*

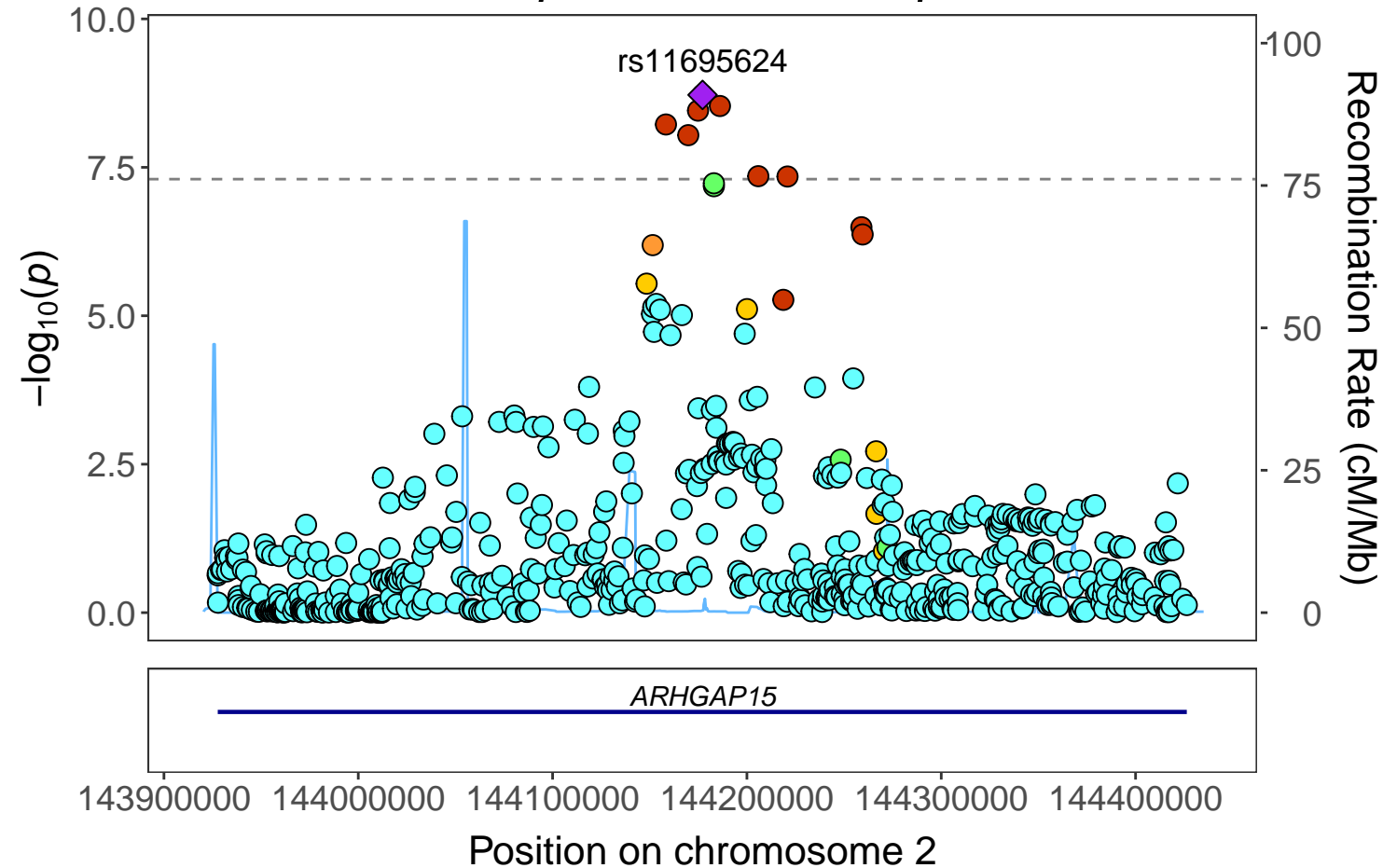

Supplement: Supplementary file 5 — Supporting Information [file CTM2-16-e70732-s001.zip › LocusZoom/Sfig_rs11695624_locusZoom.pdf]

# LocusZoom plots of GWAS top lead SNP

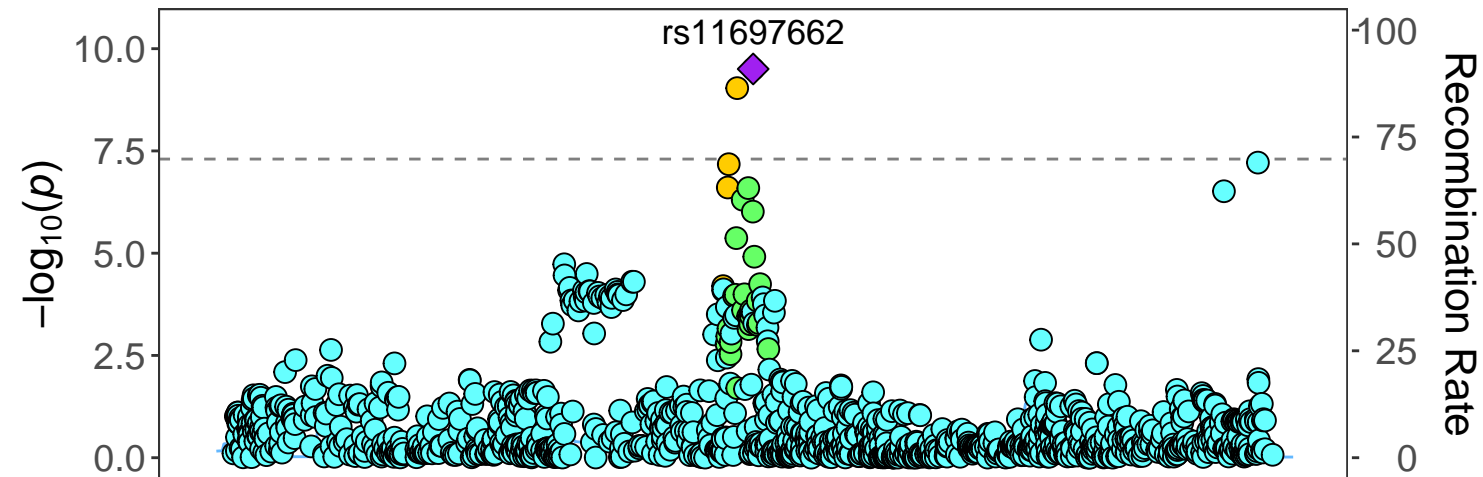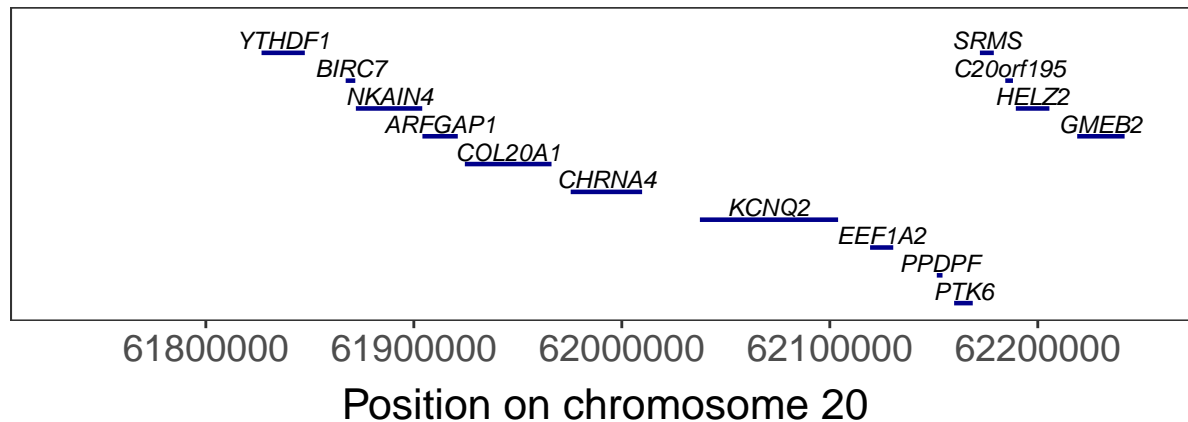

r2   miss   0.0–0.2   0.2–0.4   0.4–0.6   0.6–0.8   0.8–1.0

Supplement: Supplementary file 5 — Supporting Information [file CTM2-16-e70732-s001.zip › LocusZoom/Sfig_rs11697662_locusZoom.pdf]

# LocusZoom plots of GWAS top lead SNP

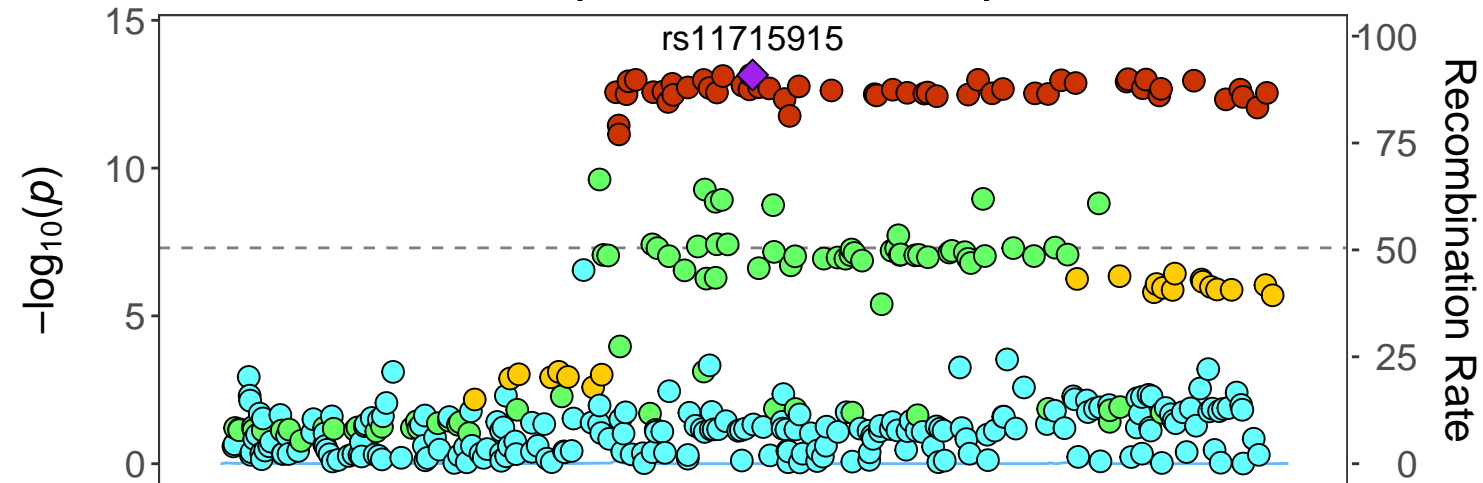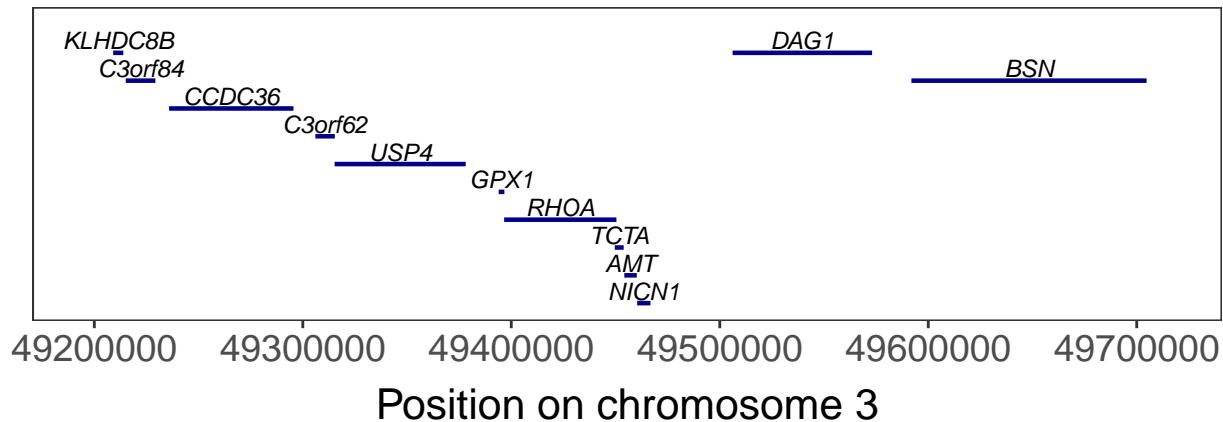

r2    miss    0.0–0.2    0.2–0.4    0.4–0.6    0.6–0.8    0.8–1.0

Supplement: Supplementary file 5 — Supporting Information [file CTM2-16-e70732-s001.zip › LocusZoom/Sfig_rs11715915_locusZoom.pdf]

# LocusZoom plots of GWAS top lead SNP

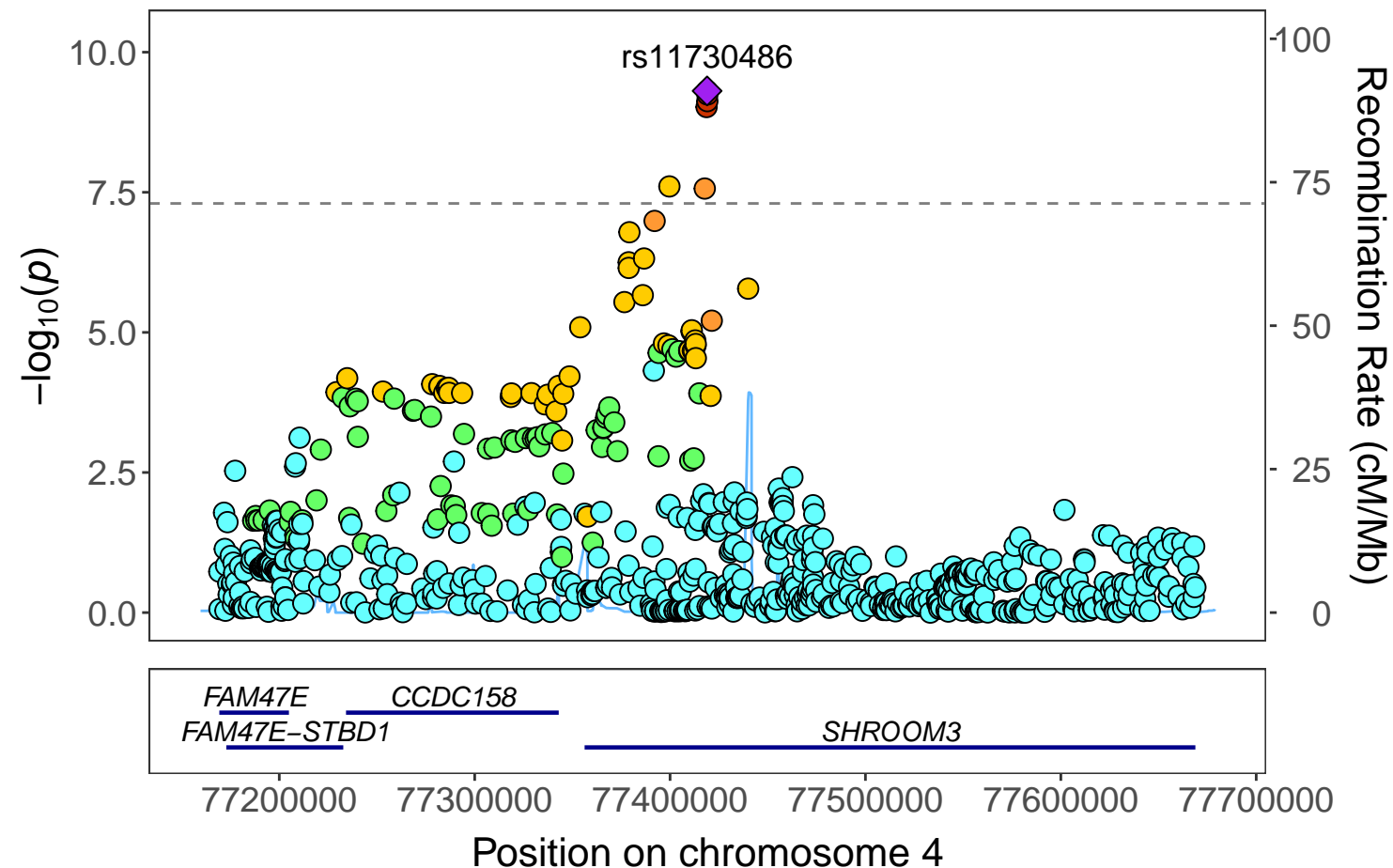

$r^2$  ○ miss ○ 0.0–0.2 ○ 0.2–0.4 ○ 0.4–0.6 ○ 0.6–0.8 ○ 0.8–1.0

Supplement: Supplementary file 5 — Supporting Information [file CTM2-16-e70732-s001.zip › LocusZoom/Sfig_rs11730486_locusZoom.pdf]

# *LocusZoom plots of GWAS top lead SNP*

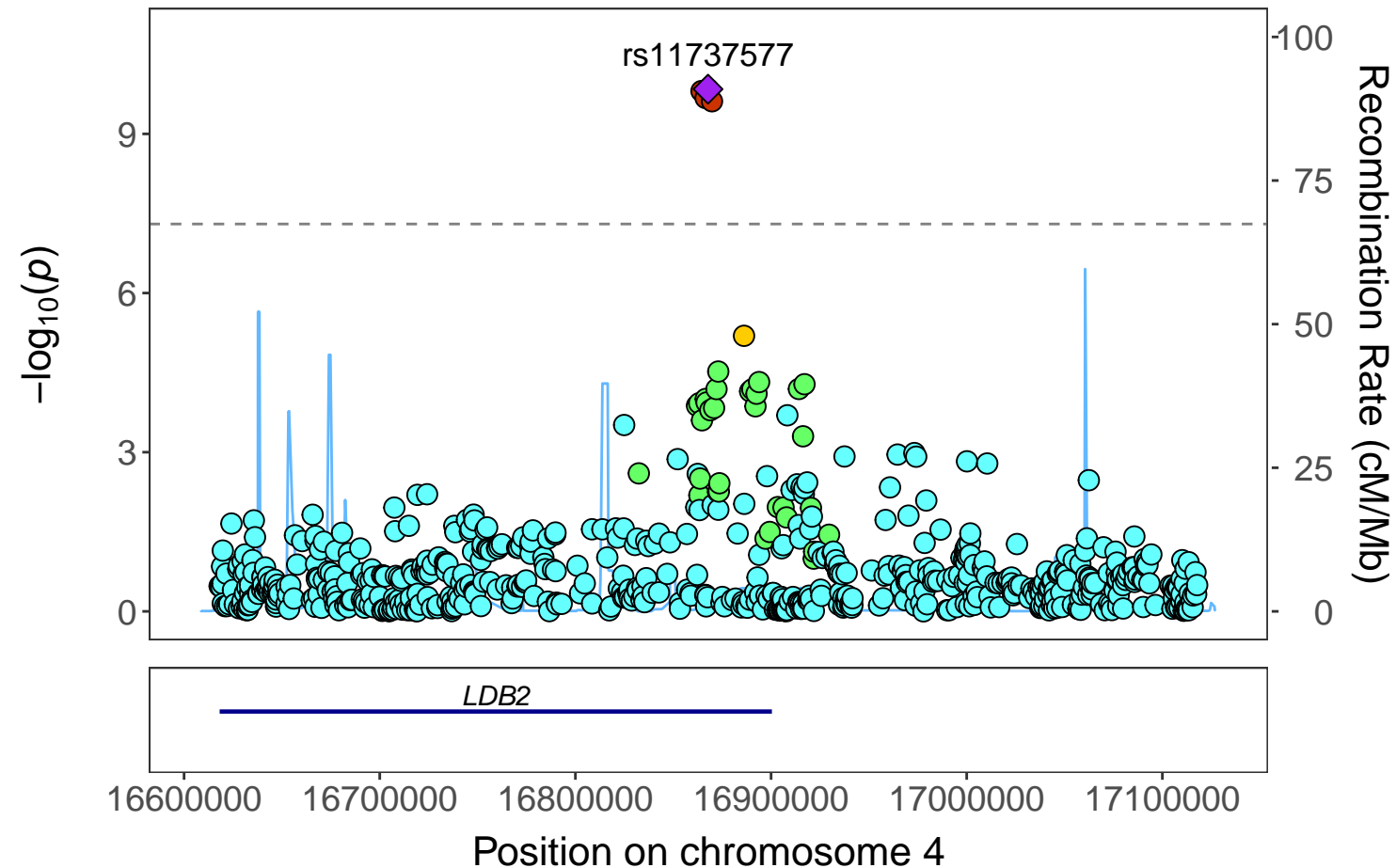

r2    miss    0.0–0.2    0.2–0.4    0.4–0.6    0.6–0.8    0.8–1.0

Supplement: Supplementary file 5 — Supporting Information [file CTM2-16-e70732-s001.zip › LocusZoom/Sfig_rs11737577_locusZoom.pdf]

# LocusZoom plots of GWAS top lead SNP

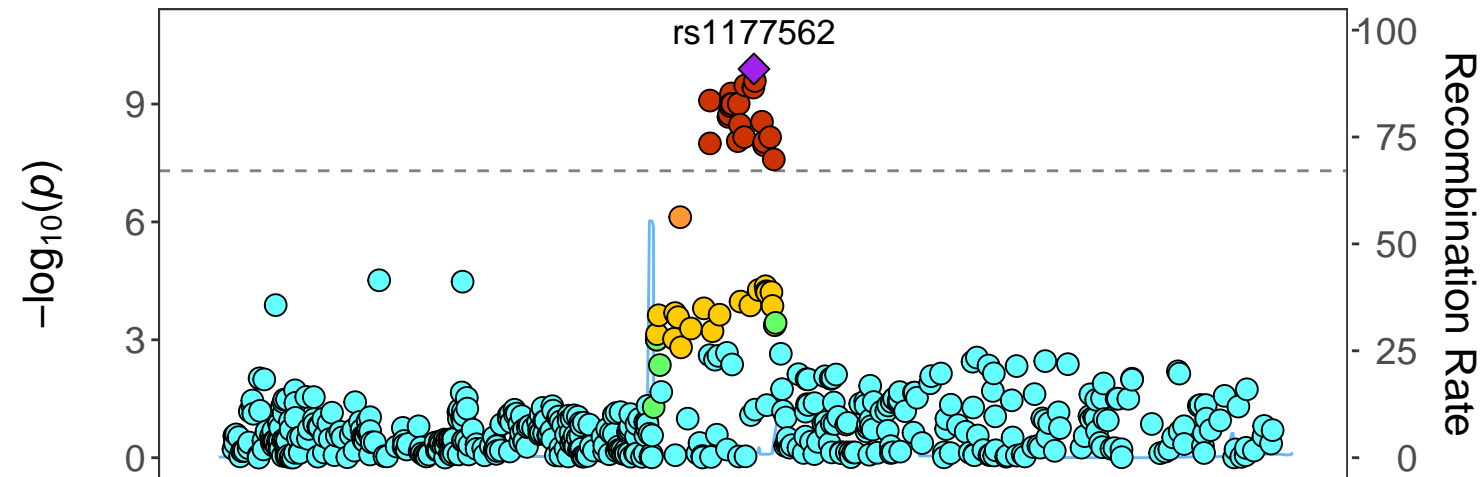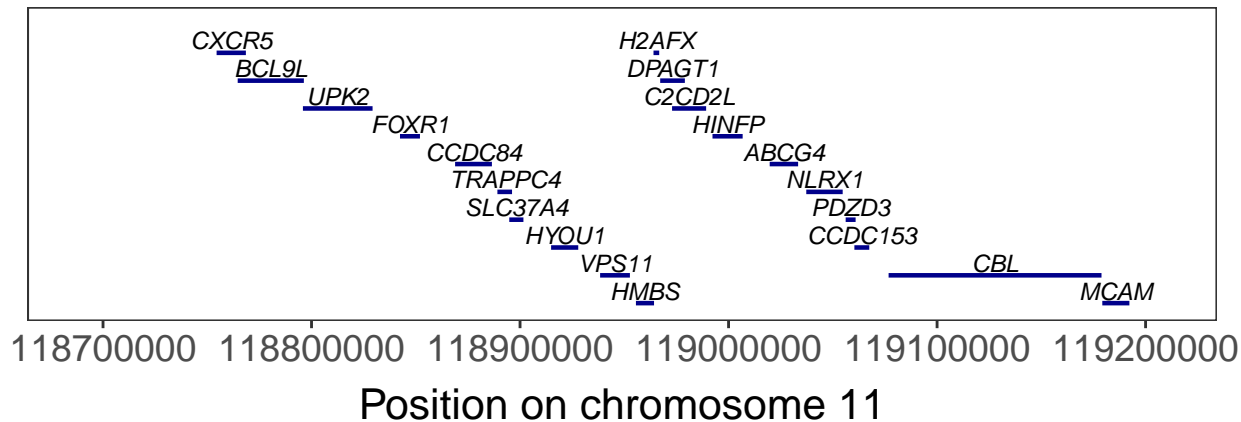

r2    miss    0.0–0.2    0.2–0.4    0.4–0.6    0.6–0.8    0.8–1.0

Supplement: Supplementary file 5 — Supporting Information [file CTM2-16-e70732-s001.zip › LocusZoom/Sfig_rs1177562_locusZoom.pdf]

# *LocusZoom plots of GWAS top lead SNP*

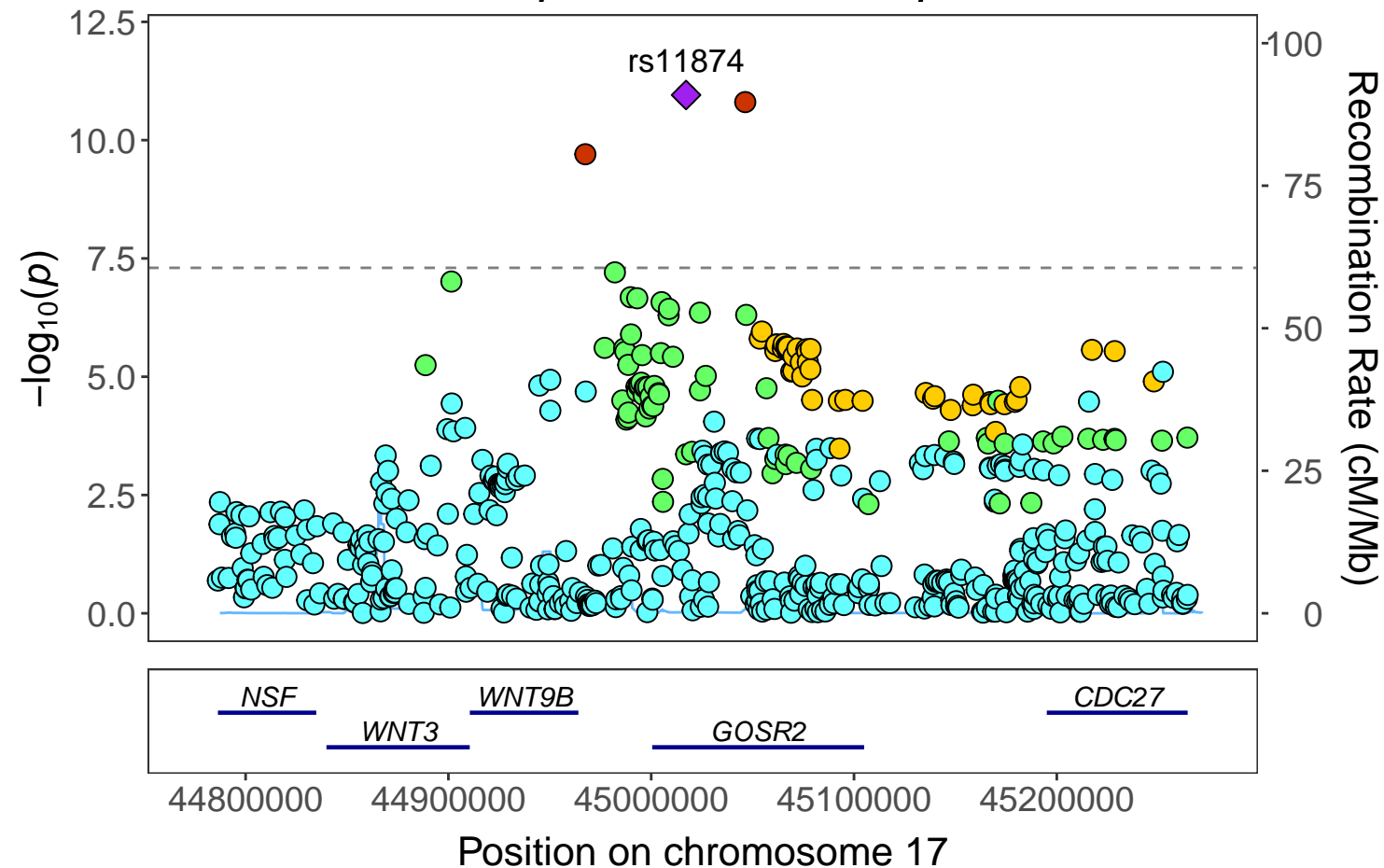

r2    miss    0.0-0.2    0.2-0.4    0.4-0.6    0.6-0.8    0.8-1.0

Supplement: Supplementary file 5 — Supporting Information [file CTM2-16-e70732-s001.zip › LocusZoom/Sfig_rs11874_locusZoom.pdf]

# LocusZoom plots of GWAS top lead SNP

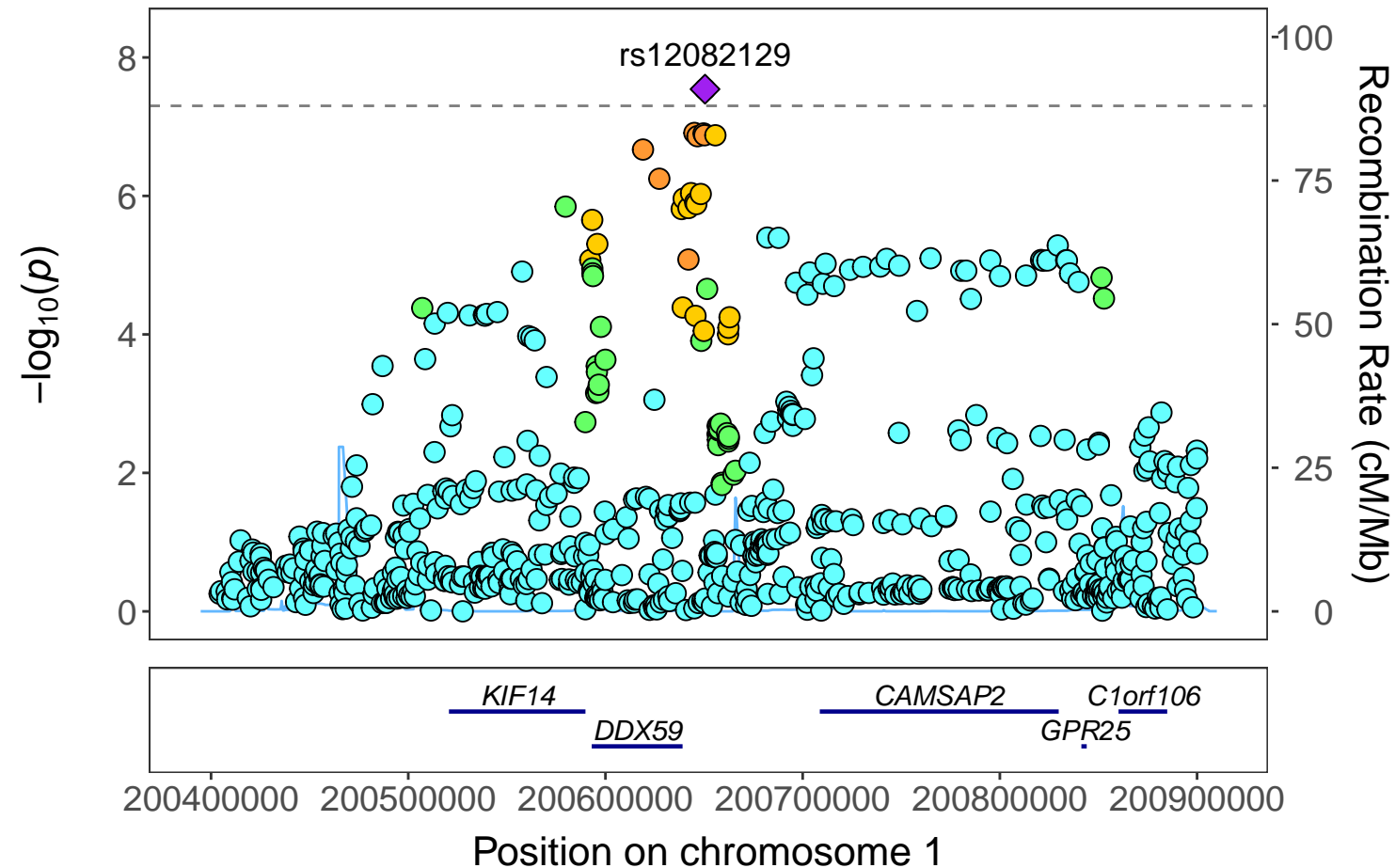

$r^2$  ○ miss ○ 0.0–0.2 ○ 0.2–0.4 ○ 0.4–0.6 ○ 0.6–0.8 ○ 0.8–1.0

Supplement: Supplementary file 5 — Supporting Information [file CTM2-16-e70732-s001.zip › LocusZoom/Sfig_rs12082129_locusZoom.pdf]

# LocusZoom plots of GWAS top lead SNP

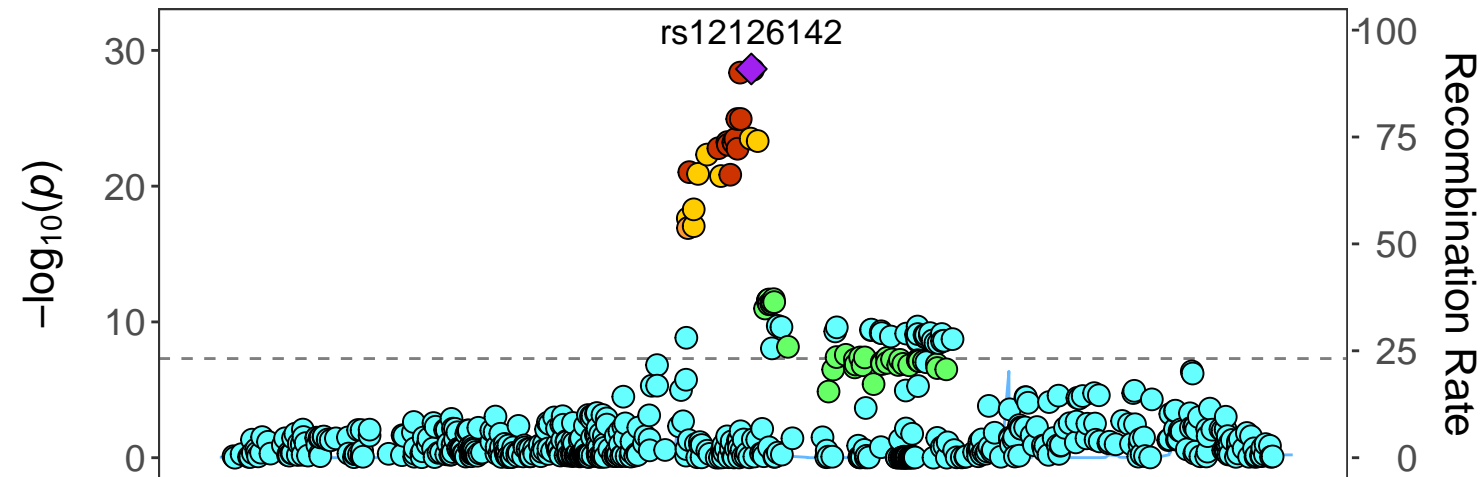

r2 ○ miss ○ 0.0–0.2 ○ 0.2–0.4 ○ 0.4–0.6 ○ 0.6–0.8 ○ 0.8–1.0

Supplement: Supplementary file 5 — Supporting Information [file CTM2-16-e70732-s001.zip › LocusZoom/Sfig_rs12126142_locusZoom.pdf]

# LocusZoom plots of GWAS top lead SNP

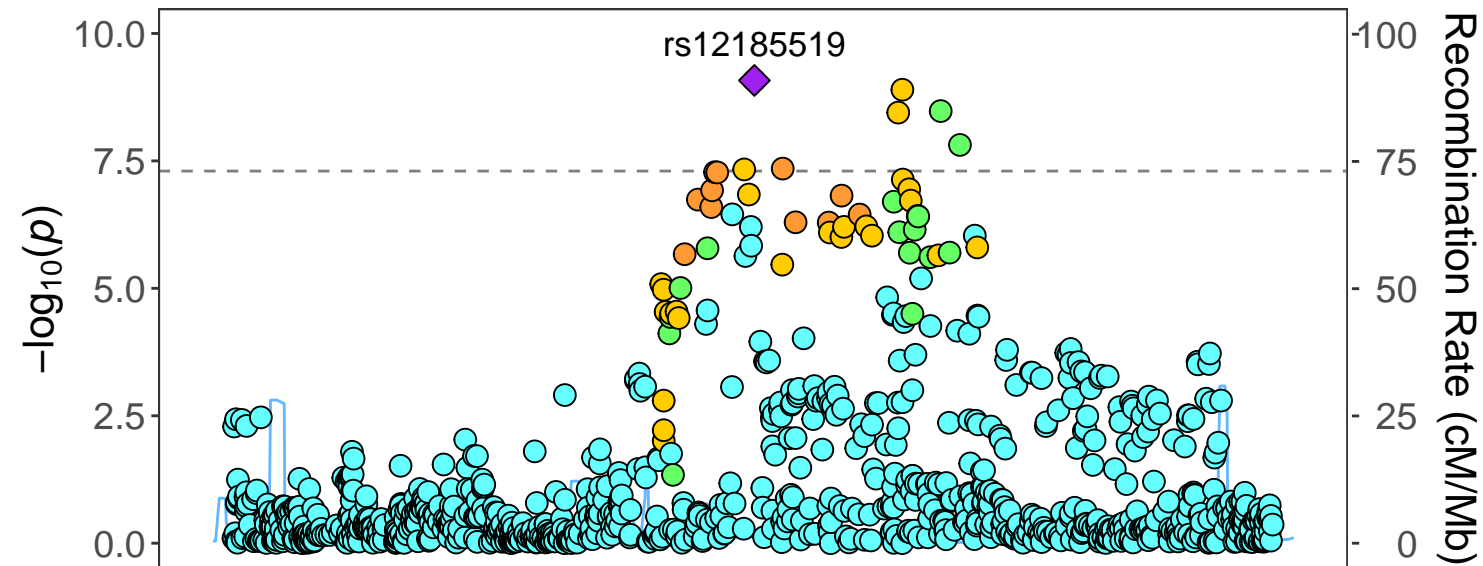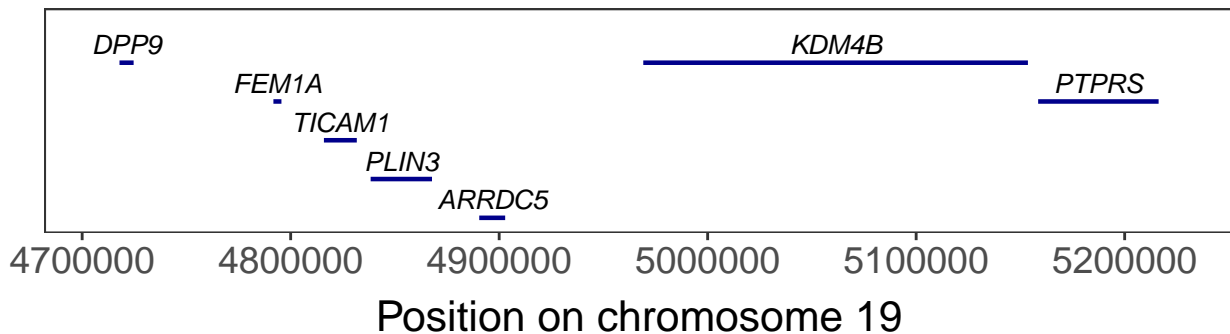

r2    miss    0.0–0.2    0.2–0.4    0.4–0.6    0.6–0.8    0.8–1.0

Supplement: Supplementary file 5 — Supporting Information [file CTM2-16-e70732-s001.zip › LocusZoom/Sfig_rs12185519_locusZoom.pdf]

# LocusZoom plots of GWAS top lead SNP

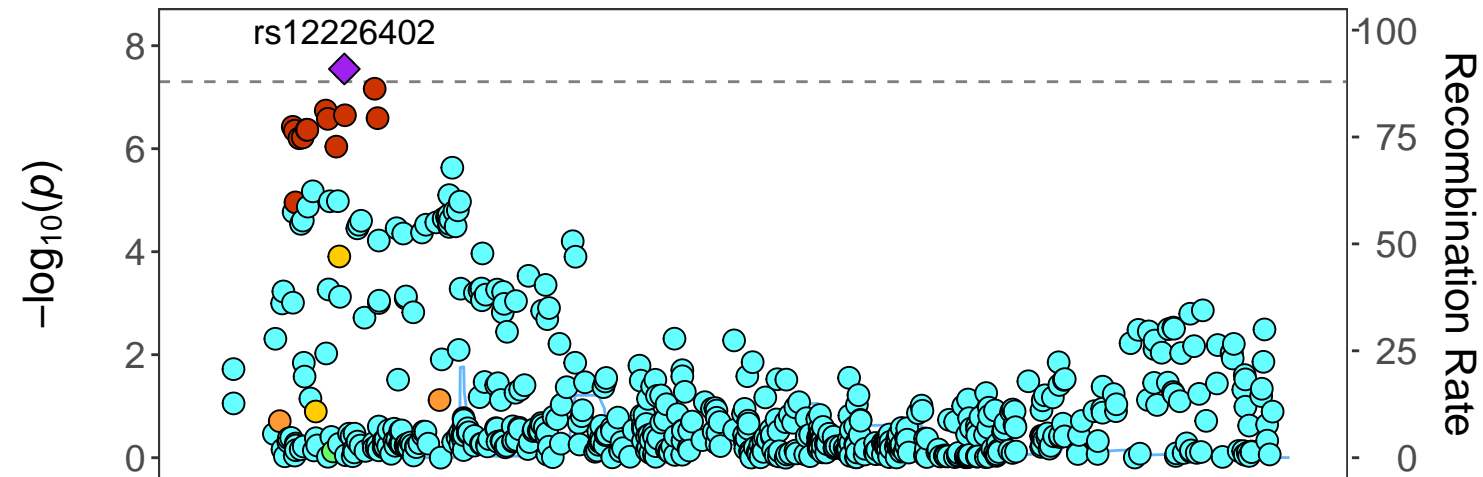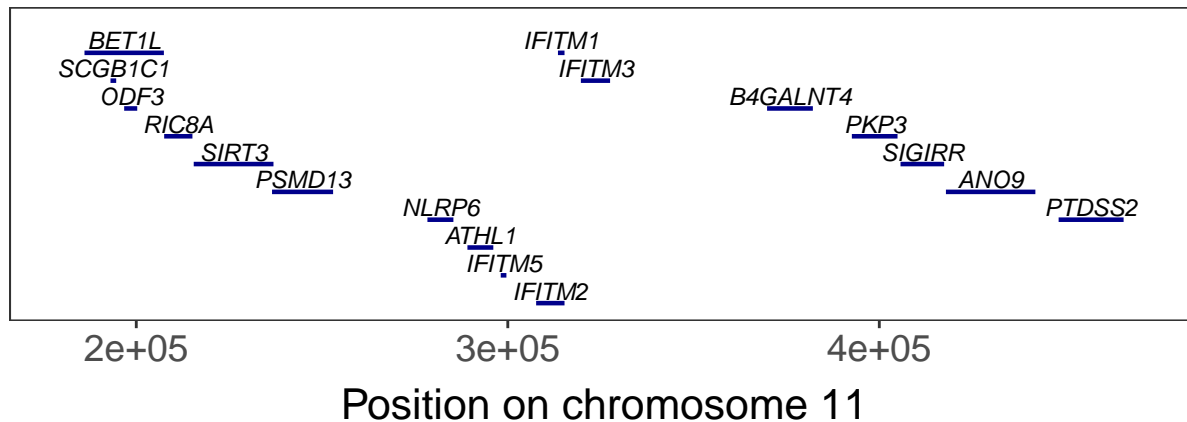

$r^2$     $\circ$  miss    $\circ$  0.0–0.2    $\circ$  0.2–0.4    $\circ$  0.4–0.6    $\circ$  0.6–0.8    $\circ$  0.8–1.0

Supplement: Supplementary file 5 — Supporting Information [file CTM2-16-e70732-s001.zip › LocusZoom/Sfig_rs12226402_locusZoom.pdf]

# LocusZoom plots of GWAS top lead SNP

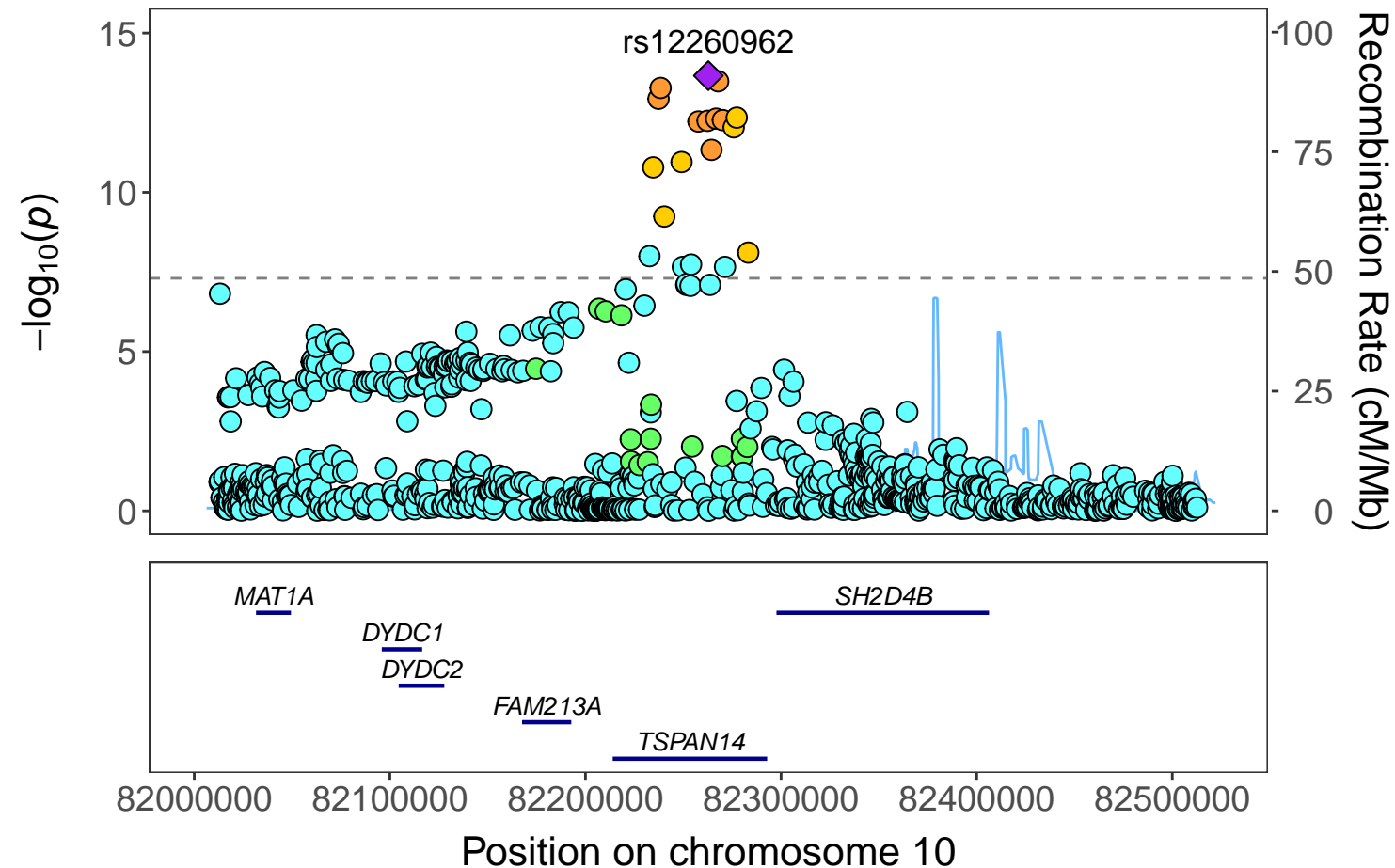

$r^2$     $\circ$  miss    $\circ$  0.0–0.2    $\circ$  0.2–0.4    $\circ$  0.4–0.6    $\circ$  0.6–0.8    $\circ$  0.8–1.0

Supplement: Supplementary file 5 — Supporting Information [file CTM2-16-e70732-s001.zip › LocusZoom/Sfig_rs12260962_locusZoom.pdf]

# LocusZoom plots of GWAS top lead SNP

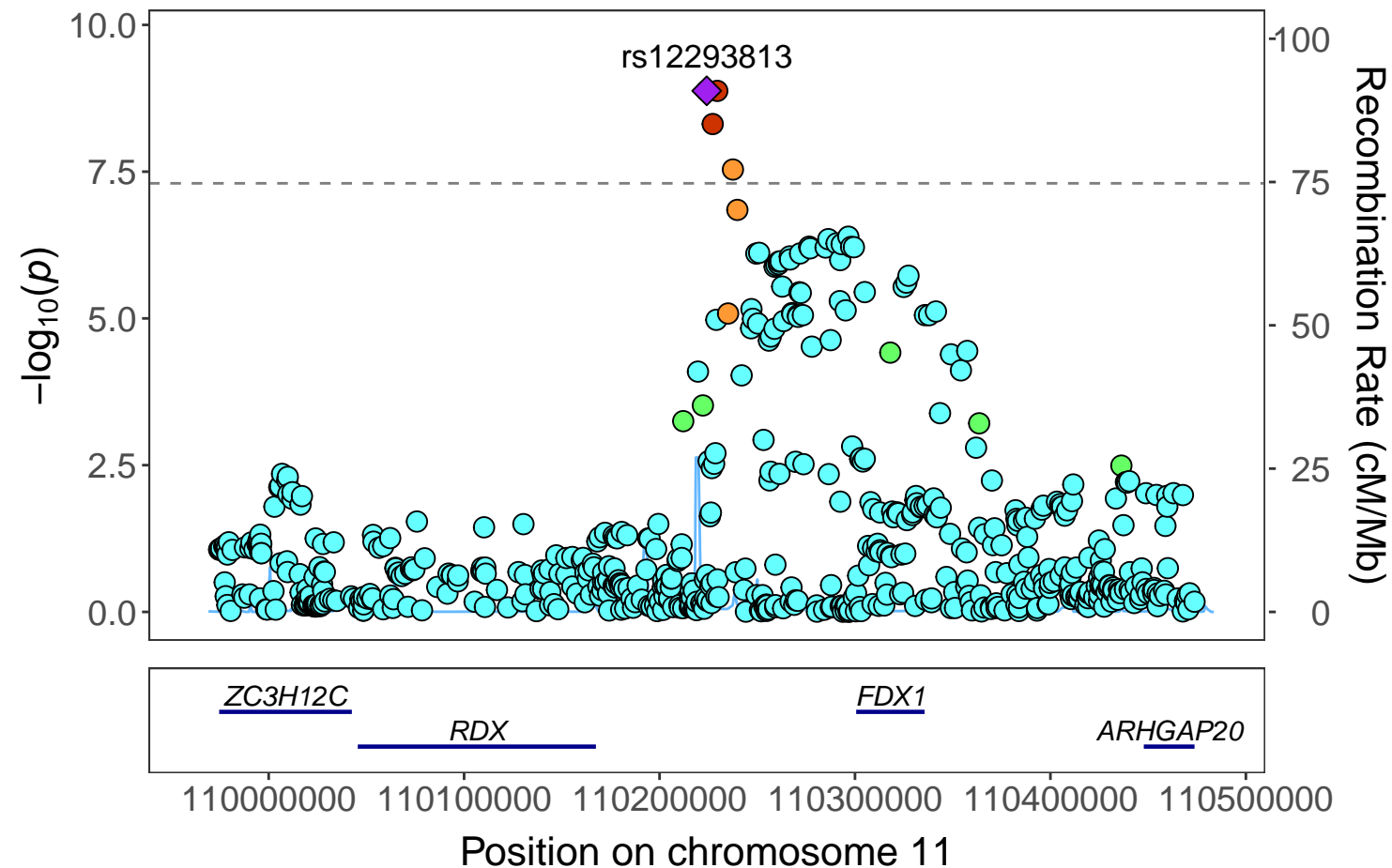

r2   miss   0.0–0.2   0.2–0.4   0.4–0.6   0.6–0.8   0.8–1.0

Supplement: Supplementary file 5 — Supporting Information [file CTM2-16-e70732-s001.zip › LocusZoom/Sfig_rs12293813_locusZoom.pdf]

# LocusZoom plots of GWAS top lead SNP

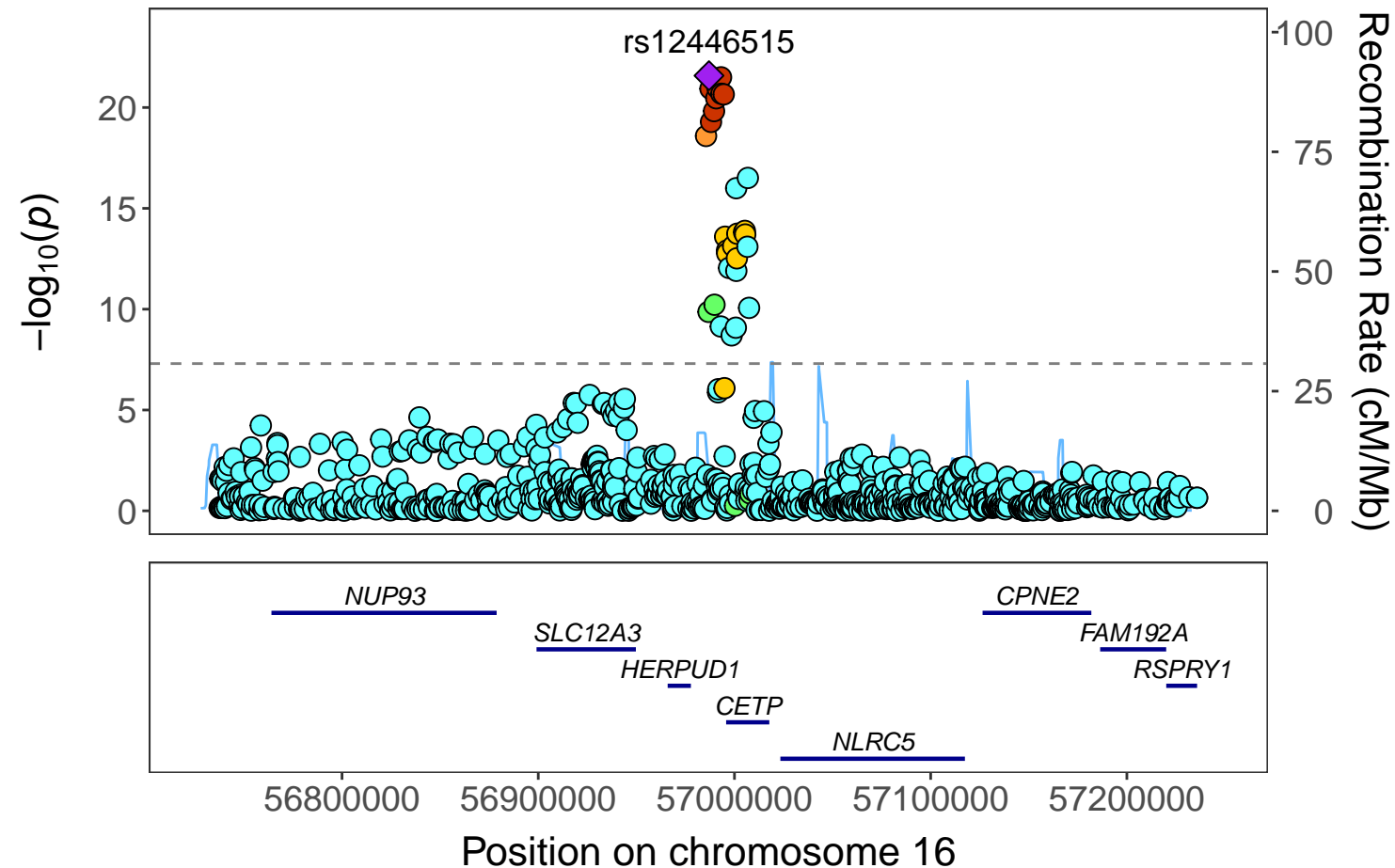

$r^2$    miss   0.0-0.2   0.2-0.4   0.4-0.6   0.6-0.8   0.8-1.0

Supplement: Supplementary file 5 — Supporting Information [file CTM2-16-e70732-s001.zip › LocusZoom/Sfig_rs12446515_locusZoom.pdf]

# LocusZoom plots of GWAS top lead SNP

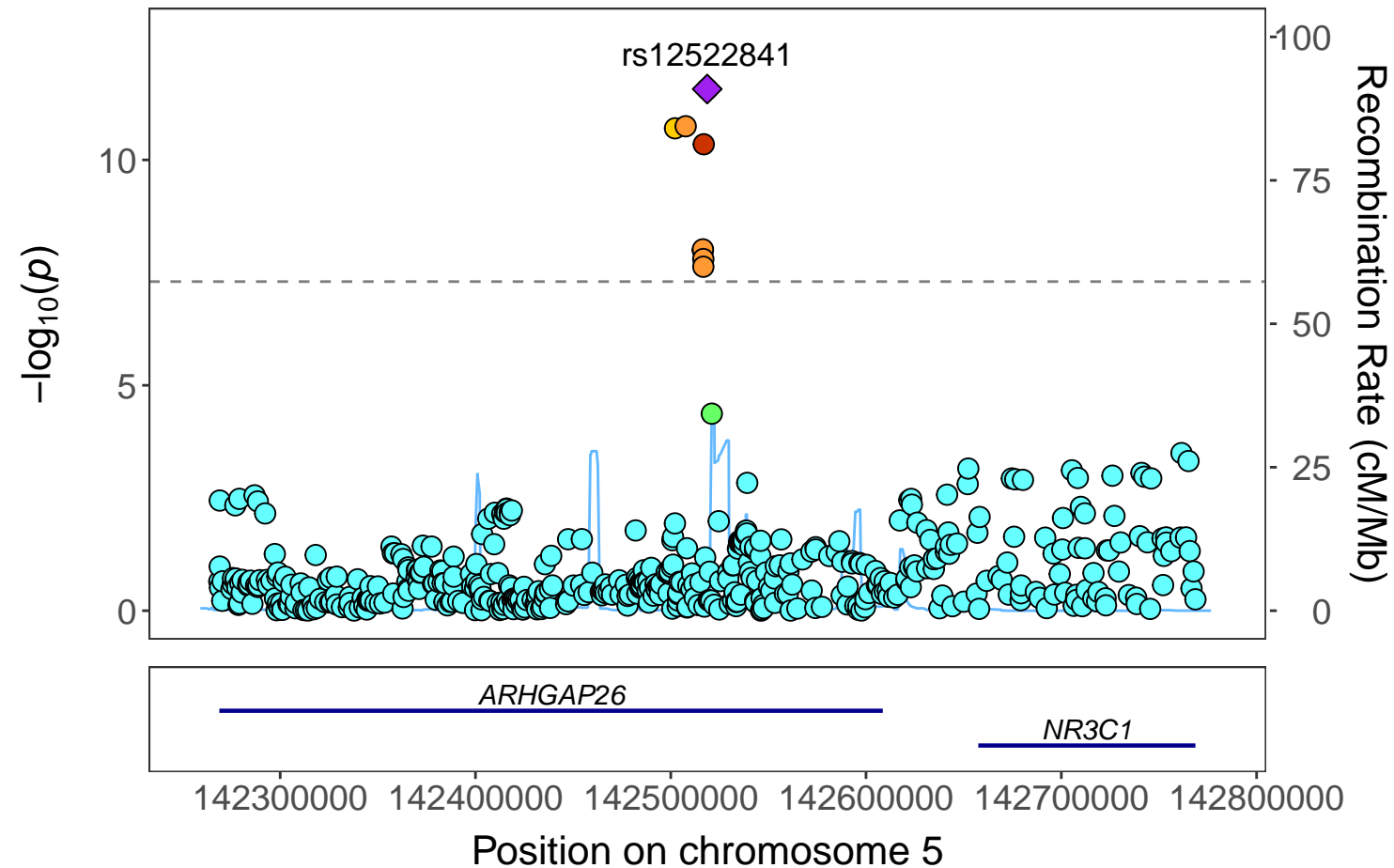

$r^2$    miss   0.0-0.2   0.2-0.4   0.4-0.6   0.6-0.8   0.8-1.0

Supplement: Supplementary file 5 — Supporting Information [file CTM2-16-e70732-s001.zip › LocusZoom/Sfig_rs12522841_locusZoom.pdf]

# *LocusZoom plots of GWAS top lead SNP*

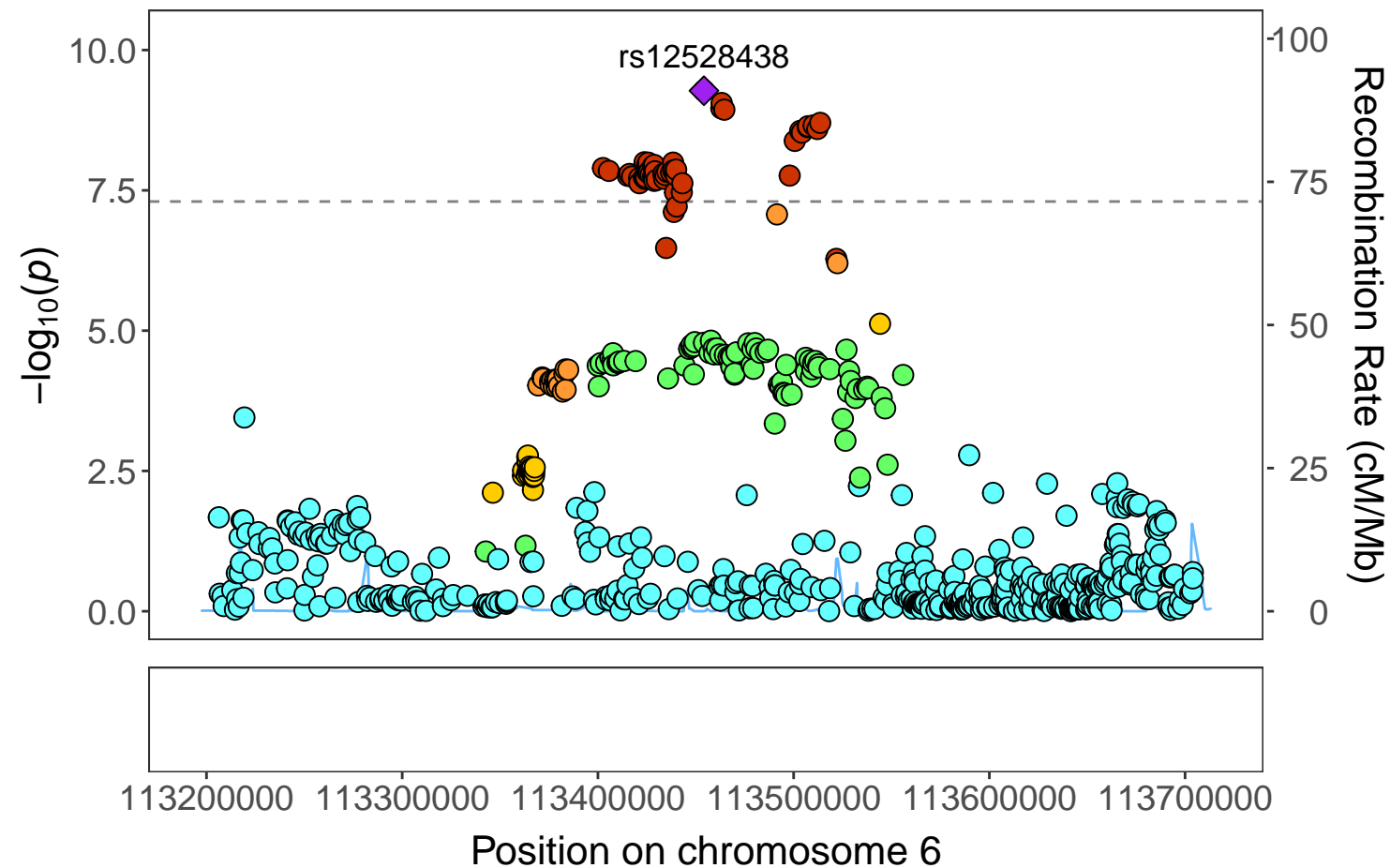

$r^2$    miss   cyan   0.0-0.2   green   0.2-0.4   yellow   0.4-0.6   orange   0.6-0.8   dark red   0.8-1.0

Supplement: Supplementary file 5 — Supporting Information [file CTM2-16-e70732-s001.zip › LocusZoom/Sfig_rs12528438_locusZoom.pdf]

# LocusZoom plots of GWAS top lead SNP

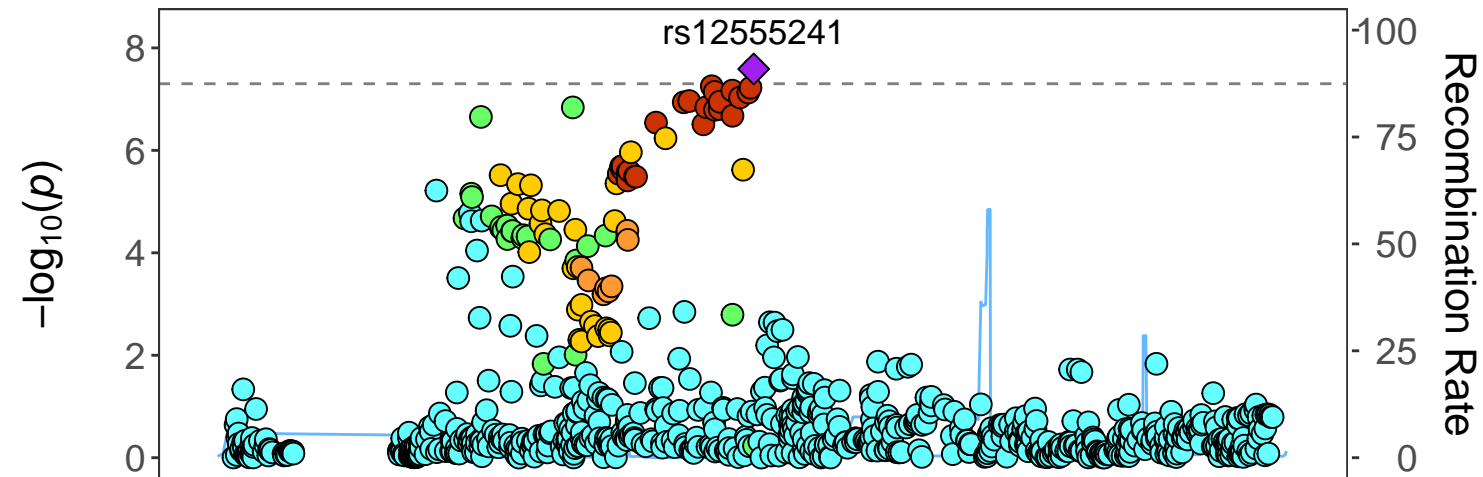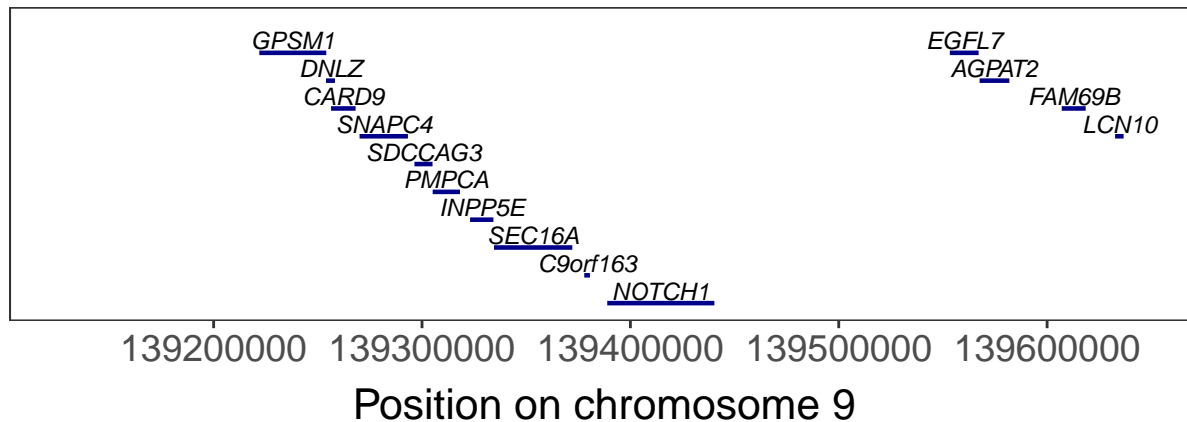

r2    miss    0.0–0.2    0.2–0.4    0.4–0.6    0.6–0.8    0.8–1.0

Supplement: Supplementary file 5 — Supporting Information [file CTM2-16-e70732-s001.zip › LocusZoom/Sfig_rs12555241_locusZoom.pdf]

# LocusZoom plots of GWAS top lead SNP

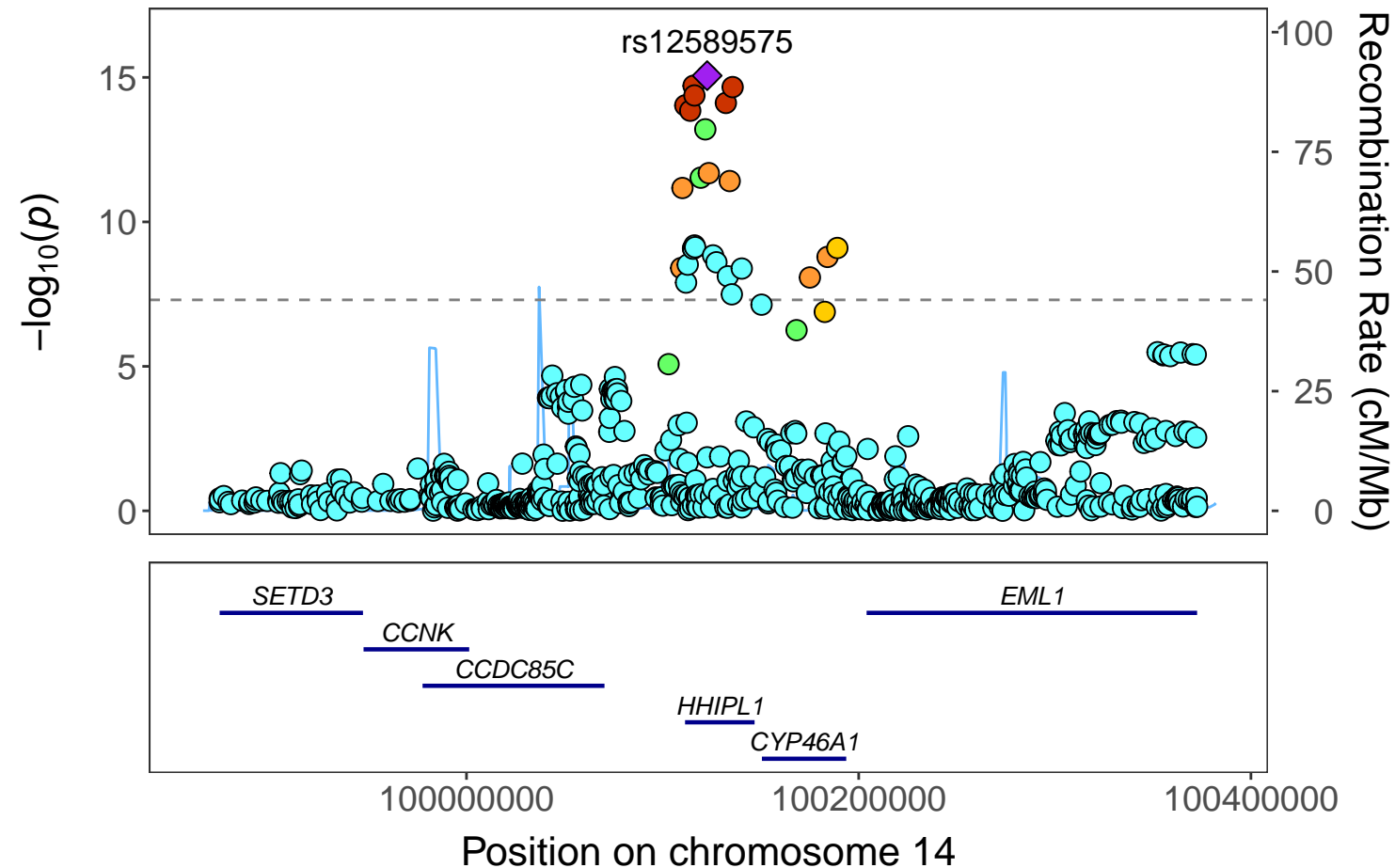

r2   miss   0.0–0.2   0.2–0.4   0.4–0.6   0.6–0.8   0.8–1.0

Supplement: Supplementary file 5 — Supporting Information [file CTM2-16-e70732-s001.zip › LocusZoom/Sfig_rs12589575_locusZoom.pdf]

# LocusZoom plots of GWAS top lead SNP

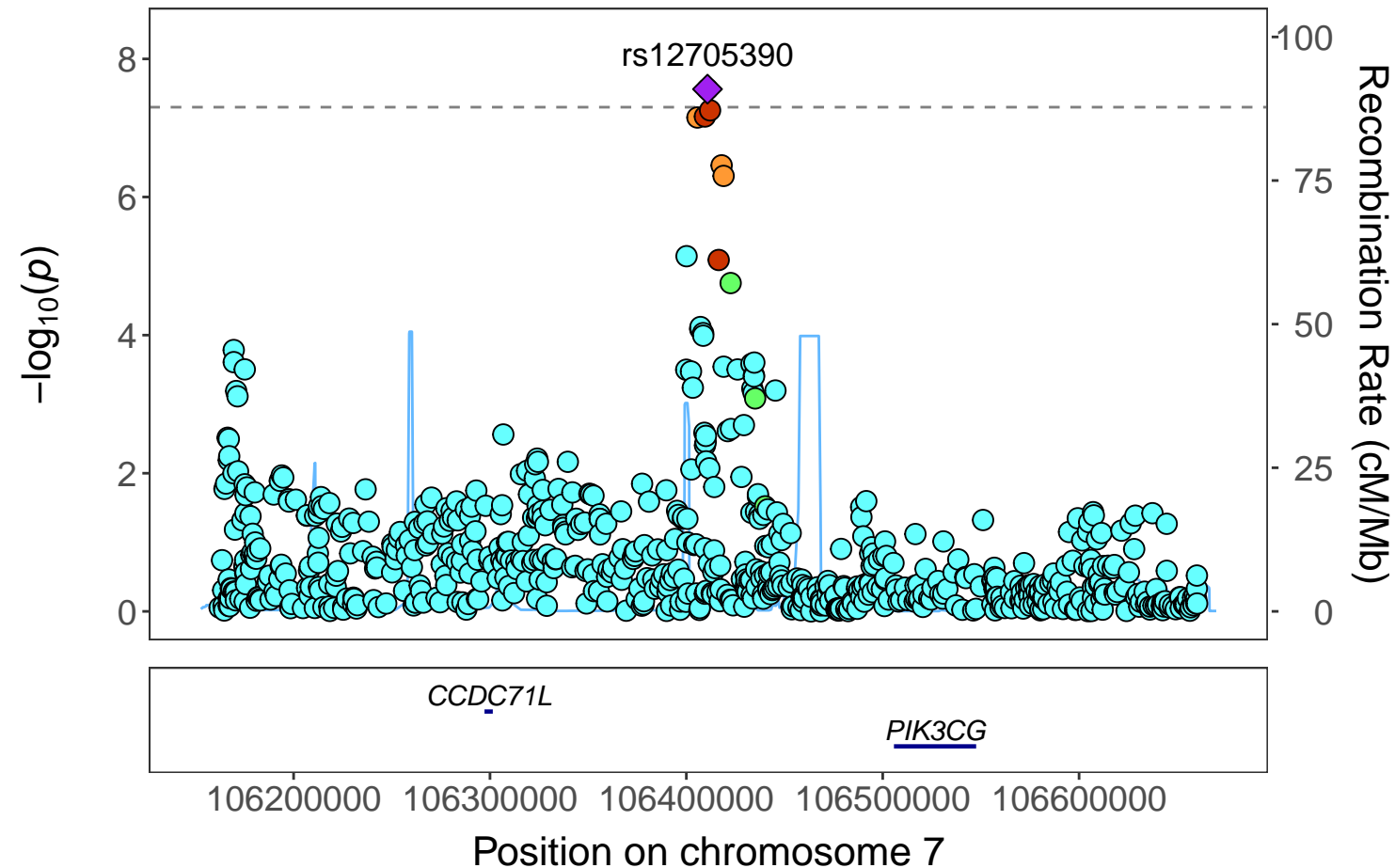

$r^2$    miss   0.0–0.2   0.2–0.4   0.4–0.6   0.6–0.8   0.8–1.0

Supplement: Supplementary file 5 — Supporting Information [file CTM2-16-e70732-s001.zip › LocusZoom/Sfig_rs12705390_locusZoom.pdf]

# LocusZoom plots of GWAS top lead SNP

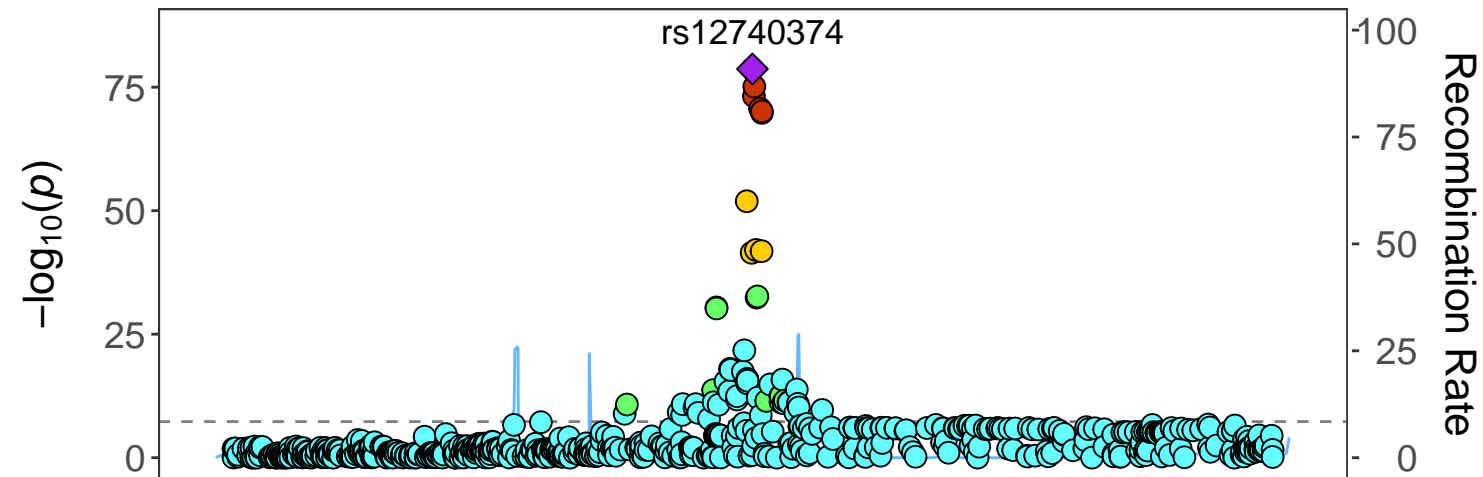

r2   miss   0.0–0.2   0.2–0.4   0.4–0.6   0.6–0.8   0.8–1.0

Supplement: Supplementary file 5 — Supporting Information [file CTM2-16-e70732-s001.zip › LocusZoom/Sfig_rs12740374_locusZoom.pdf]

# LocusZoom plots of GWAS top lead SNP

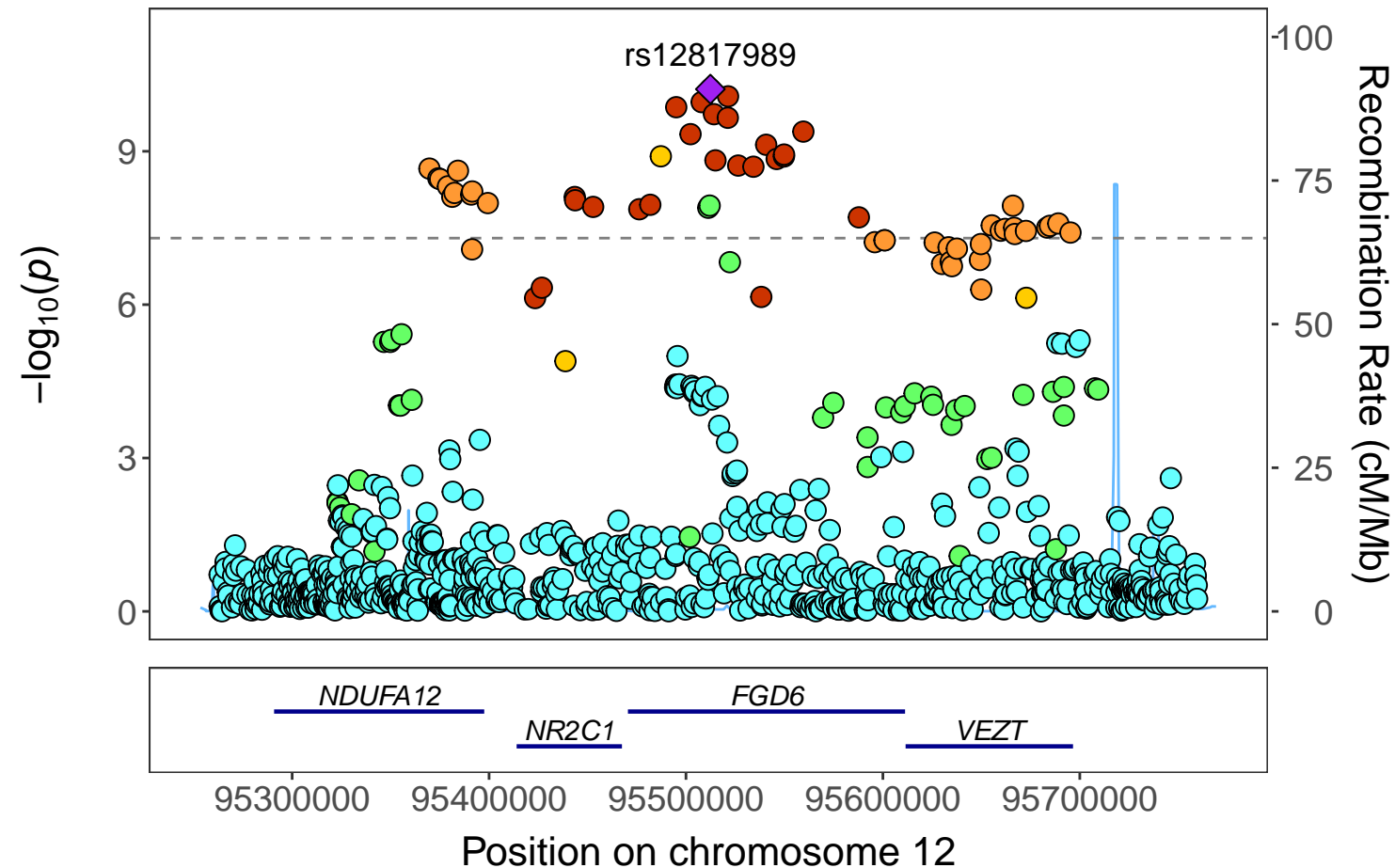

$r^2$    miss   0.0–0.2   0.2–0.4   0.4–0.6   0.6–0.8   0.8–1.0

Supplement: Supplementary file 5 — Supporting Information [file CTM2-16-e70732-s001.zip › LocusZoom/Sfig_rs12817989_locusZoom.pdf]

# *LocusZoom plots of GWAS top lead SNP*

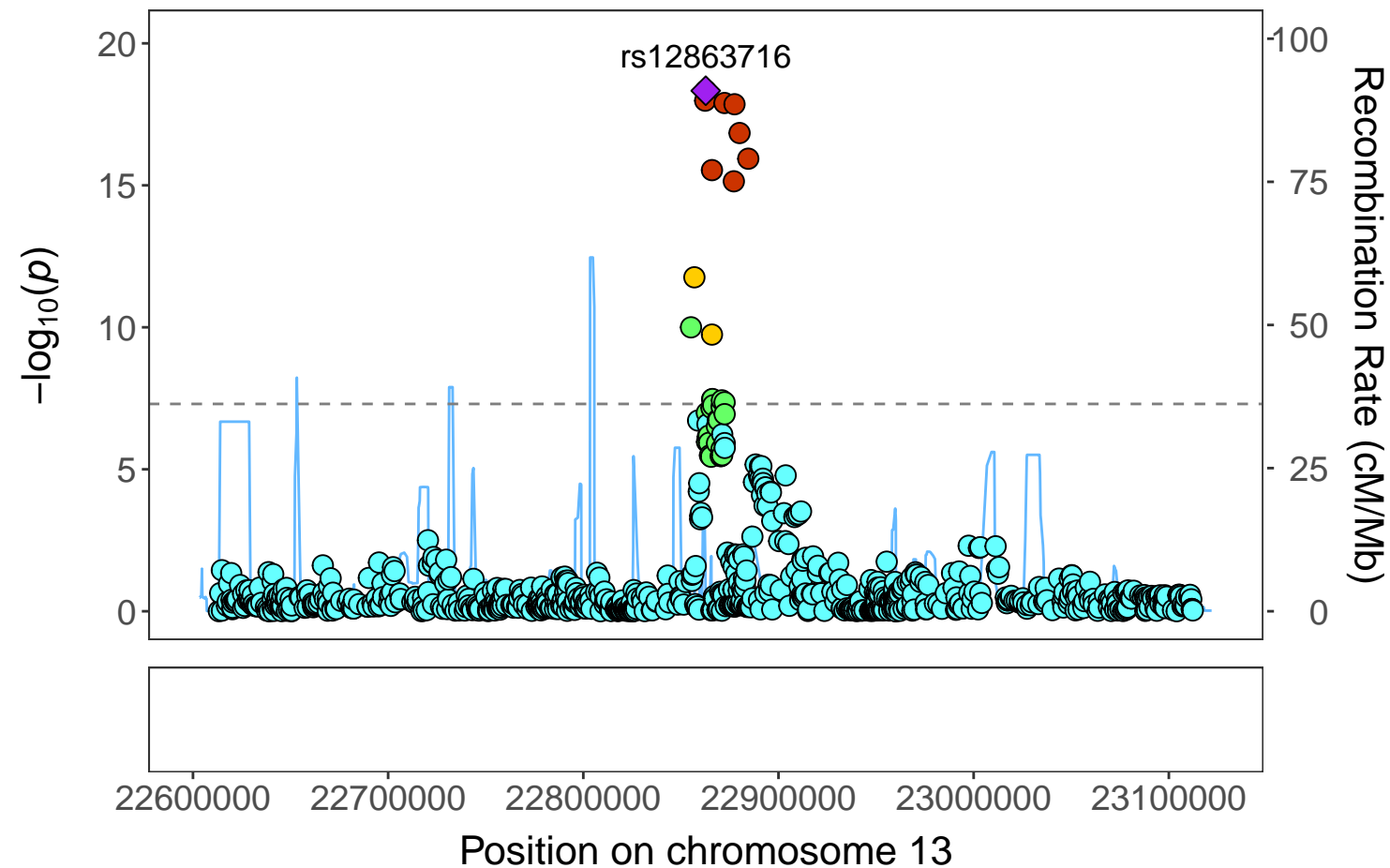

r2   miss   0.0–0.2   0.2–0.4   0.4–0.6   0.6–0.8   0.8–1.0

Supplement: Supplementary file 5 — Supporting Information [file CTM2-16-e70732-s001.zip › LocusZoom/Sfig_rs12863716_locusZoom.pdf]

# LocusZoom plots of GWAS top lead SNP

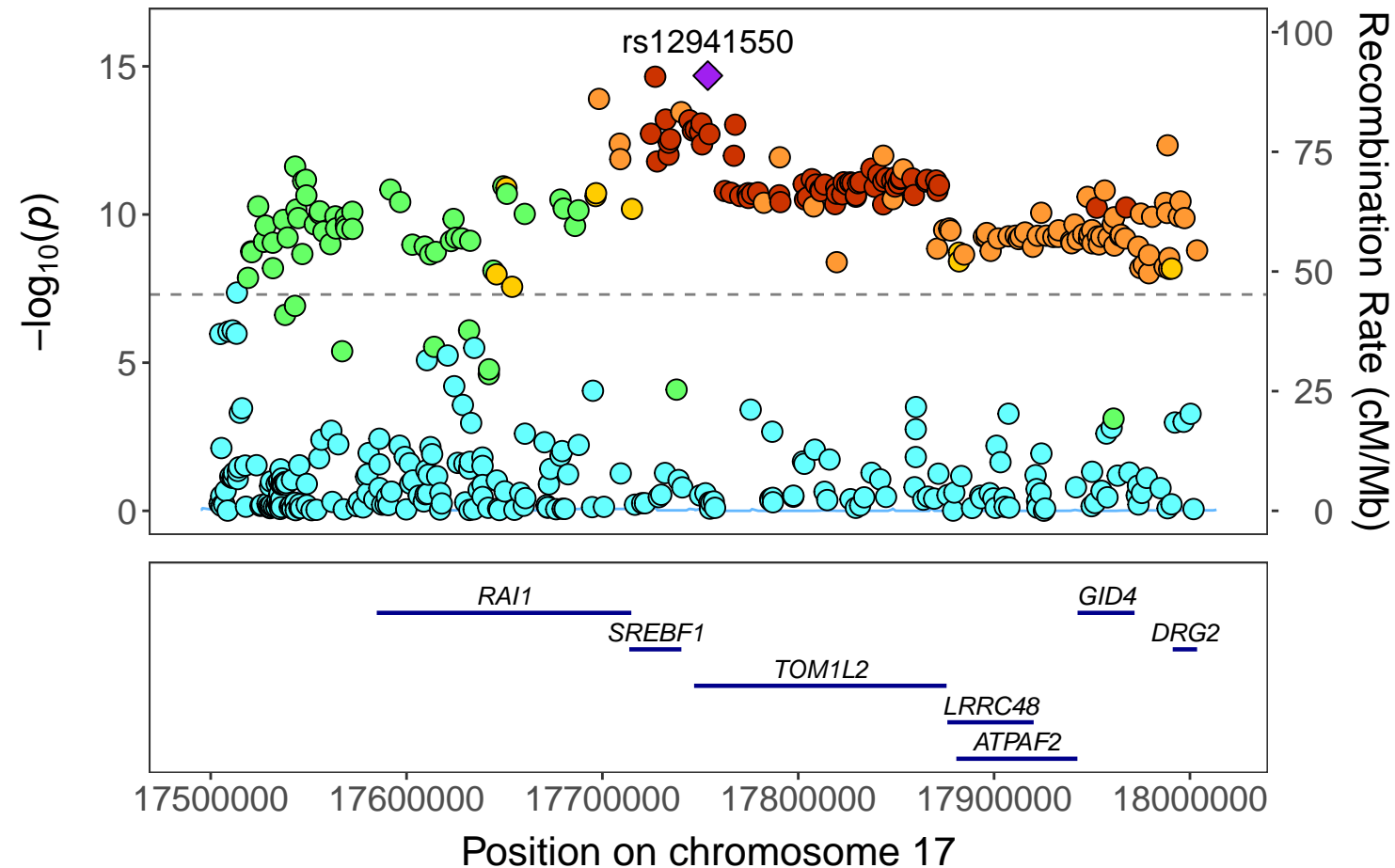

Supplement: Supplementary file 5 — Supporting Information [file CTM2-16-e70732-s001.zip › LocusZoom/Sfig_rs12941550_locusZoom.pdf]

# LocusZoom plots of GWAS top lead SNP

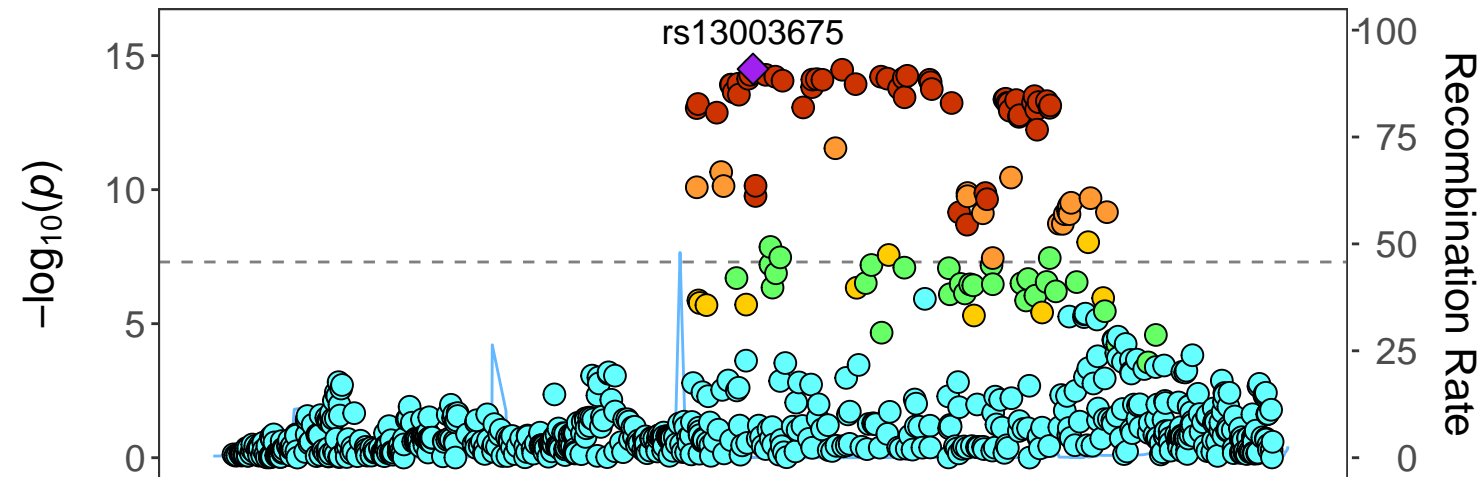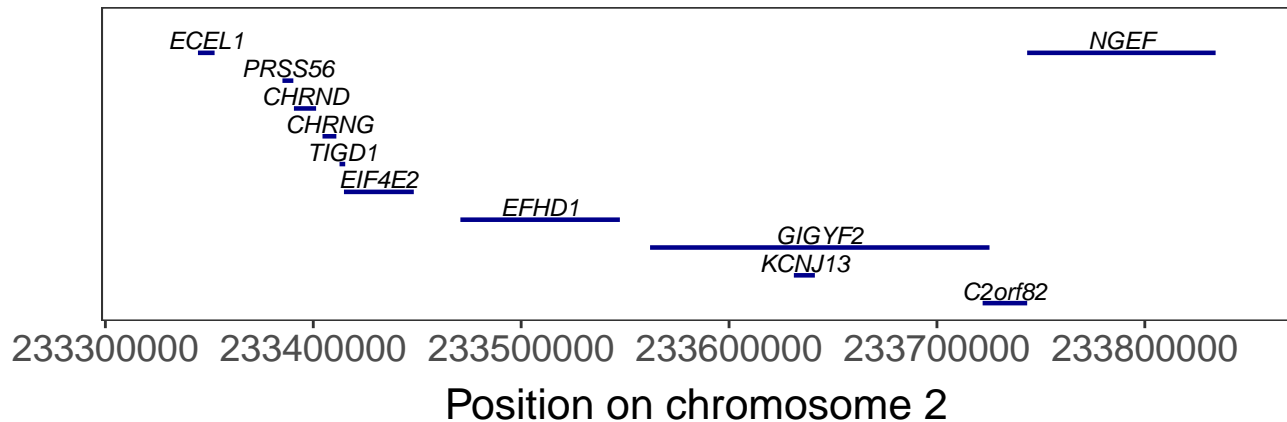

r2    miss    0.0-0.2    0.2-0.4    0.4-0.6    0.6-0.8    0.8-1.0

Supplement: Supplementary file 5 — Supporting Information [file CTM2-16-e70732-s001.zip › LocusZoom/Sfig_rs13003675_locusZoom.pdf]

# LocusZoom plots of GWAS top lead SNP

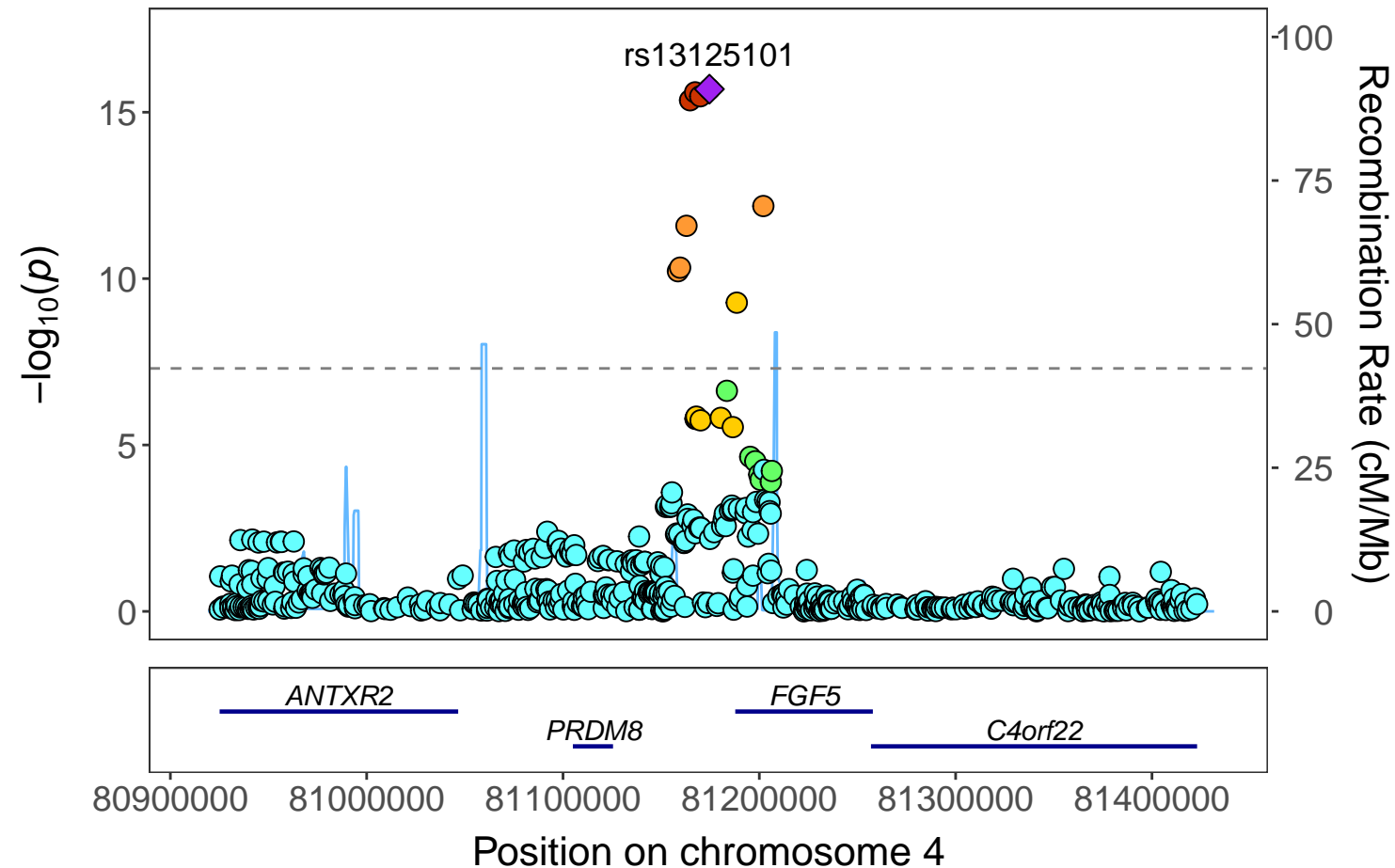

$r^2$     $\circ$  miss    $\circ$  0.0-0.2    $\circ$  0.2-0.4    $\circ$  0.4-0.6    $\circ$  0.6-0.8    $\circ$  0.8-1.0

Supplement: Supplementary file 5 — Supporting Information [file CTM2-16-e70732-s001.zip › LocusZoom/Sfig_rs13125101_locusZoom.pdf]

# LocusZoom plots of GWAS top lead SNP

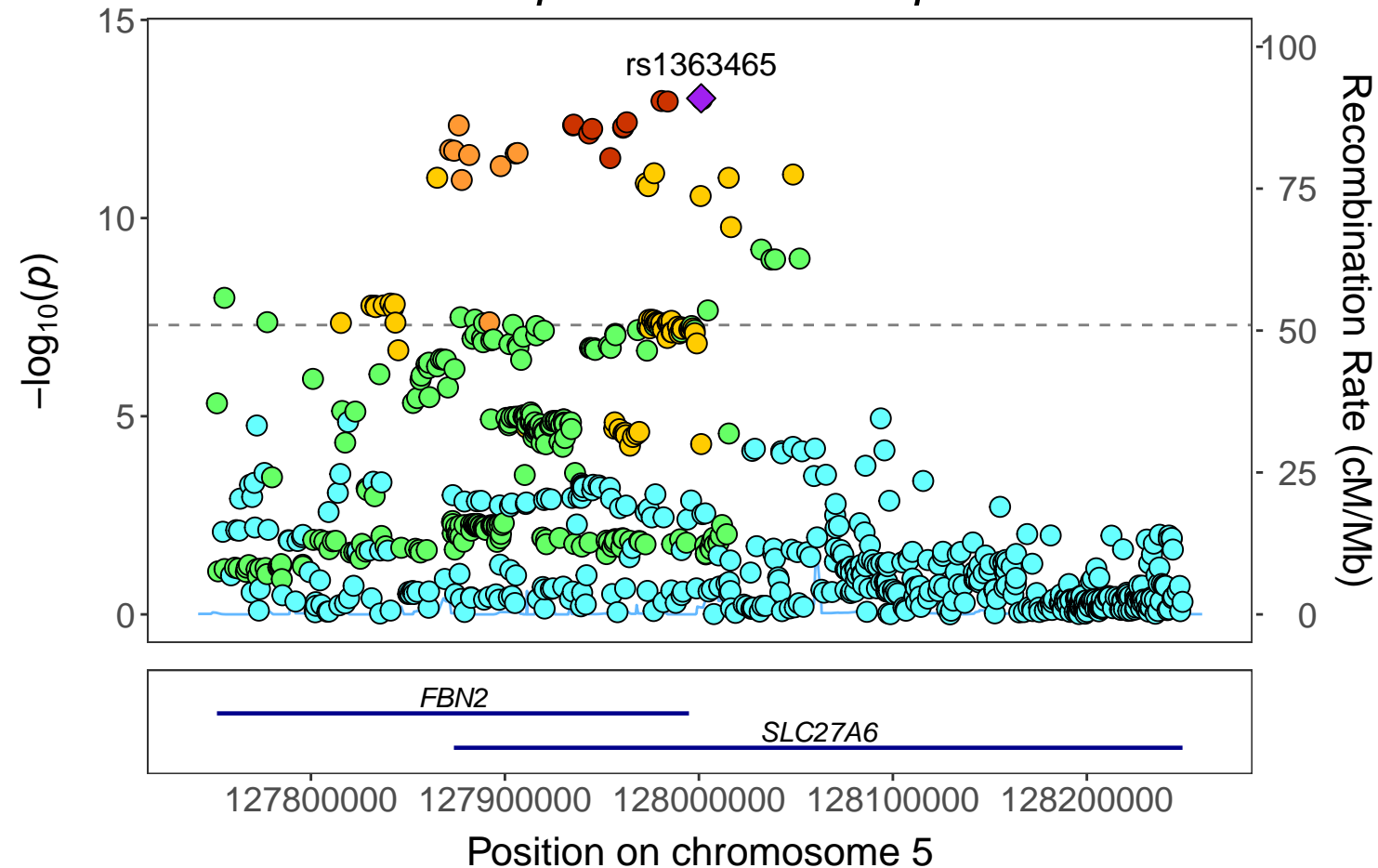

Supplement: Supplementary file 5 — Supporting Information [file CTM2-16-e70732-s001.zip › LocusZoom/Sfig_rs1363465_locusZoom.pdf]

# LocusZoom plots of GWAS top lead SNP

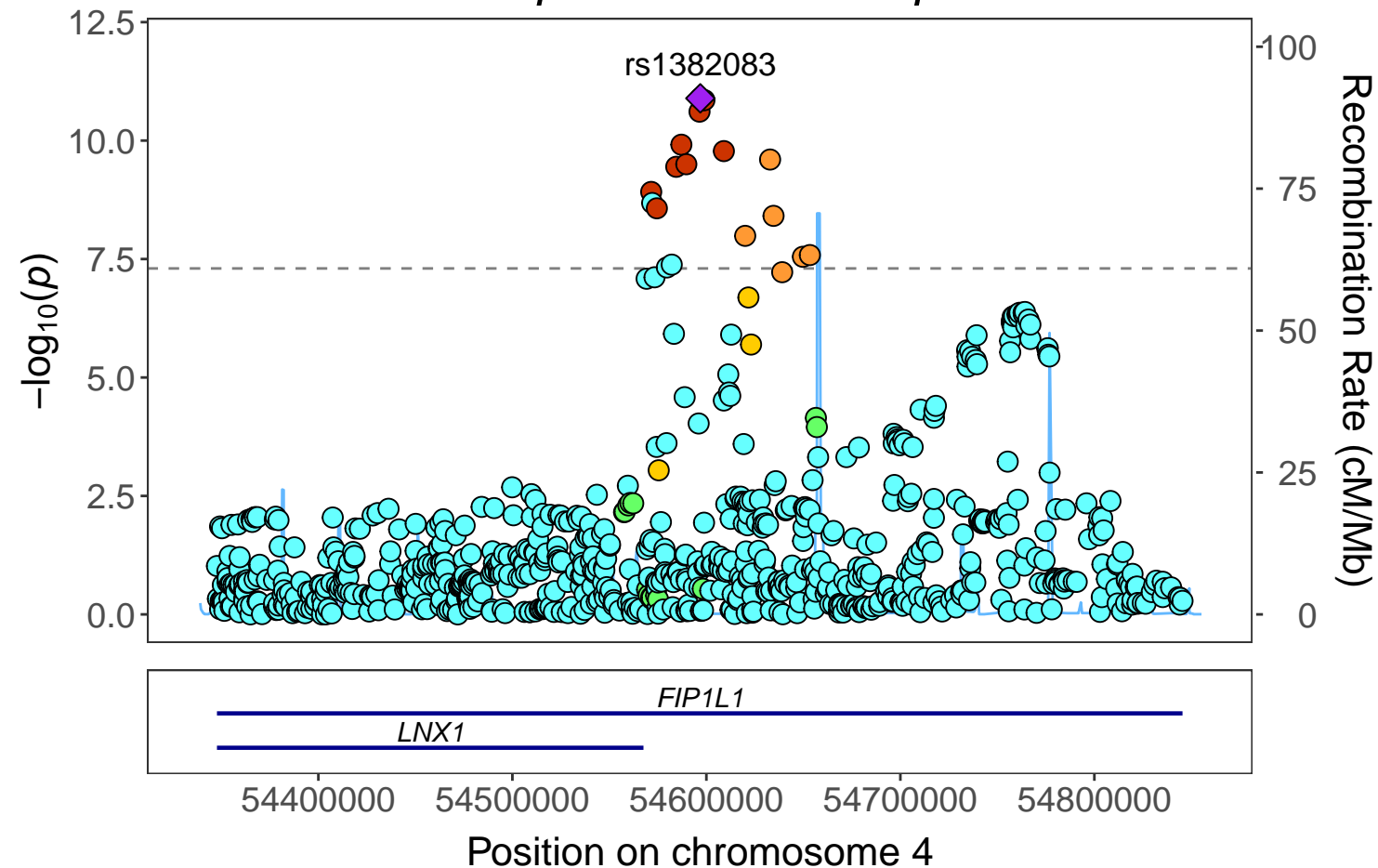

$r^2$    miss   0.0–0.2   0.2–0.4   0.4–0.6   0.6–0.8   0.8–1.0

Supplement: Supplementary file 5 — Supporting Information [file CTM2-16-e70732-s001.zip › LocusZoom/Sfig_rs1382083_locusZoom.pdf]

# LocusZoom plots of GWAS top lead SNP

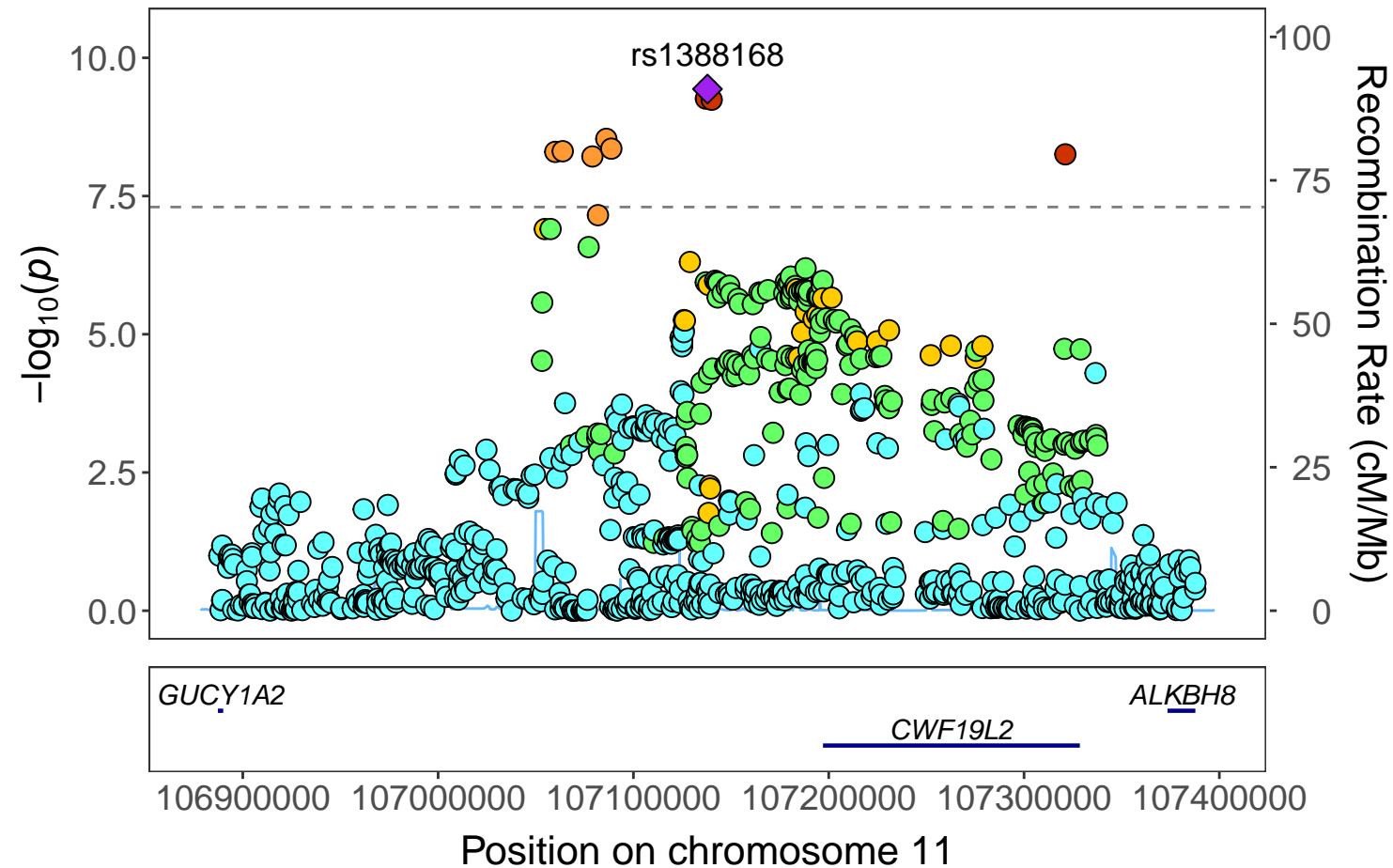

$r^2$    miss   0.0–0.2   0.2–0.4   0.4–0.6   0.6–0.8   0.8–1.0

Supplement: Supplementary file 5 — Supporting Information [file CTM2-16-e70732-s001.zip › LocusZoom/Sfig_rs1388168_locusZoom.pdf]

# LocusZoom plots of GWAS top lead SNP

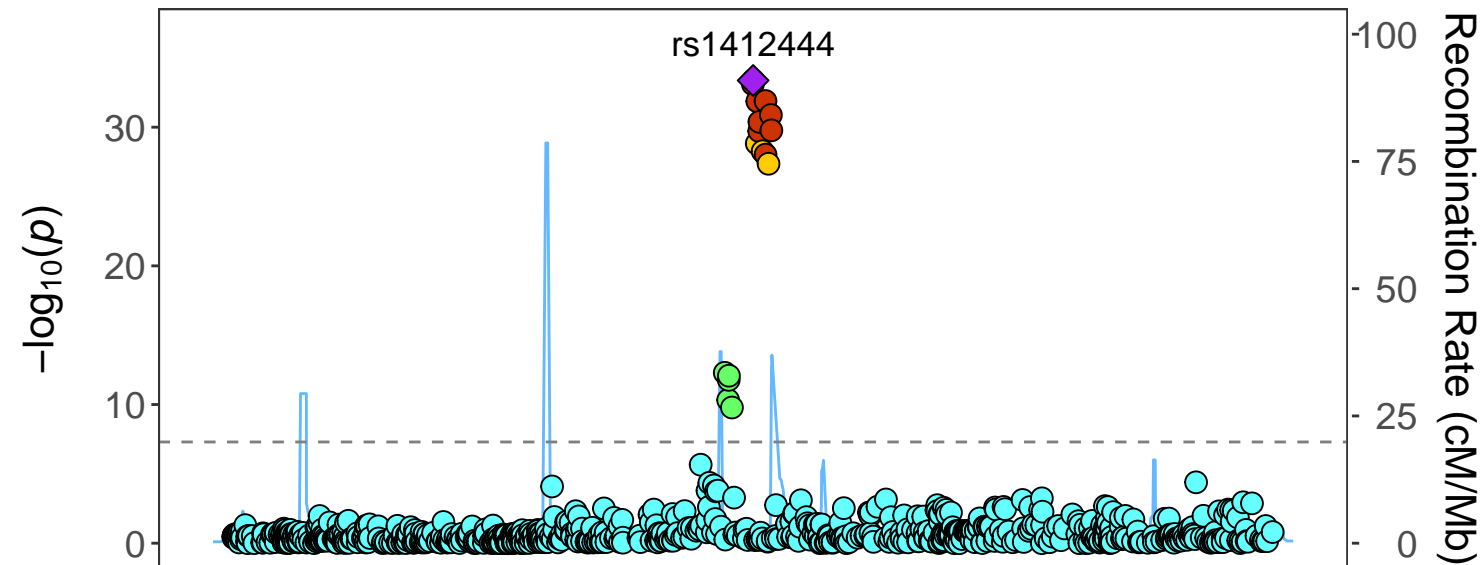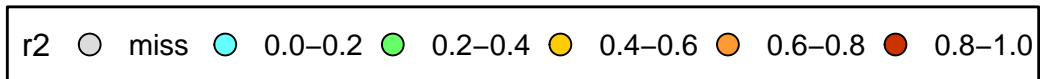

Supplement: Supplementary file 5 — Supporting Information [file CTM2-16-e70732-s001.zip › LocusZoom/Sfig_rs1412444_locusZoom.pdf]

# LocusZoom plots of GWAS top lead SNP

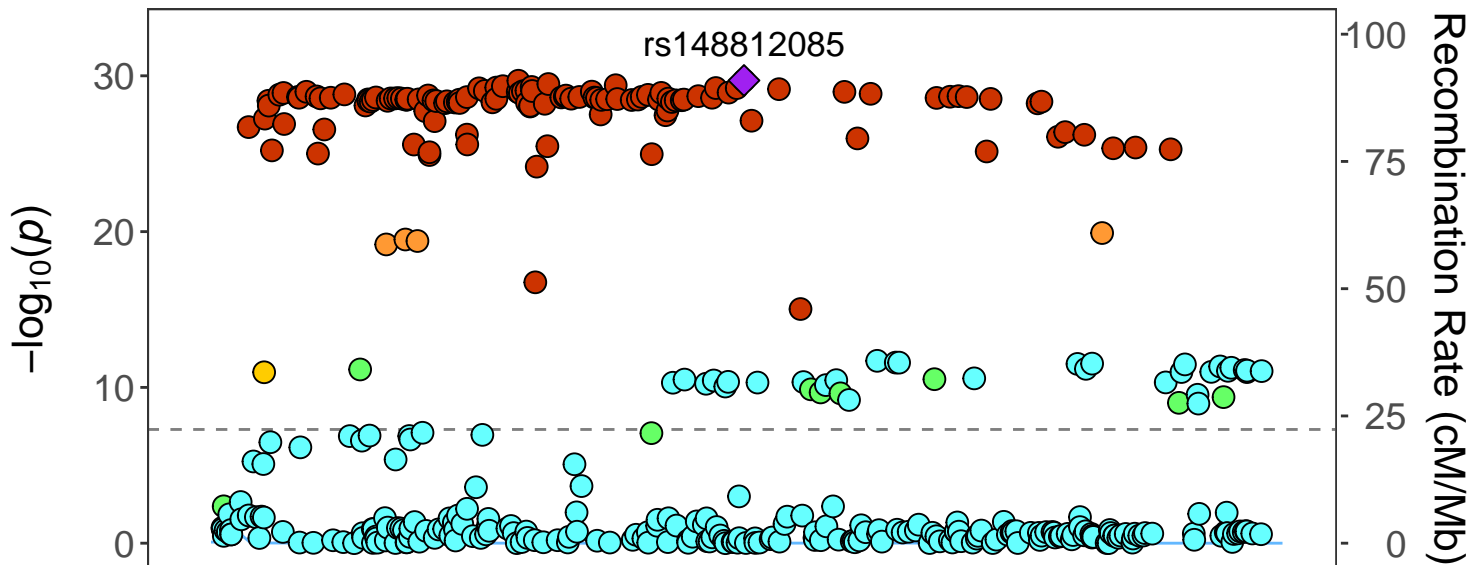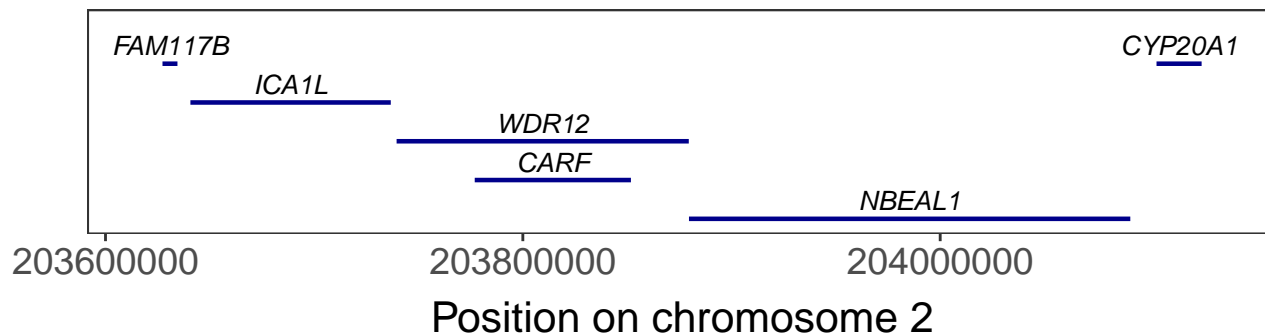

r2    miss    0.0-0.2    0.2-0.4    0.4-0.6    0.6-0.8    0.8-1.0

Supplement: Supplementary file 5 — Supporting Information [file CTM2-16-e70732-s001.zip › LocusZoom/Sfig_rs148812085_locusZoom.pdf]

# LocusZoom plots of GWAS top lead SNP

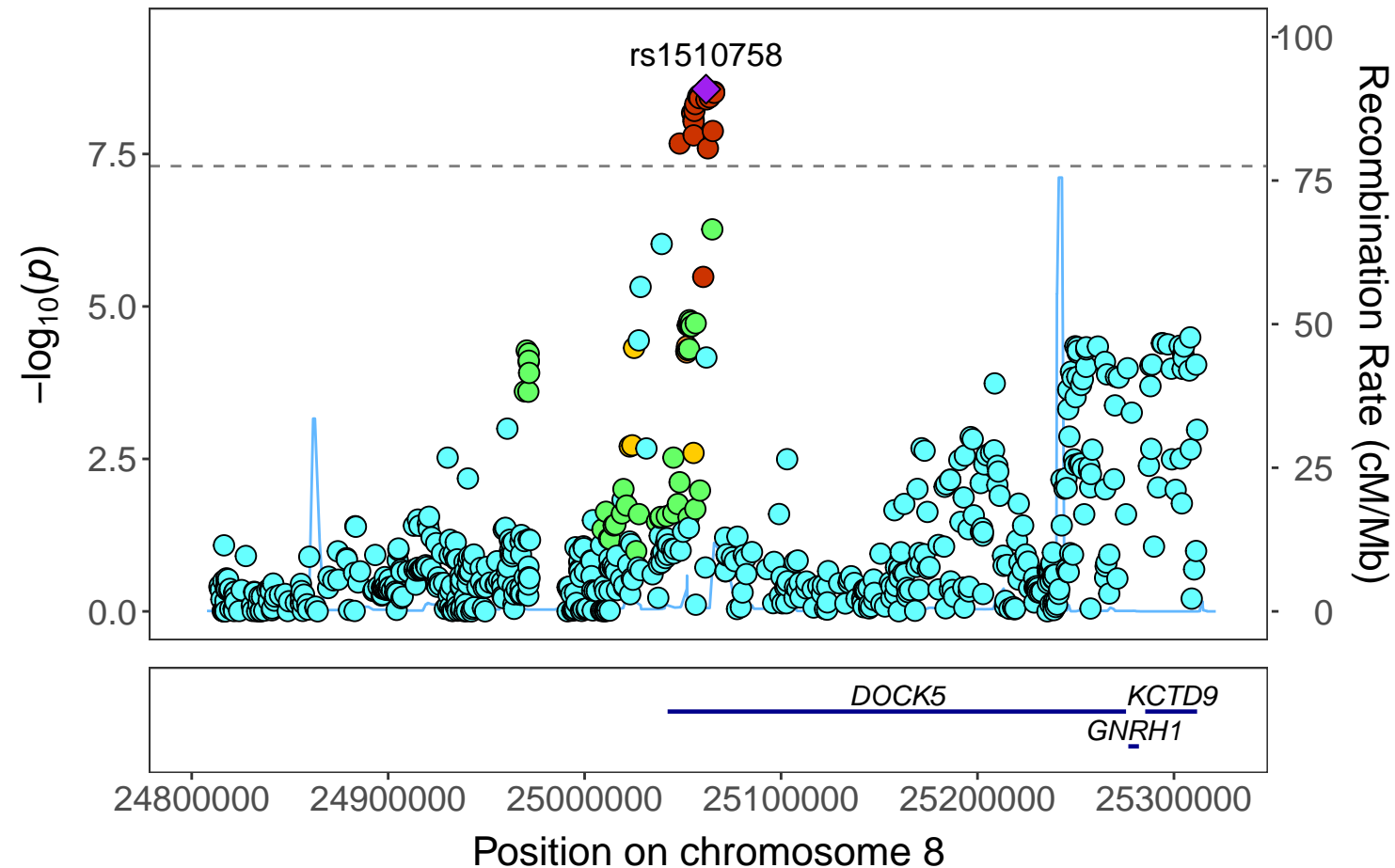

$r^2$    miss   0.0–0.2   0.2–0.4   0.4–0.6   0.6–0.8   0.8–1.0

Supplement: Supplementary file 5 — Supporting Information [file CTM2-16-e70732-s001.zip › LocusZoom/Sfig_rs1510758_locusZoom.pdf]

# LocusZoom plots of GWAS top lead SNP

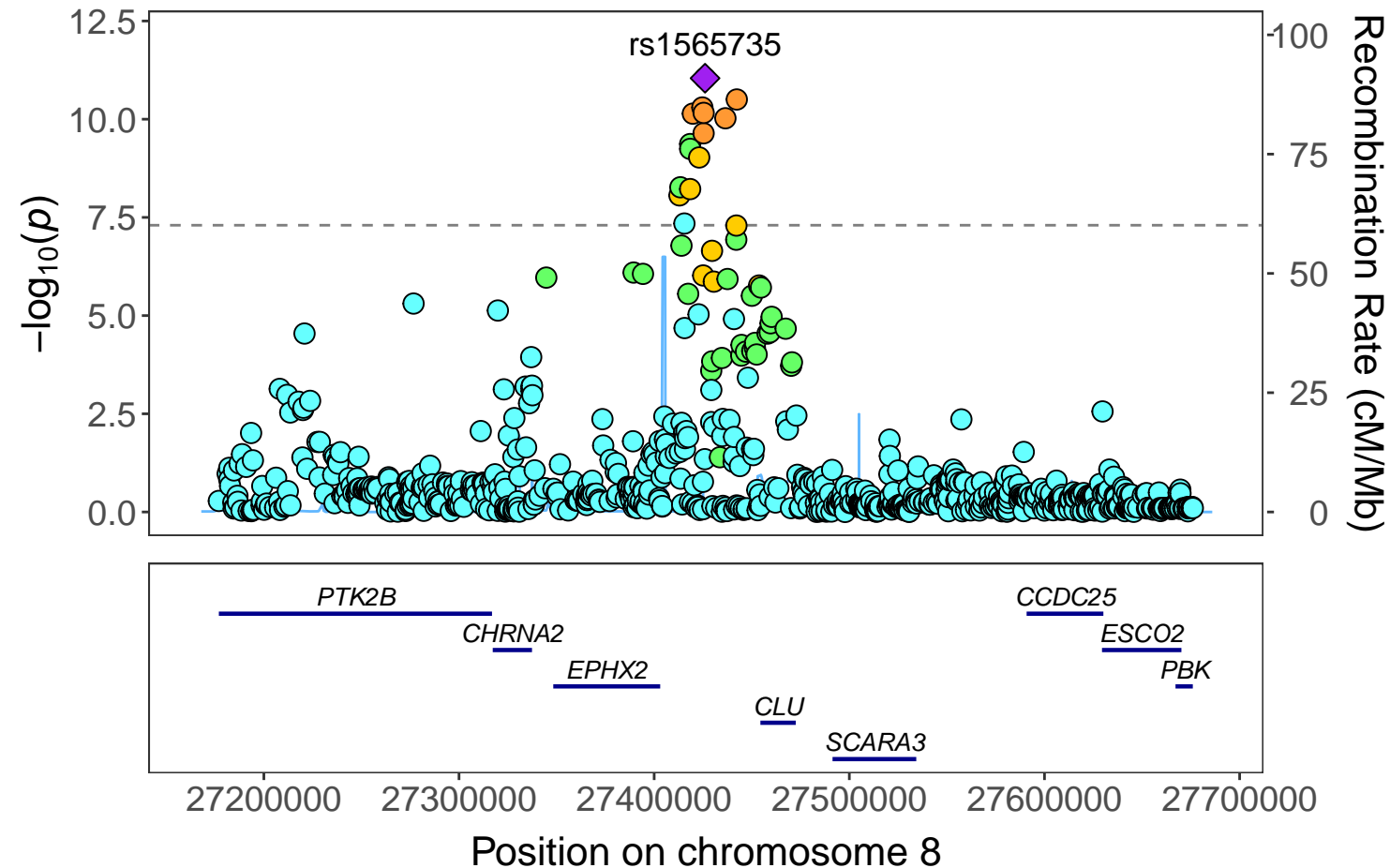

Supplement: Supplementary file 5 — Supporting Information [file CTM2-16-e70732-s001.zip › LocusZoom/Sfig_rs1565735_locusZoom.pdf]

# LocusZoom plots of GWAS top lead SNP

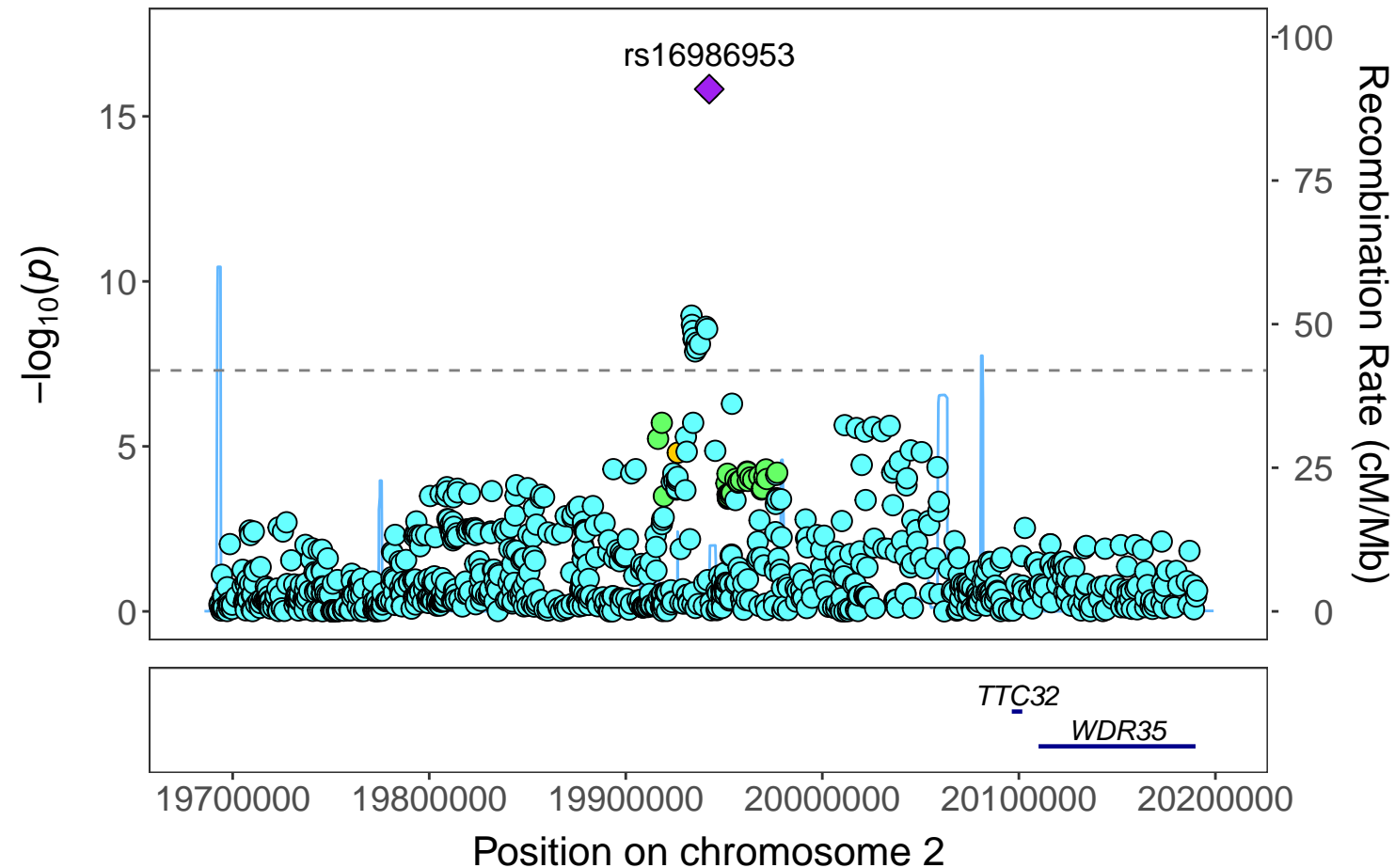

r2    miss    0.0–0.2    0.2–0.4    0.4–0.6    0.6–0.8    0.8–1.0

Supplement: Supplementary file 5 — Supporting Information [file CTM2-16-e70732-s001.zip › LocusZoom/Sfig_rs16986953_locusZoom.pdf]

# LocusZoom plots of GWAS top lead SNP

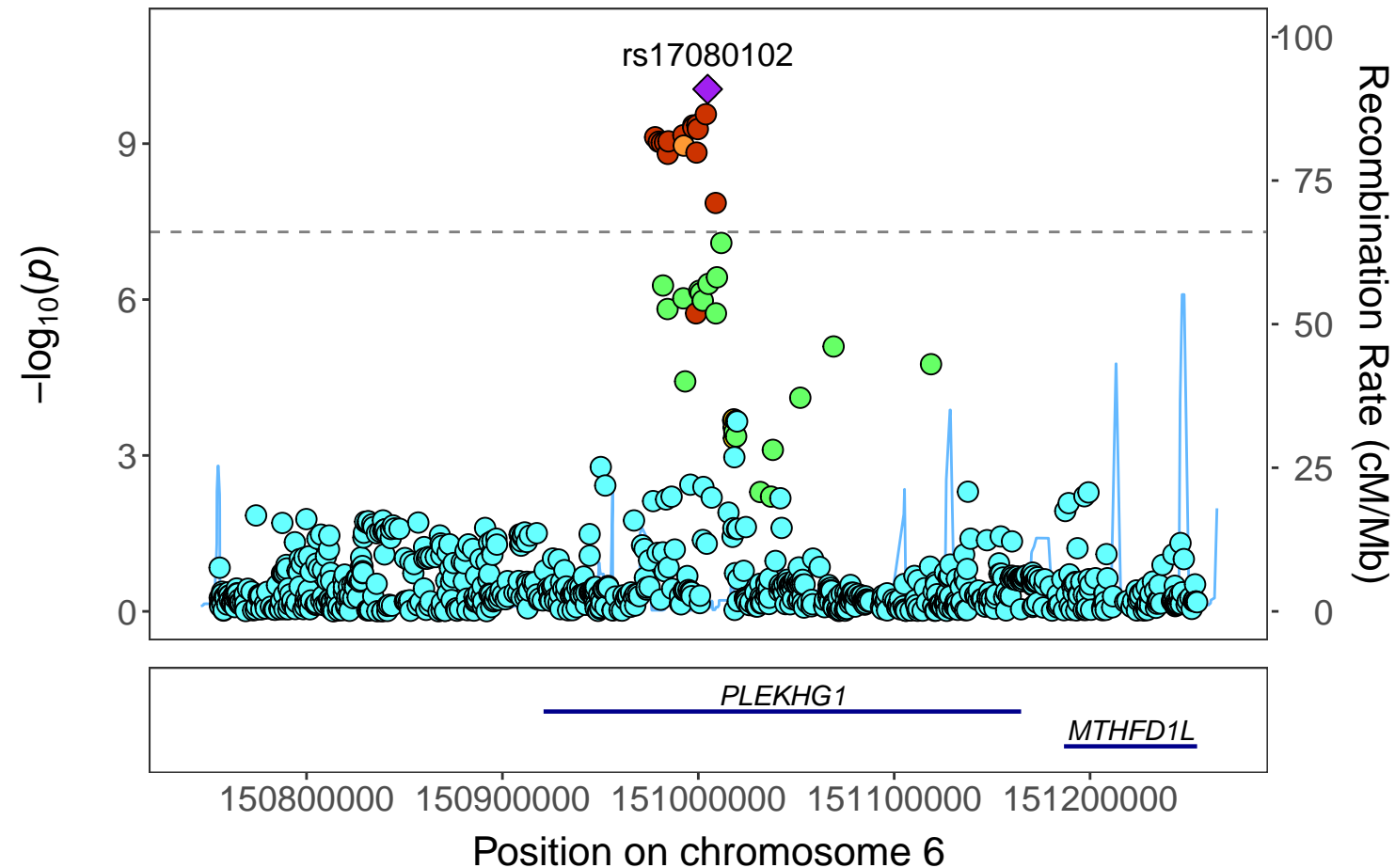

r2    miss    0.0–0.2    0.2–0.4    0.4–0.6    0.6–0.8    0.8–1.0

Supplement: Supplementary file 5 — Supporting Information [file CTM2-16-e70732-s001.zip › LocusZoom/Sfig_rs17080102_locusZoom.pdf]

# LocusZoom plots of GWAS top lead SNP

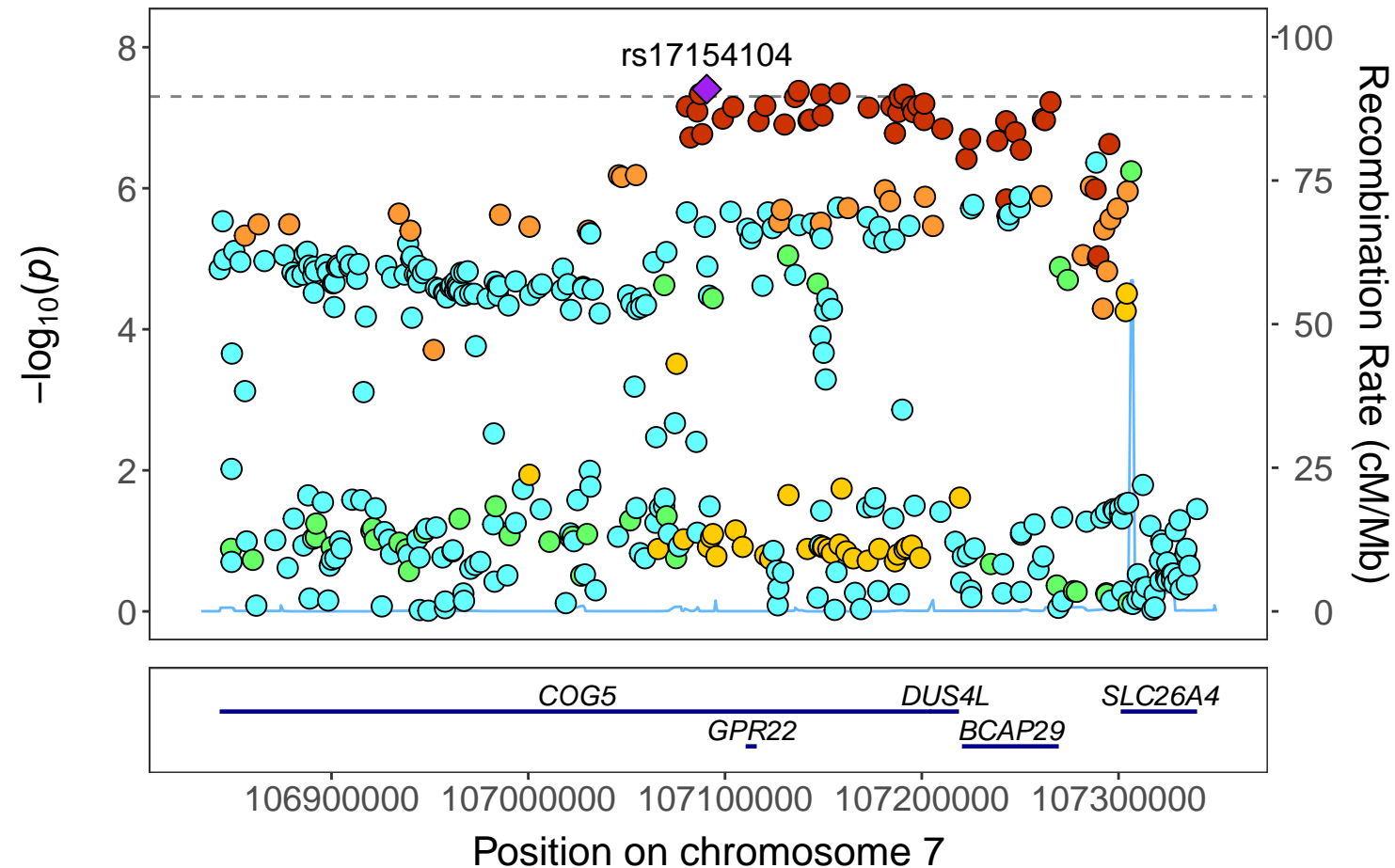

$r^2$  ○ miss ○ 0.0–0.2 ○ 0.2–0.4 ○ 0.4–0.6 ○ 0.6–0.8 ○ 0.8–1.0

Supplement: Supplementary file 5 — Supporting Information [file CTM2-16-e70732-s001.zip › LocusZoom/Sfig_rs17154104_locusZoom.pdf]

# LocusZoom plots of GWAS top lead SNP

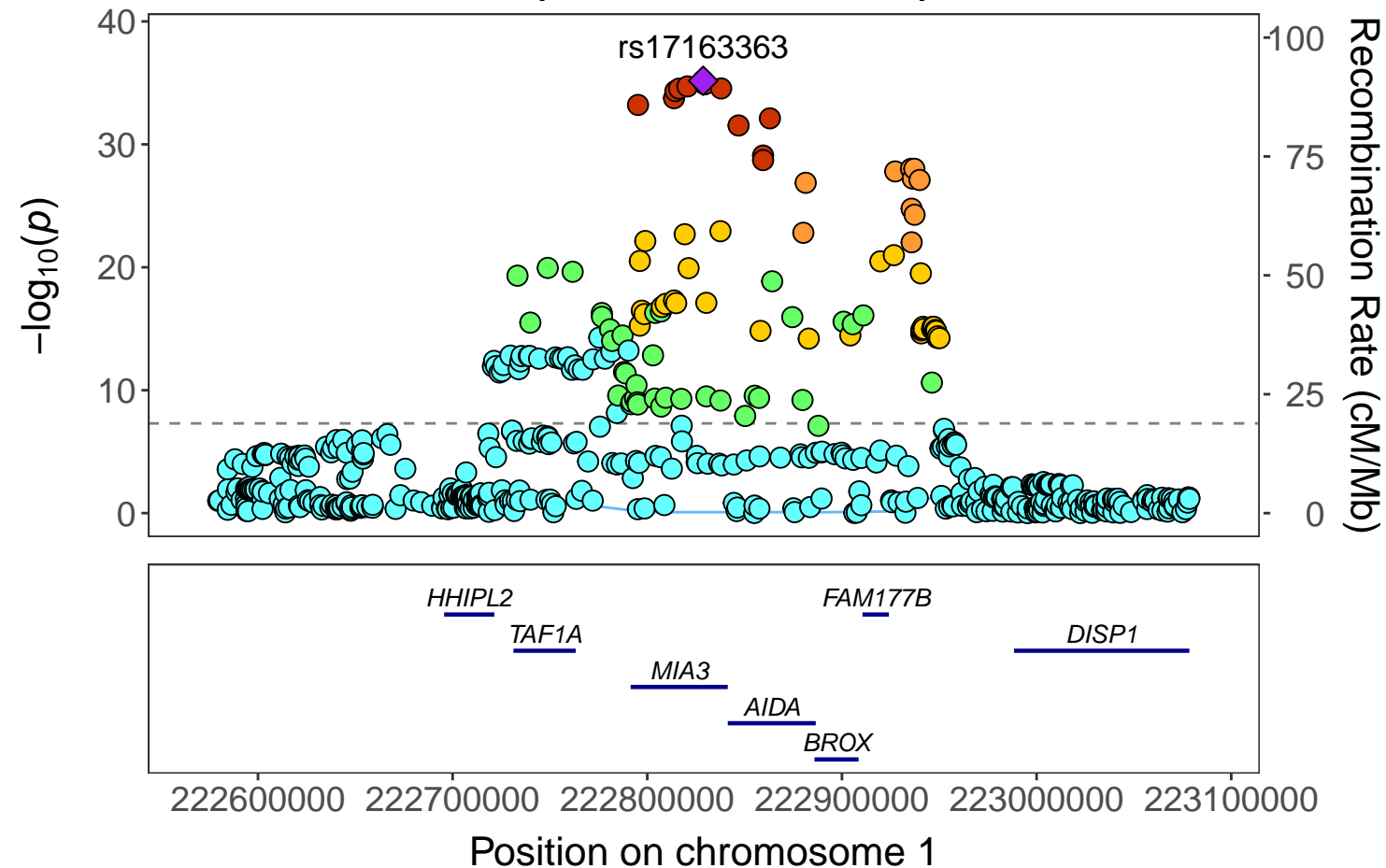

r2   miss   0.0-0.2   0.2-0.4   0.4-0.6   0.6-0.8   0.8-1.0

Supplement: Supplementary file 5 — Supporting Information [file CTM2-16-e70732-s001.zip › LocusZoom/Sfig_rs17163363_locusZoom.pdf]

# LocusZoom plots of GWAS top lead SNP

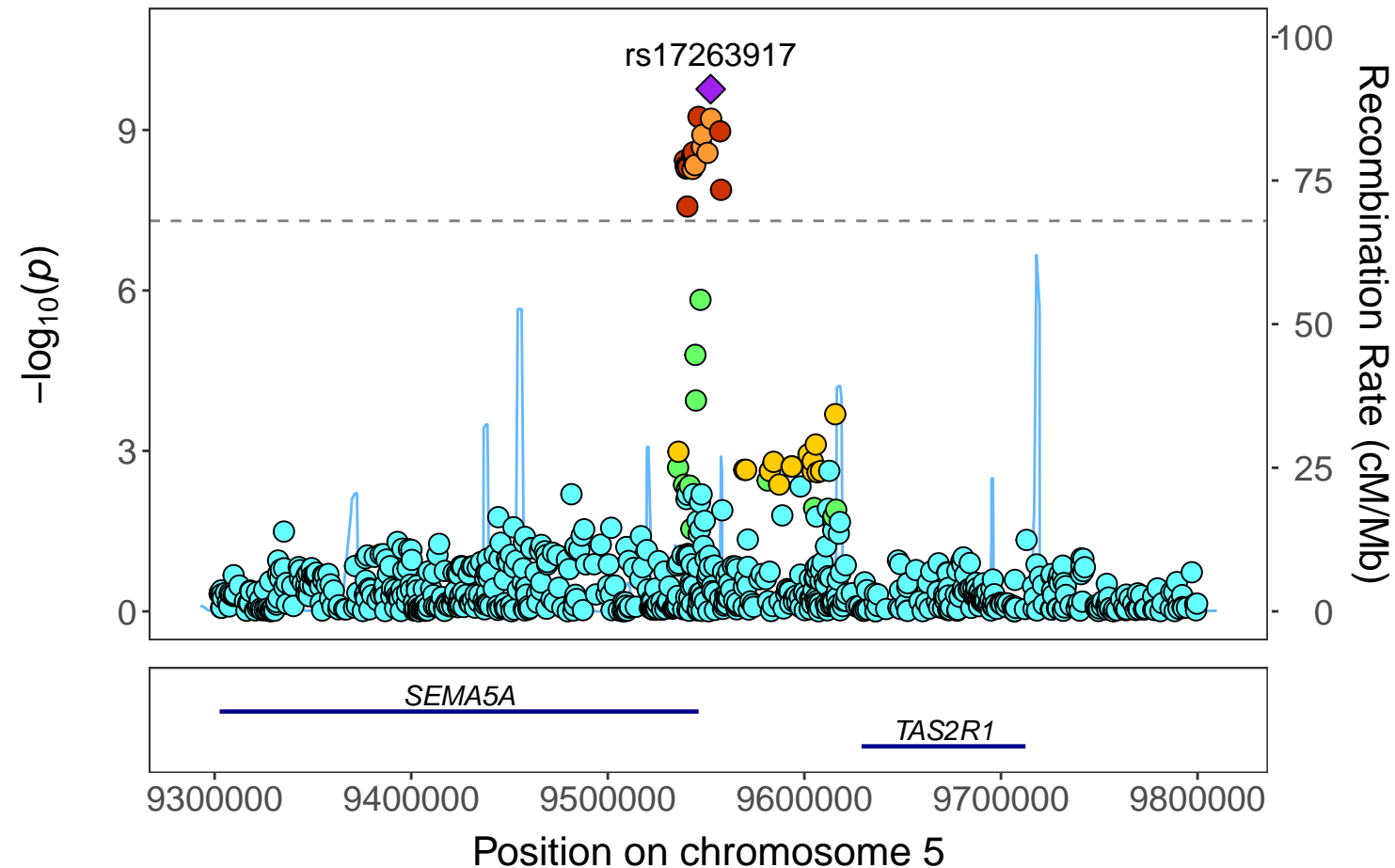

$r^2$    miss   0.0-0.2   0.2-0.4   0.4-0.6   0.6-0.8   0.8-1.0

Supplement: Supplementary file 5 — Supporting Information [file CTM2-16-e70732-s001.zip › LocusZoom/Sfig_rs17263917_locusZoom.pdf]

# LocusZoom plots of GWAS top lead SNP

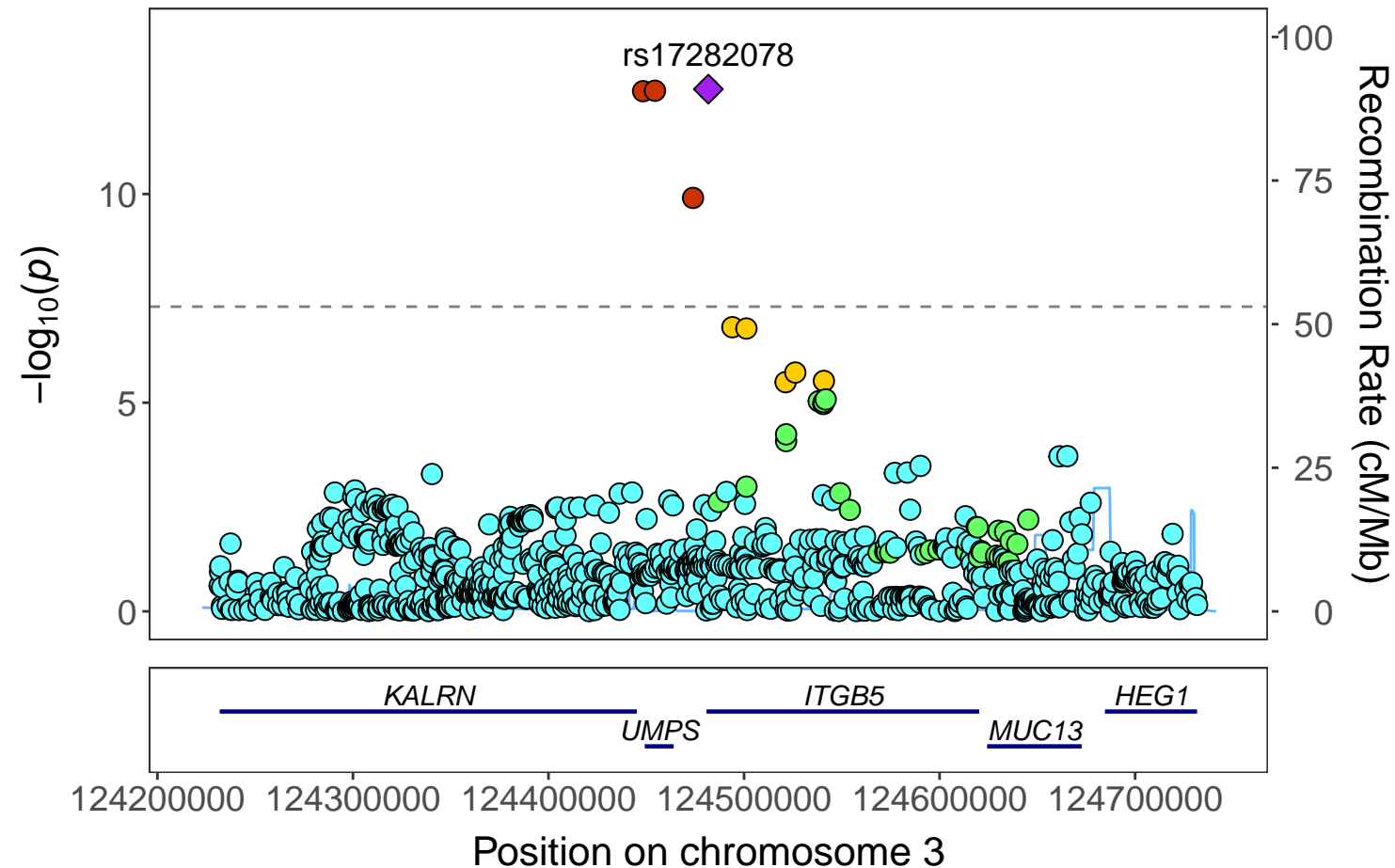

r2    miss    0.0–0.2    0.2–0.4    0.4–0.6    0.6–0.8    0.8–1.0

Supplement: Supplementary file 5 — Supporting Information [file CTM2-16-e70732-s001.zip › LocusZoom/Sfig_rs17282078_locusZoom.pdf]

# LocusZoom plots of GWAS top lead SNP

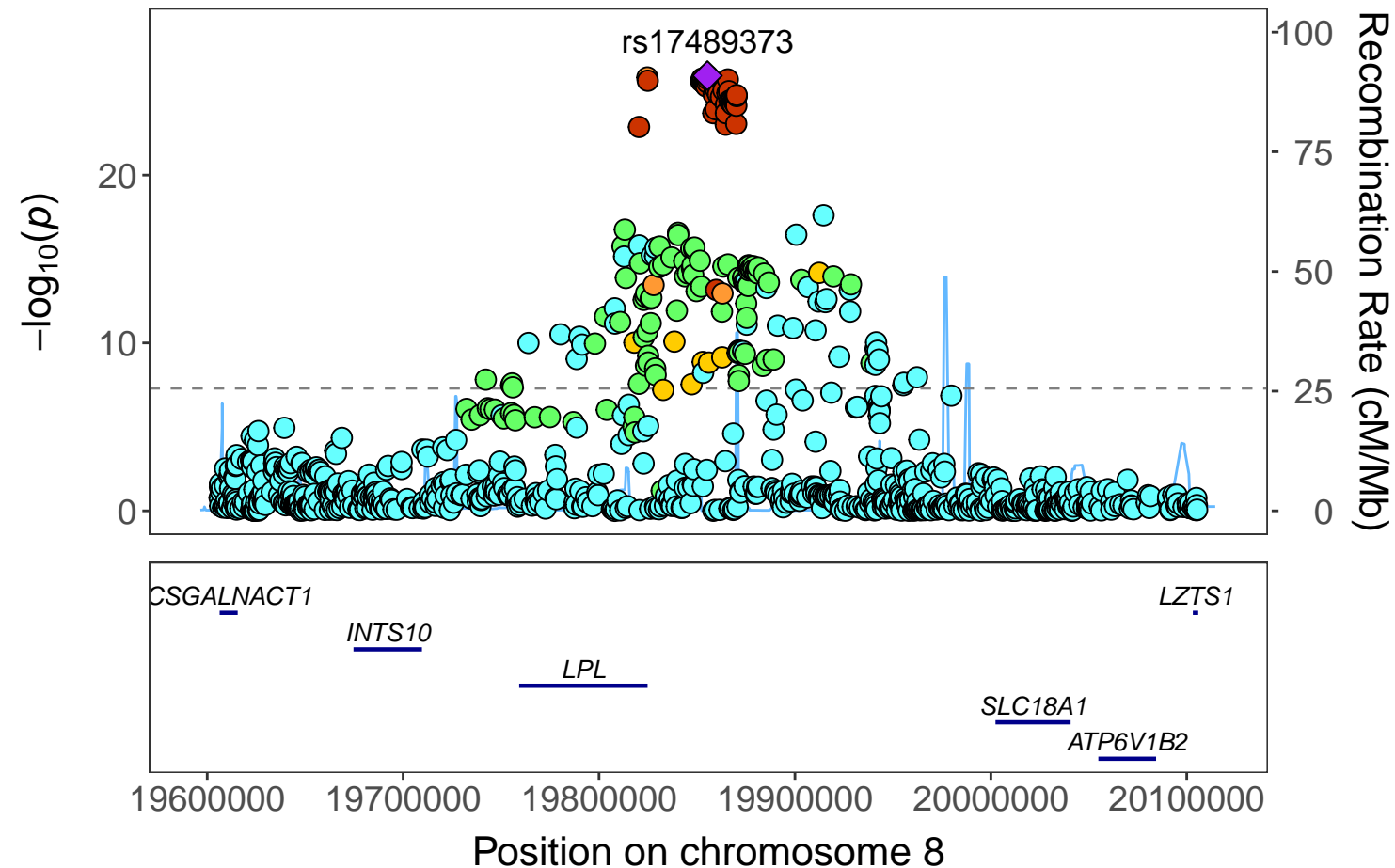

Supplement: Supplementary file 5 — Supporting Information [file CTM2-16-e70732-s001.zip › LocusZoom/Sfig_rs17489373_locusZoom.pdf]

# LocusZoom plots of GWAS top lead SNP

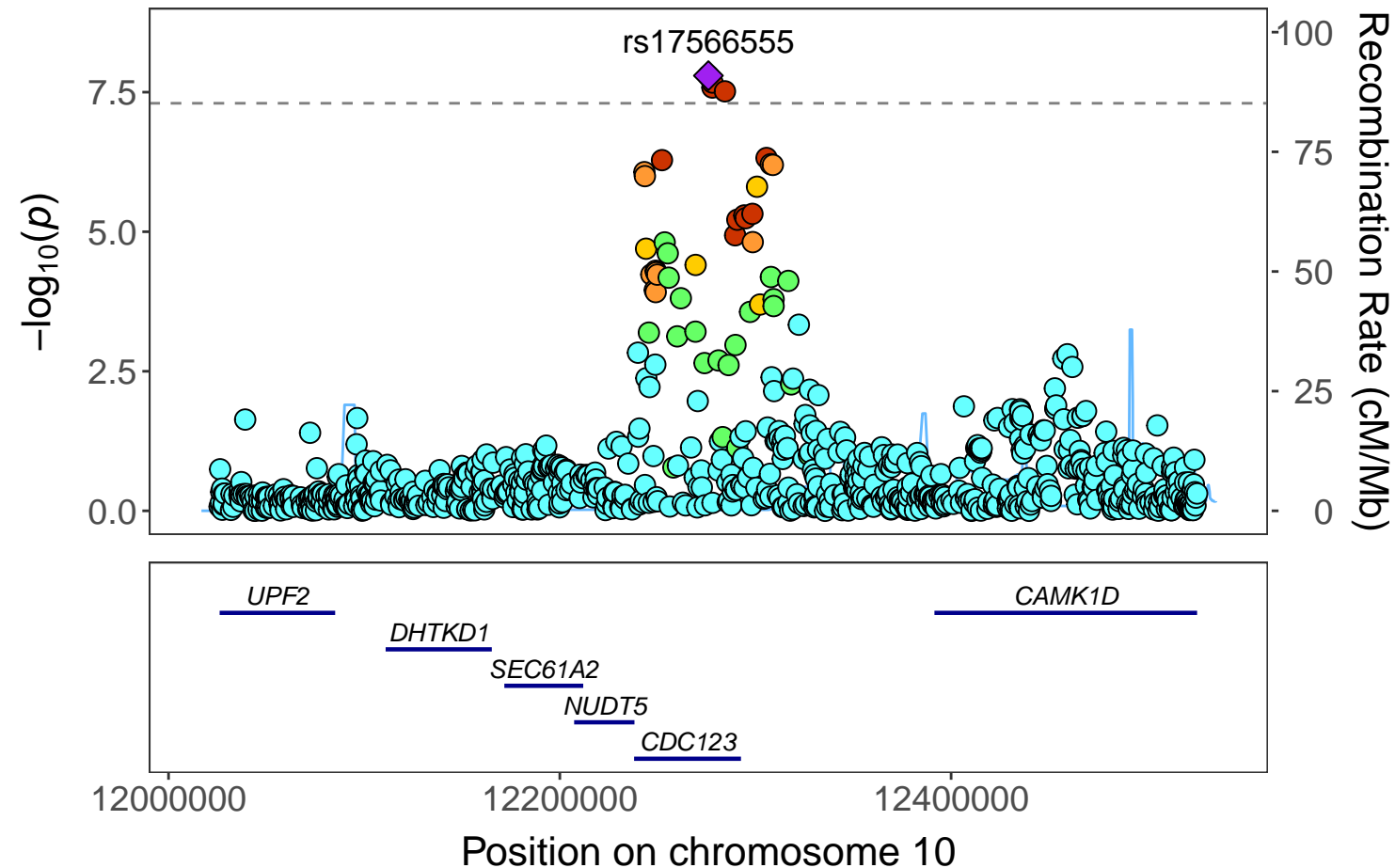

$r^2$  ○ miss ○ 0.0–0.2 ○ 0.2–0.4 ○ 0.4–0.6 ○ 0.6–0.8 ○ 0.8–1.0

Supplement: Supplementary file 5 — Supporting Information [file CTM2-16-e70732-s001.zip › LocusZoom/Sfig_rs17566555_locusZoom.pdf]

# LocusZoom plots of GWAS top lead SNP

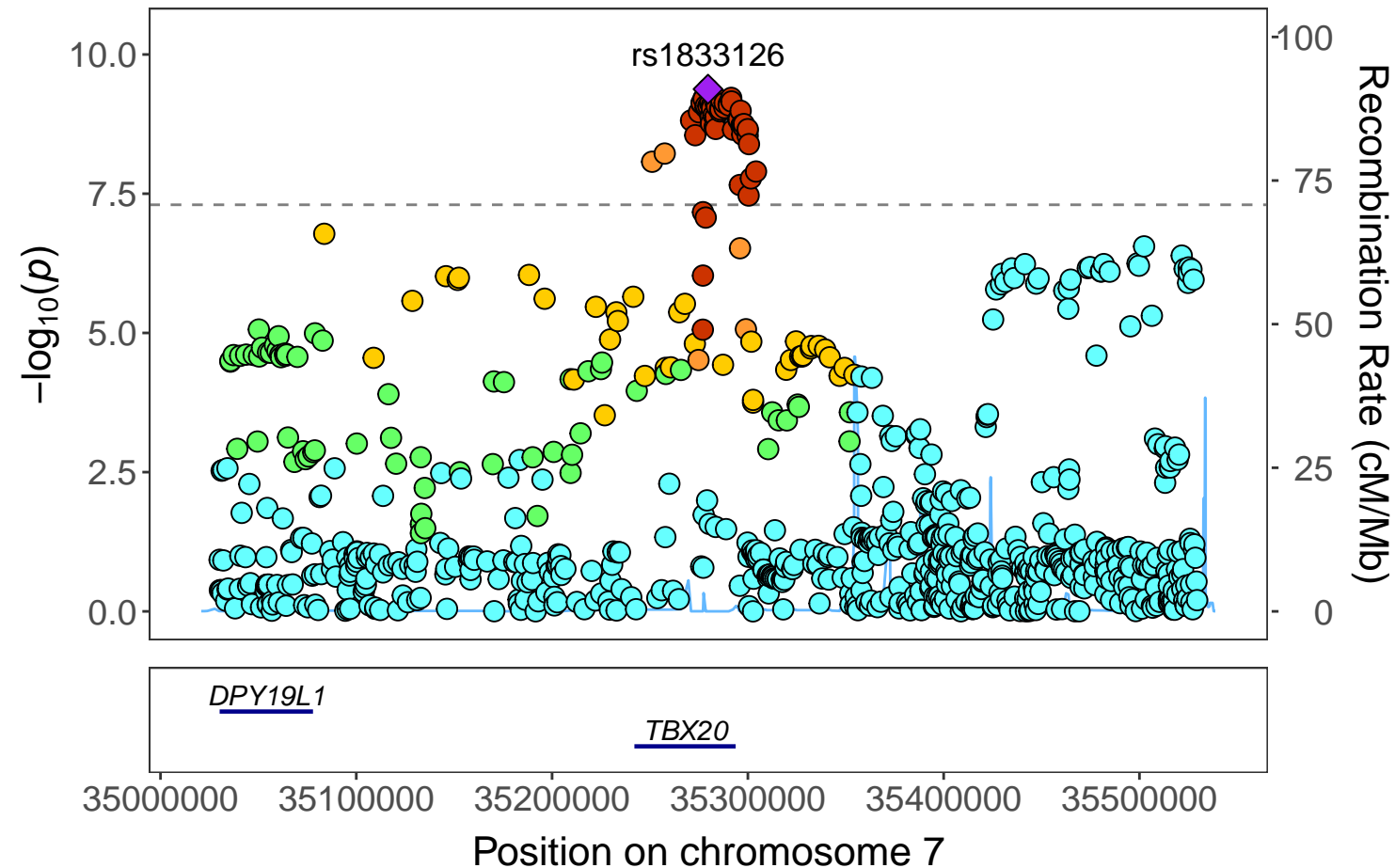

$r^2$    miss   0.0–0.2   0.2–0.4   0.4–0.6   0.6–0.8   0.8–1.0

Supplement: Supplementary file 5 — Supporting Information [file CTM2-16-e70732-s001.zip › LocusZoom/Sfig_rs1833126_locusZoom.pdf]

# *LocusZoom plots of GWAS top lead SNP*

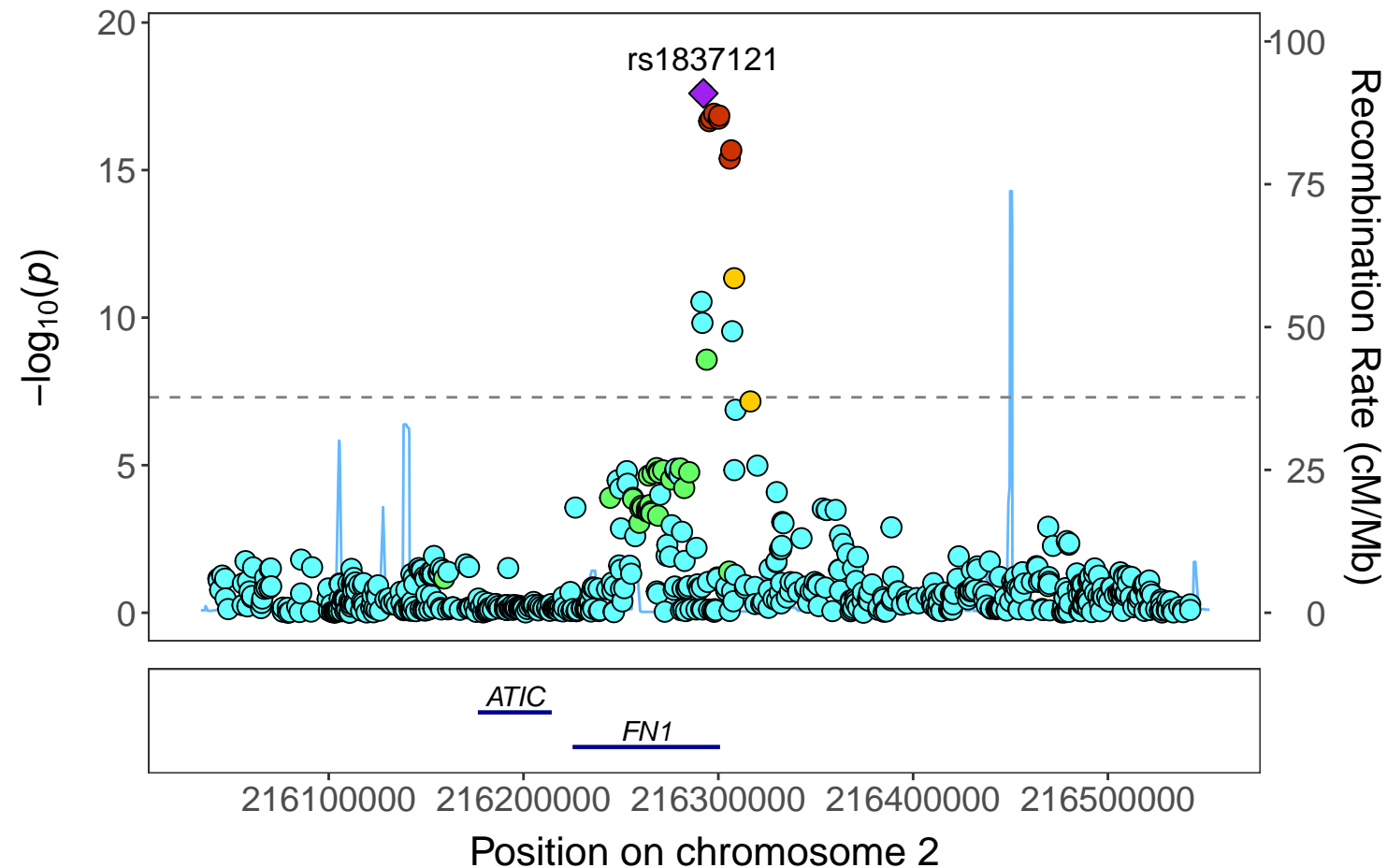

r2    ○ miss    ● 0.0–0.2    ● 0.2–0.4    ● 0.4–0.6    ● 0.6–0.8    ● 0.8–1.0

Supplement: Supplementary file 5 — Supporting Information [file CTM2-16-e70732-s001.zip › LocusZoom/Sfig_rs1837121_locusZoom.pdf]

# LocusZoom plots of GWAS top lead SNP

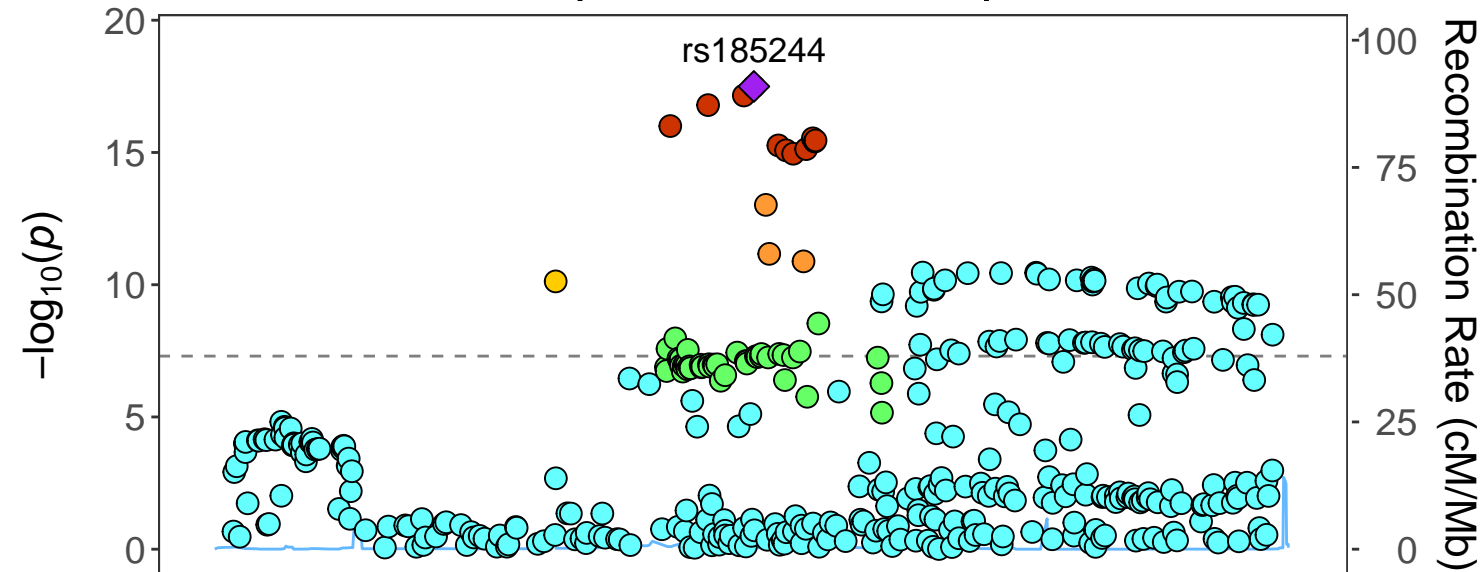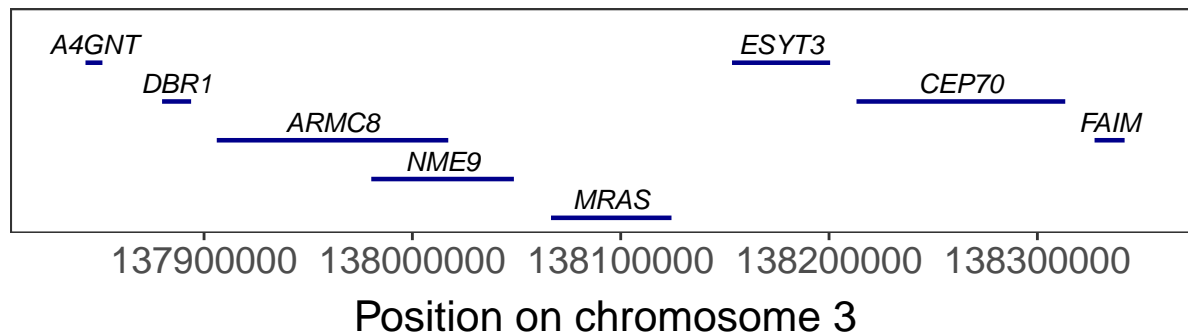

r2    miss    0.0-0.2    0.2-0.4    0.4-0.6    0.6-0.8    0.8-1.0

Supplement: Supplementary file 5 — Supporting Information [file CTM2-16-e70732-s001.zip › LocusZoom/Sfig_rs185244_locusZoom.pdf]

# *LocusZoom plots of GWAS top lead SNP*

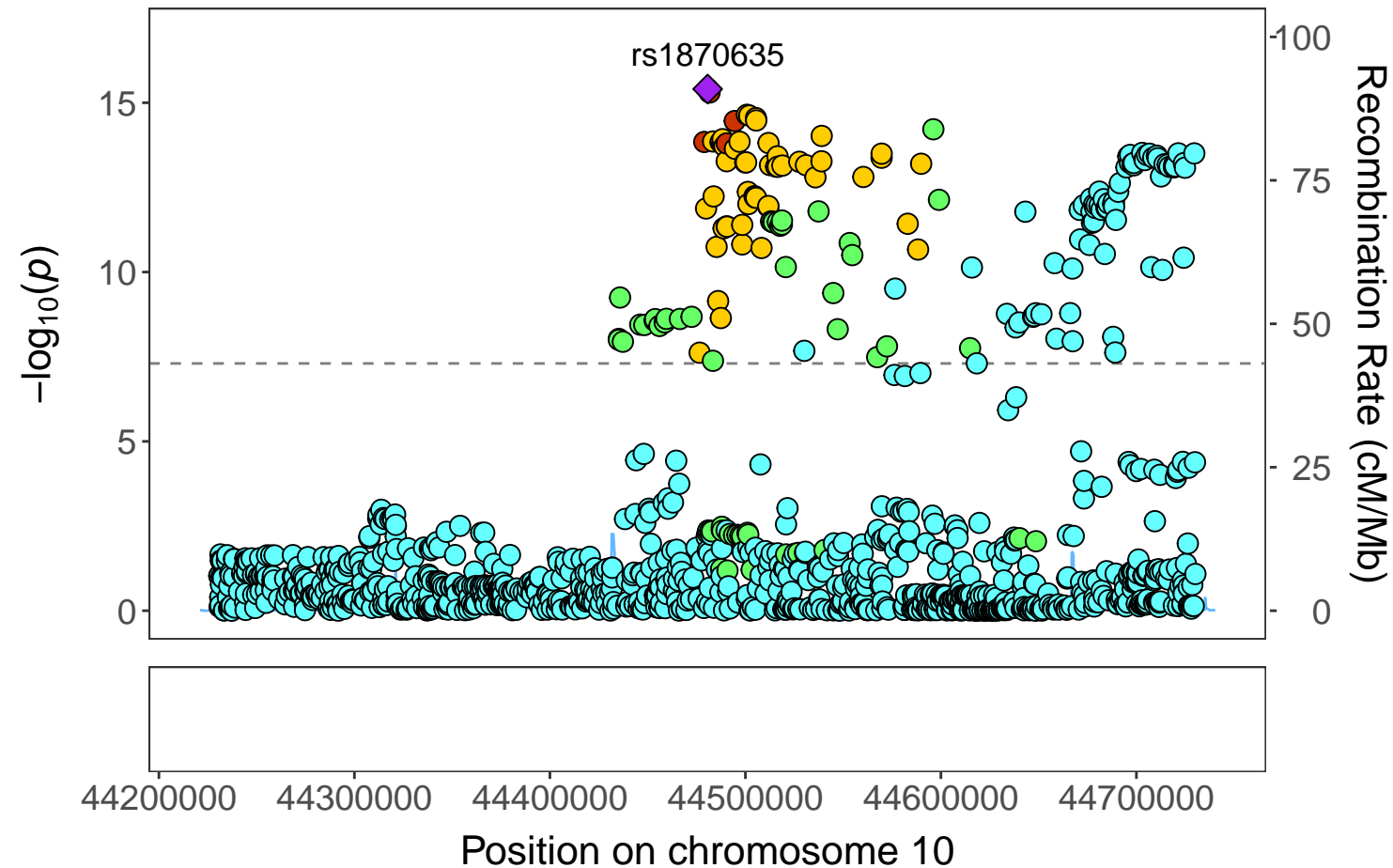

Supplement: Supplementary file 5 — Supporting Information [file CTM2-16-e70732-s001.zip › LocusZoom/Sfig_rs1870635_locusZoom.pdf]

# LocusZoom plots of GWAS top lead SNP

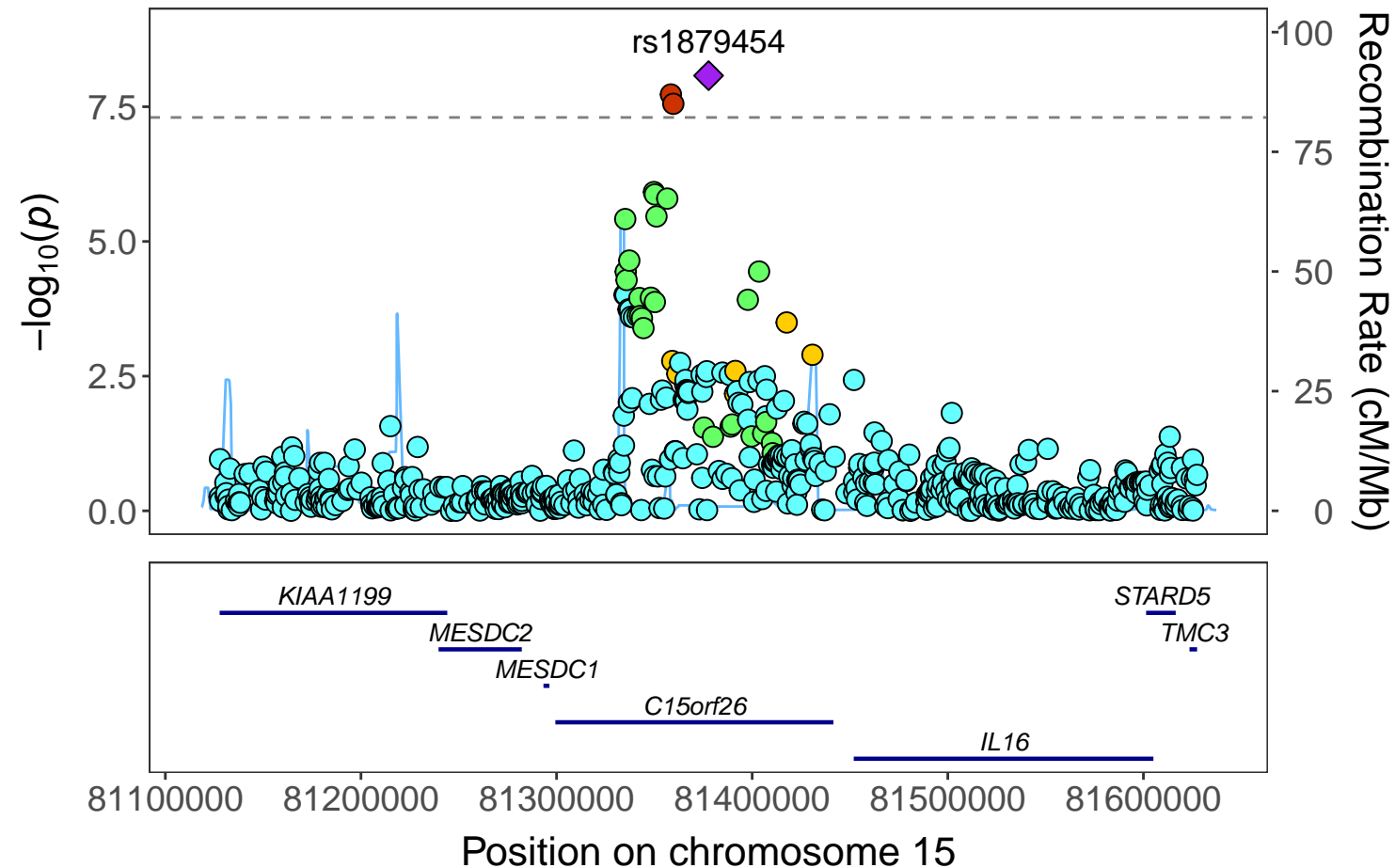

r2   miss   0.0–0.2   0.2–0.4   0.4–0.6   0.6–0.8   0.8–1.0

Supplement: Supplementary file 5 — Supporting Information [file CTM2-16-e70732-s001.zip › LocusZoom/Sfig_rs1879454_locusZoom.pdf]

# LocusZoom plots of GWAS top lead SNP

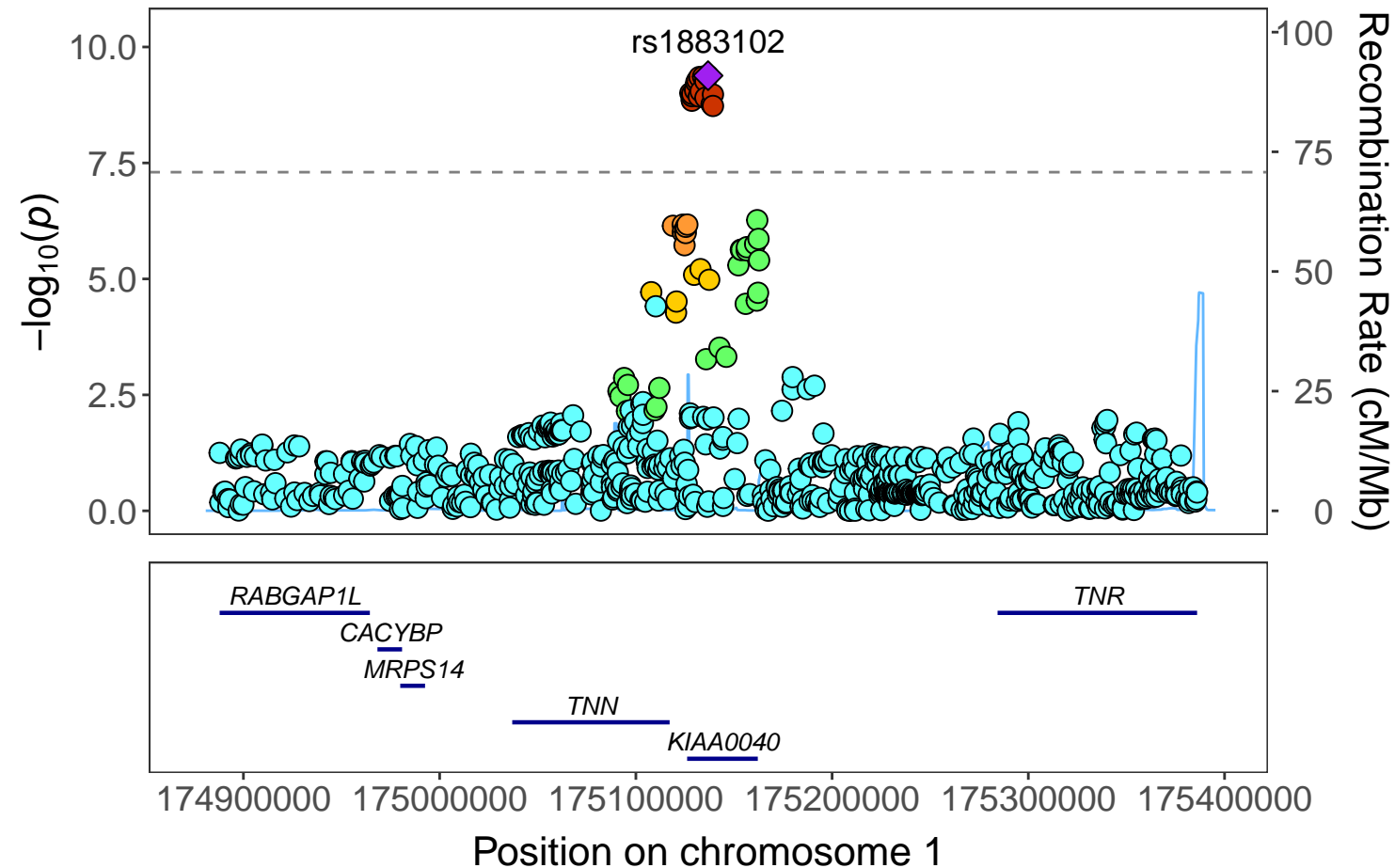

$r^2$    miss   0.0-0.2   0.2-0.4   0.4-0.6   0.6-0.8   0.8-1.0

Supplement: Supplementary file 5 — Supporting Information [file CTM2-16-e70732-s001.zip › LocusZoom/Sfig_rs1883102_locusZoom.pdf]

# *LocusZoom plots of GWAS top lead SNP*

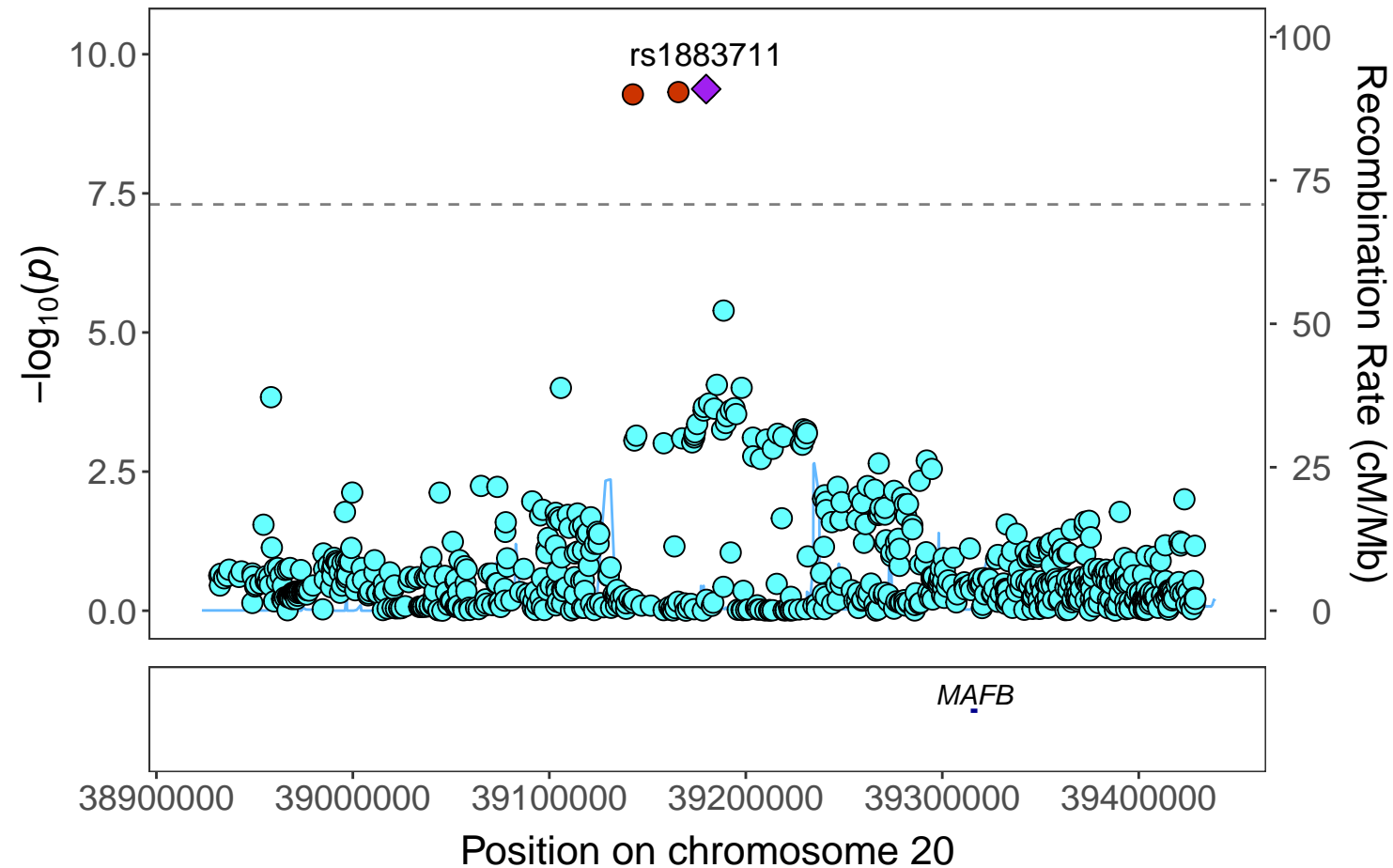

$r^2$    miss   0.0–0.2   0.2–0.4   0.4–0.6   0.6–0.8   0.8–1.0

Supplement: Supplementary file 5 — Supporting Information [file CTM2-16-e70732-s001.zip › LocusZoom/Sfig_rs1883711_locusZoom.pdf]

# LocusZoom plots of GWAS top lead SNP

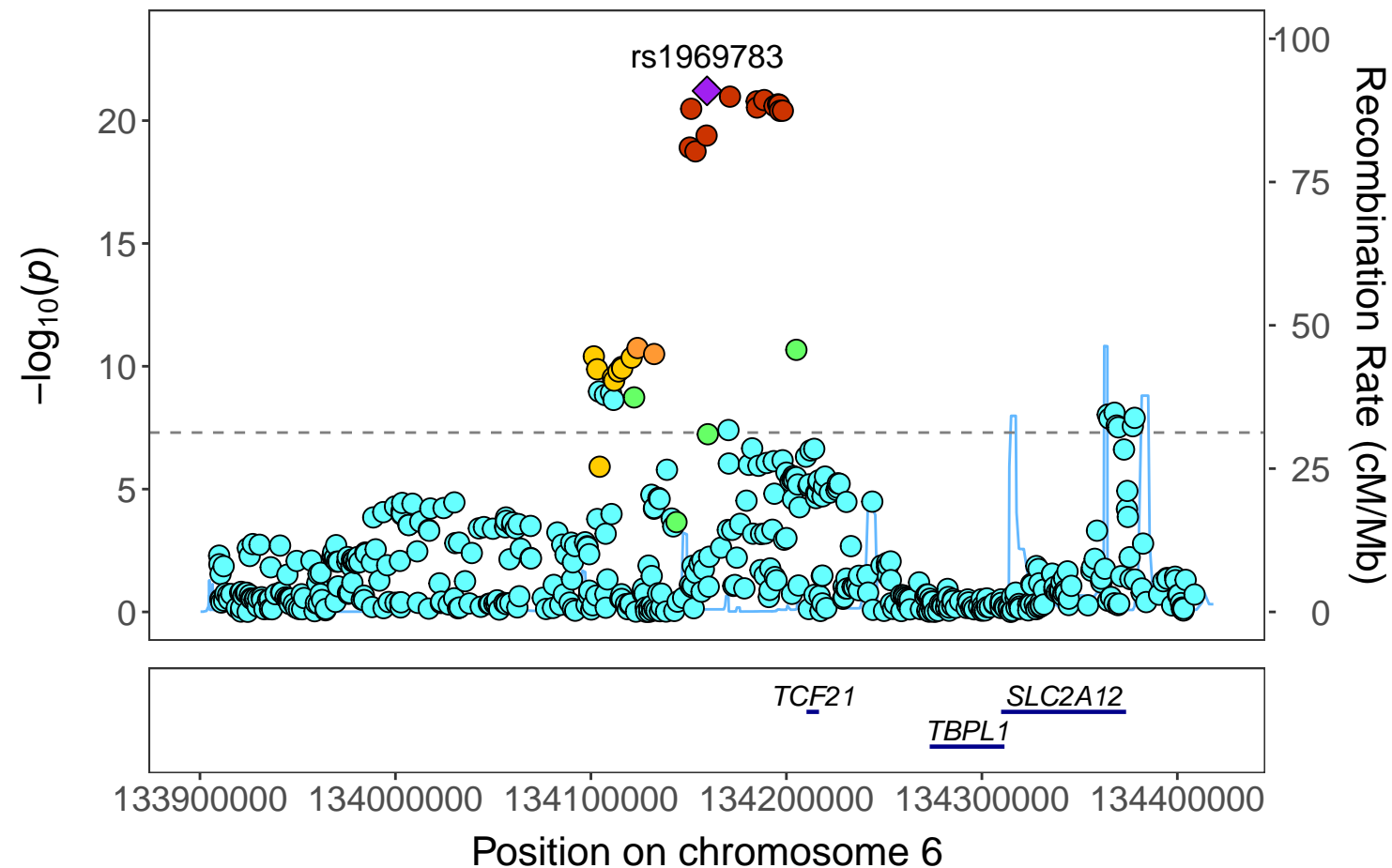

$r^2$     $\circ$  miss    $\circ$  0.0–0.2    $\circ$  0.2–0.4    $\circ$  0.4–0.6    $\circ$  0.6–0.8    $\circ$  0.8–1.0

Supplement: Supplementary file 5 — Supporting Information [file CTM2-16-e70732-s001.zip › LocusZoom/Sfig_rs1969783_locusZoom.pdf]

# *LocusZoom plots of GWAS top lead SNP*

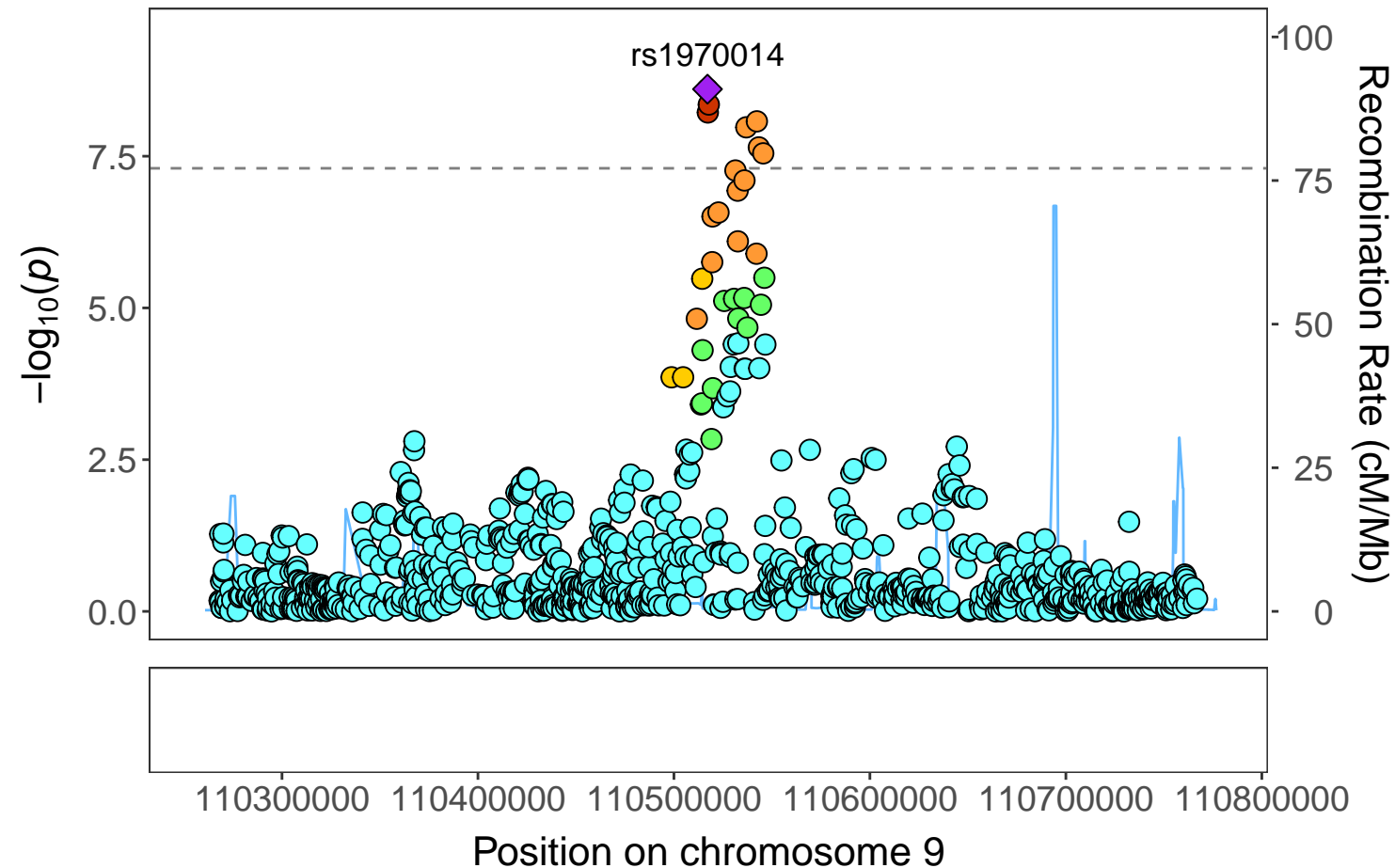

r2   miss   0.0–0.2   0.2–0.4   0.4–0.6   0.6–0.8   0.8–1.0

Supplement: Supplementary file 5 — Supporting Information [file CTM2-16-e70732-s001.zip › LocusZoom/Sfig_rs1970014_locusZoom.pdf]

# LocusZoom plots of GWAS top lead SNP

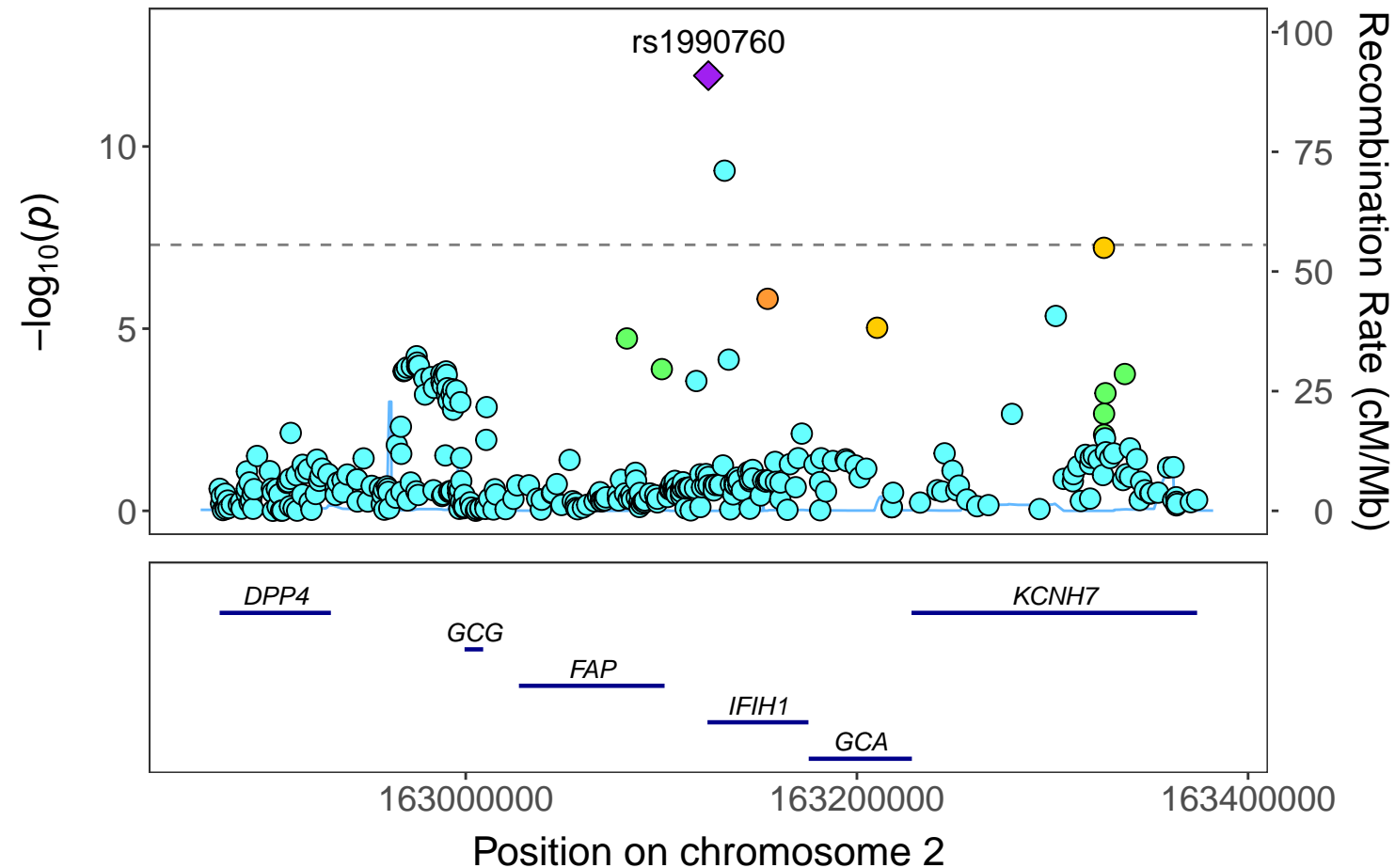

Supplement: Supplementary file 5 — Supporting Information [file CTM2-16-e70732-s001.zip › LocusZoom/Sfig_rs1990760_locusZoom.pdf]

# LocusZoom plots of GWAS top lead SNP

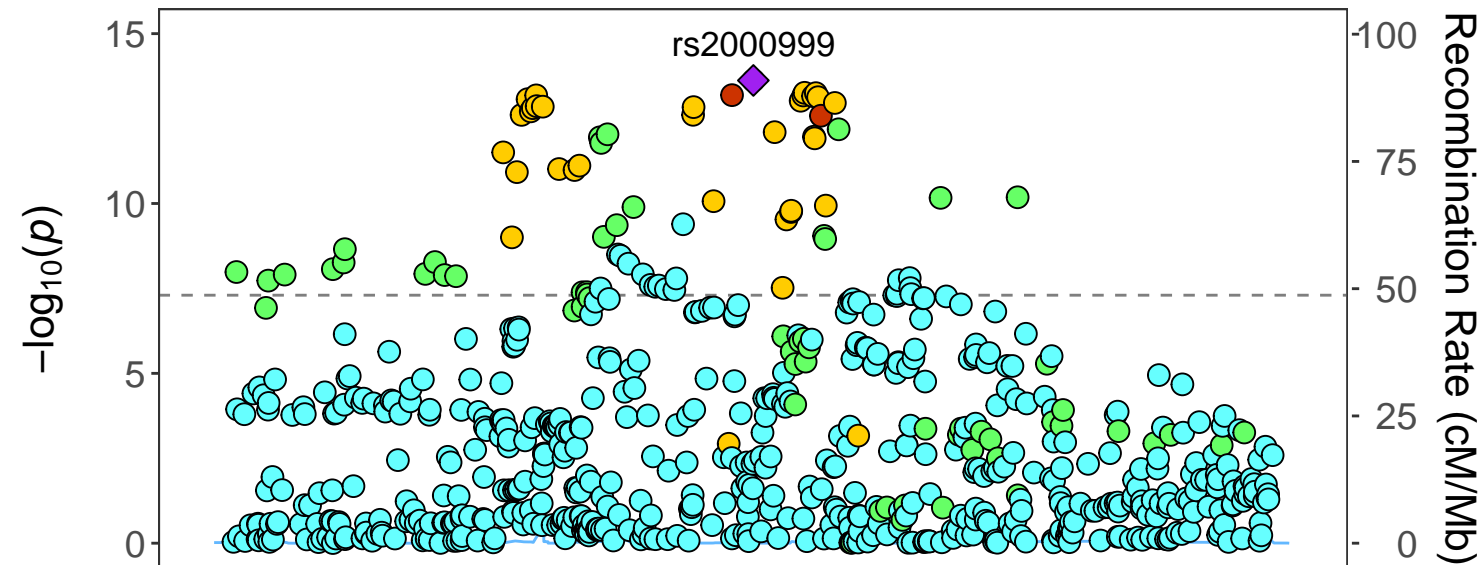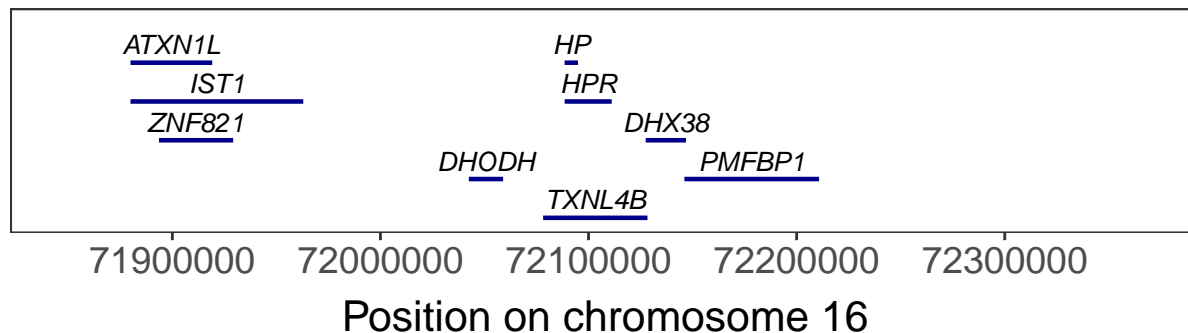

$r^2$     $\circ$  miss    $\circ$  0.0-0.2    $\circ$  0.2-0.4    $\circ$  0.4-0.6    $\circ$  0.6-0.8    $\circ$  0.8-1.0

Supplement: Supplementary file 5 — Supporting Information [file CTM2-16-e70732-s001.zip › LocusZoom/Sfig_rs2000999_locusZoom.pdf]

# LocusZoom plots of GWAS top lead SNP

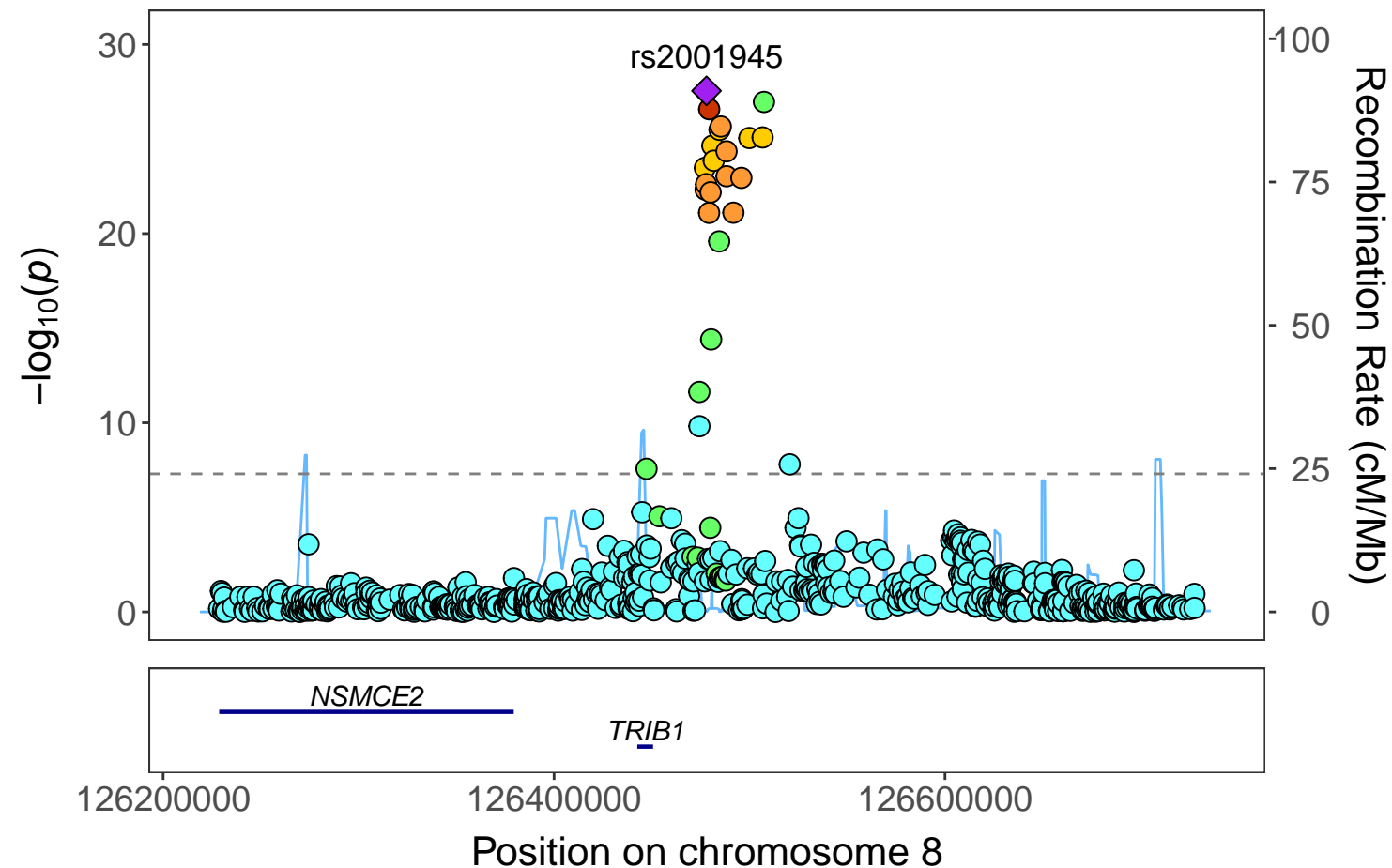

$r^2$    miss   0.0–0.2   0.2–0.4   0.4–0.6   0.6–0.8   0.8–1.0

Supplement: Supplementary file 5 — Supporting Information [file CTM2-16-e70732-s001.zip › LocusZoom/Sfig_rs2001945_locusZoom.pdf]

# *LocusZoom plots of GWAS top lead SNP*

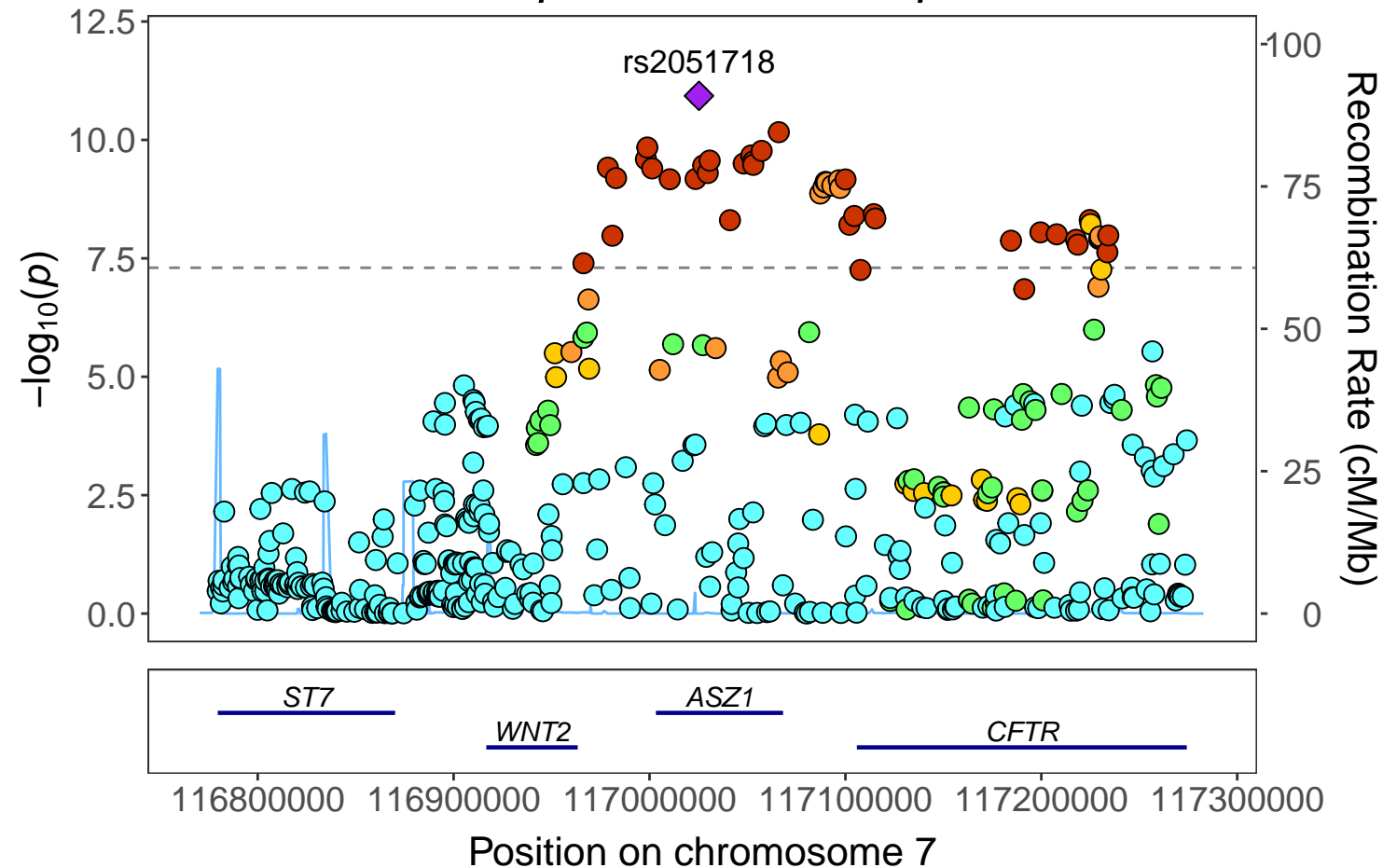

r2    miss    0.0–0.2    0.2–0.4    0.4–0.6    0.6–0.8    0.8–1.0

Supplement: Supplementary file 5 — Supporting Information [file CTM2-16-e70732-s001.zip › LocusZoom/Sfig_rs2051718_locusZoom.pdf]

# LocusZoom plots of GWAS top lead SNP

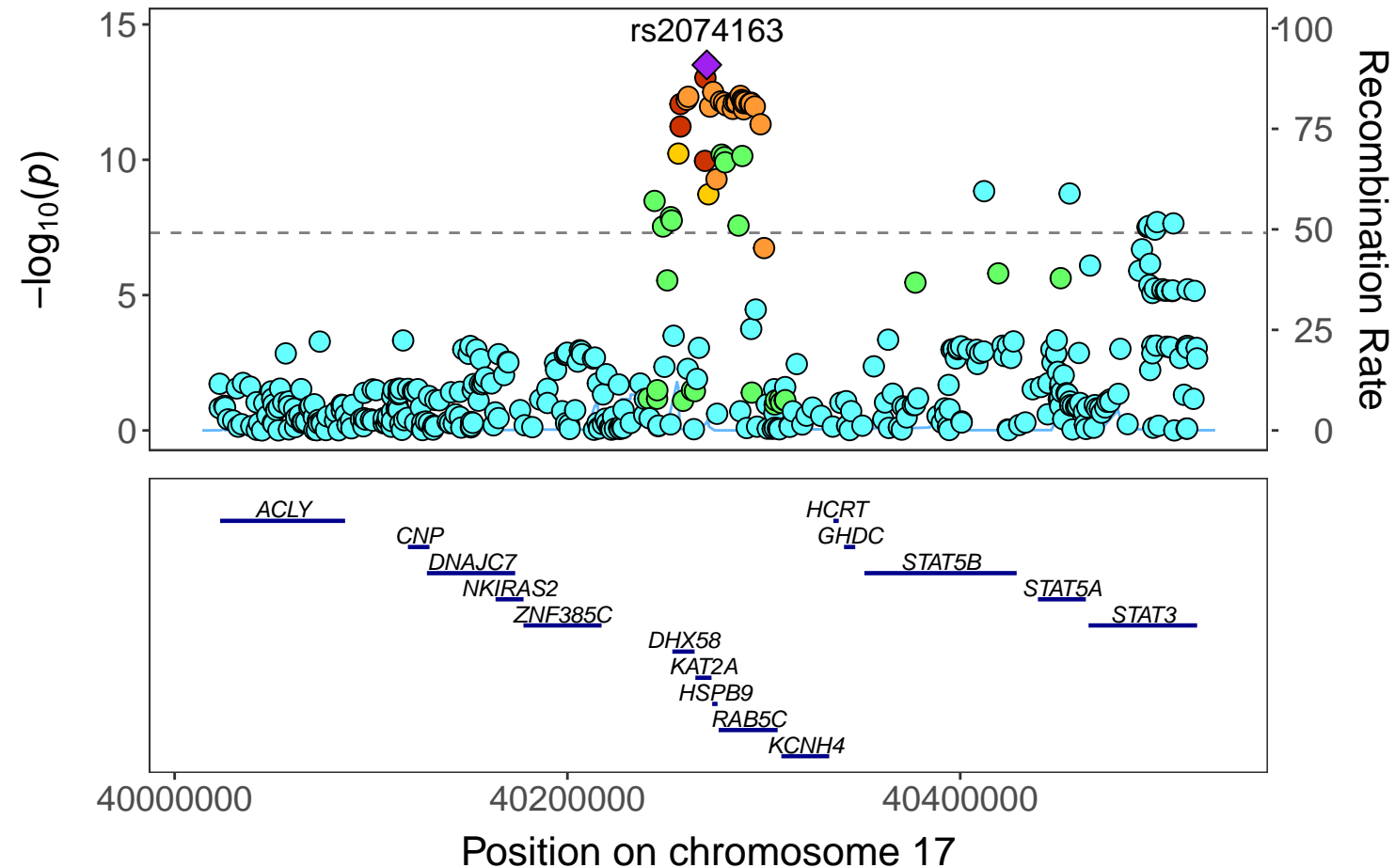

Supplement: Supplementary file 5 — Supporting Information [file CTM2-16-e70732-s001.zip › LocusZoom/Sfig_rs2074163_locusZoom.pdf]

# LocusZoom plots of GWAS top lead SNP

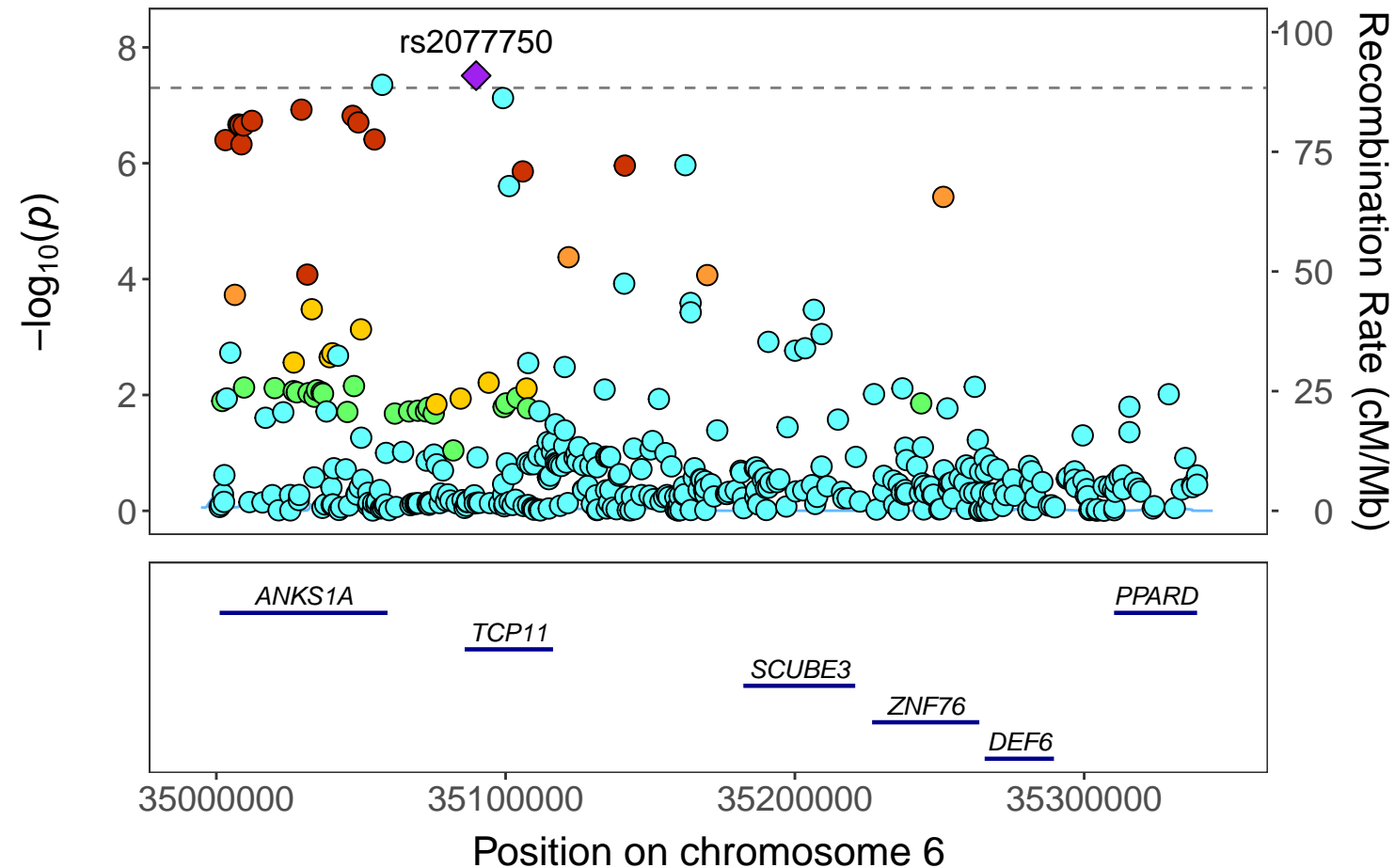

Supplement: Supplementary file 5 — Supporting Information [file CTM2-16-e70732-s001.zip › LocusZoom/Sfig_rs2077750_locusZoom.pdf]

# LocusZoom plots of GWAS top lead SNP

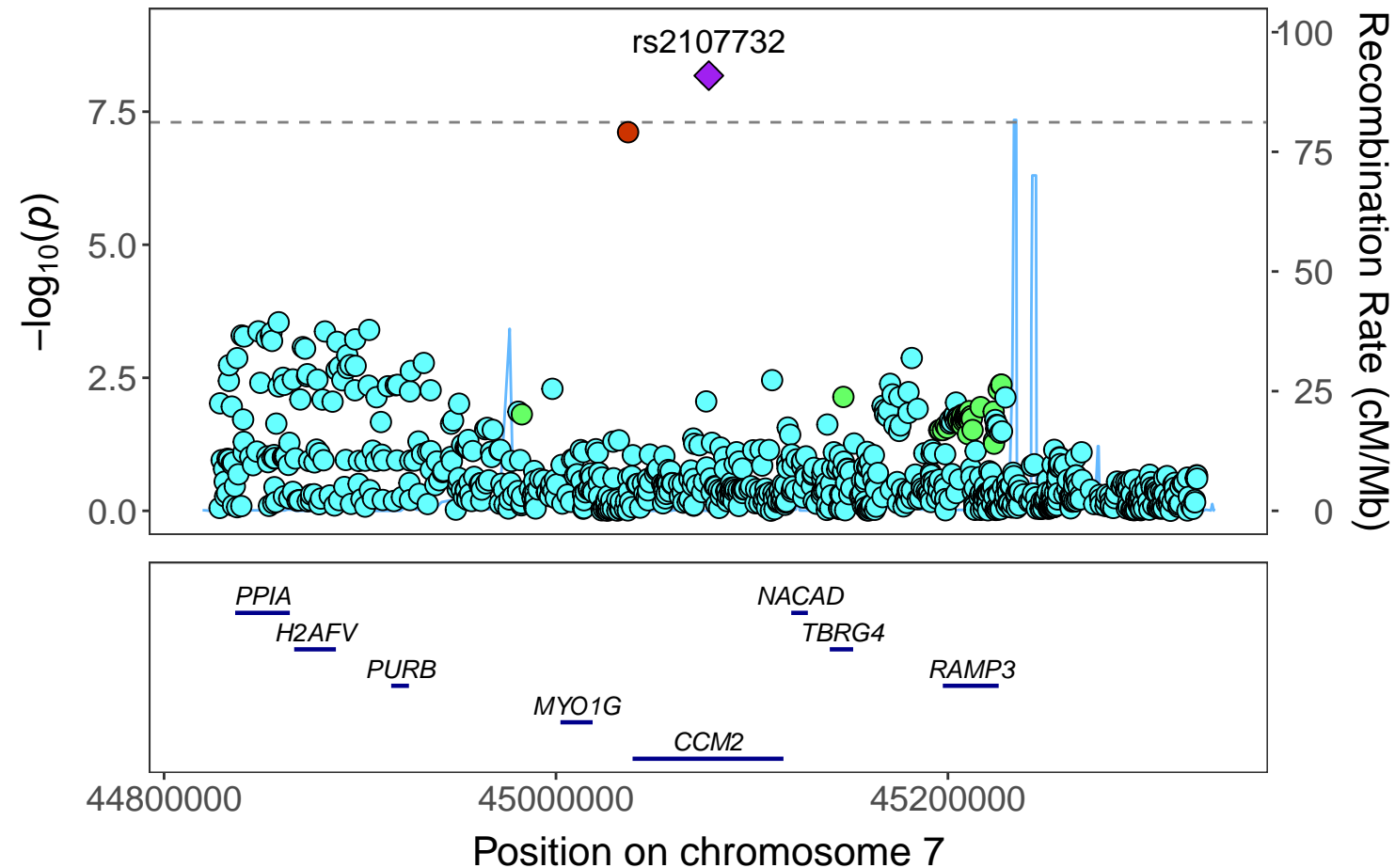

Supplement: Supplementary file 5 — Supporting Information [file CTM2-16-e70732-s001.zip › LocusZoom/Sfig_rs2107732_locusZoom.pdf]

# *LocusZoom plots of GWAS top lead SNP*

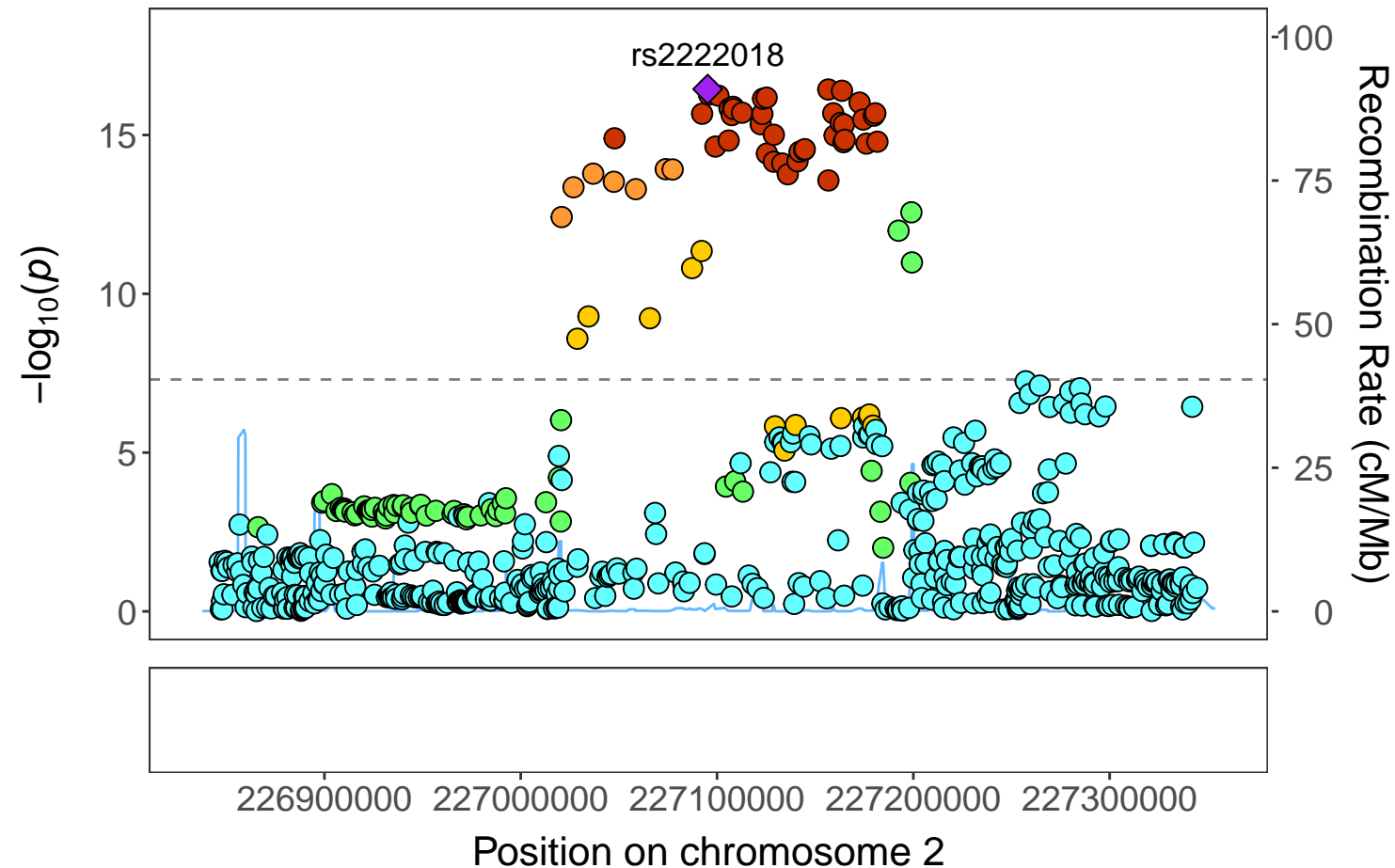

Supplement: Supplementary file 5 — Supporting Information [file CTM2-16-e70732-s001.zip › LocusZoom/Sfig_rs2222018_locusZoom.pdf]

# LocusZoom plots of GWAS top lead SNP

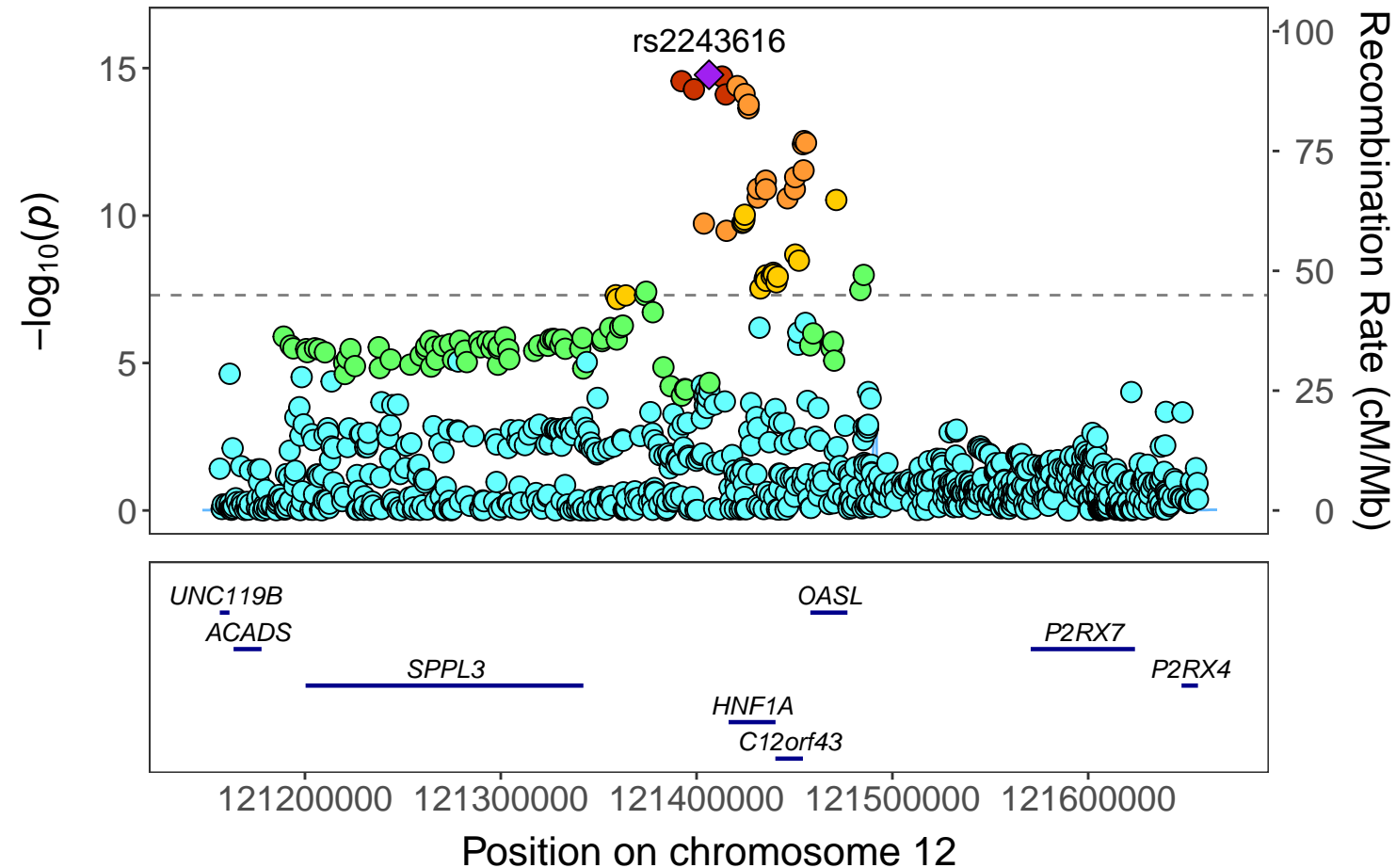

r2   miss   0.0–0.2   0.2–0.4   0.4–0.6   0.6–0.8   0.8–1.0

Supplement: Supplementary file 5 — Supporting Information [file CTM2-16-e70732-s001.zip › LocusZoom/Sfig_rs2243616_locusZoom.pdf]

# LocusZoom plots of GWAS top lead SNP

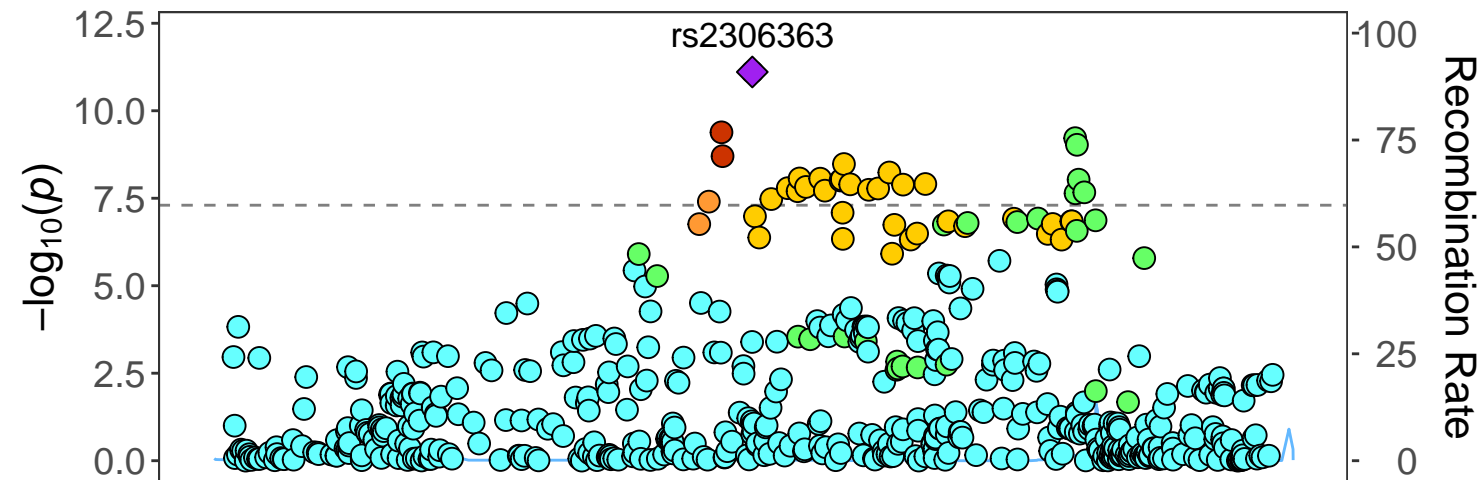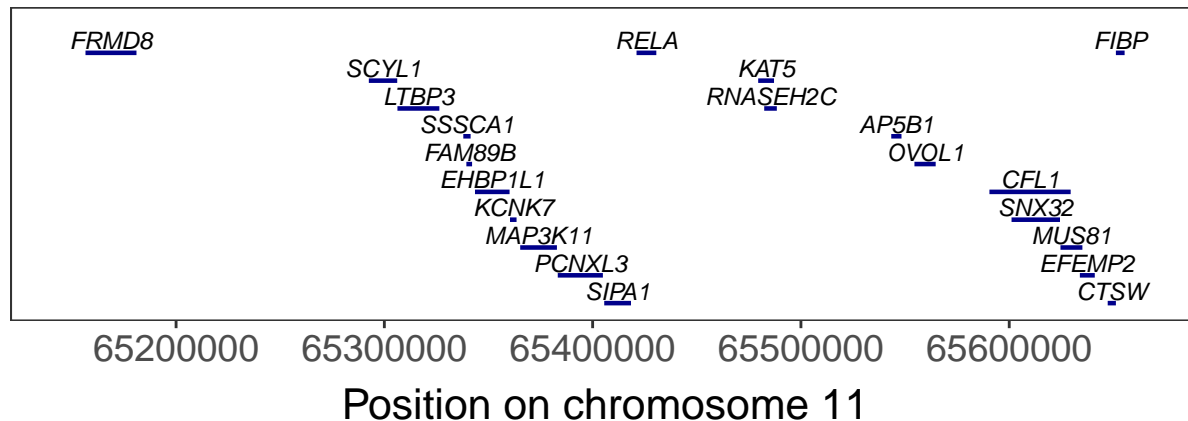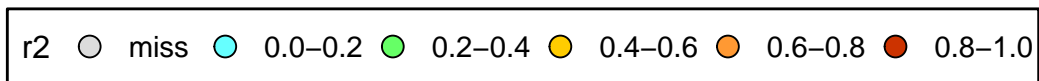

Supplement: Supplementary file 5 — Supporting Information [file CTM2-16-e70732-s001.zip › LocusZoom/Sfig_rs2306363_locusZoom.pdf]

# LocusZoom plots of GWAS top lead SNP

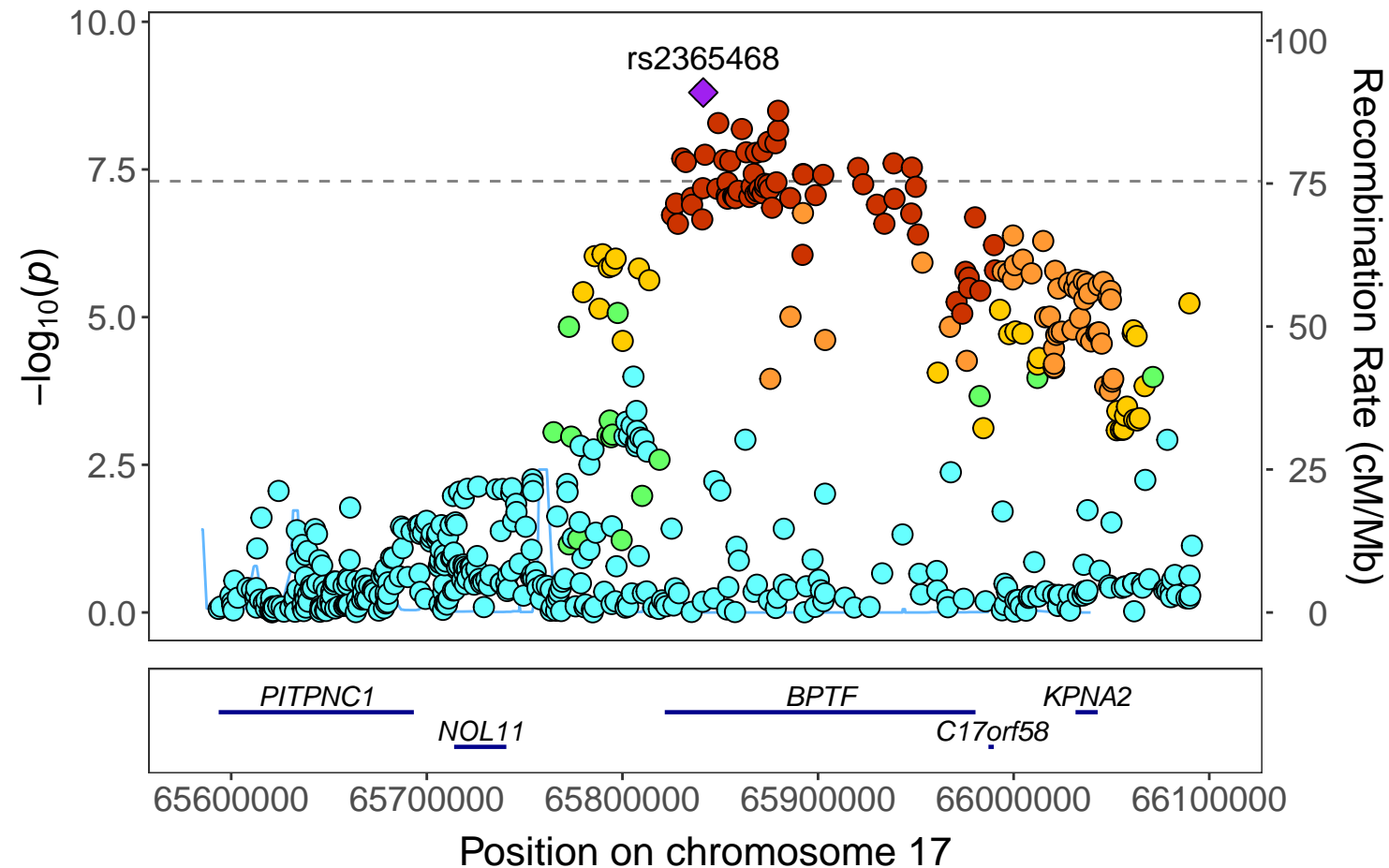

$r^2$  ○ miss ○ 0.0-0.2 ○ 0.2-0.4 ○ 0.4-0.6 ○ 0.6-0.8 ○ 0.8-1.0

Supplement: Supplementary file 5 — Supporting Information [file CTM2-16-e70732-s001.zip › LocusZoom/Sfig_rs2365468_locusZoom.pdf]

# LocusZoom plots of GWAS top lead SNP

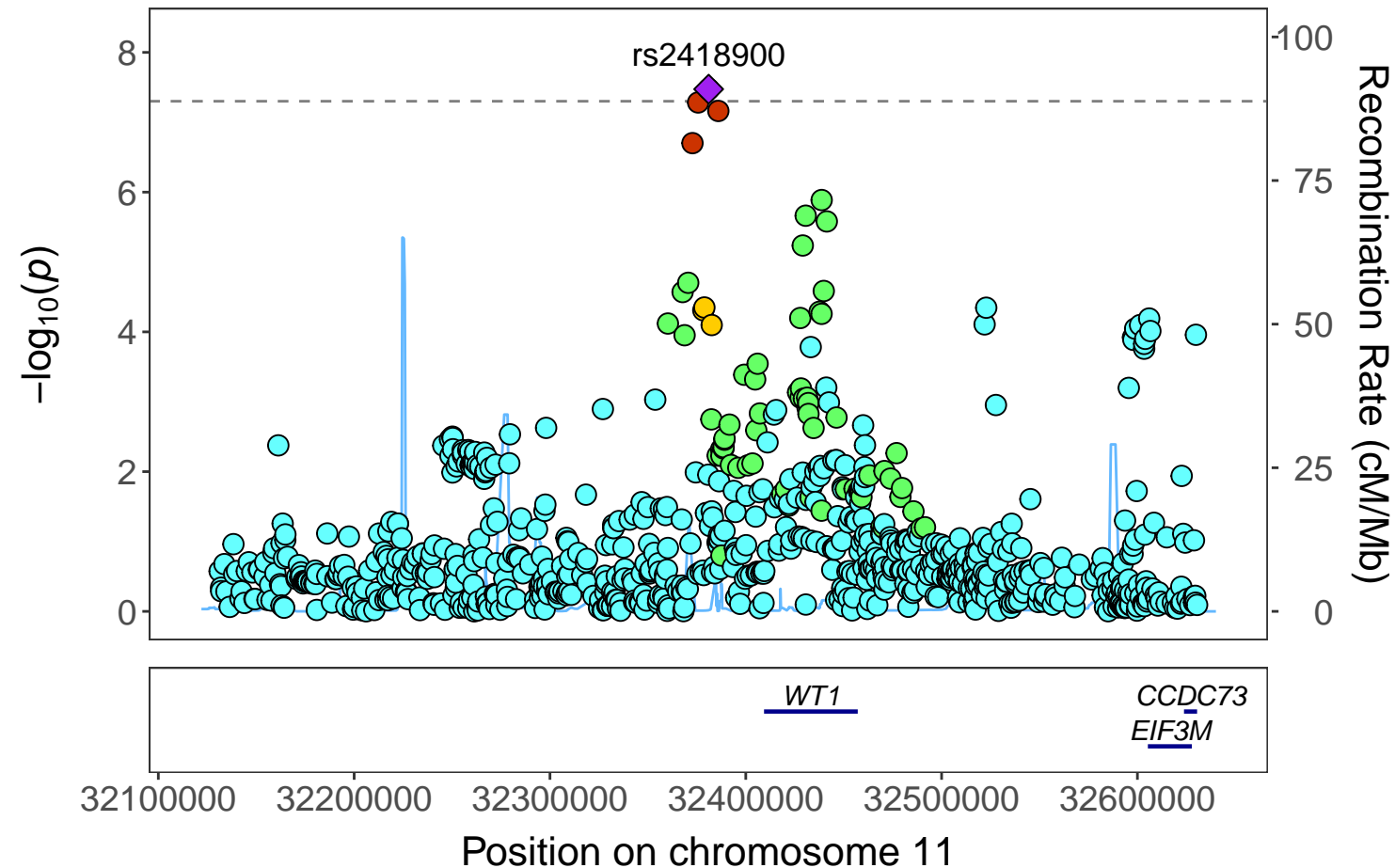

$r^2$    miss   0.0–0.2   0.2–0.4   0.4–0.6   0.6–0.8   0.8–1.0

Supplement: Supplementary file 5 — Supporting Information [file CTM2-16-e70732-s001.zip › LocusZoom/Sfig_rs2418900_locusZoom.pdf]

# LocusZoom plots of GWAS top lead SNP

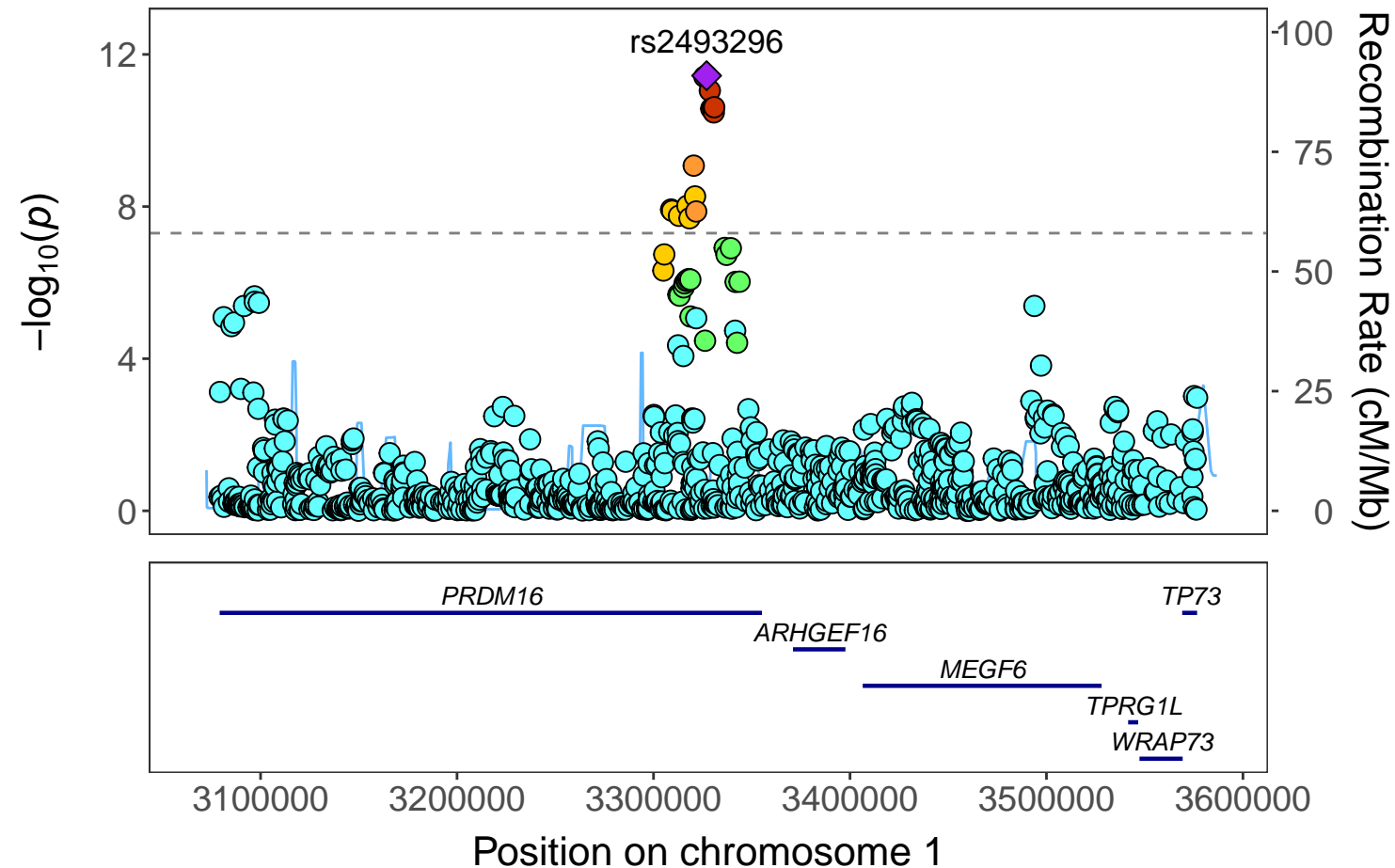

Supplement: Supplementary file 5 — Supporting Information [file CTM2-16-e70732-s001.zip › LocusZoom/Sfig_rs2493296_locusZoom.pdf]

# LocusZoom plots of GWAS top lead SNP

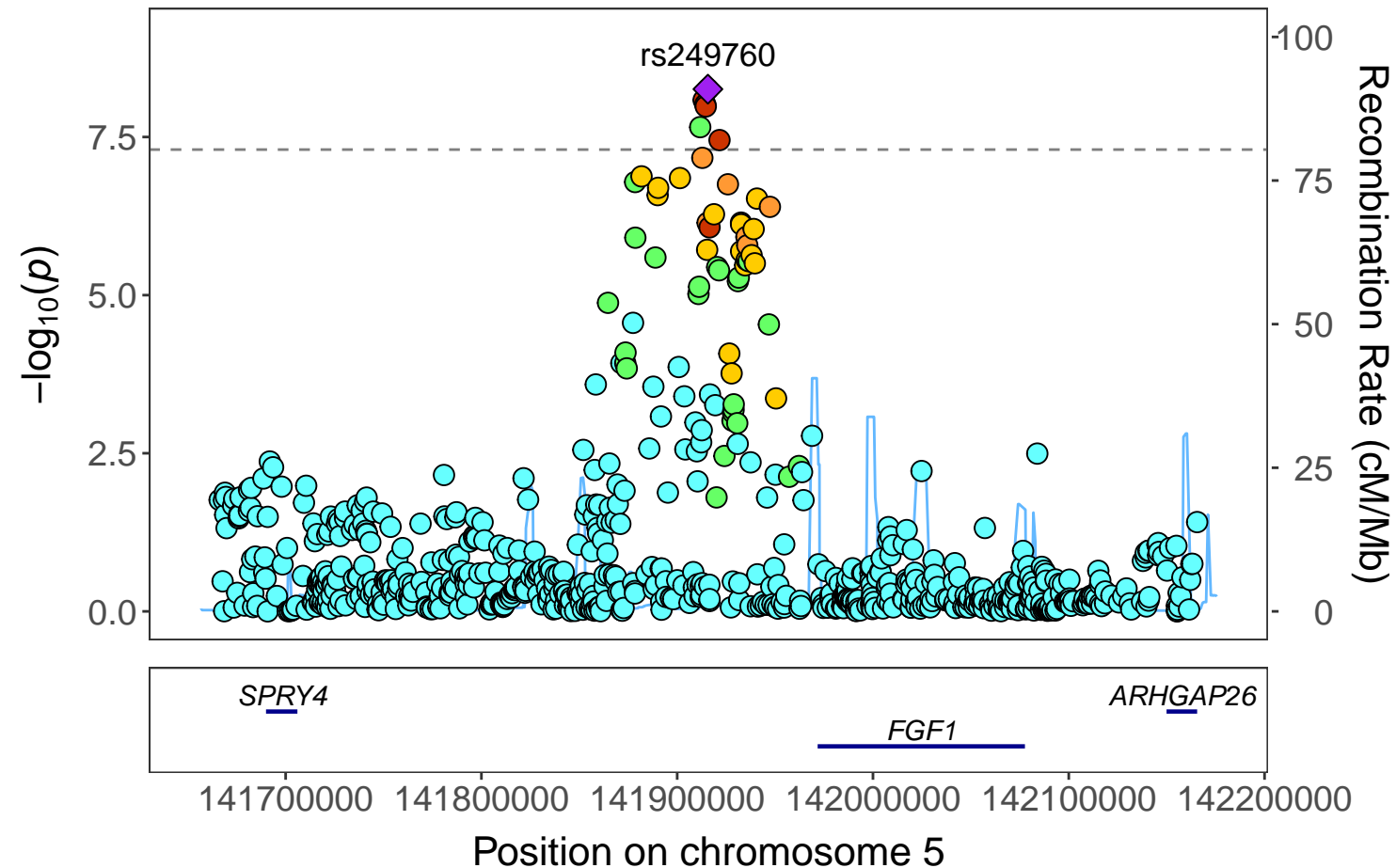

$r^2$    miss   0.0-0.2   0.2-0.4   0.4-0.6   0.6-0.8   0.8-1.0

Supplement: Supplementary file 5 — Supporting Information [file CTM2-16-e70732-s001.zip › LocusZoom/Sfig_rs249760_locusZoom.pdf]

# LocusZoom plots of GWAS top lead SNP

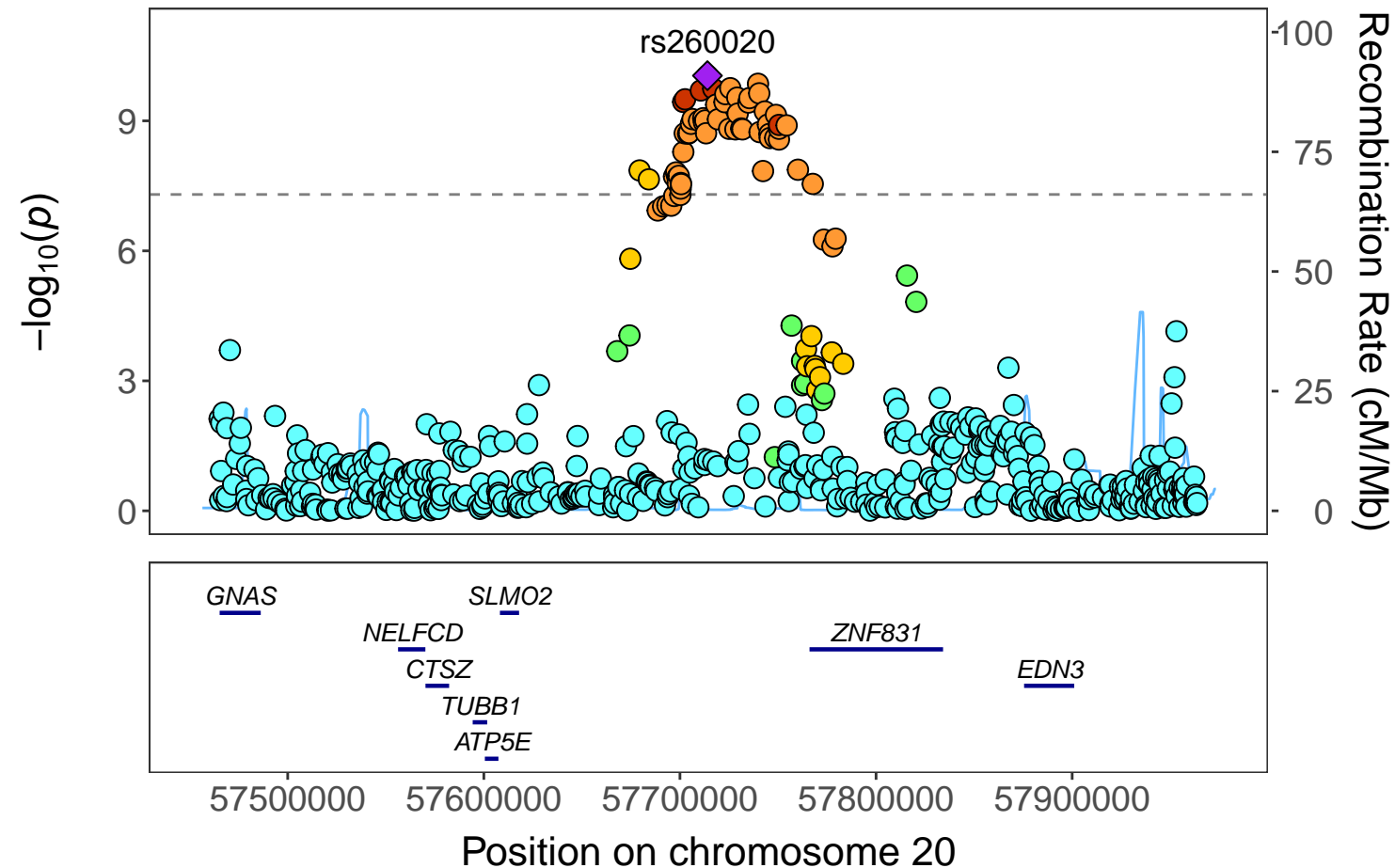

r2    miss    0.0–0.2    0.2–0.4    0.4–0.6    0.6–0.8    0.8–1.0

Supplement: Supplementary file 5 — Supporting Information [file CTM2-16-e70732-s001.zip › LocusZoom/Sfig_rs260020_locusZoom.pdf]

# LocusZoom plots of GWAS top lead SNP

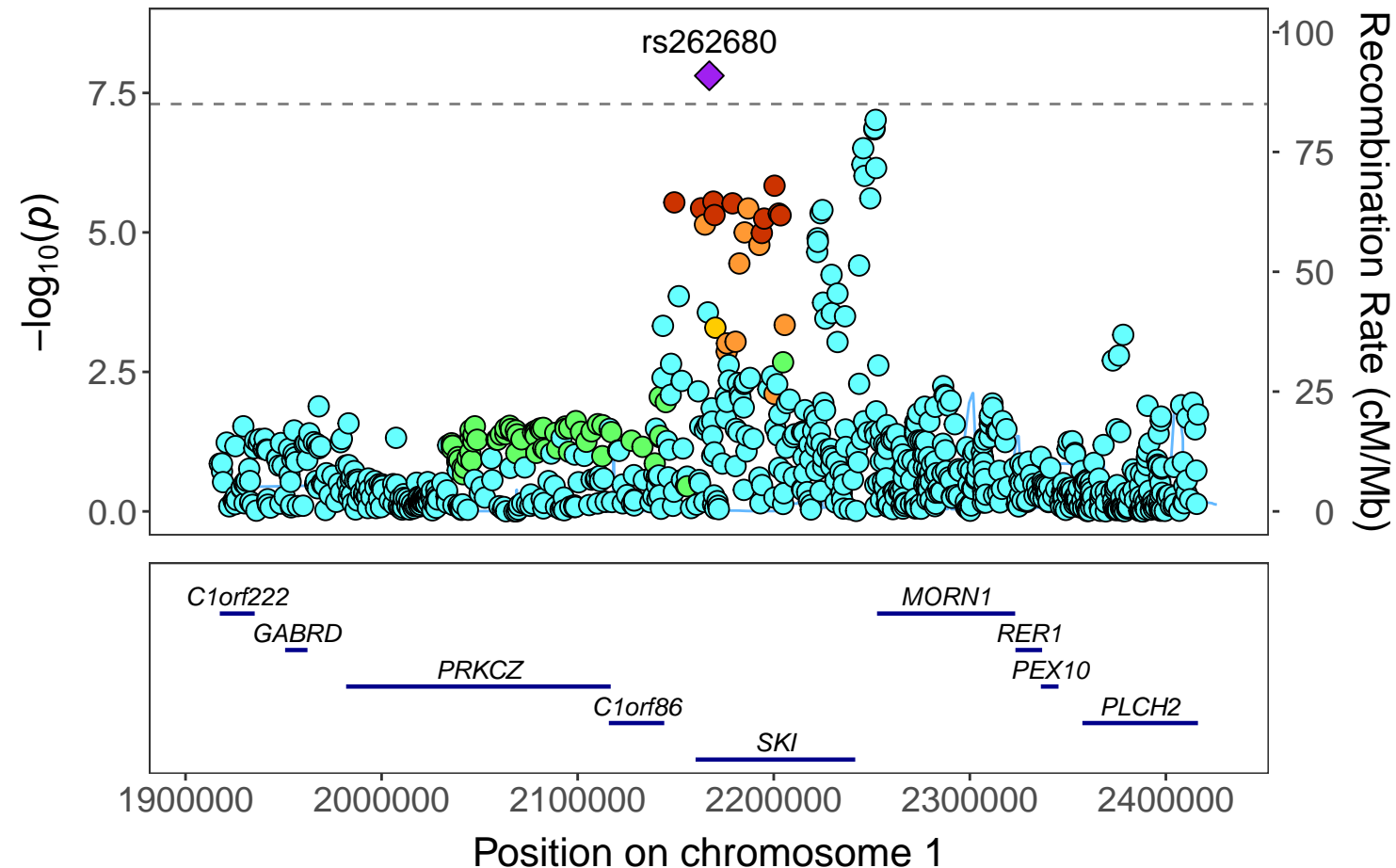

Supplement: Supplementary file 5 — Supporting Information [file CTM2-16-e70732-s001.zip › LocusZoom/Sfig_rs262680_locusZoom.pdf]

# *LocusZoom plots of GWAS top lead SNP*

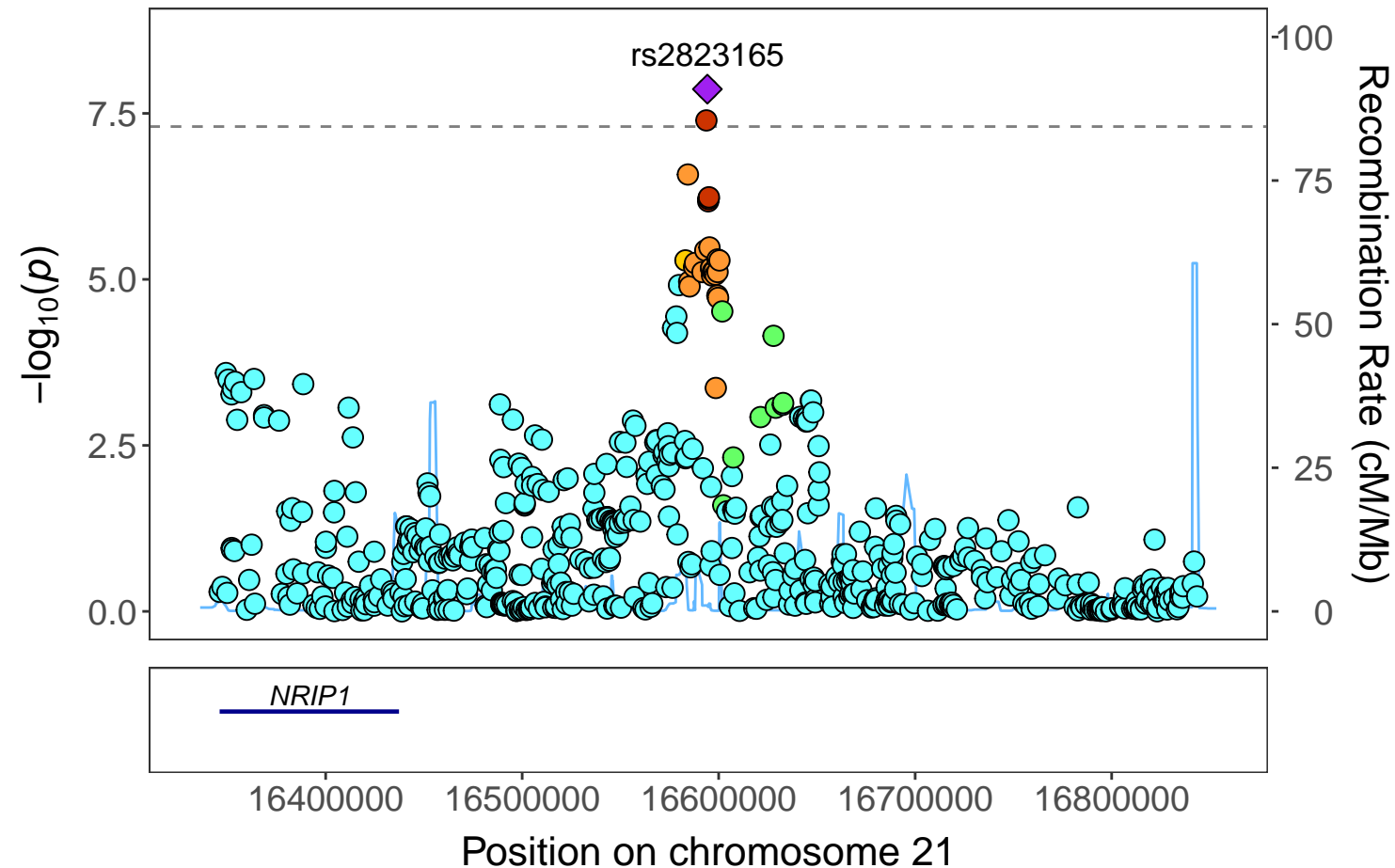

r2    miss    0.0–0.2    0.2–0.4    0.4–0.6    0.6–0.8    0.8–1.0

Supplement: Supplementary file 5 — Supporting Information [file CTM2-16-e70732-s001.zip › LocusZoom/Sfig_rs2823165_locusZoom.pdf]

# LocusZoom plots of GWAS top lead SNP

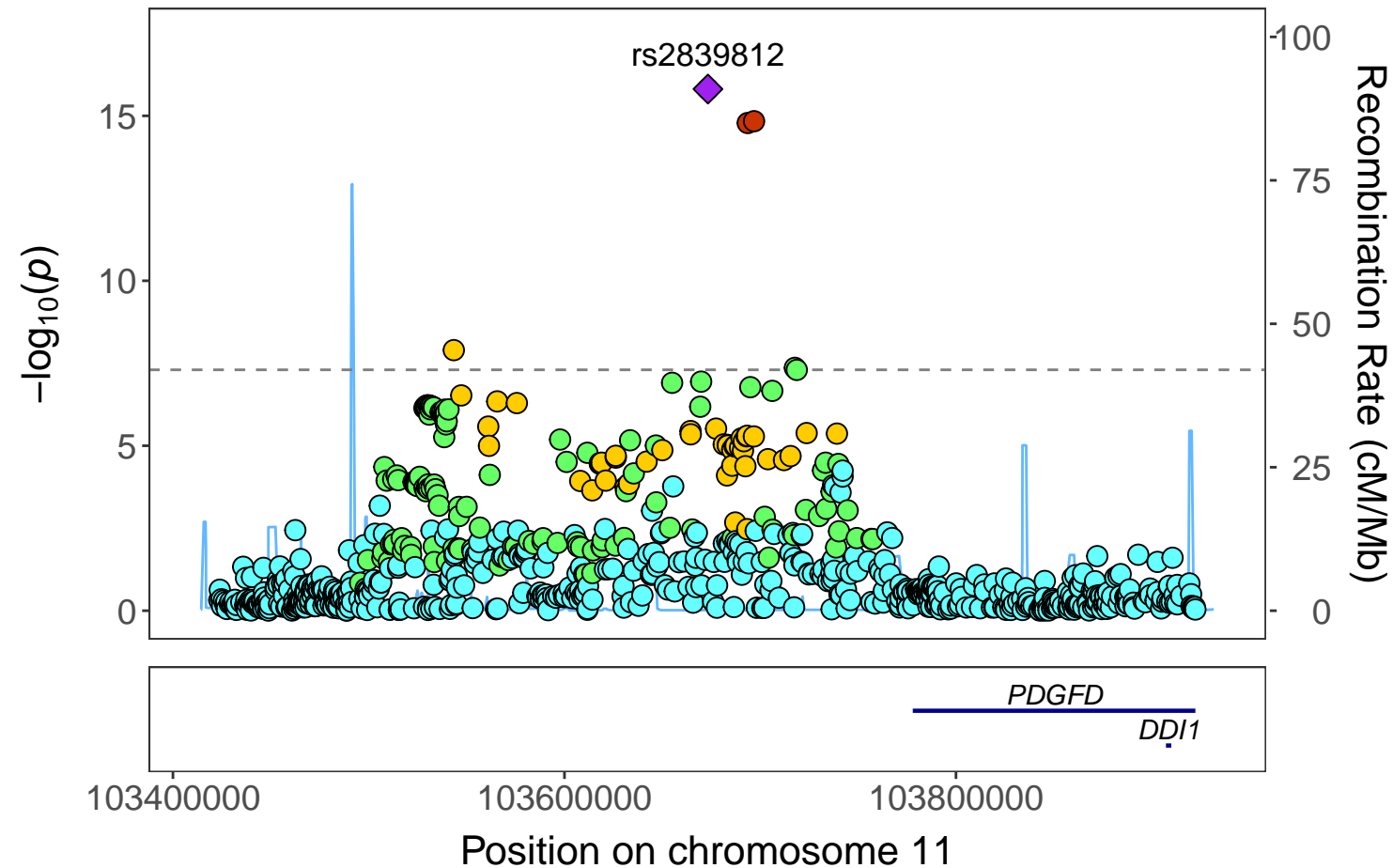

$r^2$    miss   0.0–0.2   0.2–0.4   0.4–0.6   0.6–0.8   0.8–1.0

Supplement: Supplementary file 5 — Supporting Information [file CTM2-16-e70732-s001.zip › LocusZoom/Sfig_rs2839812_locusZoom.pdf]

# LocusZoom plots of GWAS top lead SNP

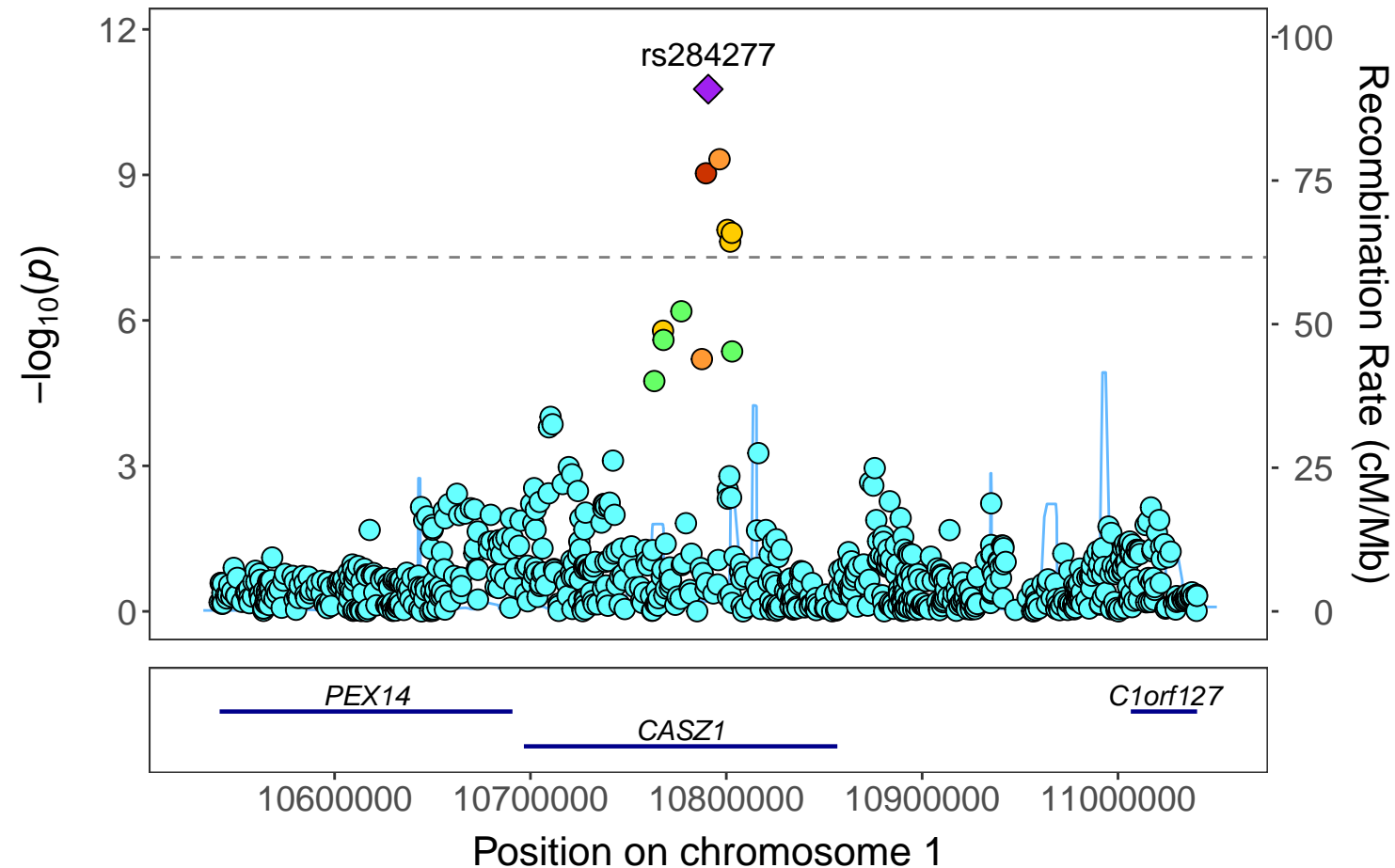

$r^2$     $\circ$  miss    $\circ$  0.0–0.2    $\circ$  0.2–0.4    $\circ$  0.4–0.6    $\circ$  0.6–0.8    $\circ$  0.8–1.0

Supplement: Supplementary file 5 — Supporting Information [file CTM2-16-e70732-s001.zip › LocusZoom/Sfig_rs284277_locusZoom.pdf]

# LocusZoom plots of GWAS top lead SNP

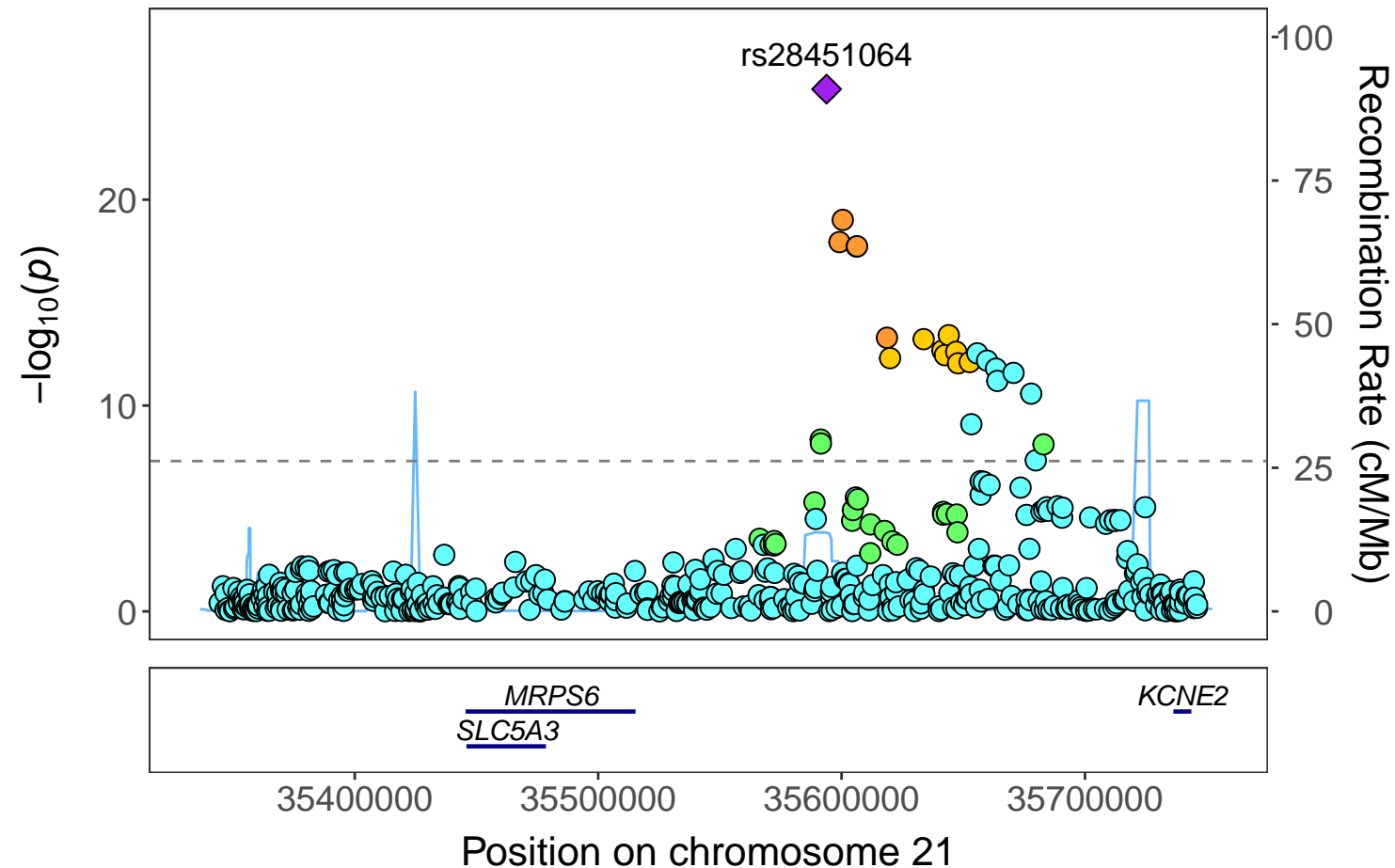

$r^2$    miss   0.0–0.2   0.2–0.4   0.4–0.6   0.6–0.8   0.8–1.0

Supplement: Supplementary file 5 — Supporting Information [file CTM2-16-e70732-s001.zip › LocusZoom/Sfig_rs28451064_locusZoom.pdf]

# LocusZoom plots of GWAS top lead SNP

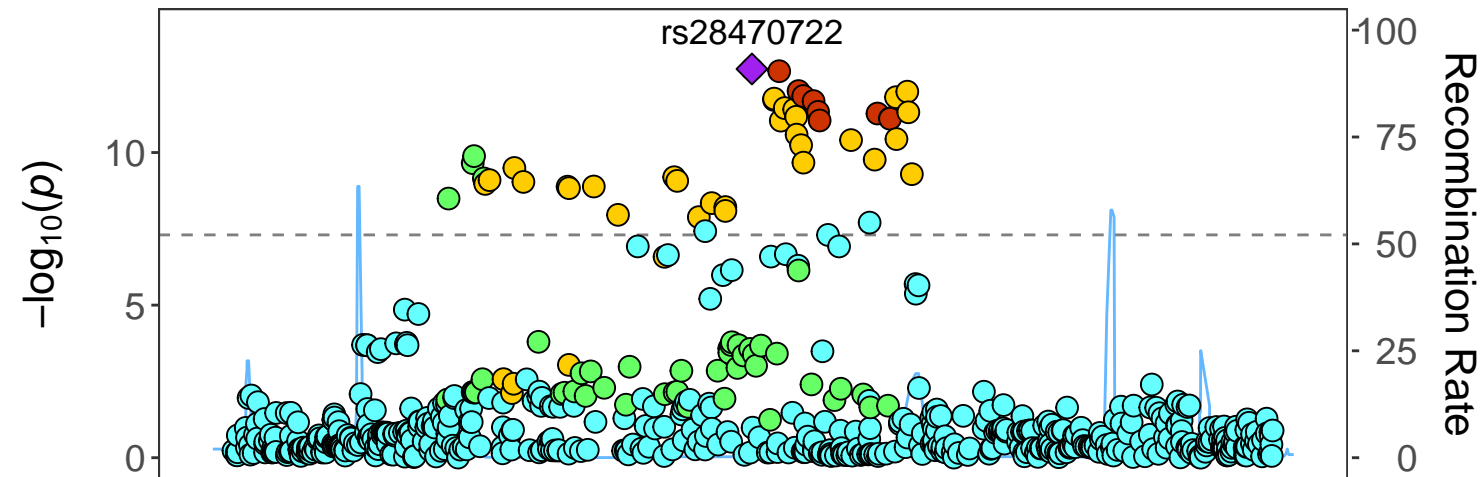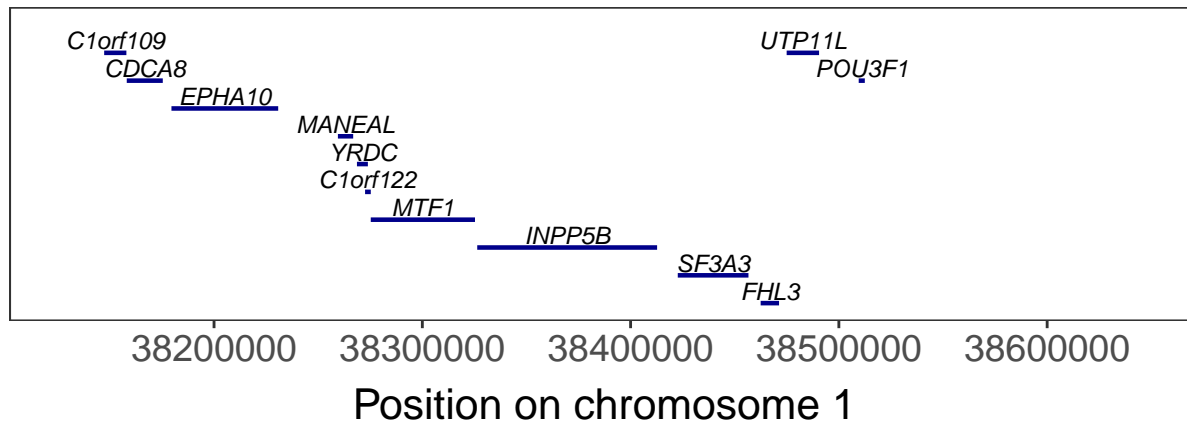

r2    miss    0.0–0.2    0.2–0.4    0.4–0.6    0.6–0.8    0.8–1.0

Supplement: Supplementary file 5 — Supporting Information [file CTM2-16-e70732-s001.zip › LocusZoom/Sfig_rs28470722_locusZoom.pdf]
