# Supplementary material for: Precision medicine for atherosclerotic cardiovascular disease: Integrative genomics maps risk loci and AI‐predicted functional consequences
Source: Clin Transl Med. 2026 Jul 10;16(7):e70732. doi: 10.1002/ctm2.70732 (PMC13351343; doi:10.1002/ctm2.70732)
Supplement: Supplementary file 5 — Supporting Information [file CTM2-16-e70732-s001.zip › LocusZoom/Sfig_rs7034361_locusZoom.pdf]

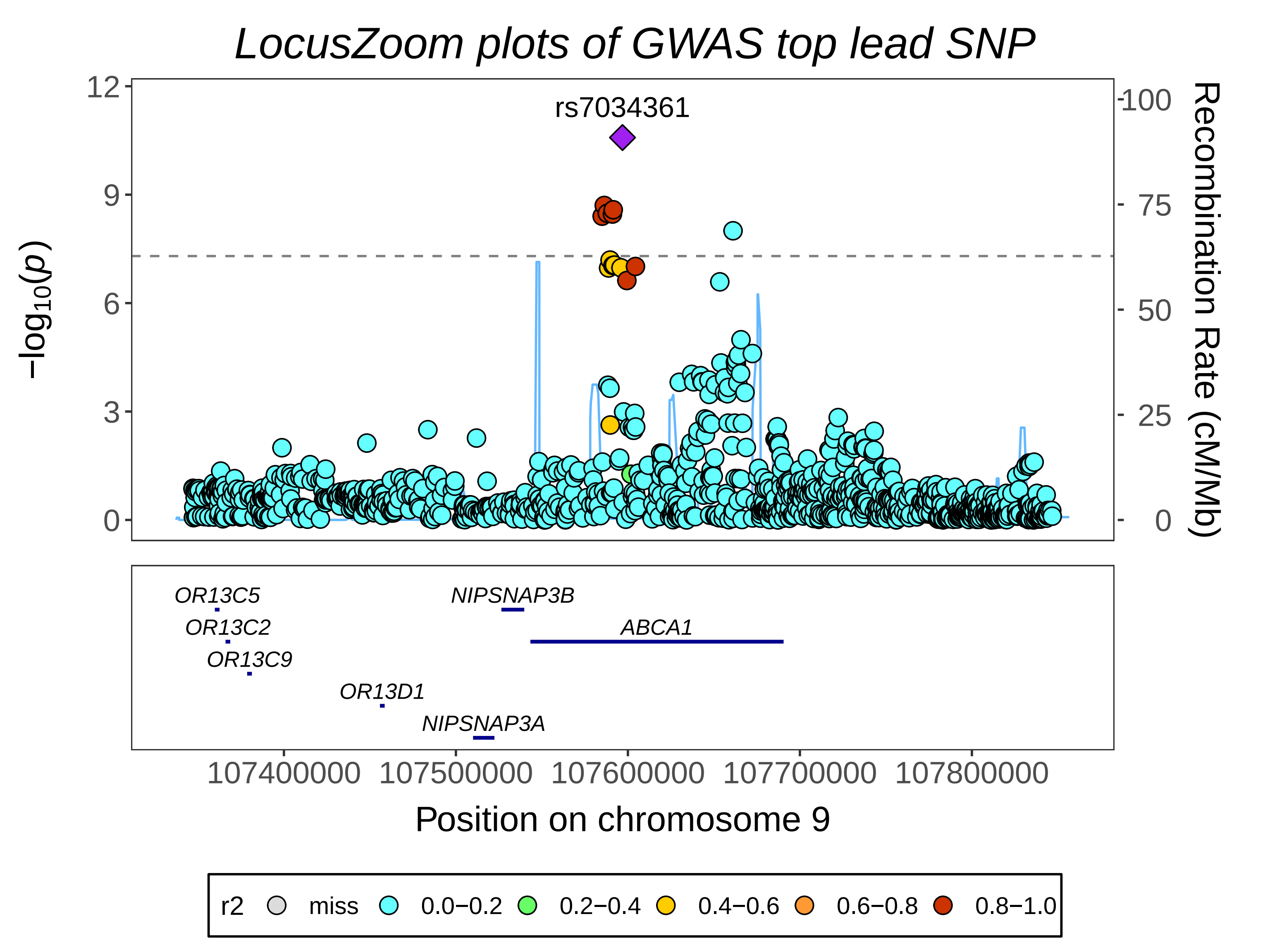

LocusZoom plots of GWAS top lead SNP
rs7034361
12
9
6
3
0
−log10(p)
100
75
50
25
0
Recombination Rate (cM/Mb)
OR13C5
OR13C2
OR13C9
OR13D1
NIPSNAP3A
NIPSNAP3B
ABCA1
107400000
107500000
107600000
107700000
107800000
Position on chromosome 9
r2
miss
0.0−0.2
0.2−0.4
0.4−0.6
0.6−0.8
0.8−1.0
