# Supplementary figures and images for: Precision medicine for atherosclerotic cardiovascular disease: Integrative genomics maps risk loci and AI‐predicted functional consequences (part 3 of 3)
Source: Clin Transl Med. 2026 Jul 10;16(7):e70732. doi: 10.1002/ctm2.70732 (PMC13351343; doi:10.1002/ctm2.70732)

# LocusZoom plots of GWAS top lead SNP

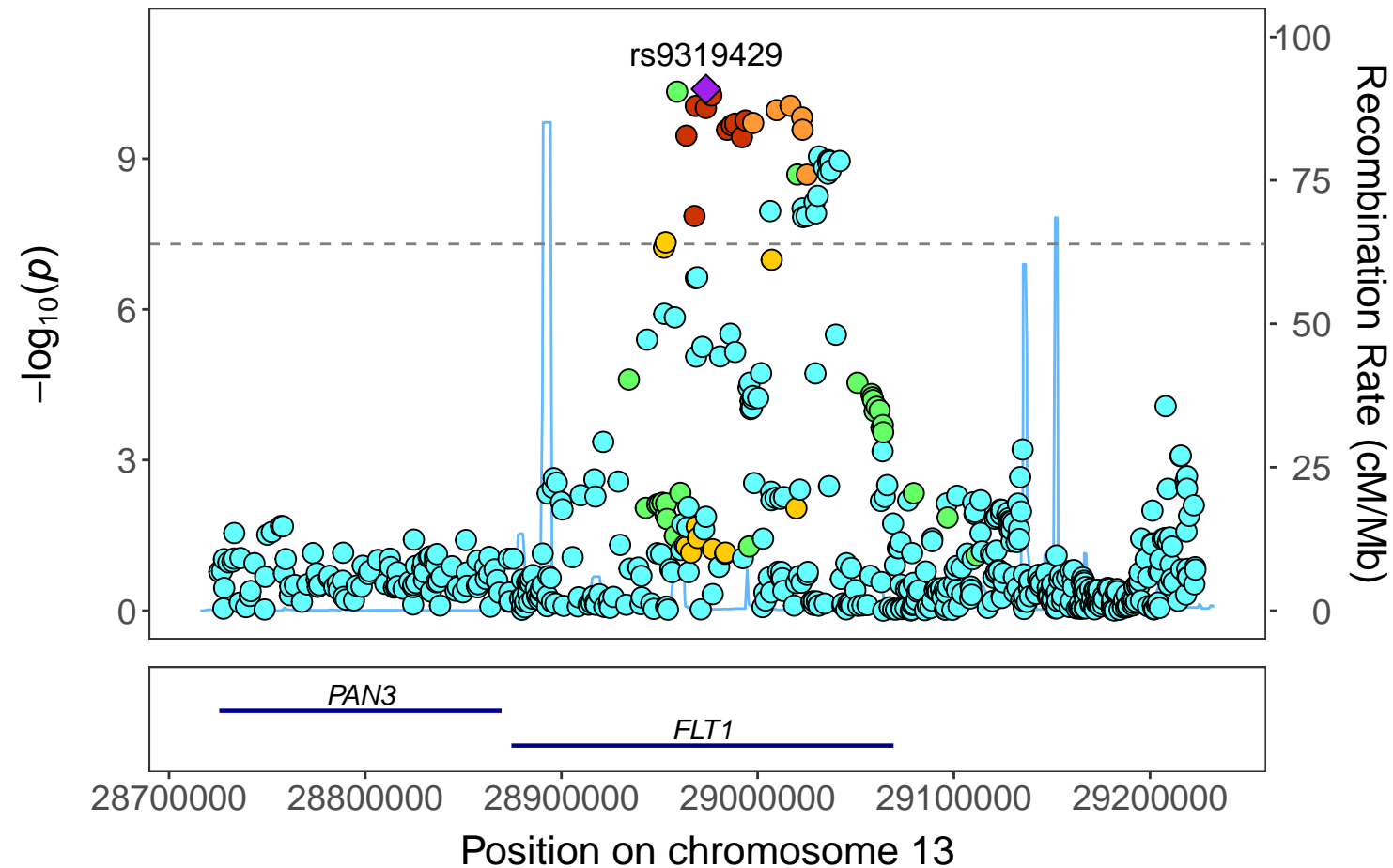

$r^2$    miss   0.0-0.2   0.2-0.4   0.4-0.6   0.6-0.8   0.8-1.0

Supplement: Supplementary file 5 — Supporting Information [file CTM2-16-e70732-s001.zip › LocusZoom/Sfig_rs9319429_locusZoom.pdf]

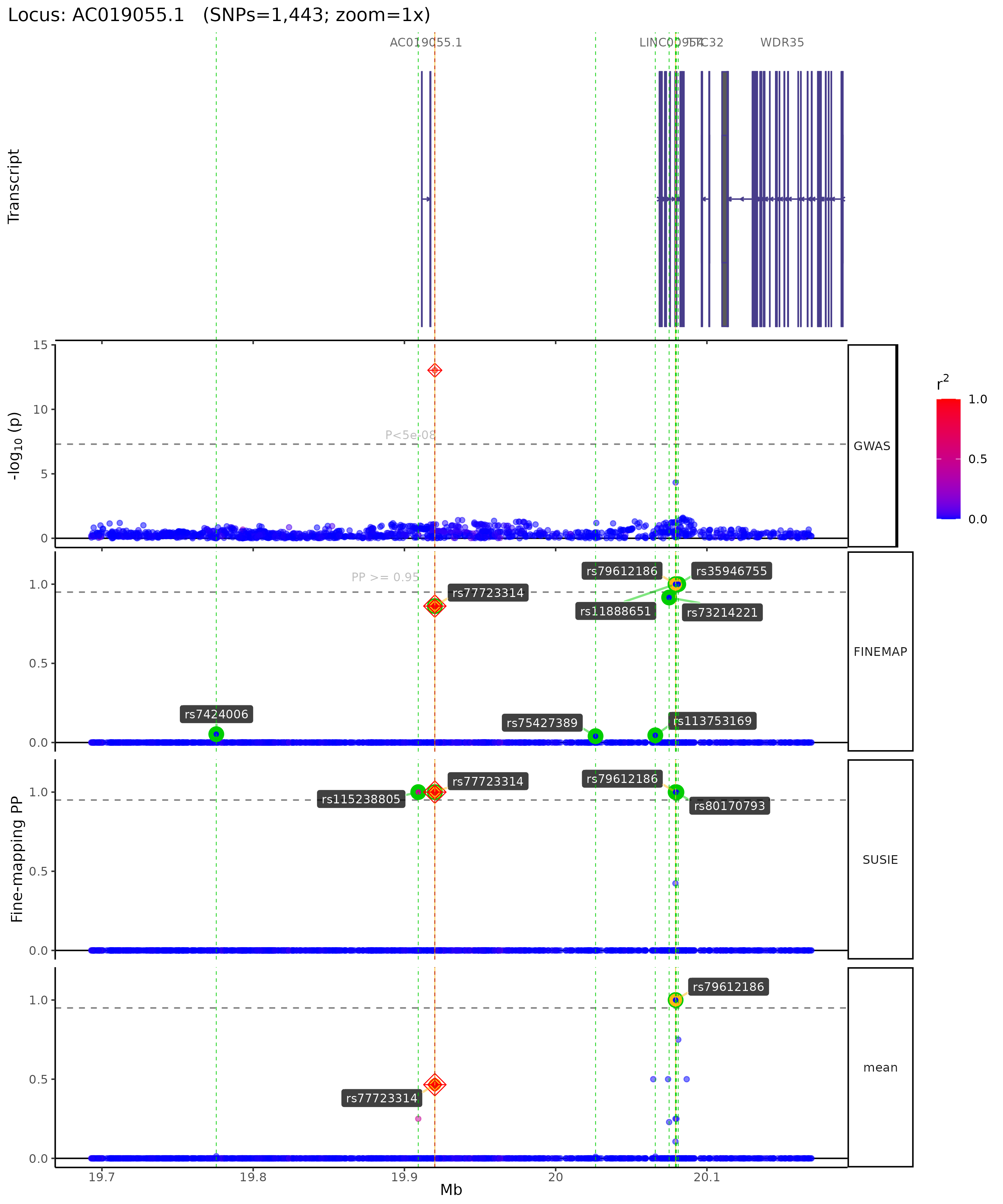

Supplement: Supplementary file 6 — Supporting Information [file CTM2-16-e70732-s005.zip › AC019055.1/multiview.AC019055.1.1KGphase3.1x.png]

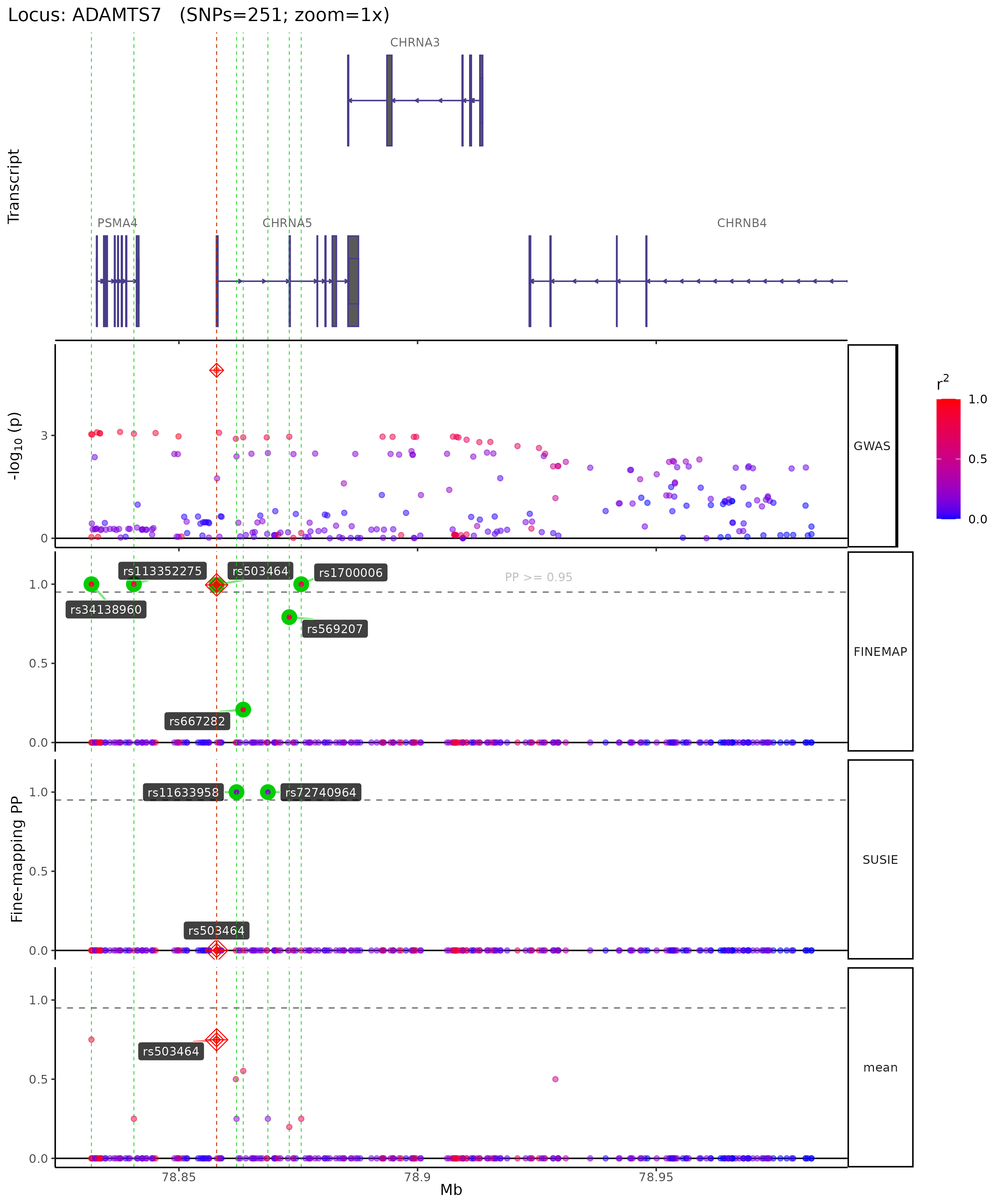

Supplement: Supplementary file 6 — Supporting Information [file CTM2-16-e70732-s005.zip › ADAMTS7/multiview.ADAMTS7.1KGphase3.1x.png]

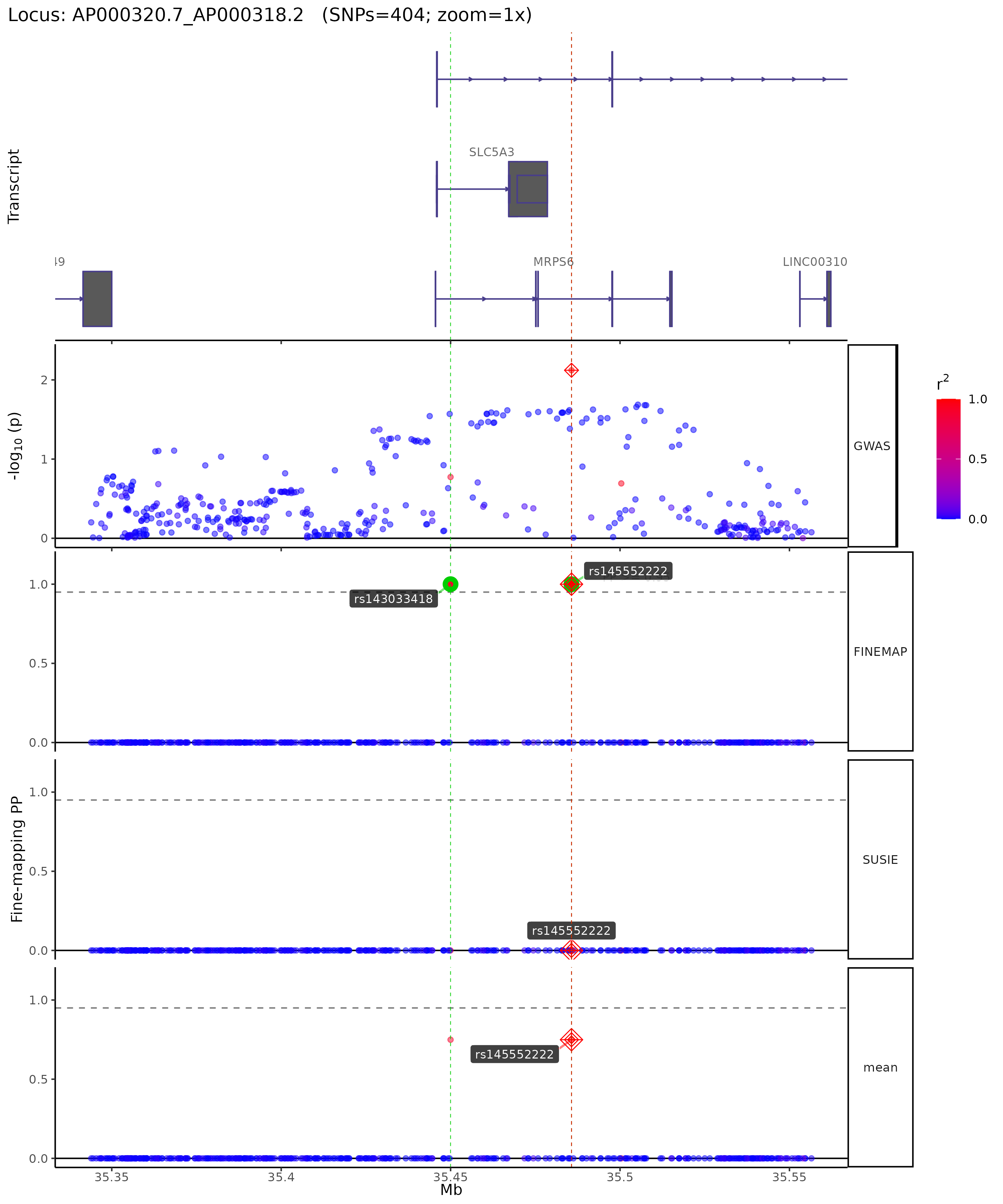

Supplement: Supplementary file 6 — Supporting Information [file CTM2-16-e70732-s005.zip › AP000320.7_AP000318.2/multiview.AP000320.7_AP000318.2.1KGphase3.1x.png]

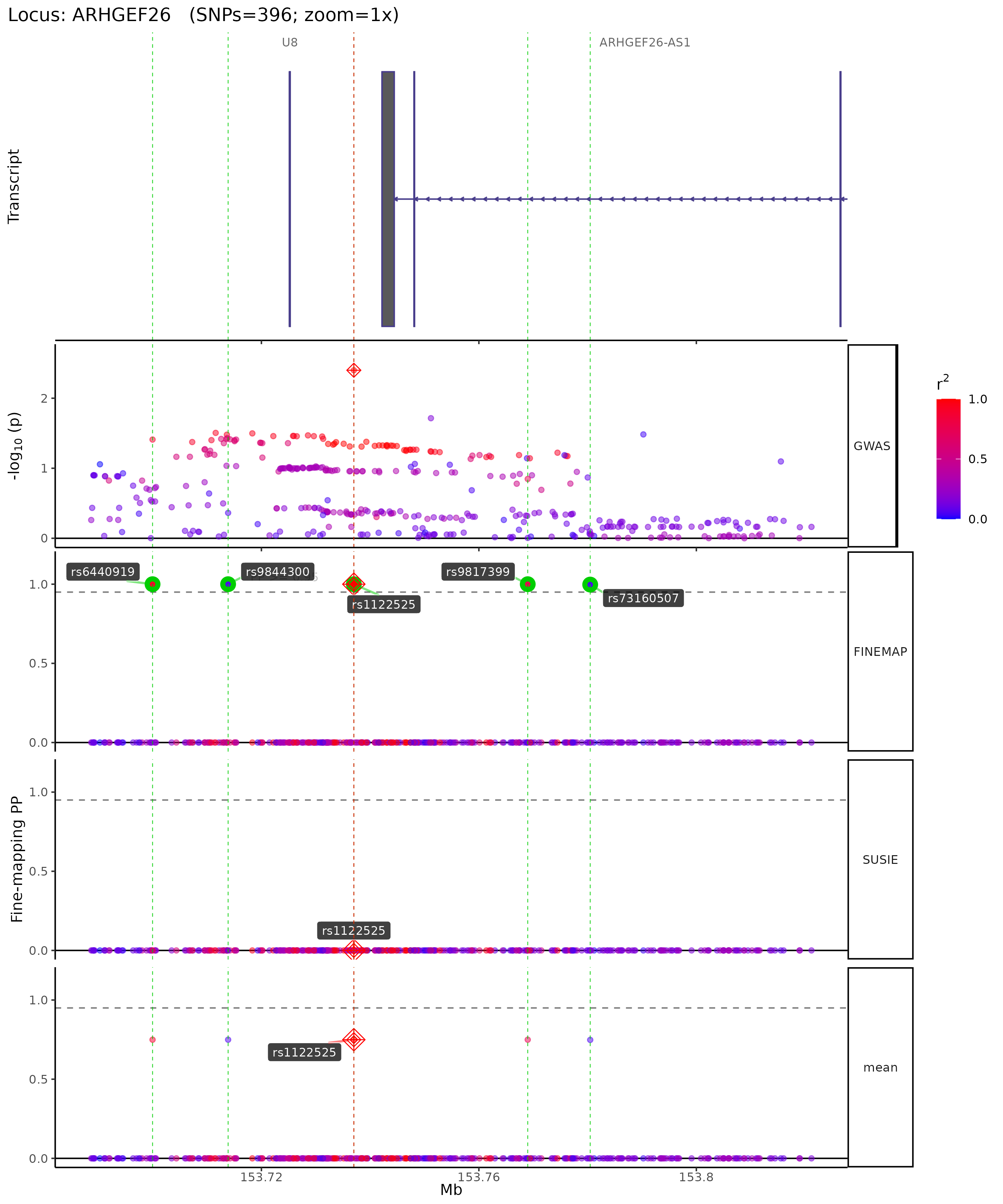

Supplement: Supplementary file 6 — Supporting Information [file CTM2-16-e70732-s005.zip › ARHGEF26/multiview.ARHGEF26.1KGphase3.1x.png]

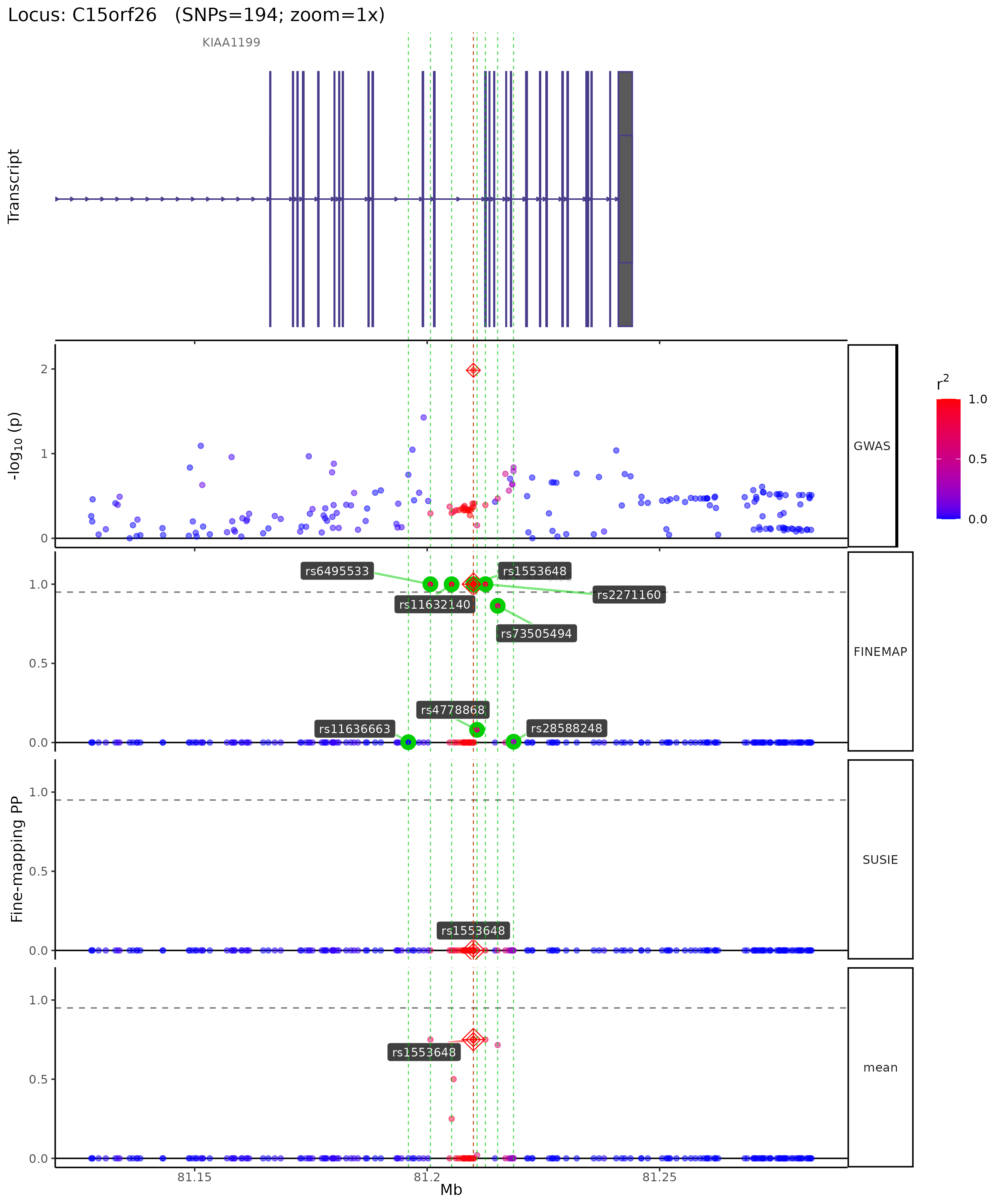

Supplement: Supplementary file 6 — Supporting Information [file CTM2-16-e70732-s005.zip › C15orf26/multiview.C15orf26.1KGphase3.1x.png]

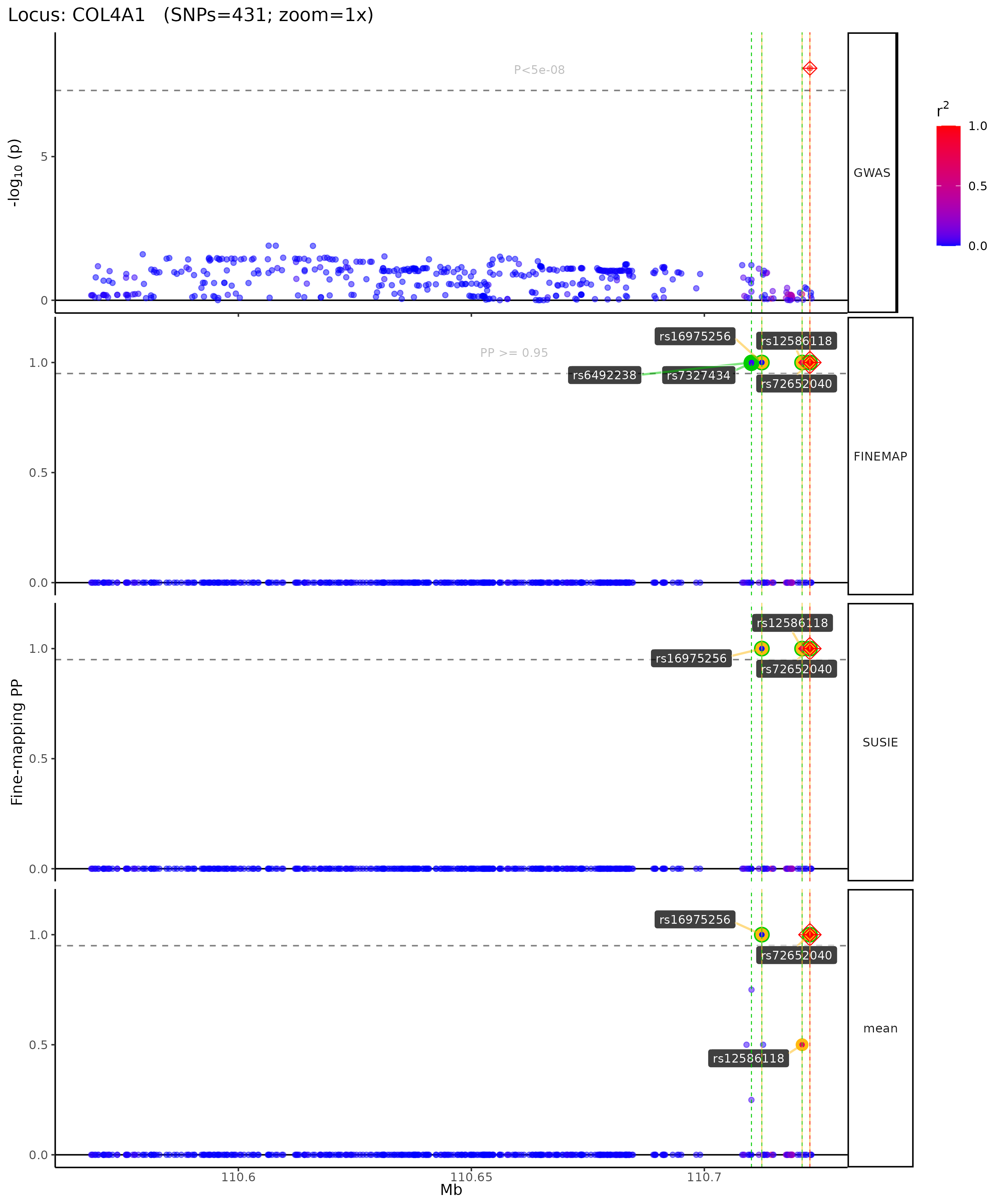

Supplement: Supplementary file 6 — Supporting Information [file CTM2-16-e70732-s005.zip › COL4A1/multiview.COL4A1.1KGphase3.1x.png]

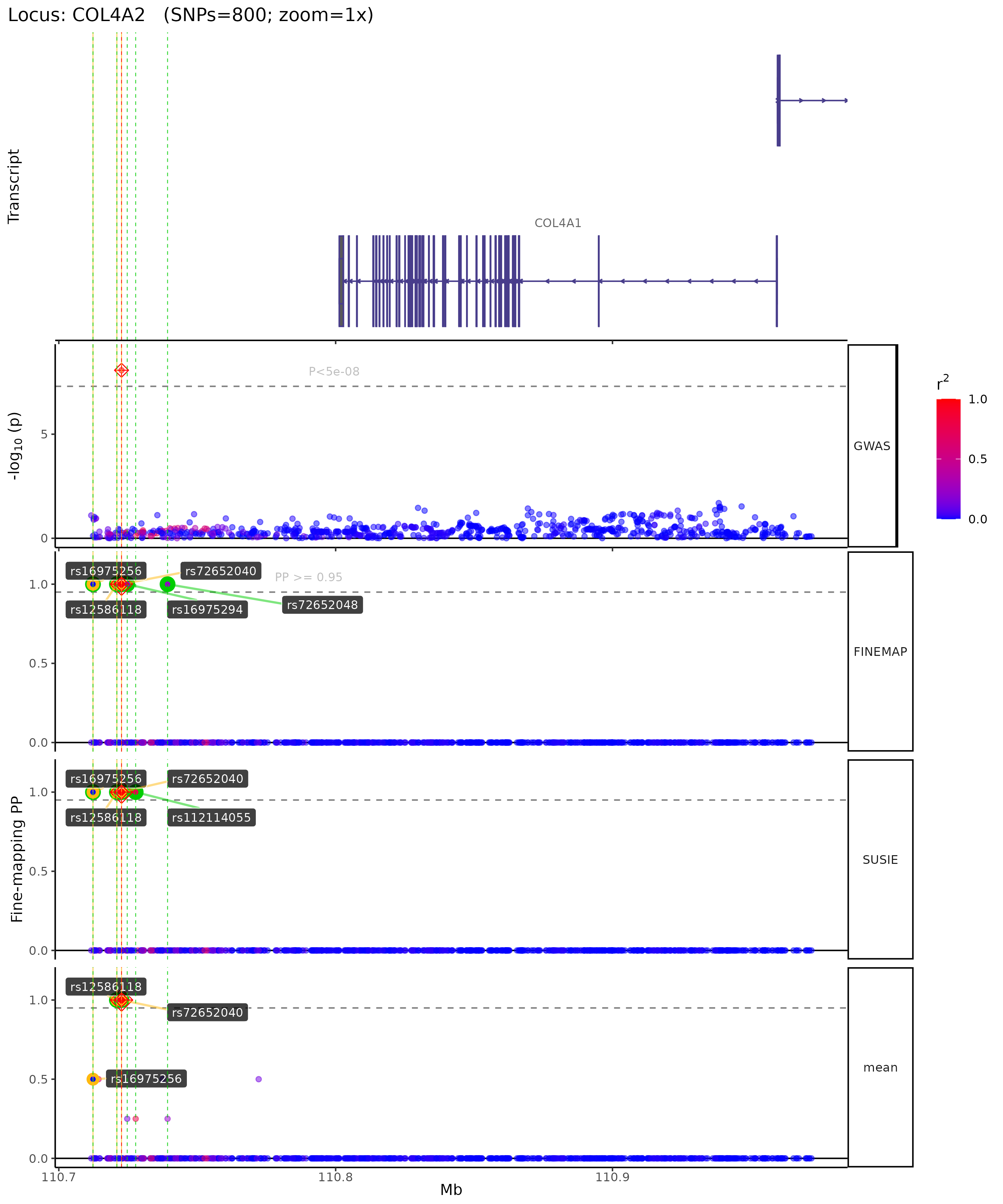

Supplement: Supplementary file 6 — Supporting Information [file CTM2-16-e70732-s005.zip › COL4A2/multiview.COL4A2.1KGphase3.1x.png]

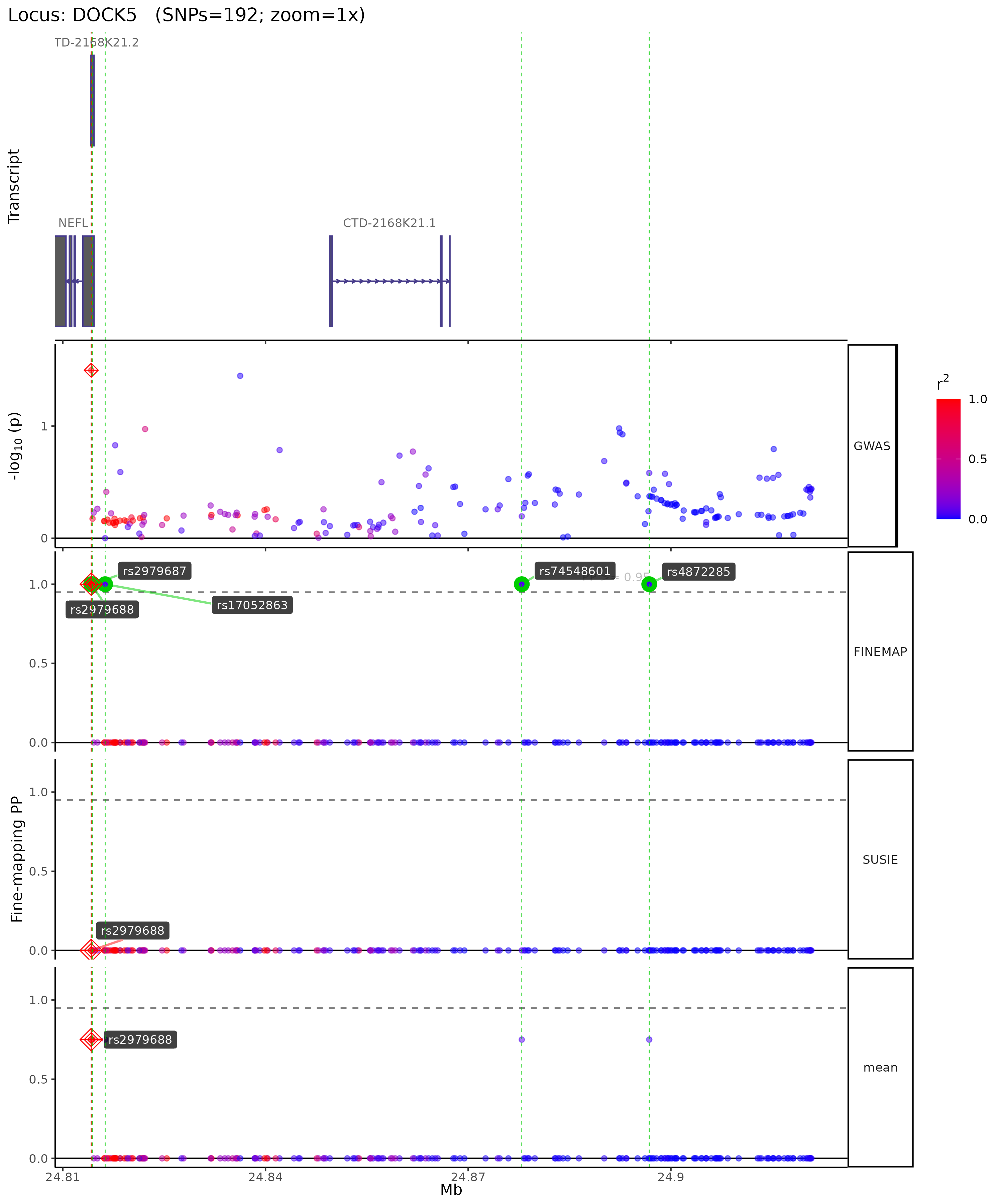

Supplement: Supplementary file 6 — Supporting Information [file CTM2-16-e70732-s005.zip › DOCK5/multiview.DOCK5.1KGphase3.1x.png]

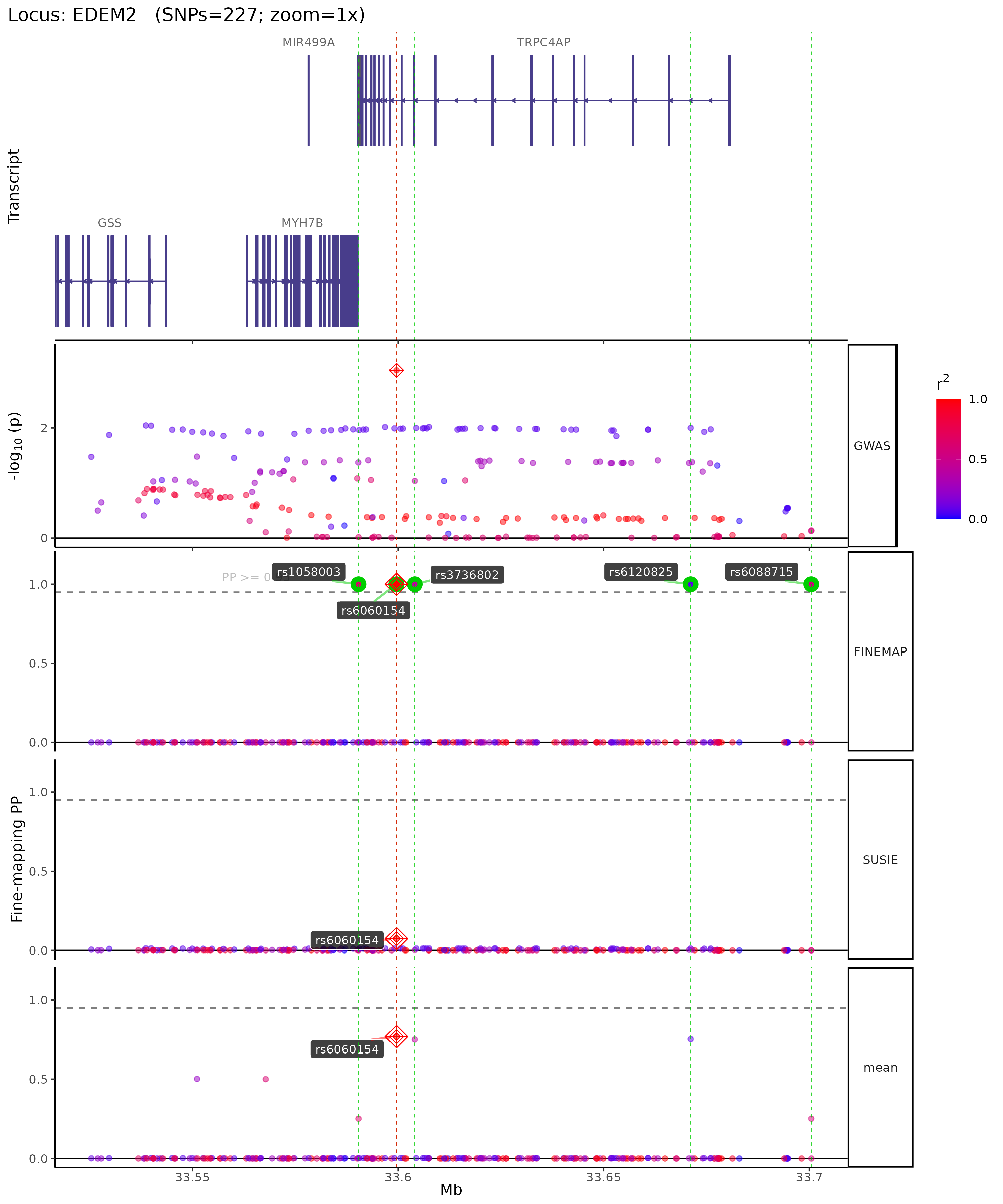

Supplement: Supplementary file 6 — Supporting Information [file CTM2-16-e70732-s005.zip › EDEM2/multiview.EDEM2.1KGphase3.1x.png]

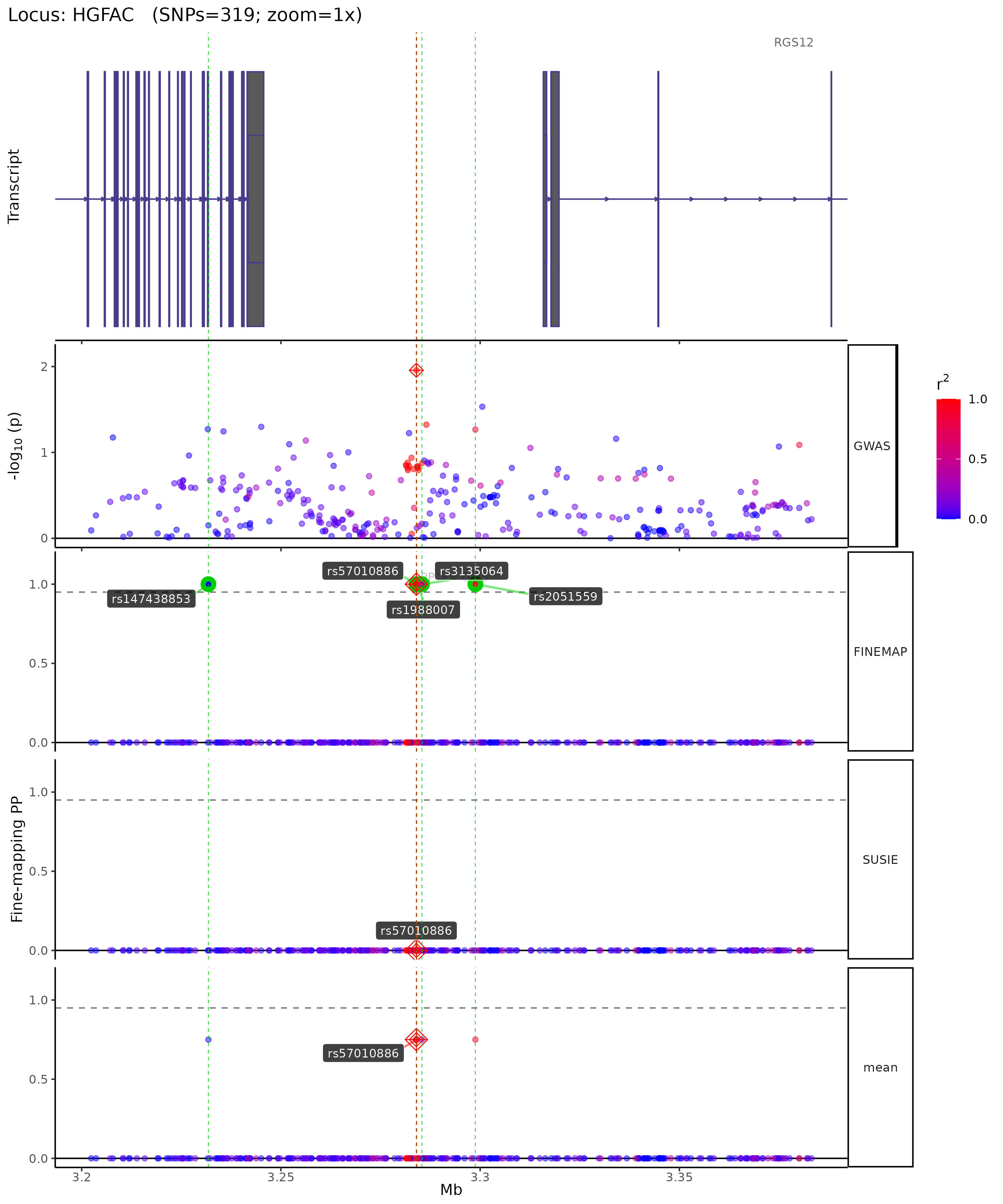

Supplement: Supplementary file 6 — Supporting Information [file CTM2-16-e70732-s005.zip › HGFAC/multiview.HGFAC.1KGphase3.1x.png]

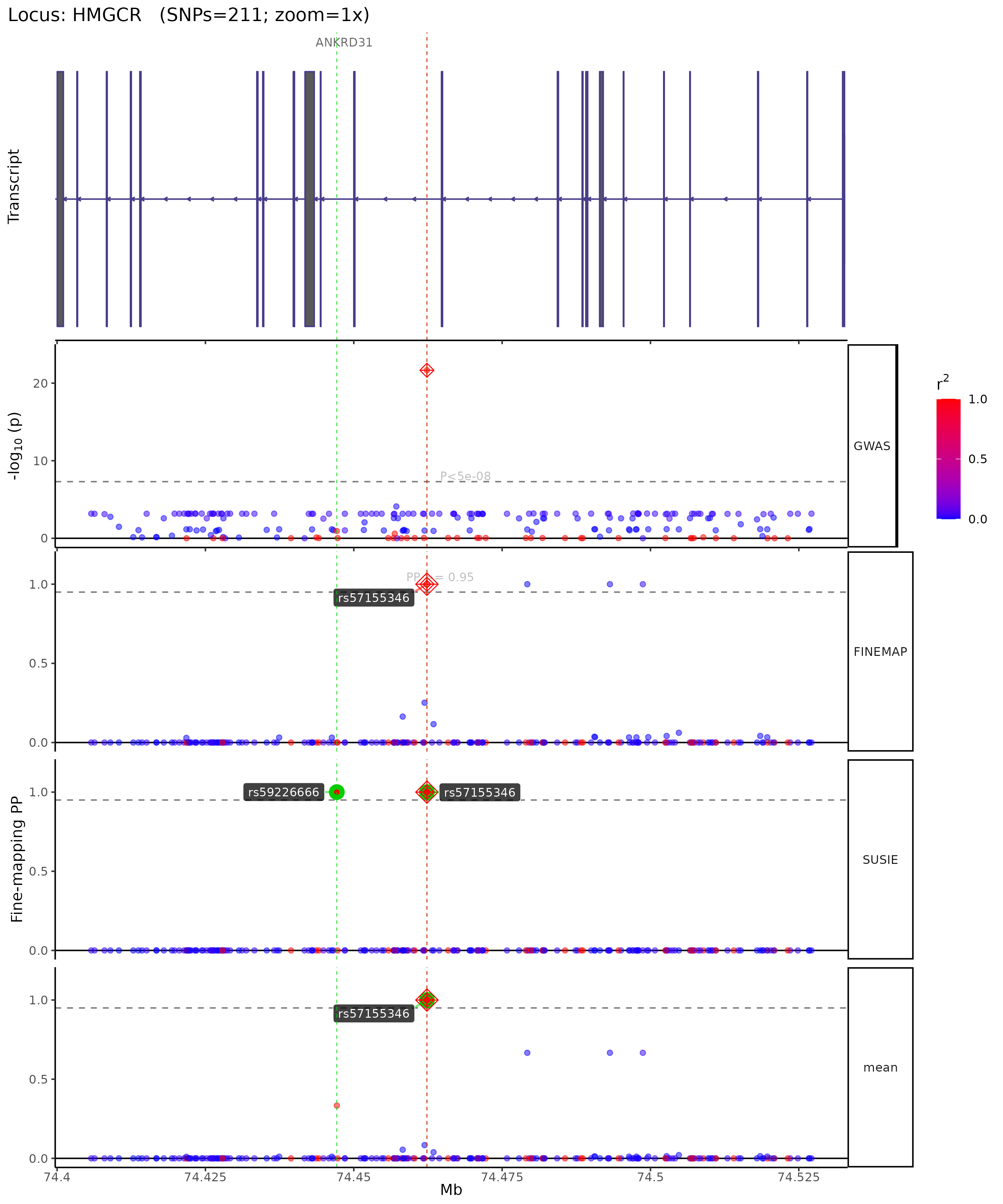

Supplement: Supplementary file 6 — Supporting Information [file CTM2-16-e70732-s005.zip › HMGCR/multiview.HMGCR.1KGphase3.1x.png]

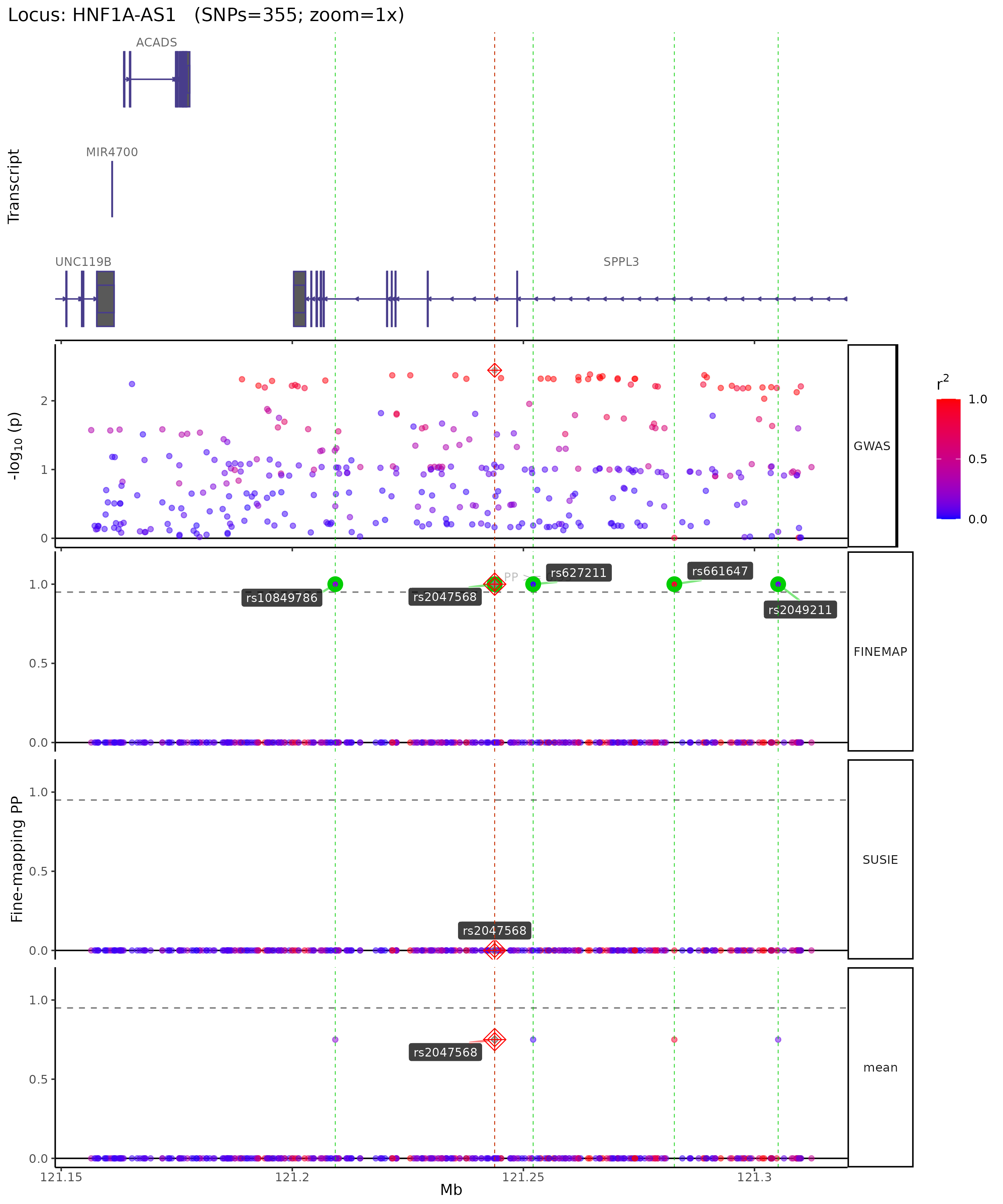

Supplement: Supplementary file 6 — Supporting Information [file CTM2-16-e70732-s005.zip › HNF1A-AS1/multiview.HNF1A-AS1.1KGphase3.1x.png]

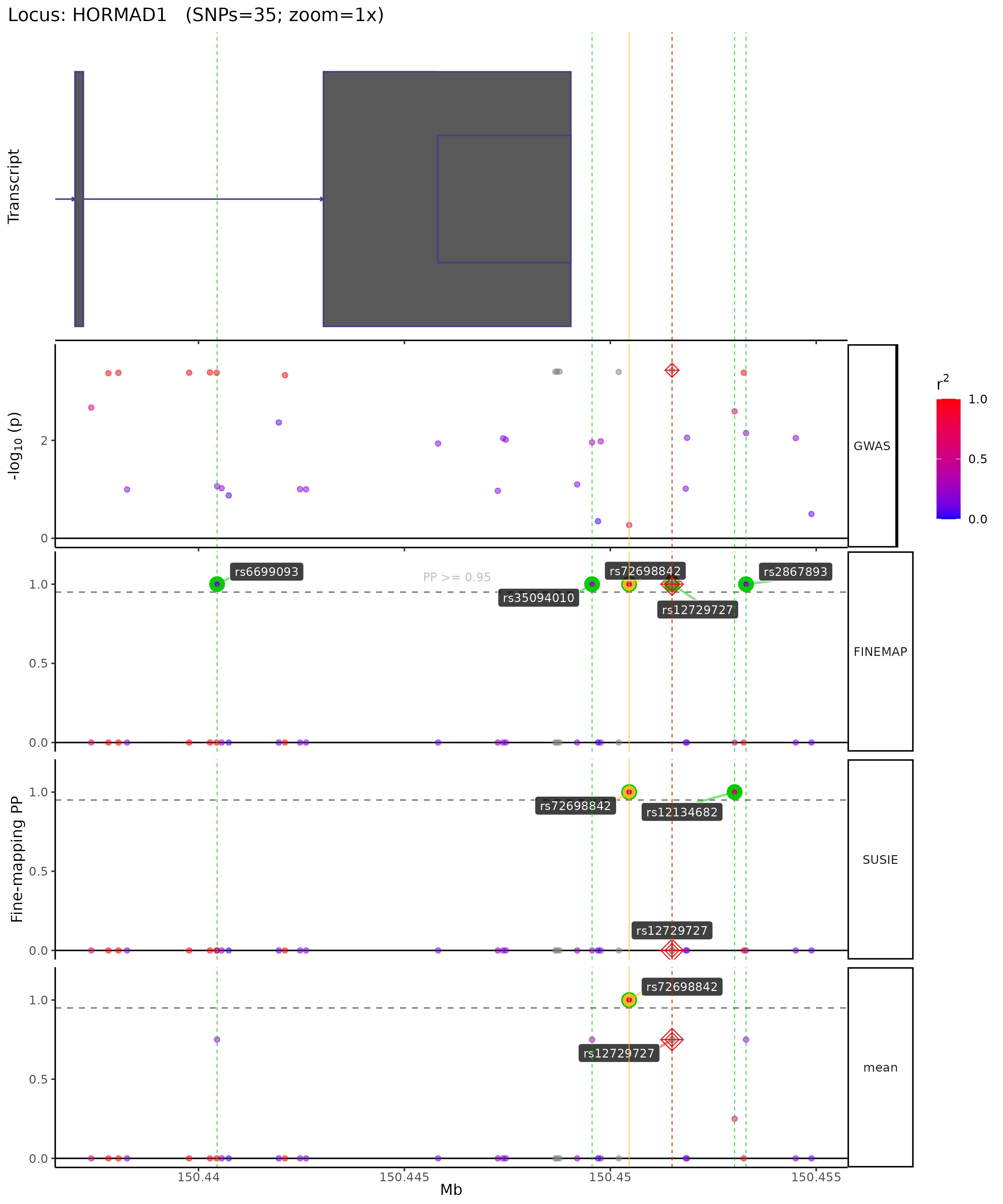

Supplement: Supplementary file 6 — Supporting Information [file CTM2-16-e70732-s005.zip › HORMAD1/multiview.HORMAD1.1KGphase3.1x.png]

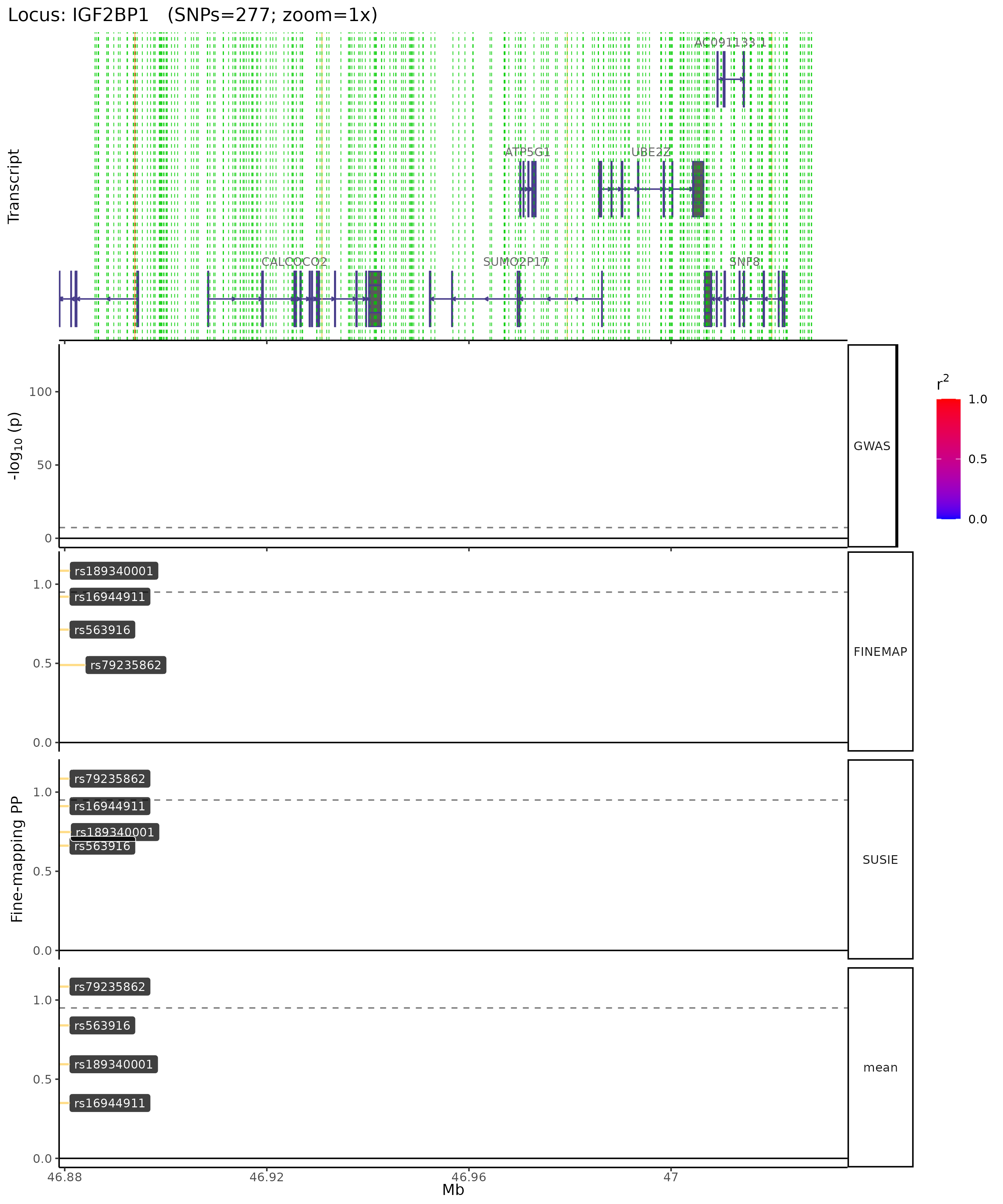

Supplement: Supplementary file 6 — Supporting Information [file CTM2-16-e70732-s005.zip › IGF2BP1/multiview.IGF2BP1.1KGphase3.1x.png]

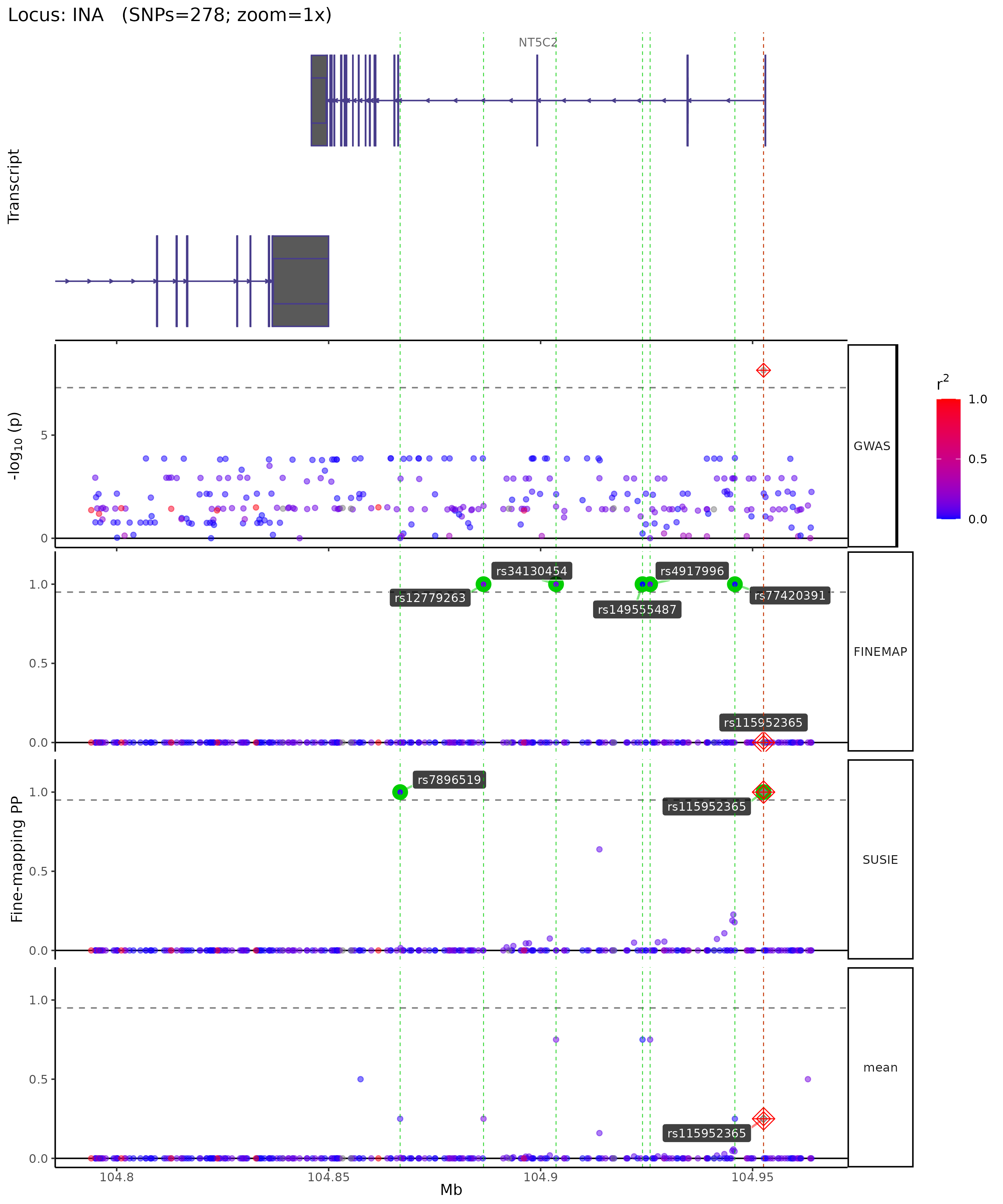

Supplement: Supplementary file 6 — Supporting Information [file CTM2-16-e70732-s005.zip › INA/multiview.INA.1KGphase3.1x.png]

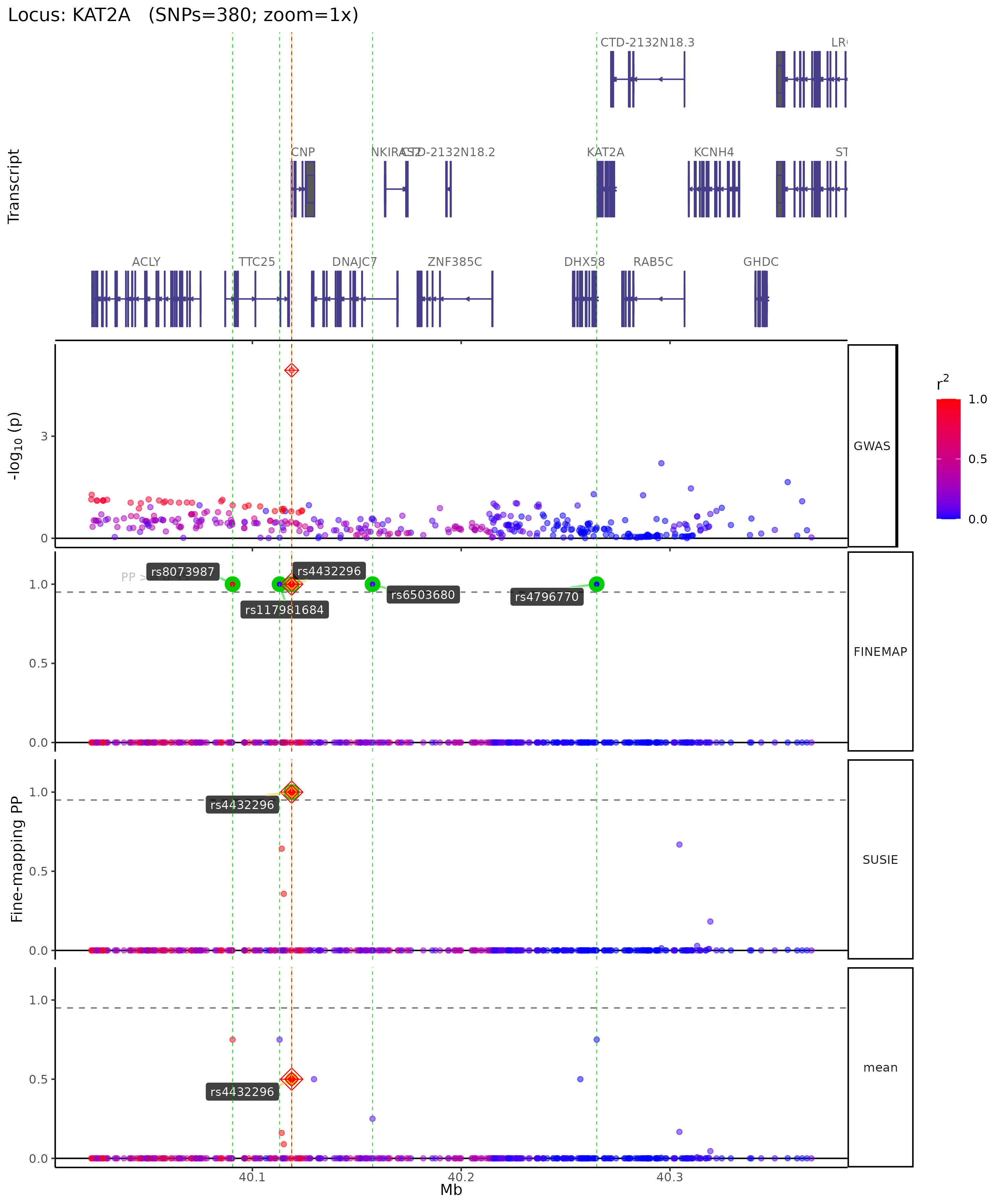

Supplement: Supplementary file 6 — Supporting Information [file CTM2-16-e70732-s005.zip › KAT2A/multiview.KAT2A.1KGphase3.1x.png]

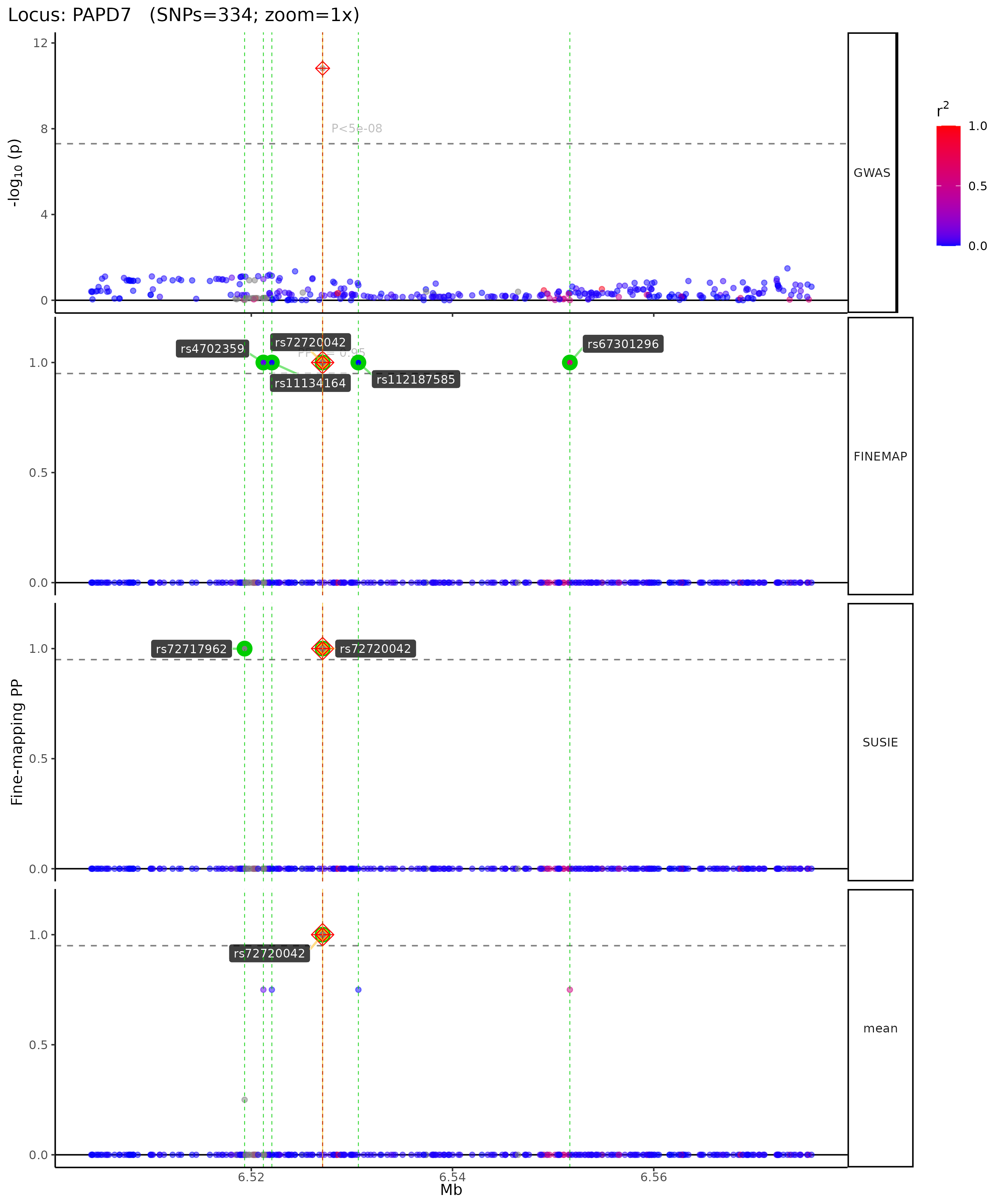

Supplement: Supplementary file 6 — Supporting Information [file CTM2-16-e70732-s005.zip › PAPD7/multiview.PAPD7.1KGphase3.1x.png]

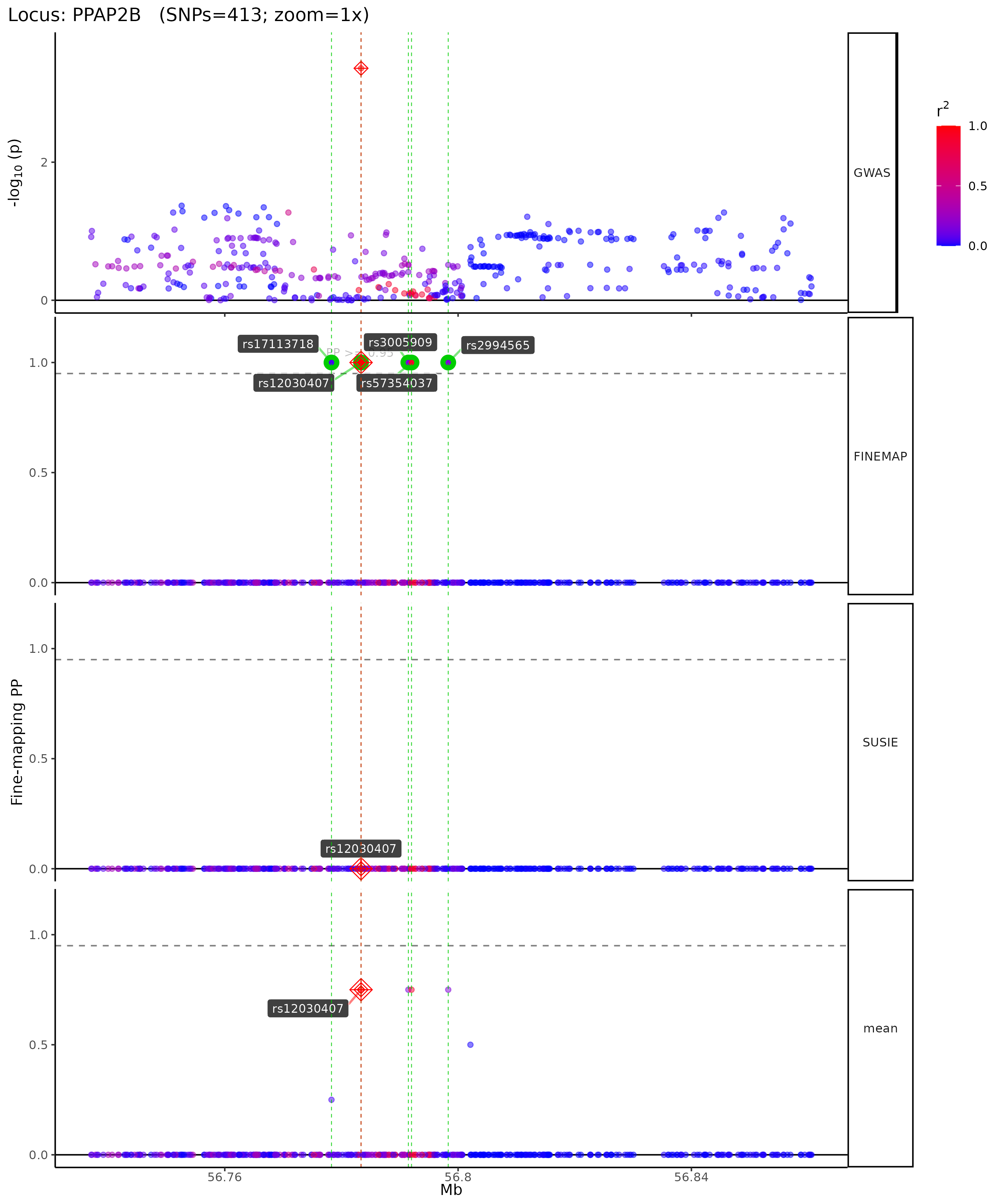

Supplement: Supplementary file 6 — Supporting Information [file CTM2-16-e70732-s005.zip › PPAP2B/multiview.PPAP2B.1KGphase3.1x.png]

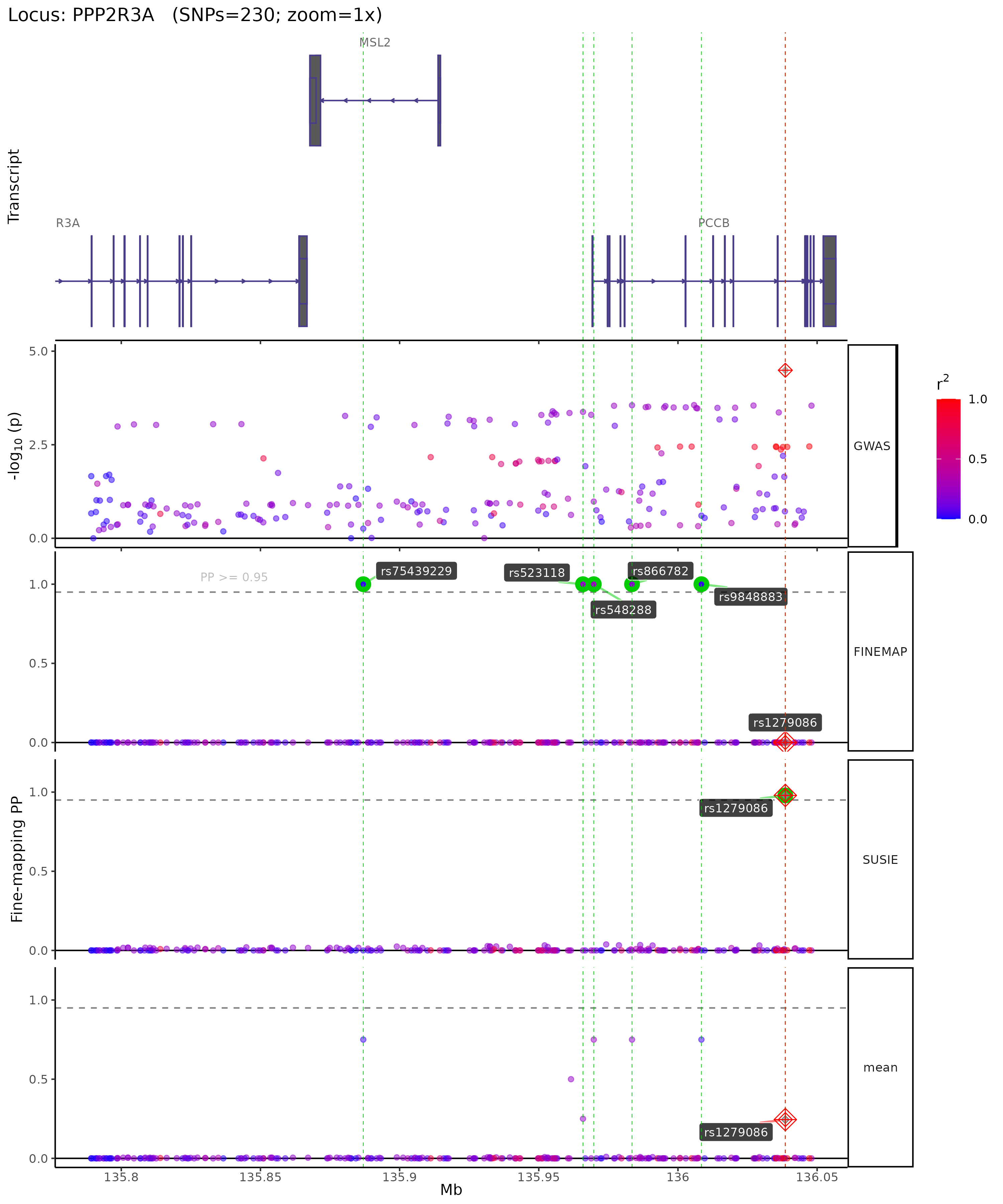

Supplement: Supplementary file 6 — Supporting Information [file CTM2-16-e70732-s005.zip › PPP2R3A/multiview.PPP2R3A.1KGphase3.1x.png]

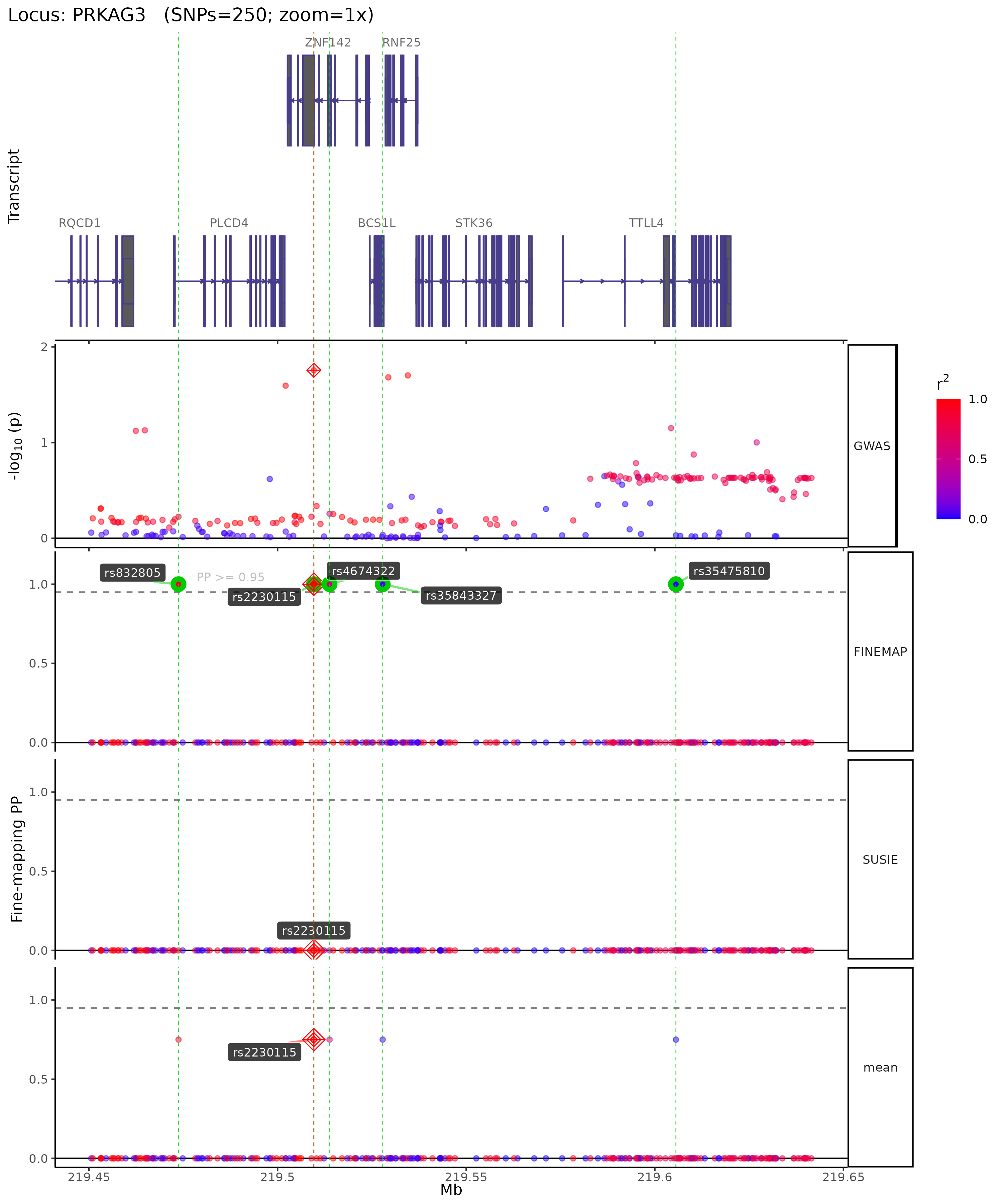

Supplement: Supplementary file 6 — Supporting Information [file CTM2-16-e70732-s005.zip › PRKAG3/multiview.PRKAG3.1KGphase3.1x.png]

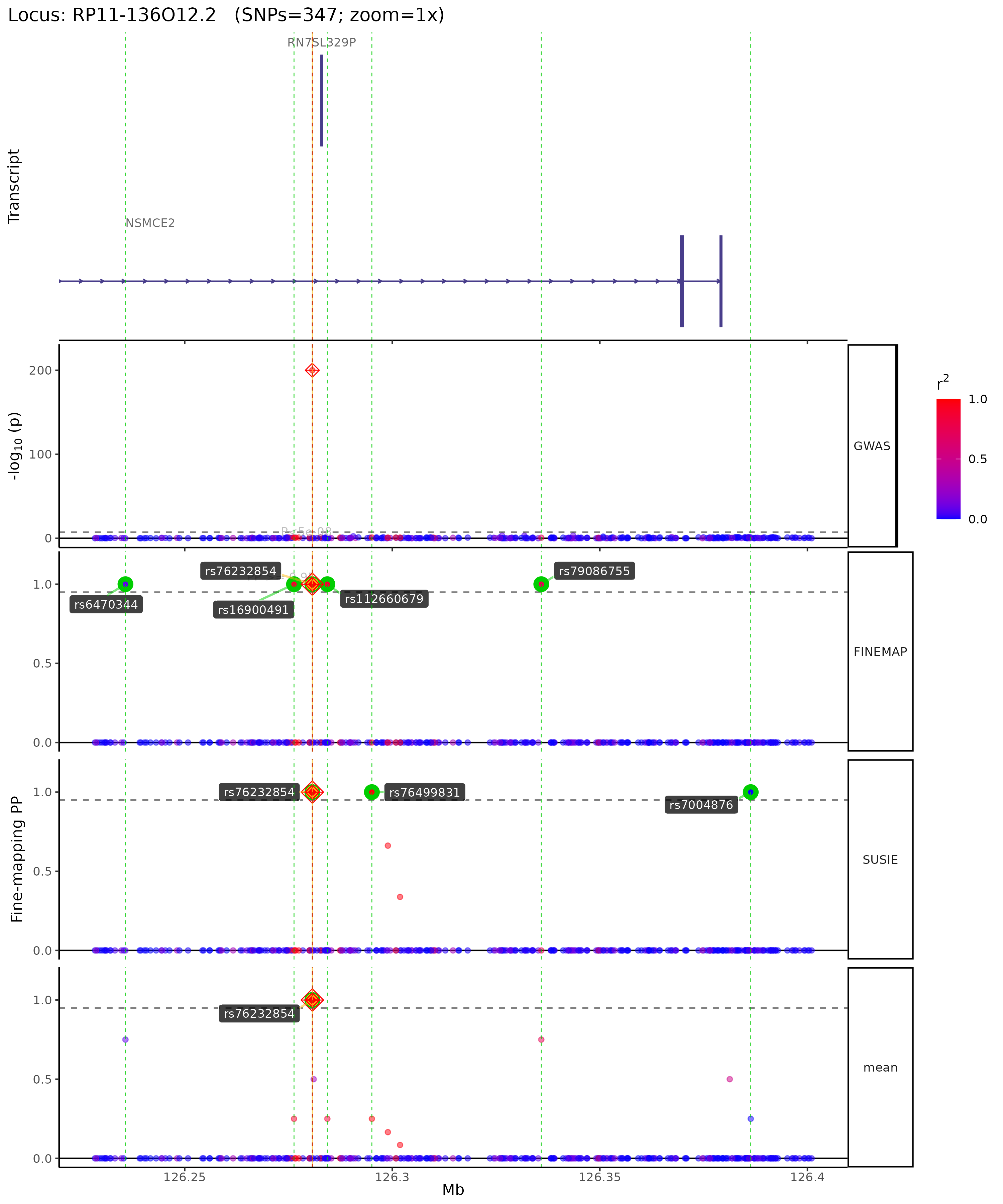

Supplement: Supplementary file 6 — Supporting Information [file CTM2-16-e70732-s005.zip › RP11-136O12.2/multiview.RP11-136O12.2.1KGphase3.1x.png]

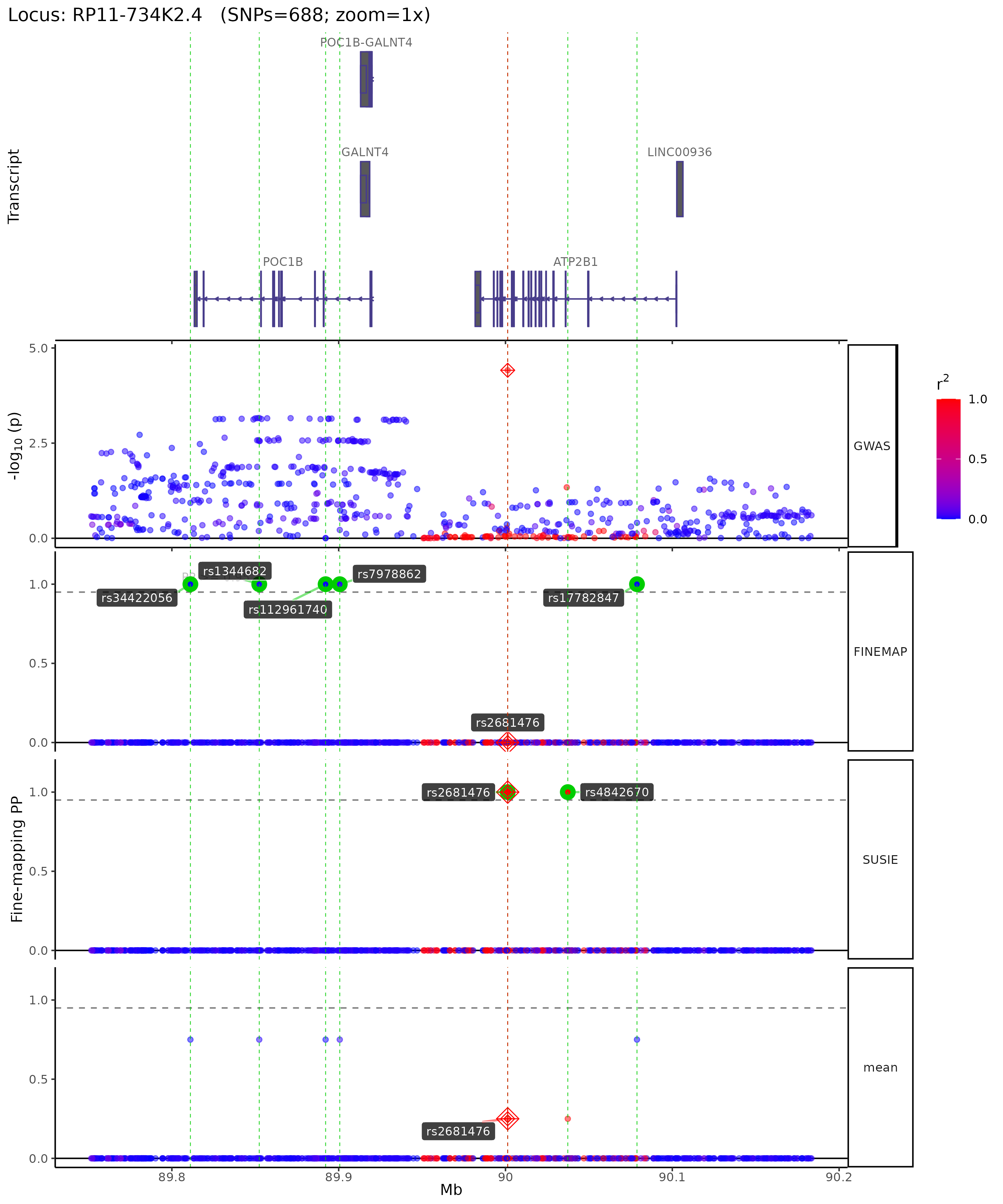

Supplement: Supplementary file 6 — Supporting Information [file CTM2-16-e70732-s005.zip › RP11-734K2.4/multiview.RP11-734K2.4.1KGphase3.1x.png]

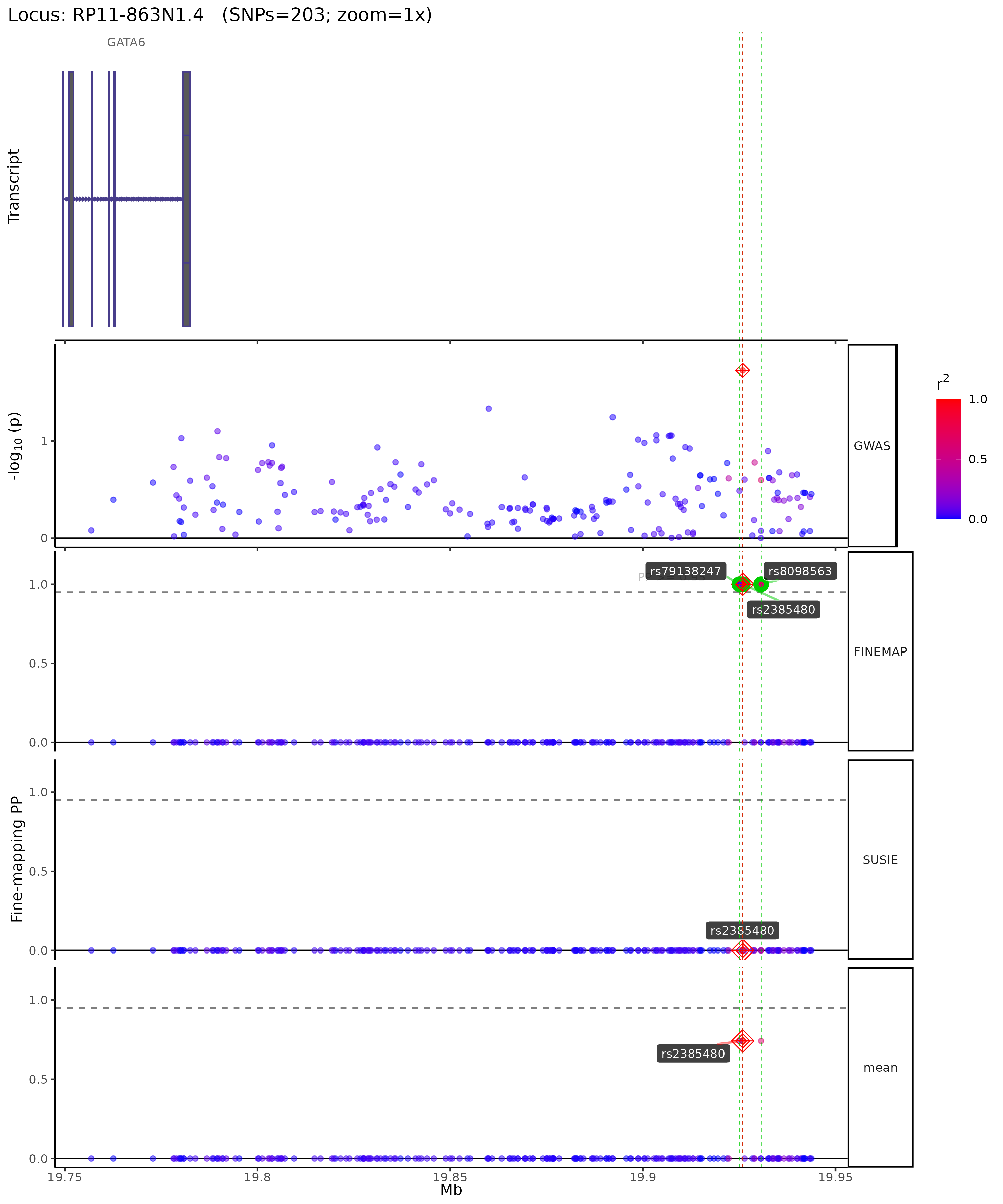

Supplement: Supplementary file 6 — Supporting Information [file CTM2-16-e70732-s005.zip › RP11-863N1.4/multiview.RP11-863N1.4.1KGphase3.1x.png]

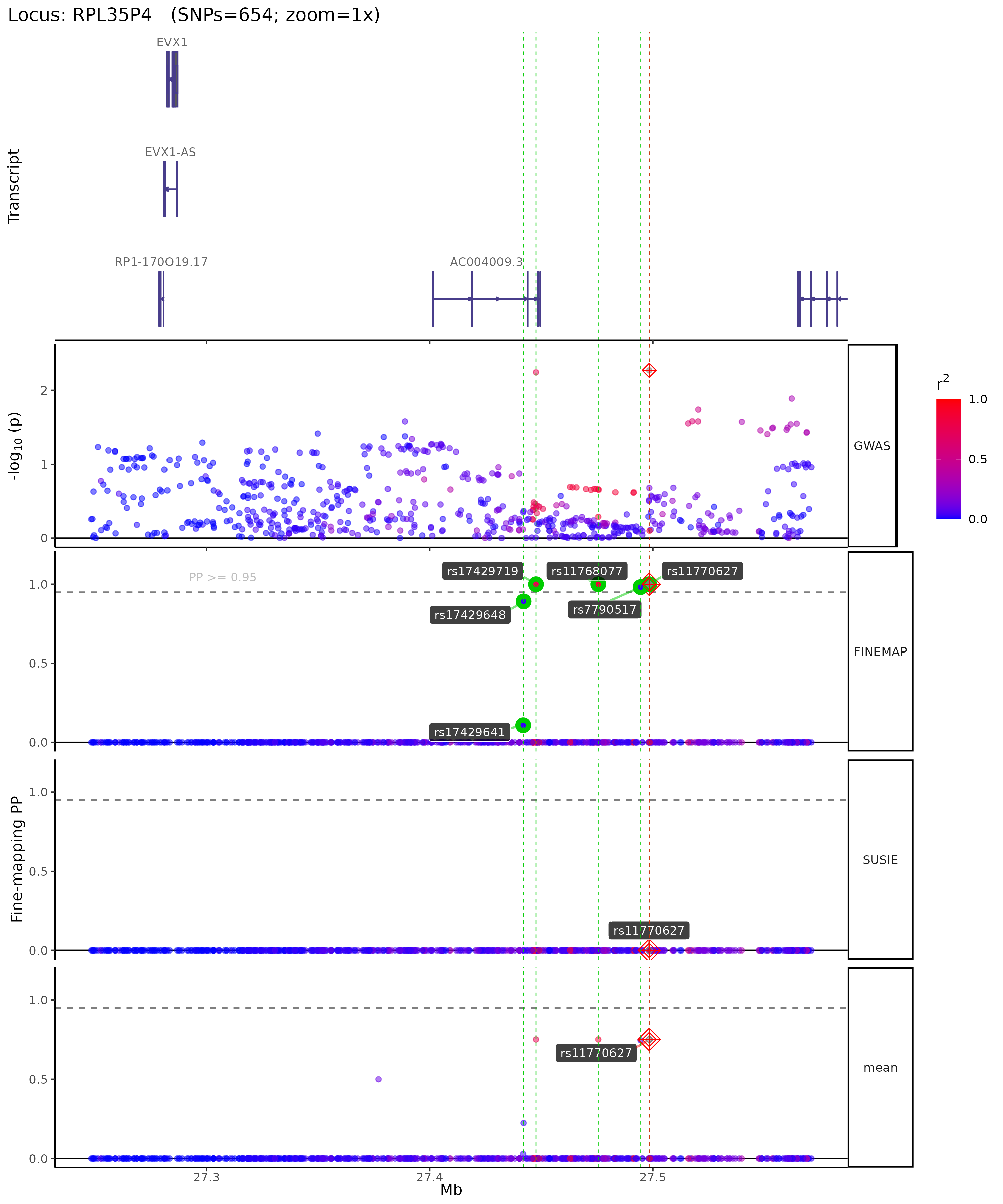

Supplement: Supplementary file 6 — Supporting Information [file CTM2-16-e70732-s005.zip › RPL35P4/multiview.RPL35P4.1KGphase3.1x.png]

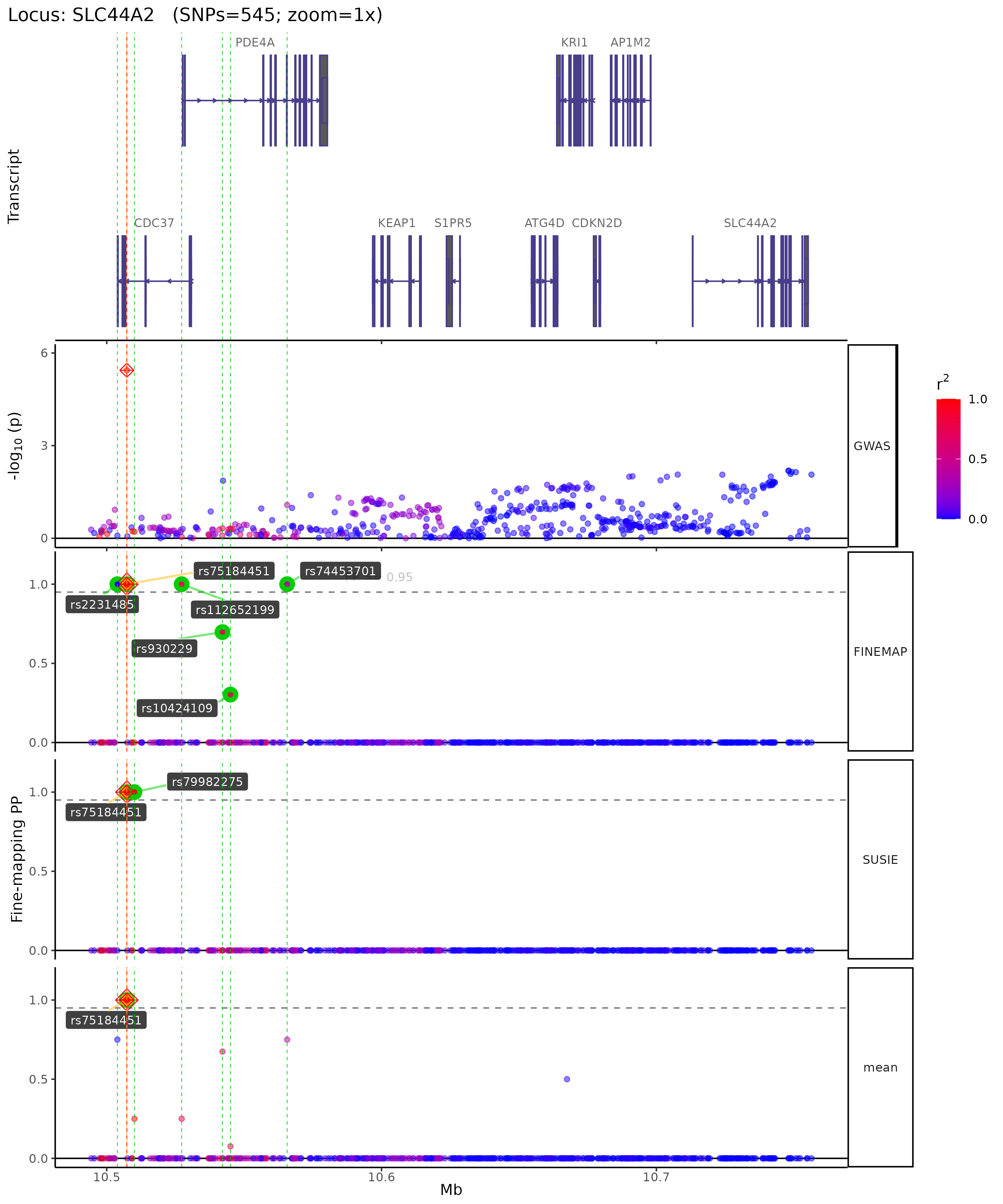

Supplement: Supplementary file 6 — Supporting Information [file CTM2-16-e70732-s005.zip › SLC44A2/multiview.SLC44A2.1KGphase3.1x.png]
