# Supplementary figures and images for: Precision medicine for atherosclerotic cardiovascular disease: Integrative genomics maps risk loci and AI‐predicted functional consequences (part 2 of 3)
Source: Clin Transl Med. 2026 Jul 10;16(7):e70732. doi: 10.1002/ctm2.70732 (PMC13351343; doi:10.1002/ctm2.70732)

# LocusZoom plots of GWAS top lead SNP

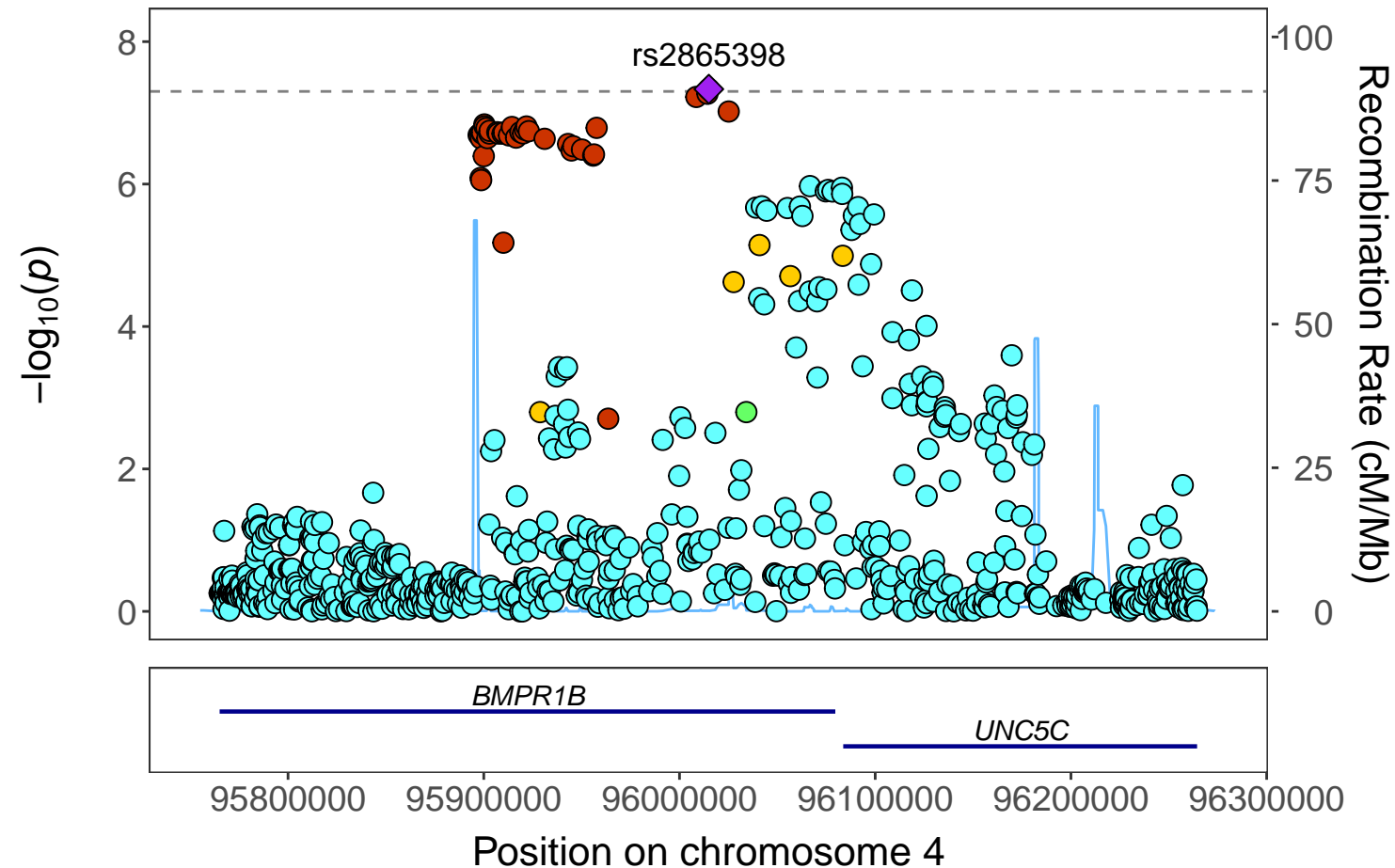

$r^2$    miss   cyan   0.0–0.2   green   0.2–0.4   yellow   0.4–0.6   orange   0.6–0.8   red   0.8–1.0

Supplement: Supplementary file 5 — Supporting Information [file CTM2-16-e70732-s001.zip › LocusZoom/Sfig_rs2865398_locusZoom.pdf]

# LocusZoom plots of GWAS top lead SNP

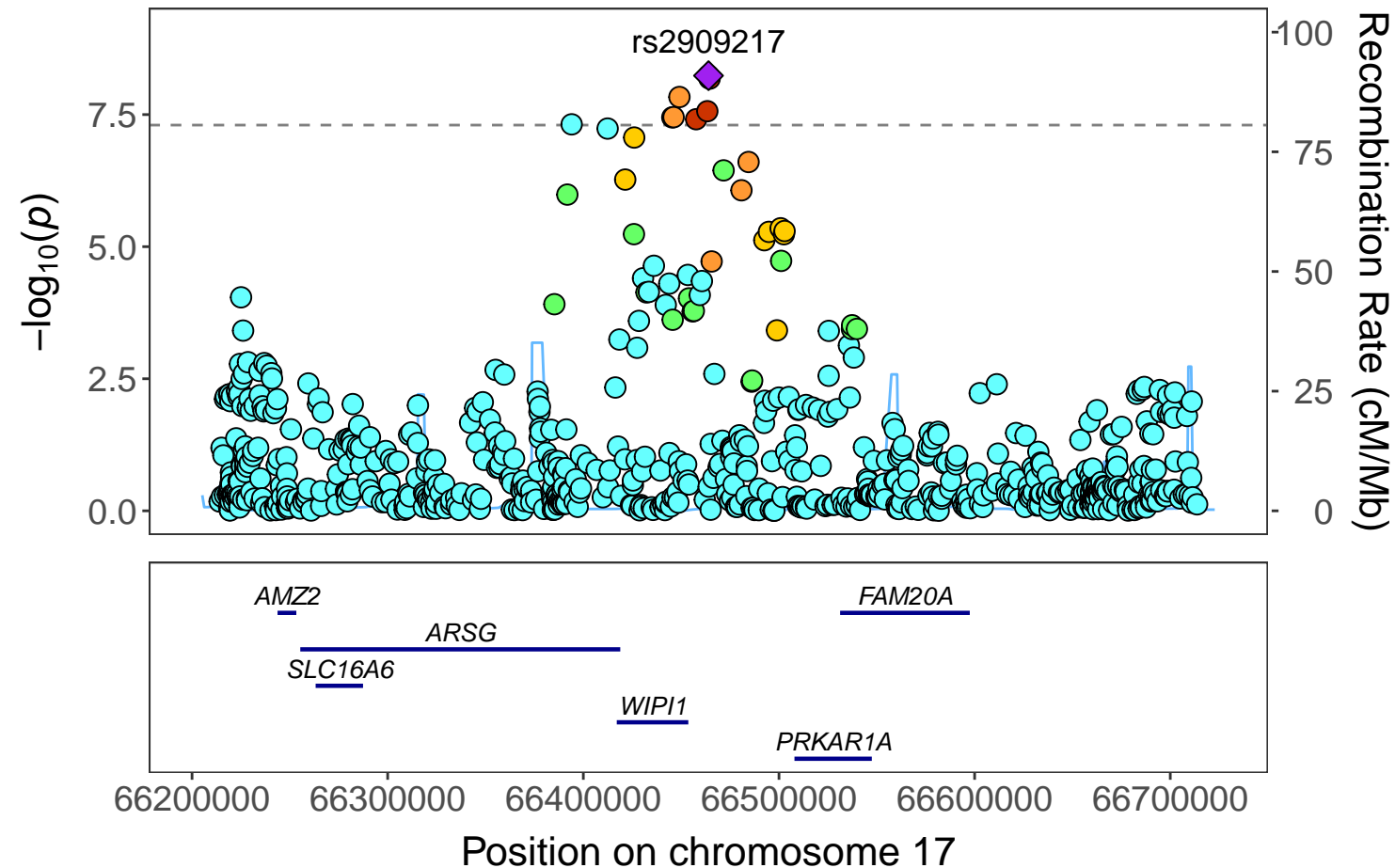

r2    miss    0.0–0.2    0.2–0.4    0.4–0.6    0.6–0.8    0.8–1.0

Supplement: Supplementary file 5 — Supporting Information [file CTM2-16-e70732-s001.zip › LocusZoom/Sfig_rs2909217_locusZoom.pdf]

# LocusZoom plots of GWAS top lead SNP

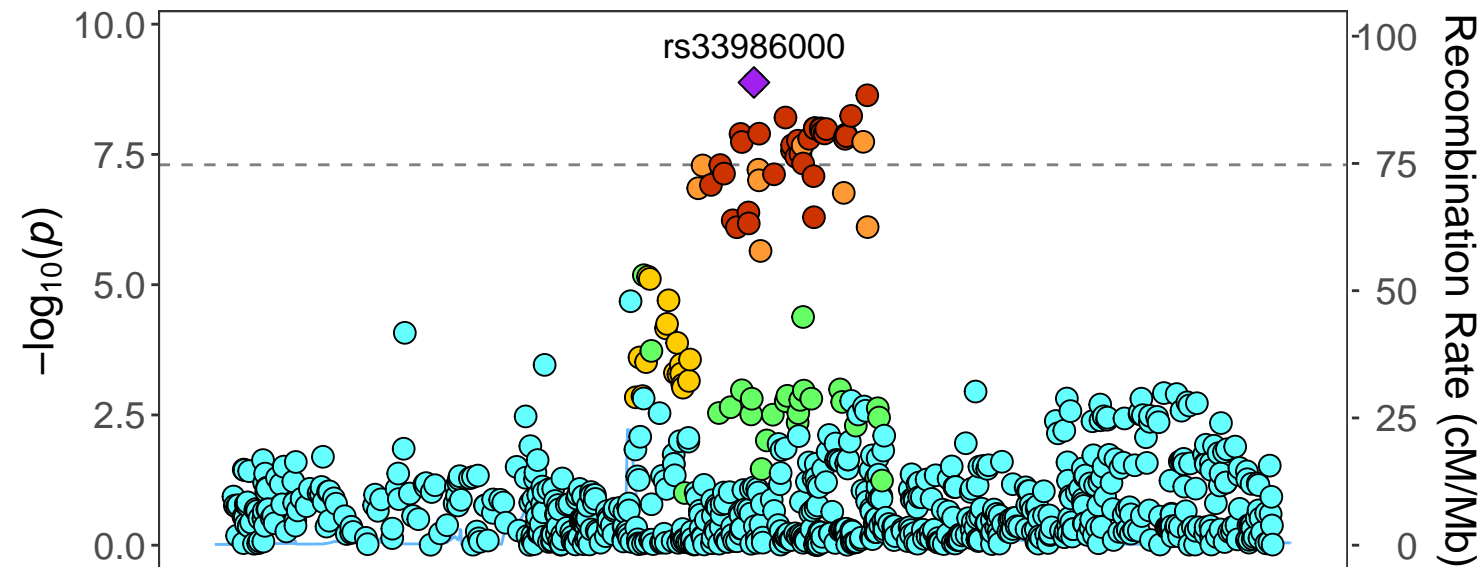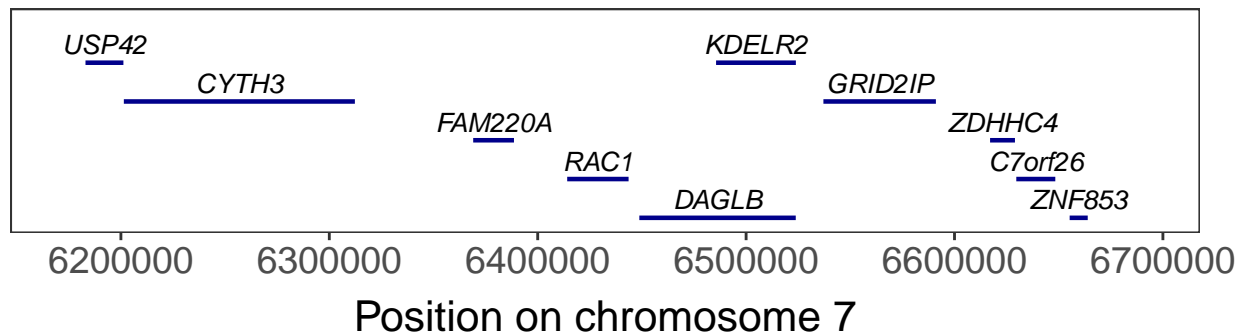

r2    miss    0.0–0.2    0.2–0.4    0.4–0.6    0.6–0.8    0.8–1.0

Supplement: Supplementary file 5 — Supporting Information [file CTM2-16-e70732-s001.zip › LocusZoom/Sfig_rs33986000_locusZoom.pdf]

# LocusZoom plots of GWAS top lead SNP

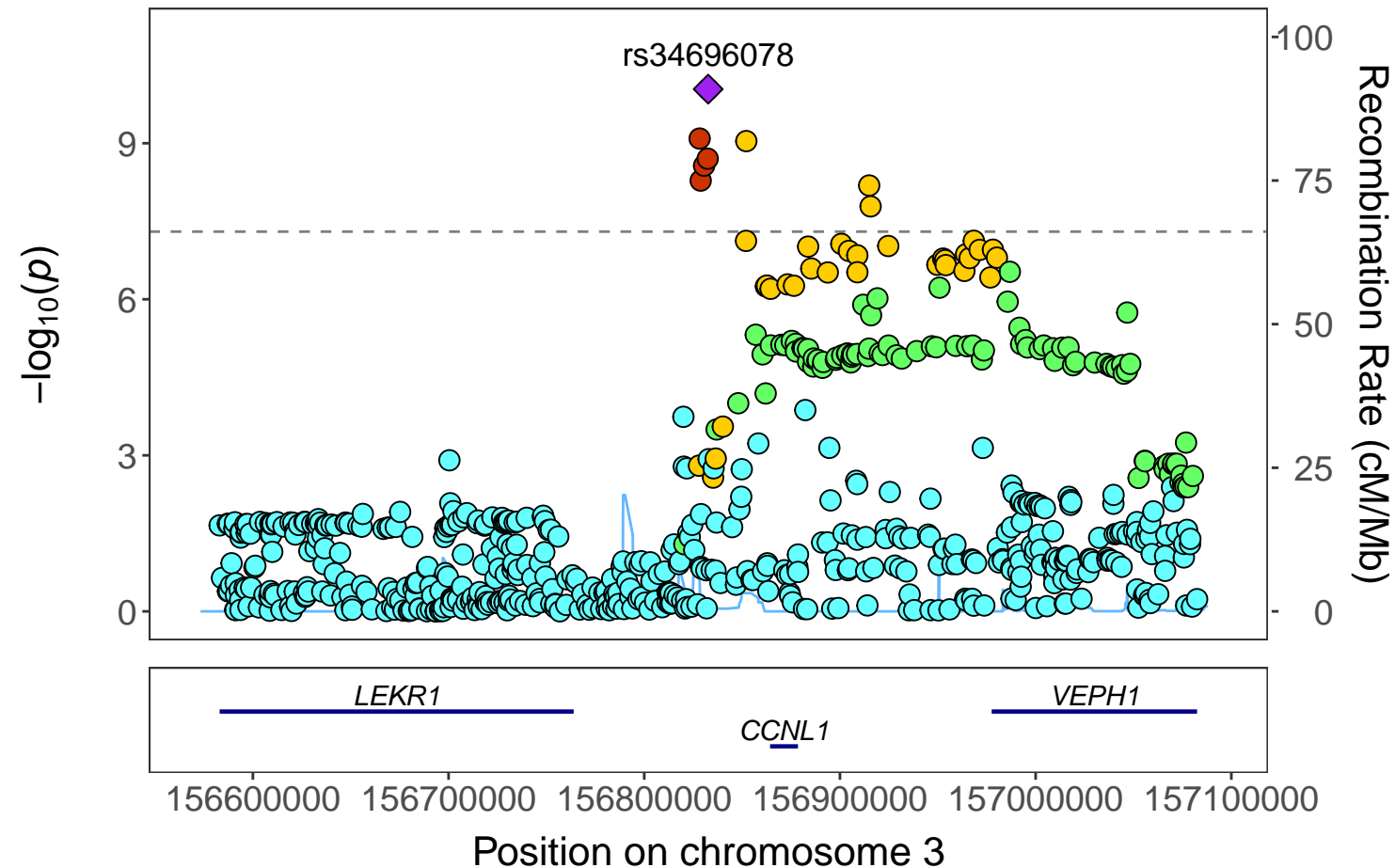

$r^2$    miss   0.0-0.2   0.2-0.4   0.4-0.6   0.6-0.8   0.8-1.0

Supplement: Supplementary file 5 — Supporting Information [file CTM2-16-e70732-s001.zip › LocusZoom/Sfig_rs34696078_locusZoom.pdf]

# LocusZoom plots of GWAS top lead SNP

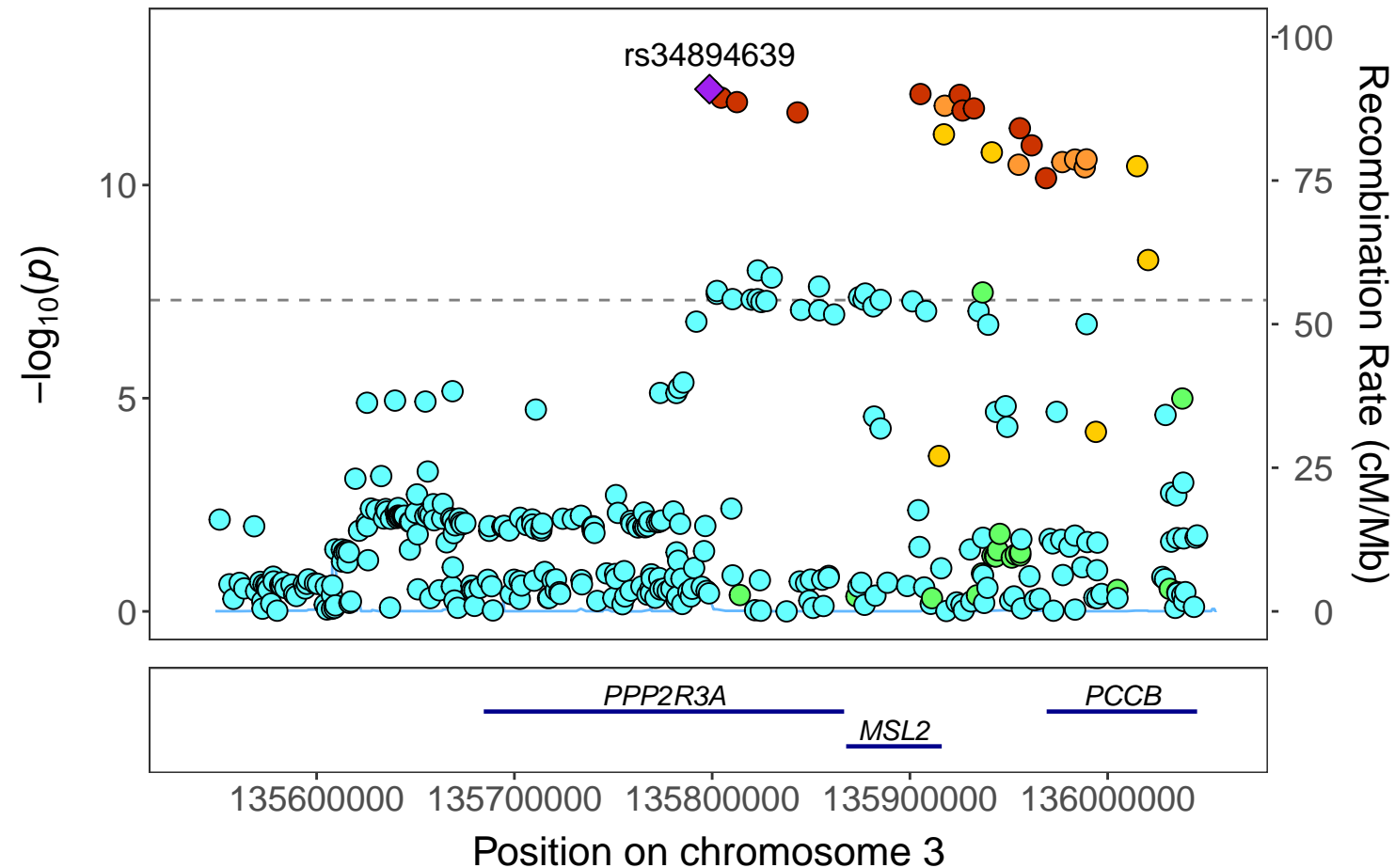

$r^2$     $\circ$  miss    $\circ$  0.0–0.2    $\circ$  0.2–0.4    $\circ$  0.4–0.6    $\circ$  0.6–0.8    $\circ$  0.8–1.0

Supplement: Supplementary file 5 — Supporting Information [file CTM2-16-e70732-s001.zip › LocusZoom/Sfig_rs34894639_locusZoom.pdf]

# LocusZoom plots of GWAS top lead SNP

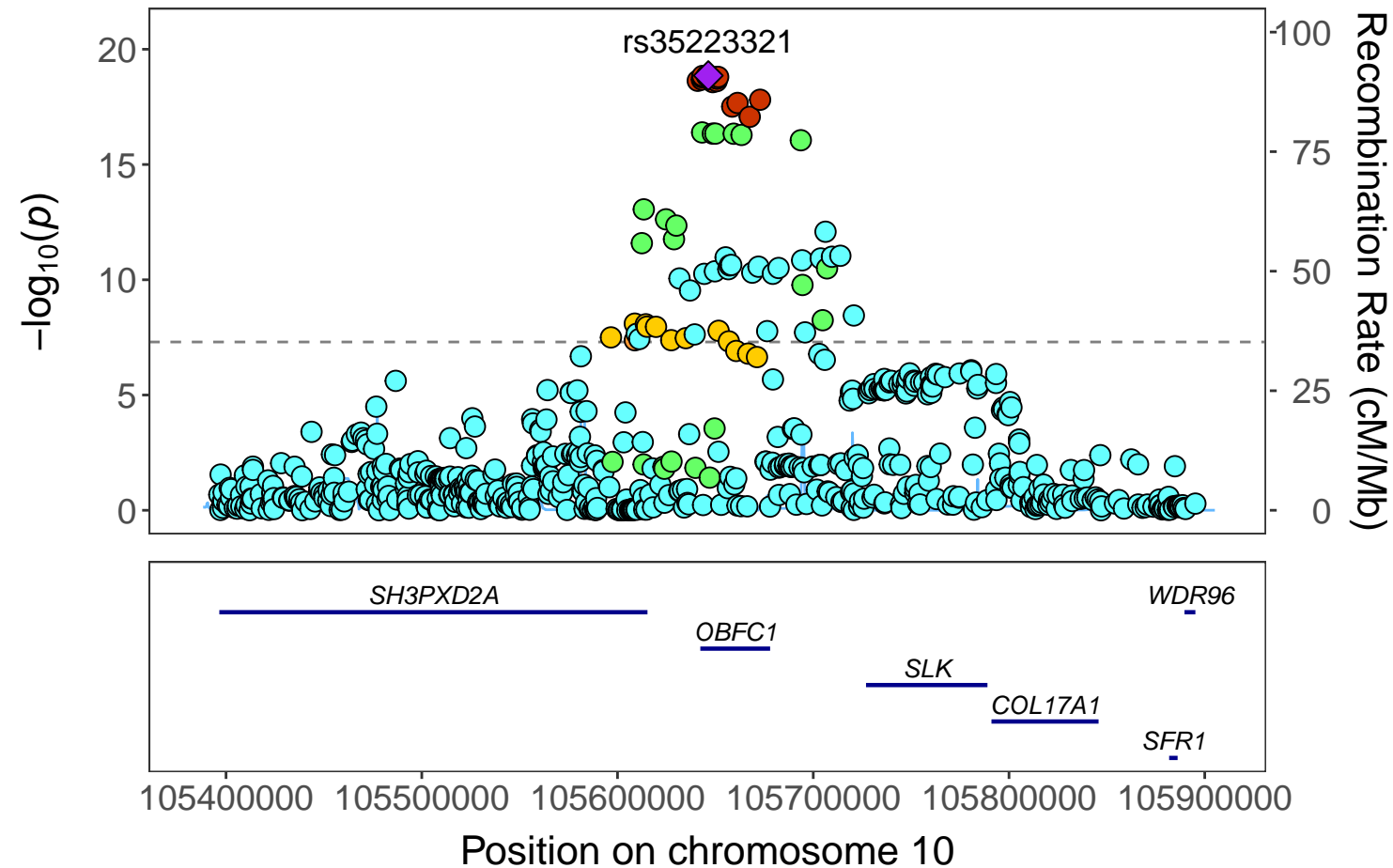

Supplement: Supplementary file 5 — Supporting Information [file CTM2-16-e70732-s001.zip › LocusZoom/Sfig_rs35223321_locusZoom.pdf]

# LocusZoom plots of GWAS top lead SNP

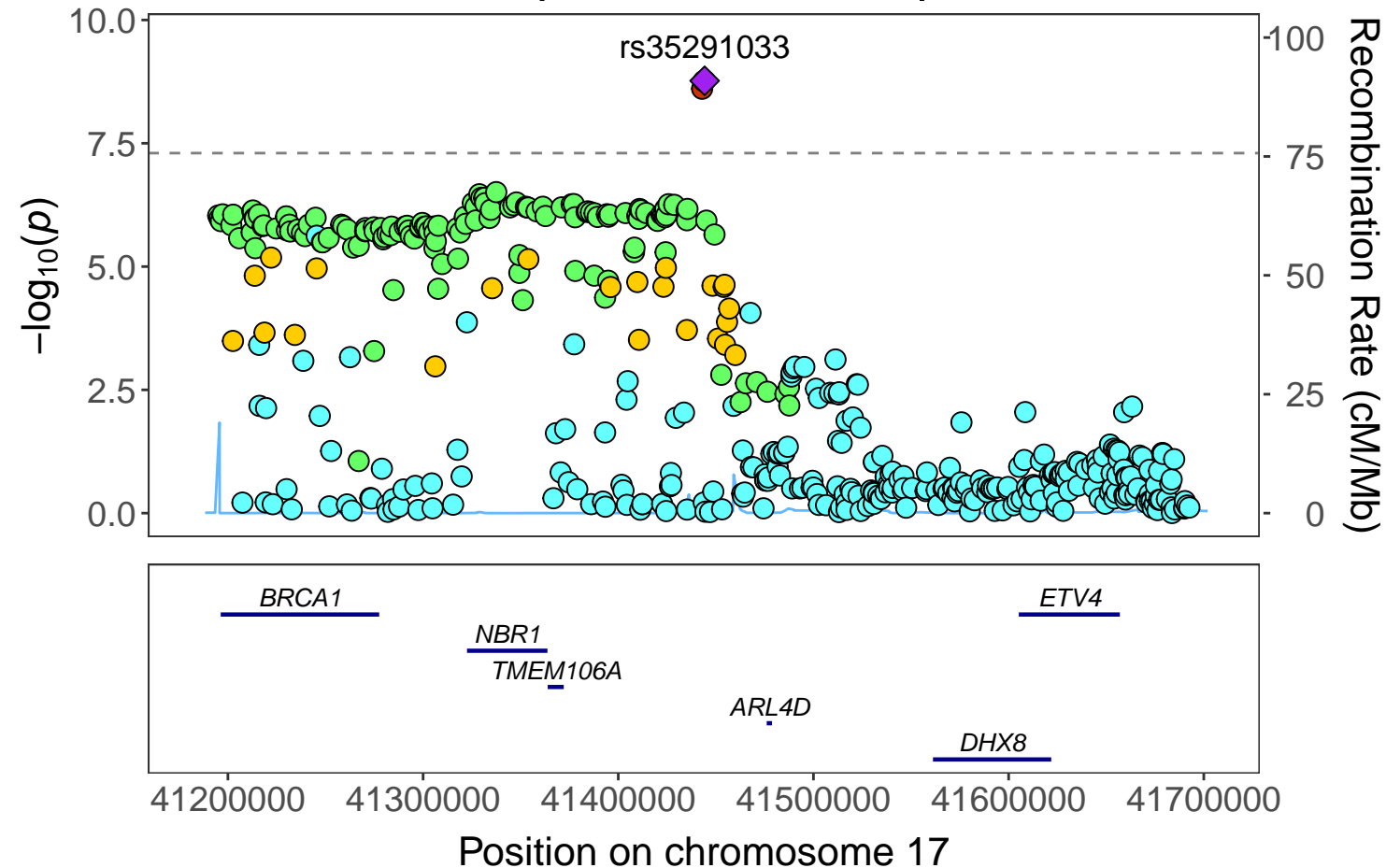

Supplement: Supplementary file 5 — Supporting Information [file CTM2-16-e70732-s001.zip › LocusZoom/Sfig_rs35291033_locusZoom.pdf]

# LocusZoom plots of GWAS top lead SNP

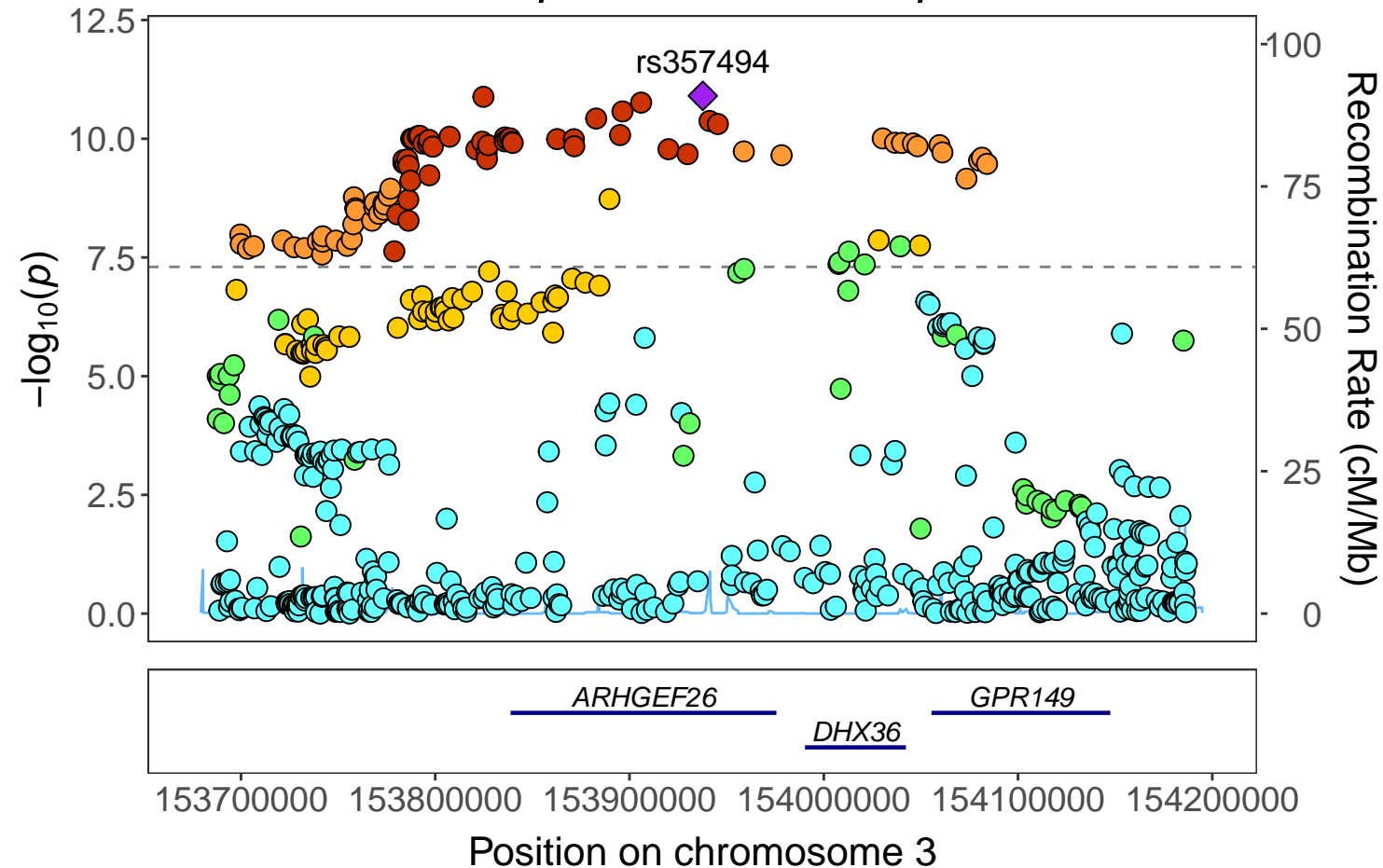

r2    miss    0.0–0.2    0.2–0.4    0.4–0.6    0.6–0.8    0.8–1.0

Supplement: Supplementary file 5 — Supporting Information [file CTM2-16-e70732-s001.zip › LocusZoom/Sfig_rs357494_locusZoom.pdf]

# LocusZoom plots of GWAS top lead SNP

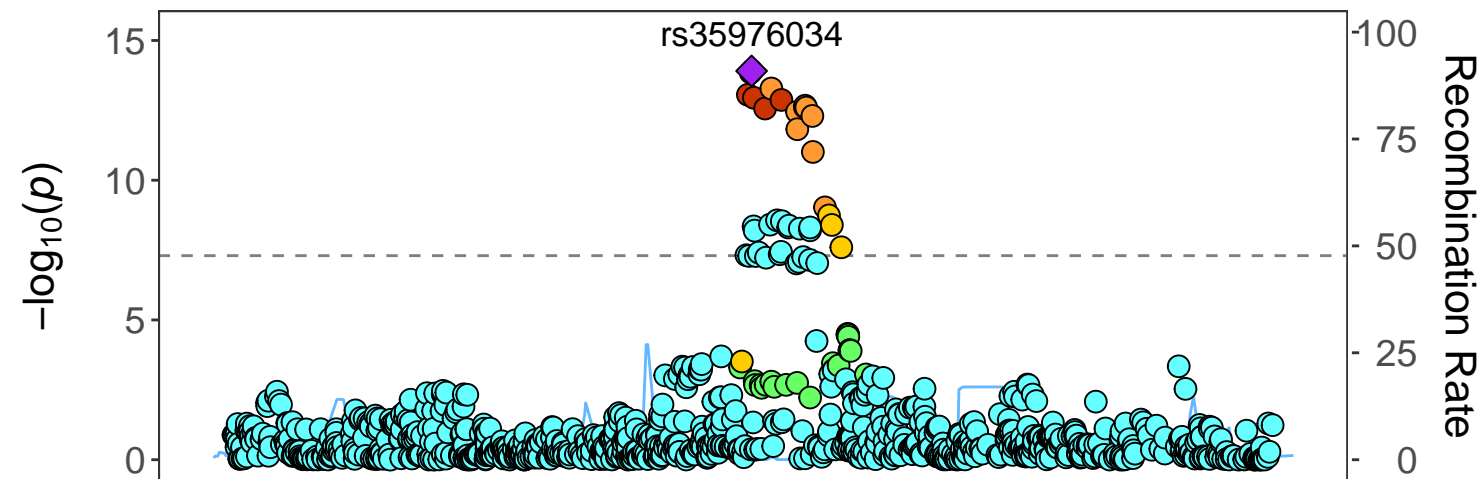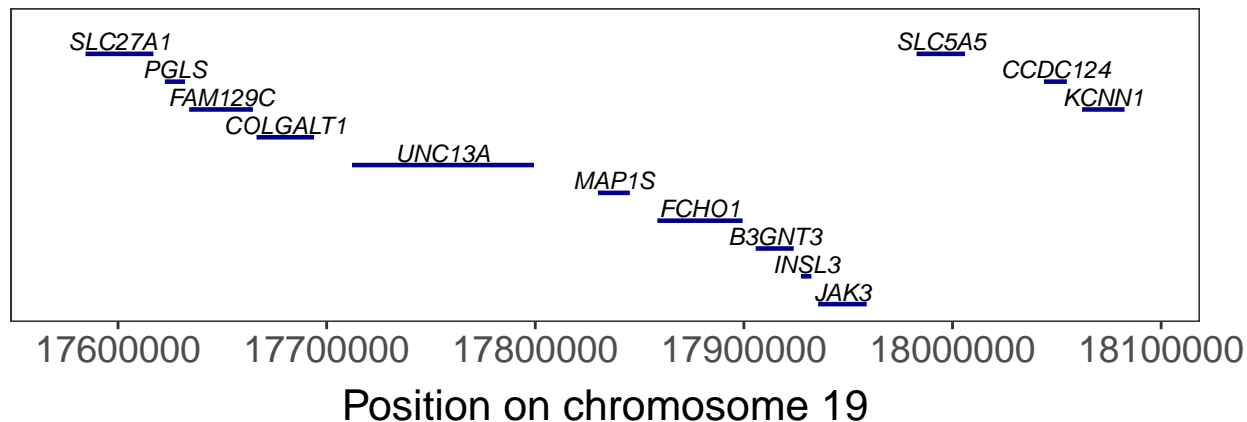

$r^2$  ○ miss ○ 0.0–0.2 ○ 0.2–0.4 ○ 0.4–0.6 ○ 0.6–0.8 ○ 0.8–1.0

Supplement: Supplementary file 5 — Supporting Information [file CTM2-16-e70732-s001.zip › LocusZoom/Sfig_rs35976034_locusZoom.pdf]

# *LocusZoom plots of GWAS top lead SNP*

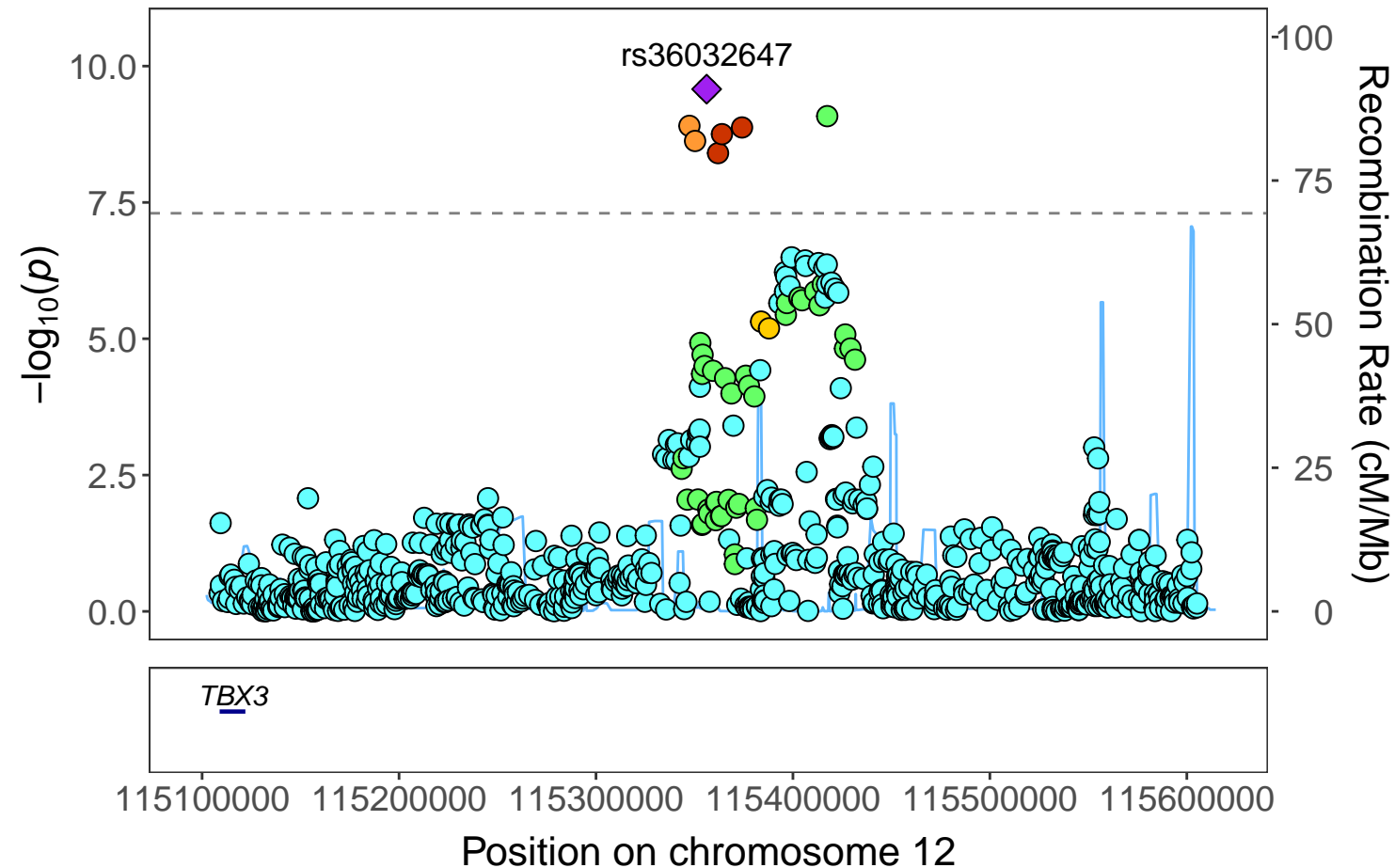

$r^2$  ○ miss ○ 0.0–0.2 ○ 0.2–0.4 ○ 0.4–0.6 ○ 0.6–0.8 ○ 0.8–1.0

Supplement: Supplementary file 5 — Supporting Information [file CTM2-16-e70732-s001.zip › LocusZoom/Sfig_rs36032647_locusZoom.pdf]

# LocusZoom plots of GWAS top lead SNP

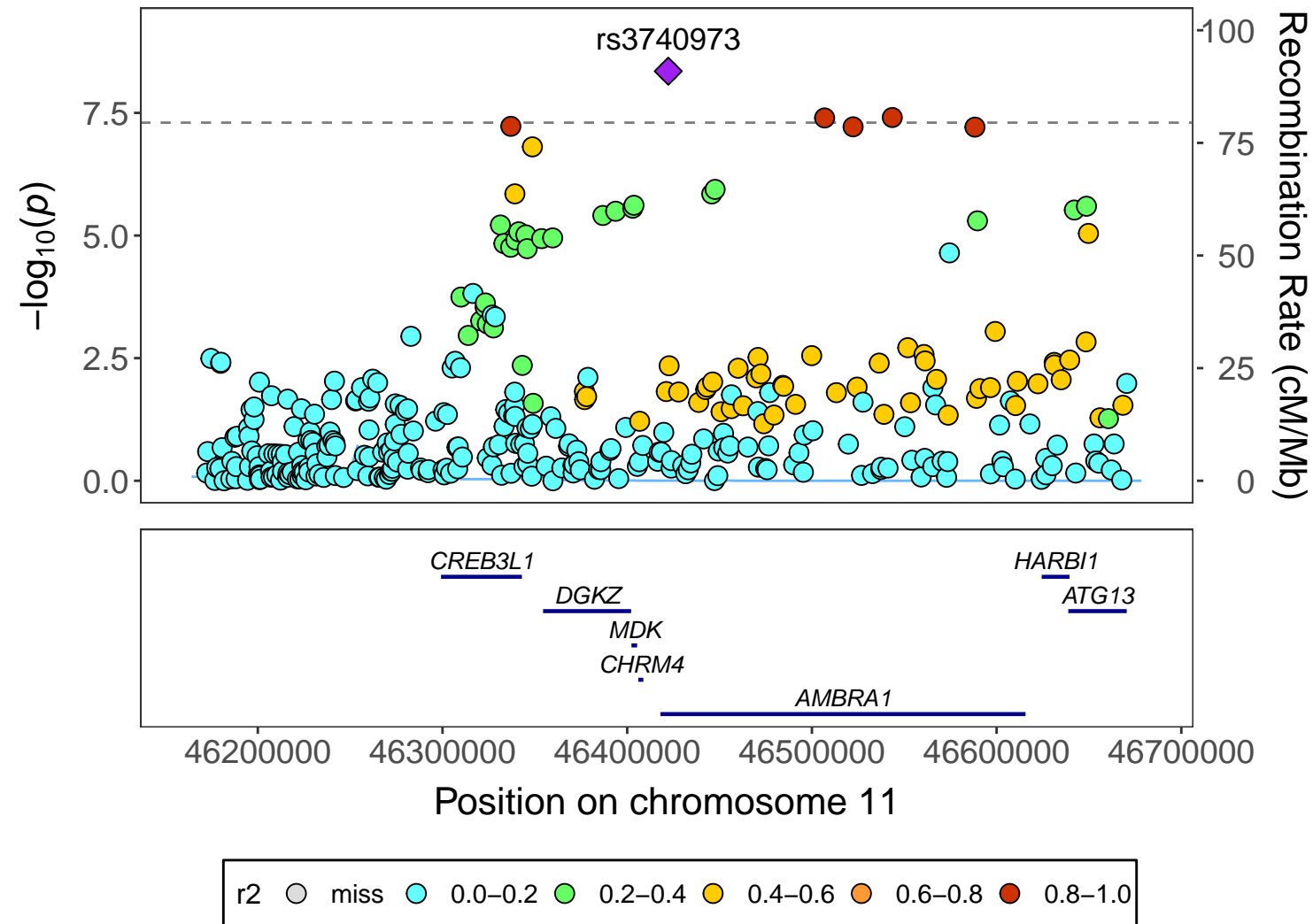

Supplement: Supplementary file 5 — Supporting Information [file CTM2-16-e70732-s001.zip › LocusZoom/Sfig_rs3740973_locusZoom.pdf]

# LocusZoom plots of GWAS top lead SNP

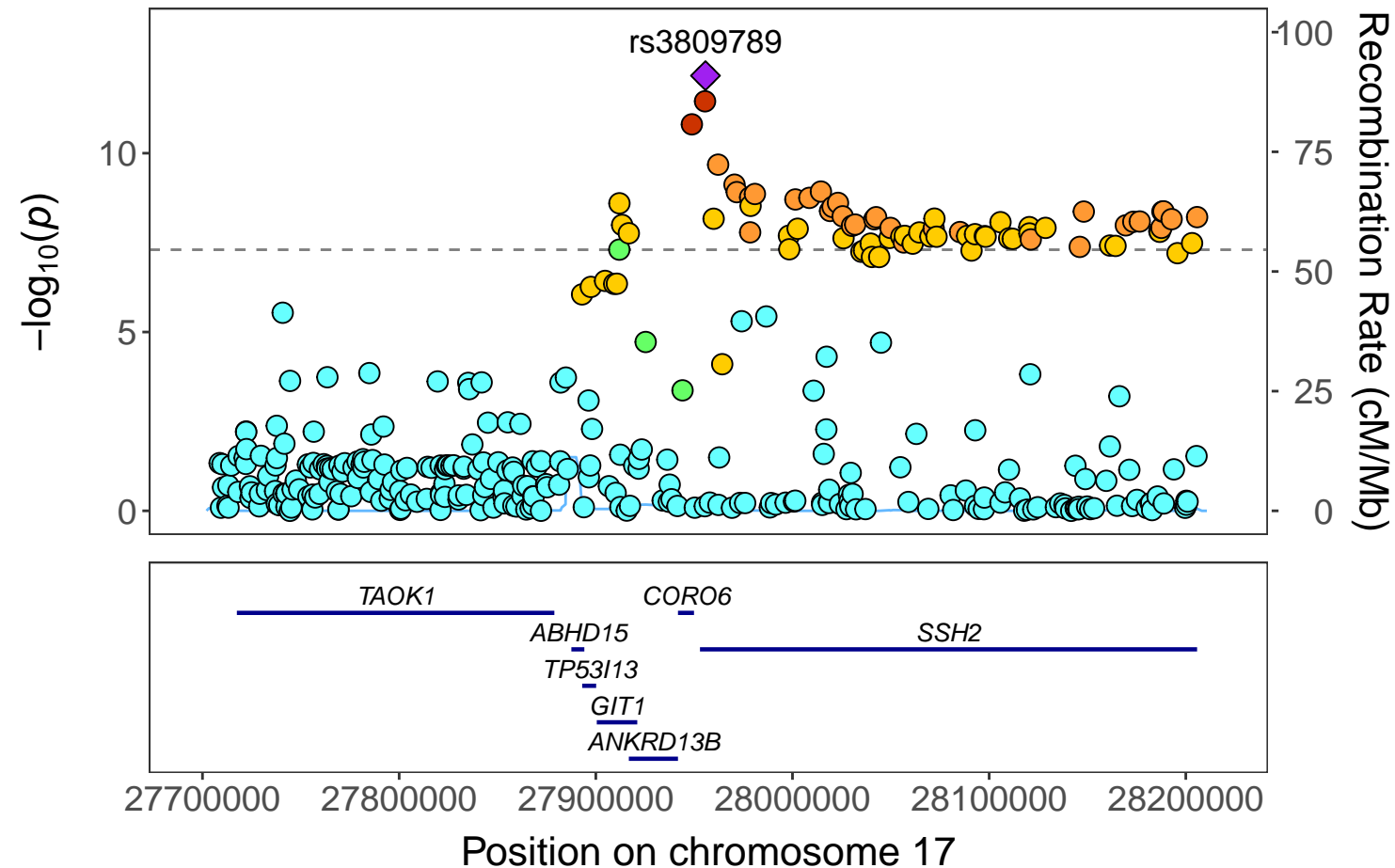

r2    miss    0.0–0.2    0.2–0.4    0.4–0.6    0.6–0.8    0.8–1.0

Supplement: Supplementary file 5 — Supporting Information [file CTM2-16-e70732-s001.zip › LocusZoom/Sfig_rs3809789_locusZoom.pdf]

# LocusZoom plots of GWAS top lead SNP

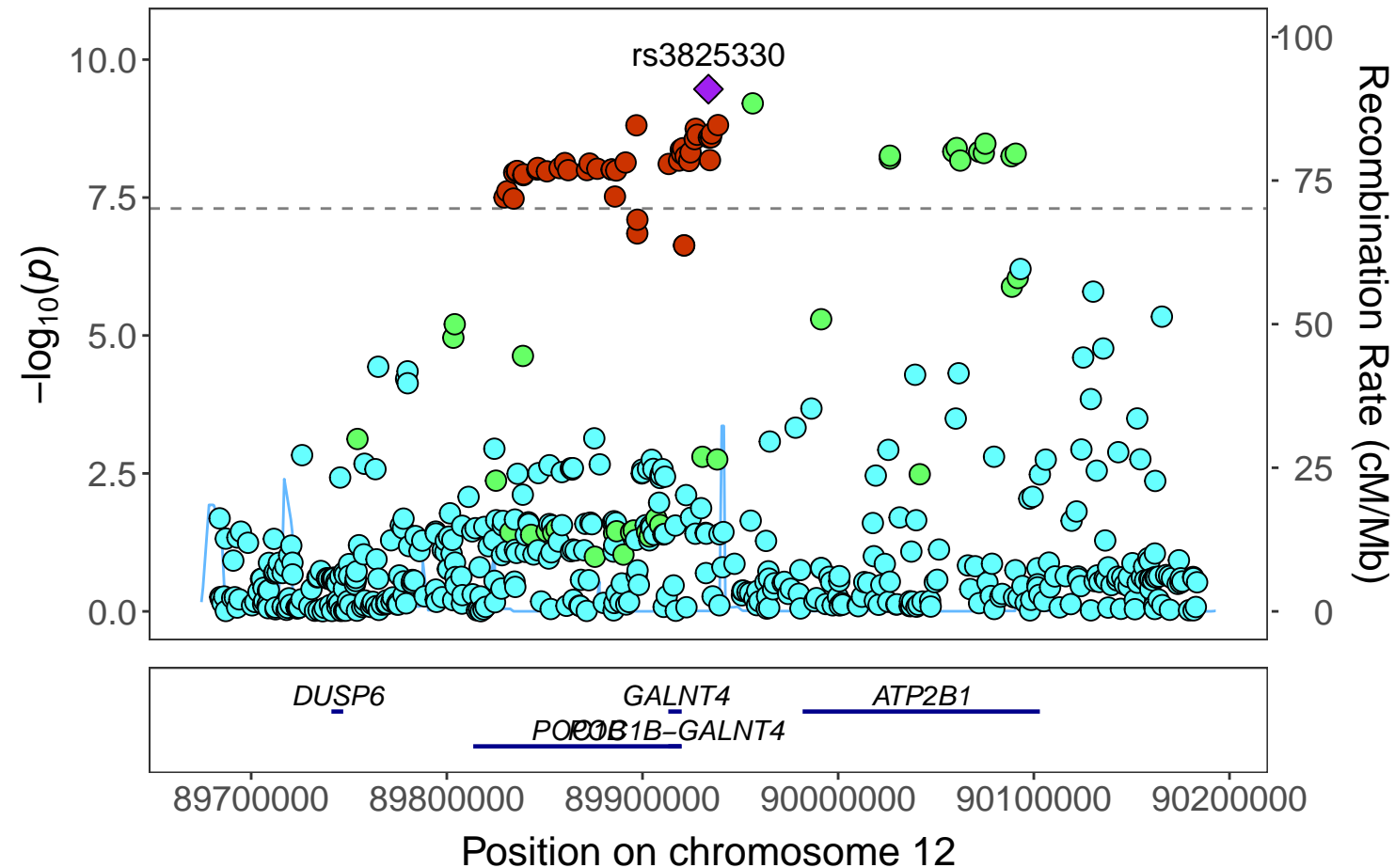

r2   miss   0.0–0.2   0.2–0.4   0.4–0.6   0.6–0.8   0.8–1.0

Supplement: Supplementary file 5 — Supporting Information [file CTM2-16-e70732-s001.zip › LocusZoom/Sfig_rs3825330_locusZoom.pdf]

# LocusZoom plots of GWAS top lead SNP

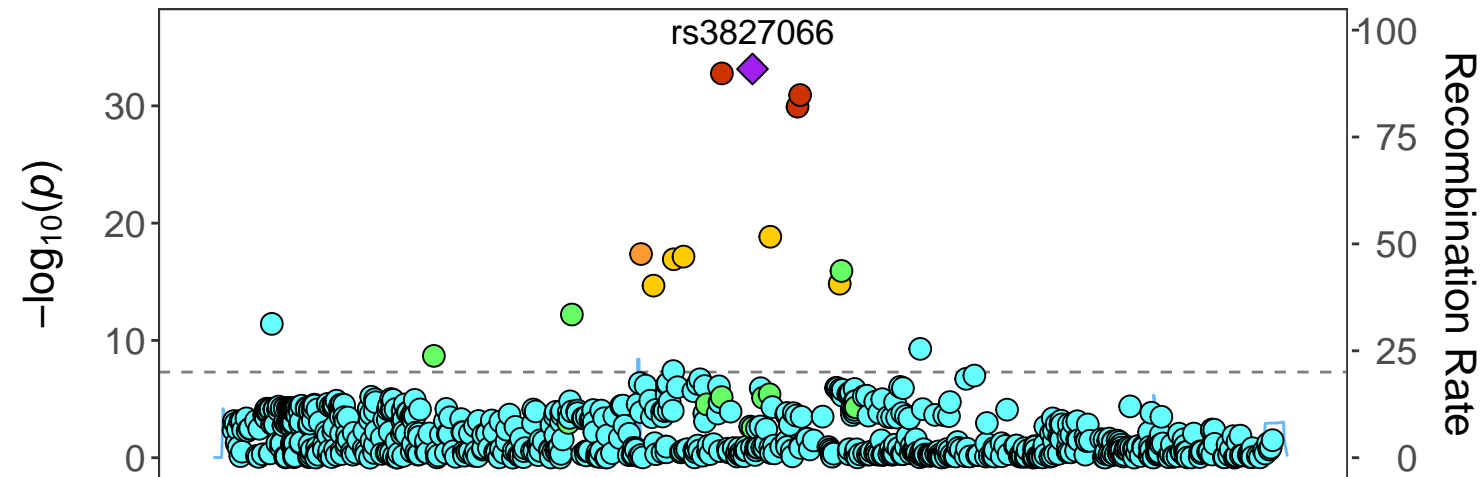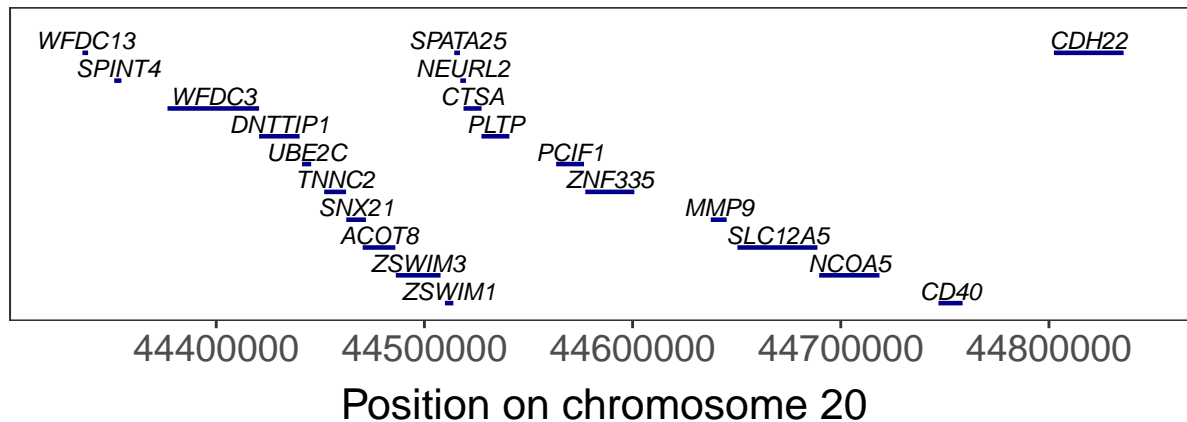

r2    miss    0.0–0.2    0.2–0.4    0.4–0.6    0.6–0.8    0.8–1.0

Supplement: Supplementary file 5 — Supporting Information [file CTM2-16-e70732-s001.zip › LocusZoom/Sfig_rs3827066_locusZoom.pdf]

# LocusZoom plots of GWAS top lead SNP

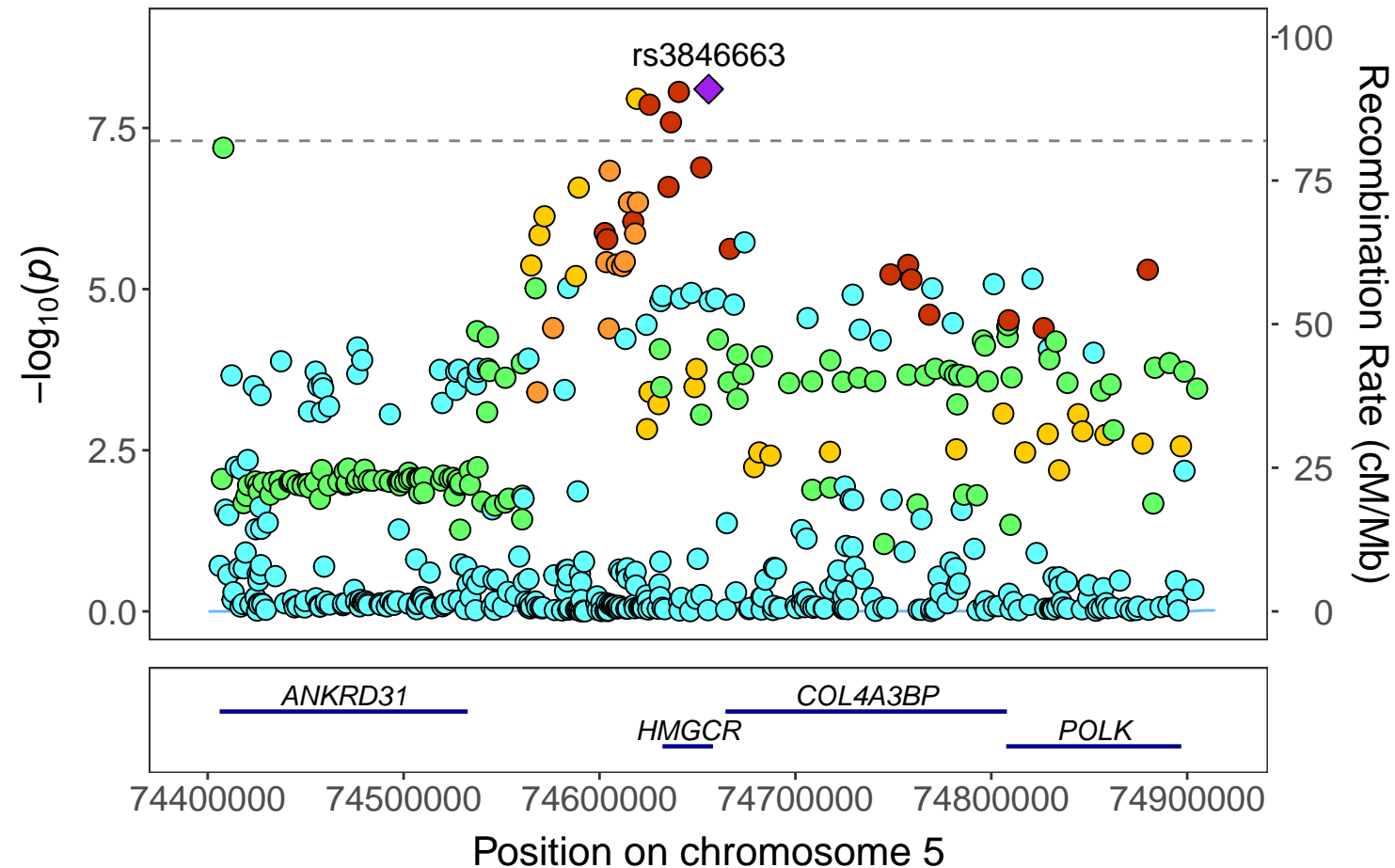

r2    miss    0.0-0.2    0.2-0.4    0.4-0.6    0.6-0.8    0.8-1.0

Supplement: Supplementary file 5 — Supporting Information [file CTM2-16-e70732-s001.zip › LocusZoom/Sfig_rs3846663_locusZoom.pdf]

# LocusZoom plots of GWAS top lead SNP

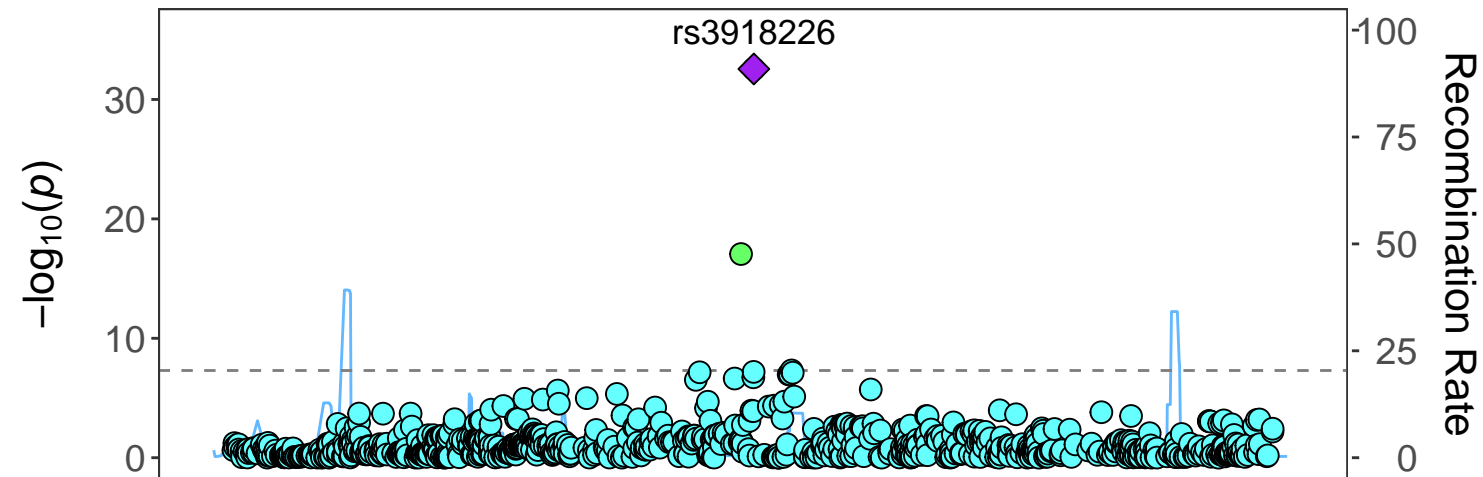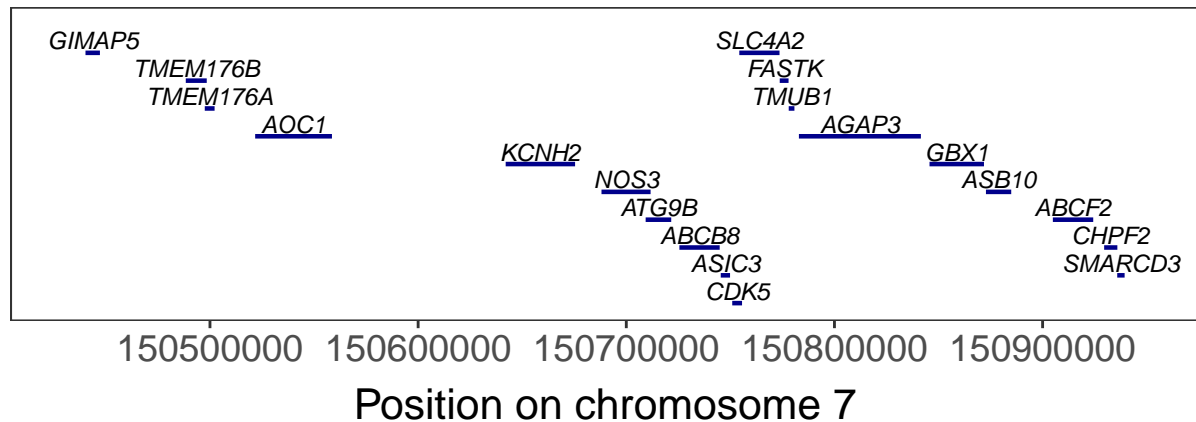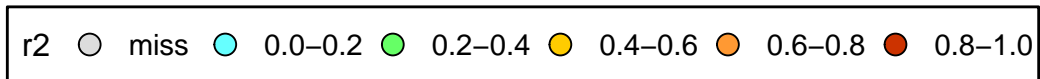

Supplement: Supplementary file 5 — Supporting Information [file CTM2-16-e70732-s001.zip › LocusZoom/Sfig_rs3918226_locusZoom.pdf]

# *LocusZoom plots of GWAS top lead SNP*

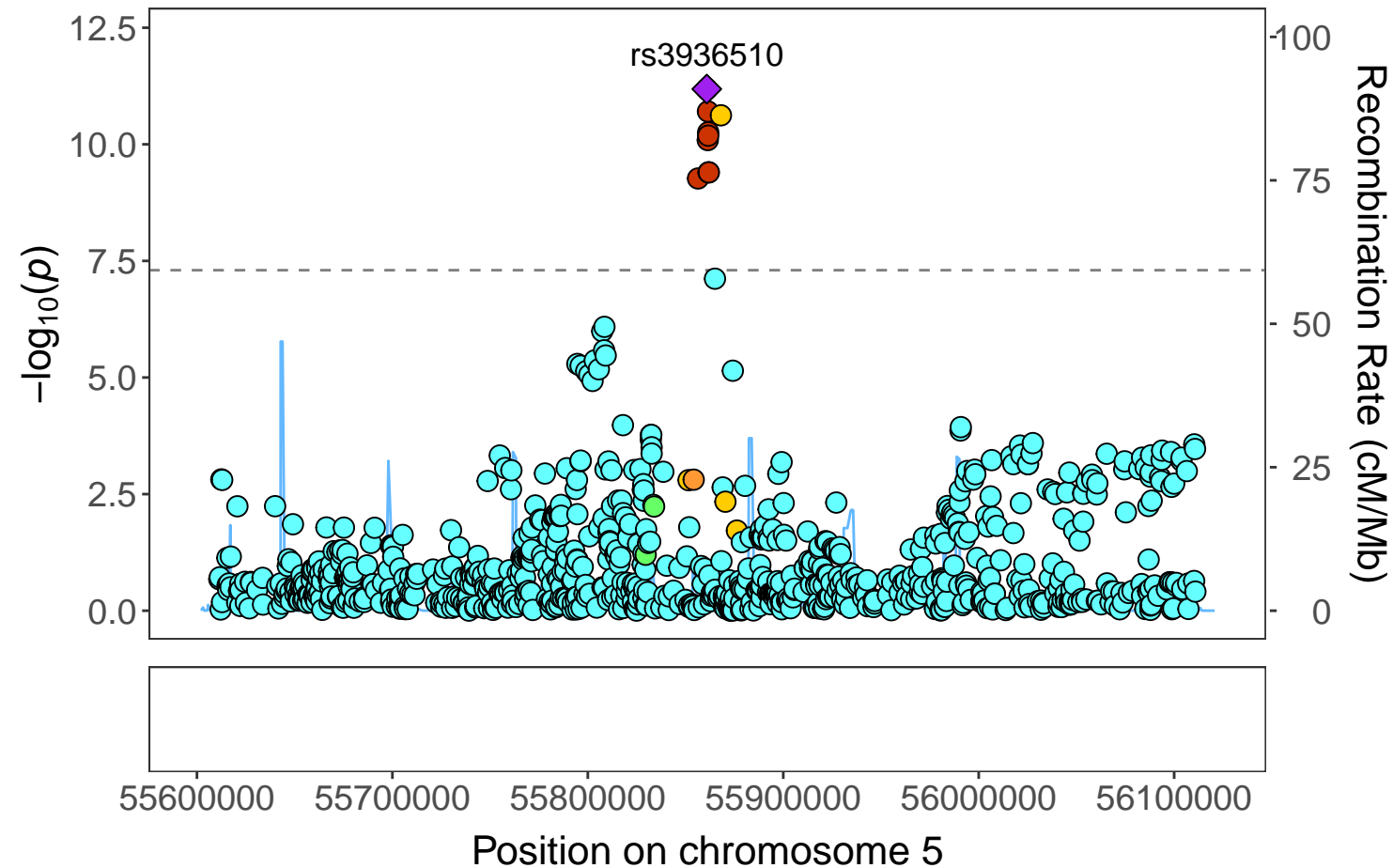

r2   miss   0.0–0.2   0.2–0.4   0.4–0.6   0.6–0.8   0.8–1.0

Supplement: Supplementary file 5 — Supporting Information [file CTM2-16-e70732-s001.zip › LocusZoom/Sfig_rs3936510_locusZoom.pdf]

# *LocusZoom plots of GWAS top lead SNP*

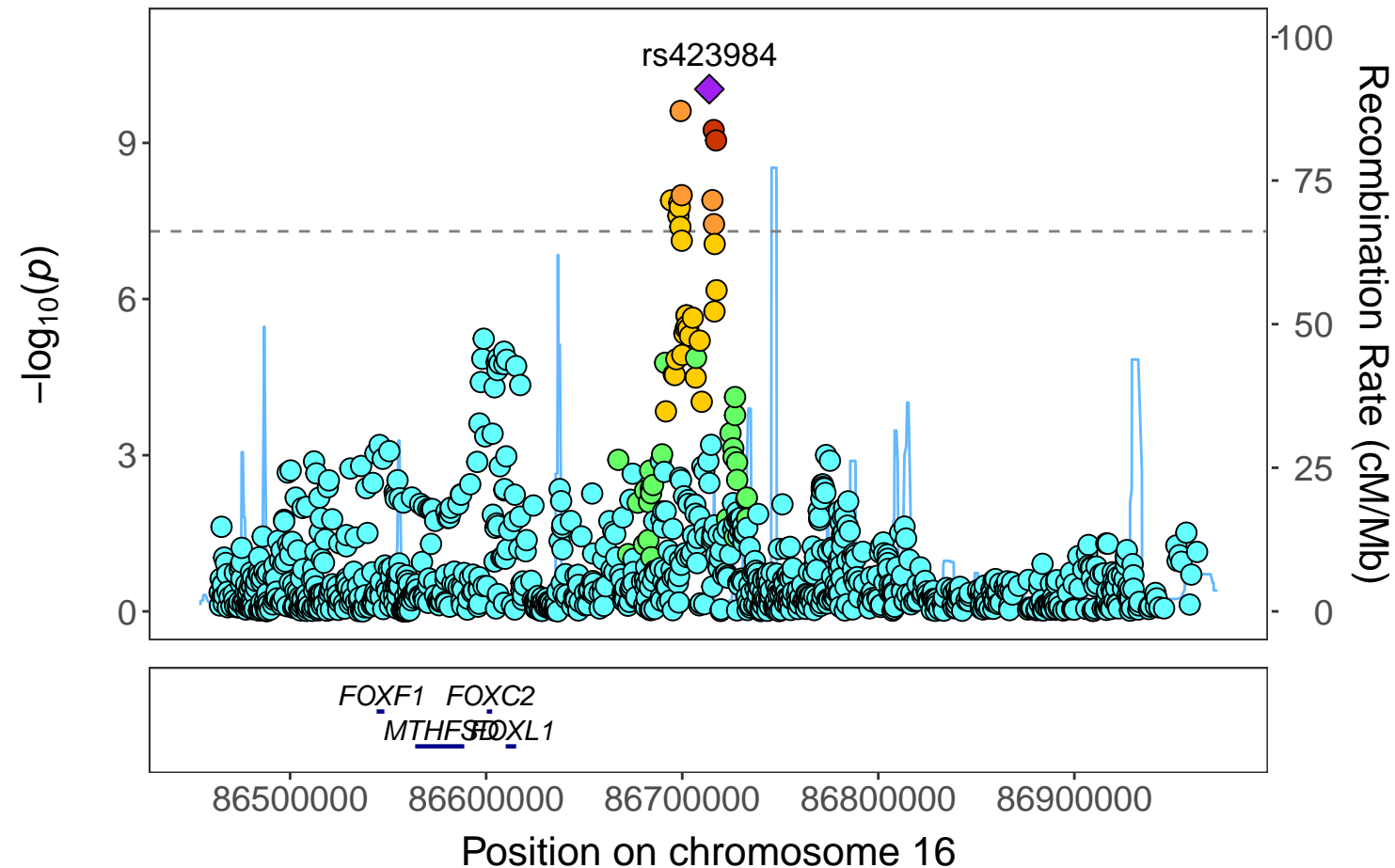

$r^2$  ○ miss ○ 0.0–0.2 ○ 0.2–0.4 ○ 0.4–0.6 ○ 0.6–0.8 ○ 0.8–1.0

Supplement: Supplementary file 5 — Supporting Information [file CTM2-16-e70732-s001.zip › LocusZoom/Sfig_rs423984_locusZoom.pdf]

# LocusZoom plots of GWAS top lead SNP

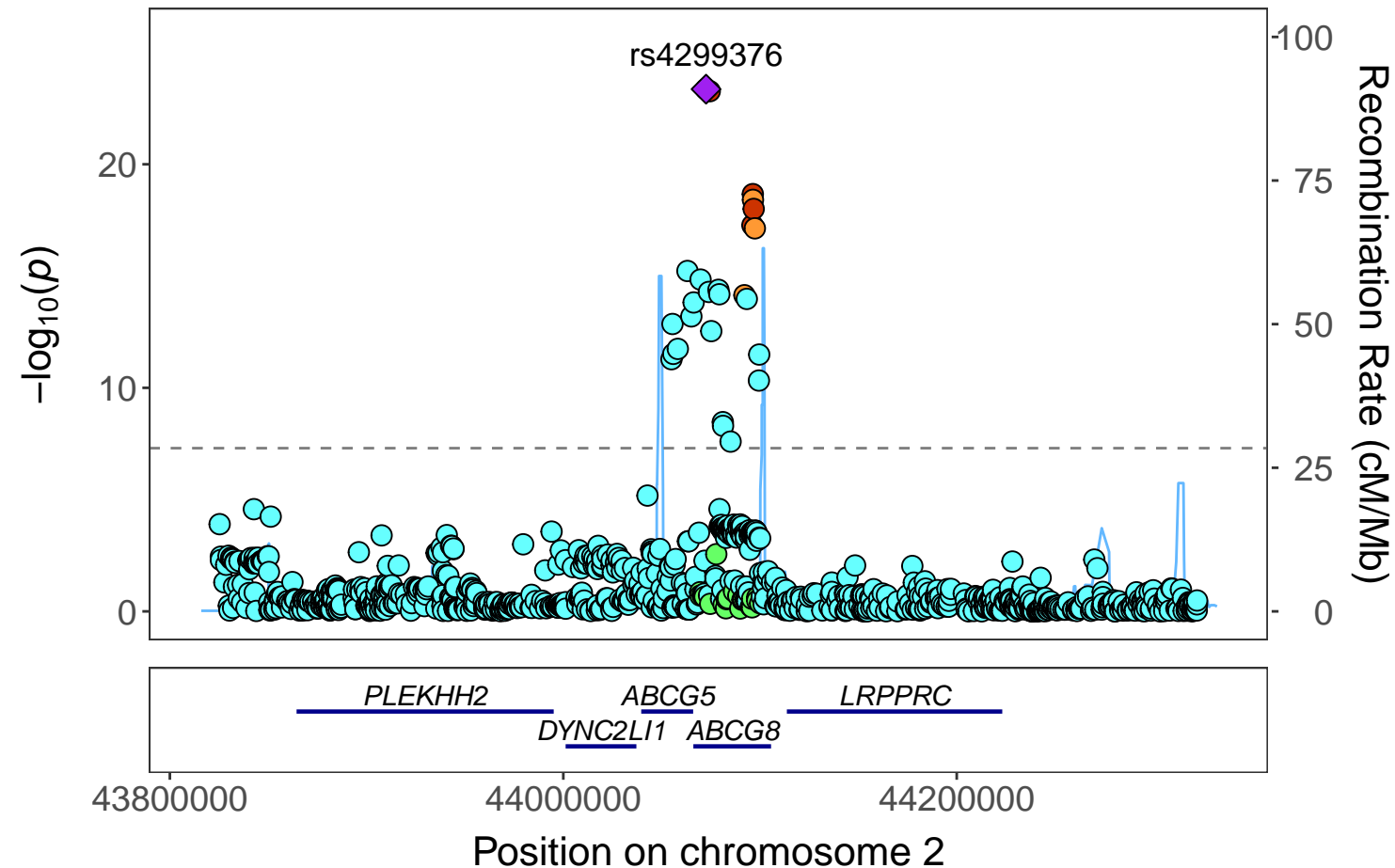

$r^2$    miss   0.0–0.2   0.2–0.4   0.4–0.6   0.6–0.8   0.8–1.0

Supplement: Supplementary file 5 — Supporting Information [file CTM2-16-e70732-s001.zip › LocusZoom/Sfig_rs4299376_locusZoom.pdf]

# LocusZoom plots of GWAS top lead SNP

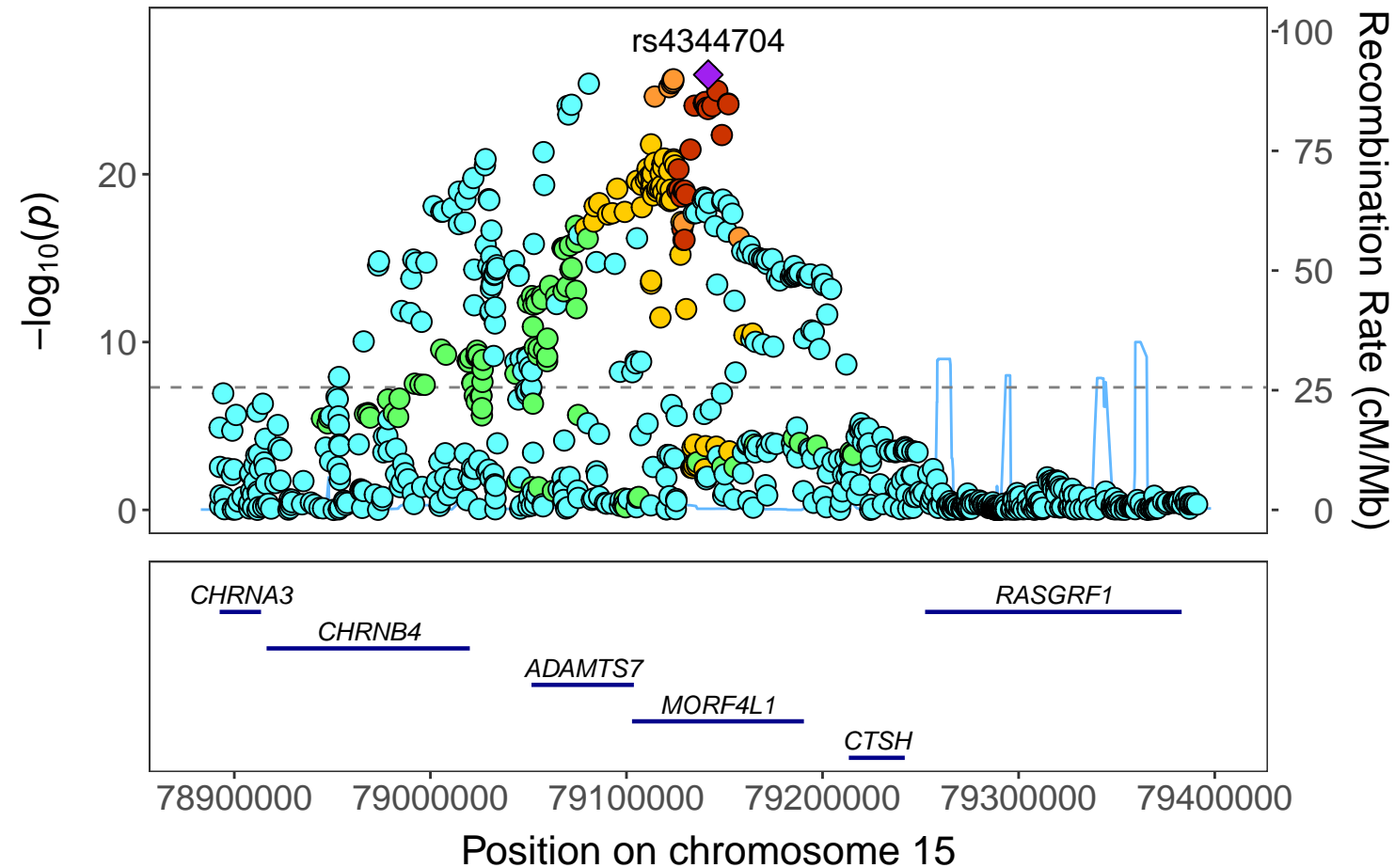

r2   miss   0.0–0.2   0.2–0.4   0.4–0.6   0.6–0.8   0.8–1.0

Supplement: Supplementary file 5 — Supporting Information [file CTM2-16-e70732-s001.zip › LocusZoom/Sfig_rs4344704_locusZoom.pdf]

# LocusZoom plots of GWAS top lead SNP

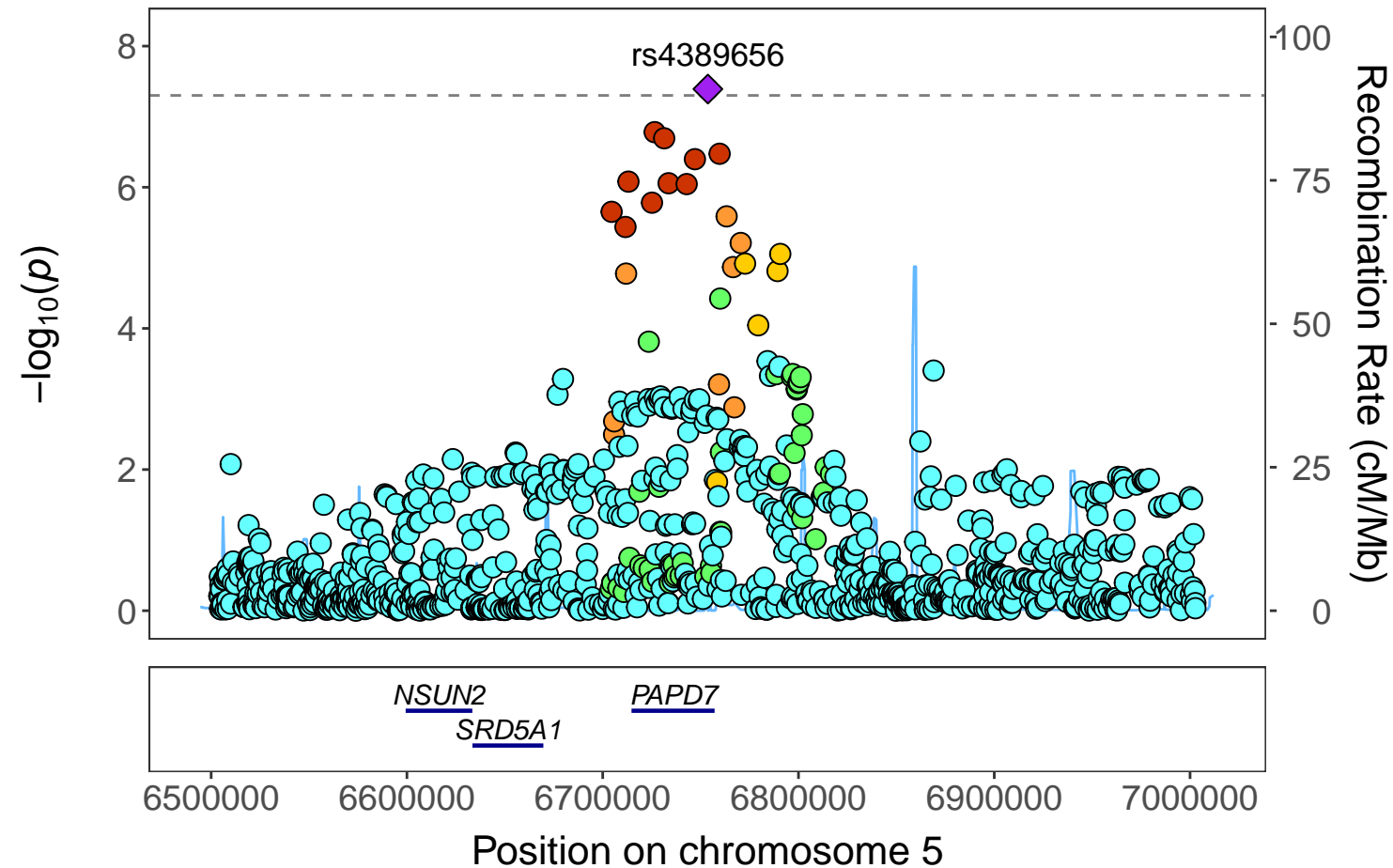

$r^2$    miss   0.0-0.2   0.2-0.4   0.4-0.6   0.6-0.8   0.8-1.0

Supplement: Supplementary file 5 — Supporting Information [file CTM2-16-e70732-s001.zip › LocusZoom/Sfig_rs4389656_locusZoom.pdf]

# LocusZoom plots of GWAS top lead SNP

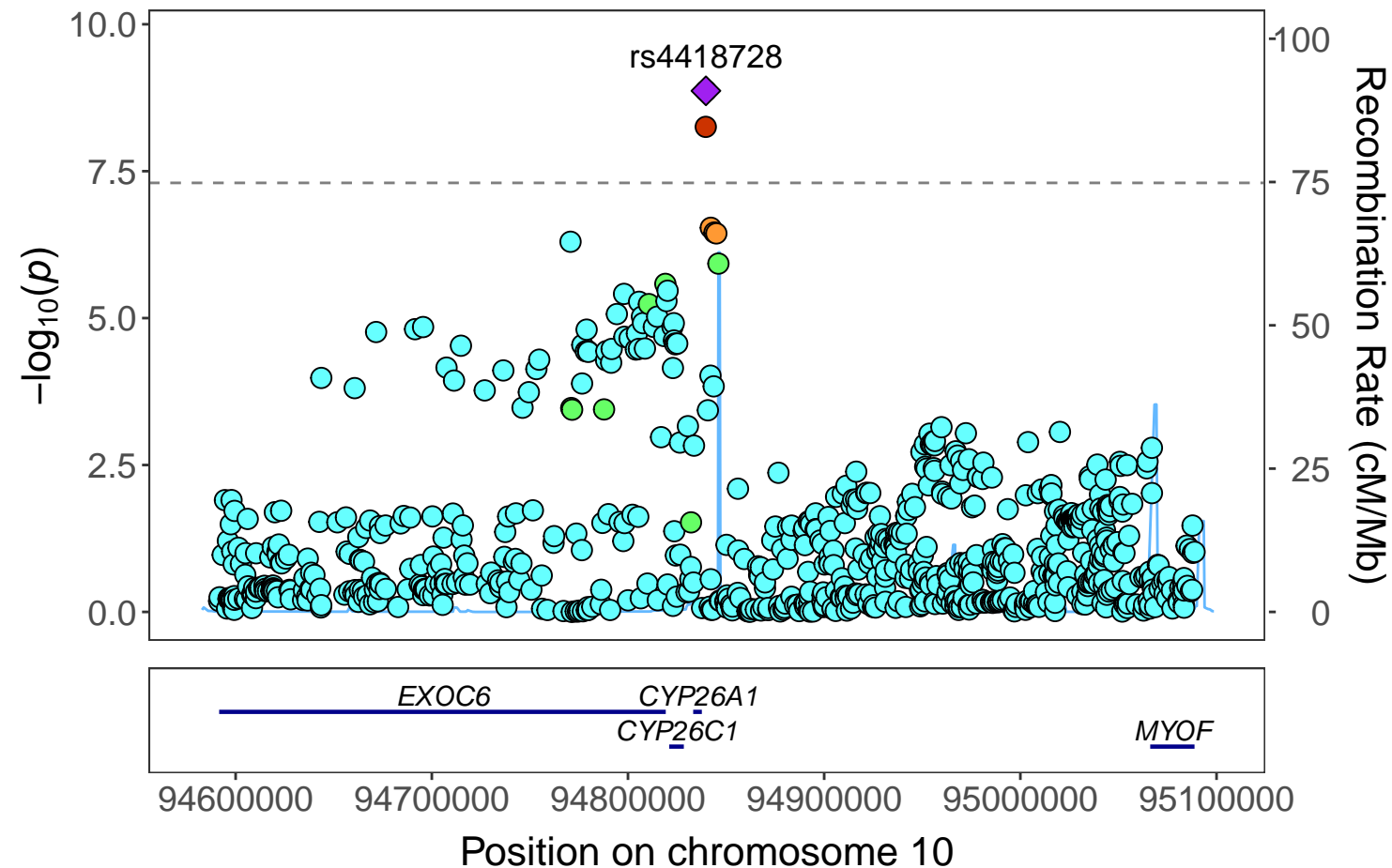

$r^2$  ○ miss ● 0.0–0.2 ● 0.2–0.4 ● 0.4–0.6 ● 0.6–0.8 ● 0.8–1.0

Supplement: Supplementary file 5 — Supporting Information [file CTM2-16-e70732-s001.zip › LocusZoom/Sfig_rs4418728_locusZoom.pdf]

# LocusZoom plots of GWAS top lead SNP

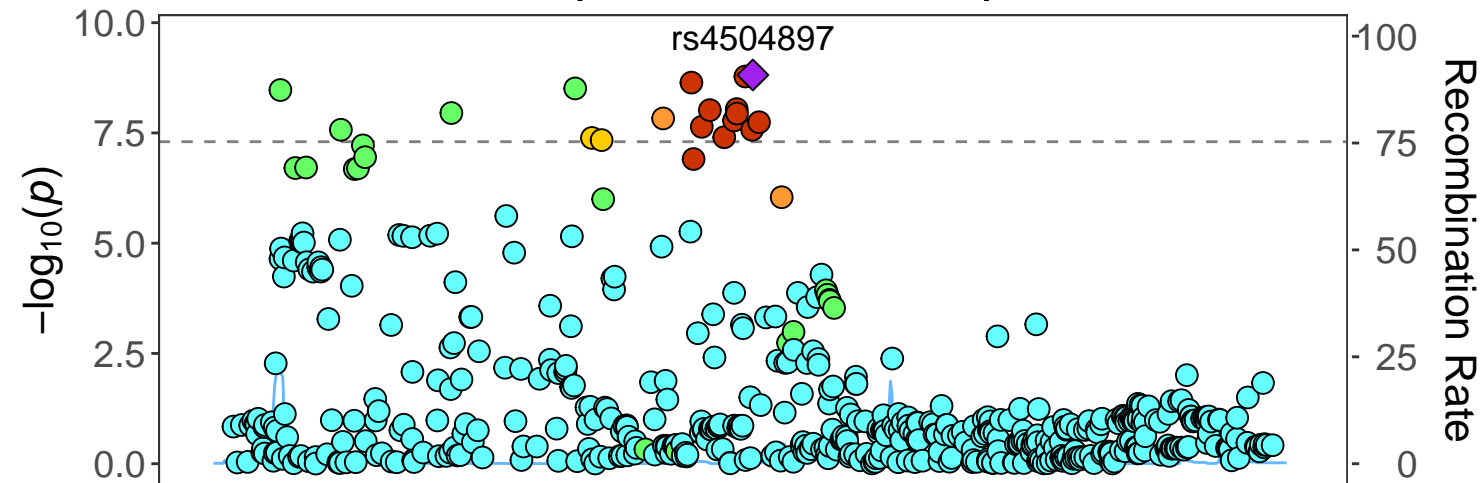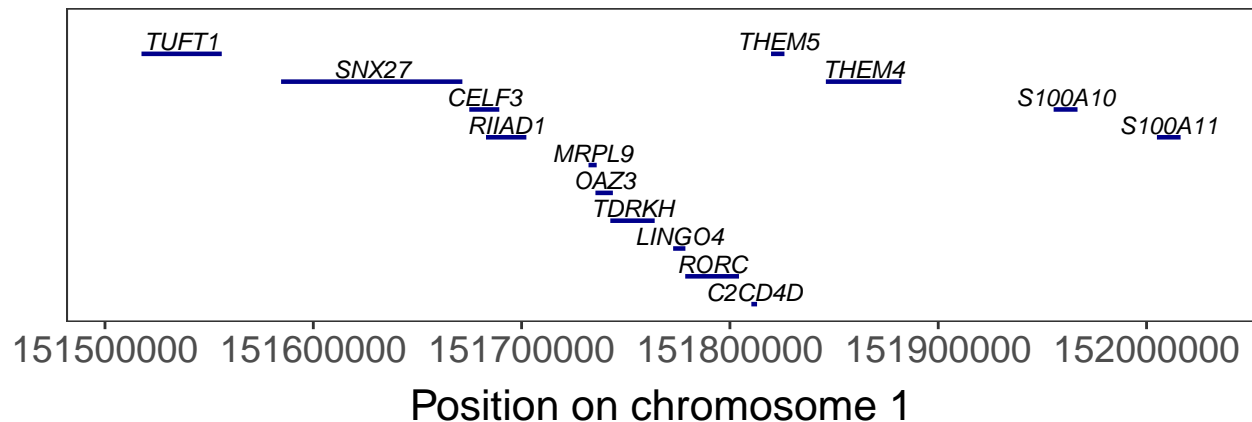

r2   miss   0.0–0.2   0.2–0.4   0.4–0.6   0.6–0.8   0.8–1.0

Supplement: Supplementary file 5 — Supporting Information [file CTM2-16-e70732-s001.zip › LocusZoom/Sfig_rs4504897_locusZoom.pdf]

# *LocusZoom plots of GWAS top lead SNP*

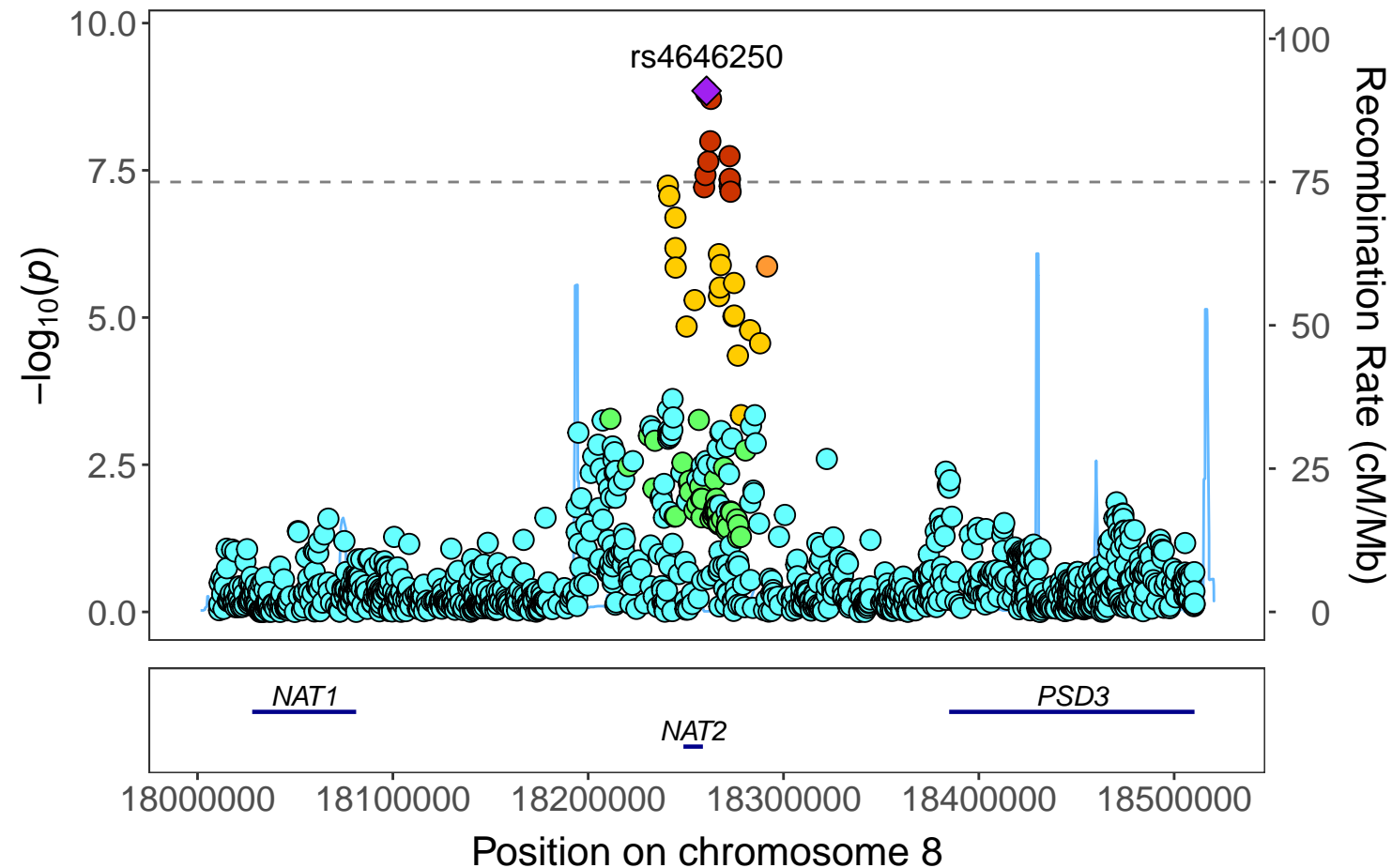

$r^2$    miss   0.0–0.2   0.2–0.4   0.4–0.6   0.6–0.8   0.8–1.0

Supplement: Supplementary file 5 — Supporting Information [file CTM2-16-e70732-s001.zip › LocusZoom/Sfig_rs4646250_locusZoom.pdf]

# LocusZoom plots of GWAS top lead SNP

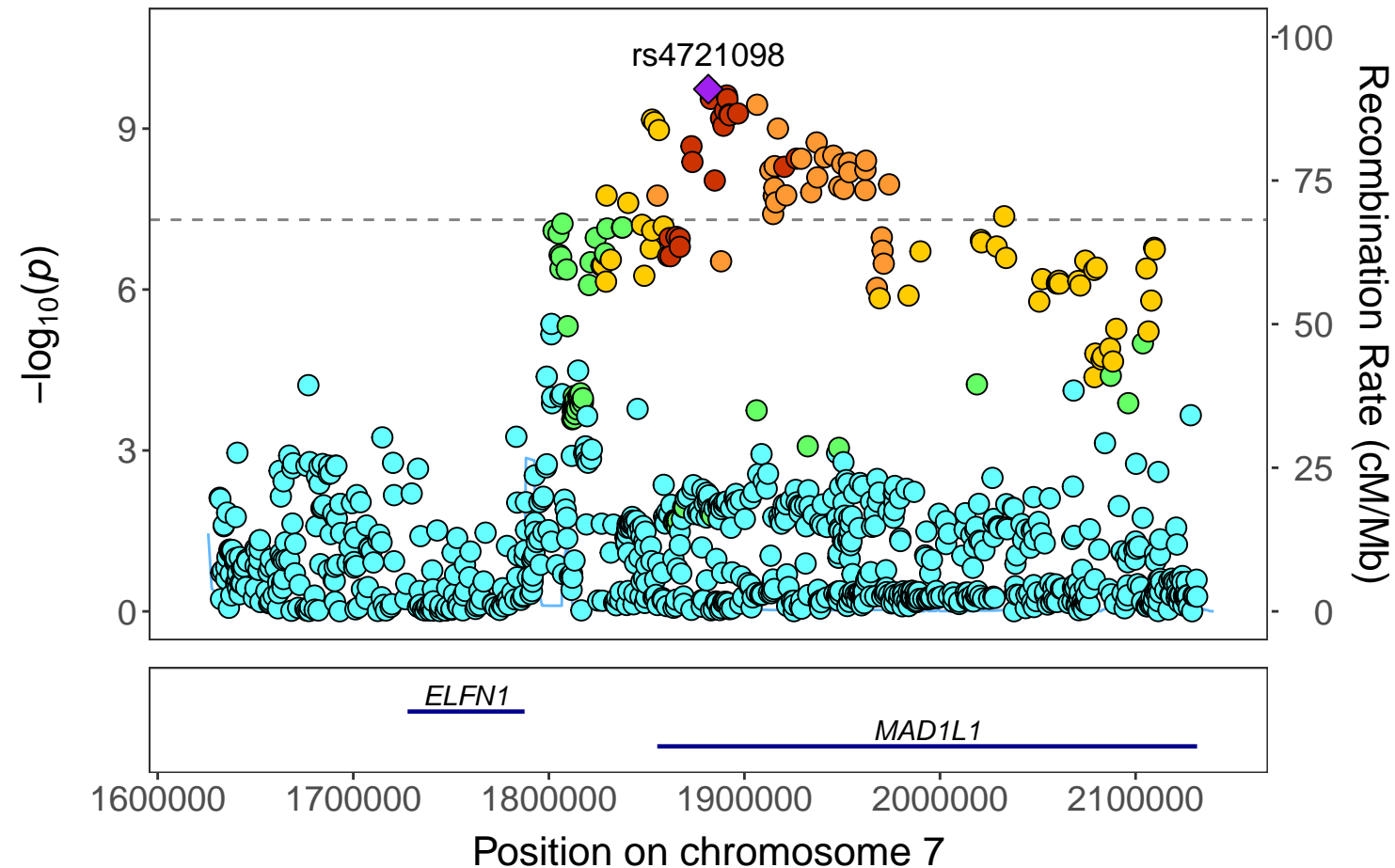

r2    miss    0.0–0.2    0.2–0.4    0.4–0.6    0.6–0.8    0.8–1.0

Supplement: Supplementary file 5 — Supporting Information [file CTM2-16-e70732-s001.zip › LocusZoom/Sfig_rs4721098_locusZoom.pdf]

# LocusZoom plots of GWAS top lead SNP

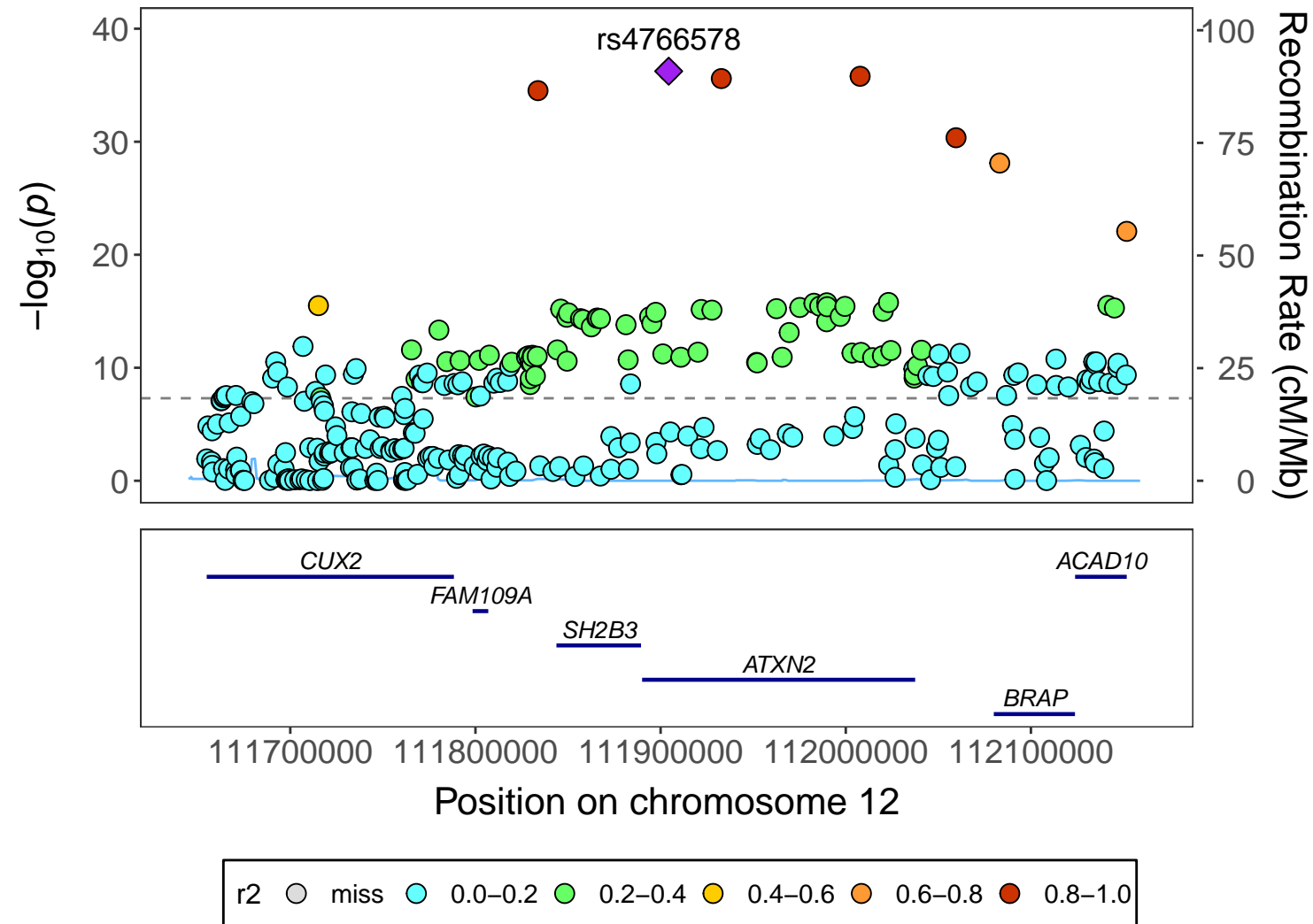

Supplement: Supplementary file 5 — Supporting Information [file CTM2-16-e70732-s001.zip › LocusZoom/Sfig_rs4766578_locusZoom.pdf]

# *LocusZoom plots of GWAS top lead SNP*

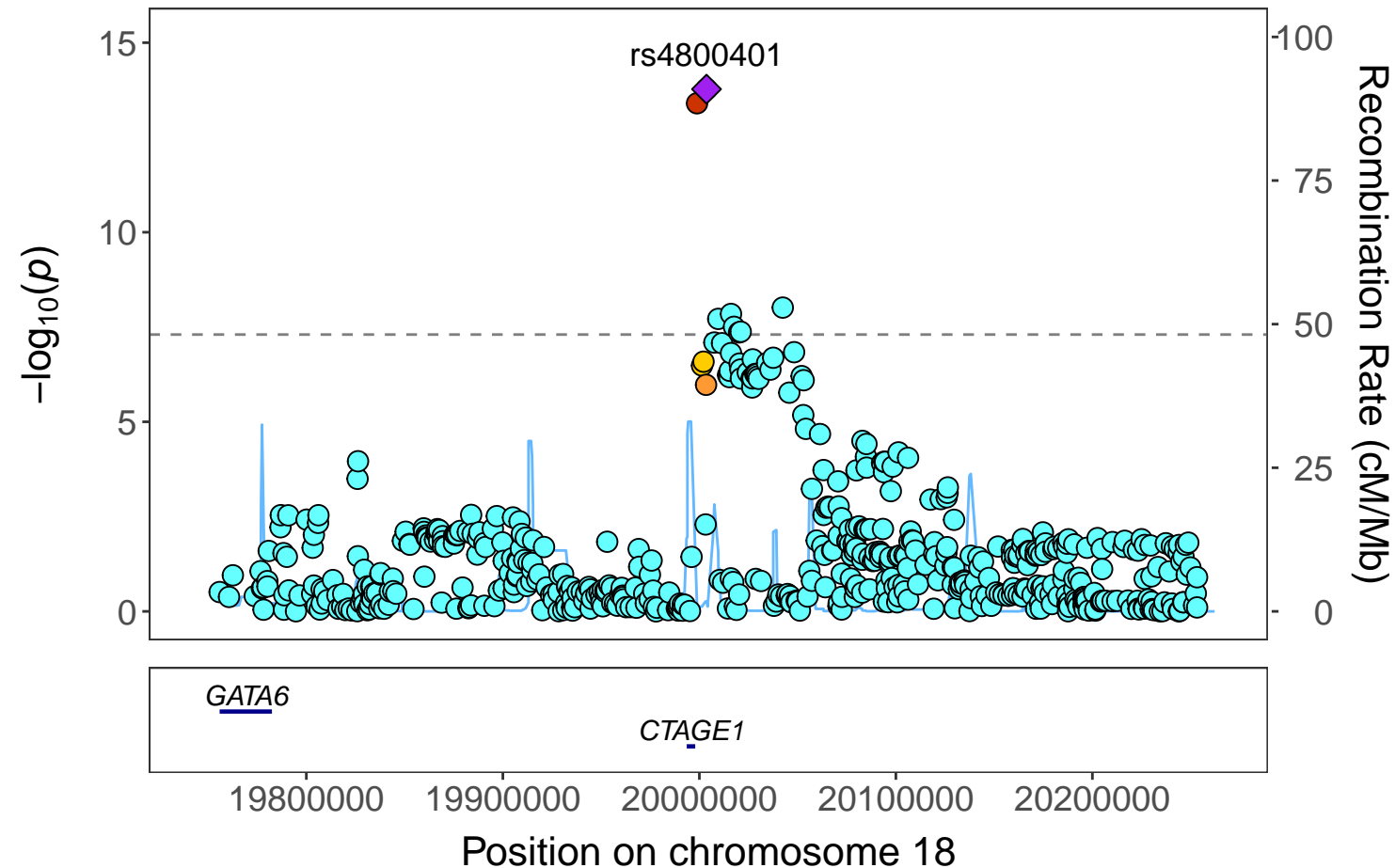

$r^2$    miss   cyan   0.0–0.2   green   0.2–0.4   yellow   0.4–0.6   orange   0.6–0.8   red   0.8–1.0

Supplement: Supplementary file 5 — Supporting Information [file CTM2-16-e70732-s001.zip › LocusZoom/Sfig_rs4800401_locusZoom.pdf]

# LocusZoom plots of GWAS top lead SNP

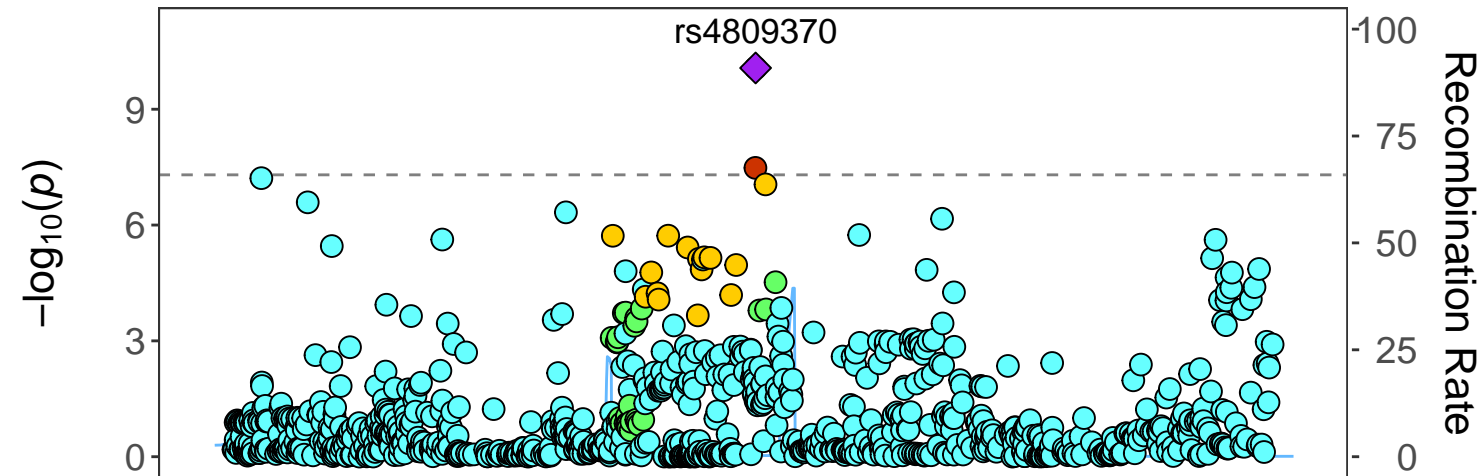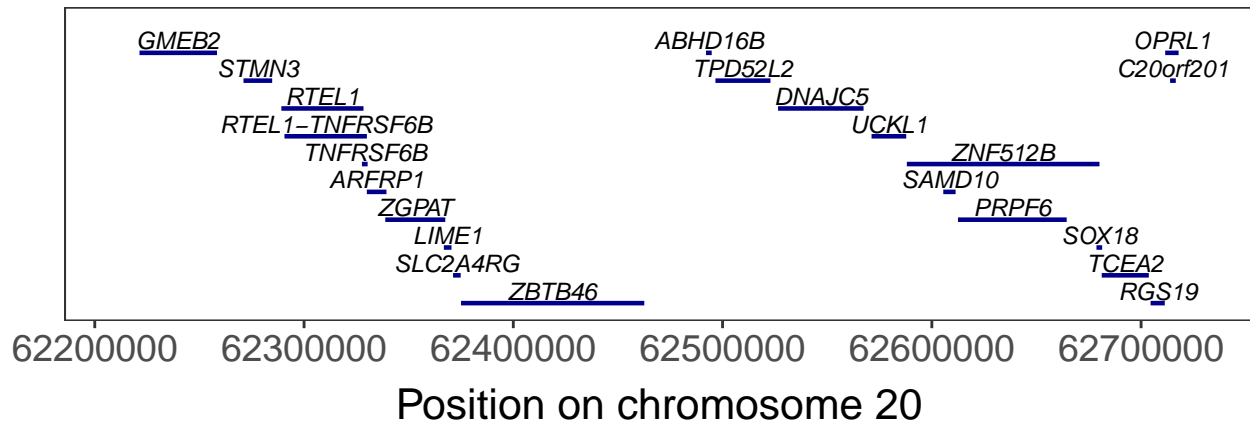

r2    miss    0.0-0.2    0.2-0.4    0.4-0.6    0.6-0.8    0.8-1.0

Supplement: Supplementary file 5 — Supporting Information [file CTM2-16-e70732-s001.zip › LocusZoom/Sfig_rs4809370_locusZoom.pdf]

# LocusZoom plots of GWAS top lead SNP

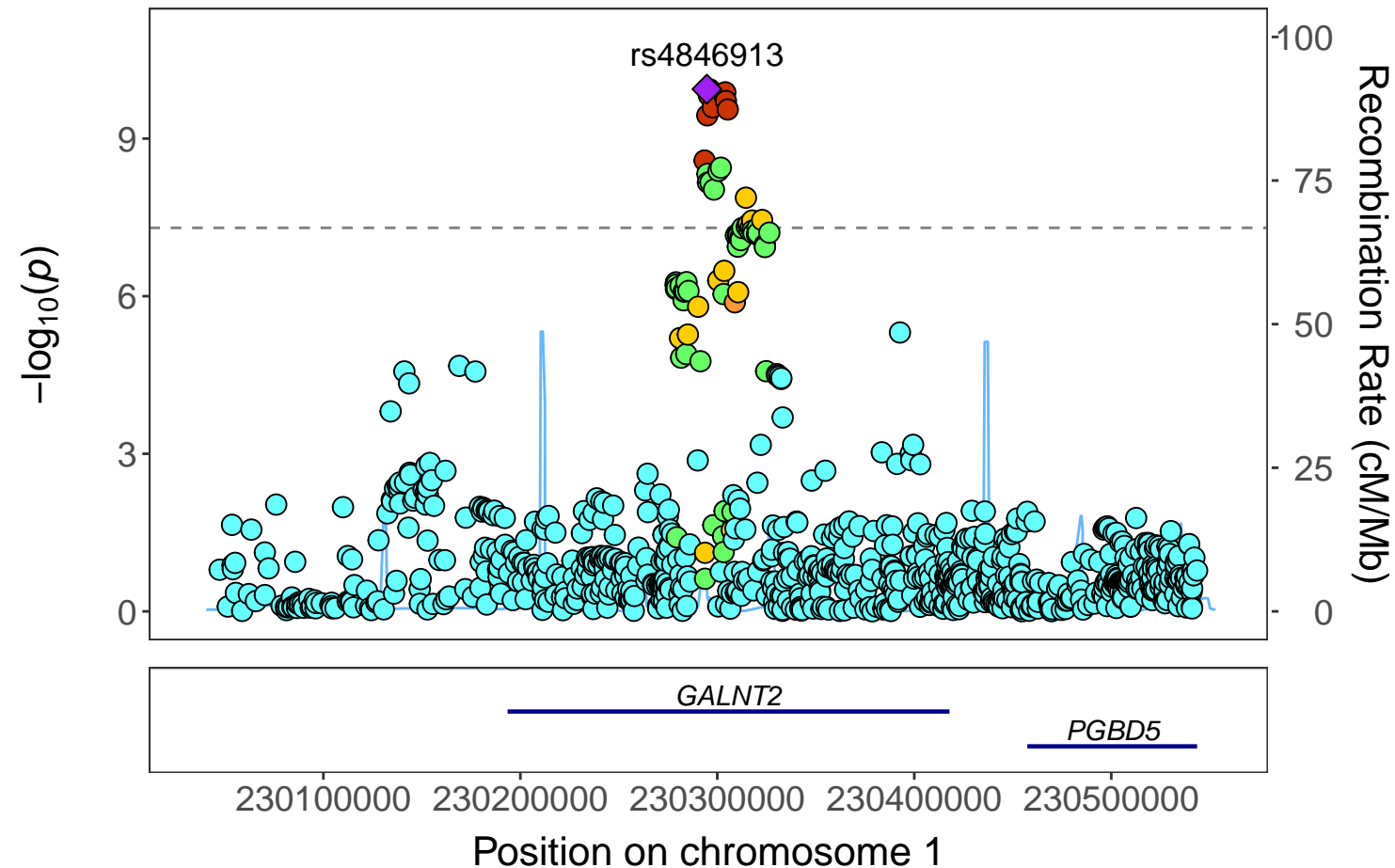

r2    miss    0.0-0.2    0.2-0.4    0.4-0.6    0.6-0.8    0.8-1.0

Supplement: Supplementary file 5 — Supporting Information [file CTM2-16-e70732-s001.zip › LocusZoom/Sfig_rs4846913_locusZoom.pdf]

# LocusZoom plots of GWAS top lead SNP

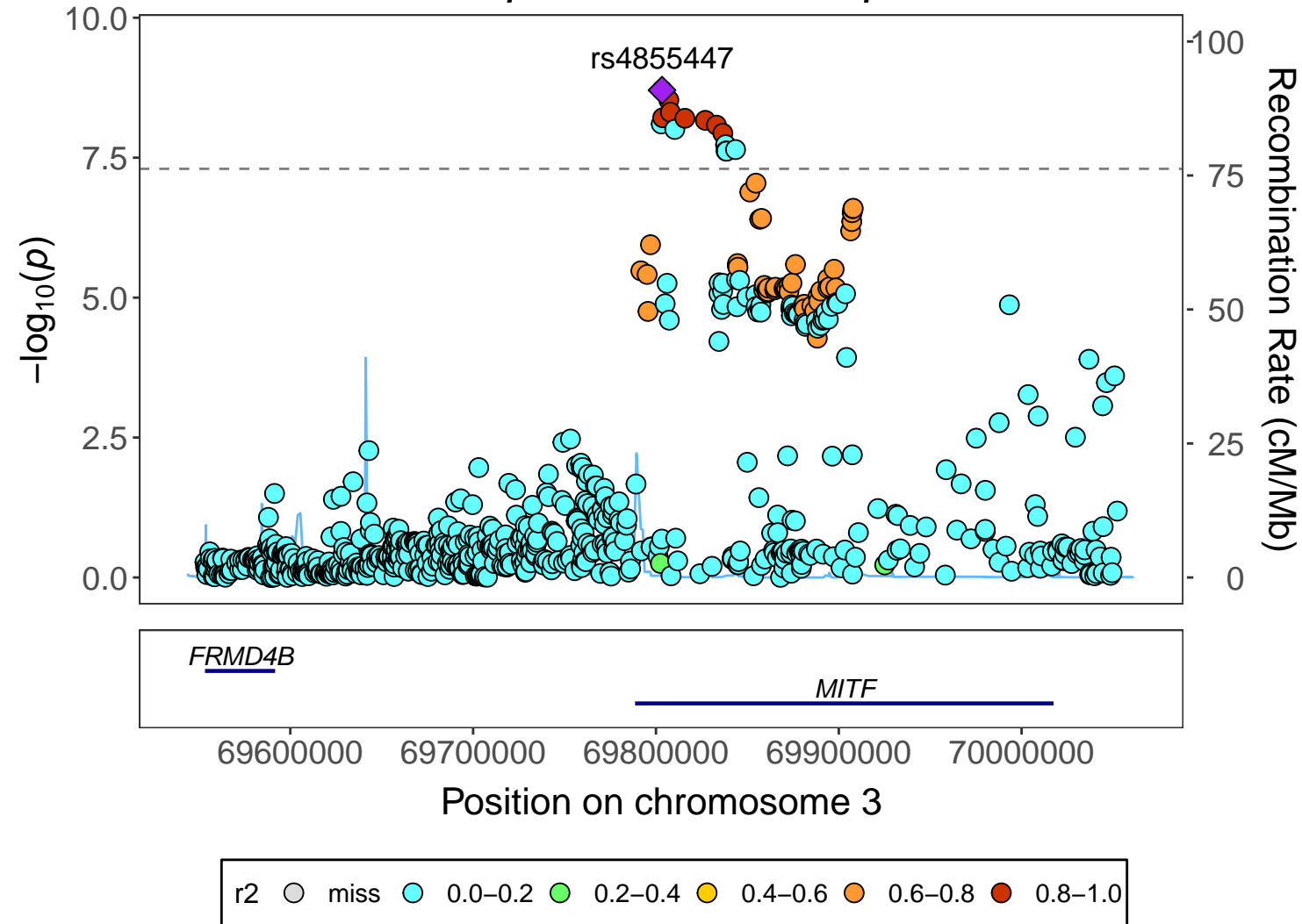

Supplement: Supplementary file 5 — Supporting Information [file CTM2-16-e70732-s001.zip › LocusZoom/Sfig_rs4855447_locusZoom.pdf]

# LocusZoom plots of GWAS top lead SNP

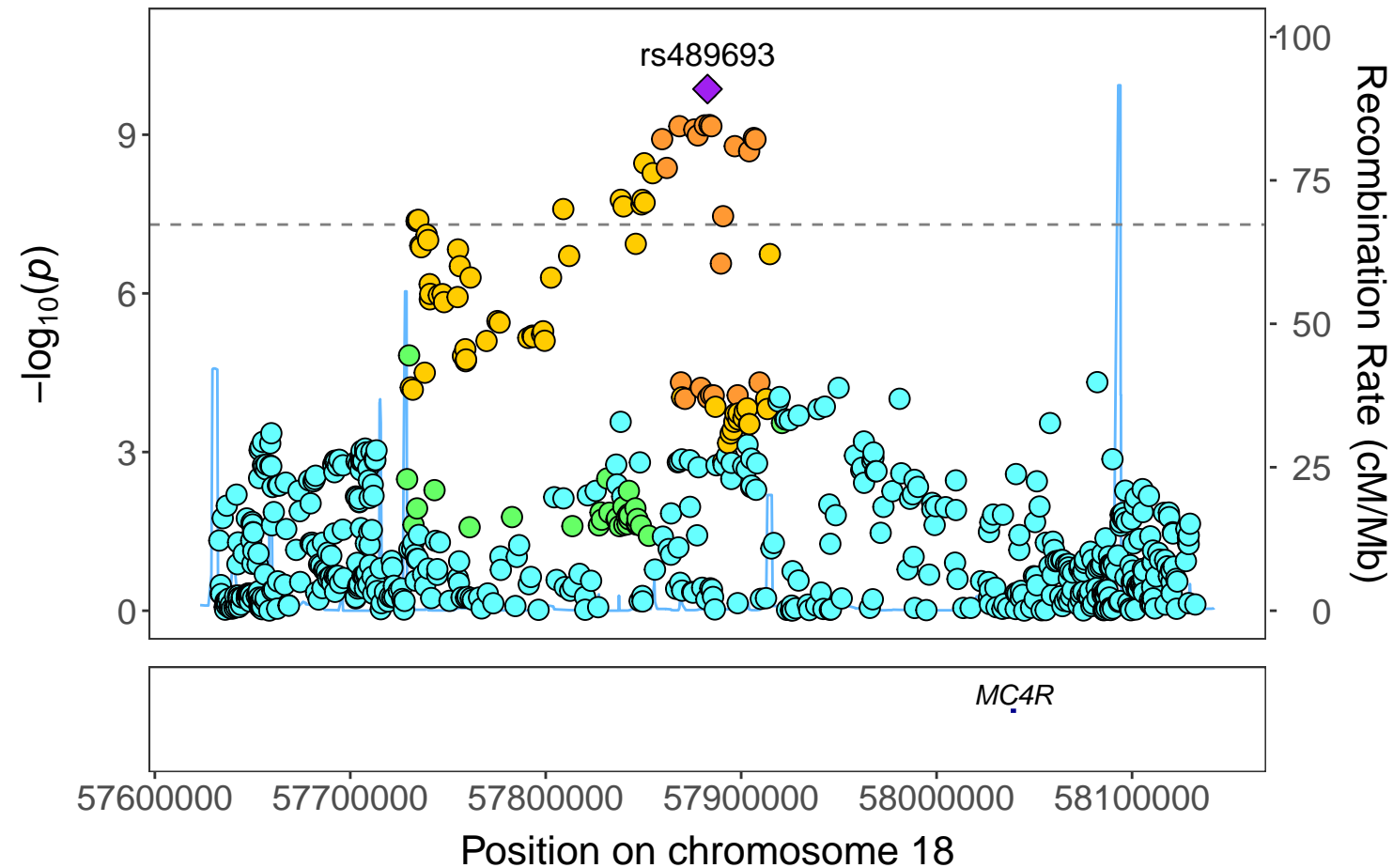

Supplement: Supplementary file 5 — Supporting Information [file CTM2-16-e70732-s001.zip › LocusZoom/Sfig_rs489693_locusZoom.pdf]

# LocusZoom plots of GWAS top lead SNP

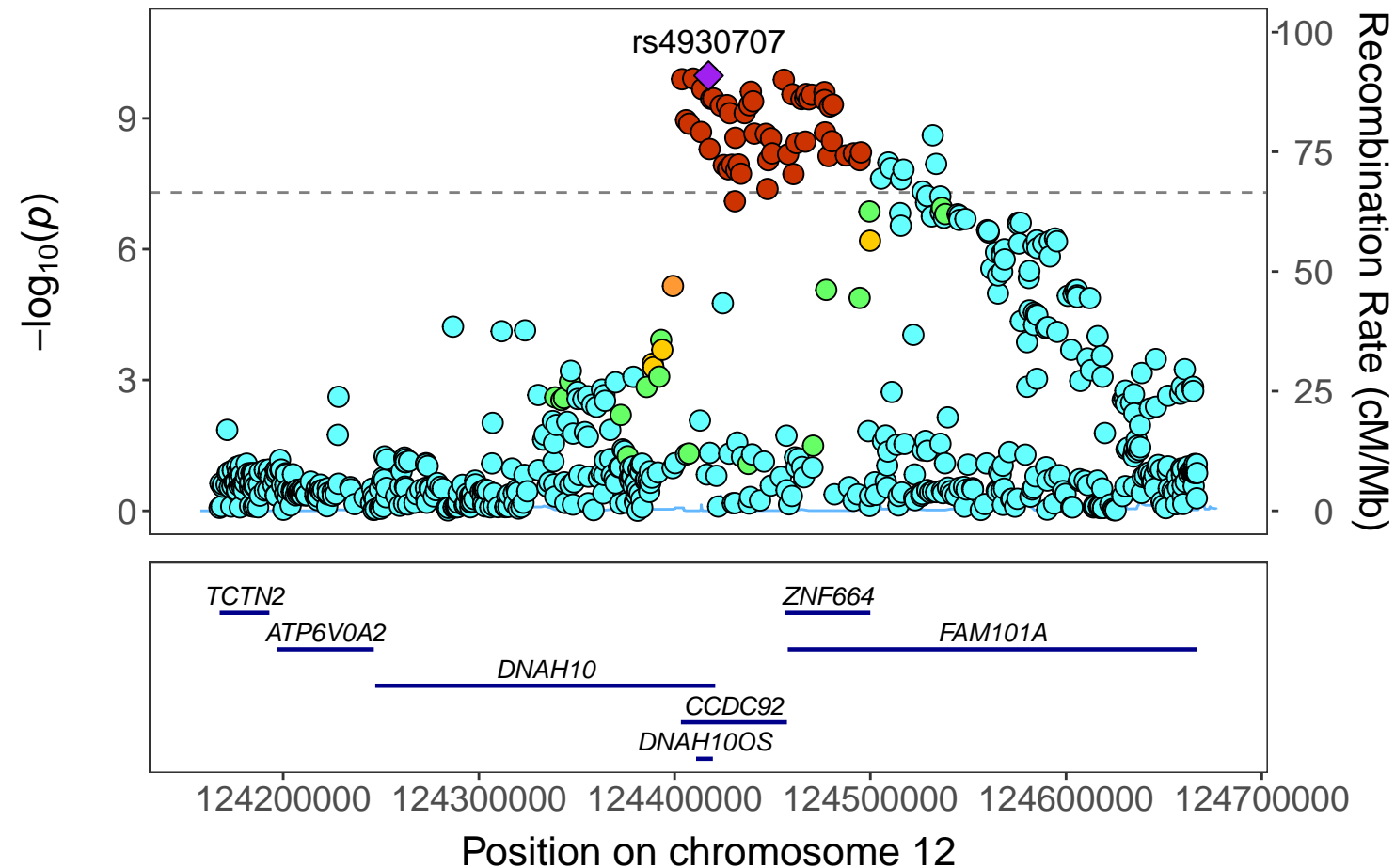

r2   miss   0.0–0.2   0.2–0.4   0.4–0.6   0.6–0.8   0.8–1.0

Supplement: Supplementary file 5 — Supporting Information [file CTM2-16-e70732-s001.zip › LocusZoom/Sfig_rs4930707_locusZoom.pdf]

# LocusZoom plots of GWAS top lead SNP

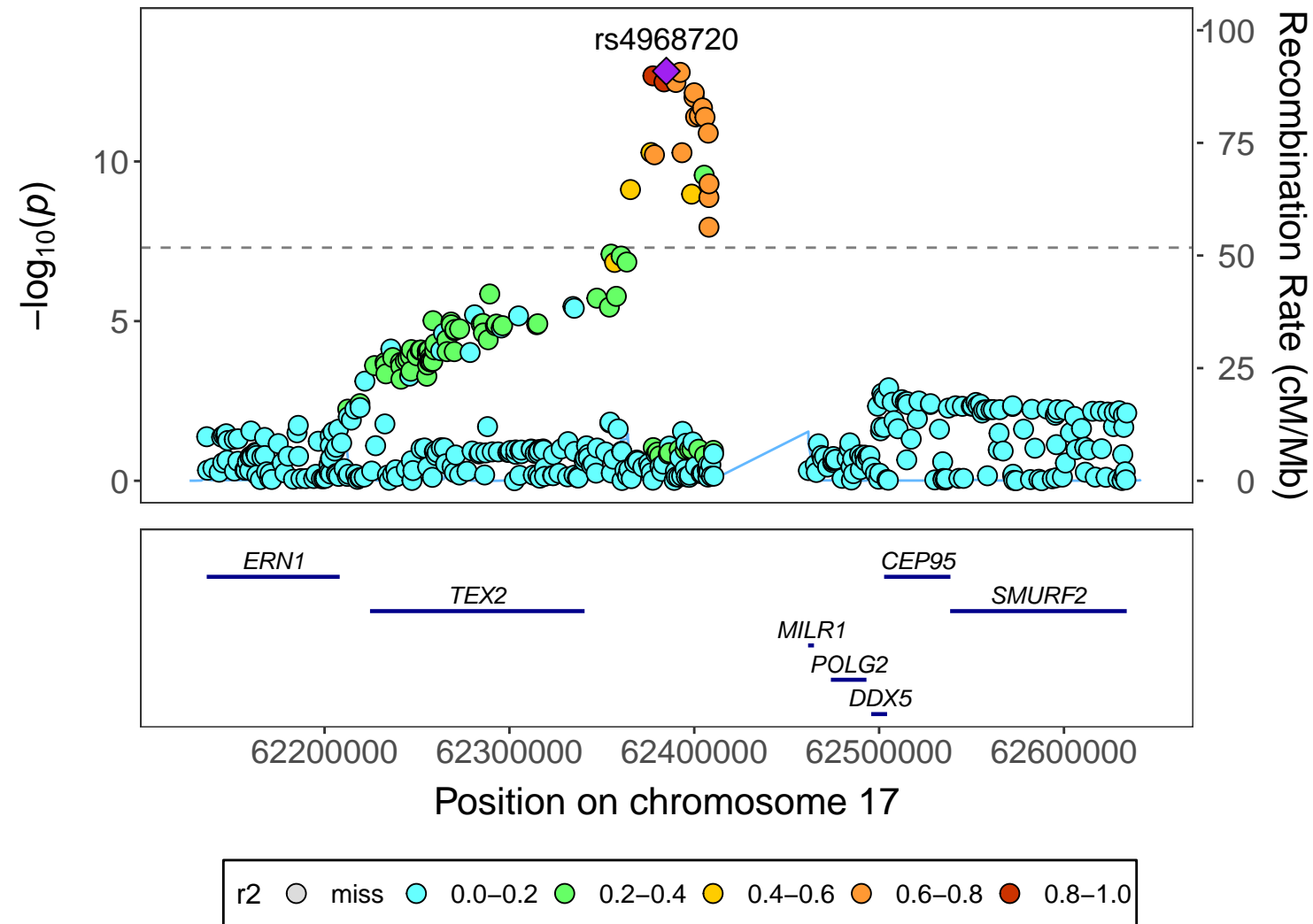

Supplement: Supplementary file 5 — Supporting Information [file CTM2-16-e70732-s001.zip › LocusZoom/Sfig_rs4968720_locusZoom.pdf]

# LocusZoom plots of GWAS top lead SNP

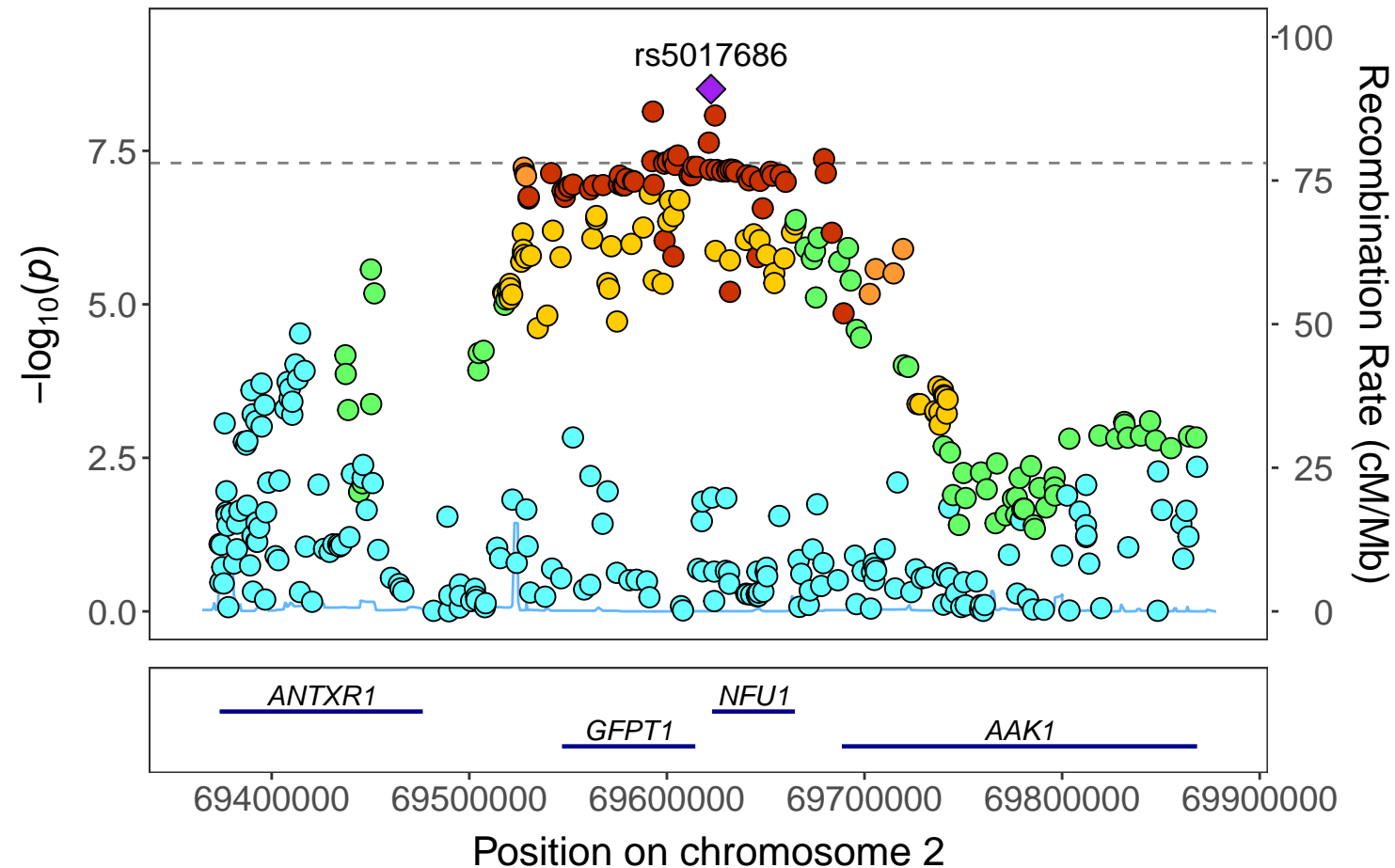

$r^2$  ○ miss ○ 0.0–0.2 ○ 0.2–0.4 ○ 0.4–0.6 ○ 0.6–0.8 ○ 0.8–1.0

Supplement: Supplementary file 5 — Supporting Information [file CTM2-16-e70732-s001.zip › LocusZoom/Sfig_rs5017686_locusZoom.pdf]

# *LocusZoom plots of GWAS top lead SNP*

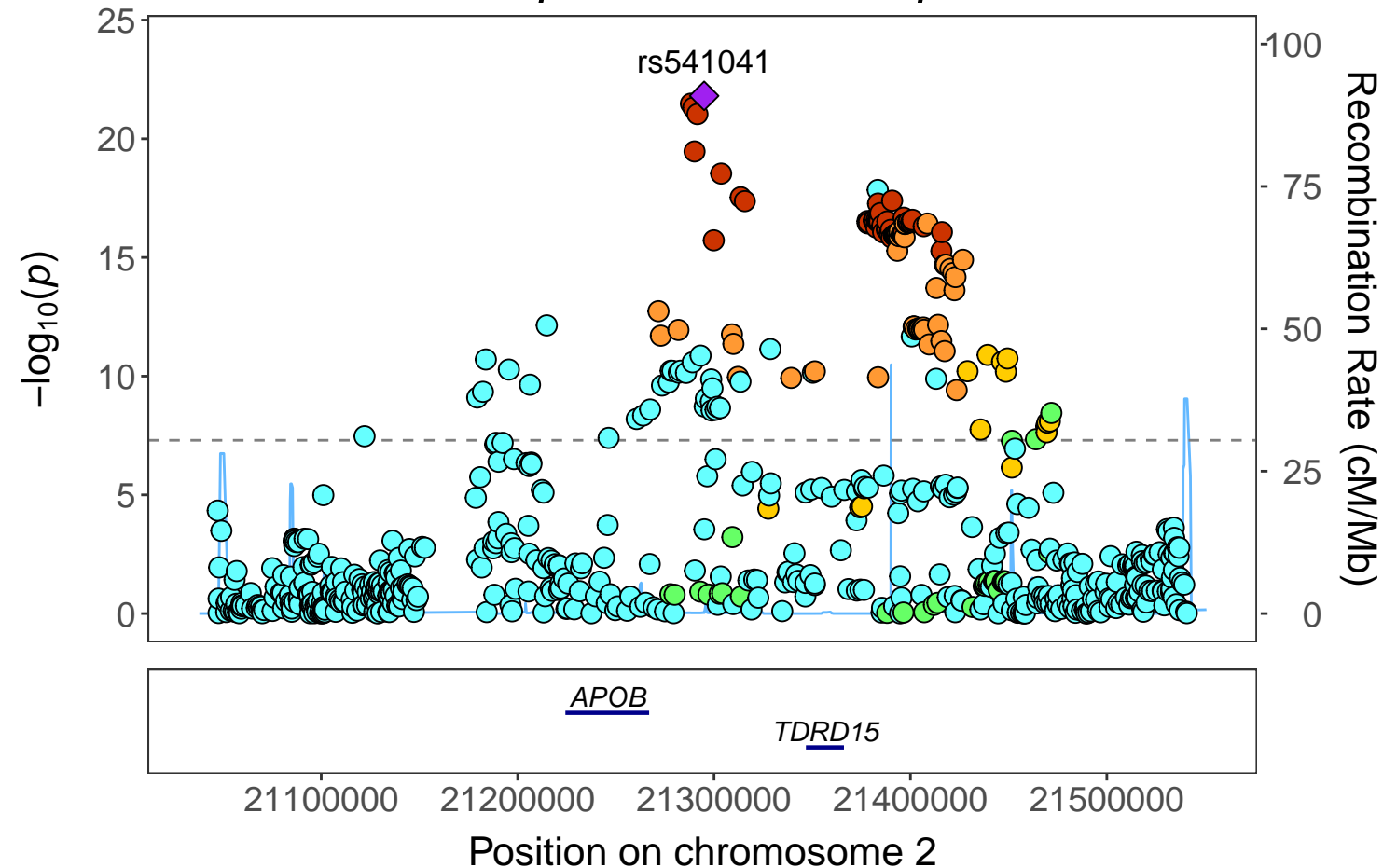

Supplement: Supplementary file 5 — Supporting Information [file CTM2-16-e70732-s001.zip › LocusZoom/Sfig_rs541041_locusZoom.pdf]

# LocusZoom plots of GWAS top lead SNP

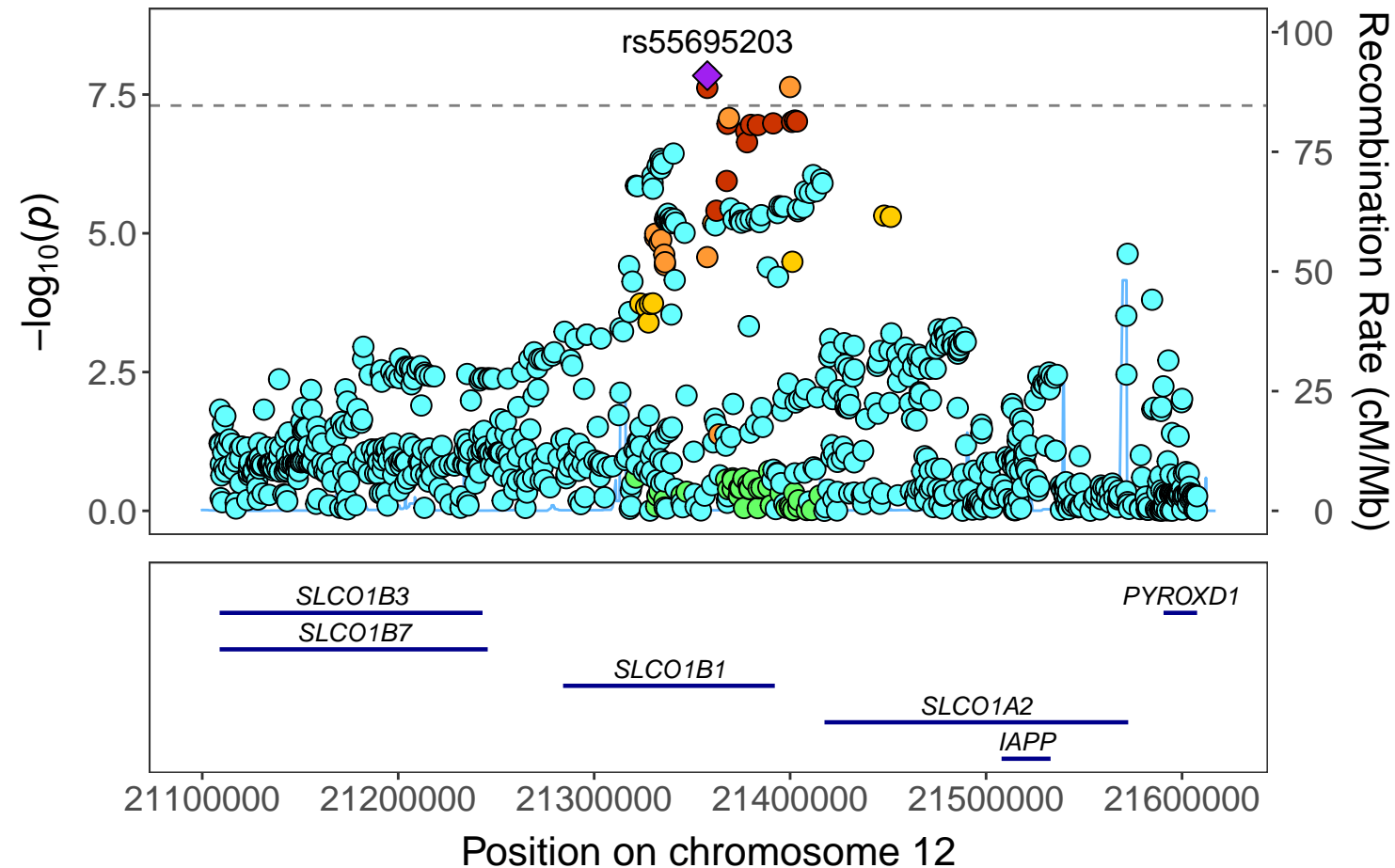

Supplement: Supplementary file 5 — Supporting Information [file CTM2-16-e70732-s001.zip › LocusZoom/Sfig_rs55695203_locusZoom.pdf]

# *LocusZoom plots of GWAS top lead SNP*

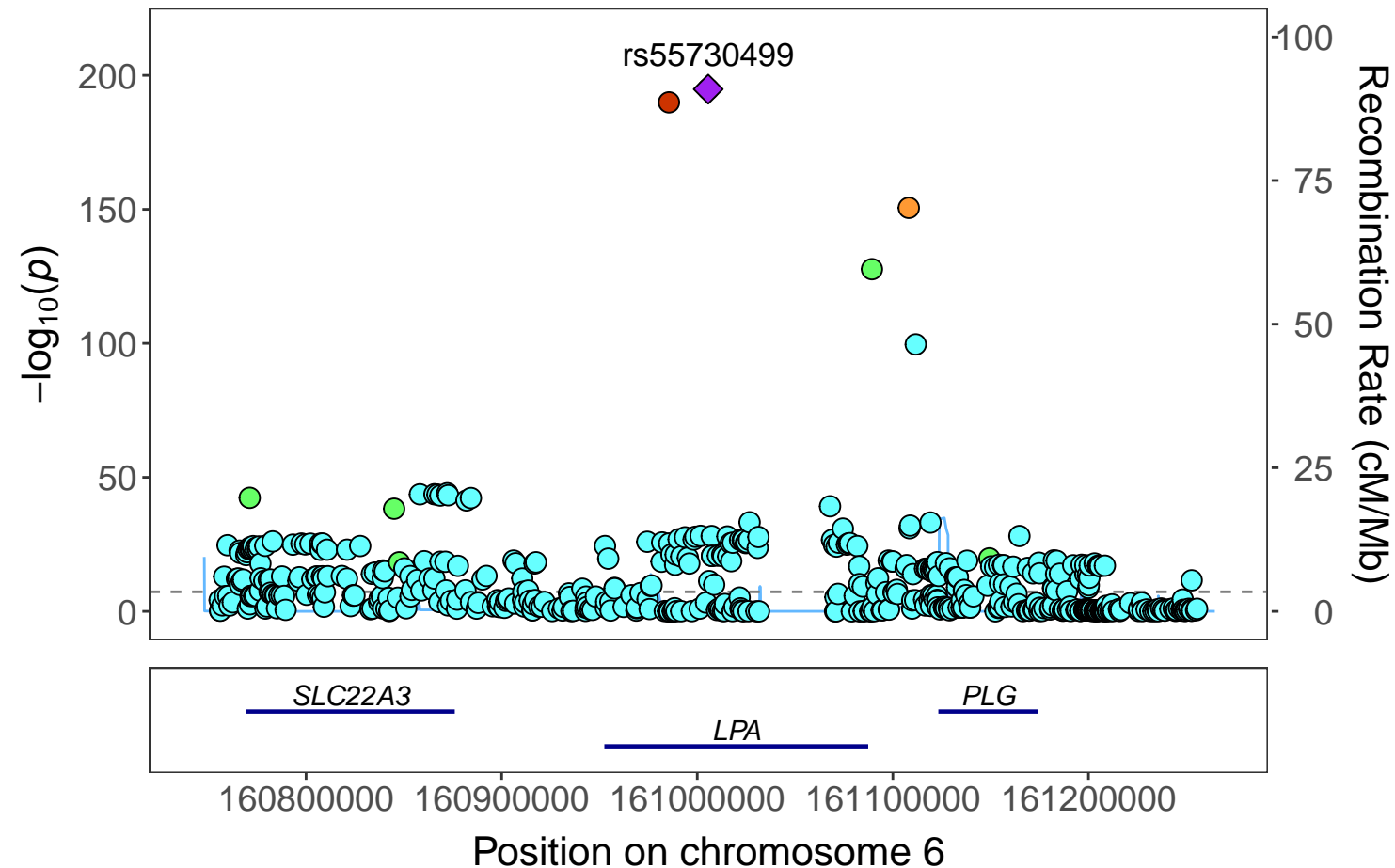

r2    miss    0.0–0.2    0.2–0.4    0.4–0.6    0.6–0.8    0.8–1.0

Supplement: Supplementary file 5 — Supporting Information [file CTM2-16-e70732-s001.zip › LocusZoom/Sfig_rs55730499_locusZoom.pdf]

# LocusZoom plots of GWAS top lead SNP

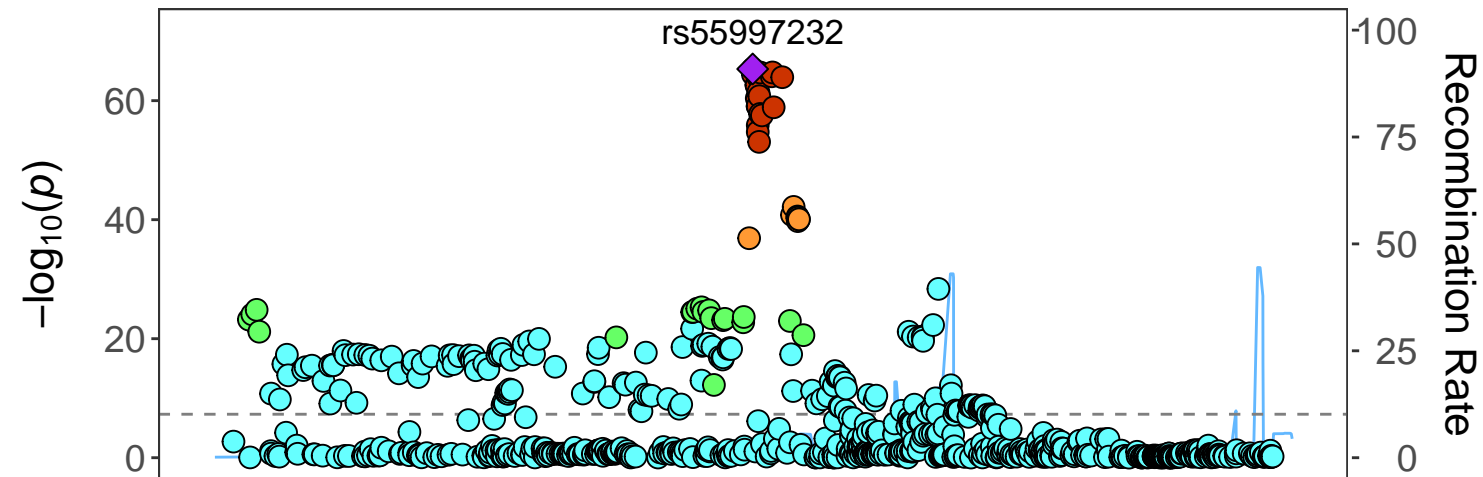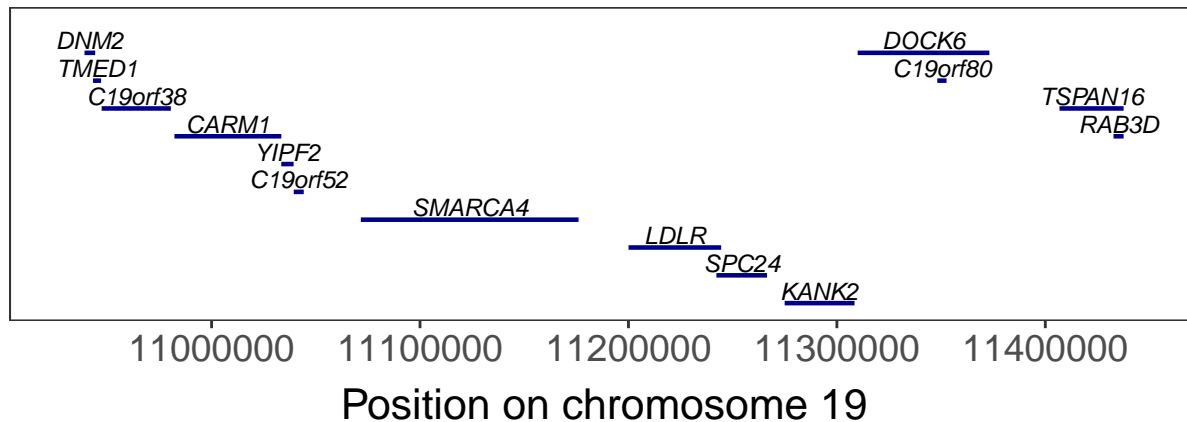

r2    miss    0.0–0.2    0.2–0.4    0.4–0.6    0.6–0.8    0.8–1.0

Supplement: Supplementary file 5 — Supporting Information [file CTM2-16-e70732-s001.zip › LocusZoom/Sfig_rs55997232_locusZoom.pdf]

## LocusZoom plots of GWAS top lead SNP

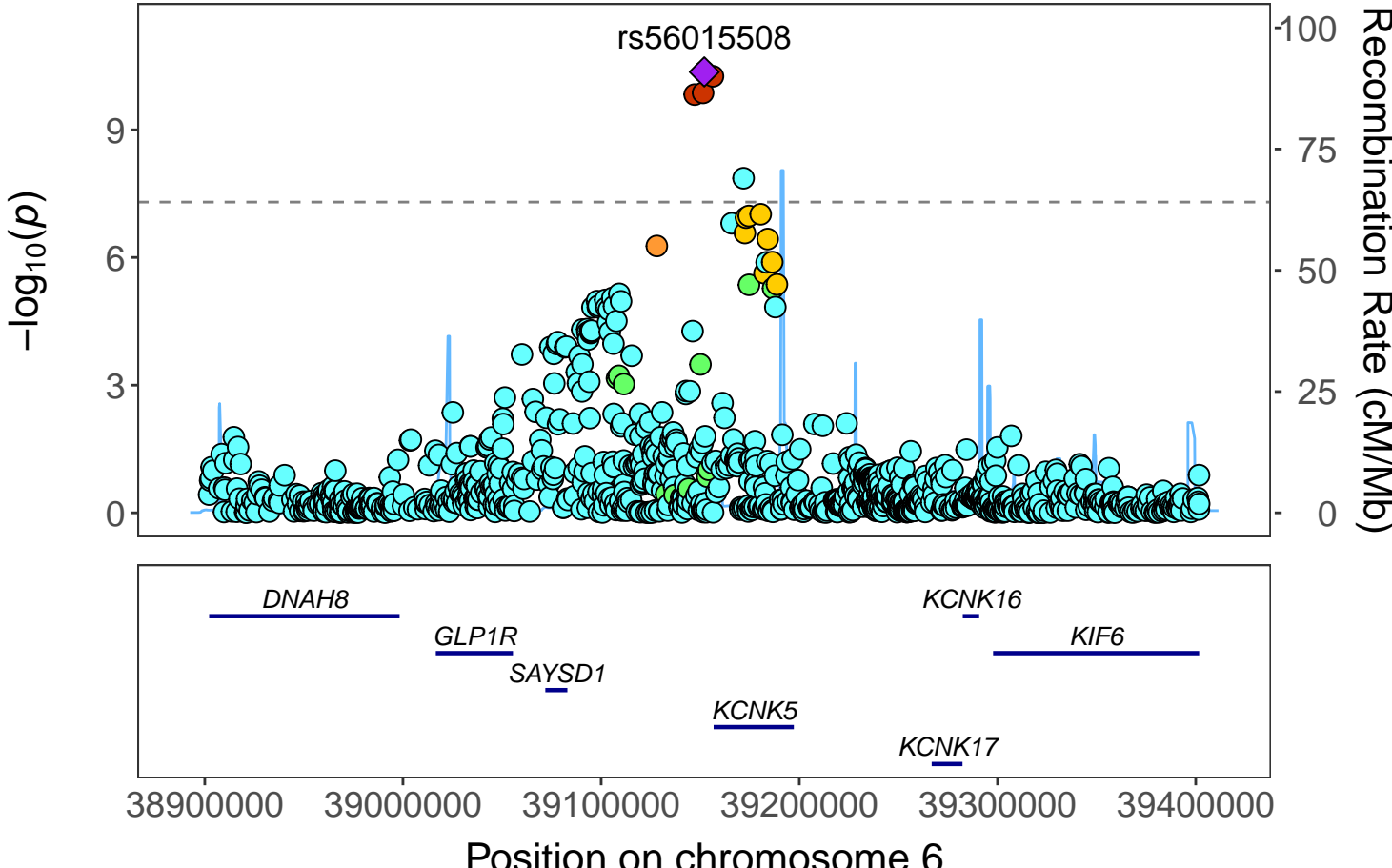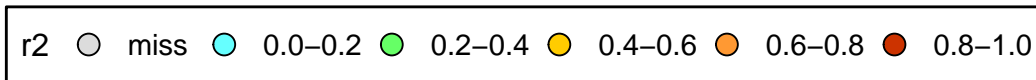

Supplement: Supplementary file 5 — Supporting Information [file CTM2-16-e70732-s001.zip › LocusZoom/Sfig_rs56015508_locusZoom.pdf]

# LocusZoom plots of GWAS top lead SNP

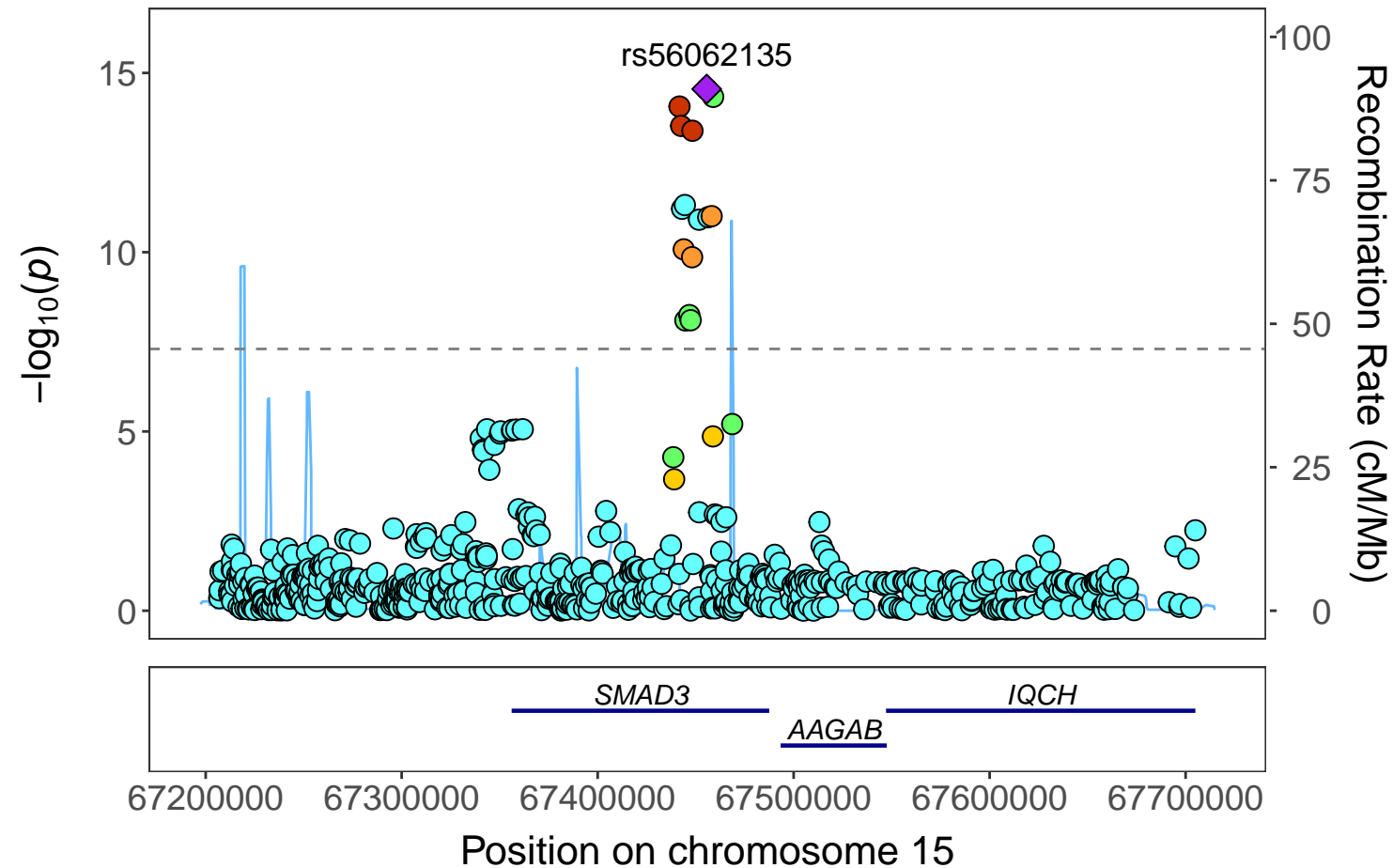

$r^2$    miss   cyan   0.0-0.2   green   0.2-0.4   yellow   0.4-0.6   orange   0.6-0.8   red   0.8-1.0

Supplement: Supplementary file 5 — Supporting Information [file CTM2-16-e70732-s001.zip › LocusZoom/Sfig_rs56062135_locusZoom.pdf]

# LocusZoom plots of GWAS top lead SNP

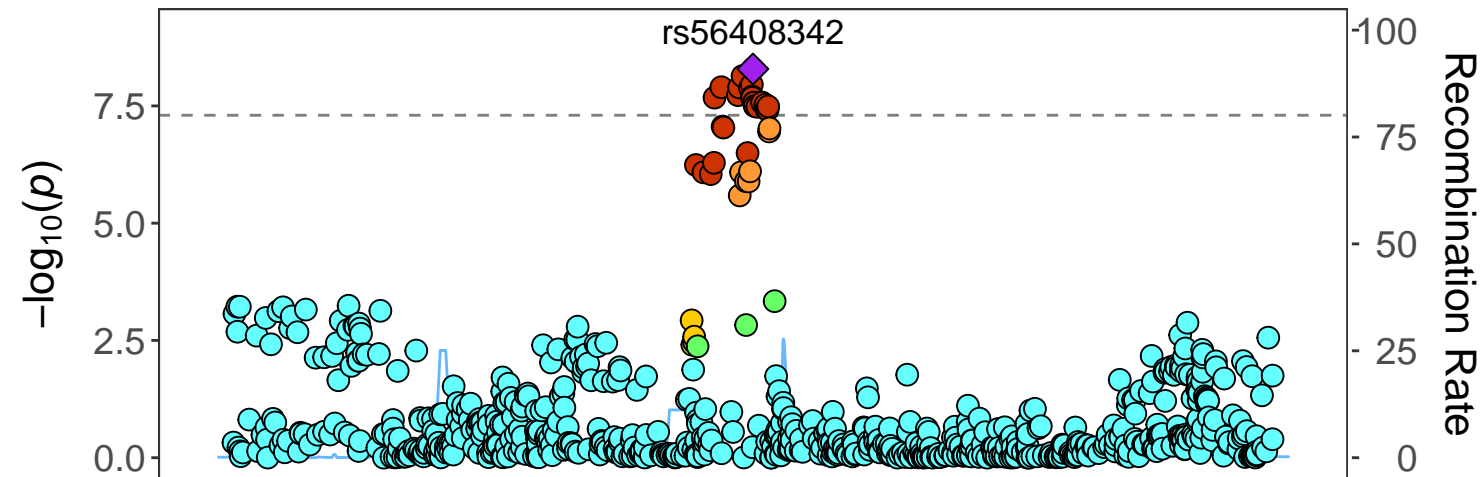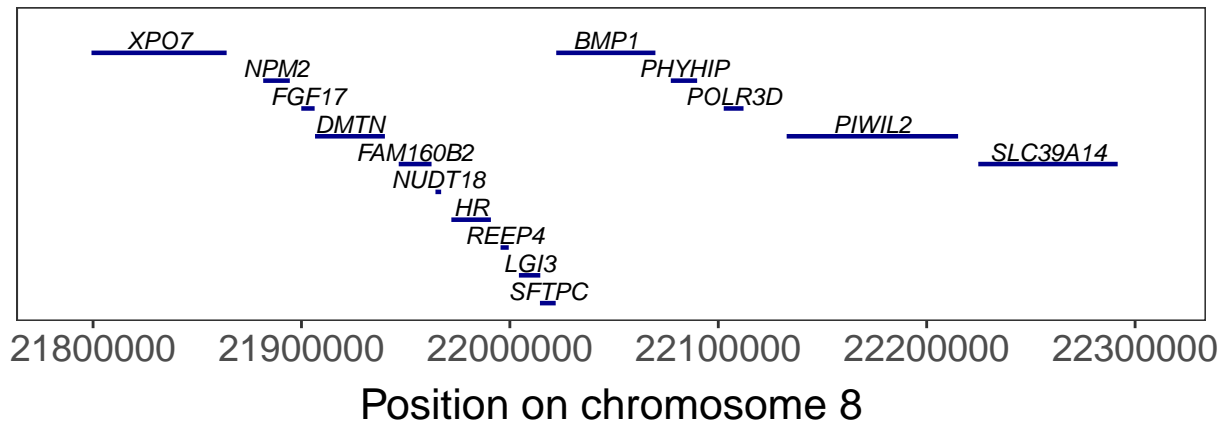

r2   ○   miss   ●   0.0–0.2   ●   0.2–0.4   ●   0.4–0.6   ●   0.6–0.8   ●   0.8–1.0

Supplement: Supplementary file 5 — Supporting Information [file CTM2-16-e70732-s001.zip › LocusZoom/Sfig_rs56408342_locusZoom.pdf]

# LocusZoom plots of GWAS top lead SNP

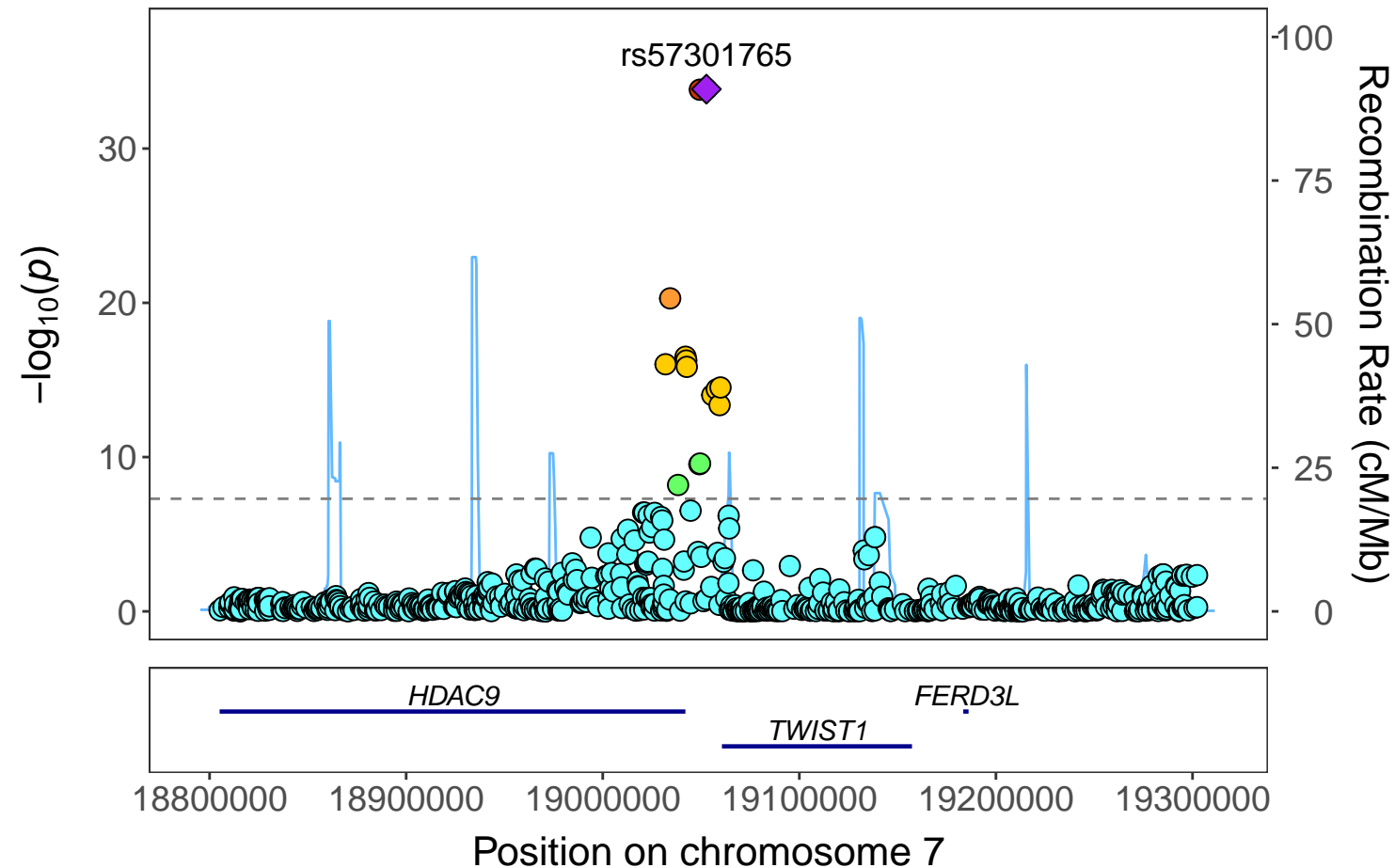

r2   miss   0.0–0.2   0.2–0.4   0.4–0.6   0.6–0.8   0.8–1.0

Supplement: Supplementary file 5 — Supporting Information [file CTM2-16-e70732-s001.zip › LocusZoom/Sfig_rs57301765_locusZoom.pdf]

# LocusZoom plots of GWAS top lead SNP

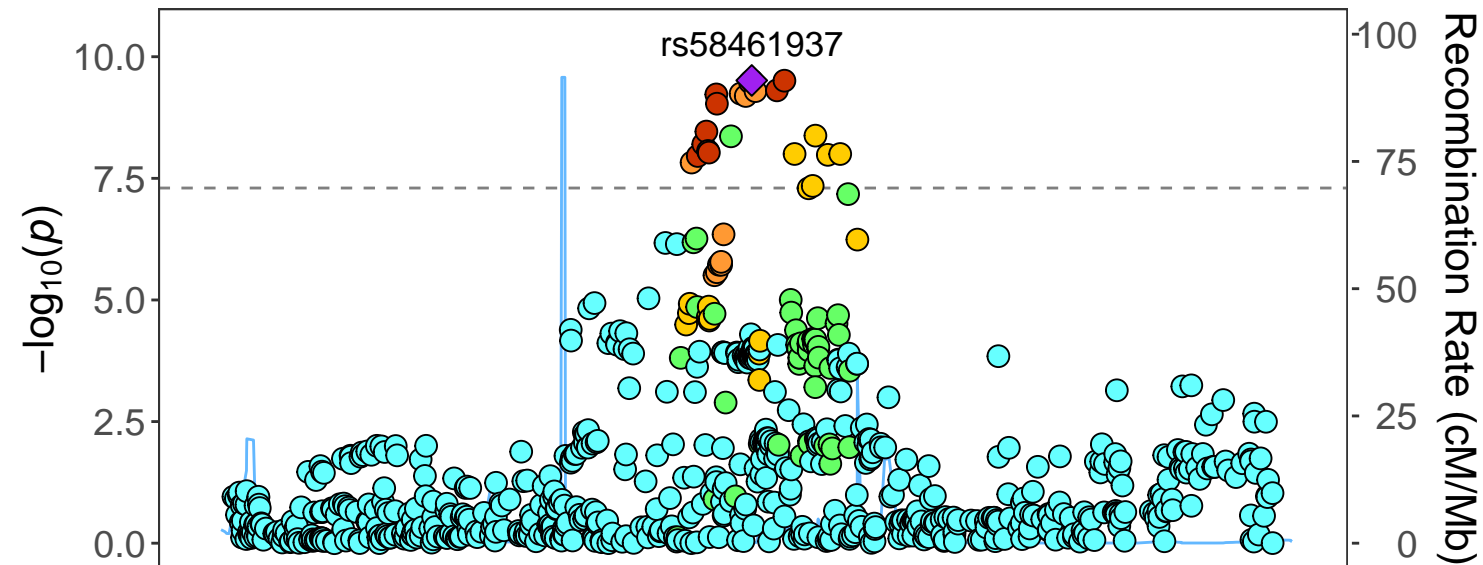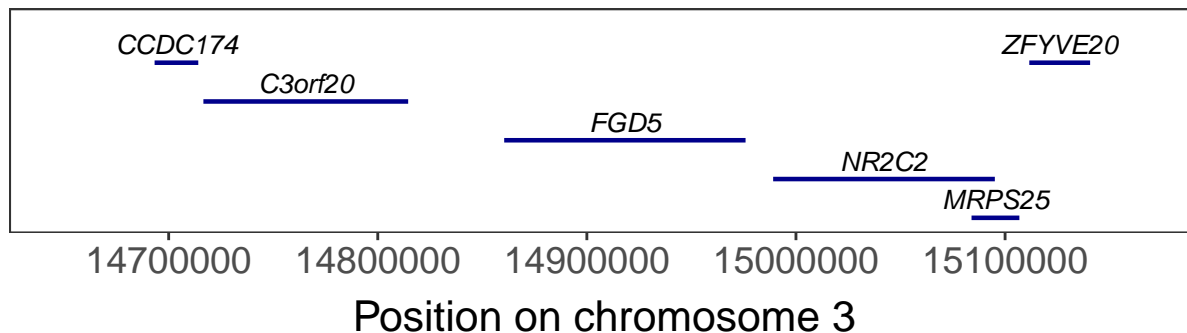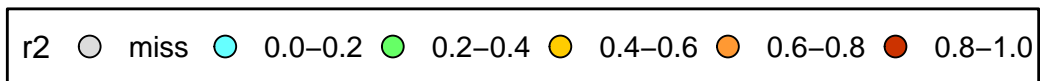

Supplement: Supplementary file 5 — Supporting Information [file CTM2-16-e70732-s001.zip › LocusZoom/Sfig_rs58461937_locusZoom.pdf]

# LocusZoom plots of GWAS top lead SNP

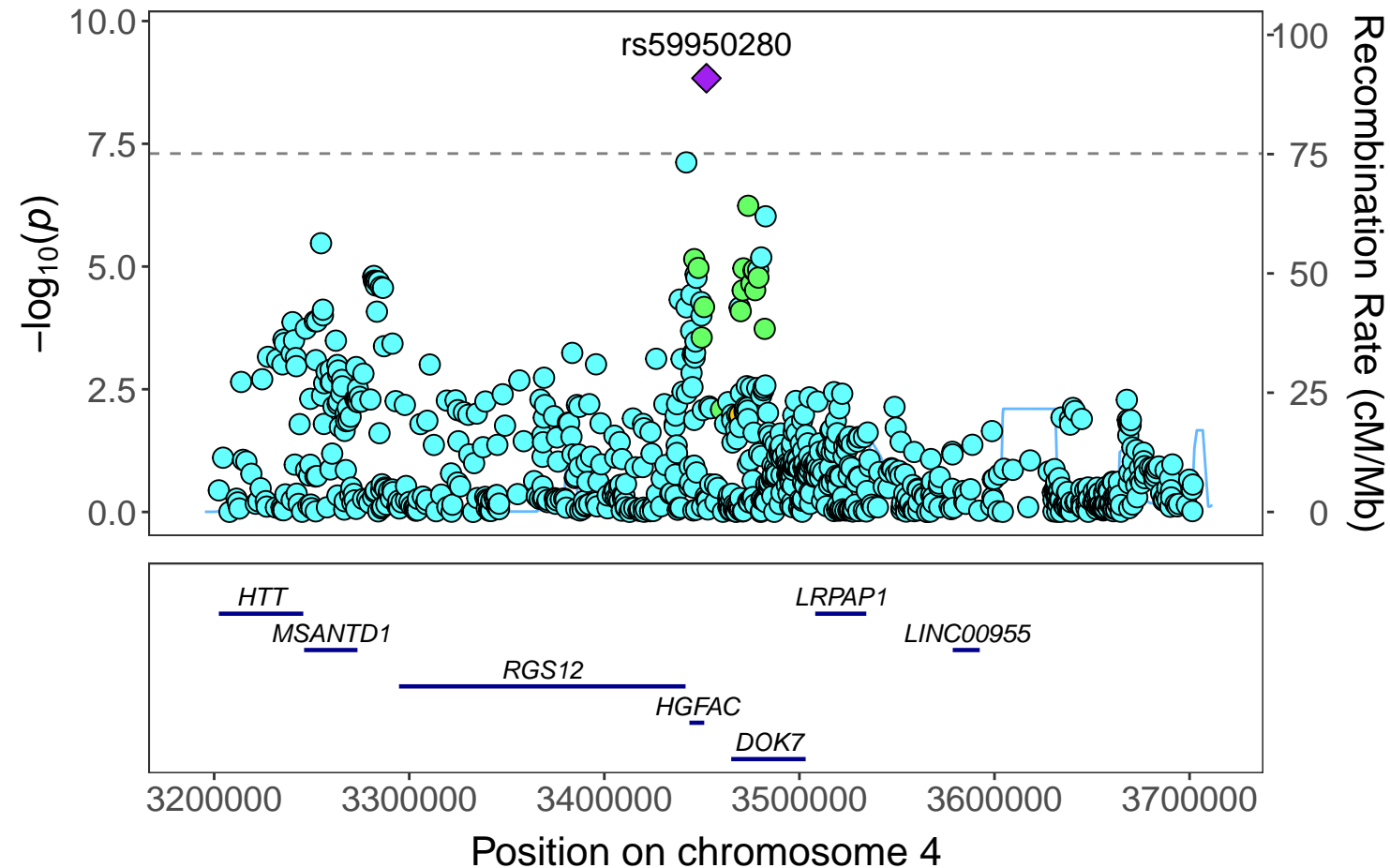

r2   ○   miss   ○   0.0-0.2   ○   0.2-0.4   ○   0.4-0.6   ○   0.6-0.8   ○   0.8-1.0

Supplement: Supplementary file 5 — Supporting Information [file CTM2-16-e70732-s001.zip › LocusZoom/Sfig_rs59950280_locusZoom.pdf]

# LocusZoom plots of GWAS top lead SNP

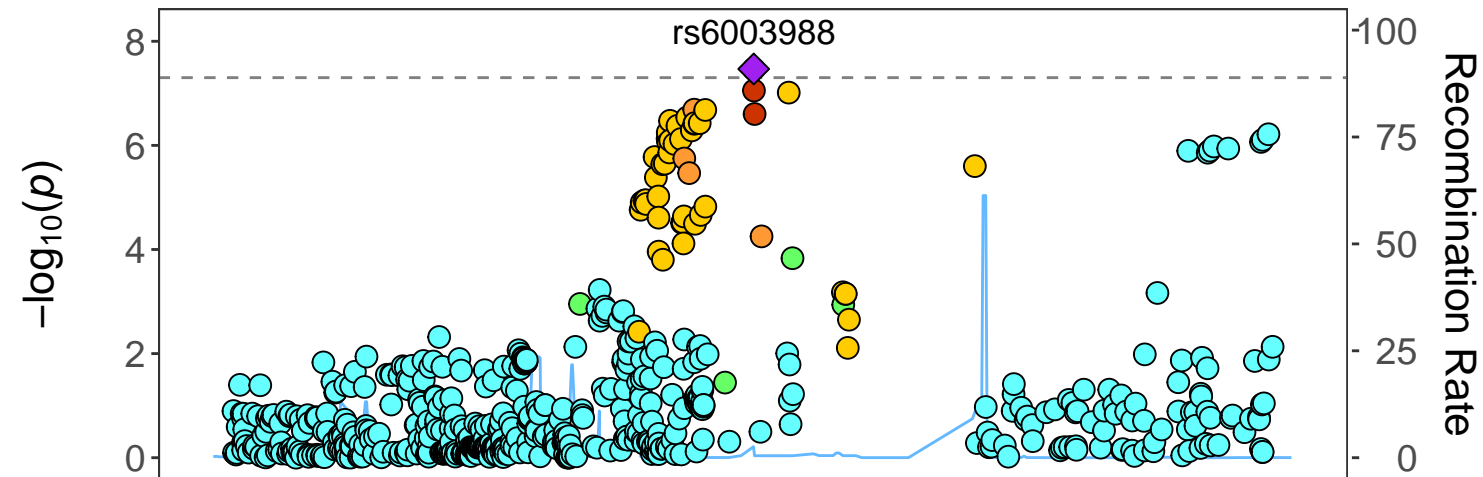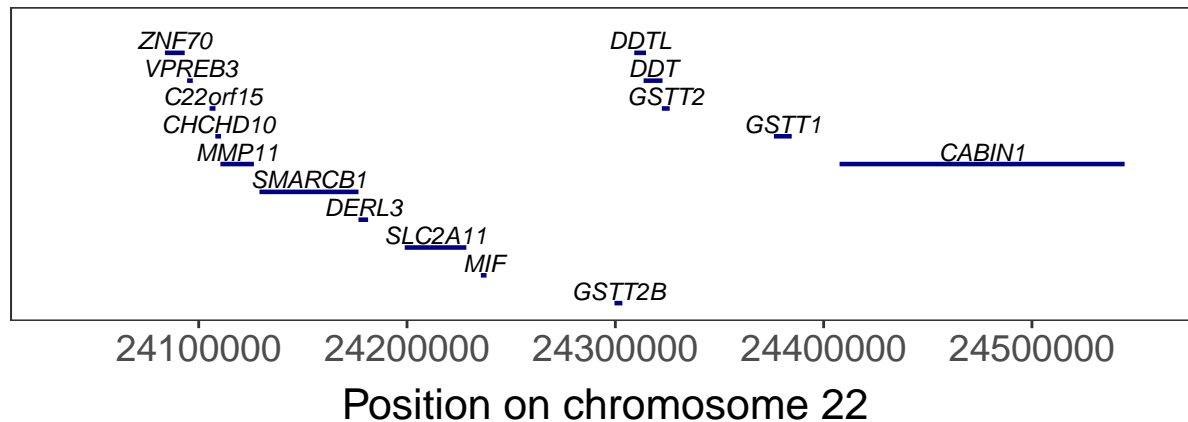

$r^2$     $\circ$  miss    $\circ$  0.0-0.2    $\circ$  0.2-0.4    $\circ$  0.4-0.6    $\circ$  0.6-0.8    $\circ$  0.8-1.0

Supplement: Supplementary file 5 — Supporting Information [file CTM2-16-e70732-s001.zip › LocusZoom/Sfig_rs6003988_locusZoom.pdf]

# LocusZoom plots of GWAS top lead SNP

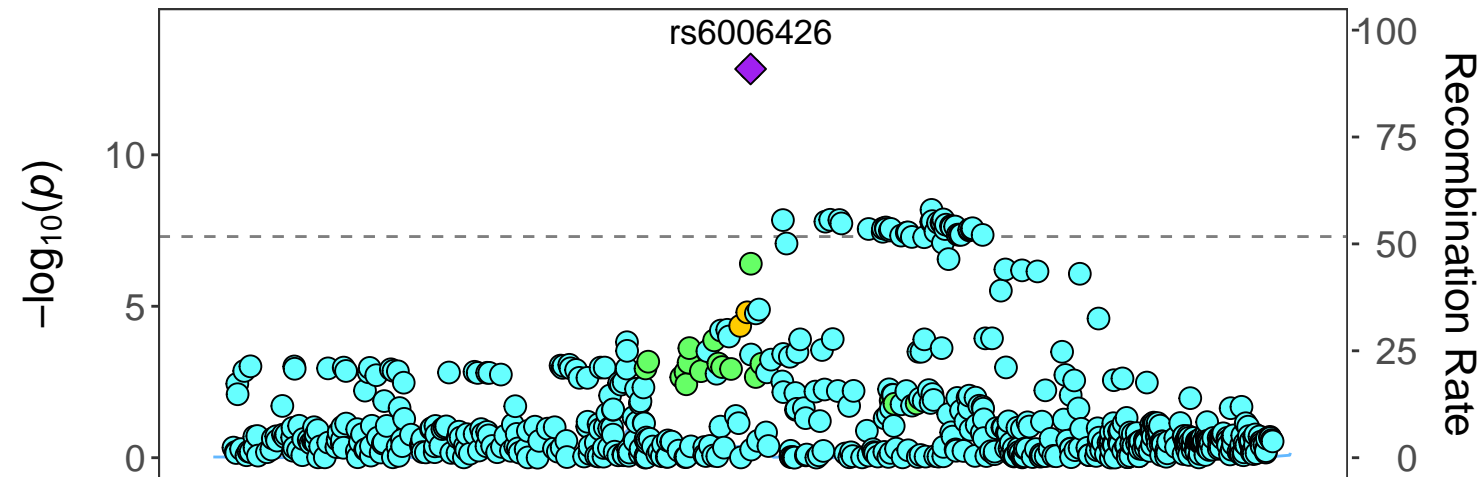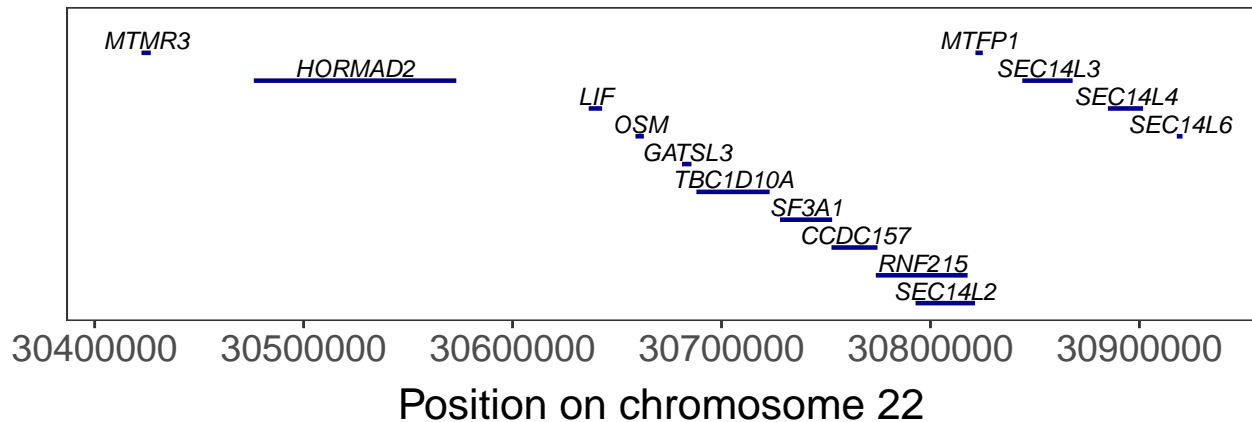

$r^2$     $\circ$  miss    $\circ$  0.0–0.2    $\circ$  0.2–0.4    $\circ$  0.4–0.6    $\circ$  0.6–0.8    $\circ$  0.8–1.0

Supplement: Supplementary file 5 — Supporting Information [file CTM2-16-e70732-s001.zip › LocusZoom/Sfig_rs6006426_locusZoom.pdf]

# LocusZoom plots of GWAS top lead SNP

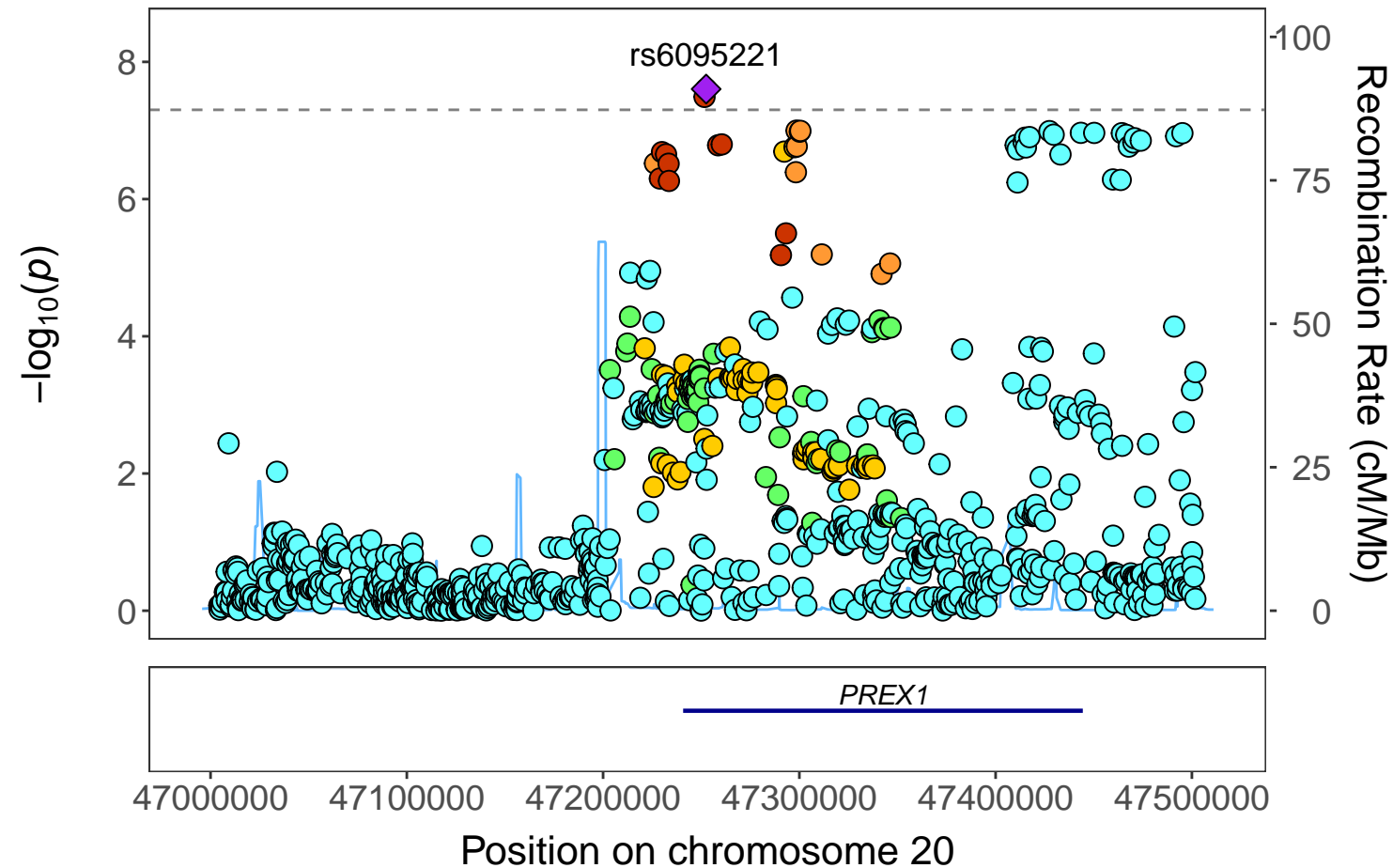

$r^2$    miss   cyan   0.0–0.2   green   0.2–0.4   yellow   0.4–0.6   orange   0.6–0.8   dark red   0.8–1.0

Supplement: Supplementary file 5 — Supporting Information [file CTM2-16-e70732-s001.zip › LocusZoom/Sfig_rs6095221_locusZoom.pdf]

# LocusZoom plots of GWAS top lead SNP

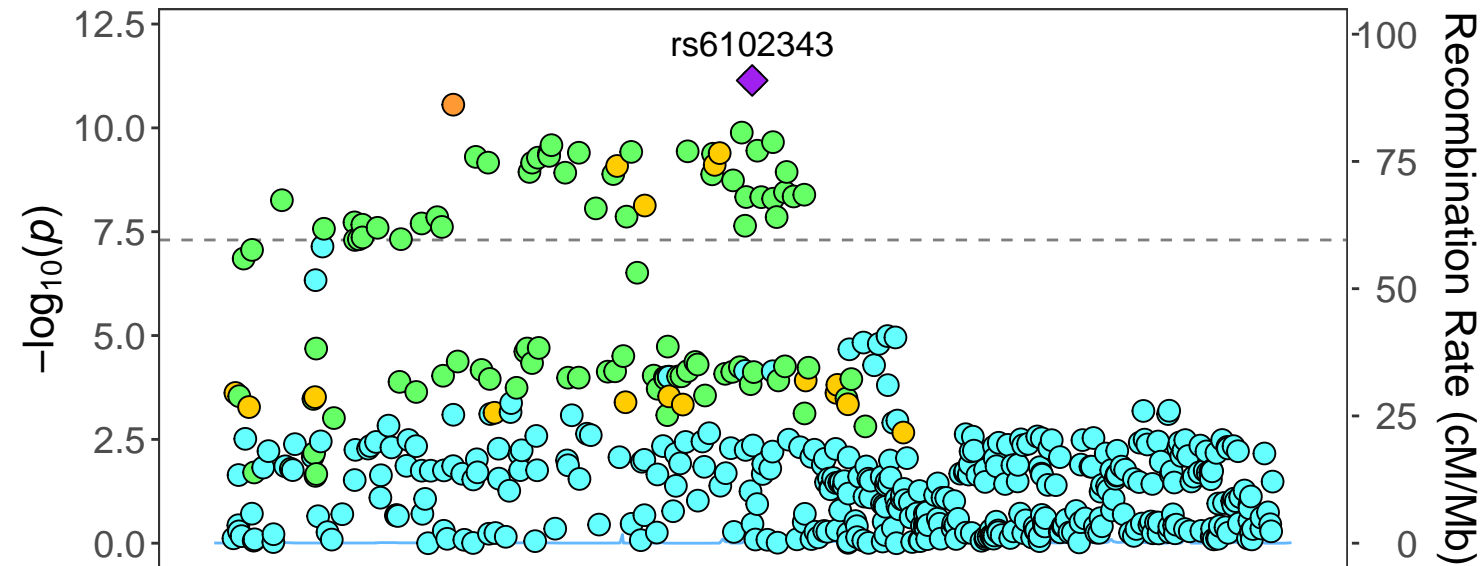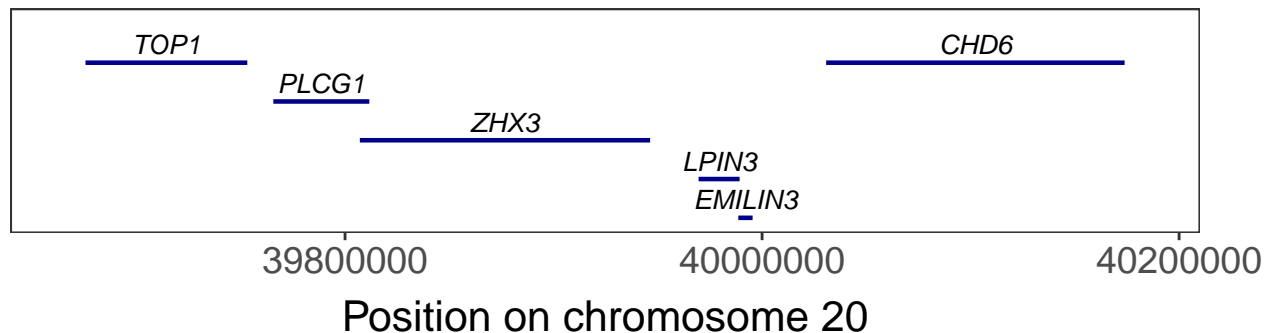

r2   miss   0.0-0.2   0.2-0.4   0.4-0.6   0.6-0.8   0.8-1.0

Supplement: Supplementary file 5 — Supporting Information [file CTM2-16-e70732-s001.zip › LocusZoom/Sfig_rs6102343_locusZoom.pdf]

# LocusZoom plots of GWAS top lead SNP

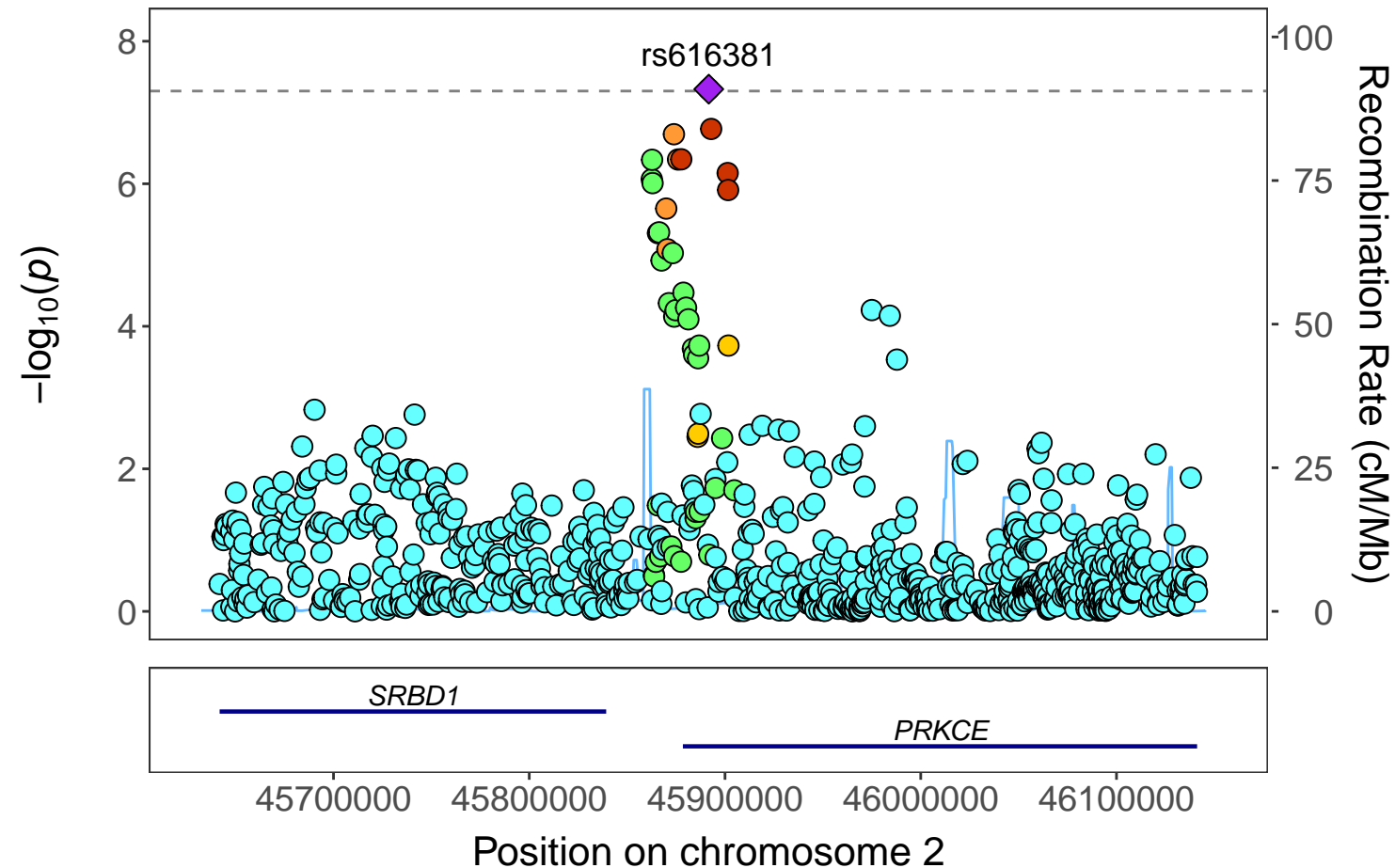

$r^2$    miss   cyan   0.0–0.2   green   0.2–0.4   yellow   0.4–0.6   orange   0.6–0.8   red   0.8–1.0

Supplement: Supplementary file 5 — Supporting Information [file CTM2-16-e70732-s001.zip › LocusZoom/Sfig_rs616381_locusZoom.pdf]

# LocusZoom plots of GWAS top lead SNP

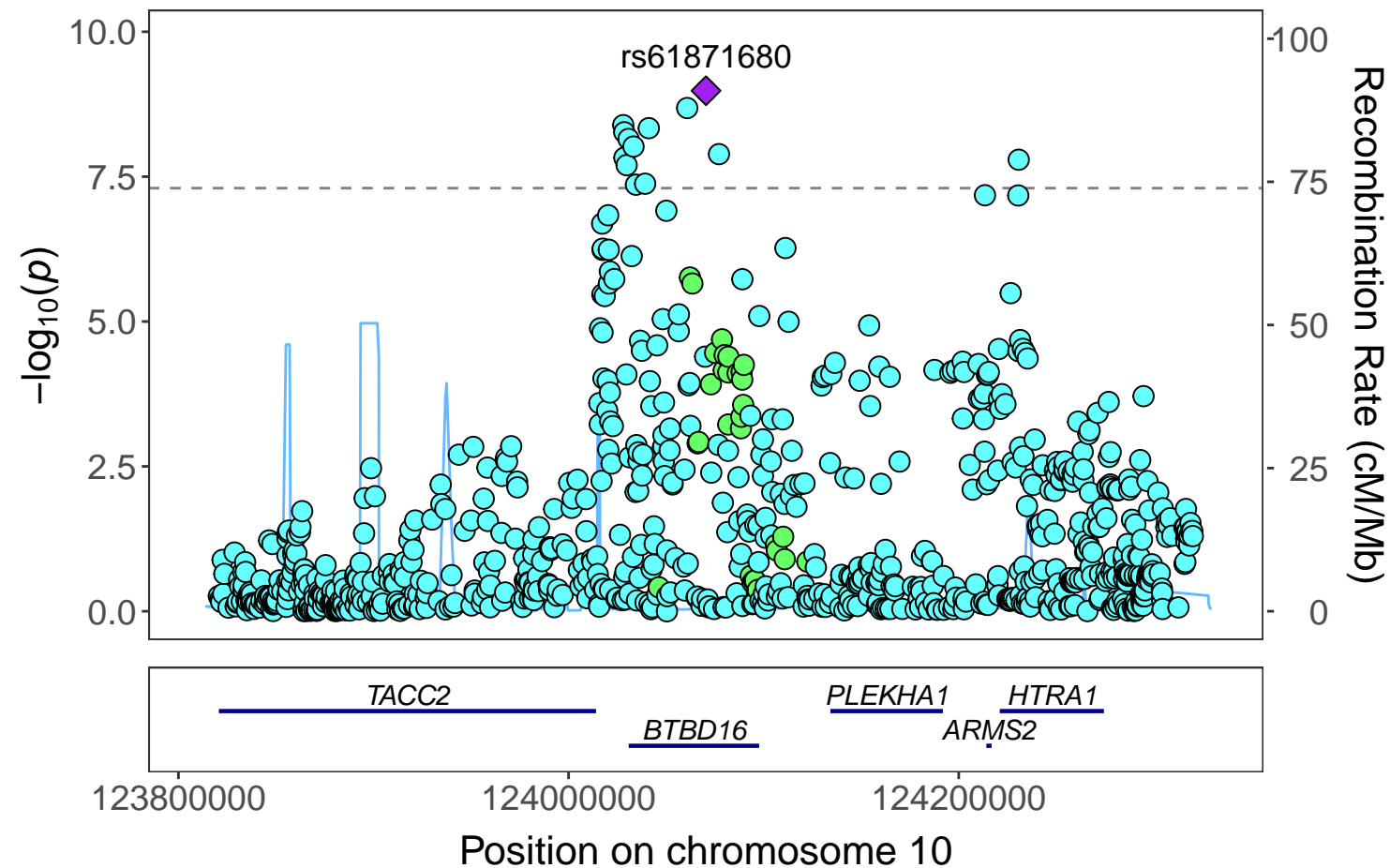

$r^2$  ○ miss ● 0.0–0.2 ● 0.2–0.4 ● 0.4–0.6 ● 0.6–0.8 ● 0.8–1.0

Supplement: Supplementary file 5 — Supporting Information [file CTM2-16-e70732-s001.zip › LocusZoom/Sfig_rs61871680_locusZoom.pdf]

# LocusZoom plots of GWAS top lead SNP

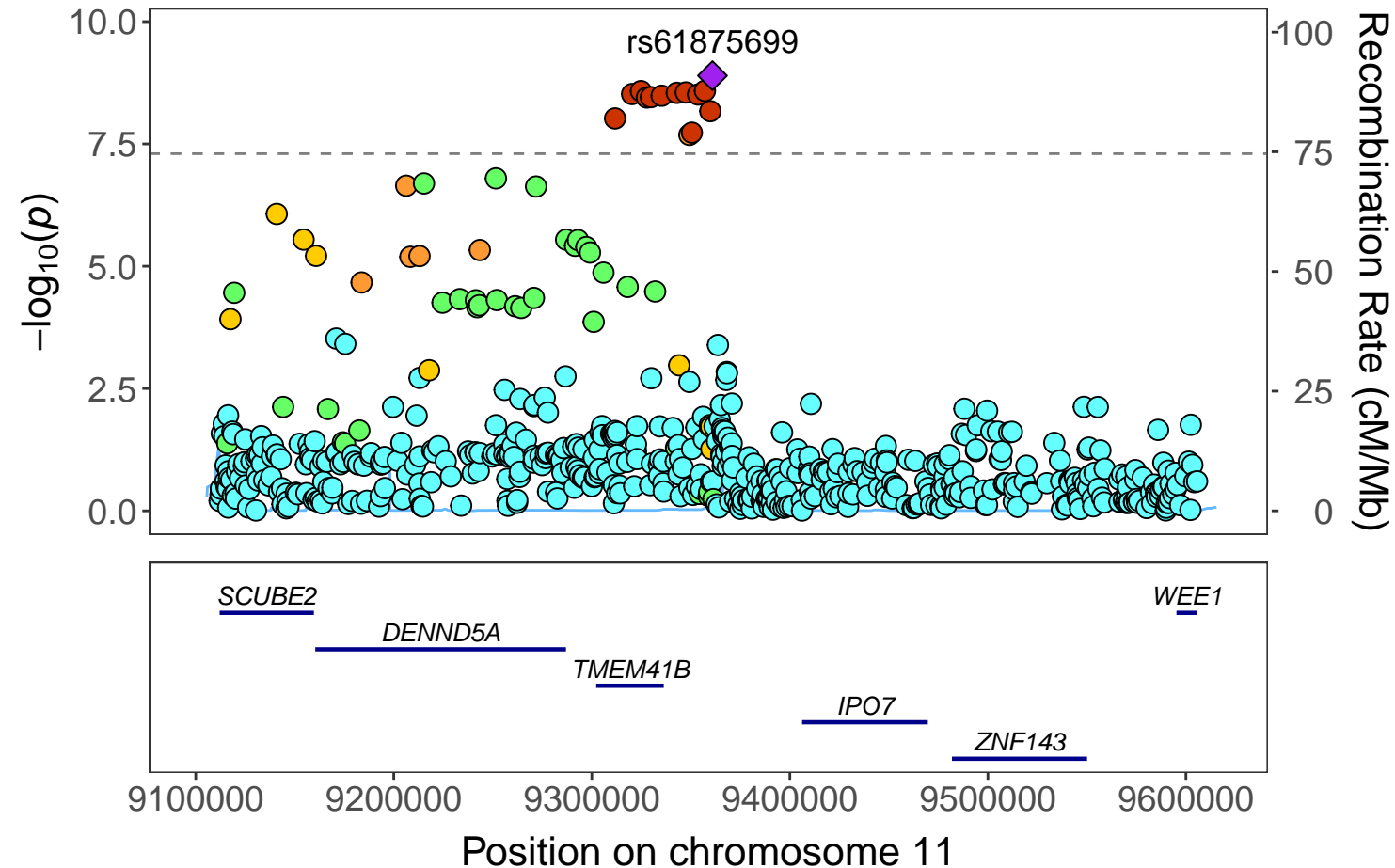

r2    miss    0.0–0.2    0.2–0.4    0.4–0.6    0.6–0.8    0.8–1.0

Supplement: Supplementary file 5 — Supporting Information [file CTM2-16-e70732-s001.zip › LocusZoom/Sfig_rs61875699_locusZoom.pdf]

# LocusZoom plots of GWAS top lead SNP

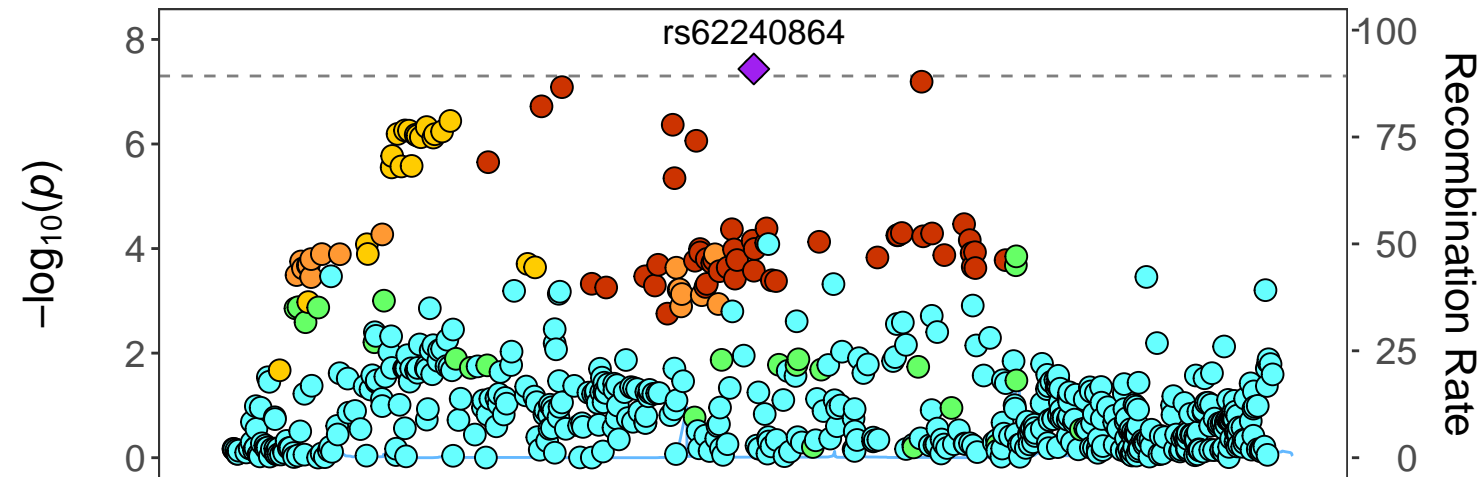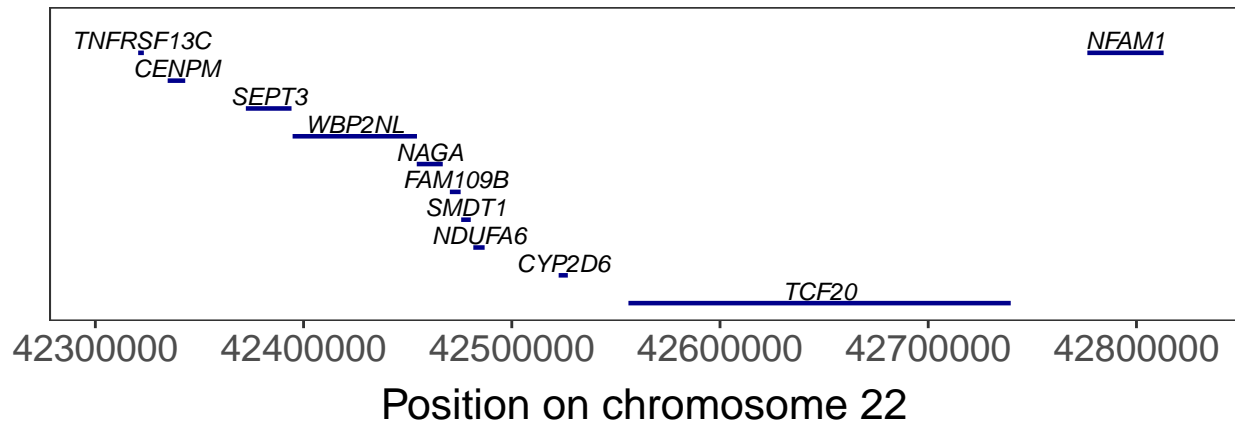

$r^2$    miss   0.0–0.2   0.2–0.4   0.4–0.6   0.6–0.8   0.8–1.0

Supplement: Supplementary file 5 — Supporting Information [file CTM2-16-e70732-s001.zip › LocusZoom/Sfig_rs62240864_locusZoom.pdf]

# *LocusZoom plots of GWAS top lead SNP*

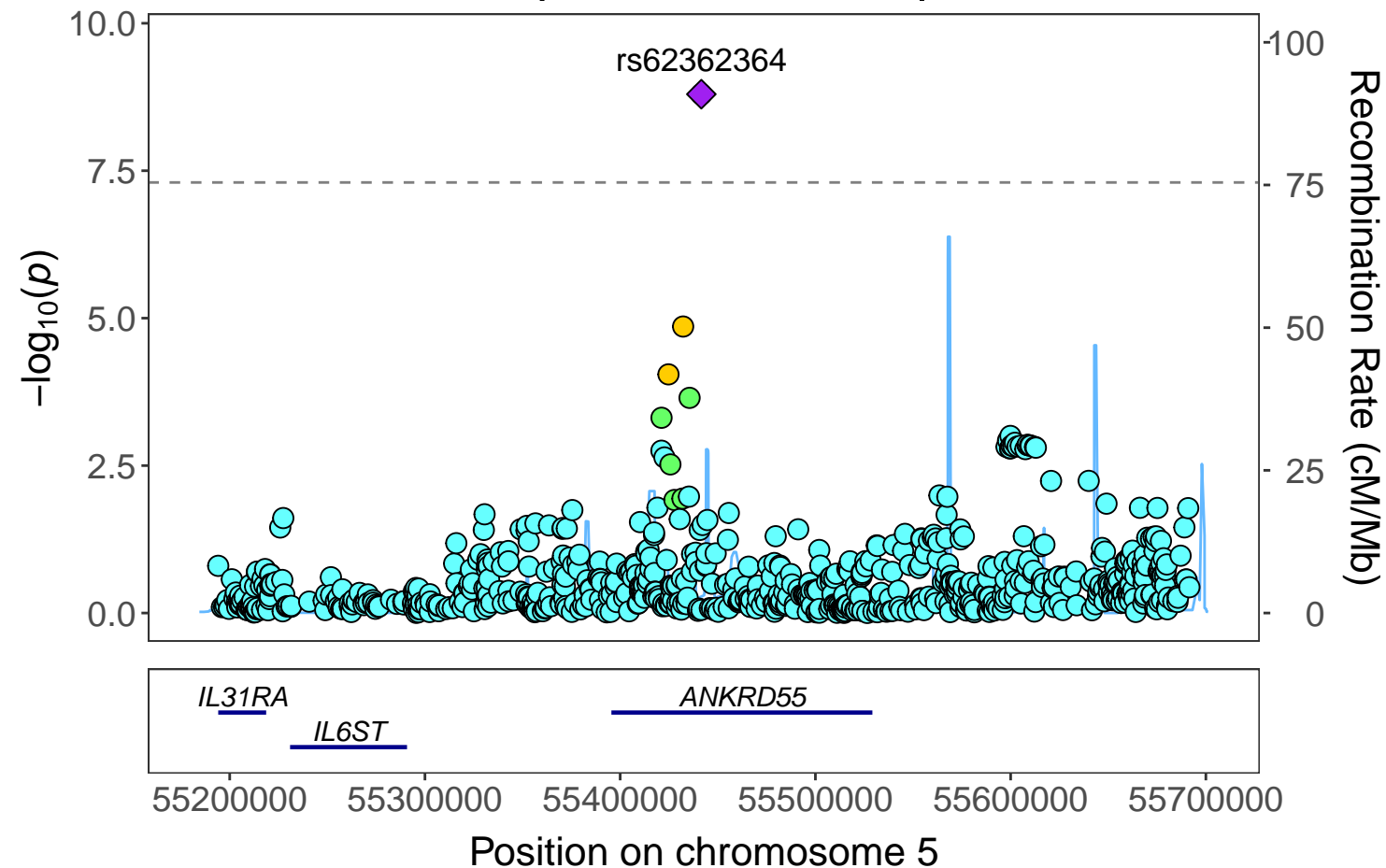

Supplement: Supplementary file 5 — Supporting Information [file CTM2-16-e70732-s001.zip › LocusZoom/Sfig_rs62362364_locusZoom.pdf]

# *LocusZoom plots of GWAS top lead SNP*

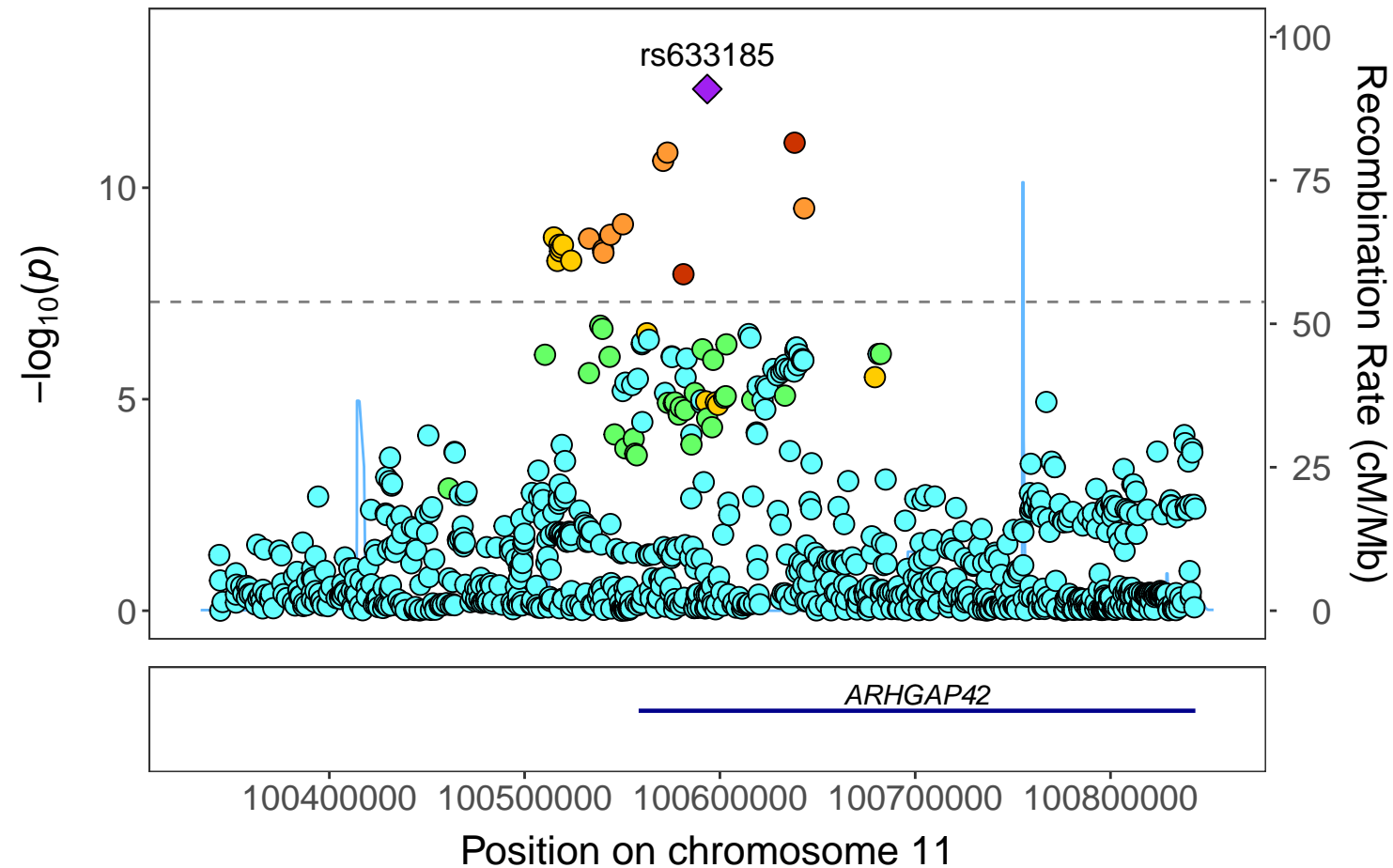

$r^2$    miss   cyan   0.0–0.2   green   0.2–0.4   yellow   0.4–0.6   orange   0.6–0.8   red   0.8–1.0

Supplement: Supplementary file 5 — Supporting Information [file CTM2-16-e70732-s001.zip › LocusZoom/Sfig_rs633185_locusZoom.pdf]

# LocusZoom plots of GWAS top lead SNP

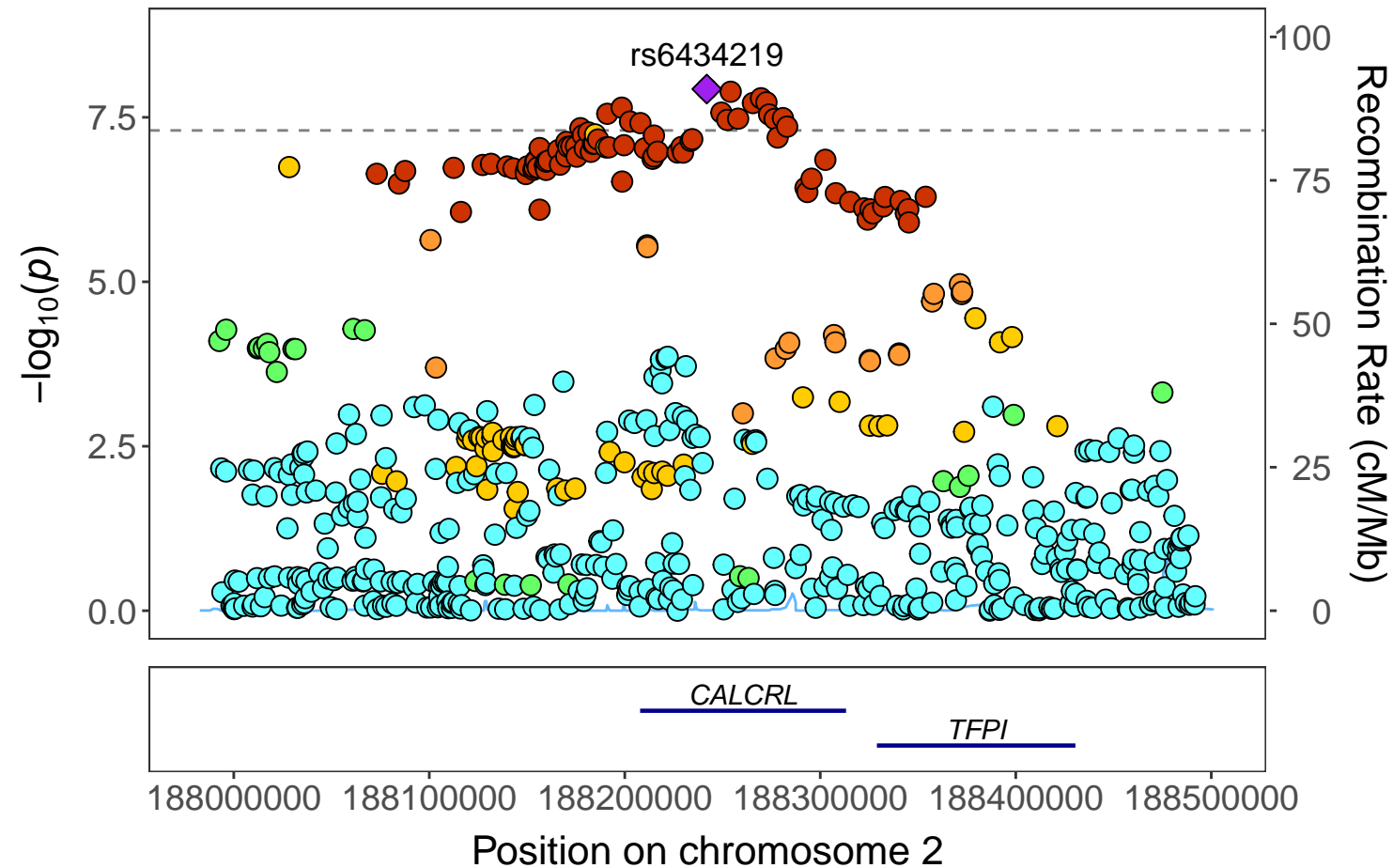

$r^2$    miss   0.0-0.2   0.2-0.4   0.4-0.6   0.6-0.8   0.8-1.0

Supplement: Supplementary file 5 — Supporting Information [file CTM2-16-e70732-s001.zip › LocusZoom/Sfig_rs6434219_locusZoom.pdf]

# LocusZoom plots of GWAS top lead SNP

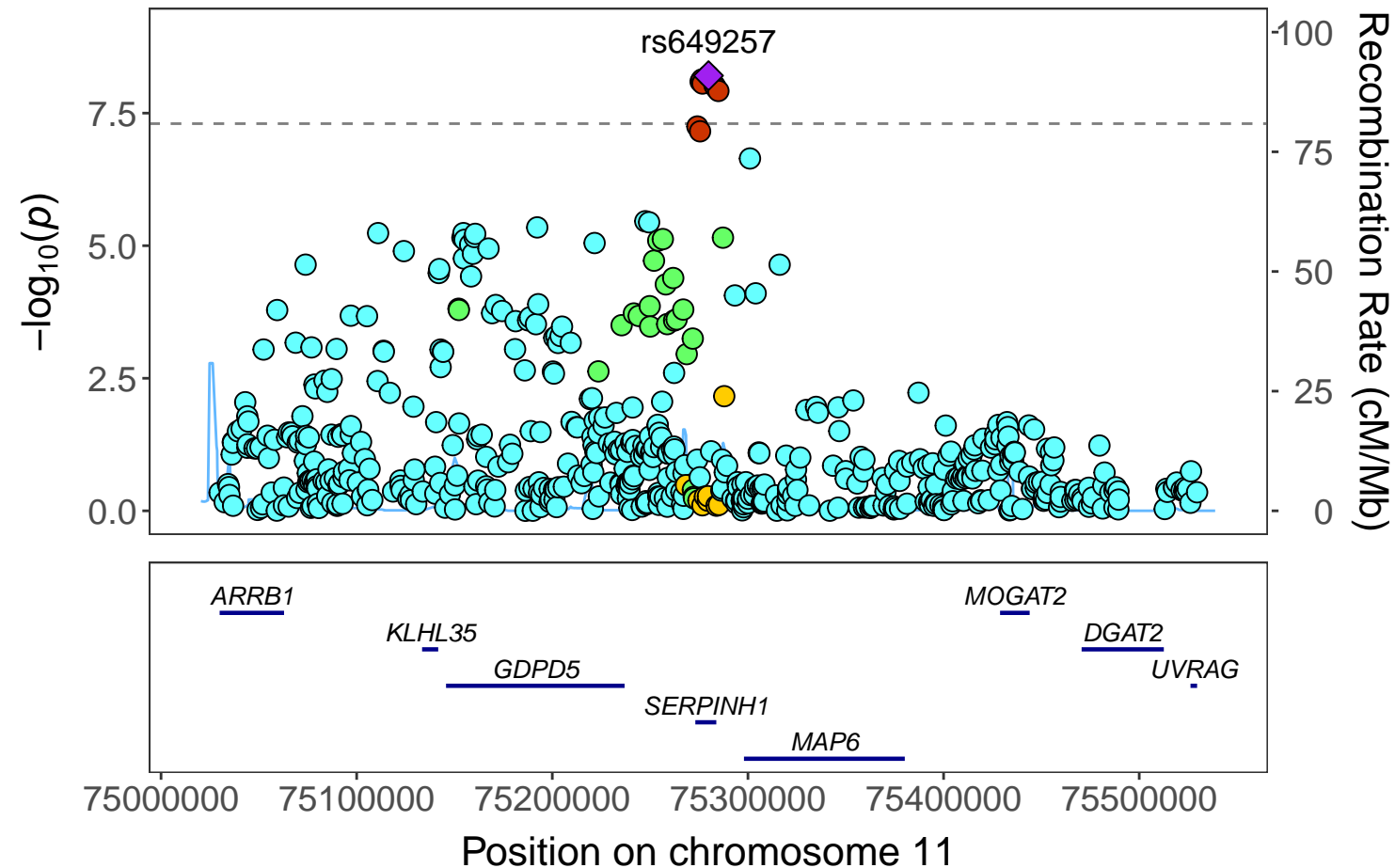

r2   miss   0.0-0.2   0.2-0.4   0.4-0.6   0.6-0.8   0.8-1.0

Supplement: Supplementary file 5 — Supporting Information [file CTM2-16-e70732-s001.zip › LocusZoom/Sfig_rs649257_locusZoom.pdf]

# LocusZoom plots of GWAS top lead SNP

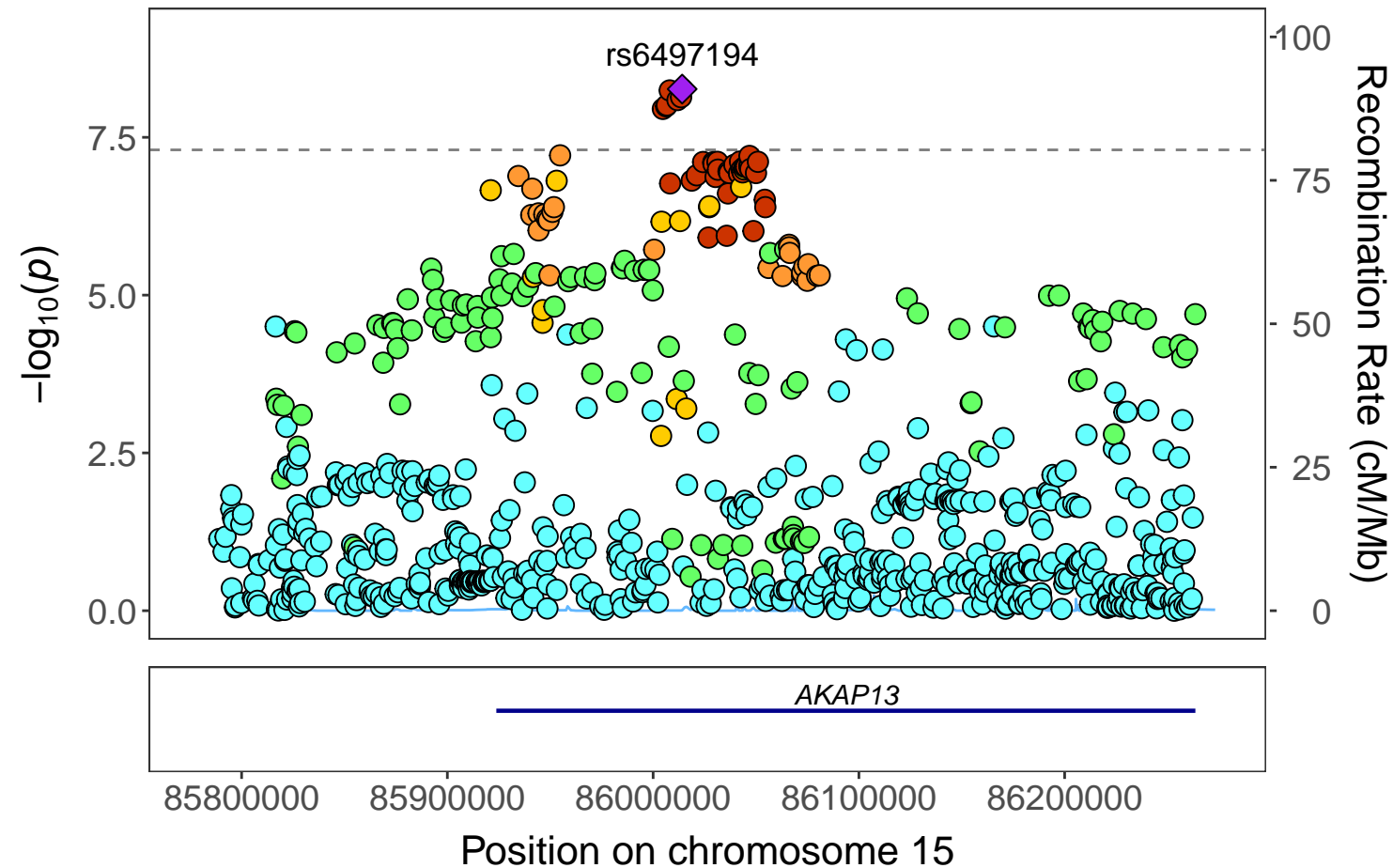

$r^2$    miss   0.0–0.2   0.2–0.4   0.4–0.6   0.6–0.8   0.8–1.0

Supplement: Supplementary file 5 — Supporting Information [file CTM2-16-e70732-s001.zip › LocusZoom/Sfig_rs6497194_locusZoom.pdf]

# LocusZoom plots of GWAS top lead SNP

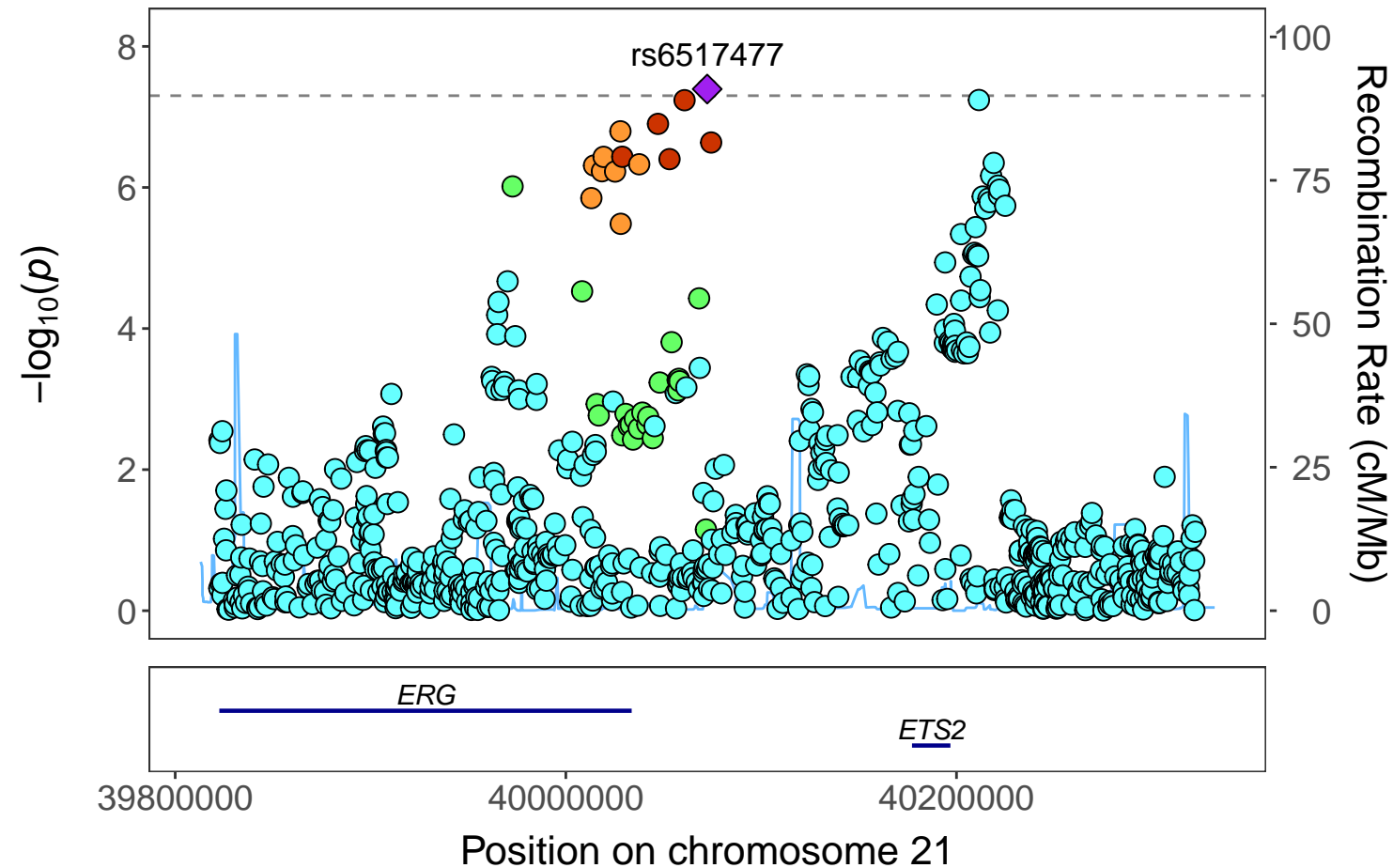

Supplement: Supplementary file 5 — Supporting Information [file CTM2-16-e70732-s001.zip › LocusZoom/Sfig_rs6517477_locusZoom.pdf]

# LocusZoom plots of GWAS top lead SNP

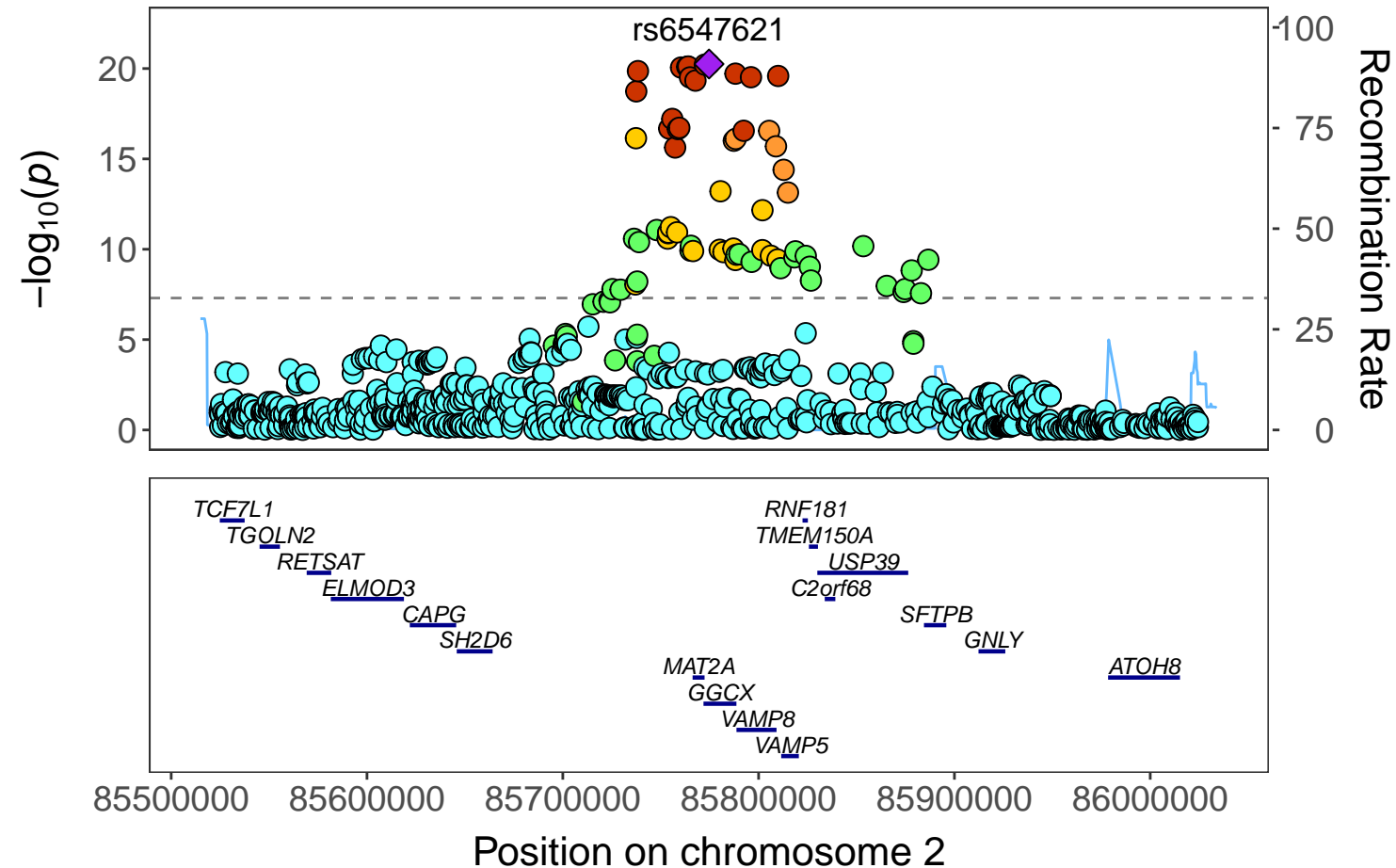

Supplement: Supplementary file 5 — Supporting Information [file CTM2-16-e70732-s001.zip › LocusZoom/Sfig_rs6547621_locusZoom.pdf]

# LocusZoom plots of GWAS top lead SNP

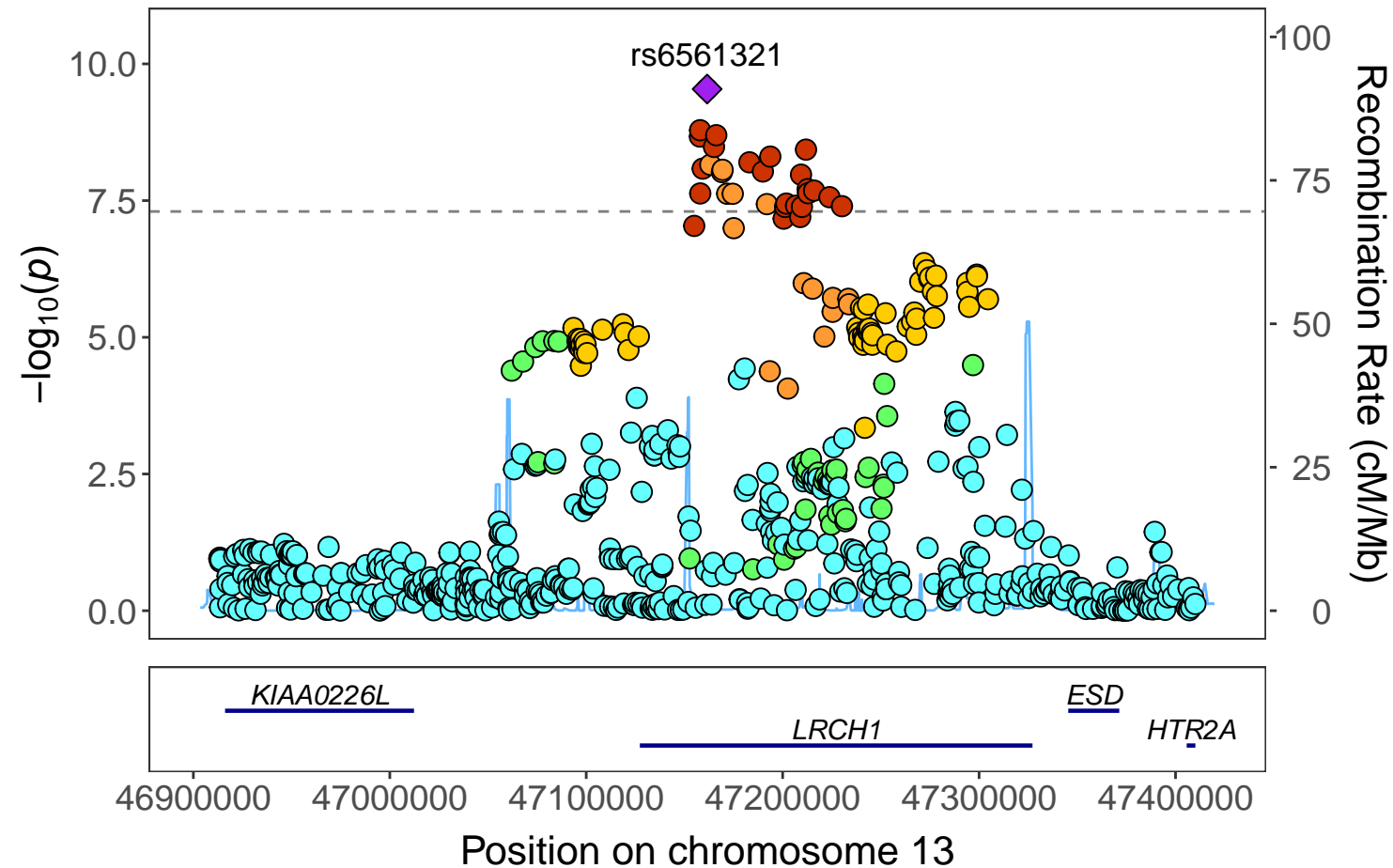

$r^2$    miss   0.0–0.2   0.2–0.4   0.4–0.6   0.6–0.8   0.8–1.0

Supplement: Supplementary file 5 — Supporting Information [file CTM2-16-e70732-s001.zip › LocusZoom/Sfig_rs6561321_locusZoom.pdf]

# LocusZoom plots of GWAS top lead SNP

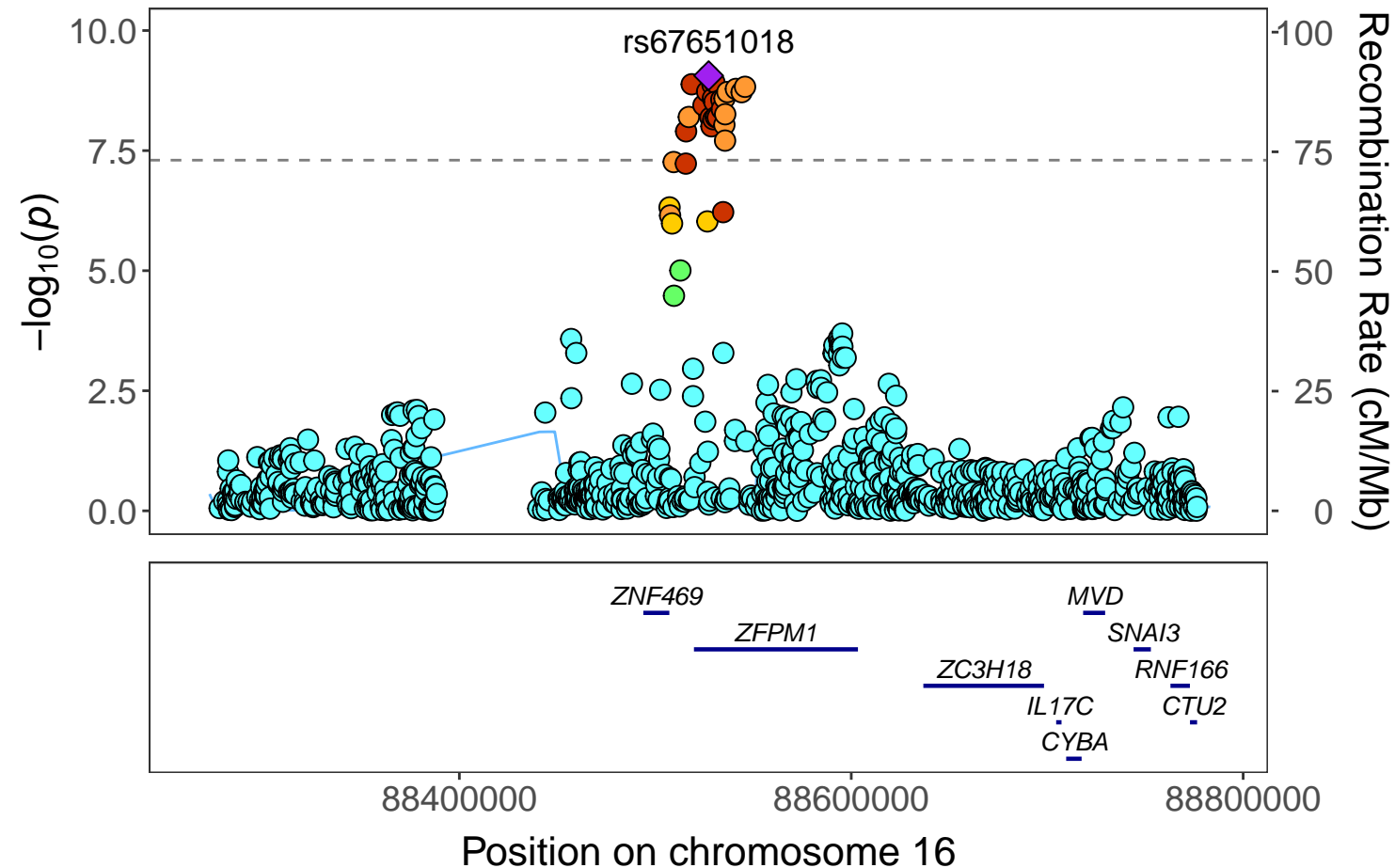

Supplement: Supplementary file 5 — Supporting Information [file CTM2-16-e70732-s001.zip › LocusZoom/Sfig_rs67651018_locusZoom.pdf]

# LocusZoom plots of GWAS top lead SNP

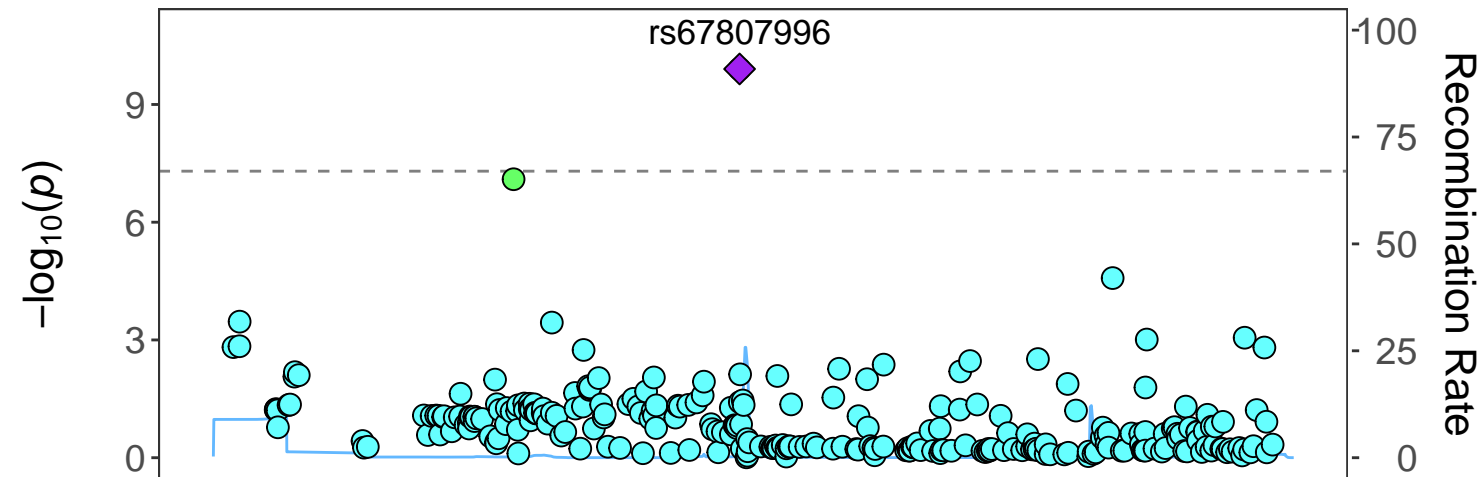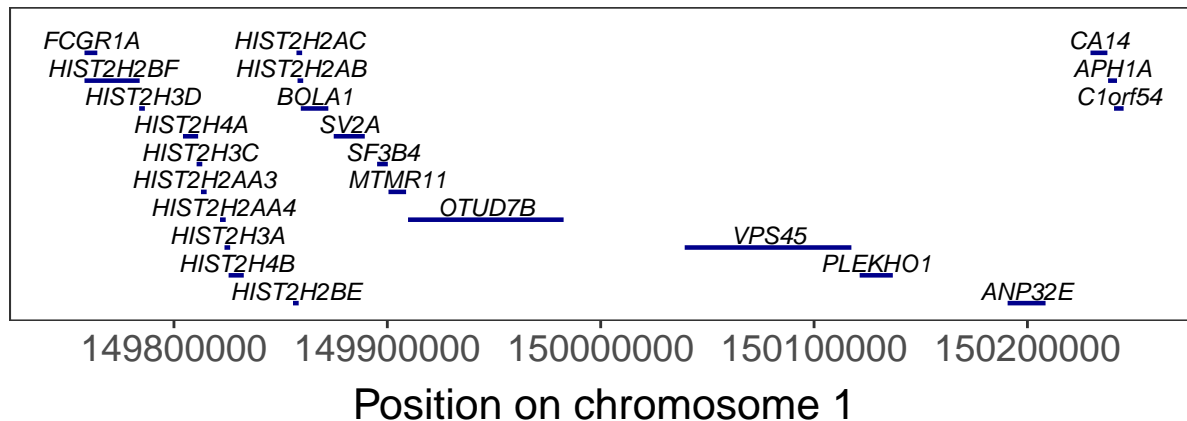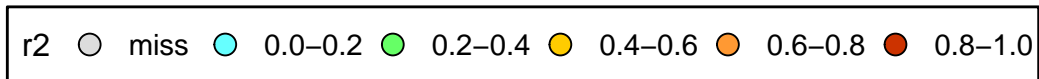

Supplement: Supplementary file 5 — Supporting Information [file CTM2-16-e70732-s001.zip › LocusZoom/Sfig_rs67807996_locusZoom.pdf]

# LocusZoom plots of GWAS top lead SNP

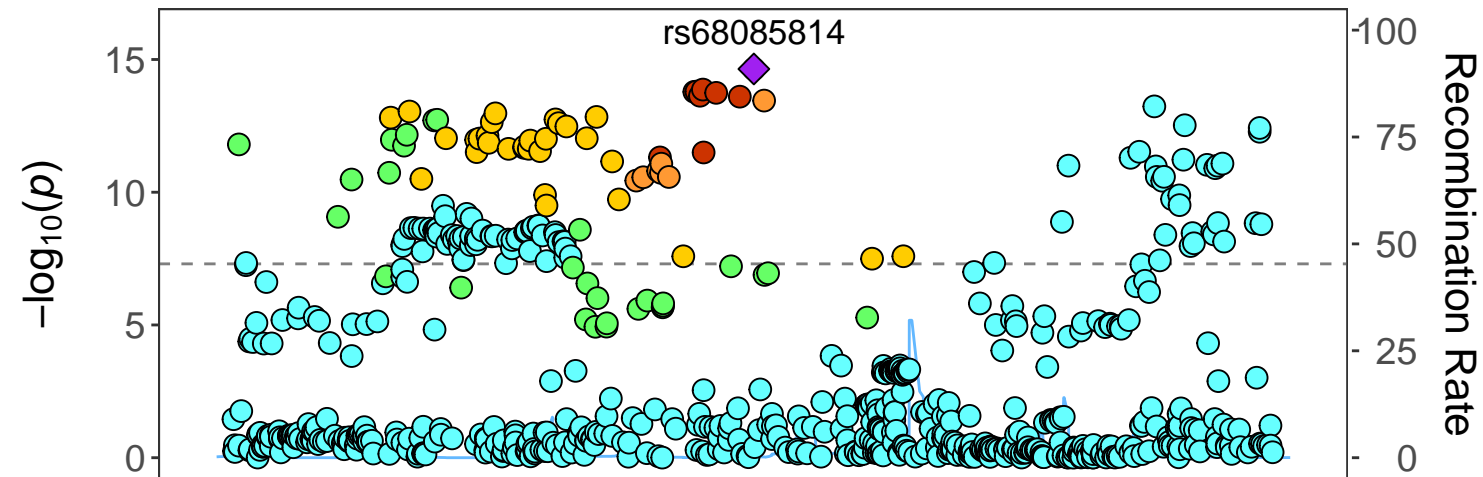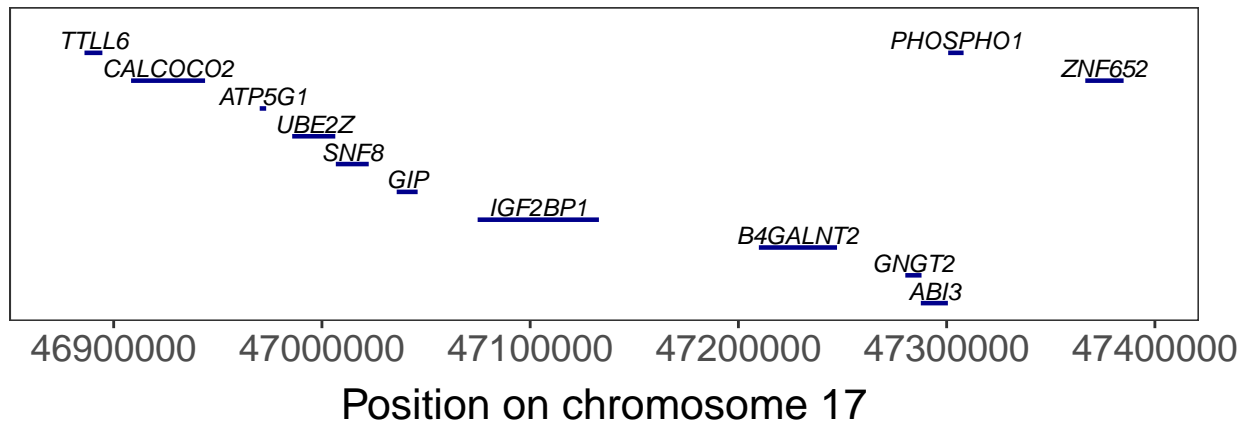

$r^2$     $\circ$  miss    $\circ$  0.0–0.2    $\circ$  0.2–0.4    $\circ$  0.4–0.6    $\circ$  0.6–0.8    $\circ$  0.8–1.0

Supplement: Supplementary file 5 — Supporting Information [file CTM2-16-e70732-s001.zip › LocusZoom/Sfig_rs68085814_locusZoom.pdf]

# LocusZoom plots of GWAS top lead SNP

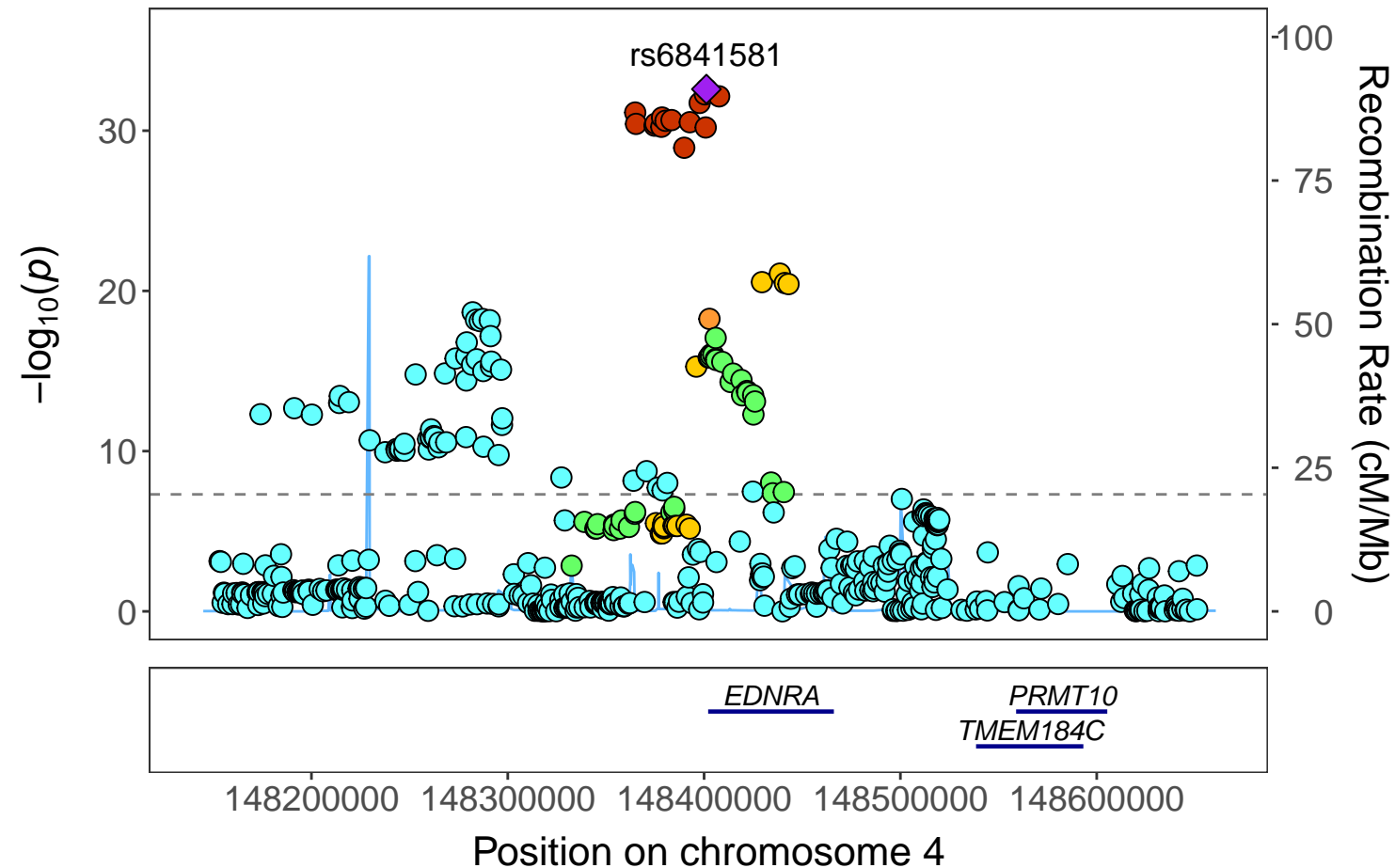

Supplement: Supplementary file 5 — Supporting Information [file CTM2-16-e70732-s001.zip › LocusZoom/Sfig_rs6841581_locusZoom.pdf]

# LocusZoom plots of GWAS top lead SNP

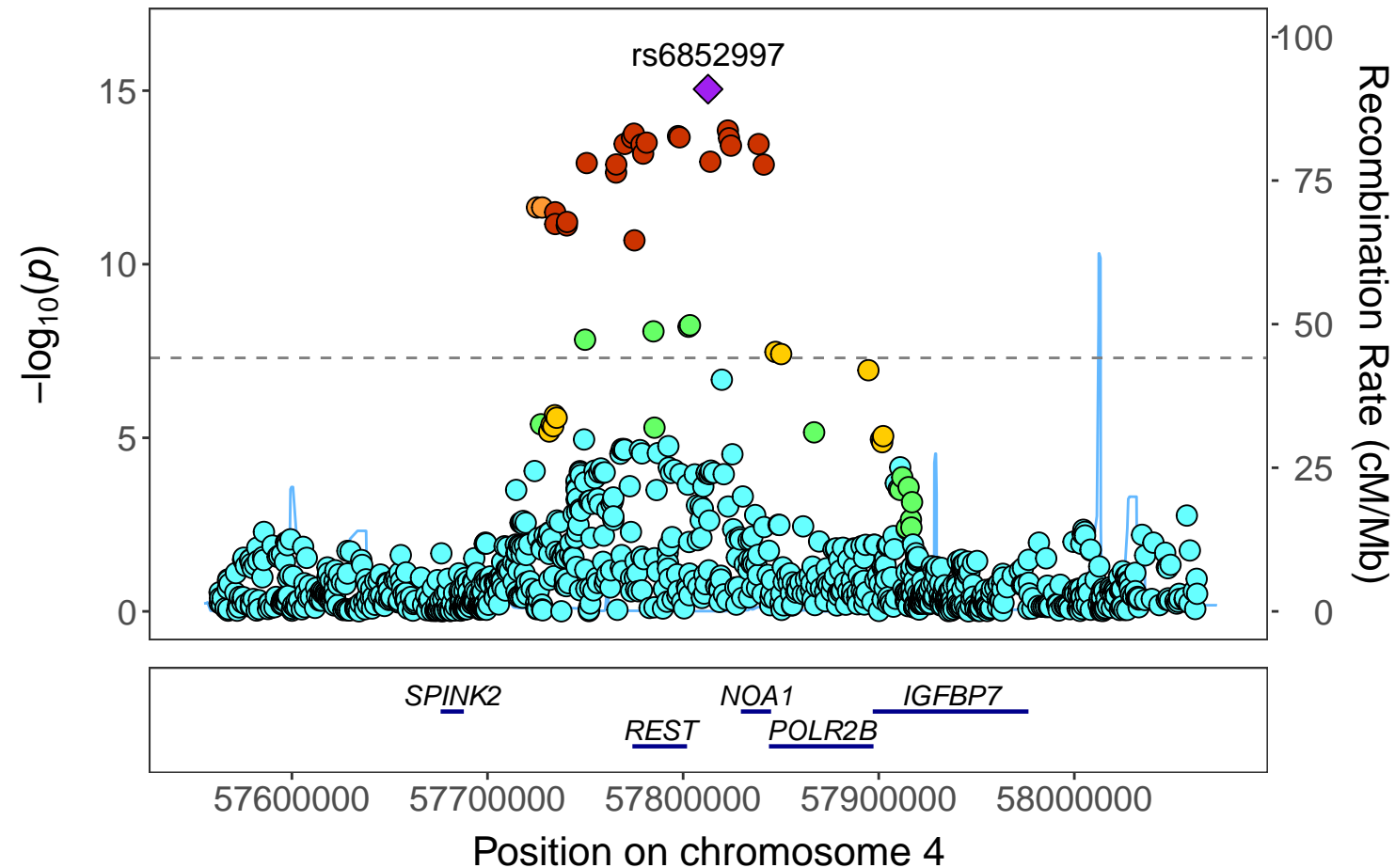

$r^2$  ○ miss ○ 0.0–0.2 ○ 0.2–0.4 ○ 0.4–0.6 ○ 0.6–0.8 ○ 0.8–1.0

Supplement: Supplementary file 5 — Supporting Information [file CTM2-16-e70732-s001.zip › LocusZoom/Sfig_rs6852997_locusZoom.pdf]

# LocusZoom plots of GWAS top lead SNP

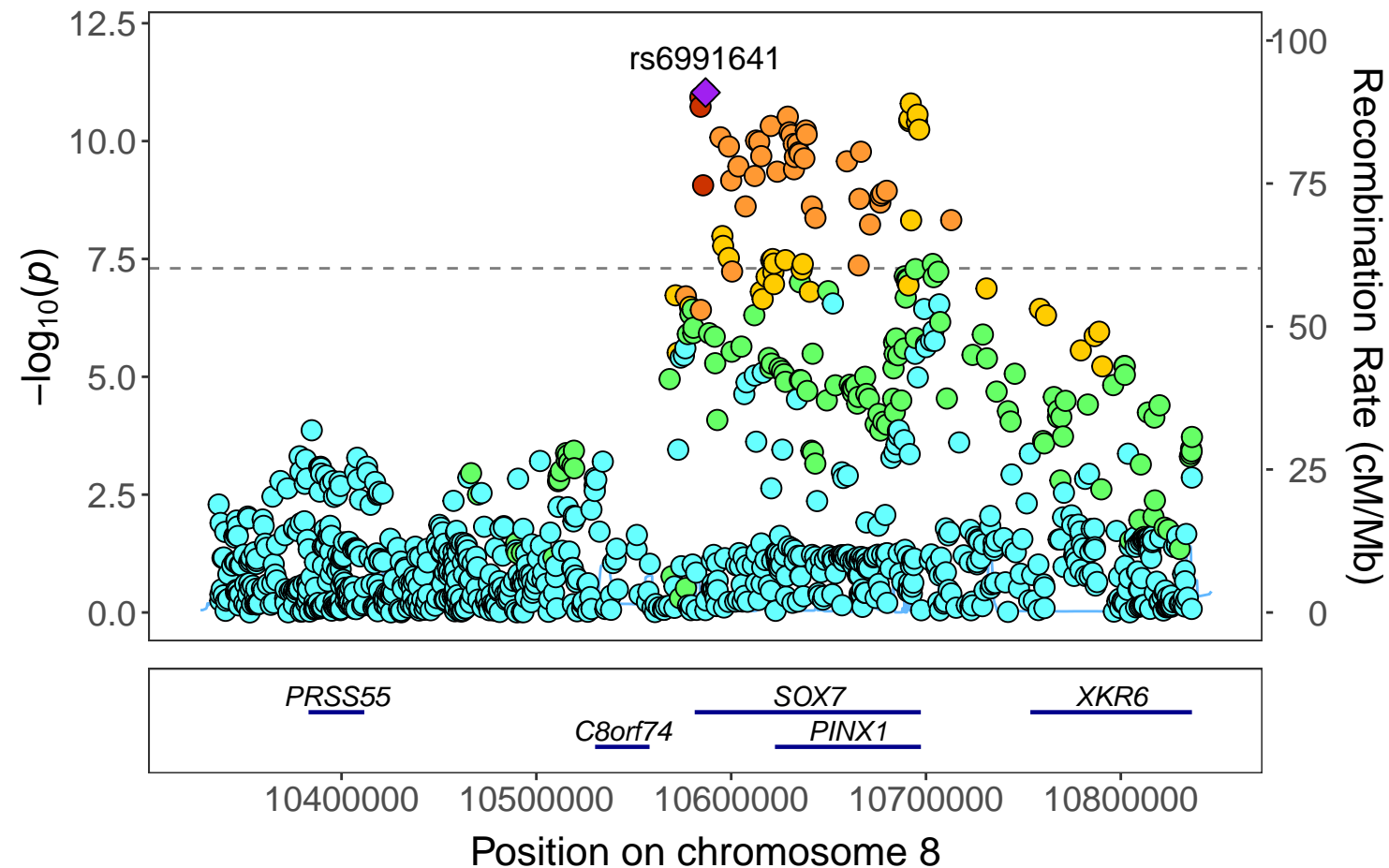

r2    miss    0.0–0.2    0.2–0.4    0.4–0.6    0.6–0.8    0.8–1.0

Supplement: Supplementary file 5 — Supporting Information [file CTM2-16-e70732-s001.zip › LocusZoom/Sfig_rs6991641_locusZoom.pdf]

# *LocusZoom plots of GWAS top lead SNP*

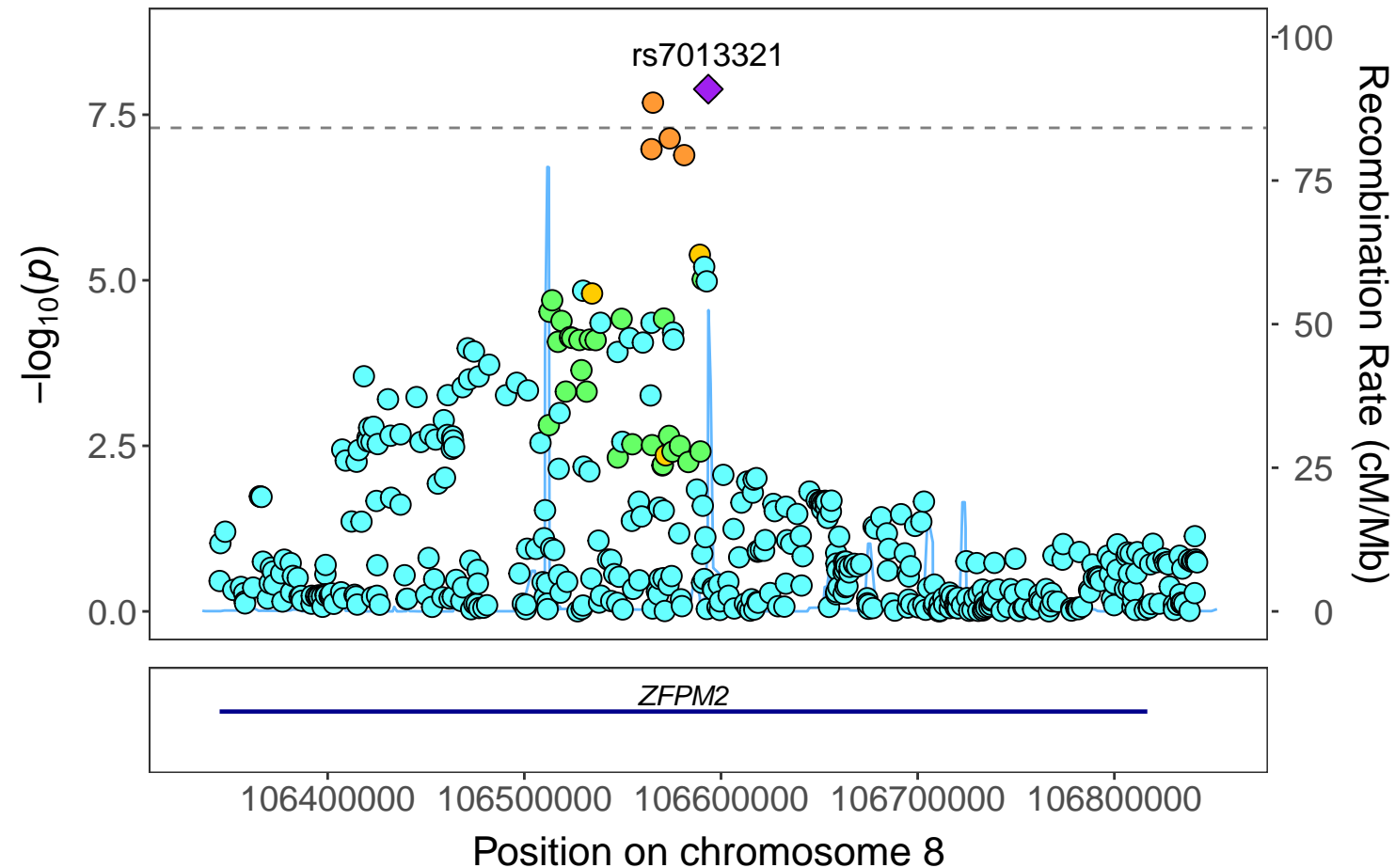

r2   miss   0.0-0.2   0.2-0.4   0.4-0.6   0.6-0.8   0.8-1.0

Supplement: Supplementary file 5 — Supporting Information [file CTM2-16-e70732-s001.zip › LocusZoom/Sfig_rs7013321_locusZoom.pdf]

# LocusZoom plots of GWAS top lead SNP

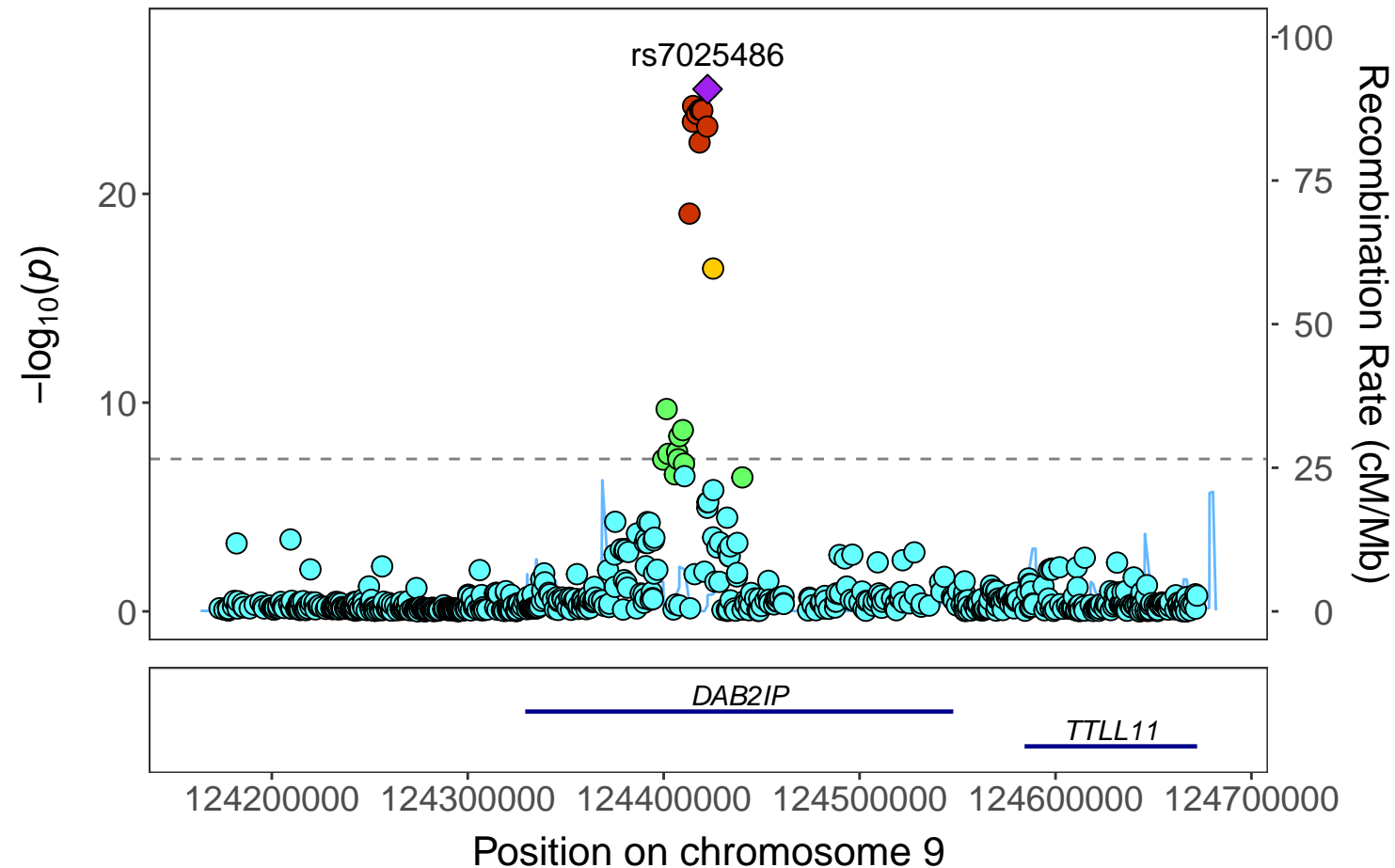

$r^2$    miss   0.0-0.2   0.2-0.4   0.4-0.6   0.6-0.8   0.8-1.0

Supplement: Supplementary file 5 — Supporting Information [file CTM2-16-e70732-s001.zip › LocusZoom/Sfig_rs7025486_locusZoom.pdf]

# LocusZoom plots of GWAS top lead SNP

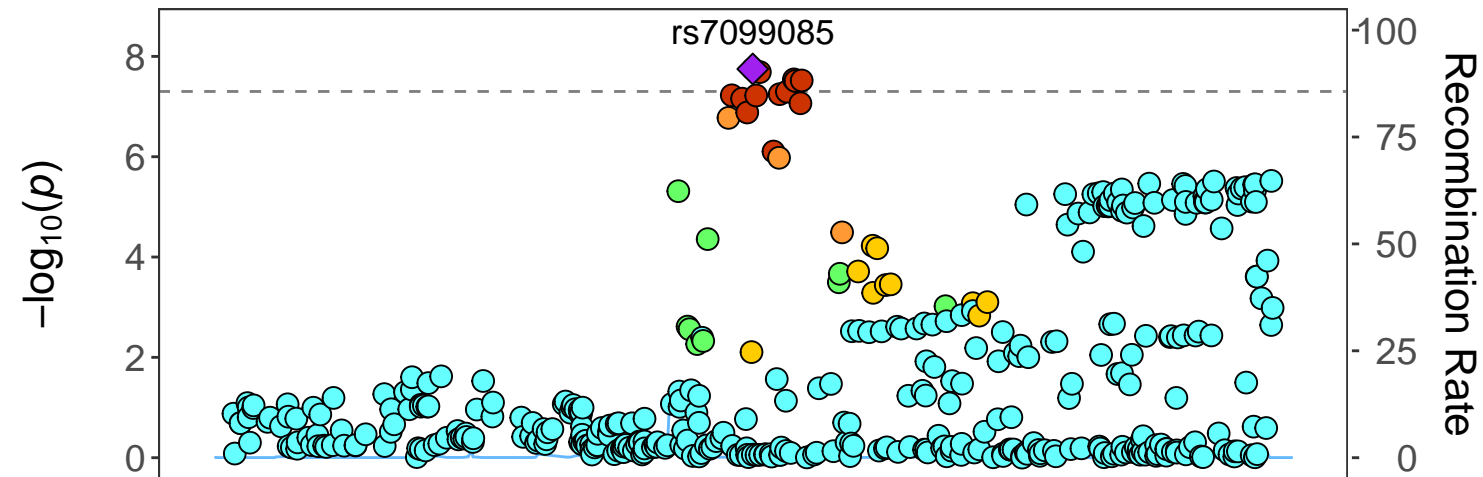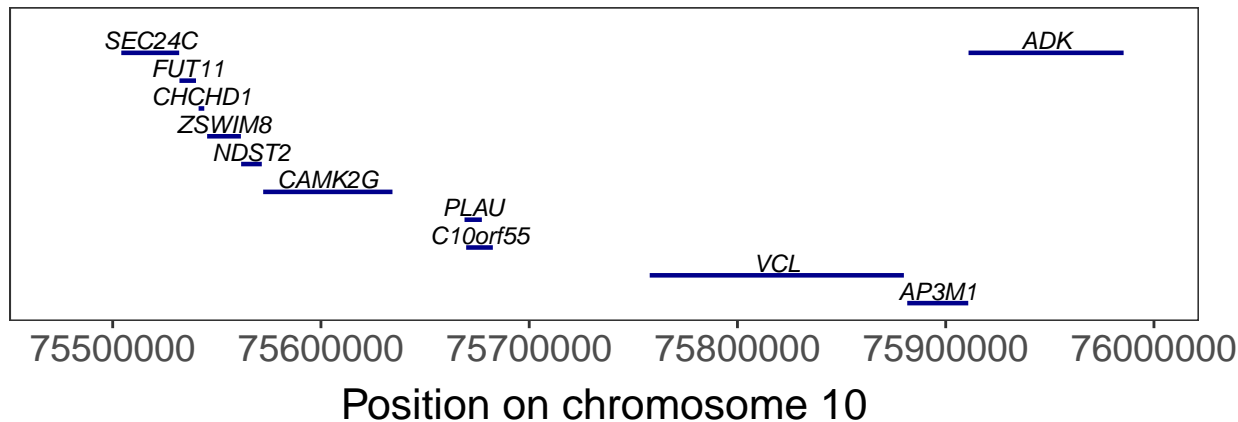

$r^2$    miss   cyan   0.0-0.2   green   0.2-0.4   yellow   0.4-0.6   orange   0.6-0.8   dark red   0.8-1.0

Supplement: Supplementary file 5 — Supporting Information [file CTM2-16-e70732-s001.zip › LocusZoom/Sfig_rs7099085_locusZoom.pdf]

# LocusZoom plots of GWAS top lead SNP

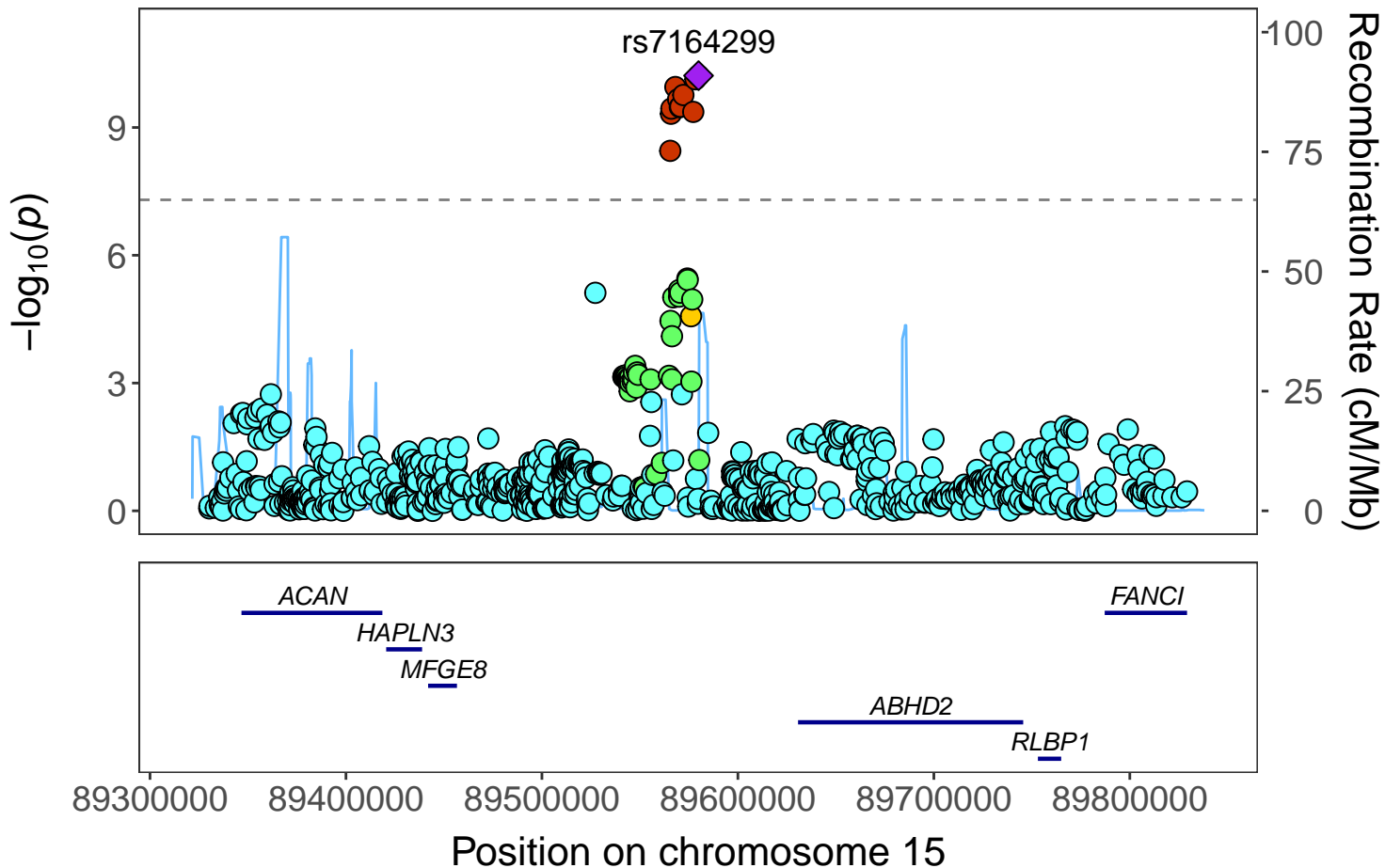

$r^2$     $\circ$  miss    $\circ$  0.0–0.2    $\circ$  0.2–0.4    $\circ$  0.4–0.6    $\circ$  0.6–0.8    $\circ$  0.8–1.0

Supplement: Supplementary file 5 — Supporting Information [file CTM2-16-e70732-s001.zip › LocusZoom/Sfig_rs7164299_locusZoom.pdf]

# *LocusZoom plots of GWAS top lead SNP*

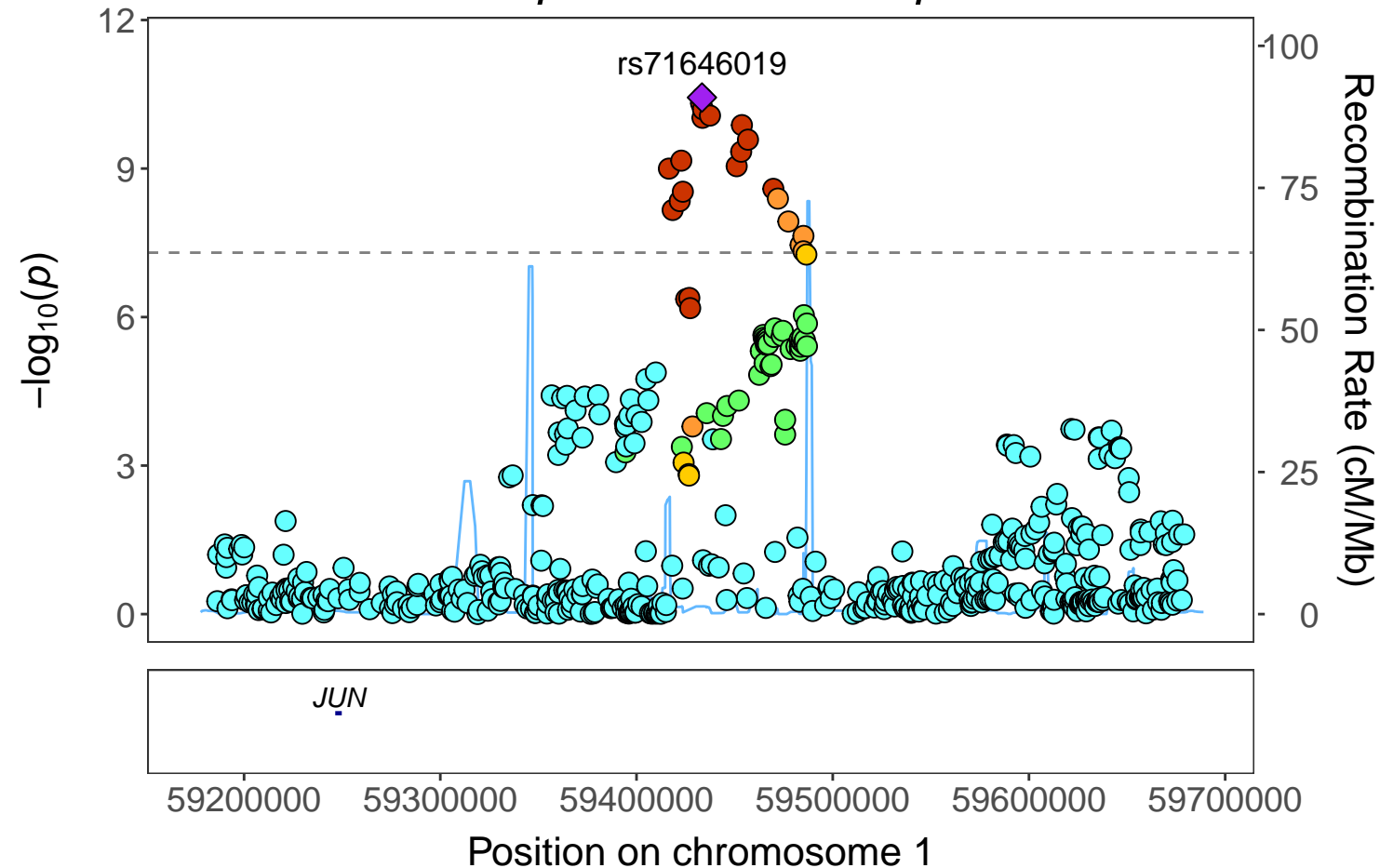

r2    miss    0.0–0.2    0.2–0.4    0.4–0.6    0.6–0.8    0.8–1.0

Supplement: Supplementary file 5 — Supporting Information [file CTM2-16-e70732-s001.zip › LocusZoom/Sfig_rs71646019_locusZoom.pdf]

# LocusZoom plots of GWAS top lead SNP

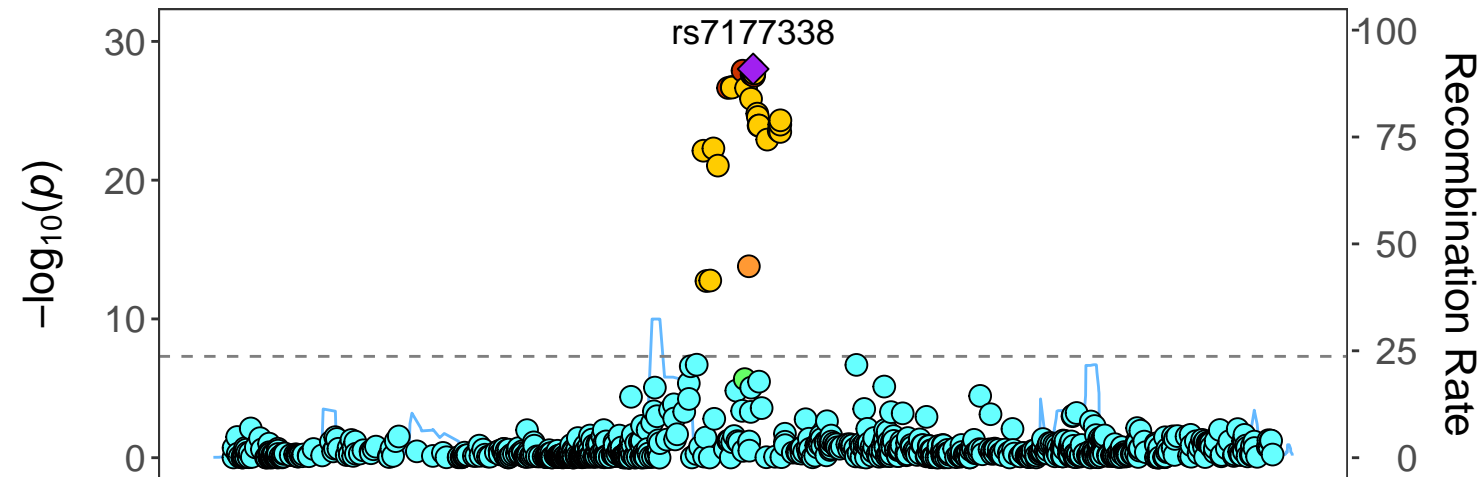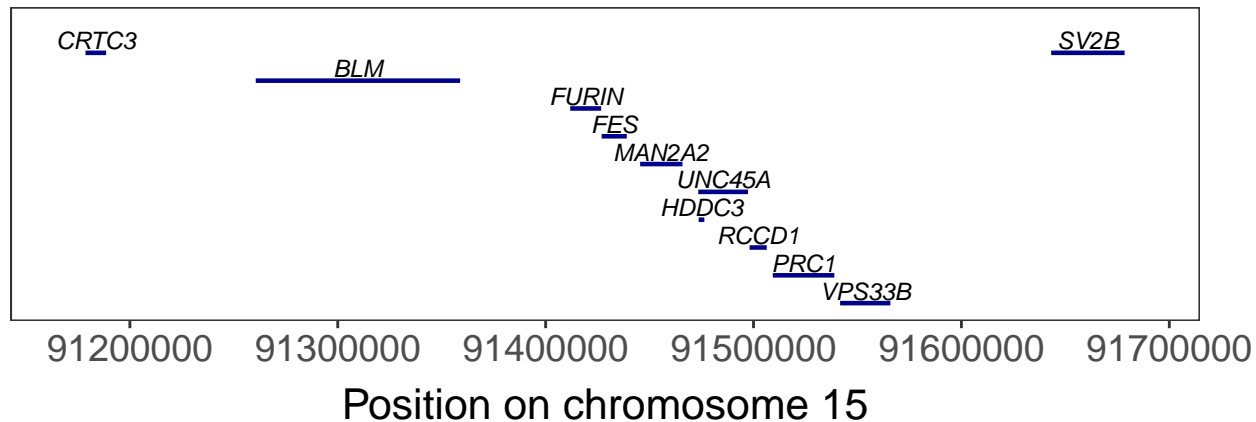

r2    miss    0.0–0.2    0.2–0.4    0.4–0.6    0.6–0.8    0.8–1.0

Supplement: Supplementary file 5 — Supporting Information [file CTM2-16-e70732-s001.zip › LocusZoom/Sfig_rs7177338_locusZoom.pdf]

# LocusZoom plots of GWAS top lead SNP

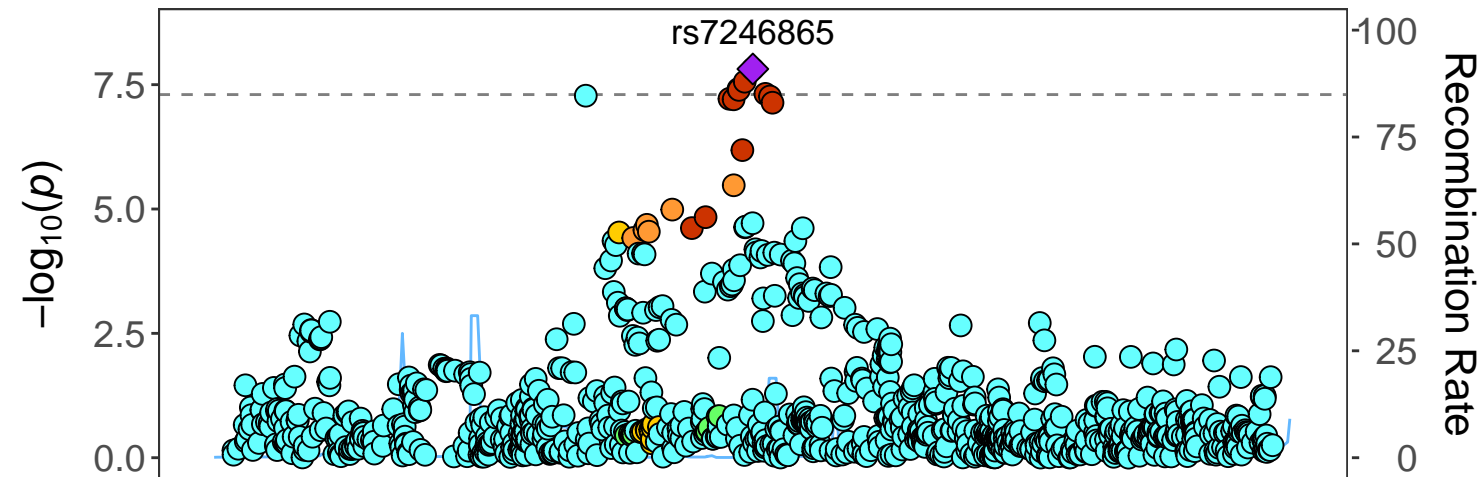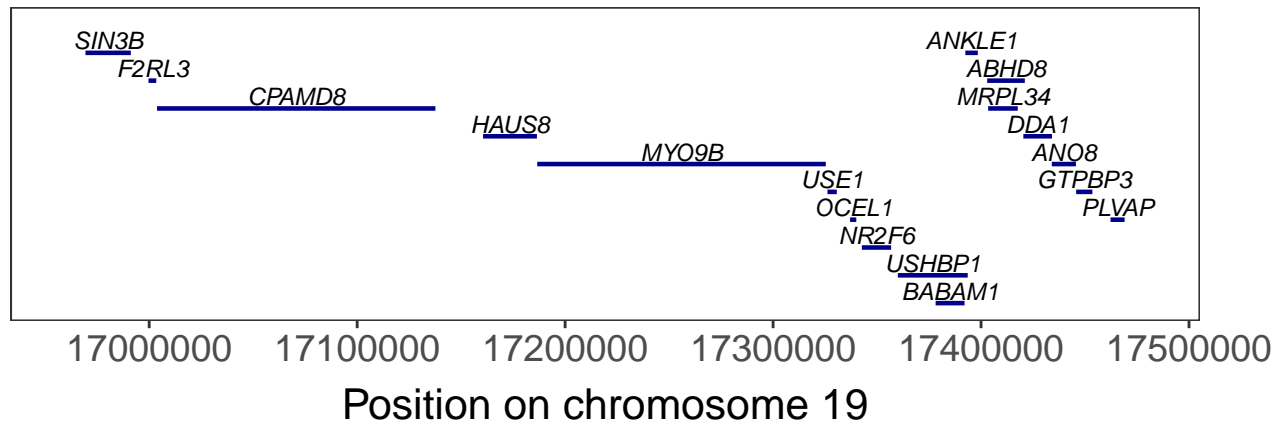

r2    miss    0.0–0.2    0.2–0.4    0.4–0.6    0.6–0.8    0.8–1.0

Supplement: Supplementary file 5 — Supporting Information [file CTM2-16-e70732-s001.zip › LocusZoom/Sfig_rs7246865_locusZoom.pdf]

# LocusZoom plots of GWAS top lead SNP

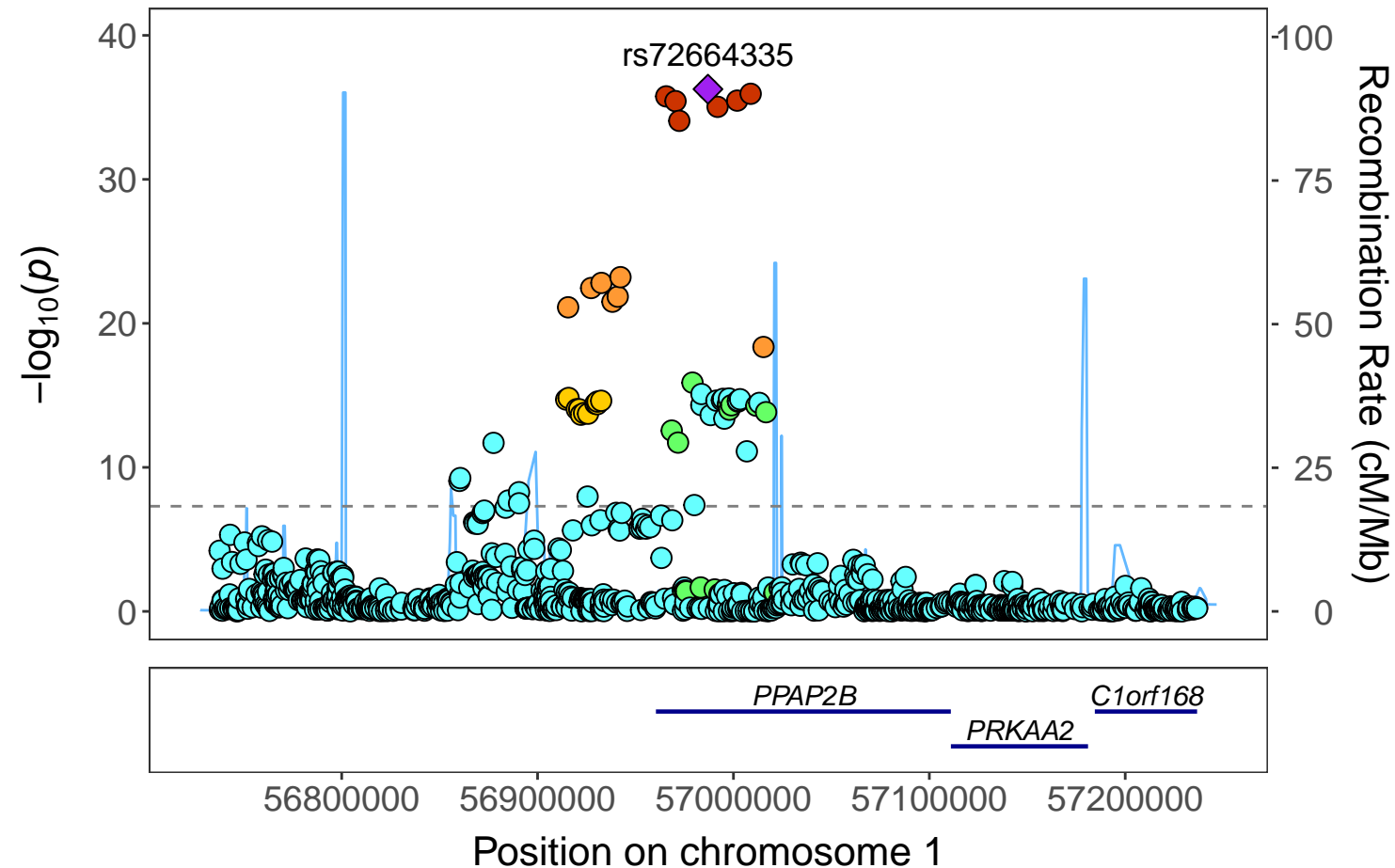

$r^2$     $\circ$  miss    $\circ$  0.0–0.2    $\circ$  0.2–0.4    $\circ$  0.4–0.6    $\circ$  0.6–0.8    $\circ$  0.8–1.0

Supplement: Supplementary file 5 — Supporting Information [file CTM2-16-e70732-s001.zip › LocusZoom/Sfig_rs72664335_locusZoom.pdf]

# LocusZoom plots of GWAS top lead SNP

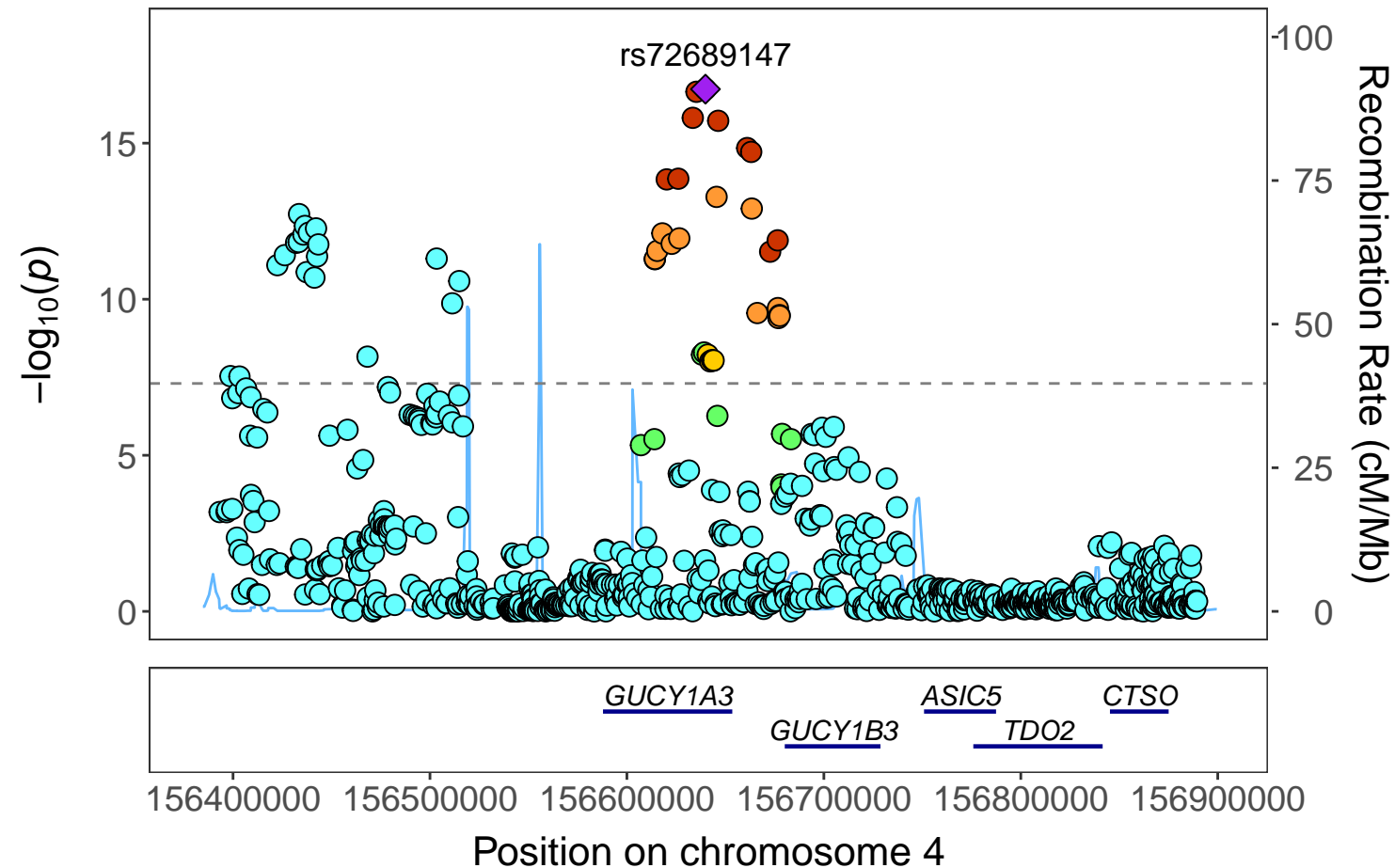

$r^2$    miss   cyan   0.0–0.2   green   0.2–0.4   yellow   0.4–0.6   orange   0.6–0.8   dark red   0.8–1.0

Supplement: Supplementary file 5 — Supporting Information [file CTM2-16-e70732-s001.zip › LocusZoom/Sfig_rs72689147_locusZoom.pdf]

# LocusZoom plots of GWAS top lead SNP

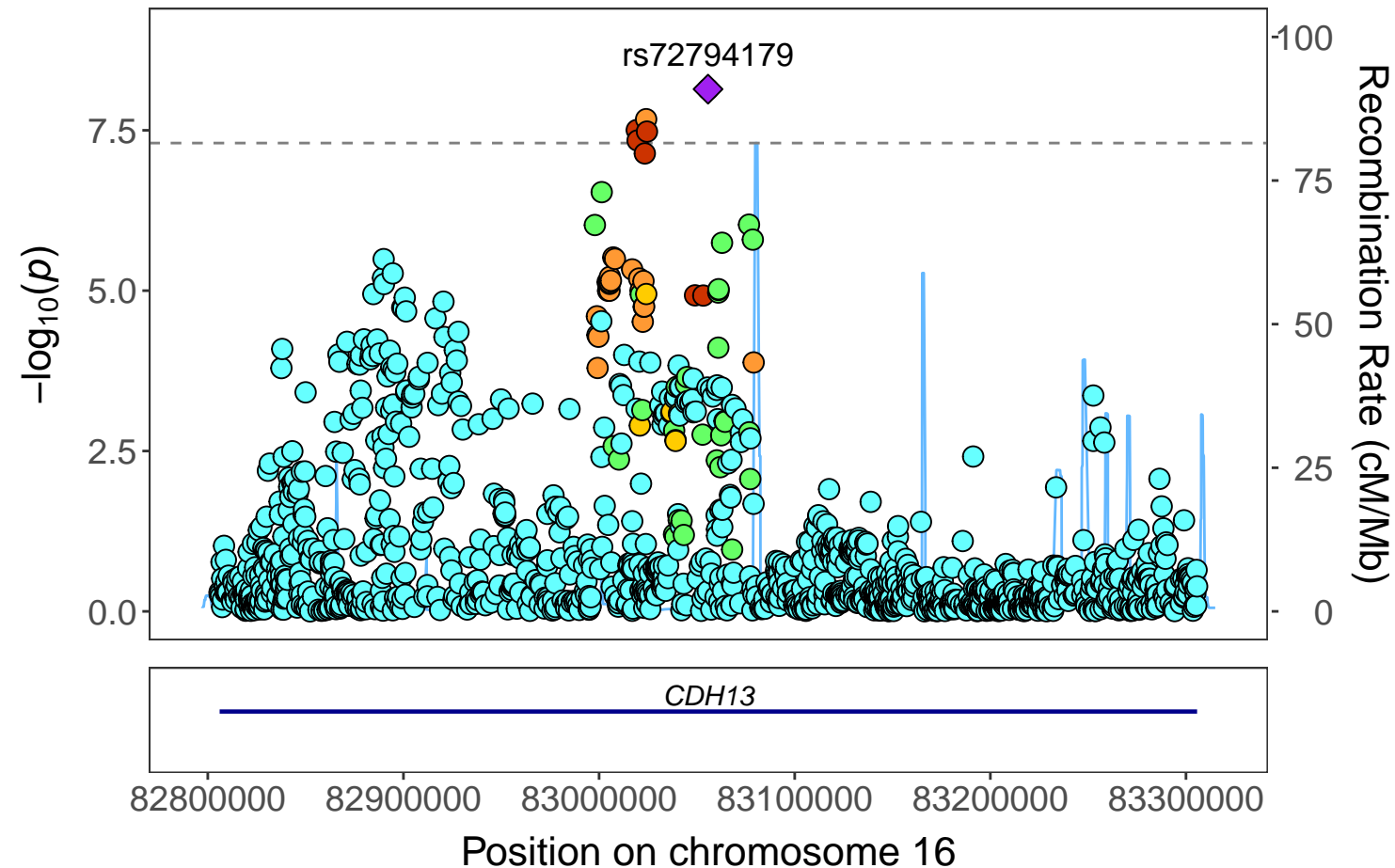

$r^2$    miss   0.0–0.2   0.2–0.4   0.4–0.6   0.6–0.8   0.8–1.0

Supplement: Supplementary file 5 — Supporting Information [file CTM2-16-e70732-s001.zip › LocusZoom/Sfig_rs72794179_locusZoom.pdf]

# LocusZoom plots of GWAS top lead SNP

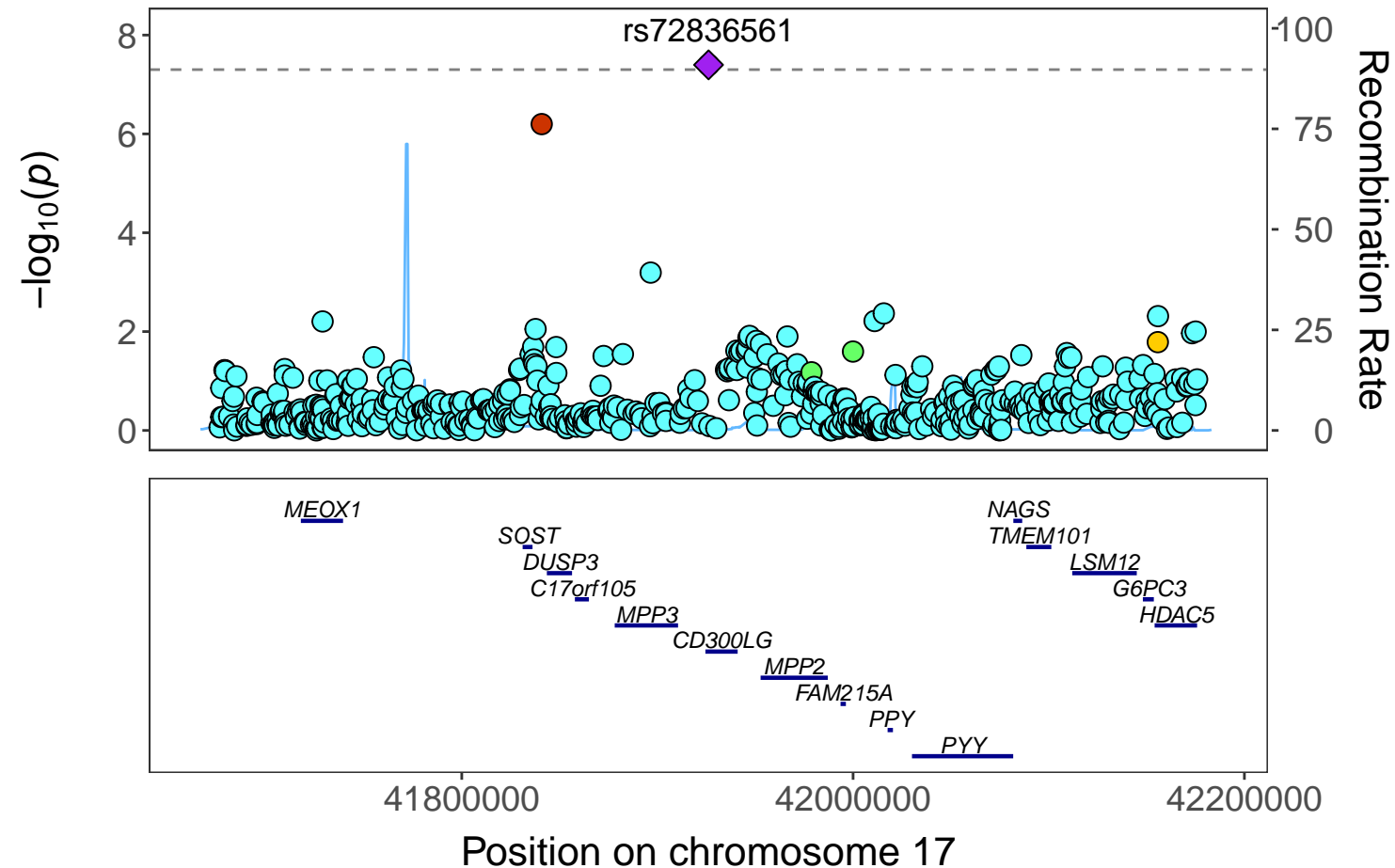

$r^2$  ○ miss ● 0.0–0.2 ● 0.2–0.4 ● 0.4–0.6 ● 0.6–0.8 ● 0.8–1.0

Supplement: Supplementary file 5 — Supporting Information [file CTM2-16-e70732-s001.zip › LocusZoom/Sfig_rs72836561_locusZoom.pdf]

# LocusZoom plots of GWAS top lead SNP

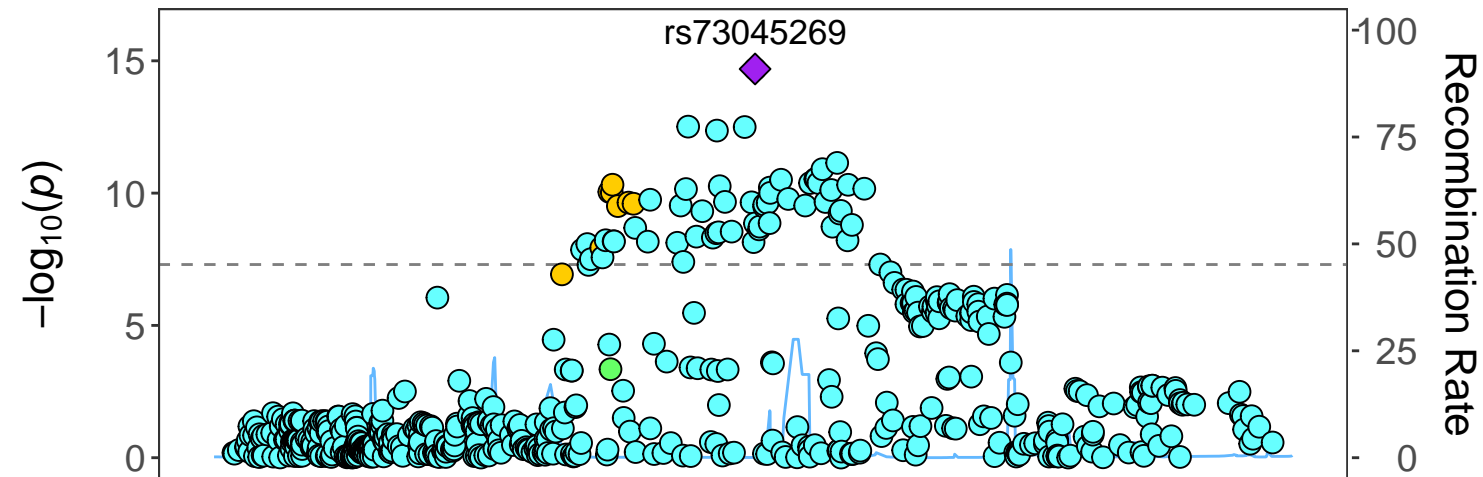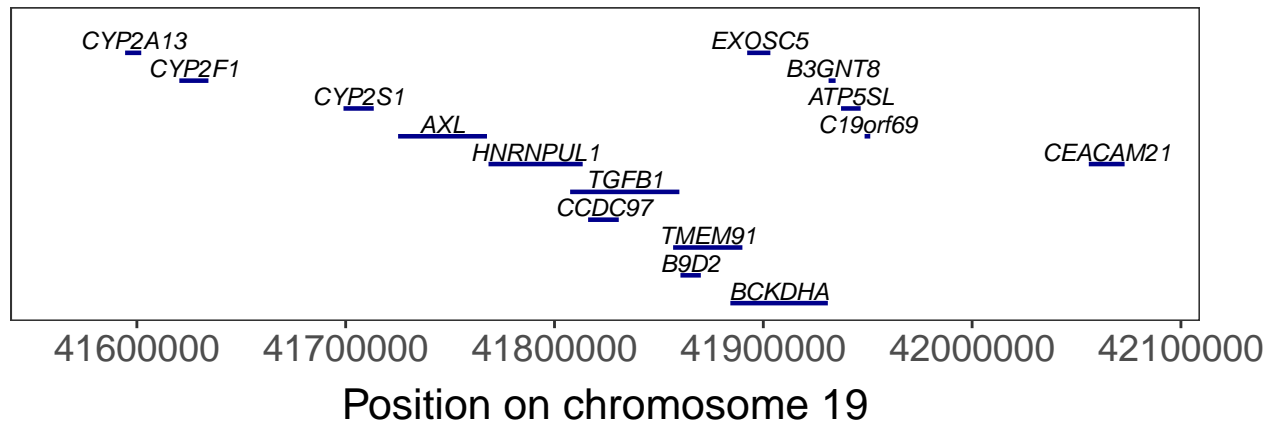

r2    miss    0.0–0.2    0.2–0.4    0.4–0.6    0.6–0.8    0.8–1.0

Supplement: Supplementary file 5 — Supporting Information [file CTM2-16-e70732-s001.zip › LocusZoom/Sfig_rs73045269_locusZoom.pdf]

# *LocusZoom plots of GWAS top lead SNP*

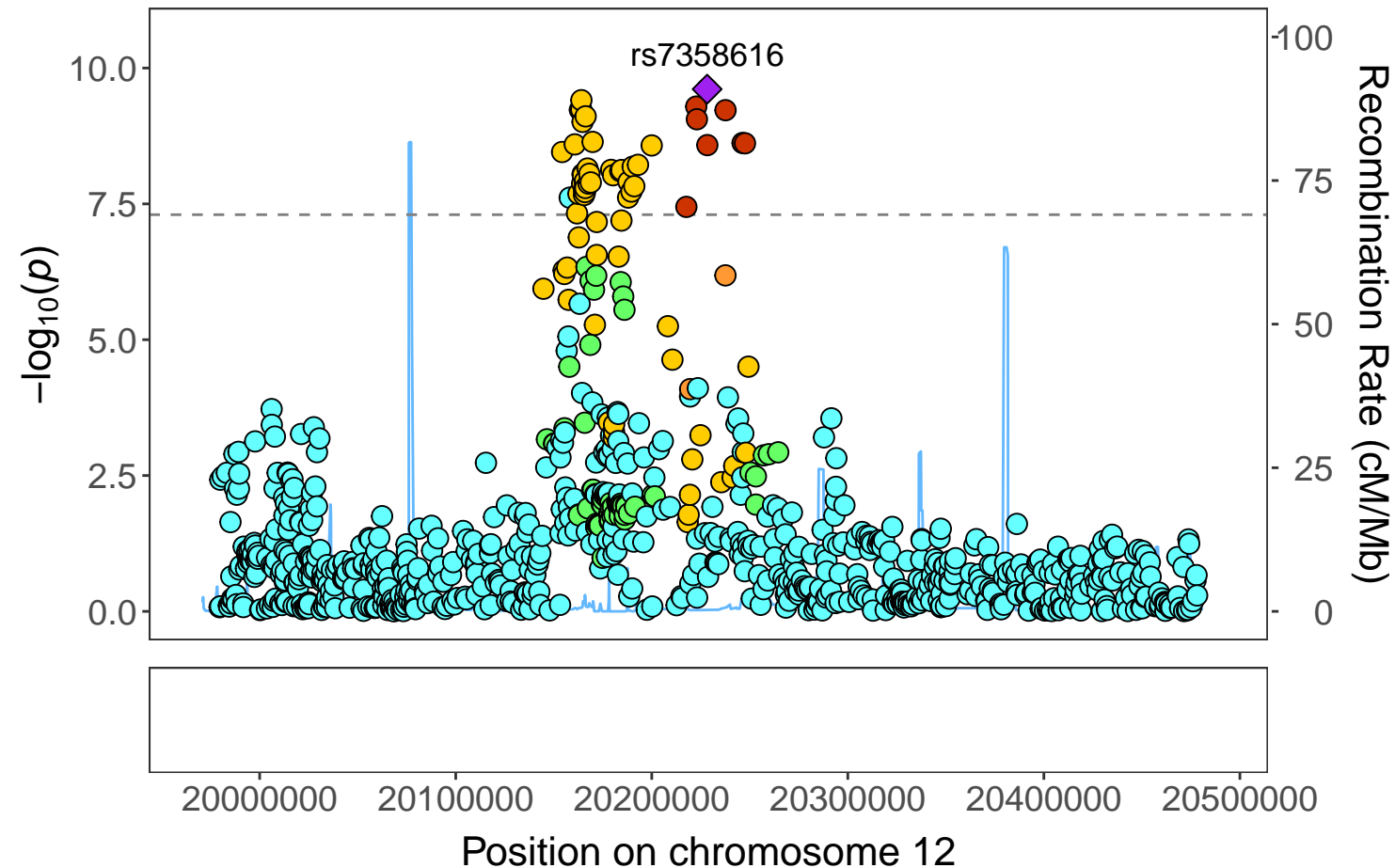

$r^2$    miss   0.0-0.2   0.2-0.4   0.4-0.6   0.6-0.8   0.8-1.0

Supplement: Supplementary file 5 — Supporting Information [file CTM2-16-e70732-s001.zip › LocusZoom/Sfig_rs7358616_locusZoom.pdf]

# LocusZoom plots of GWAS top lead SNP

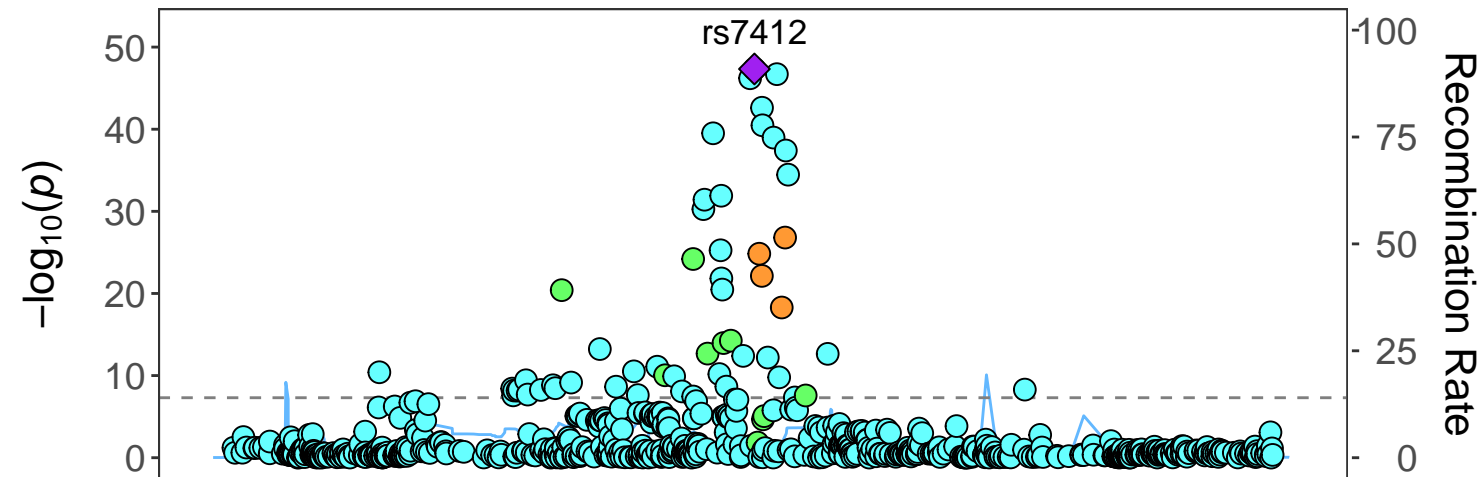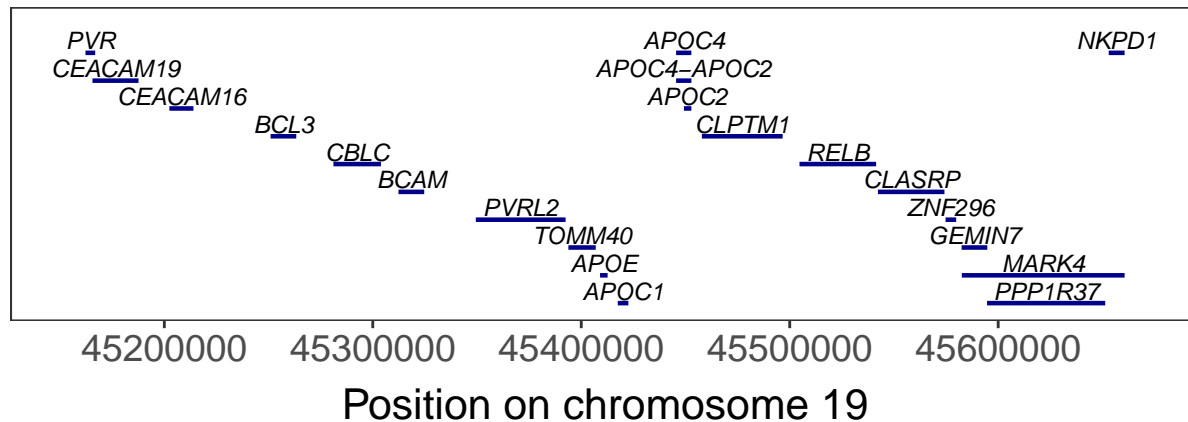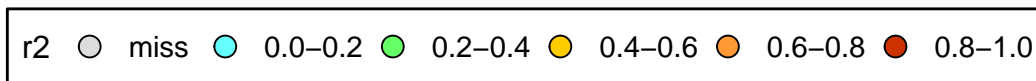

Supplement: Supplementary file 5 — Supporting Information [file CTM2-16-e70732-s001.zip › LocusZoom/Sfig_rs7412_locusZoom.pdf]

# LocusZoom plots of GWAS top lead SNP

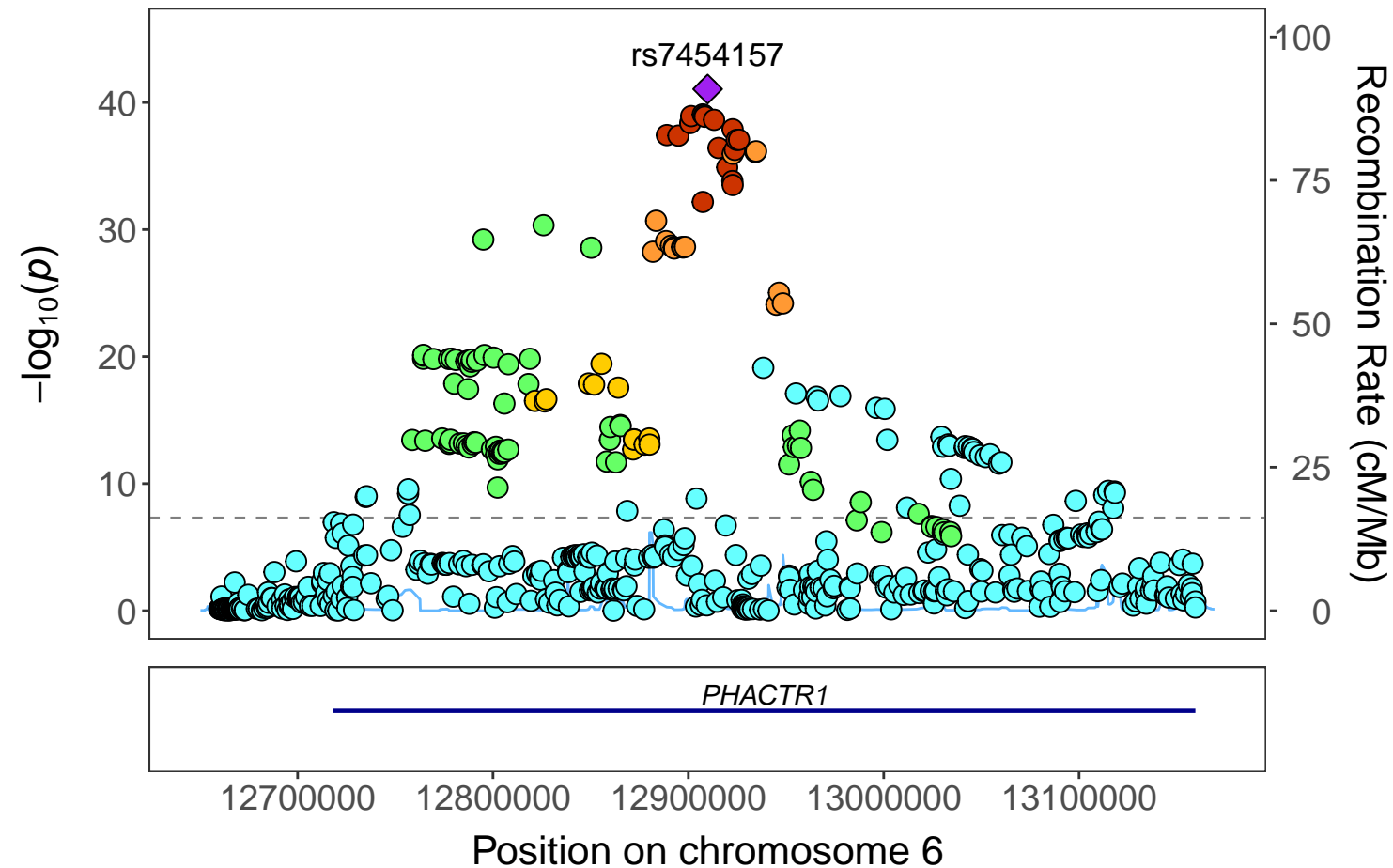

$r^2$    miss   0.0-0.2   0.2-0.4   0.4-0.6   0.6-0.8   0.8-1.0

Supplement: Supplementary file 5 — Supporting Information [file CTM2-16-e70732-s001.zip › LocusZoom/Sfig_rs7454157_locusZoom.pdf]

# LocusZoom plots of GWAS top lead SNP

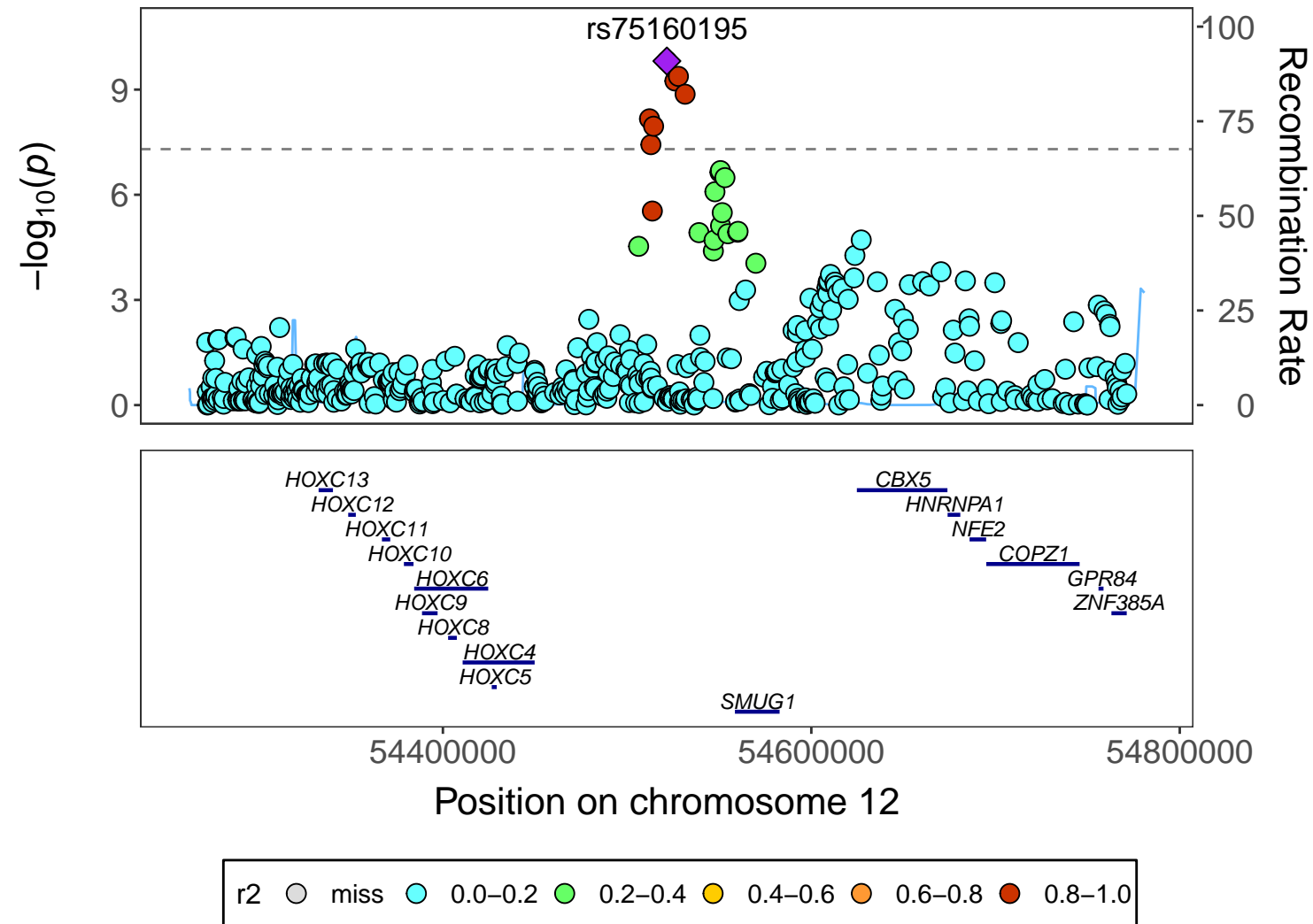

Supplement: Supplementary file 5 — Supporting Information [file CTM2-16-e70732-s001.zip › LocusZoom/Sfig_rs75160195_locusZoom.pdf]

# LocusZoom plots of GWAS top lead SNP

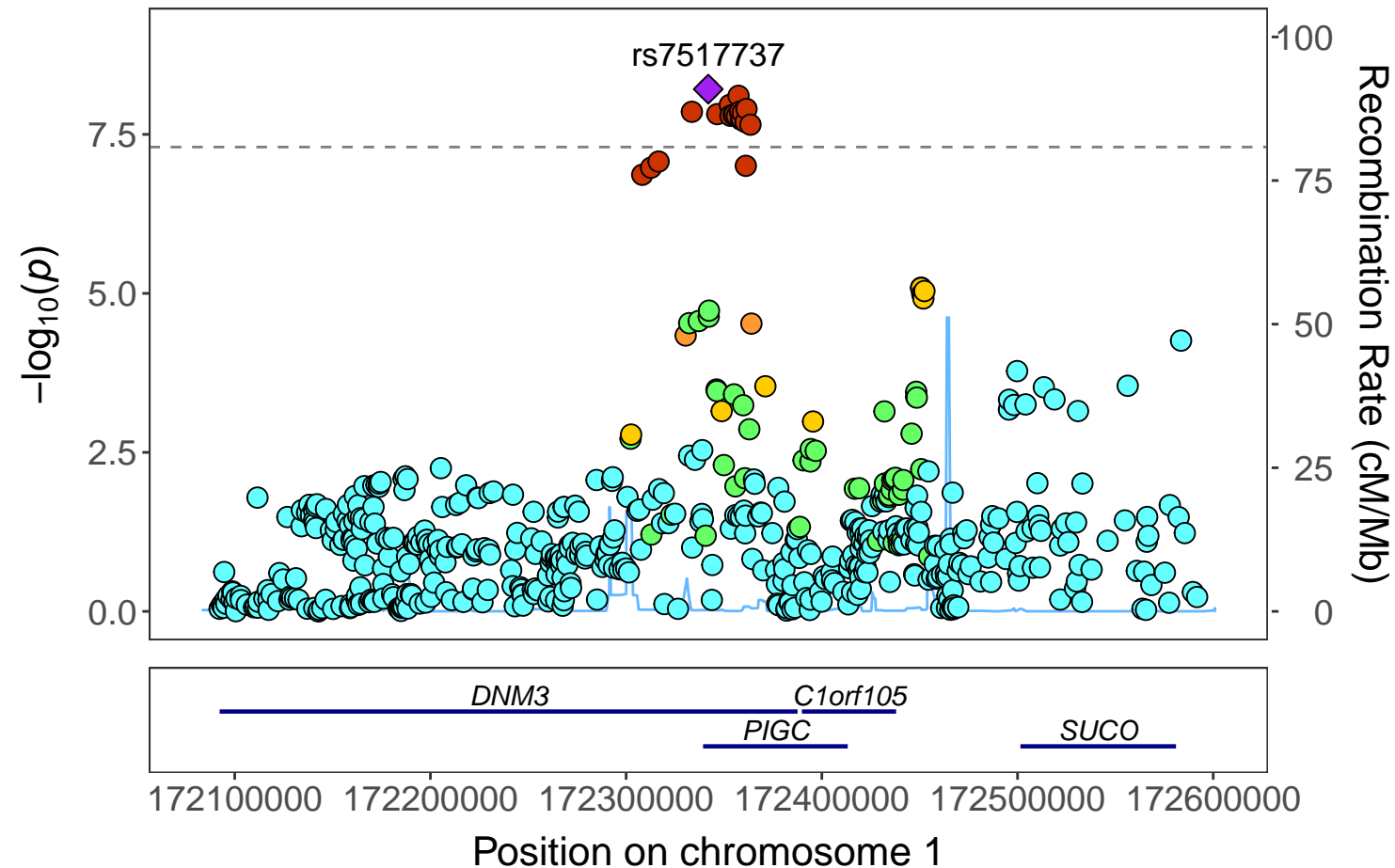

r2   miss   0.0-0.2   0.2-0.4   0.4-0.6   0.6-0.8   0.8-1.0

Supplement: Supplementary file 5 — Supporting Information [file CTM2-16-e70732-s001.zip › LocusZoom/Sfig_rs7517737_locusZoom.pdf]

# LocusZoom plots of GWAS top lead SNP

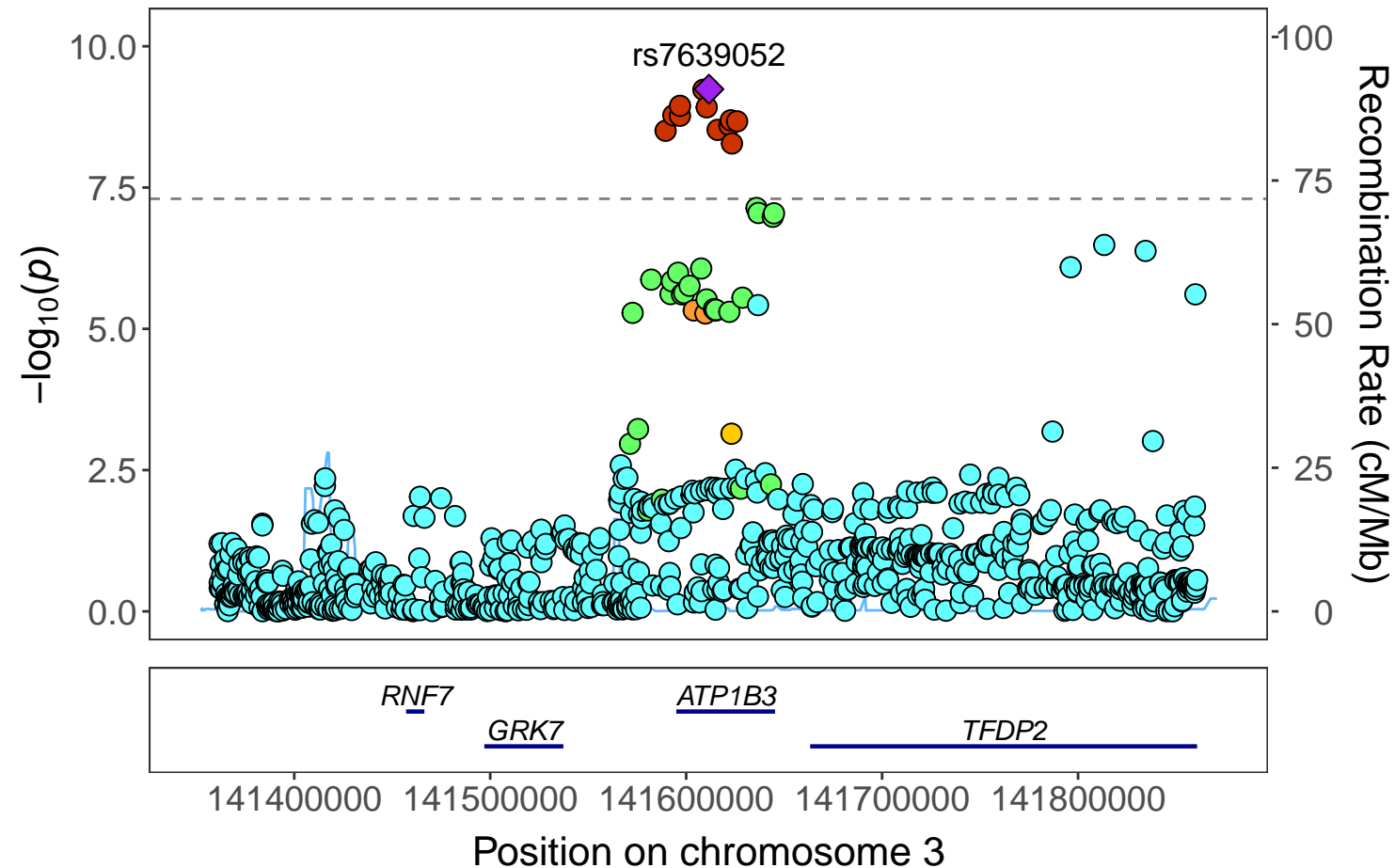

r2    miss    0.0–0.2    0.2–0.4    0.4–0.6    0.6–0.8    0.8–1.0

Supplement: Supplementary file 5 — Supporting Information [file CTM2-16-e70732-s001.zip › LocusZoom/Sfig_rs7639052_locusZoom.pdf]

# LocusZoom plots of GWAS top lead SNP

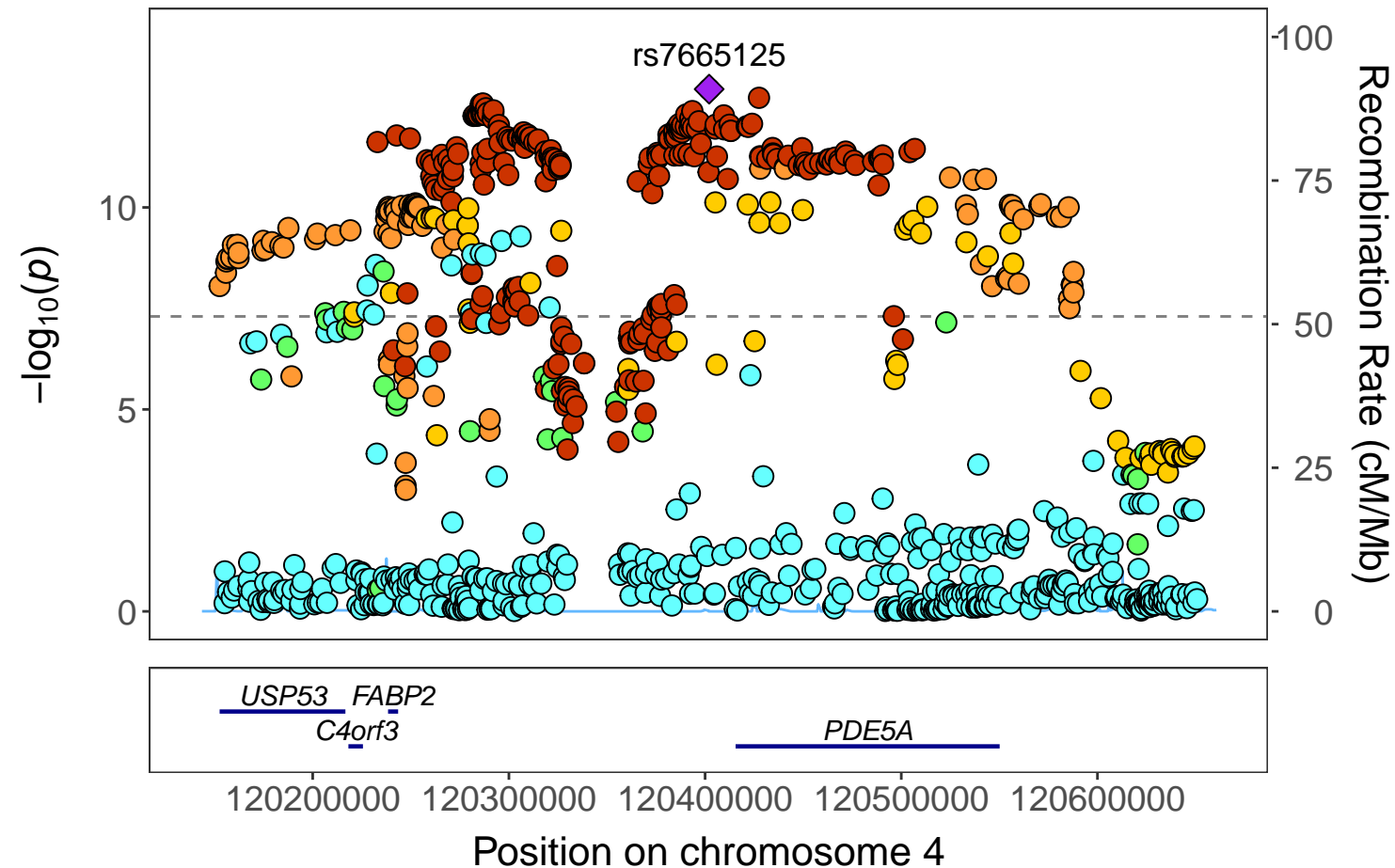

Supplement: Supplementary file 5 — Supporting Information [file CTM2-16-e70732-s001.zip › LocusZoom/Sfig_rs7665125_locusZoom.pdf]

# LocusZoom plots of GWAS top lead SNP

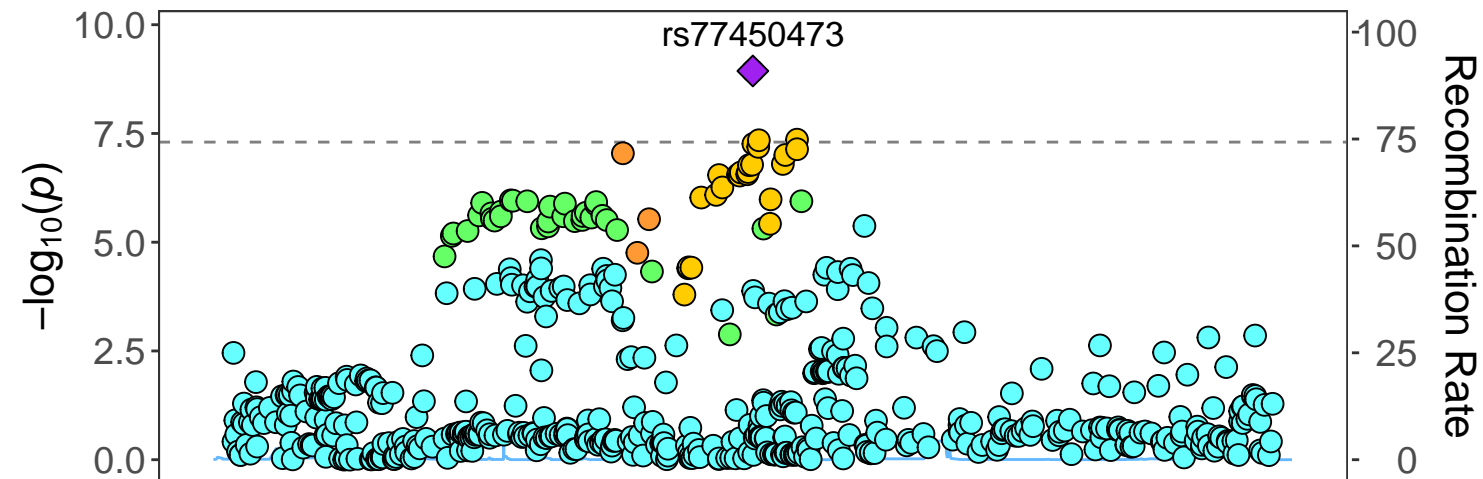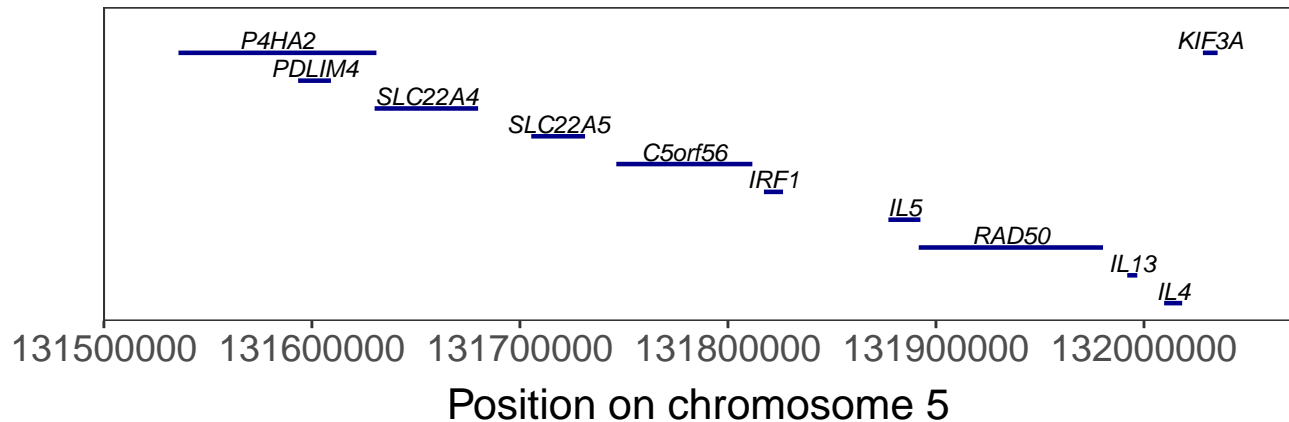

r2    miss    0.0–0.2    0.2–0.4    0.4–0.6    0.6–0.8    0.8–1.0

Supplement: Supplementary file 5 — Supporting Information [file CTM2-16-e70732-s001.zip › LocusZoom/Sfig_rs77450473_locusZoom.pdf]

# LocusZoom plots of GWAS top lead SNP

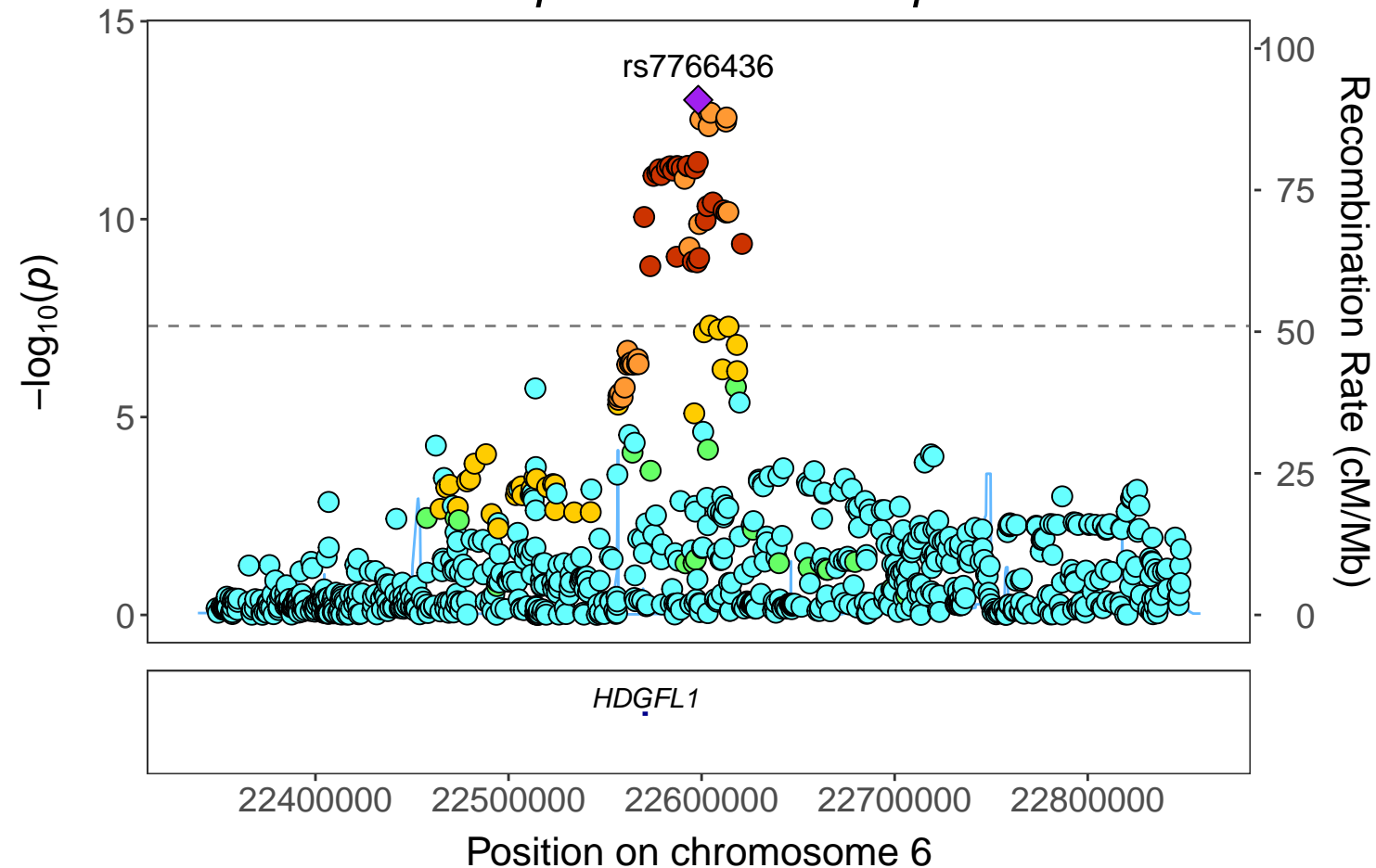

$r^2$    miss   cyan   0.0–0.2   green   0.2–0.4   yellow   0.4–0.6   orange   0.6–0.8   red   0.8–1.0

Supplement: Supplementary file 5 — Supporting Information [file CTM2-16-e70732-s001.zip › LocusZoom/Sfig_rs7766436_locusZoom.pdf]

# LocusZoom plots of GWAS top lead SNP

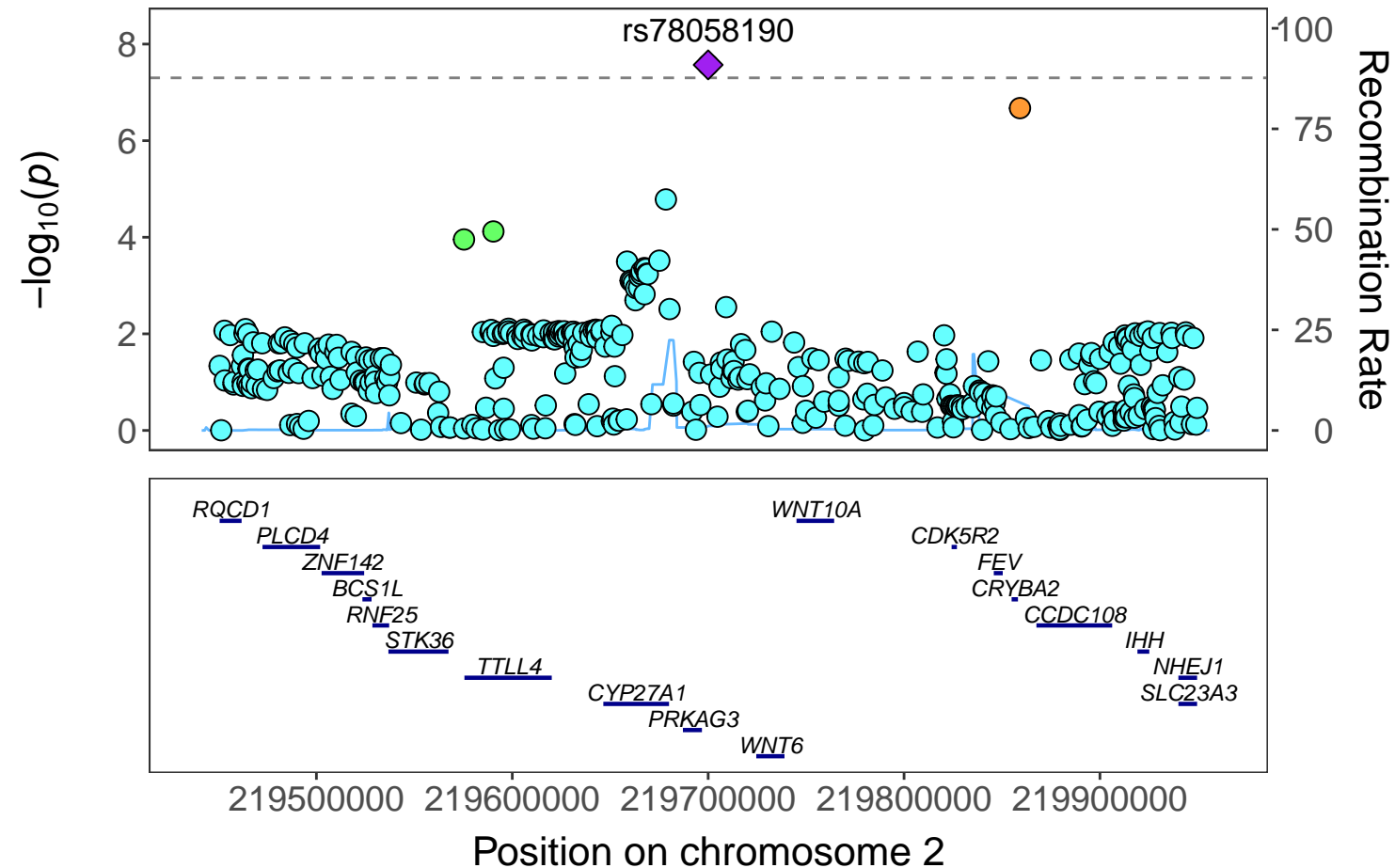

Supplement: Supplementary file 5 — Supporting Information [file CTM2-16-e70732-s001.zip › LocusZoom/Sfig_rs78058190_locusZoom.pdf]

# LocusZoom plots of GWAS top lead SNP

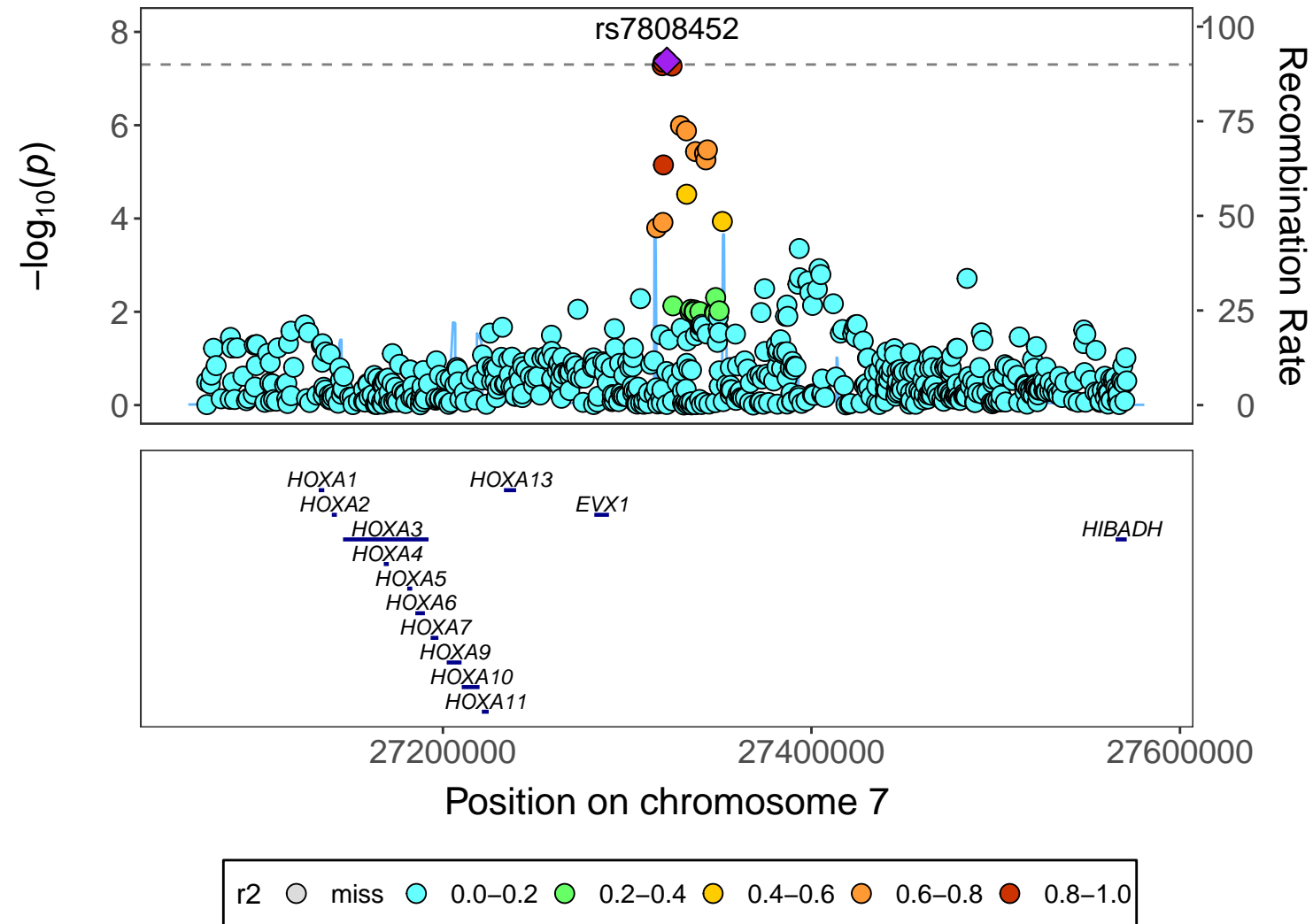

Supplement: Supplementary file 5 — Supporting Information [file CTM2-16-e70732-s001.zip › LocusZoom/Sfig_rs7808452_locusZoom.pdf]

# LocusZoom plots of GWAS top lead SNP

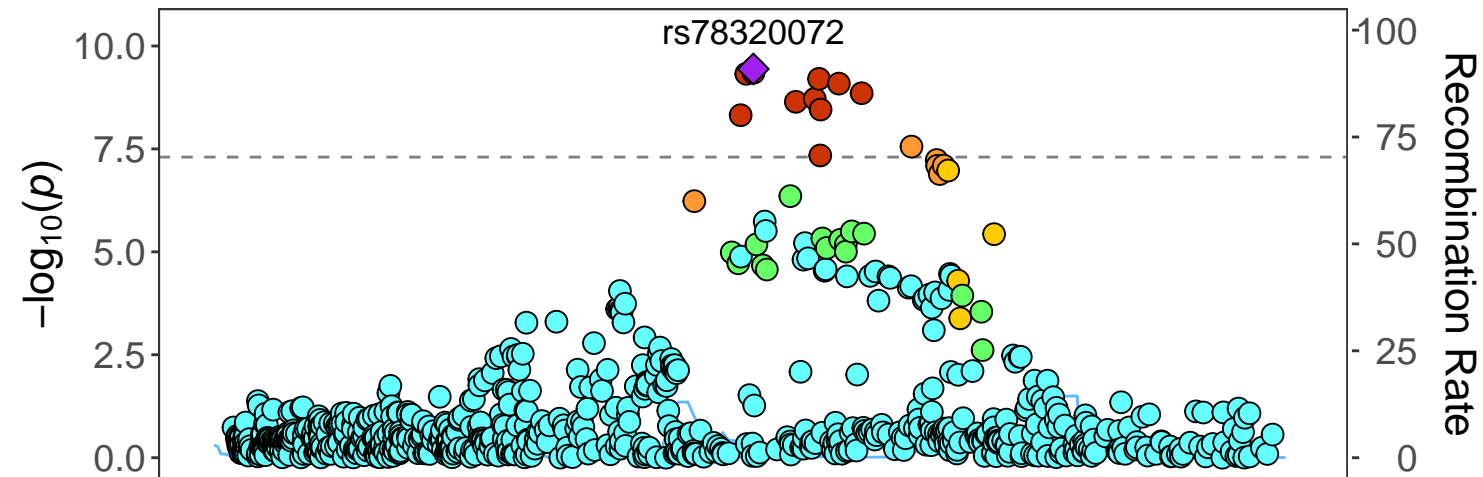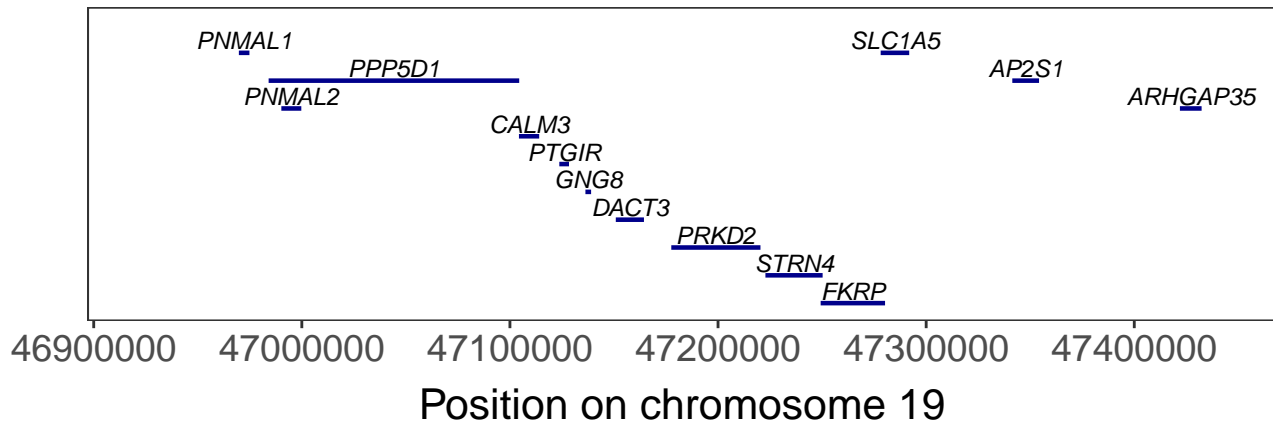

$r^2$     $\circ$  miss    $\circ$  0.0–0.2    $\circ$  0.2–0.4    $\circ$  0.4–0.6    $\circ$  0.6–0.8    $\circ$  0.8–1.0

Supplement: Supplementary file 5 — Supporting Information [file CTM2-16-e70732-s001.zip › LocusZoom/Sfig_rs78320072_locusZoom.pdf]

# LocusZoom plots of GWAS top lead SNP

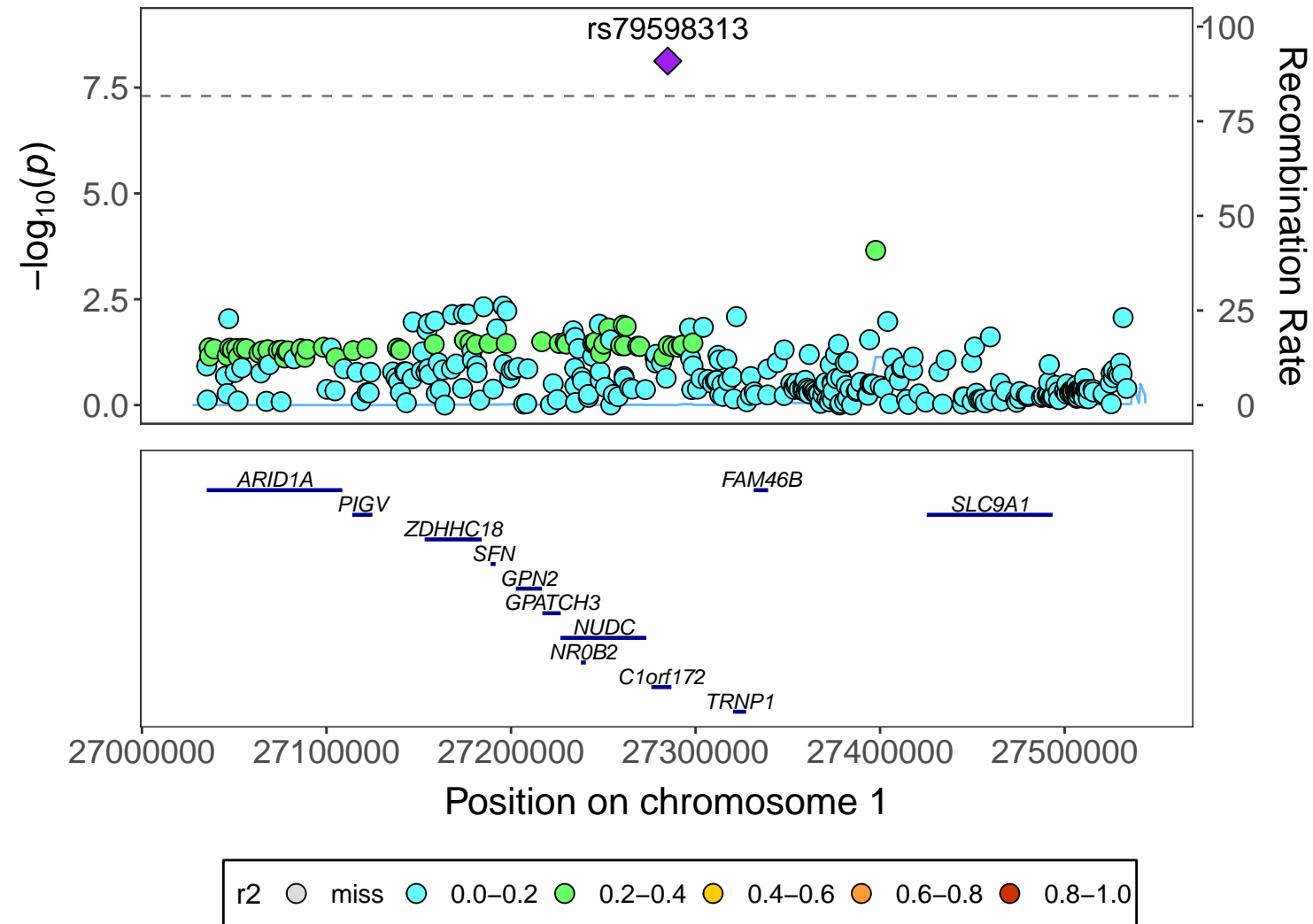

Supplement: Supplementary file 5 — Supporting Information [file CTM2-16-e70732-s001.zip › LocusZoom/Sfig_rs79598313_locusZoom.pdf]

# LocusZoom plots of GWAS top lead SNP

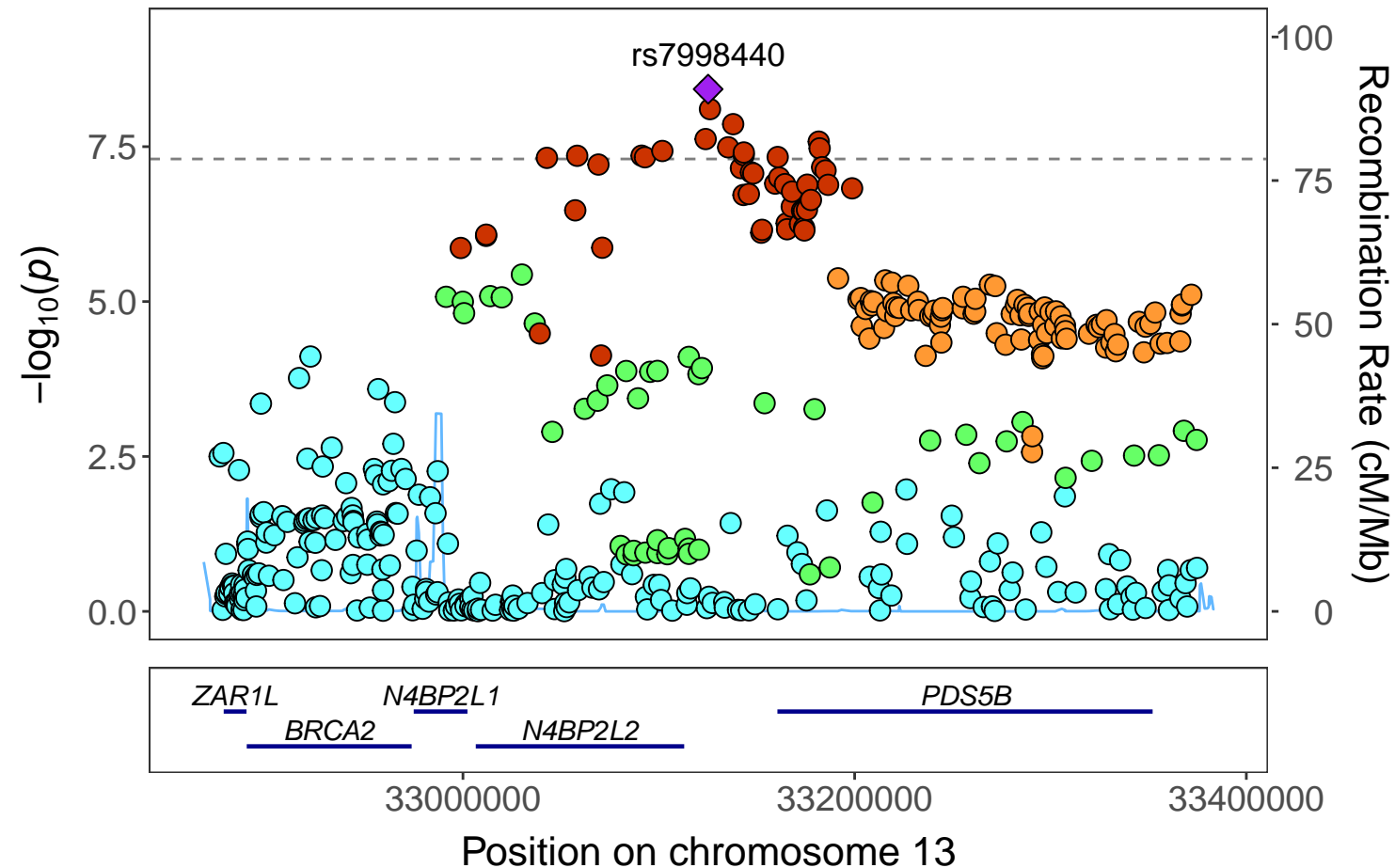

r2   miss   0.0–0.2   0.2–0.4   0.4–0.6   0.6–0.8   0.8–1.0

Supplement: Supplementary file 5 — Supporting Information [file CTM2-16-e70732-s001.zip › LocusZoom/Sfig_rs7998440_locusZoom.pdf]

# LocusZoom plots of GWAS top lead SNP

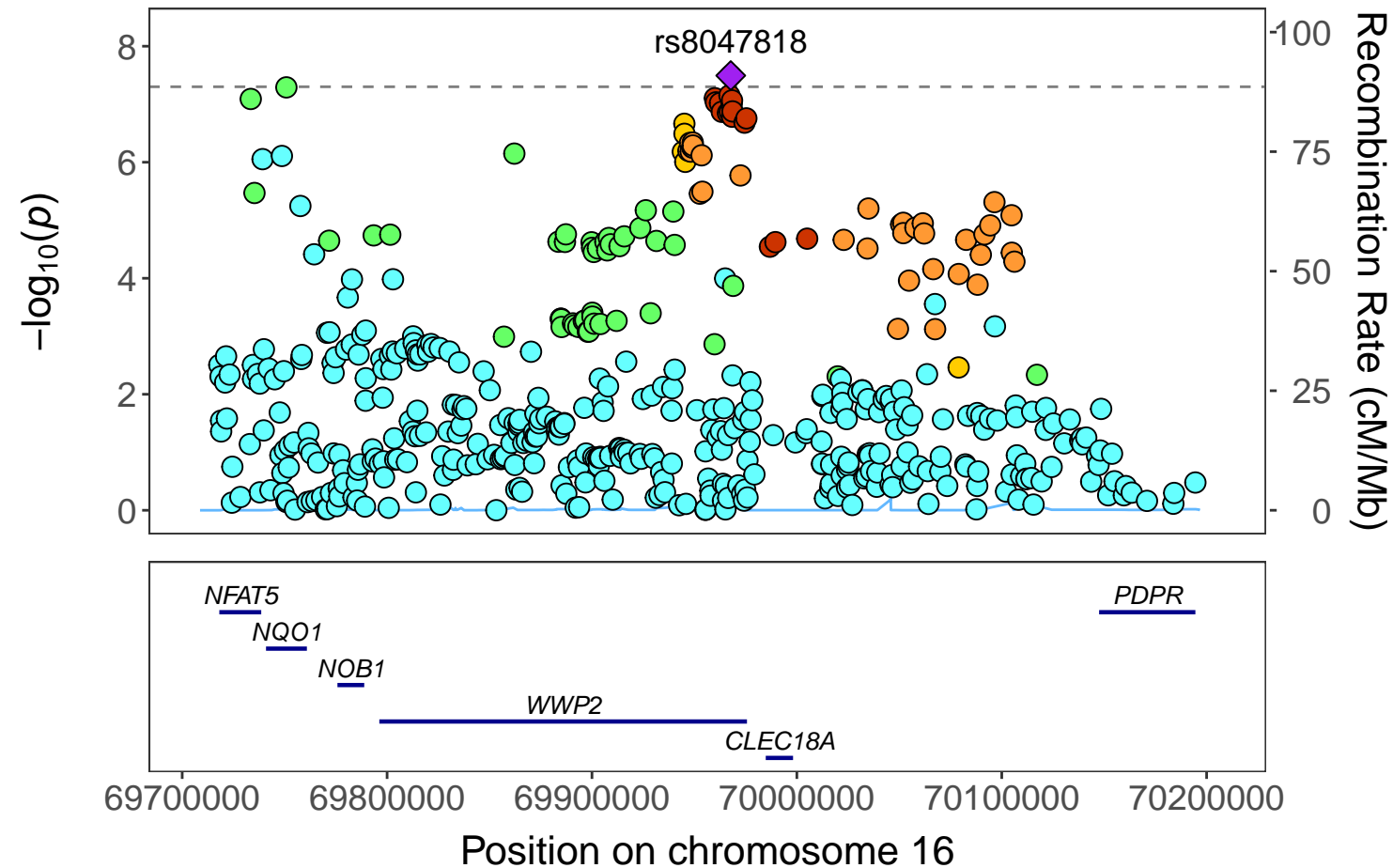

r2    miss    0.0–0.2    0.2–0.4    0.4–0.6    0.6–0.8    0.8–1.0

Supplement: Supplementary file 5 — Supporting Information [file CTM2-16-e70732-s001.zip › LocusZoom/Sfig_rs8047818_locusZoom.pdf]

# LocusZoom plots of GWAS top lead SNP

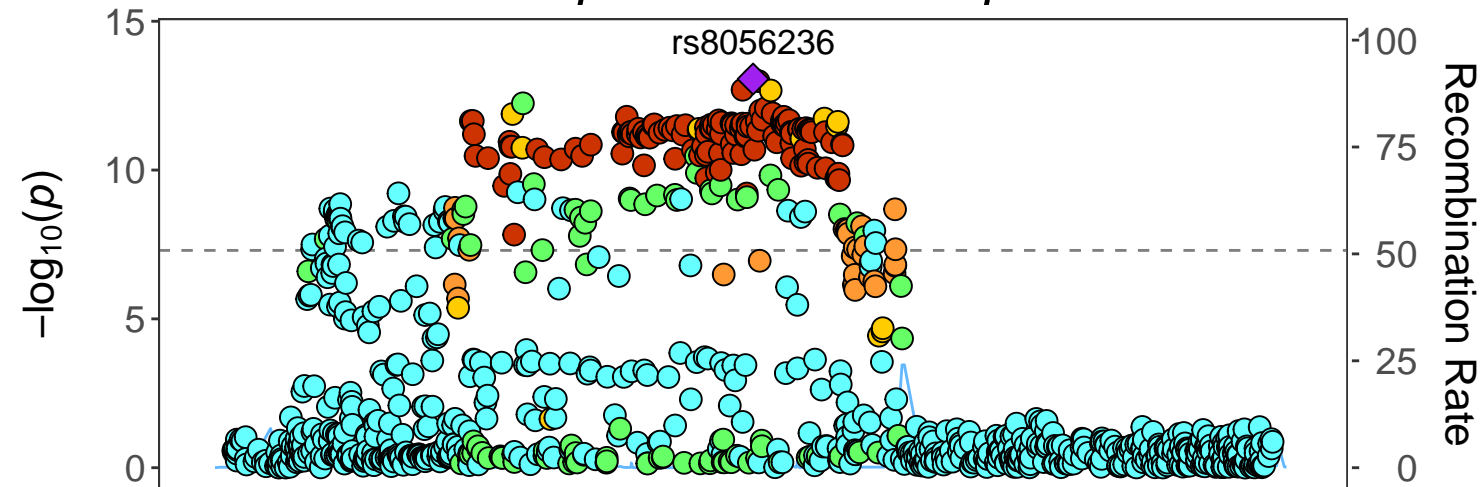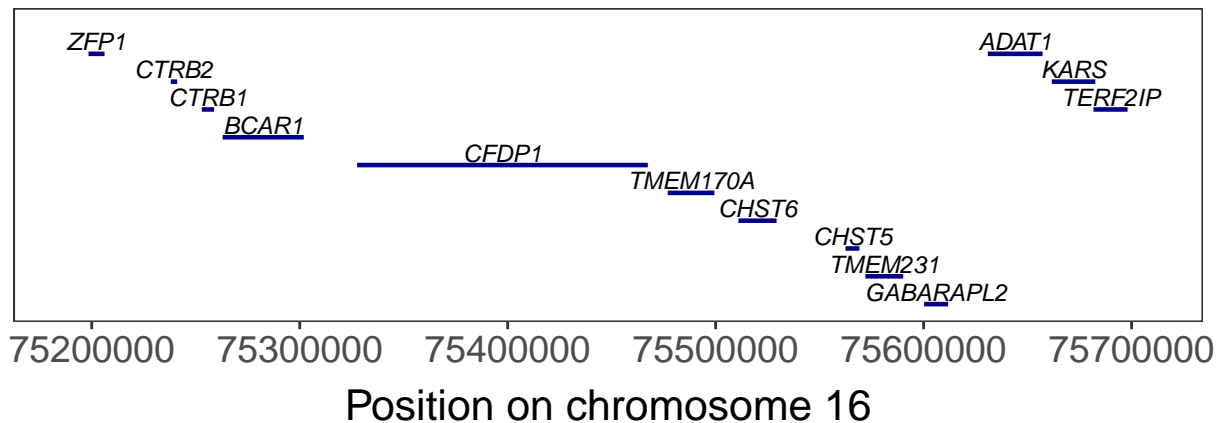

$r^2$     $\circ$  miss    $\circ$  0.0–0.2    $\circ$  0.2–0.4    $\circ$  0.4–0.6    $\circ$  0.6–0.8    $\circ$  0.8–1.0

Supplement: Supplementary file 5 — Supporting Information [file CTM2-16-e70732-s001.zip › LocusZoom/Sfig_rs8056236_locusZoom.pdf]

# LocusZoom plots of GWAS top lead SNP

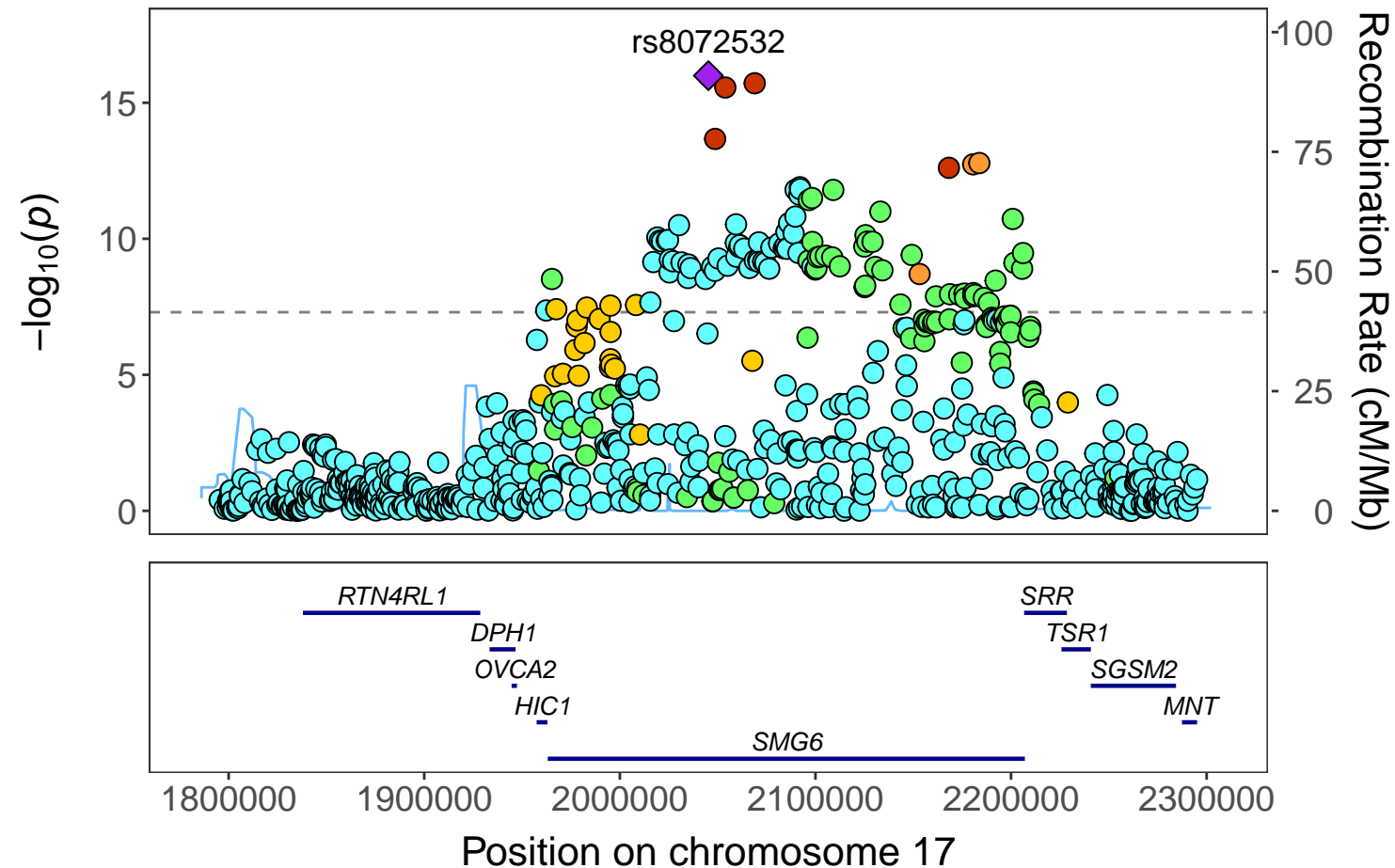

Supplement: Supplementary file 5 — Supporting Information [file CTM2-16-e70732-s001.zip › LocusZoom/Sfig_rs8072532_locusZoom.pdf]

# LocusZoom plots of GWAS top lead SNP

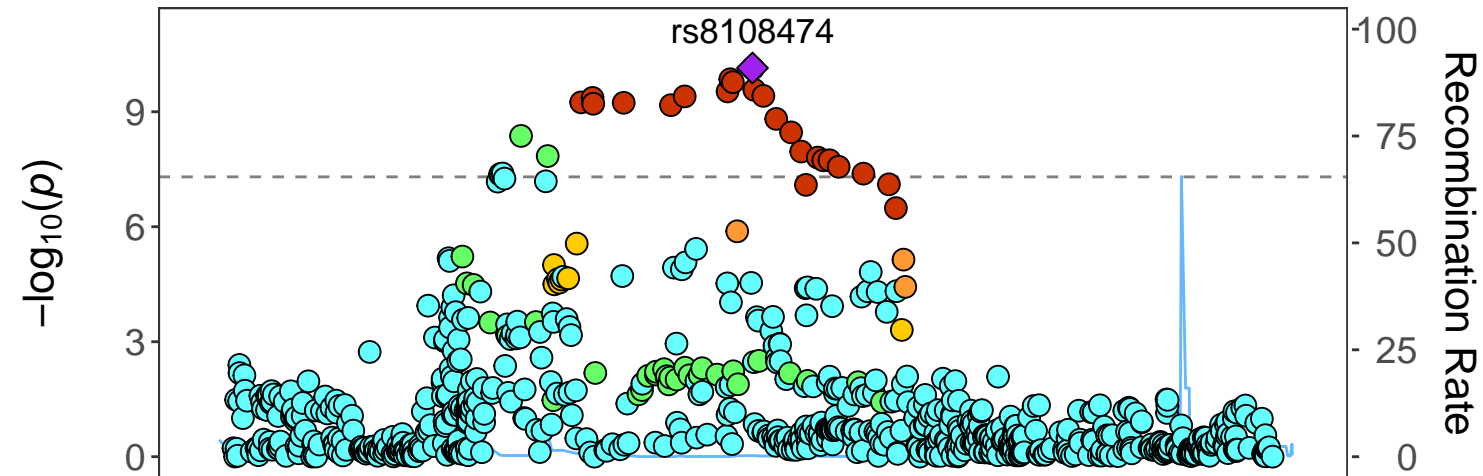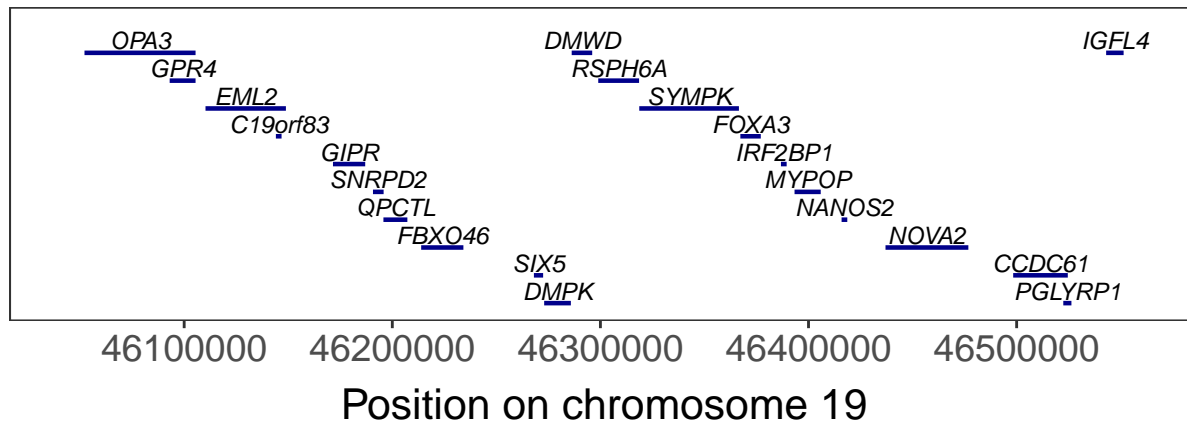

r2    ○   miss    ●   0.0–0.2    ●   0.2–0.4    ●   0.4–0.6    ●   0.6–0.8    ●   0.8–1.0

Supplement: Supplementary file 5 — Supporting Information [file CTM2-16-e70732-s001.zip › LocusZoom/Sfig_rs8108474_locusZoom.pdf]

# LocusZoom plots of GWAS top lead SNP

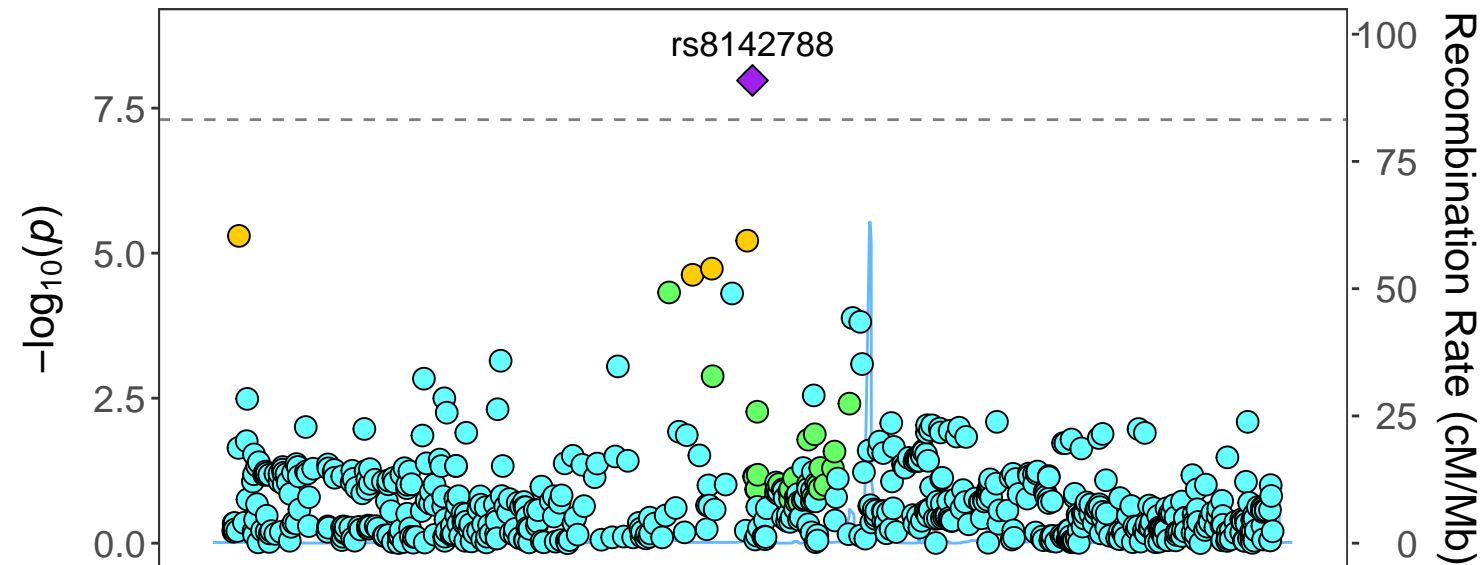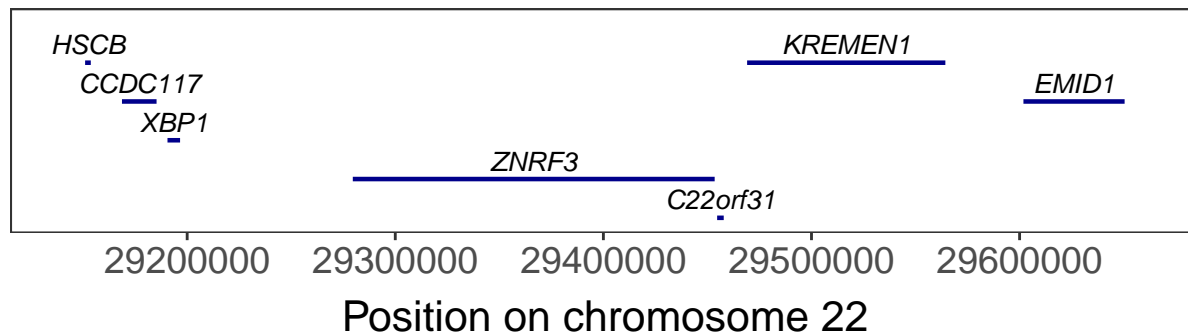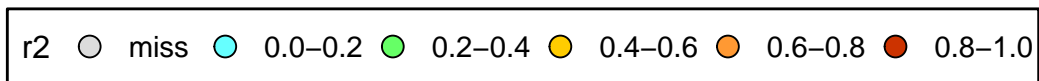

Supplement: Supplementary file 5 — Supporting Information [file CTM2-16-e70732-s001.zip › LocusZoom/Sfig_rs8142788_locusZoom.pdf]

# LocusZoom plots of GWAS top lead SNP

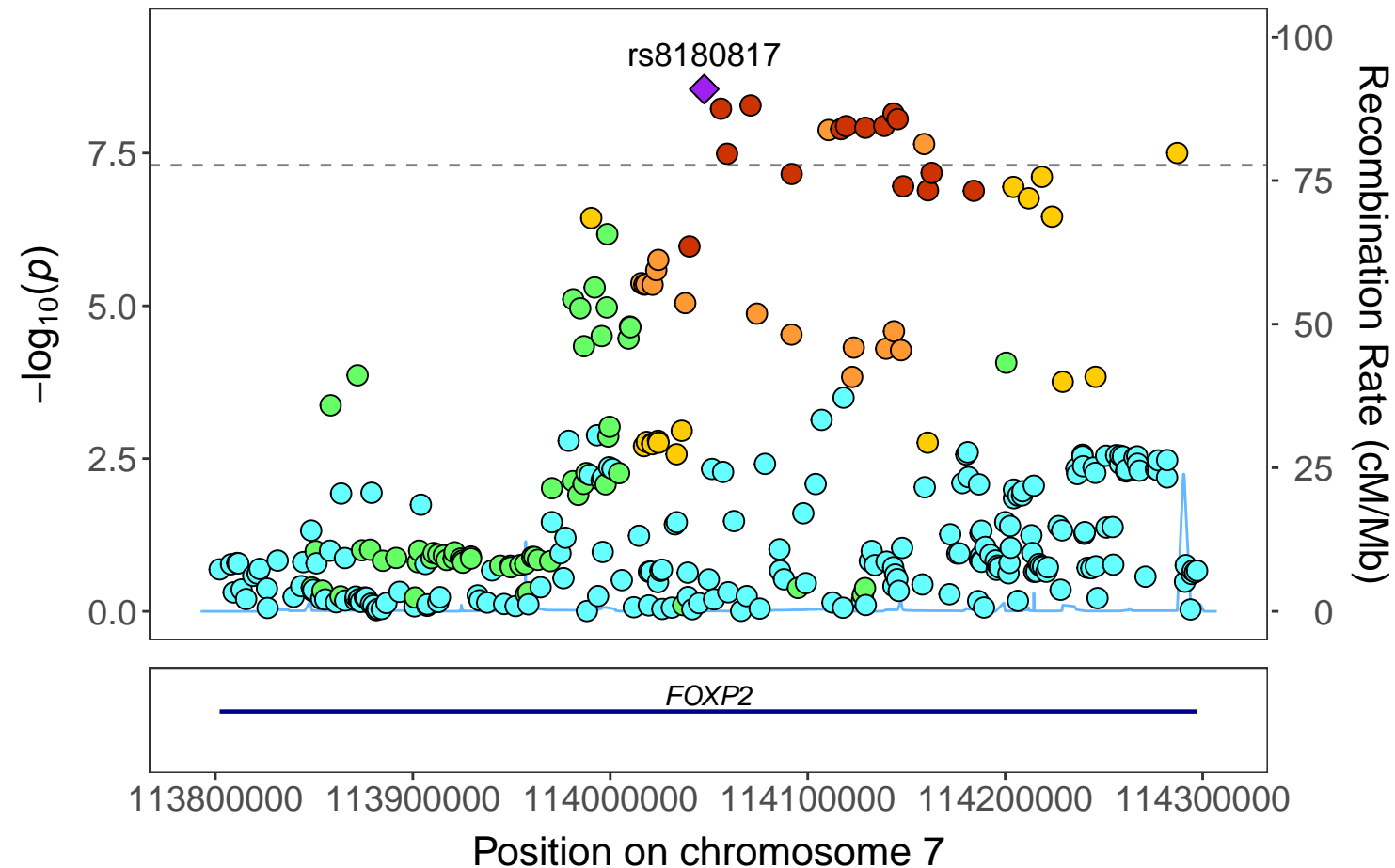

Supplement: Supplementary file 5 — Supporting Information [file CTM2-16-e70732-s001.zip › LocusZoom/Sfig_rs8180817_locusZoom.pdf]

# LocusZoom plots of GWAS top lead SNP

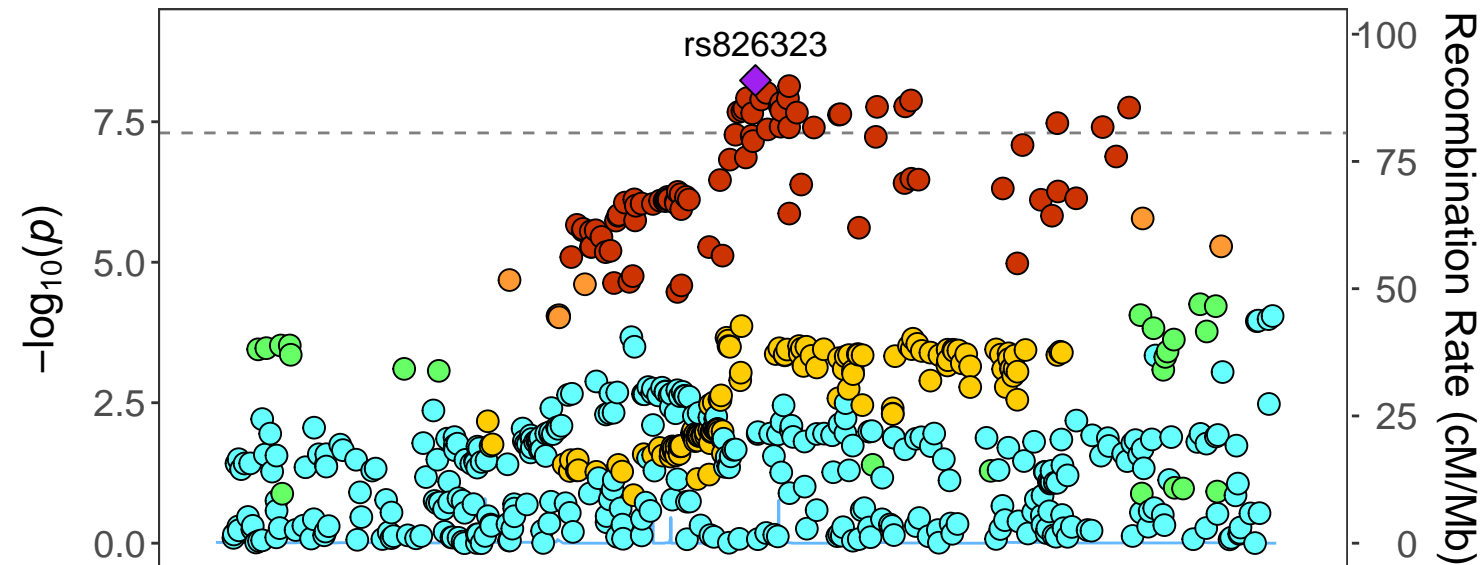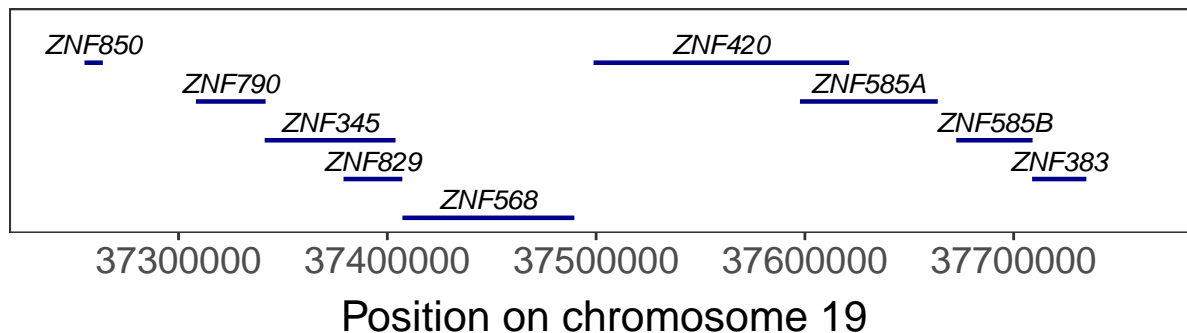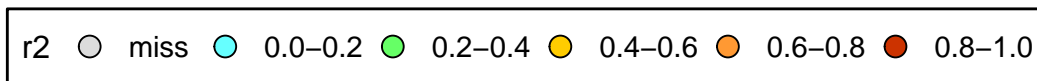

Supplement: Supplementary file 5 — Supporting Information [file CTM2-16-e70732-s001.zip › LocusZoom/Sfig_rs826323_locusZoom.pdf]

# LocusZoom plots of GWAS top lead SNP

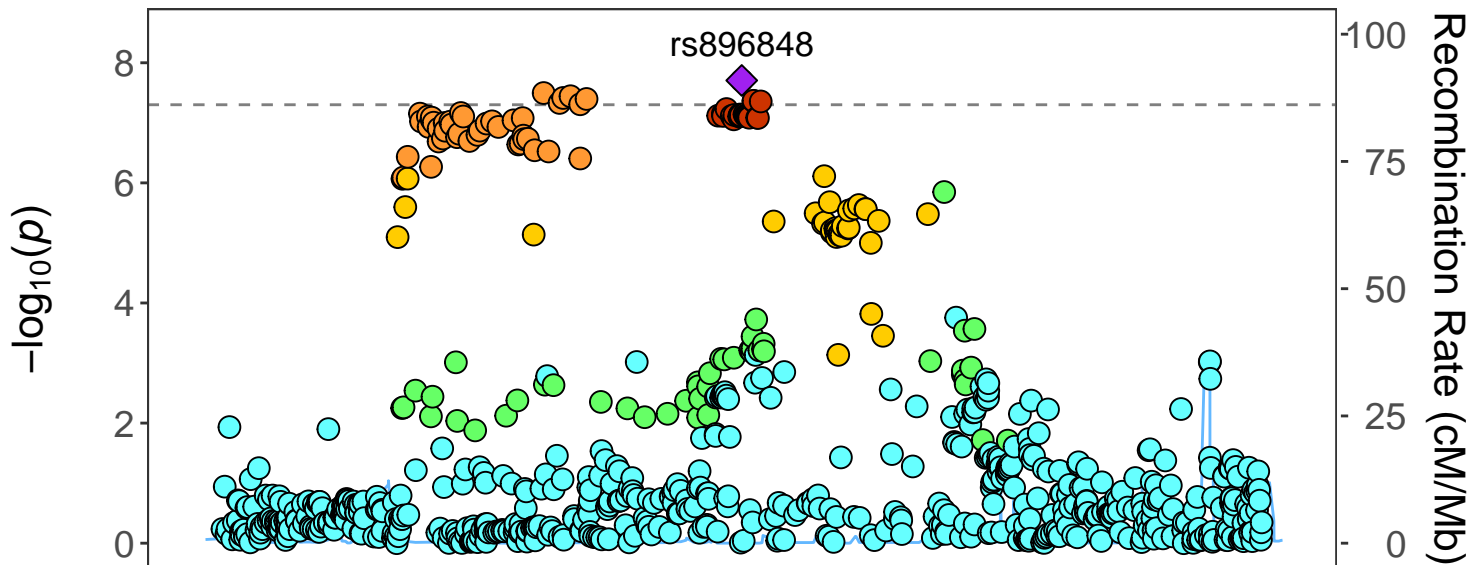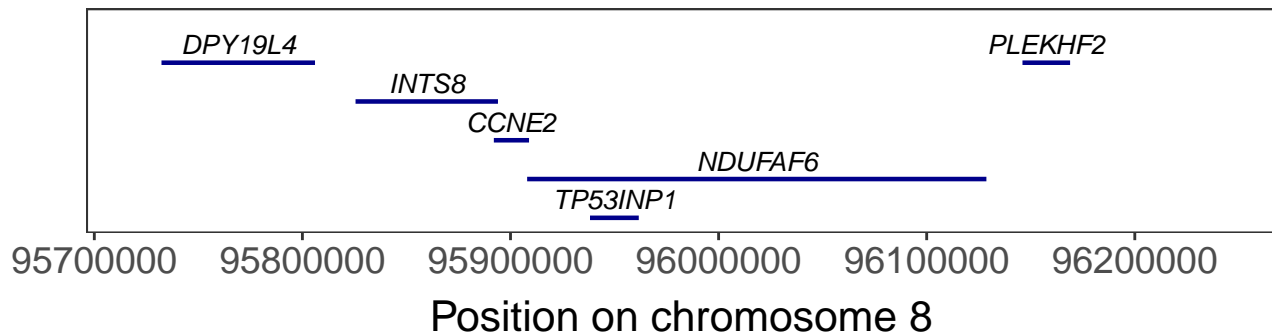

r2   miss   0.0-0.2   0.2-0.4   0.4-0.6   0.6-0.8   0.8-1.0

Supplement: Supplementary file 5 — Supporting Information [file CTM2-16-e70732-s001.zip › LocusZoom/Sfig_rs896848_locusZoom.pdf]
