# Supplementary material for: Precision medicine for atherosclerotic cardiovascular disease: Integrative genomics maps risk loci and AI‐predicted functional consequences
Source: Clin Transl Med. 2026 Jul 10;16(7):e70732. doi: 10.1002/ctm2.70732 (PMC13351343; doi:10.1002/ctm2.70732)
Supplement: Supplementary file 5 — Supporting Information [file CTM2-16-e70732-s001.zip › LocusZoom/Sfig_rs9606203_locusZoom.pdf]

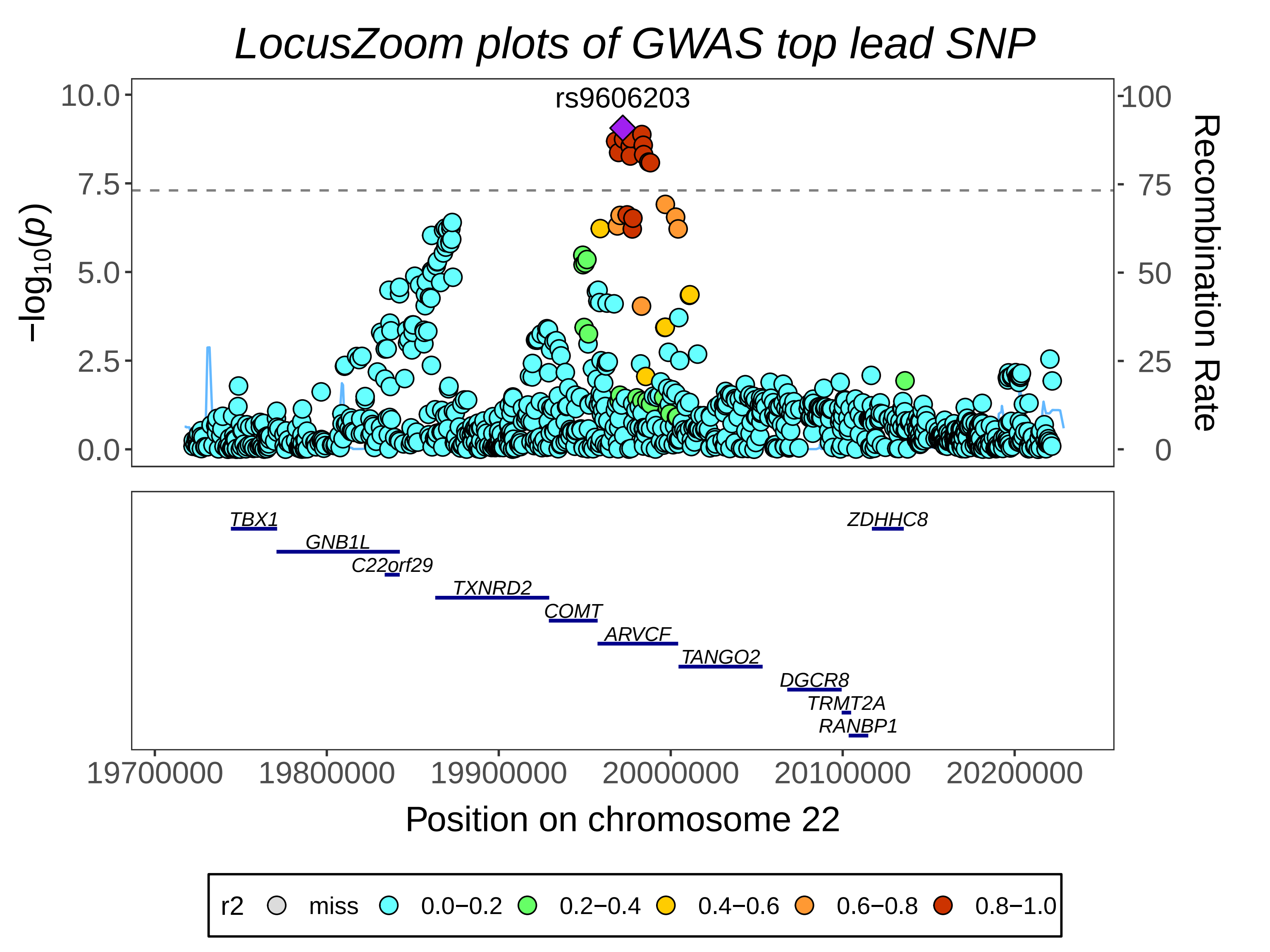
LocusZoom plots of GWAS top lead SNP
rs9606203
−log10(p)
Recombination Rate
10.0
7.5
5.0
2.5
0.0
100
75
50
25
0
TBX1
GNB1L
C22orf29
TXNRD2
COMT
ARVCF
TANGO2
DGCR8
TRMT2A
RANBP1
ZDHHC8
19700000
19800000
19900000
20000000
20100000
20200000
Position on chromosome 22
r2
miss
0.0−0.2
0.2−0.4
0.4−0.6
0.6−0.8
0.8−1.0
