# Supplementary material for: Precision medicine for atherosclerotic cardiovascular disease: Integrative genomics maps risk loci and AI‐predicted functional consequences
Source: Clin Transl Med. 2026 Jul 10;16(7):e70732. doi: 10.1002/ctm2.70732 (PMC13351343; doi:10.1002/ctm2.70732)
Supplement: Supplementary file 5 — Supporting Information [file CTM2-16-e70732-s001.zip › LocusZoom/Sfig_rs9827970_locusZoom.pdf]

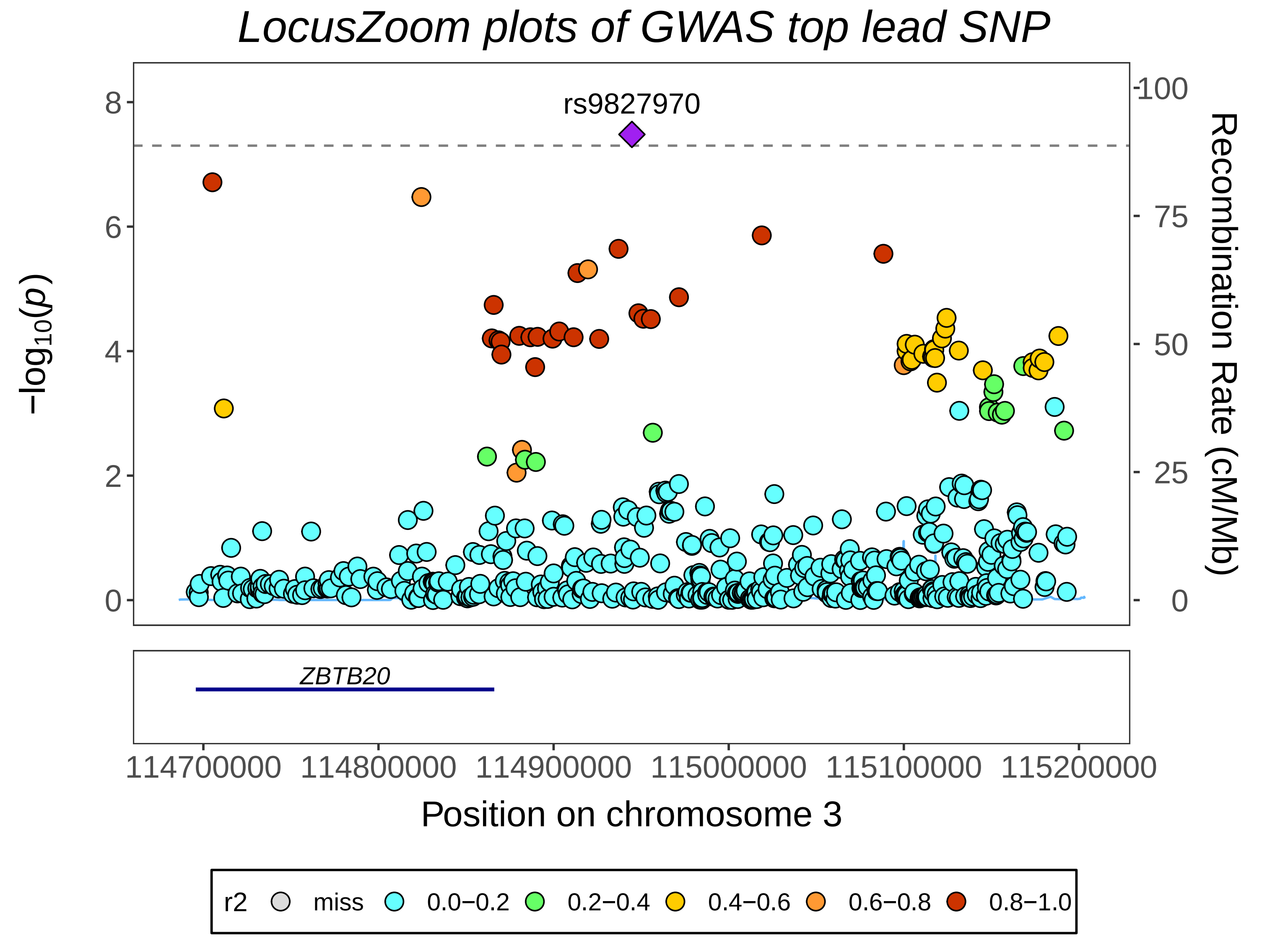

LocusZoom plots of GWAS top lead SNP
rs9827970
−log10(p)
8
6
4
2
0
Recombination Rate (cM/Mb)
100
75
50
25
0
ZBTB20
114700000
114800000
114900000
115000000
115100000
115200000
Position on chromosome 3
r2
miss
0.0−0.2
0.2−0.4
0.4−0.6
0.6−0.8
0.8−1.0
