# Supplementary material for: Precision medicine for atherosclerotic cardiovascular disease: Integrative genomics maps risk loci and AI‐predicted functional consequences
Source: Clin Transl Med. 2026 Jul 10;16(7):e70732. doi: 10.1002/ctm2.70732 (PMC13351343; doi:10.1002/ctm2.70732)
Supplement: Supplementary file 5 — Supporting Information [file CTM2-16-e70732-s001.zip › LocusZoom/Sfig_rs9943599_locusZoom.pdf]

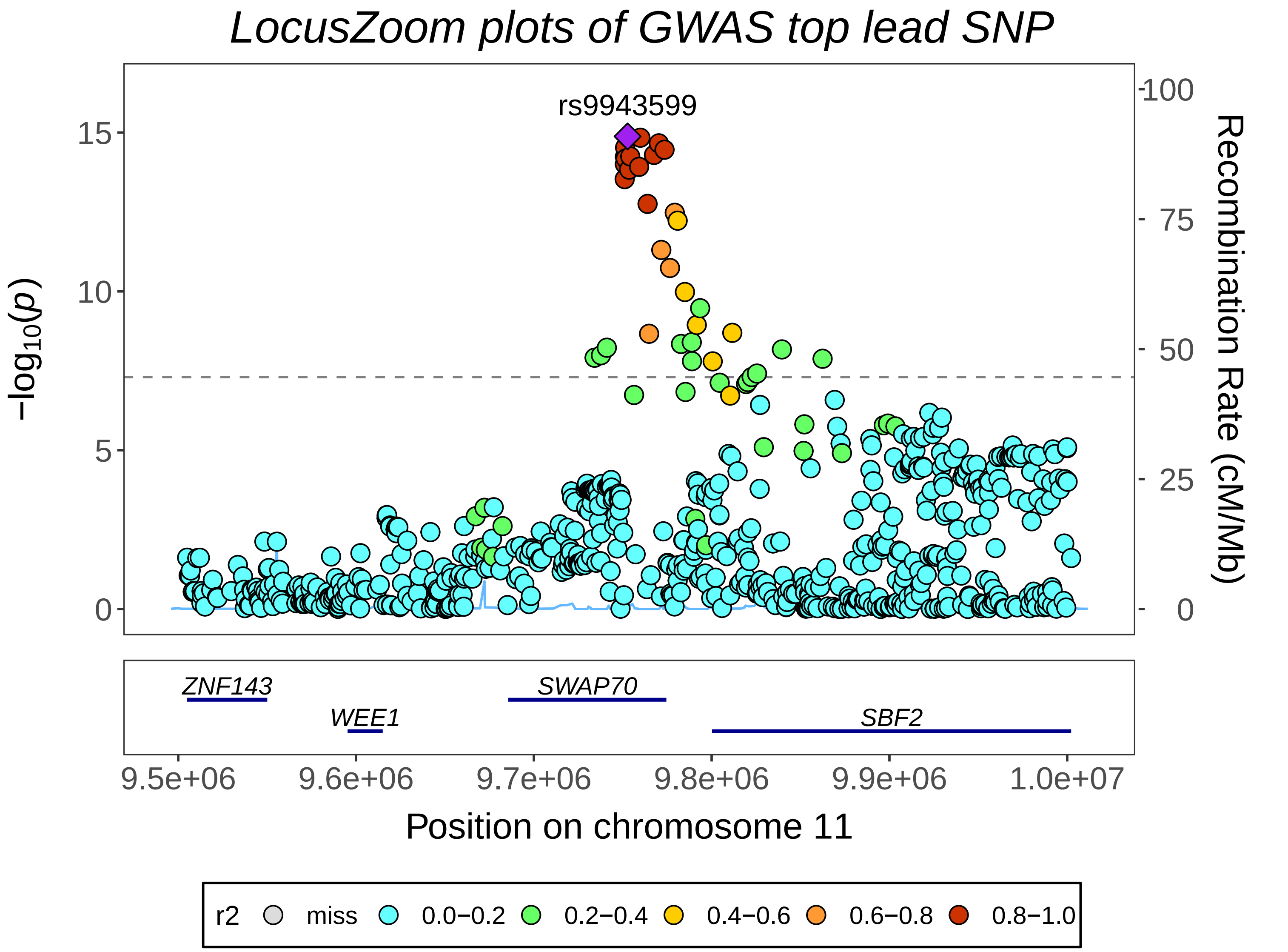

LocusZoom plots of GWAS top lead SNP
rs9943599
−log10(p)
0
5
10
15
Recombination Rate (cM/Mb)
0
25
50
75
100
ZNF143
WEE1
SWAP70
SBF2
9.5e+06
9.6e+06
9.7e+06
9.8e+06
9.9e+06
1.0e+07
Position on chromosome 11
r2
miss
0.0−0.2
0.2−0.4
0.4−0.6
0.6−0.8
0.8−1.0
