# Supplementary material for: Precision medicine for atherosclerotic cardiovascular disease: Integrative genomics maps risk loci and AI‐predicted functional consequences
Source: Clin Transl Med. 2026 Jul 10;16(7):e70732. doi: 10.1002/ctm2.70732 (PMC13351343; doi:10.1002/ctm2.70732)
Supplement: Supplementary file 5 — Supporting Information [file CTM2-16-e70732-s001.zip › LocusZoom/Sfig_rs9945890_locusZoom.pdf]

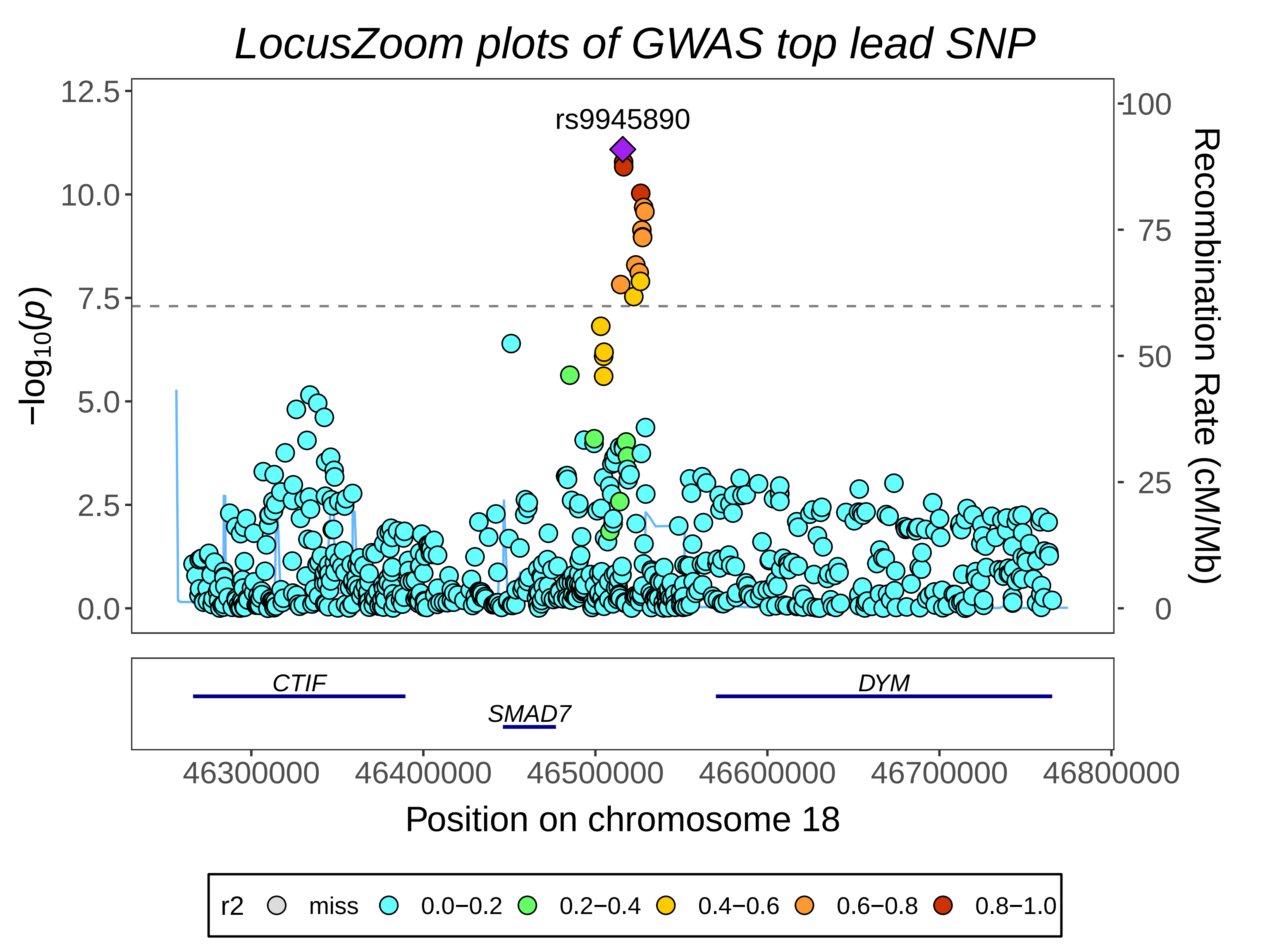
LocusZoom plots of GWAS top lead SNP
rs9945890
12.5
10.0
7.5
5.0
2.5
0.0
$-\log_{10}(p)$
100
75
50
25
0
Recombination Rate (cM/Mb)
CTIF
SMAD7
DYM
46300000
46400000
46500000
46600000
46700000
46800000
Position on chromosome 18
r2
miss
0.0−0.2
0.2−0.4
0.4−0.6
0.6−0.8
0.8−1.0
