# Supplementary material for: Precision medicine for atherosclerotic cardiovascular disease: Integrative genomics maps risk loci and AI‐predicted functional consequences
Source: Clin Transl Med. 2026 Jul 10;16(7):e70732. doi: 10.1002/ctm2.70732 (PMC13351343; doi:10.1002/ctm2.70732)
Supplement: Supplementary file 5 — Supporting Information [file CTM2-16-e70732-s001.zip › LocusZoom/Sfig_rs998584_locusZoom.pdf]

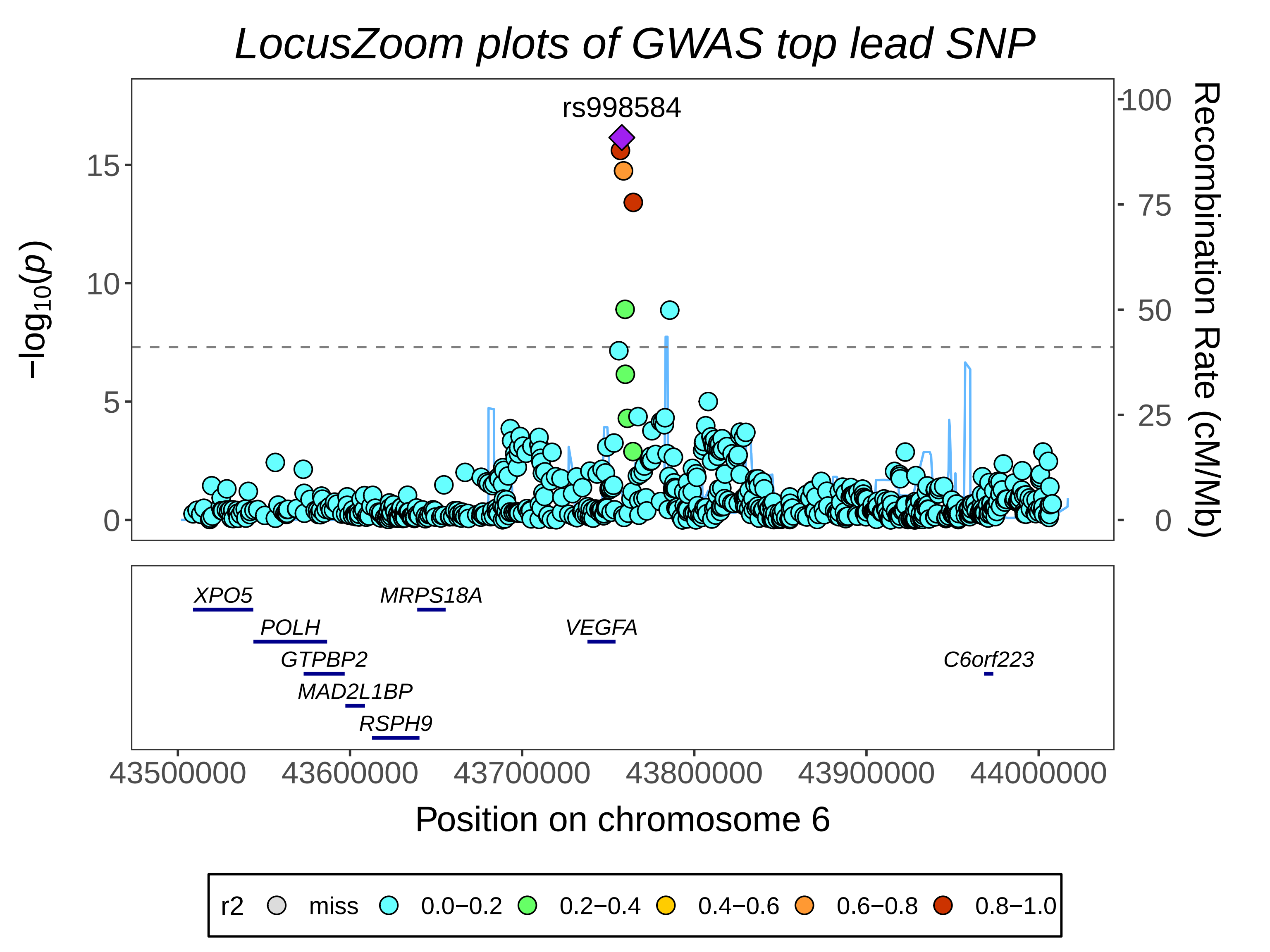

LocusZoom plots of GWAS top lead SNP
rs998584
15
10
5
0
-log10(p)
100
75
50
25
0
Recombination Rate (cM/Mb)
XPO5
MRPS18A
POLH
VEGFA
GTPBP2
C6orf223
MAD2L1BP
RSPH9
43500000
43600000
43700000
43800000
43900000
44000000
Position on chromosome 6
r2
miss
0.0−0.2
0.2−0.4
0.4−0.6
0.6−0.8
0.8−1.0
